# High resolution discovery and confirmation of copy number variants in 90 Yoruba Nigerians

**DOI:** 10.1186/gb-2009-10-11-r125

**Published:** 2009-11-09

**Authors:** Hajime Matsuzaki, Pei-Hua Wang, Jing Hu, Rich Rava, Glenn K Fu

**Affiliations:** 1 Affymetrix, Inc., 3420 Central Expressway, Santa Clara, CA 95051, USA

## Abstract

**Background:**

Copy number variants (CNVs) account for a large proportion of genetic variation in the genome. The initial discoveries of long (> 100 kb) CNVs in normal healthy individuals were made on BAC arrays and low resolution oligonucleotide arrays. Subsequent studies that used higher resolution microarrays and SNP genotyping arrays detected the presence of large numbers of CNVs that are < 100 kb, with median lengths of approximately 10 kb. More recently, whole genome sequencing of individuals has revealed an abundance of shorter CNVs with lengths < 1 kb.

**Results:**

We used custom high density oligonucleotide arrays in whole-genome scans at approximately 200-bp resolution, and followed up with a localized CNV typing array at resolutions as close as 10 bp, to confirm regions from the initial genome scans, and to detect the occurrence of sample-level events at shorter CNV regions identified in recent whole-genome sequencing studies. We surveyed 90 Yoruba Nigerians from the HapMap Project, and uncovered approximately 2,700 potentially novel CNVs not previously reported in the literature having a median length of approximately 3 kb. We generated sample-level event calls in the 90 Yoruba at nearly 9,000 regions, including approximately 2,500 regions having a median length of just approximately 200 bp that represent the union of CNVs independently discovered through whole-genome sequencing of two individuals of Western European descent. Event frequencies were noticeably higher at shorter regions < 1 kb compared to longer CNVs (> 1 kb).

**Conclusions:**

As new shorter CNVs are discovered through whole-genome sequencing, high resolution microarrays offer a cost-effective means to detect the occurrence of events at these regions in large numbers of individuals in order to gain biological insights beyond the initial discovery.

## Abstract 2

Most microRNAs have a stronger inhibitory effect in estrogen receptor-negative than in estrogen receptor-positive breast cancers.

## Background

Genetic differences between individuals occur at many levels, starting with single nucleotide polymorphisms (SNPs) [1], short insertions and deletions of several nucleotides (indels) [2], and extending out to copy number variants (CNVs) that span several orders of magnitude in length [3]. A thorough cataloging of genetic variations in the human genome is well underway, as evidenced by the HapMap Project [1] and 1,000 Genomes Project [4], and data repositories such as dbSNP [5] and the Database of Genomic Variants (DGV) [6]. The ability to genotype large numbers of individuals in various study cohorts at large numbers of known loci has in turn led to significant associations between specific genetic differences and phenotypic differences, which often manifest as complex disorders. Recent notable studies have associated SNP markers with bipolar disorder, coronary artery disease, Crohn's disease, hypertension, rheumatoid arthritis, type 1 diabetes, and type 2 diabetes [7], and CNVs with autism and schizophrenia [8-10].

Progressively higher resolution microarrays, starting with earlier low resolution bacterial artificial chromosome (BAC) arrays followed by commercially available array comparative genome hybridization (CGH) and SNP genotyping arrays, have steadily driven the discovery of new CNVs and have refined the boundaries of earlier reported CNVs. Specifically, the earliest CNVs described by Sebat *et al*. [11] and Iafrate *et al*. [6], using BAC arrays and lower resolution oligonucleotide arrays, had median lengths of approximately 222 kb and approximately 156 kb, respectively. Later, Redon *et al*. [12] used both BAC arrays and SNP genotyping arrays from Affymetrix to report CNVs with median lengths of approximately 234 kb and approximately 63 kb, respectively. More recent examples are the Perry *et al*. [13] study, which used Agilent high resolution CGH arrays, the McCarroll *et al*. [14] study, which used the Affymetrix SNP 6.0 array, and the Wang *et al*. [15] study, which used data from Illumina BeadChips. The Perry *et al*. [13] study examined known regions in the DGV (November 2006) at approximately 1 kb resolution, and refined the lengths of over 1,000 CNVs to a revised median length of approximately 10.2 kb. The Wang *et al*. [15] study analyzed genome-wide SNP genotype data having median inter-SNP distance of approximately 3 kb from over a hundred individuals to detect CNVs having median lengths of approximately 12 kb. The McCarroll *et al*. [14] study examined the entire genome (as represented in the whole-genome sampling of *Nsp*I and *Sty*I restriction fragments) at approximately 2-kb resolution, and reported > 1,300 CNVs having a median length of approximately 7.4 kb.

Here in this study, we set out to demonstrate the benefits, as well as limitations, of Affymetrix oligonucleotide arrays with higher resolution than previously available arrays, first in unbiased whole-genome scans to discover CNV regions, and subsequently in localized regions to determine sample-level CNV calls. Our custom arrays were manufactured using standard Affymetrix processes [16], but with phosphoramidite nucleosides bearing an improved protecting group to provide for more efficient photolysis and chain extension [17], which enabled the synthesis of longer probes. We first used our genome-scan arrays to examine the entire genome with uniform coverage at a resolution of approximately 200 bp. We designed a set of three custom oligonucleotide whole-genome scan arrays that span the entire non-repetitive portion of the human genome. Each of the genome-scan arrays consists of over 10 million 49-nucleotide long probes that are spaced at a median distance of approximately 200 bp apart along the chromosomes. The set of 90 Yoruba Nigerians from the HapMap Project [1] was chosen for the scans because they represent an anthropologically early population likely to be harboring a fair proportion of common and more older CNVs, similar to the occurrence of common SNPs [1]. A number of previous CNV studies also used some or all of the Yoruba individuals, making it possible to compare event calls reported in the literature with those observed in our work. Additionally, because the 90 Yoruba individuals are each members of 30 family trios, inheritance patterns of the observed and reported events can be measures of accuracy and event call completeness.

A fourth custom oligonucleotide array was designed to confirm putative CNV regions identified from the initial genome scans, as well as subsets of CNVs reported in the DGV (November 2008), including those reported by Perry *et al*. [13], Wang *et al*. [15], and McCarroll *et al*. [14], and to determine sample-level event occurrence. Additionally, we were particularly interested in observing events in the 90 Yoruba at shorter CNVs discovered through the whole-genome sequencing of two individuals. The design of our CNV-typing array prioritized CNVs reported in the landmark Levy *et al*. [18] and Wheeler *et al*. [19] studies, which contributed the initial whole-genome sequences of two individuals of Western European descent. Since the Bentley *et al*. [20] and Wang *et al*. [21] studies were added to the DGV after the design of the CNV-typing array, the shorter regions discovered by whole-genome sequencing of one of the Yoruba and an Asian were not included. The CNV-typing array consists of approximately 2.4 million 60-nucleotide long probes concentrated at the known and putative CNVs, at variable spacing as close as 10 bp apart.

Our arrays are essentially tiling designs with probe sequences picked from the reference genome (build 36), and are more similar to early BAC and Agilent CGH arrays than to recent genotyping arrays, such as the Affymetrix SNP 6.0 or the Illumina BeadChips, which generate allele-specific signals (with the exception of subsets of non-genotyping copy number probes). To observe copy number events on our arrays, we processed our probe signals with circular binary segmentation (CBS) [22], a CNV detection algorithm originally developed for BAC arrays but also suitable for our tiling arrays.

## Results

### Whole-genome scan

**Figure 1:**
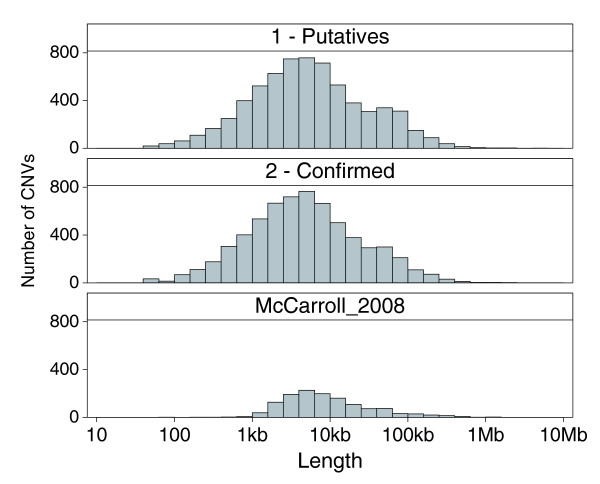
Length distributions. The top two panels show the length distributions of putative and confirmed CNVs, respectively. The smaller second peak in the putative and to a lesser degree in the confirmed CNVs reflects the longer CNVs identified in the secondary smoothed segmentation analysis. For comparison, the approximately 1,300 CNVs reported in the McCarroll *et al*. [14] study, which used Affymetrix SNP 6.0 arrays on 270 HapMap individuals including the 90 Yoruba, are shown in the bottom panel. Lengths are shown in log scale.

DNA samples from each of the 90 Yoruba individuals was whole-genome amplified, randomly fragmented, end-labeled with biotin, and then hybridized to the three genome-scan arrays (see Materials and methods). Probe signals were quantile normalized [23] across the 90 individuals separately for each design; then for each individual, changes in signal log ratios based on median signals from > 90 arrays were detected as gain and loss events using CBS [22] (see Materials and methods). Probes are sequentially inter-digitated across the three genome-scan arrays, allowing the three arrays to be treated as technical replicate experiments. Segments above or below the detection thresholds must be observed in at least two of the three designs before assigning a CNV event to an individual. In total, 6,578 putative CNV regions were identified in the whole-genome scans of the 90 Yoruba, where a putative region had at least one detected event among the individuals; a subset of 3,850 regions showed events in at least two individuals (Table 1). Based on the longest detected events at each region, the putative CNVs had a median length of approximately 4.9 kb, with 25th and 75th percentiles ranging from 1.7 kb to 15.7 kb, respectively. In order to capture the wide spectrum of CNV lengths, two separate segmentation analyses were run: the first using all probes (no smoothing) for the shorter ranges, and a secondary smoothed analysis to fill out the longer ranges (see Materials and methods). The median lengths were approximately 4 kb and approximately 70 kb, respectively, with the smoothed analysis accounting for only approximately 11% of the putative CNVs (Table 1). The length distribution of the putative CNVs is mostly symmetric about the median, but with a noticeable bias toward longer lengths, and a smaller second peak reflecting the longer regions from the smoothed segmentation analysis (Figure 1). The genome locations (build 36) and estimated lengths of the putative CNVs are listed in Additional data file 2.

**Table 1:** Summary of putative and confirmed CNVs

	Putative CNVs	High conf	Singleton	CBS all probes	CBS smoothed	Confirmed CNVs	Confirmed high conf	Confirmed singleton
Parent set		Putatives	Putatives	Putatives	Putatives	Putatives	Putative high conf	Putative singleton
								
Number of CNVs	6,578	3,850	2,728	5,842	736	**6,368**	3,799	2,569
% of parent set		58.5%	41.5%	88.8%	11.2%	**96.8%**	98.7%	94.2%
								
Median length	4.9 kb	5.9 kb	3.7 kb	4.0 kb	70.7 kb	**4.4 kb**	5.3 kb	3.1 kb
25th percentile	1.7 kb	2.3 kb	1.1 kb	1.5 kb	48.5 kb	1.5 kb	2.1 kb	1.0 kb
75th percentile	15.7 kb	19.0 kb	12.0 kb	9.8 kb	105.9 kb	13.2 kb	16.8 kb	9.1 kb
								
DGV overlap	3,780	2,587	1,193	3,346	434	**3,678**	2,551	1,127
% DGV	57.5%	67.2%	43.7%	57.3%	59.0%	**57.8%**	67.1%	43.9%
Med len in DGV	6.6 kb	7.6 kb	4.5 kb	5.2 kb	77.0 kb	**5.8 kb**	6.8 kb	3.9 kb
								
Novel CNVs	2,798	1,263	1,535	2,496	302	**2,690**	1,248	1,442
Med len novel	3.4 kb	3.6 kb	3.2 kb	2.8 kb	64.5 kb	**3.0 kb**	3.2 kb	2.6 kb

Putative CNVs are regions where at least one event was observed in the initial genome scan; confirmed CNVs are a subset of putative CNVs where at least one event was observed on the CNV-typing array. 'High conf' (high confidence) refers to putative CNVs that had events observed in at least two Yoruba, while singletons are putative CNVs with observed events in only one Yoruba. 'CBS all probes' refers to putative CNVs identified in the segmentation analysis using all probes on the genome-scan arrays, while 'CBS smoothed' refers to generally longer CNVs identified in smoothed segmentation analysis. At least 5% of a CNV region was required to overlap a record from the DGV (March 2009). Med len, median length.

Of the 3,850 putative CNVs having events observed in at least two individuals (defined as high confidence), approximately 67% overlapped at least one record in the DGV (March 2009), while only approximately 44% of the remaining regions having an event in only one individual (singletons) overlapped a DGV record (Table 1). Overlap is defined as greater than 5% of a putative region coinciding with a DGV record, not including inversions and records with lengths less than 100 bp. The minimum requirement of 5% overlap with DGV records was set low to accommodate a wide range of differences in resolutions between previous studies and our genome-scan. Since the union of DGV records (March 2009) covers a fair proportion of the genome (approximately 30%), a > 5% overlap does not necessarily validate a region, but serves as a starting point for comparison with previous studies. The high resolution of the genome-scan arrays revealed several instances of multiple smaller CNVs lying within regions that were earlier reported as one longer CNV in studies using lower resolution methods. Two such examples are shown in Figure S2 in Additional data file 1; the first is a 200-kb region with at least four CNVs and the second is a 20-kb region with two CNVs. These example regions overlap multiple DGV records from earlier studies such as Redon *et al*. [12], and more recent higher resolution studies such as Perry *et al*. [13]. The putative CNVs observed in the 90 Yoruba more closely match the shorter DGV records from the newer studies (Figure S2 in Additional data file 1).

**Figure 2:**
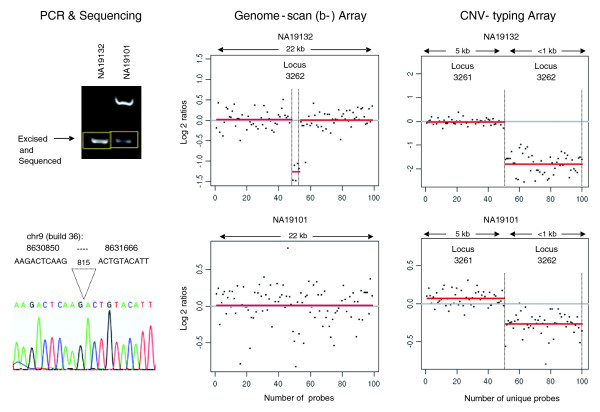
Examples of loss events detected by segmentation analysis [22] in two Yoruba DNAs, NA19132 and NA19101, at putative CNV locus_id 3262. PCR across the putative breakpoint of the events showed truncated bands from both DNAs, which were excised and sequenced. The sequences of the truncated amplicons were mapped on build 36 to determine the precise breakpoints, which corresponded to identical 815-bp deletions in both DNAs. Although the homozygous deletion in NA19132 was detected on the genome-scan arrays, the one copy loss in NA19101 was missed. The red lines in the log2 ratio plots indicate the segments detected by CBS. Although not shown, the results from the a- and c- genome-scan arrays were nearly identical to the b-design. The events in both DNAs, however, were detected on the CNV-typing array. The CNV-typing array showed no events in the preceding CNV locus_id, 3261, approximately 350 kb upstream on chromosome 9. The log2 ratio (y-axis) scales are different between the genome-scan array and CNV-typing array, and reflect a higher response in the latter.

**Figure 3:**
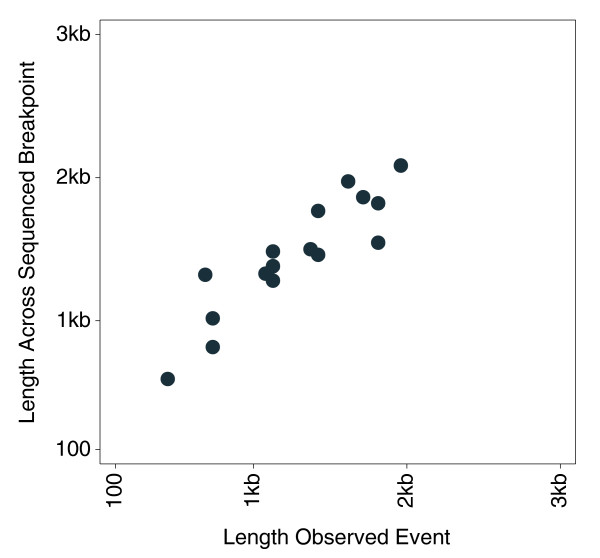
Results of breakpoint mapping by sequencing are compared with observed event lengths. Lengths are shown in linear scale.

To experimentally validate a sampling of the putative CNVs, we randomly selected observed events between 400 bp and 10 kb for PCR or quantitative PCR (qPCR). PCR primers were designed to amplify across putative breakpoints, while primers for qPCR were designed within gain regions. Figure 2 shows an example of loss events in two Yoruba DNAs, NA19132 and NA19101, which appear as the shorter PCR amplicons in the electrophoresis gel. The amplicon bands were excised from the gel and sequenced to precisely map breakpoints, which corresponded to identical 815-bp deletions in both DNAs. This process was carried out at 18 regions, and breakpoints at 16 were successfully mapped (Table S3 in Additional data file 1). Observed event lengths closely matched the actual event lengths determined by sequencing across breakpoints, which ranged from 593 to 2,085 bp (Figure 3). Eight of the 16 successfully sequenced regions overlapped at least one record in the DGV (March 2009), and actual event lengths determined by PCR and sequencing exactly matched (to within less than 3 nucleotides) 6 DGV records from sequencing-based studies (Figure S3B in Additional data file 1). Out of 44 randomly selected events for PCR, 4 failed to give specific amplicons, leaving 40, of which 31 were successfully validated, while 6 were ambiguous (77.5% to 92.5% validation rate; Additional data file 3).

These PCR results provided some assurance that the genome scans had relatively low false discovery rates for CNV regions; however, because of the stringent requirements applied to call an event, a noticeable false-negative observation rate was also demonstrated. PCR tests were performed on Yoruba DNAs selected in pairs, whereby an event was observed in one DNA but not the other on the genome-scan arrays. However, the patterns of bands in the PCR gels showed cases of actual losses or gains in 'non-event' DNAs (Figure 2; Additional data file 3). At three regions where truncated PCR amplicons from 'non-event' DNAs were excised and sequenced (including the CNV shown in Figure 2), the deletions mapped to the exact same breakpoints as in the event DNAs (Table S3 in Additional data file 1). For qPCR, out of16 selected gain events tested, 9 were confirmed and 3 were ambiguous, but 4 showed clear evidence of homozygous deletions in the 'non-event' DNA rather than gains in the 'event' DNA (Table S5 in Additional data file 1). Similar to the gel based PCRs, the qPCR results confirmed a fair proportion of putative regions, but also demonstrated that event calls in many individuals were missed.

Because the primary objective of the genome-scans was CNV region discovery, we set stringent requirements for event detection that prioritized low false discovery of regions at the expense of sensitivity to observe sample level calls at those regions. Once CNV regions had been identified in the genome scans, we focused on designing a new array more suited to generating sensitive and reliable sample-level calls, where space on the genome-scan array originally occupied by additional array probes residing outside of CNV regions can now be better used. To optimize array design parameters that would increase sample-level call sensitivity, we designed a small test array with variable probe lengths from 39 to 69 nucleotides, variable probe feature sizes, and 5 replicates of each unique probe, at 150 arbitrarily chosen regions of which 105 were putative CNVs from the genome scan and the remainder were records from the DGV. Filters were not applied to the choice of probe sequences for the test array, which included probes that overlapped any known repetitive regions, including Alu elements. Results from a subset of 12 Yoruba individuals on the small test array suggested the use of 60-nucleotide long probes at 5 micron pitch, with 3 replicates per probe, and the inclusion of probes in repetitive regions, with the exception of Alu elements (data not shown). Probes on the test array corresponding to nearly all Alu elements were not responsive to copy number differences, while probes at other repetitive regions had variable responses that ranged from no change (similar to Alus), reduced response, or full response (similar to non-repetitive regions), with no clear correlation to the class of repeat elements (data not shown). Based on the test array findings, the CNV-typing array was designed to have higher sensitivity for event detection, and includes probes corresponding to repetitive regions (other than Alu elements). Using data from the CNV-typing array, a thorough study of the possible relationships between repeat elements and CNVs is also possible, but is beyond the scope of the current work.

### CNV genotyping

There were approximately 98,000 events observed at the putative CNVs across the 90 Yoruba on the CNV-typing array. Nearly 97% (6,368) of the putative CNV regions discovered in the genome scans were confirmed to have at least one observed event on the CNV-typing array (Table 1). The high confidence putative CNVs had a higher confirmation rate of approximately 99% compared to the singletons (approximately 94%), suggesting a degree of specificity in the region confirmations. Integer copy number event calls, where 0 is homozygous loss, 1 is one copy heterozygous loss, and 3 or more are gain events, were based on CBS at thresholds determined by comparison to reference calls. The reference calls were primarily from the McCarroll *et al*. [14] study, which used the Affymetrix SNP 6.0 genotyping array to determine event calls at approximately 1,300 CNVs in 270 individuals from the HapMap Project [1], including the 90 Yoruba. The validation PCRs (discussed above) were a secondary reference set. Comparisons with the reference calls provided a measure of event sensitivity; and a subset of CNVs that had no events among the Yoruba in the McCarroll *et al*. [14] study, provided an estimate of event specificity (see Materials and methods). Sample-level event calls in the 90 Yoruba individuals at the confirmed CNVs, and at CNVs from the McCarroll *et al*. [14] study, are listed in Additional data files 6 and 7, respectively. Often an individual had two or more event segments within a putative region; this was either because event segments were split by intervening repeat elements, where probes were not responsive to copy number differences, or because the region is complex, having two or more smaller CNVs within a narrow region. Split event segments within a region were treated as one event call if the direction of the multiple segments was consistently all loss or all gain in an individual. On the other hand, complex regions were identified wherever a loss and gain event was observed within a region in the same individual. Complex regions are annotated in Additional data file 2. The positions of the confirmed CNVs listed in Additional data file 2 are based on the first and last positions of event segments detected among individuals.

**Figure 4:**
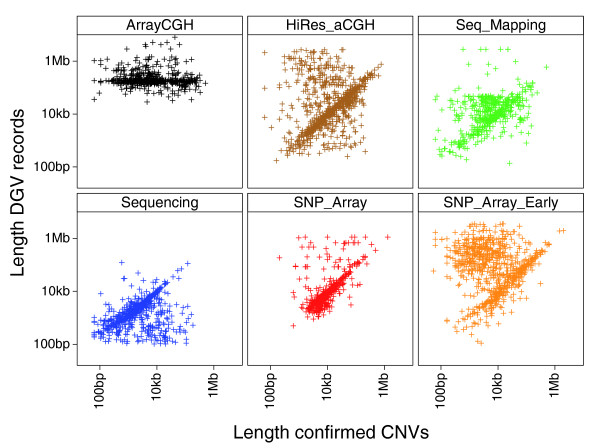
Pair-wise comparison of lengths. The lengths of confirmed CNVs from our work are compared with the closest matching DGV records subdivided by six classifications of CNV discovery methods. The lowest points in the panel sub-plots reflect the limiting resolution of the method classes. Data points above the diagonals represent instances where our higher resolution survey has refined the boundaries of previously reported longer regions, while points below the diagonals are cases where lengths are likely overestimated in our work. Lengths are shown in log scale. Methods from 27 references cited in the DGV (March 2009) were classified (listed in Table S6 in Additional data file 1).

The median length of the confirmed CNVs was 4.4 kb, which was slightly shorter than the median length of the putative CNVs (Table 1). The length distribution of the confirmed CNVs is noticeably more symmetric about the median compared to the lengths of the putative CNVs because many of the overestimated lengths from the smoothed CBS analysis (second peak in the putative distribution) have now been refined downward (Figure 1). The distribution of the CNVs reported in the McCarroll *et al*. [14] study, where the resolution of the SNP6 array is estimated to be approximately 2 kb, starts at approximately 1 kb and is similarly symmetric but is also biased toward longer lengths (Figure 1). The approximately 58% of confirmed CNVs that overlapped DGV had a longer median length of approximately 5.8 kb, while the 2,690 potentially new CNVs not reported in the DGV (6,368 confirmed minus 3,678 that overlap DGV) had a median length of approximately 3.0 kb (Table 1). In cases where a confirmed CNV overlapped with more than one DGV record, it was paired with the closest matching record based on start and end positions in genome build 36. A breakdown of the pair-wise comparisons by the reported discovery methods is shown in Figure 4. The lowest points in the plots reflect the limiting resolution of the various methods; for example, Array CGH is capped below at approximately 30 kb, while whole-genome sequencing (Sequencing in Figure 4) is only limited by the arbitrary minimum cutoff of 100 bp applied to the DGV records. Length correlations were poorest with earlier lower resolution methods, such as BAC arrays (ArrayCGH), and progressively better with regions identified by higher resolution CGH arrays from Agilent (HiRes_aCGH) and earlier SNP genotyping arrays, such as the Affymetrix 500 K and Illumina 550 BeadChip (SNP_Array_Early). The SNP_Array_Early classification also includes shorter CNVs identified by Mendelian inconsistencies and haplotype analysis of SNP data from earlier arrays. Poor correlations in these comparisons with earlier methods are generally instances where our higher resolution arrays have refined the boundaries of previously reported longer regions. The length correlations were higher with pair-end sequence mapping analysis (Seq_Mapping) and recent SNP arrays, namely the Affymetrix SNP 6.0 and Illumina 1 M BeadChip (SNP_Array). The correlation with whole-genome sequencing (Sequencing in Figure 4) was also high, but there was a noticeable subset of regions where the reported DGV lengths are shorter and likely overestimated in our work. The overlapping DGV records were from 27 references [2,6,11-15,18-21,24-39] cited in the DGV (Table S6 in Additional data file 1). CNV discovery methods described in the previous studies were classified as listed in Table S6 in Additional data file 1; the paired DGV records for each of the overlapping confirmed CNVs are listed in Additional data file 2. The pair-wise comparison does not take into account the number of individual samples, or the ethnicity of the individuals. Therefore, in addition to reflecting the differences in resolution among the various discovery methods, the correlation of lengths may be indicative of actual population- or individual-specific differences in overlapping CNV regions.

In order to further compare our results with DGV records at the individual sample level, we selected six recent studies, including the McCarroll *et al*. [14] study, where event calls for one or more Yoruba individuals were reported. The Korbel *et al*. [31] and Kidd *et al*. [30] studies were based on pair-end mapping of sequencing reads from one and four Yoruba individuals, respectively; in the Bentley *et al*. [20] study, one of the Yoruba was whole-genome sequenced; the Perry *et al*. [13] study examined known copy number variants in 10 Yoruba using Agilent microarrays; and in the Wang *et al*. [15] study, 36 Yoruba were genotyped using Illumina BeadChips. For each Yoruba individual in common between our work and a previous study, events were matched based on the longest overlap at genome build 36 positions. Events in complex regions were not included in these comparisons. Event calls reported in the six studies along with the corresponding genome build 36 positions are listed in Additional data file 8. Due to differences in the resolution of the methods, one reported event could match many events observed in our work, and vice-versa. Table 2 lists two sets of comparisons for each study because of these many-to-one and one-to-many matches. The number of observed or reported events in the common Yoruba, and the percentage of these events that were matched and compared, give an indication of the extent of missed events in either our work or the previous studies. Although we report integer copy number calls, some of the studies report events as either loss or gain; in order to simplify the comparisons, we treat integer 0 and 1 copy calls as loss, and 3 or more copy calls as gains. For each Yoruba in common between two sets of calls, we tally pair-wise instances of agreement in the direction of the events, and count disagreements whenever a loss in one set is matched to a gain in the second set, or vice-versa. Sample-level comparisons among pairs of previous studies showed varying degrees of agreement in the direction of calls, and in the numbers of matched regions in common (Table S6 in Additional data file 1). Similarly, the events observed in our work had varying degrees of call agreement and region counts in common with the previous studies (Table 2). For example, the Bentley *et al*. [20] study, which was based on whole-genome sequencing, reported over 4,000 events in the one Yoruba; our work observed approximately 800 events in the same individual, of which only approximately 330 events were in common, with only approximately 93% of these calls in agreement (Table 2). In contrast, the Wang *et al*. [15] study, which was based on Illumina SNP genotyping BeadChips, reported only approximately 1,200 events among 36 Yoruba (approximately 30 per individual) compared to > 40,000 events (approximately 1,000 per individual) in our work; but of the > 800 events that were in common, the direction of > 99% of the calls were in agreement with our work (Table 2).

**Table 2:** Comparison of Yoruba event calls

Study (method)	Common Yoruba	% call agreement	Events compared	Events in study	% study compared	Events in our work	% our work compared
Bentley *et al*. 2008	1	92.6%	338	4,103	8.2%		
(Sequencing)		93.6%	326			792	41.2%
							
Kidd *et al*. 2008	4	92.1%	316	944	33.5%		
(Seq_Mapping)		93.1%	320			4,680	6.8%
							
Korbel *et al*. 2007	1	87.4%	199	732	27.2%		
(Seq_Mapping)		87.0%	200			903	22.1%
							
McCarroll *et al*. 2008	90	99.7%	5,442	7,752	70.2%		
(SNP_Array)		99.6%	5,699			97,745	5.8%
							
Perry *et al*. 2008	10	89.5%	1,403	6,695	21.0%		
(HiRes_aCGH)		89.9%	1,344			10,951	12.3%
							
Wang *et al*. 2007	36	99.3%	814	1,156	70.4%		
(SNP_Array_Early)		99.2%	869			40,739	2.1%

Event calls at confirmed CNVs were compared with events reported in six recent studies that included one or more Yoruba individuals. For each Yoruba in common, events were matched based on the longest overlap. Because of differences in resolution among methods, an event at a confirmed CNV could match many reported events, and vice-versa. For each study, the numbers of compared events differ slightly depending on whether our event calls were compared against study events, or vice versa. Agreement was determined by comparing loss versus gain events, and not integer copy numbers. The percentage of events that overlapped reflects the relative degree of missed events in either our work or the previous study.

Since the 90 Yoruba are each members of 30 family trios, we examined the inheritance of events from parents to children. The majority of copy number polymorphisms are inherited [32], rather than rare *de novo *occurrences [14]. The observations of events in children but not in either of the parents are due to false-positive observation in the child, or false-negative detection in either or both of the parents, with only a very small proportion likely to be true *de novo *events. The approximately 98,000 event calls at 6,368 confirmed CNVs across the 90 Yoruba were grouped by the 30 family trios. Of the total observed events, approximately 10,500 (10.8%) were observed in only the children of trios. The same 30 trios were also part of the McCarroll *et al*. [14] study, in which there were approximately 7,800 reported events (along with approximately 1,600 no_calls) at 859 CNVs in the Yoruba, of which only 25 (0.3%) events were observed in only the children. The 36 Yoruba genotyped in the Wang *et al*. [15] study are members of 12 of the trios, in which approximately 1,110 events were reported, of which 13 (1.2%) were observed only in children. The event calls in the McCarroll *et al*. [14] study benefited from having two fully replicated data sets of 270 individuals run independently in separate laboratories, as well as manual curation of scatter plots that were used to cluster the samples into estimated copy number classes. The sensitivity and specificity of event calls in the Wang *et al*. [15] study benefited from the direct use of the family trio information in the calling algorithm, which markedly reduced the observations of what Wang *et al*. referred to as CNVs inferred in offspring but not detected in parents (CNV-NDPs).

In order to delineate the observations of false positives in children and false negatives in parents in our work, the trio event calls from the McCarroll *et al*. [14] and Wang *et al*. [15] studies were used for a three-way comparison. For each of three comparisons, two of the three data sets were used to create a consensus reference set of event calls from the 12 trios common to the three sets. To reduce the probability of any spurious singleton calls in the reference set, we included only event instances seen at least twice in a given family. The occurrence of false-negative and false-positive event calls in the third data set not in the consensus reference was tallied as shown in Table 3; the individual trio calls in the three comparisons are listed in Additional data file 4. The event calls in our work had a comparable but slightly higher false-positive observation rate (specificity) than the two other studies, but a noticeably higher false-negative detection rate (lower sensitivity) (Table 3). The breakdown of rates in our work, 9.6% false negative versus 1.5% false positive, indicates that the majority of the approximately 10.8% of total events observed only in the children of trios was due to missed events in the parents rather than spurious false observations in the children. Because of the higher resolution of the CNV-typing array, the false-positive rate of our work may be slightly overestimated, particularly in instances where neighboring smaller CNVs from our work were compared with one larger reported CNV from the studies. One such example occurred in one of the trios, trio_id 5, at locus_ids 3804 and 3805, which are separated by approximately 15 kb on chromosome 10. These two CNVs from our work were compared with single overlapping larger DGV records: variation_9648 or variation_37784, from the Wang *et al*. [15] and McCarroll *et al*. [14] studies, respectively (Additional data file 4A). Our work showed loss at locus_id 3804 and gain at locus_id 3805, while both studies called gain in the corresponding larger region. The loss calls at the smaller locus_id 3804 are tallied as disagreements in Table 3; however, our higher resolution array indicates that the loss event was passed from father to child in this trio (Additional data file 4A), which raises the possibility that these events may have been missed in the two studies.

**Table 3:** Three-way comparison of event calls in trios

	**Confirmed loci**		**McCarroll *et al*. (2008)**		**Wang *et al*. (2007)**	
			
Reference events	428		338		348	
						
Calls compared	387	90.4%	328	97.0%	329	94.5%
False negatives	41	9.6%	10	3.0%	19	5.5%
Missed loss	18		7		15	
Missed gain	23		3		4	
						
Agree with reference	384	99.2%	328	100.0%	329	100.0%
Disagree	3	0.8%	0	0.0%	0	0.0%
						
Called events	393		331		333	
False positves	6	1.5%	3	0.9%	4	1.2%

Twelve Yoruba family trios are common to the Wang *et al*. [15] and McCarroll *et al*. [14] studies, and our work. For each comparison, two of the three data sets were used to create a consensus reference. Consensus among the references and agreement with the references were determined by comparing loss versus gain events, and not integer copy numbers. The sample-level calls in each of the three comparisons are listed in Additional data file 4.

### Events at CNVs discovered by whole-genome sequencing

The CNV-typing array has probes corresponding to shorter (< 1 kb) CNVs discovered by sequencing individual genomes [18,19], enabling estimates of event frequencies at these CNVs in our Yoruba samples. DGV records with lengths < 1 kb are classified as indels, but for our array design we included records down to an arbitrary cutoff of 100 bp, and consider these longer indels as shorter CNVs. Probes on the CNV-typing array corresponding to regions from the Levy *et al*. [18] and Wheeler *et al*. [19] studies were grouped as Levy+Wheeler, corresponding to regions in common between the two studies, or Levy_only or Wheeler_only, corresponding to regions reported in only one of the studies (Table 4). Sample-level calls at the three groups of regions from the Levy *et al*. [18] and Wheeler *et al*. [19] studies are listed in Additional data file 7. Regions from the two studies that overlapped any of the putative CNVs from our genome-scan were excluded. The overlap between putative CNVs, and regions from the Levy *et al*. [18] and Wheeler *et al*. [19] studies was only 9% and 22%, respectively. In contrast, there was 91% overlap with 859 CNVs (median length of 7.4 kb), with at least one reported event in a Yoruba from the McCarroll *et al*. [14] study.

**Table 4:** Summary of events at CNV regions discovered by sequencing

	Confirmed CNVs	Confirmed < 1 kb	Levy + Wheeler	Levy_only	Wheeler_only
Reported CNVs			221	1,753	957
with YRI events	6,368	1,107	172	1,651	740
% with events			77.8%	94.2%	77.3%
					
Median length	4,380 bp	490 bp	193 bp	190 bp	240 bp
25th percentile	1,519 bp	290 bp	110 bp	120 bp	120 bp
75th percentile	13,230 bp	735 bp	849 bp	380 bp	974 bp
					
Events	97,953	27,718	4056	40,968	13,968
Events per region	15.4	25.0	23.6	24.8	18.9
					
Homozygous loss (0)	5,792	2,177	321	1,004	1,092
One copy loss (1)	55,593	14,335	1,792	20,353	6,882
One copy gain (3)	31,198	9,082	1,415	16,926	4,879
Multiple gains (4+)	5,370	2,124	528	2,685	1,115
					
Loss:gain	1.7	1.5	1.1	1.1	1.3

Regions are from the Levy *et al*. [18] and Wheeler *et al*. [19] whole-genome sequencing studies. Levy+Wheeler refers to regions common to both studies, while Levy_only and Wheeler_only are regions reported only in either study. Regions that overlap any of the putative CNVs from the genome-scan were not included in these three sets. Reported refers to the numbers of CNVs discovered in the studies. 'With events' is the tally of reported regions having at least one observed event on the CNV-typing array, and 'events' is the tally from all 90 Yoruba at all regions. The events are broken down by tallies of integer copy number calls, where 0 is homozygous loss, 1 and 3 are heterozygous loss and gain, and 4+ is the tally of multiple gains. 'Loss:gain' is the ratio of loss and gain event tallies.

**Figure 5:**
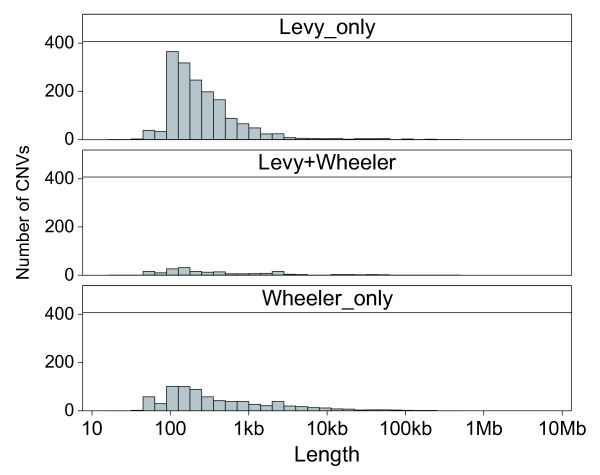
Length distributions of CNV regions discovered by sequencing. Lengths of regions as summarized in Table 1 with an event in at least one Yoruba. Lengths are shown in log scale.

**Figure 6:**
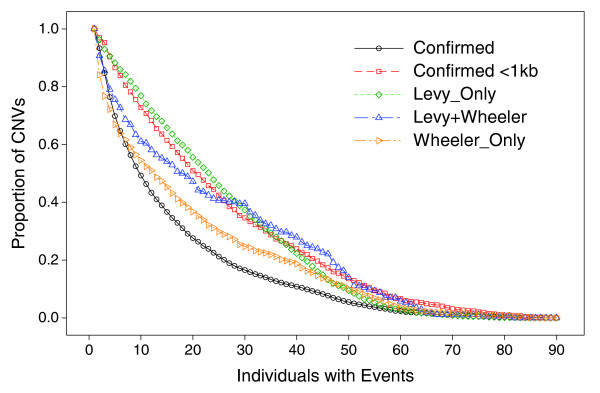
Cumulative event occurrence across the 90 Yoruba. The region groups are summarized in Table 4. The numbers of regions in each group are scaled to 1.

**Figure 7:**
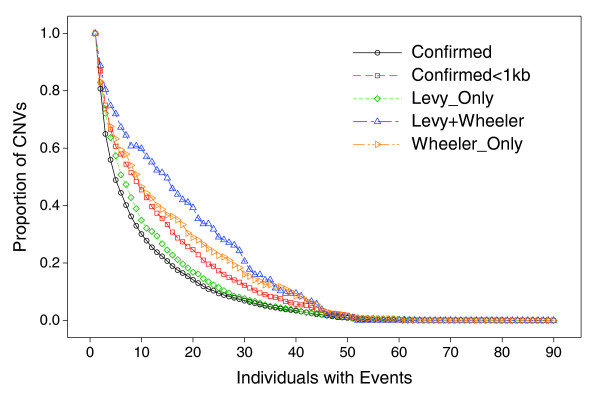
Similar cumulative event occurrence when applying a more stringent event threshold of 0.35 compared to 0.175 in Figure 6. Under the stringent threshold where fewer events were observed, approximately two-thirds of the regions summarized in Table 4 showed at least one event: 4,733 of confirmed CNVs, and 107, 1,153, and 411, of Levy+Wheeler, Levy_only, and Wheeler_only regions, respectively.

**Figure 8:**
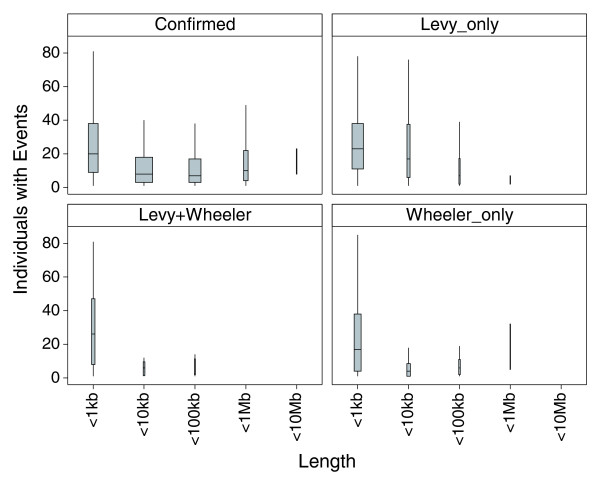
Breakdown of event occurrence tallies by region lengths. Panels correspond to confirmed CNVs from our work, and regions discovered by whole-genome sequencing as summarized in Table 4. Box-plots show medians and interquartile ranges, with whiskers extending to maximum or minimum values within 1.5 times the 75th or 25th percentiles, respectively. The width of boxes is proportional to the number of regions.

A large majority (> 77%) of the shorter CNVs that were discovered by sequencing individuals of Western European descent had at least one observed event in the Yoruba (Table 4). Based on detected events across the 90 Yoruba, the median lengths were 190 bp and 240 bp in the Levy_only and Wheeler_only groups, respectively (Table 4), and the length distributions of these regions were skewed toward the 100-bp cutoff (Figure 5). Bearing in mind that observed frequencies may be underestimated due to missed event calls as suggested by the trio analysis above, the three groups of regions had noticeably higher event frequencies compared to the 6,368 confirmed CNVs from our work, as measured by average events per region, or cumulative events in the 90 Yoruba (Table 4, Figure 6). But a subset of 1,107 confirmed CNVs from our work, having lengths < 1 kb, had similar high event frequencies, and cumulative events, resembling the Levy_only group (Figure 6). The cumulative event curves are distinctly different between the Levy_only and Wheeler_only groups, with the Levy+Wheeler curve intermediate between the two. Increasing the specificity of event calls (lowering false-positive events at the expense of sensitivity) noticeably lowered event frequencies in the Levy_only group, and to a lesser degree in the < 1 kb confirmed CNVs from our work, but the Levy+Wheeler and Wheeler_only groups maintained high relative event frequencies (Figure 7). The occurrence of loss events was higher than gain events at the confirmed CNVs, but to a lesser degree in the Wheeler_only group, and even less so in the Levy_only and Levy+Wheeler groups (Table 4). For comparison, in previous studies the ratio of loss:gain in Yoruba ranged from 6.3, 3.5, 2.5, to 0.9, and 0.9 in the McCarroll *et al*. [14], Korbel *et al*. [31], Wang *et al*. [15], Perry *et al*. [13], and Kidd *et al*. [30] studies, respectively. In total, we generated sample-level event calls in the 90 Yoruba at nearly 9,000 regions (approximately 4% of genome), including > 3,300 shorter regions (< 1 kb). A breakdown of event occurrence by region lengths shows that event frequencies were higher in subsets of shorter (< 1 kb) CNVs from both our work or the Levy *et al*. [18] and Wheeler *et al*. [19] studies (Figure 8).

## Discussion

That our high resolution genome scans of the 90 Yoruba uncovered as many as 2,690 potentially new CNVs with a median length of approximately 3.0 kb suggests that there are many more CNVs yet to be discovered on the shorter end of the size range. Because of the high resolution of our genome-scan arrays, we were able to delineate neighboring multiple smaller CNVs at regions earlier reported as single larger CNVs, as illustrated in Figure S2 in Additional data file 1. Perry *et al*. [13] observed and validated other such instances of multiple CNVs in close proximity, and describe these cases as architecturally complex CNV regions. The tight correlation between observed event lengths and actual lengths determined by PCR and breakpoint sequencing (Figure 2b) reflects fairly accurate breakpoint mapping of events in the approximately 1 to 2 kb range, and suggests, by extrapolation, accuracy in longer ranges. Of the 16 CNVs confirmed by PCR and breakpoint sequencing, six were exact matches to DGV records reported by sequencing-based methods (Table S3 in Additional data file 1). Specifically, three of the six matched records from the Mills *et al*. [2] study, which mapped publicly available sequencing trace data, two matched records from the Wheeler *et al*. [19] study, which whole-genome sequenced an individual of Western European descent, and one matched a record from the Bentley *et al*. [20] study, which sequenced a different Yoruba. These are instances of the same exact events occurring in different individuals of varying ethnicities, and likely represent older CNVs that have taken root in the genome. The whole-genome sequencing data generated by sequencing more individuals, such as in the 1000 Genomes Project, will undoubtedly produce a more thorough catalog of shorter CNVs in the genome, including an assessment of the age of these variations.

Even at a resolution of approximately 200 bp, our genome scan detected only a fraction of the CNVs reported in whole-genome sequencing studies (Levy *et al*. [18] and Wheeler *et al*. [19] studies at 9% and 22%, respectively). Our inability to detect shorter (< 1 kb) CNVs shows one limitation of using microarrays, although continued advances in array manufacturing technology could further increase array probe density in the future. In the meantime, a viable approach is to rely on DNA sequencing for CNV region discovery in limited numbers of samples, and follow up with microarrays for localized sample-level event detection across larger sample sets as we have done here. Shorter (< 1 kb) regions that were identified in our genome-scan, such as the example shown in Figure 2, were often instances of homozygous deletions, which manifest stronger event segments. In contrast, one-copy-loss events give weaker segments that were often missed, but are likely to occur more frequently than homozygous deletions. These instances of false-negative CNV discovery, particularly in shorter regions with rare event frequencies, could be mitigated by using an improved genome-scan array design with longer probes and the inclusion of multiple replicates of each probe, just as we have demonstrated for the CNV-typing array. In contrast, the higher overlap (91%) between putative CNVs and the generally longer CNVs (median length approximately 7.4 kb) from the McCarroll *et al*. [14] study suggests that the genome scan captured a fair proportion of CNVs > 1 kb. We were able to observe events at approximately 97% of the putative CNVs from the genome scans on the CNV-typing array. The low false-positive rate of putative CNVs on the typing array, and the fairly successful PCR validation, are consistent with the stringent requirement of having had to observe events in at least two of three genome-scan array designs, which served as technical replicates. To reduce noise from probe to probe intensity variations, the CNV-typing array has each unique probe placed in triplicate at scattered locations on the array, and the signals from the triplicate probes were summarized by median polish. The example segmentation results shown in Figure 2 illustrate the reduction in noise on the typing array. In addition to the triplicate probes, the CNV-typing array has improved sensitivity for event detection by the use of 60-nucleotide long probes compared to non-replicated 49-mer probes on the genome scan arrays.

The disparity in agreement of sample-level event calls and matched regions between our work and previous studies (Table 2) may be due to sampling differences, which ranged from only one Yoruba individual in common up to 90 individuals; but more likely reflects underlying differences in specificity and sensitivity, as well as genome coverage biases inherent in the various methods, as well as in our work. These differences are also apparent in pair-wise comparisons among the six previous studies (Table S6 in Additional data file 1), and point to the difficulty in determining absolute accuracy and event call completeness. An examination of the inheritance of events from parents to children among the Yoruba trios in our work, along with events reported among trios in the McCarroll *et al*. [14] and Wang *et al*. [15] studies, provided an assessment of false-positive and false-negative rates of event detection. Although slightly higher, the specificity of event detection on the CNV-typing array was comparable to the previous studies, and may be underestimated because of the higher resolution; on the other hand, the sensitivity to detect events was noticeably lower (Table 3). The majority of events observed only in the children of trios were due to missed events in the parents. The sensitivity could improve with the availability of additional and replicate data sets and manual curation of intermediate results, or the use of family trio information, as was likely the case in the McCarroll *et al*. [14] and Wang *et al*. [15] studies, respectively. That these two previous studies also showed varying degrees of false negatives and false positives, and the low proportion of CNVs in common between the Levy *et al*. [18] and Wheeler *et al*. [19] sequencing studies (Table 4), reinforces the benefit of building a consensus from multiple studies. As more sample-level data become available, particularly from whole-genome sequencing and higher resolution microarray-based studies, many of the discrepancies in the inter-reference comparisons (Table 2; Table S6 in Additional data file 1) should be resolved through higher confidence consensus among methods and studies.

Events observed in the 90 Yoruba showed higher frequencies at shorter CNVs compared to longer CNVs (> 1 kb; Figure 8). The higher frequencies are consistent with expectations that events in shorter regions are under less selective pressure than at longer regions, which are more likely to be deleterious [40]. The differences in the cumulative event frequencies, even under stringent specificity thresholds (Figures 6 and 7), are likely a reflection of differences in the di-deoxy and 454 polony sequencing methods used in the Levy *et al*. [18] and Wheeler *et al*. [19] studies, respectively, and suggest that the CNV-typing array is sensitive to detect some subtle characteristic differences inherent in the regions discovered in the two separate studies.

## Conclusions

Recent studies using high resolution microarrays and whole-genome sequencing have made major inroads toward a complete catalog of CNVs in the human genome. Our work demonstrated the use of even higher resolution microarrays to uncover approximately 2,700 potentially new CNVs, and to observe events in 90 Yoruba at regions discovered by whole-genome sequencing of single individuals. The approximately 3,300 shorter regions (< 1 kb) examined in our current work are likely just a fraction of what will eventually be discovered through sequencing more individuals. In the near term, high resolution microarrays offer a cost-effective means to confirm these shorter CNVs, and type large numbers of individuals in order to gain biological insights beyond the initial discovery.

## Materials and methods

### Array synthesis

Arrays were synthesized following standard Affymetrix GeneChip manufacturing methods utilizing contact lithography and phosphoramidite nucleoside monomers bearing photolabile 5'-protecting groups. Fused-silica wafer substrates were prepared by standard methods with trialkoxy aminosilane as previously described [16]. An improved 5'-protecting group provided for more efficient photolysis and chain extension, and therefore fewer truncated probe sequences [17]. The genome-scan arrays and CNV-typing array required 141 and 179 synthesis steps, respectively, resulting in 3'-immobilized DNA probes of 49 and 60 nucleotides in length. After the final lithographic exposure step, the wafer was de-protected in an ethanolic amine solution for a total of 8 hours prior to dicing and packaging.

### Array designs

Candidate 49-mer probe sequences for the three genome-scan array designs were chosen from the non-repetitive regions of the genome, and filtered for extraneous matches to the genome in the central 16 nucleotides, resulting in a total of 32 million unique probes. Rather than placing probes sequentially across the three arrays, probes were dispersed such that every second and third probe against the genome was placed on separate arrays (Figure S1A in Additional data file 1). Because of the inter-digitating of probes across the three designs, the inter-probe interval in any one design between the center positions of neighboring probes is generally 147 bp (the combined length of three probes). However, because probes were filtered out at repetitive regions throughout the genome the overall median interval between neighboring probes on the genome-scan arrays is 196 bp.

The CNV-typing array design consists of approximately 800,000 unique probes, with each in triplicate for a total of approximately 2.4 million 60-mer probes. The replicate probes are placed in separated locations on the array to mitigate any regional variations in signals. The approximately 800,000 unique probes are organized into approximately 16,000 partitions, each containing up to 50 unique probes. The probe partitions correspond to putative or reported CNV boundaries. The probes within a partition are evenly spaced along chromosomes, with the exception of regions corresponding to Alu elements and occurrences of high allele frequency SNPs. In order to mitigate any potential compounding effects on signals, probes with a common SNP (minor allele frequency > 0.05) in the HapMap repository [41] within the central 30 nucleotides were not allowed. In contrast to the genome-scan arrays where probes in repetitive elements were mostly filtered out, the CNV-typing design has probes in all repeat regions other than Alu elements. The closest spacing between the central positions in the 60-mer probes is 10 bp apart. For CNVs shorter than 500 bp, the partition will contain less than 50 unique probes; for example, a 300 bp region will have 30 overlapping probes with center positions spaced 10 bp apart. For CNVs longer than 500 bp, the 50 probes will be spaced further apart; for example, a 3,000 bp CNV will have 50 probes lined end-to-end, with no overlap between 60-mer probes. Partitions corresponding to shorter CNVs discovered by whole-genome sequencing of individual genomes [18,19] were prioritized and assigned first, followed by putative CNVs from the genome scan, and then supplemented with regions that overlapped in at least two records in the DGV (November 2008) (Figure S1B in Additional data file 1). Because shorter CNVs were assigned first, the shorter CNVs tend to have the highest probe density. A longer CNV that overlaps a shorter CNV will be represented by two partitions with different probe densities. In this way, a partition can map to one or more CNV regions; conversely, a CNV can be represented by one or more probe partition (Figure S1B in Additional data file 1). The probe sequences and build 36 chromosome positions of all the four array designs are available at ArrayExpress [42] under accession number E-TABM-838.

### Yoruba samples

The 90 Yoruba individuals are from the HapMap Project [1]; genomic DNA samples were obtained as immortalized cell line isolates from the Coriell Institute [43]. During initial analysis of the genome scans, unusually high occurrences of gain events in chromosome 12 from NA19193, and chromosome 9 from NA19208 were observed (Figure S3 in Additional data file 1). These observations are consistent with lymphoblastoid cell line artifacts that have been previously reported in these two samples [12,44]. Data from these two chromosomes were excluded from all subsequent segmentation analyses.

### Sample preparation

Whole-genome amplification of genomic DNA samples was performed using the REPLI-g Midi kit (Qiagen, Valencia, CA, USA) following manufacturer-supplied instructions, starting with 200 ng of input DNA in a 60 μl reaction. Amplified DNA was randomly fragmented by controlled partial digestion with DNase I. The optimal DNA target length for hybridization to the arrays was found to be in the range of 50 to 300 bp, with the majority of fragments at 100 to 200 bp. DNaseI at 2.5 U/μl (Affymetrix, Santa Clara, CA, USA) was freshly diluted in 10 mM Tris pH 8 to a concentration of 0.3 U/μl; 3 μl of the diluted DNaseI was added to 60 μl of amplified DNA and 7 μl Fragmentation buffer (Affymetrix) at 37°C. To achieve the optimal size range, test fragmentation time courses were first performed using a small amount of the amplified DNA samples, where the incubation varied from 4 to 26 minutes. Following fragmentation, the amplified DNA was ethanol precipitated and resuspended in 33.5 μl water; 1 μl was removed to measure concentration, which was typically approximately 1.5 μg/μl. The fragmented DNA was then end-labeled with biotin using 2.5 μl of 30 mM DNA labeling reagent (Affymetrix) and 5 μl of Terminal Transferase (Affymetrix) in a 50 μl reaction, which included 10 μl of 5× TdT buffer (Affymetrix). Labeling reactions were incubated for 2 hours at 37°C until heat inactivation at 95°C for 10 minutes.

### Hybridization to arrays

The labeled DNAs were hybridized to each array in 200-μl volumes. In addition to 15 μl of approximately 1 μg/μl labeled DNAs, the hybridization solution contained 100 μg denatured Herring sperm DNA (Promega, Madison, WI, USA), 100 μg Yeast RNA (Ambion, Austin, TX, USA), 20 μg freshly denatured COT-1 DNA (Invitrogen, Carlsbad, CA, USA), 12% formamide, 0.25 pM gridding oligo (Affymetrix), and 140 μl hybridization buffer, which consists of 4.8 M TMACl, 15 mM Tris pH 8, and 0.015% Triton X-100. Hybridizations were carried out in Affymetrix ovens for 40 hours at 50°C with rotation set at 30 rpm. Following hybridization, arrays were rinsed twice, and then incubated with 0.2× SSPE containing 0.005% Trition X-100 for 30 minutes at 42°C with rotation set at 15 rpm. The arrays were rinsed and filled with Wash buffer A (Affymetrix). Staining with streptavidin, R-phycoerythrin conjugate (Invitrogen) and scanning with the GCS3000 instrument (Affymetrix) were performed as described in the Affymetrix GeneChip SNP 6.0 manual [45].

### PCR and sequencing

A sampling of putative CNVs in pairs of Yoruba samples was selected where an event was observed in one DNA but not the other (Additional data file 3 and Table S5 in Additional data file 1). For standard PCR, putative CNVs having an event segment within a sample in the range 400 bp to 2.5 kb were tested; for quantitative PCR, CNVs having gain segments between 500 bp and 10 kb were tested. Primer sequences for standard PCR were designed from 300-bp candidate regions upstream or downstream of the longest event segments within a sample, and for qPCR, from within the shortest gain segment. Candidate regions having less than 50% RepeatMask (UCSC) were processed in either Primer3 [46] or PrimerExpress 3.0 (Applied Biosystems, Foster City, CA, USA) for standard or qPCR primer design, respectively. Primer sequences are listed in Additional data file 5. Standard PCRs using Advantage LA polymerase (Clontech, Mountain View, CA, USA) and 400 nM primers (synthesized by IDT Integrated DNA Technologies, Coraville, IA, USA) started with 100 ng sample DNA. Following denaturation at 94°C for 1 minute, reactions were cycled 30 times as follows: 94°C for 30 seconds, 58°C for 30 seconds, and 72°C for 3 minutes, with a 72°C final hold for 7 minutes. Amplicons corresponding to loss events were excised from agarose gels, and sequenced using either of the PCR primers. CNV loss breakpoints were determined by mapping the amplicon sequences to genome build 36 with BLAT [47] (UCSC). An Applied Biosystems 7300 machine was used for quantitative PCRs, according to the manufacturer's instructions. Typically, the reactions included SYBR Advantage 2× qPCR mix (Clontech), 200 nM primers, 500 nM ROX reference (Roche, Indianapolis, IN, USA), and 90 ng sample DNA. Following denaturation at 95°C for 30 seconds, reactions were cycled 40 times as follows: 95°C for 5 seconds and 60°C for 34 seconds.

### Data processing

Signal intensities were quantile normalized [23] in sets of > 90 samples for each of the 3 genome-scan chip designs, or in two separate sets of 45 samples for the CNV-typing design. The triplicate probes on the CNV-typing array were summarized by median polish. For probes on autosomal chromosomes, median signals were calculated using all samples, while for probes on chromosome X and chromosome Y only female or male samples were used, respectively. Two to five additional non-Yoruba samples were part of the normalization and calculation of medians in the genome-scan, but were not included in subsequent analyses. The medians were the basis of log2 ratios for segmentation analysis. In the initial analysis of the genome-scans, a small subset of samples had disproportionately high occurrences of apparent gain or loss events. These artifact events were no longer observed after filtering out probes with GC content > 0.6, and by applying a sample-specific correction to the log2 ratios. The corrected log2 ratios were derived in the following manner: for each probe, calculate the GC content of its surrounding 50 kb region; sort the GC content values into 50 equal size bins; within each sample, for each bin, calculate the median of the log2 ratios for all probes with GC content in that bin; correct the log2 ratios in that sample by subtracting off the medians derived in the prior step. Figure S4A in Additional data file 1 shows an example of artificially high log2 ratio values corresponding to probes with high GC content in one of the samples with artifact gain events. The benefit of the filtering and correction to the segmentation analysis is illustrated in Figure S4B in Additional data file 1. Similar benefits of probe filtering and correction have been reported in copy number analysis using other arrays [48,49].

### Segmentation: genome scan

CBS [22] was implemented in the R package DNAcopy [50]. For each sample and each genome-scan design, sets of 750 probes (approximately 150 to 200 kb windows) were analyzed using signal from all probes (without smoothing) to specifically look for CNVs shorter than 100 kb. To identify longer CNVs, segmentation analysis was performed with signal smoothing using Nexus Rank Segmentation (BioDiscovery, El Segundo, CA, USA), a proprietary algorithm based on CBS. The smoothed segmentation was run on entire chromosomes. Signals were smoothed by averaging eight consecutive probes. The level of smoothing was chosen based on chromosome X receiver operating characteristic (ROC) analyses that compared smoothing with 2 probes up to 256 probes (Figure S5B in Additional data file 1). Initially, consecutive inter-digitated probes from the three genome-scan arrays were combined to get the highest possible resolution, up to 49 bp in non-repetitive regions. However, the ROC analysis in Figure S5B in Additional data file 1 shows lower sensitivity and specificity when combining the three designs, compared to using probes from only one design (Figure S5A in Additional data file 1). Averaging the combined probes from the three designs (smooth 3 in Figure S5B in Additional data file 1) appears to have comparable performance to the unsmoothed curve using only one of the array designs (all probes in Figure S5A in Additional data file 1). However, actual segmentation analysis from averaging three probes combined from the three designs resulted in a highly disparate range of event tallies in individual samples, indicative of false positives. Although using probes from only one design at a time entailed a lower resolution (at best 147 bp) in the genome scan, segmentation was computed separately for each design. By using the three genome-scan designs as technical replicates instead of in combination, lower rates of false discovery (higher specificity) was prioritized over higher resolution and sensitivity to detect shorter CNVs.

For both non-smoothed and smoothed segmentation analyses, gain and loss event thresholds were set to segment mean log2 ratios of > 0.25 and <-0.25, respectively. For each sample, overlapping segments from at least two of three chip designs was required to meet the thresholds in order to call a gain or loss. The boundaries of an individual event were defined by the longest overlap between any two event segments meeting the threshold. A putative CNV was defined as regions having events observed in at least one individual; and the boundaries of a CNV were defined by the longest event among individuals. There were 401 regions where putative CNVs from the non-smoothed segmentation intersected putative regions from the smoothed segmentation. In regions where multiple putative CNVs from the non-smoothed segmentation corresponded to one putative region from the smoothed segmentation, the non-smoothed CNVs were chosen. In regions of one-to-one correspondence, the generally longer putative CNVs from the smoothed segmentation were chosen.

### Segmentation: CNV typing

The sample-level event calling thresholds used in the segmentation analysis of the CNV-typing array data were determined by comparing against reference event calls taken primarily from the McCarroll *et al*. [14] study and, to a lesser extent, from the PCR validation. The McCarroll *et al*. [14] study reported integer copy number calls at 1,301 CNVs in 270 individuals from the HapMap Project, including the 90 Yoruba. Of these 1,301 CNVs, 1,153 regions were represented on the CNV-typing array by at least one probe partition corresponding to regions overlapping at least two records in the DGV (November 2008). These 1,153 CNVs were grouped into a subset of 859 CNVs with at least one reported event in a Yoruba, and a second subset of 294 regions that did not have any Yoruba events reported in the McCarroll *et al*. [14] study. These non-Yoruba event regions were further checked against five other papers cited in the DGV [13,15,20,30,31], where events were reported in at least one Yoruba. Of the subset of 294 CNVs without Yoruba events in the McCarroll *et al*. [14] study, 234 regions had no reported Yoruba events in any of the five other papers. After excluding no-calls from the McCarroll *et al*. [14] study, there were a total of 20,847 diploid calls at the 234 regions in 90 Yoruba (listed as REF-NonPoly6papers in Additional data file 8). These diploid calls were used as reference to assess call specificity, as reflected in false-positive event observations. Initial comparisons of CNV-typing data with the 859 CNVs having reported Yoruba events in the McCarroll *et al*. [14] study, showed that a subset of 127 regions had reported calls that agreed only when offset by one integer. Comparisons with calls reported in the five other papers with Yoruba events showed lower agreement in this subset of 127. A cursory examination of HapMap genotypes suggested higher congruence with offset calls at many of the 127 regions, where, for example, one-copy-loss events should correspond to consecutive SNP loci with homozygous genotypes, but instead diploid copy number calls were reported. After omitting these 127 regions, the remaining 732 CNVs from the McCarroll *et al*. [14] study had 7,752 reported events that were used as reference to assess event sensitivity (listed as REF-McCarroll-Sel in Additional data file 8). Events from PCRs shown in Tables S3 and S5 in Additional data file 1, and in Additional data file 3 were also used as reference (listed as pcr-GS in Additional data file 8).

To assess specificity and sensitivity of event detection in the CNV-typing data, segmentation thresholds were titrated at the 732 McCarroll reference CNVs, and at 6,578 putative CNVs from the genome scan. Any false positives or false negatives in the McCarroll reference event calls will artificially lower the estimates of sensitivity or specificity, respectively, of the CNV-typing array. Figure S6 in Additional data file 1 summarizes the results at seven threshold values that ranged from 0.35 (-0.35) to 0.10 (-10), and shows the trade-off between higher specificity and lower sensitivity. Event thresholds of -0.175 and 0.175 for loss and gain calls, respectively, were chosen; based on further titrations, second-level thresholds of -0.70 and 0.45 were chosen for homozygous deletions and multi-copy gain events, respectively. For each individual Yoruba sample, sets of probes for each CNV were analyzed separately by CBS, and segments with log2 ratios above or below the thresholds were called as events. Probes in the CNV-typing design were grouped into partitions corresponding to known or putative CNVs, where a given CNV may be represented by more than one partition (Figure S1B in Additional data file 1). Although the CNVs vary in the number and density (probes per base-pair) of corresponding probes, the degree of discrimination of log2 ratios above or below the event thresholds were comparable across a range of event lengths and numbers of probes, with only slight loss of discrimination at longer lengths and fewer probes (Figure S7 in Additional data file 1). Microarray raw intensities and chip library files are available at ArrayExpress [42] under accession number E-TABM-838. Reported CNVs are displayed at the DGV [51].

## Abbreviations

BAC: bacterial artificial chromosome; CBS: circular binary segmentation; CGH: comparative genome hybridization; CNV: copy number variant; DGV: Database of Genomic Variants; qPCR: quantitative PCR; ROC: receiver operating characteristic; SNP: single-nucleotide polymorphism.

## Competing interests

All authors are current or former employees of Affymetrix.

## Authors' contributions

RR and GF conceived the experiments and designed the genome-scan arrays. HM and GF designed the typing array. PW and GF prepared samples and hybridized the arrays. PW, GF, and HM ran PCRs. HM and JH performed data analysis. HM and GF wrote the manuscript.

## Additional data files

The following additional data are available with the online version of this paper: Figures S1 to S7 and Tables S3, S5, S6 and S7 (Additional data file 1); a table listing confirmed and putative CNVs (Additional data file 2); a table listing PCR validation results at 44 regions along with gel images, which correspond to 4% agarose (E-gel), gradient polyacrlyamide (PA gel), and 1% agarose (1% gel) electrophoresis gels (Additional data file 3); list of event calls and consensus reference in trios (Additional data file 4); list of primer sequences, along with sizes of the expected amplicons (Additional data file 5); integer copy number events observed on the CNV-typing array in 90 Yoruba at 6,368 confirmed CNVs (Additional data file 6); observed events on the CNV-typing array in the 90 Yoruba at 1,153 CNVs reported in the McCarroll *et al*. [14] study (listed as chp-McCarroll2008) and at regions from the Levy *et al*. [18] and Wheeler *et al*. [19] studies as summarized in Table 4 (listed as chp-LevyWheel, chp-LevyOnly, and chp-WheelerOnly) (Additional data file 7); reported events from six papers that included at least one Yoruba (Additional data file 8).

## Supplementary Material

Additional data file 1 Figure S1 is a description of the chip designs. Figure S1A: the sequential 49-mer probes against the genome were dispersed across the three chip designs. Probes corresponding to extraneous matches to the genome in the central 16 nucleotides were omitted from the designs. Figure S1B: probes on the CNV-typing design were organized into probe partitions corresponding to putative CNVs from the genome scan (in red), reported CNVs from whole-genome sequencing studies (Levy *et al*. [18] and Wheeler *et al*. [19]; in blue), and CNV regions in the DGV (November 2008) overlapping in at least two database records (in green). The five example partitions correspond to regions of varying length, and are represented by up to 50 probes each; regions less than 500 bp have fewer probes because the probe spacing is capped at 10 bp per probe. A partition can map to more than one CNV; conversely, a CNV can be represented by one or more partitions. Figure S2 shows regions with reported CNVs in proximity. Two example regions of width approximately 200 kb (Figure S2A) and approximately 20 kb (Figure S2B) are displayed in Nexus chromosome views (BioDiscovery), along with DGV browser views [51]. The Nexus views show the percentage of Yoruba samples with observed gains and losses in green and red, respectively. DGV records that were paired with putative CNVs are colored with blue stripes. In the first example (Figure S2A), the DGV records with red stripes more closely match the smaller CNVs. Figure S3 shows cell line artifacts. The initial smoothed segmentation analysis, displayed in Nexus (BioDiscovery) drill-down views, showed disproportionately high gain events across chromosome 12 in Yoruba sample NA19193 (Figure S3A) and chromosome 9 in sample NA19208 (Figure S3B). The green and red bars along the chromosome pictograms mark regions with gains and losses, respectively. These observations are consistent with previously reported lymphoblastoid cell-line artifacts, namely mosaic duplications, in these samples [12,44]. Figure S4 shows probe GC filtering and correction. Figure S4A: a handful of samples, including NA18870, showed disproportionately high numbers of events in the initial segmentation analysis when using all probes and without GC correction. The plots show log ratios across the range of probe GC content for a random sampling of 20,000 probes in chromosome 20 from the b-chip experiment run on sample NA18870. Before probe filtering and correction, there is a noticeable 'fishtail' of high log ratios corresponding to higher GC content, which manifests in artificially high numbers of gain events. Similarly, in samples with high numbers of loss events, tails of low log ratios were observed. Figure S4B: segmentation analysis results for chromosome 20 from the b-chip experiment run on sample NA18870. The average log ratios of the delineated segments are plotted against their lengths (in log scale). Before the filtering and correction, there is a noticeable bolus of segments with lengths between approximately 500 kb and approximately 5 kb that have average log ratios well above the threshold value of 0.25 for gains. There are also longer segments up to approximately 500 kb that have ratios above the 0.25 threshold. After filtering and correction, however, the number of segments above or below the 0.25 and -0.25 thresholds is much fewer, and the vast majority of segments have average ratios hovering close to 0, indicative of non-events, and lengths close to approximately 200 kb, which is in line with the windows of 750 probes in the segmentation analysis. Figure S5 is a receiver operator characteristic (ROC) analysis of chromosome X. The ROC curves show the tradeoff between false positives and sensitivity [52], based on comparing chromosome X probes in female and male samples. Log2 ratios were calculated for all 90 samples at approximately 470,000 chromosome X probes in each chip design, using median signals based only on female samples. Ratios close to 0 are indicative of two copies of chromosome X (non-events), while lower ratios, particularly in male samples, are indicative of one copy (surrogate 'loss' events). Thresholds for the ratios were varied from -3.0 to 3.0 in 0.01 increments, and at each increment the cumulative fraction of probes below the thresholds were determined separately for female and male samples. The ROC curves show sensitivity as the fraction of probes in males below the threshold, and the false positives as the log of the fraction of probes in females below the threshold. Consecutive probes were averaged to generate the family of smoothed curves. The ROC curve for the b-chip design is shown in panel A, while panel B shows the result of combining consecutive inter-digitated probes from the three chip designs. At the segmentation threshold of -0.25 for loss events, the b-chip had sensitivity of 0.83 and false positives of 0.08 without smoothing (all in panel A), and sensitivity of 0.91 and false positives of 0.008 when smoothing with eight probes (smooth 8 in panel A). The sensitivity was 0.88 and false positives 0.05 when smoothing over three probes combined from the three designs (smooth 3 in panel B). Compared with just the b-chip alone, these ROC measures for the combined probes suggested higher performance at the same effective resolution. However, the segmentation with the combined probes resulted in greater variation in the tallies of events in individual samples, compared to probes from each design separately. Because the ROC measures are based on aggregating the entire sample set, subtle variations that manifest at the individual sample level may not be apparent. Figure S6 is a threshold titration curve. CBS event thresholds were titrated from 0.35 (most stringent) down to 0.10. Sensitivity (y-axis) represents the proportion of empirically detected events on the CNV-typing array at 1,153 McCarroll CNVs and 6,578 putative CNVs, compared to reference events at the 732 McCarroll reference CNVs and validation PCRs (listed as REF-McCarroll-Sel and pcr-GS in Additional data file 8), respectively. Because of the possibility of false-positive calls in the McCarroll *et al*. [14] study, and the small sampling size of the PCRs, the sensitivity estimates are approximations useful for comparing CBS thresholds, and not absolute measures. The x-axis, 1 - Specificity, represents the proportion of possibly false-positive events called on the CNV-typing array at McCarroll CNVs compared to reported diploid calls at the 234 non-event CNVs from the McCarroll *et al*. [14] study (listed as REF-NonPoly6papers in Additional data file 8). Any instances of false-negative events missed in the McCarroll *et al*. [14] study that were actually called in our survey will artificially lower the specificity estimates. Figure S7 are plots of event segment Log2 ratios. Log2 ratios of 97,953 event segments at the 6,368 confirmed CNVs were grouped based on rounded segment lengths (Figure S7A), or rounded numbers of probes (log2) in the segments (Figure S7B), and summarized in box-plots for either gain or loss events. Box-plots show medians and interquartile ranges, with whiskers extending to maximum or minimum values within 1.5 times the 75th or 25th percentiles, respectively. The width of boxes is proportional to the number of events. Table S3 shows breakpoint mapping. Table S3A: amplicon bands corresponding to 19 loss events at 16 regions were excised from gels and sequenced. Shown are the build 36 reference sequences 50 nucleotides upstream and downstream of the mapped breakpoints of the loss events. Differences from the reference sequence in individual Yoruba samples are in lower case. The actual lengths (len) based on the breakpoints are listed. At putative CNV locus_ids 3262, 3689, and 5439, the non-event DNA in Yoruba pairs also had actual events at the exact same breakpoints as in the event DNAs. Table S3B: the 16 regions with successful breakpoint sequences are listed along with the closest matching records in the DGV (March 2009). Table S5 is a summary of quantitative PCR results at 16 putative CNVs. DNAs were run in pairs with one having an observed gain event, and the other with no event on the genome-scan arrays. Cycle thresholds (Ct) were normalized against GAPDH PCRs, and compared in each DNA pair. In all cases, the event DNA had a lower Ct value. The status of each pair was marked based on differences in normalized Ct values: confirm or maybe (ambiguous). The differences in the Ct values were scaled, such that difference less than or equal to 0.6 are represented by one '+' symbol, and differences greater than 0.6 are represented by a proportionate number of two or more '+' symbols (Scaled_Diff_Ct). At four of the CNVs, the difference in Ct values was dramatic, indicative of homozygous losses in the non-event DNAs, rather than gains in the event DNAs. Table S6 is a list of references cited in the DGV alongside the methods used in the studies. For the pair-wise comparison shown in Figure 3, methods from the cited references were classified into the six categories. The numbers of overlapping confirmed CNVs from our work is also listed. Table S7 is a comparison of Yoruba event calls among six studies. Reported events were compared in all possible pairs of six recent studies that included one or more Yoruba individuals. Just as in the comparisons shown in Table 2, for each Yoruba in common, events were matched based on the longest overlap, and agreement was determined by comparing loss versus gain events, and not integer copy numbers. The percentage of events that overlapped reflects the relative degree of missed events in either of the studies in the paired comparisons. Click here for file

Additional data file 2 Each putative CNV identified in the genome scan was assigned a unique identifier (locus_id). CNVs with locus_id numbers starting at 100,000 were from the smoothed segmentation analysis. Chromosome locations are on genome build 36. Confirmed CNVs had at least one Yoruba with an event on the CNV-typing array. For confirmed CNVs that overlapped at least one DGV record (March 2009), the closest matching record (variation_id) is listed along with its build 36 coordinates, length, cited reference, and discovery method. Regions were flagged as 'Complex' if both a loss and gain event were observed in the same individual. Click here for file

Additional data file 3 Gel images correspond to 4% agarose (E-gel), gradient polyacrlyamide (PA gel), and 1% agarose (1% gel) electrophoresis gels. DNAs were run in pairs with one having an observed event (Event DNA_ID), and the other without an observed event (non-event DNA_ID). Confirmation calls (Call Lane 1 or 2) were made based on amplicon length differences in each DNA pair, and marked the status of each pair: confirm, maybe (ambiguous), no (no evidence of event), or fail (PCR did not yield expected amplicons). At a subset of regions, amplicon bands were excised and sequenced (seq). The lengths of the putative CNVs are also listed. Click here for file

Additional data file 4 **(A) **Event calls at confirmed CNVs are compared against consensus references from the Wang *et al*. [15] and McCarroll *et al*. [14] studies. Calls in red are in disagreement with the reference, and calls in blue are cases of possible false-positive calls not in the reference. Missed gain and loss events are shown as blue and red boxes, respectively. Consensus among the references and agreement with the references were determined by comparing loss versus gain events, and not integer copy numbers. Trio_ids are detailed in (D). **(B) **Calls reported in the McCarroll *et al*. [14] study are compared against consensus reference from our survey and the Wang *et al*. [15] study. **(C) **Calls reported in the Wang *et al*. [15] study are compared against consensus reference from our survey and the McCarroll *et al*. [14] study. **(D) **Yoruba trios were arbitrarily assigned trio_ids. The DNA_ids of the 90 Yoruba are listed with the corresponding trio_ids. Click here for file

Additional data file 5 Primer sequences, along with sizes of the expected amplicons. Click here for file

Additional data file 6 Log 2 ratios of the event segments are also listed, along with event coordinates on genome build 36. Click here for file

Additional data file 7 Observed events on the CNV-typing array in the 90 Yoruba at 1,153 CNVs reported in the McCarroll *et al*. [14] study (listed as chp-McCarroll2008) and at regions from the Levy *et al*. [18] and Wheeler *et al*. [19] studies as summarized in Table 4 (listed as chp-LevyWheel, chp-LevyOnly, and chp-WheelerOnly). Click here for file

Additional data file 8 When available, event calls were listed as integer copy numbers from 0 to 4, reported copy numbers > 4 were listed as 4, and no-calls were listed as -1. In papers that reported only loss (deletion) or gain, the calls were listed as 1 or 3, respectively. For papers with genome positions in build 35, the liftOver utility at UCSC [53] was used to map coordinates on build 36. Also listed are diploid calls in Yoruba from the McCarroll *et al*. [14] study (listed as REF-NonPoly6papers), and event calls based on PCR (listed as pcr-GS). Click here for file
