# Supplementary material for: High resolution discovery and confirmation of copy number variants in 90 Yoruba Nigerians
Source: Genome Biol. 2009 Nov 9;10(11):R125. doi: 10.1186/gb-2009-10-11-r125 (PMC3091319; doi:10.1186/gb-2009-10-11-r125)
Supplement: Additional data file 1 — Figure S1 is a description of the chip designs. Figure S1A: the sequential 49-mer probes against the genome were dispersed across the three chip designs. Probes corresponding to extraneous matches to the genome in the central 16 nucleotides were omitted from the designs. Figure S1B: probes on the CNV-typing design were organized into probe partitions corresponding to putative CNVs from the genome scan (in red), reported CNVs from whole-genome sequencing studies (Levy et al. [18] and Wheeler et al. [19]; in blue), and CNV regions in the DGV (November 2008) overlapping in at least two database records (in green). The five example partitions correspond to regions of varying length, and are represented by up to 50 probes each; regions less than 500 bp have fewer probes because the probe spacing is capped at 10 bp per probe. A partition can map to more than one CNV; conversely, a CNV can be represented by one or more partitions. Figure S2 shows regions with reported CNVs in proximity. Two example regions of width approximately 200 kb (Figure S2A) and approximately 20 kb (Figure S2B) are displayed in Nexus chromosome views (BioDiscovery), along with DGV browser views [51]. The Nexus views show the percentage of Yoruba samples with observed gains and losses in green and red, respectively. DGV records that were paired with putative CNVs are colored with blue stripes. In the first example (Figure S2A), the DGV records with red stripes more closely match the smaller CNVs. Figure S3 shows cell line artifacts. The initial smoothed segmentation analysis, displayed in Nexus (BioDiscovery) drill-down views, showed disproportionately high gain events across chromosome 12 in Yoruba sample NA19193 (Figure S3A) and chromosome 9 in sample NA19208 (Figure S3B). The green and red bars along the chromosome pictograms mark regions with gains and losses, respectively. These observations are consistent with previously reported lymphoblastoid cell-line artifacts, namely mosaic duplications, i [file gb-2009-10-11-r125-S1.PDF]

Supplementary Figure 1A.

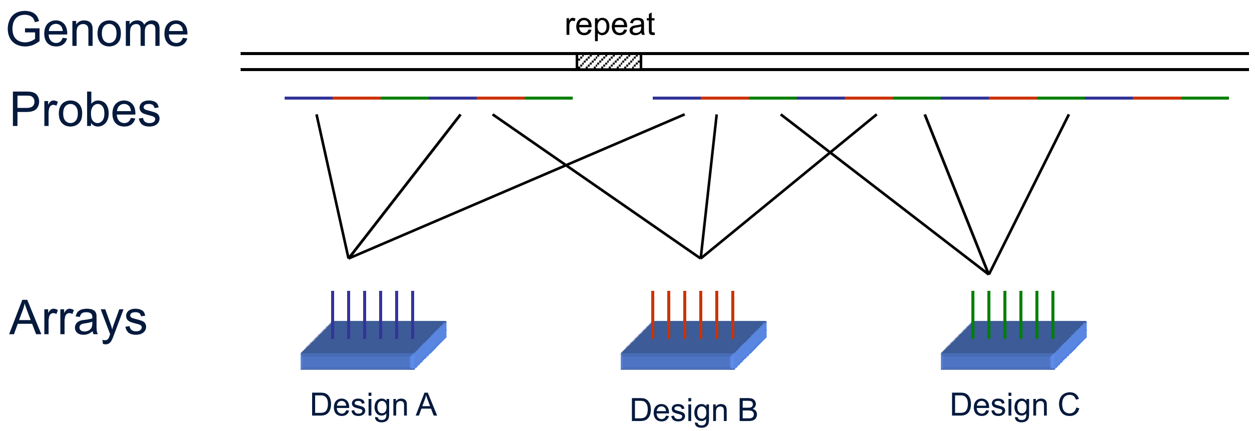

Supplementary Figure 1B.

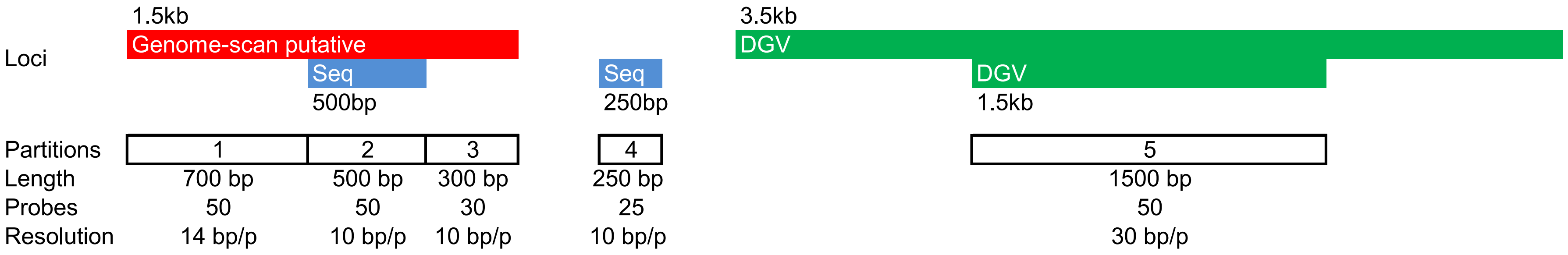

Supplementary Figure 2A.

chr13:56610607-56809449 (Group 335)

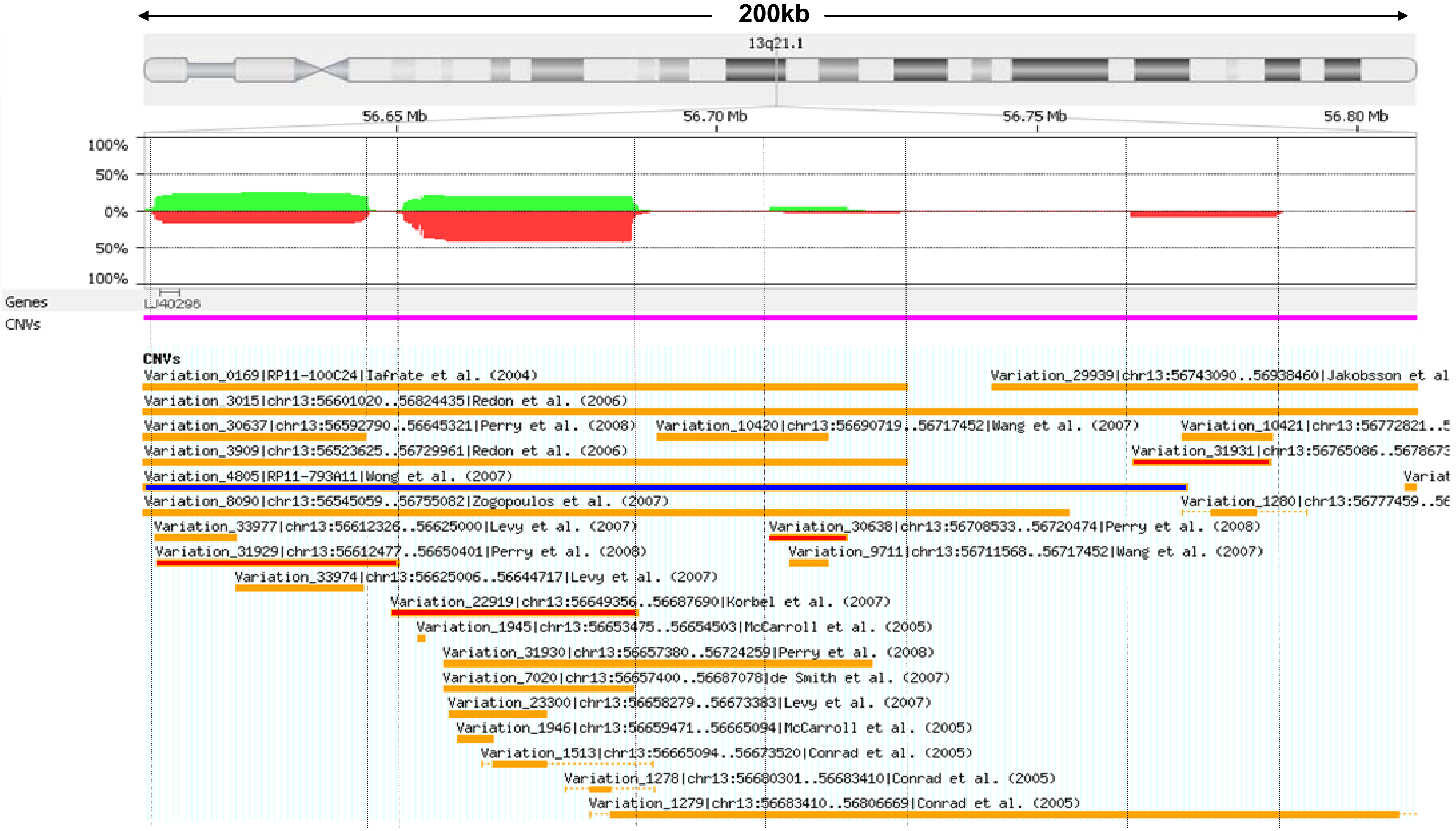

Supplementary Figure 2B.

chr11:93322975-93341693 (Group 313)

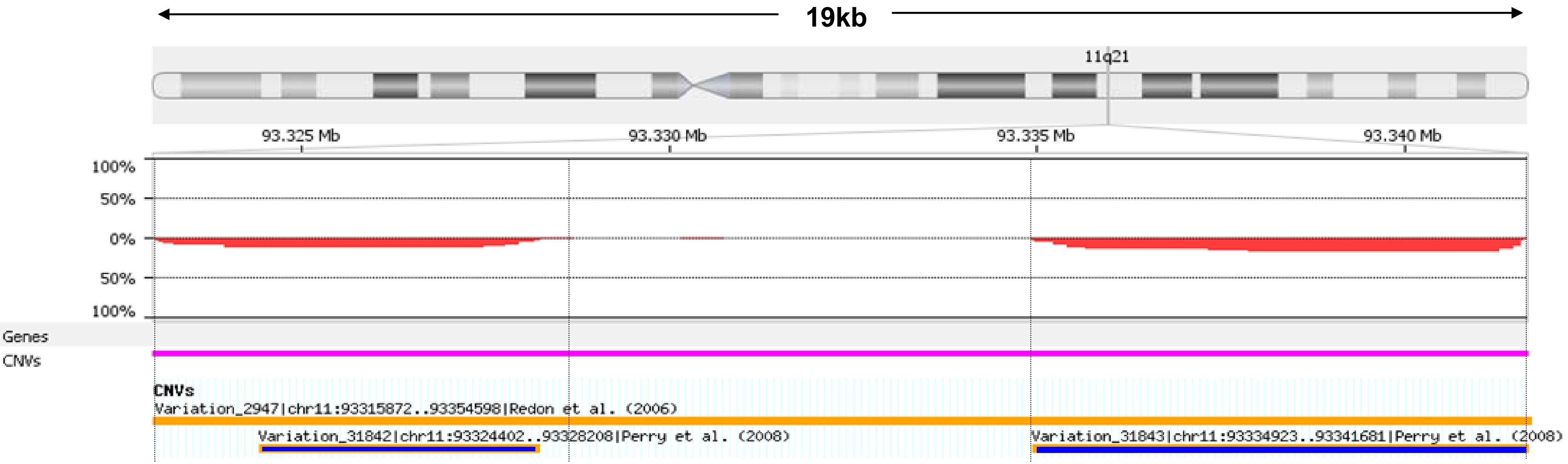

Supplementary Figure 3A.

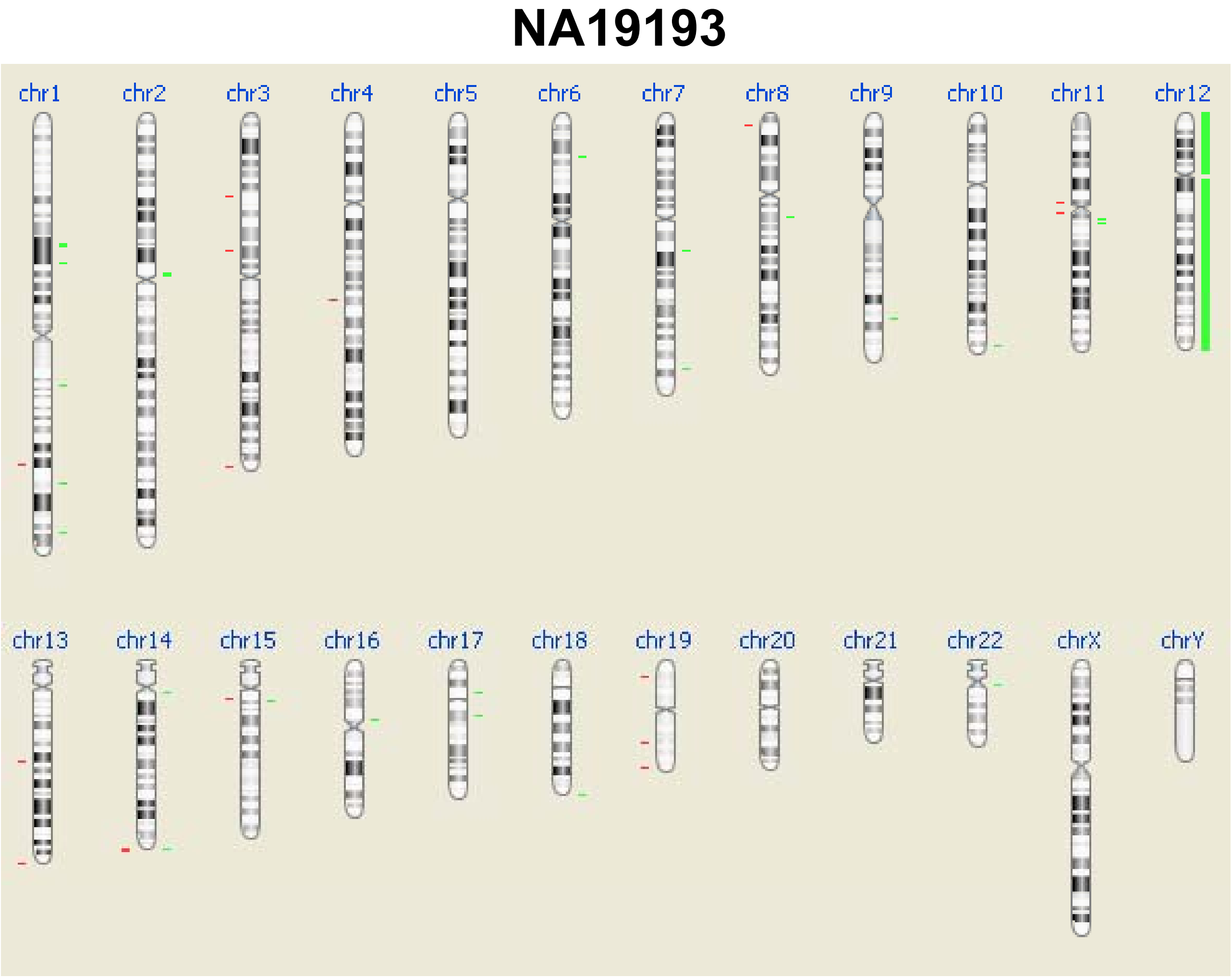

Supplementary Figure 3B.

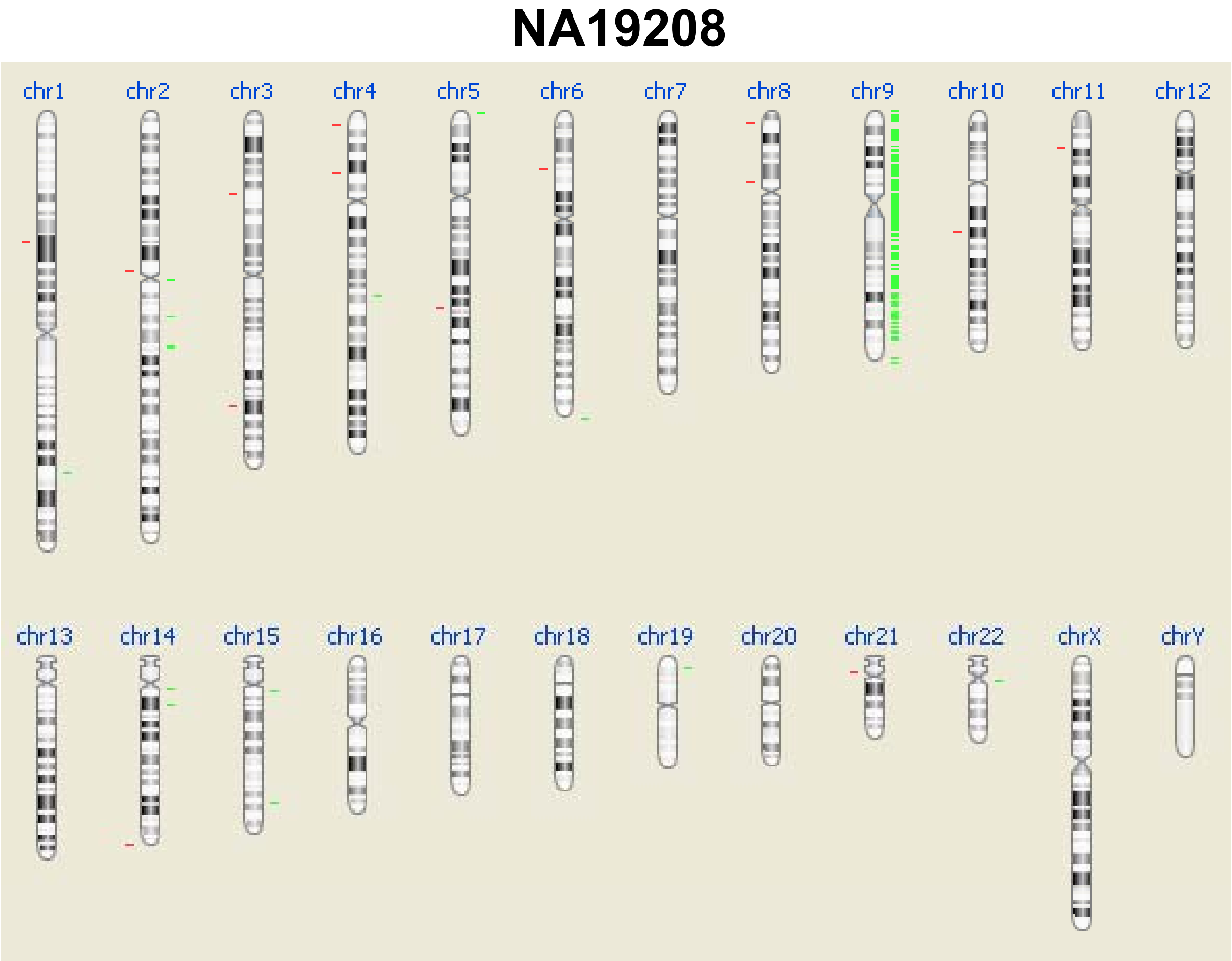

Supplementary Figure 4A.

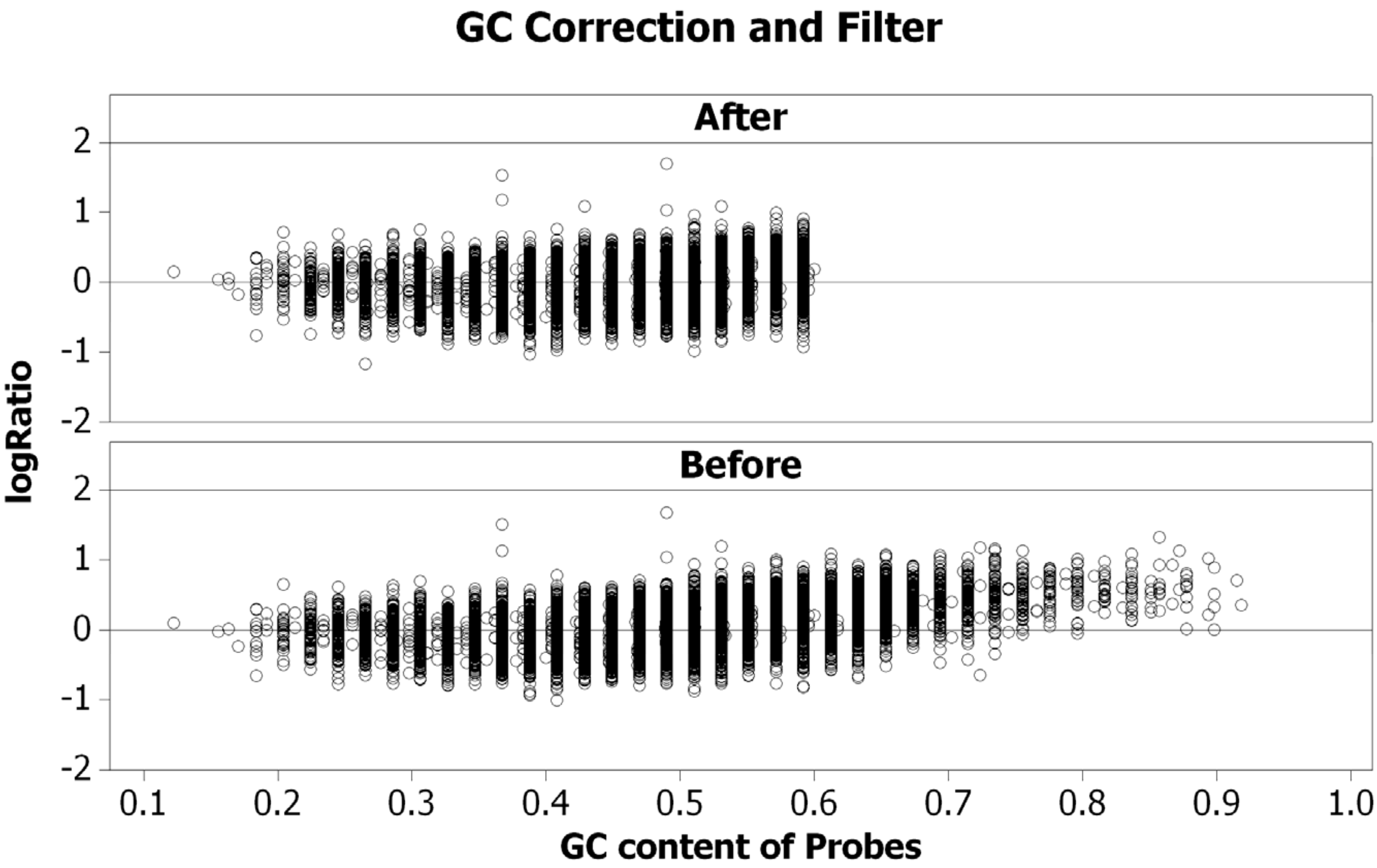

(random 20k probes from chr20, NA18870, b-chip)

Supplementary Figure 4B.

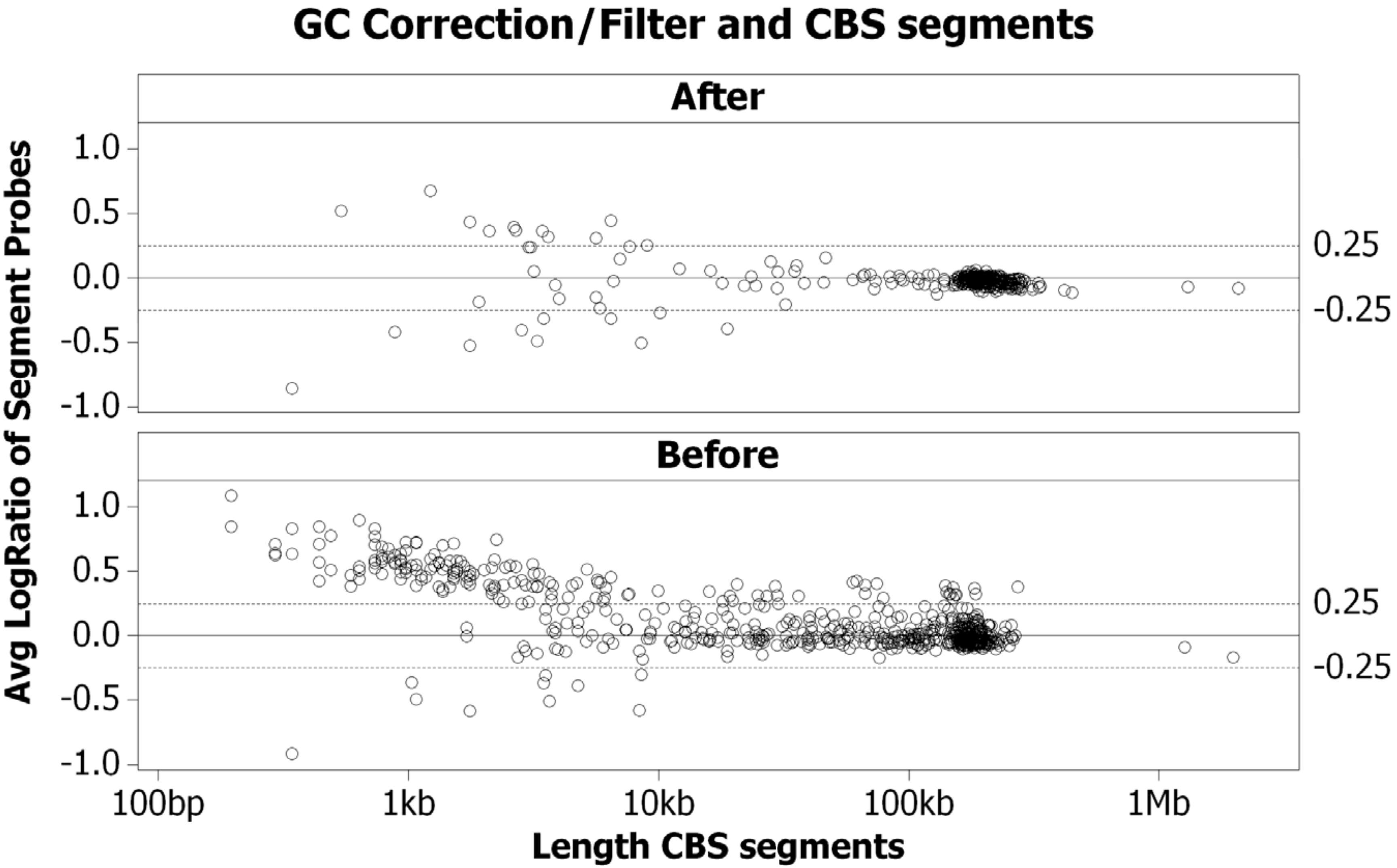

(chr20 in NA18870, b-chip)

Supplementary Figure 5A.

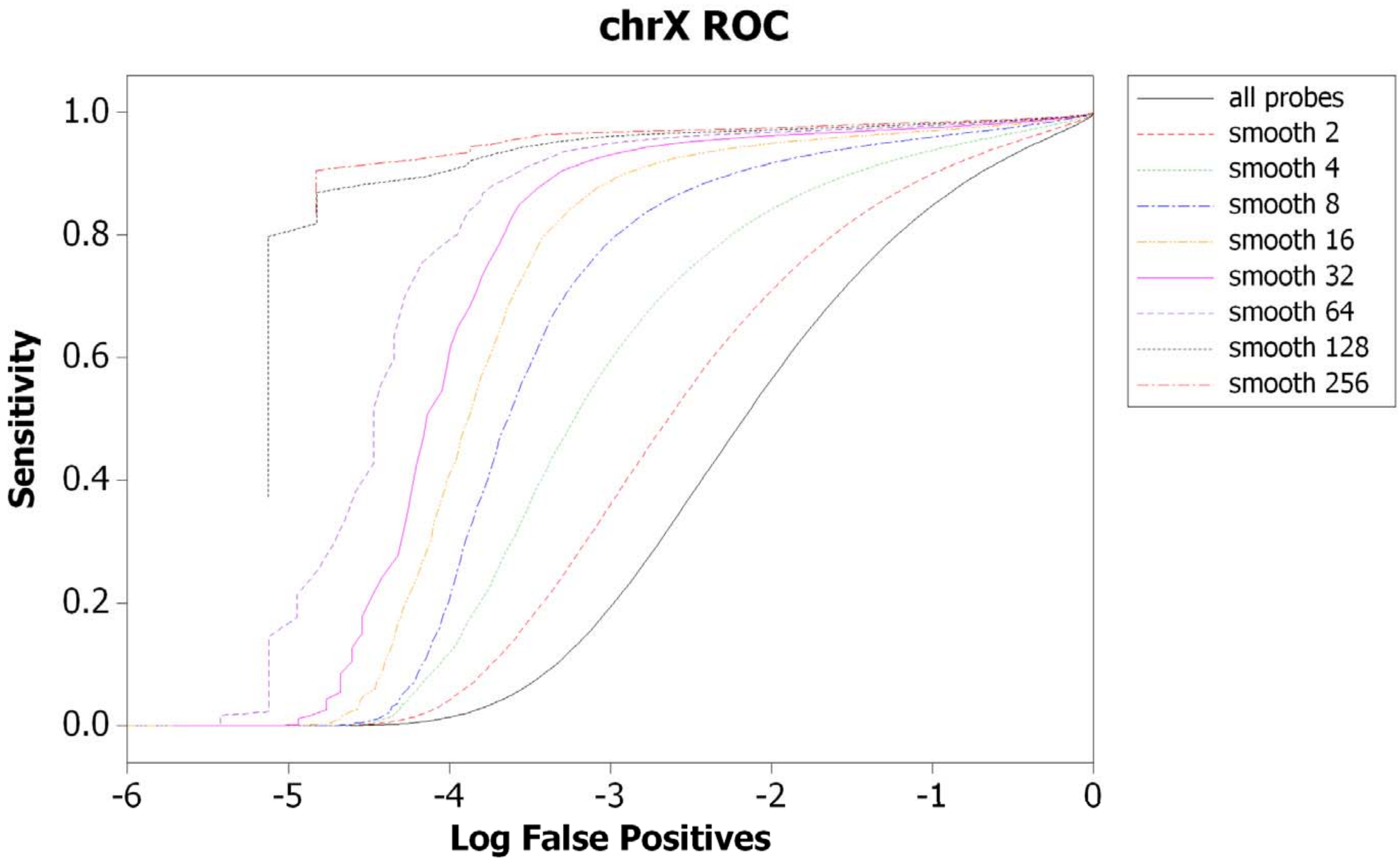

N90 Yoruba, b-chip

Supplementary Figure 5B.

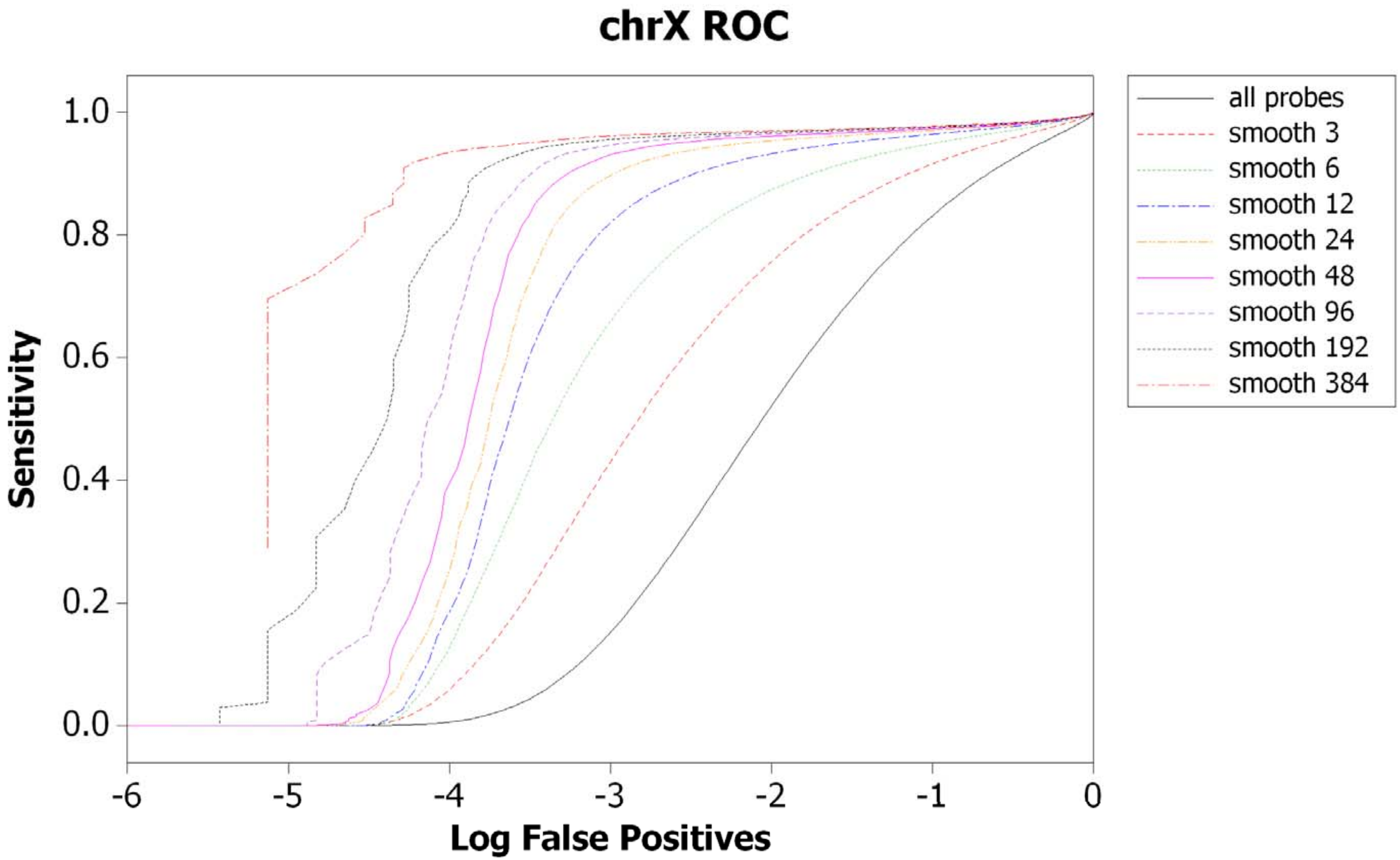

N90 Yoruba, abc

Supplementary Figure 6.

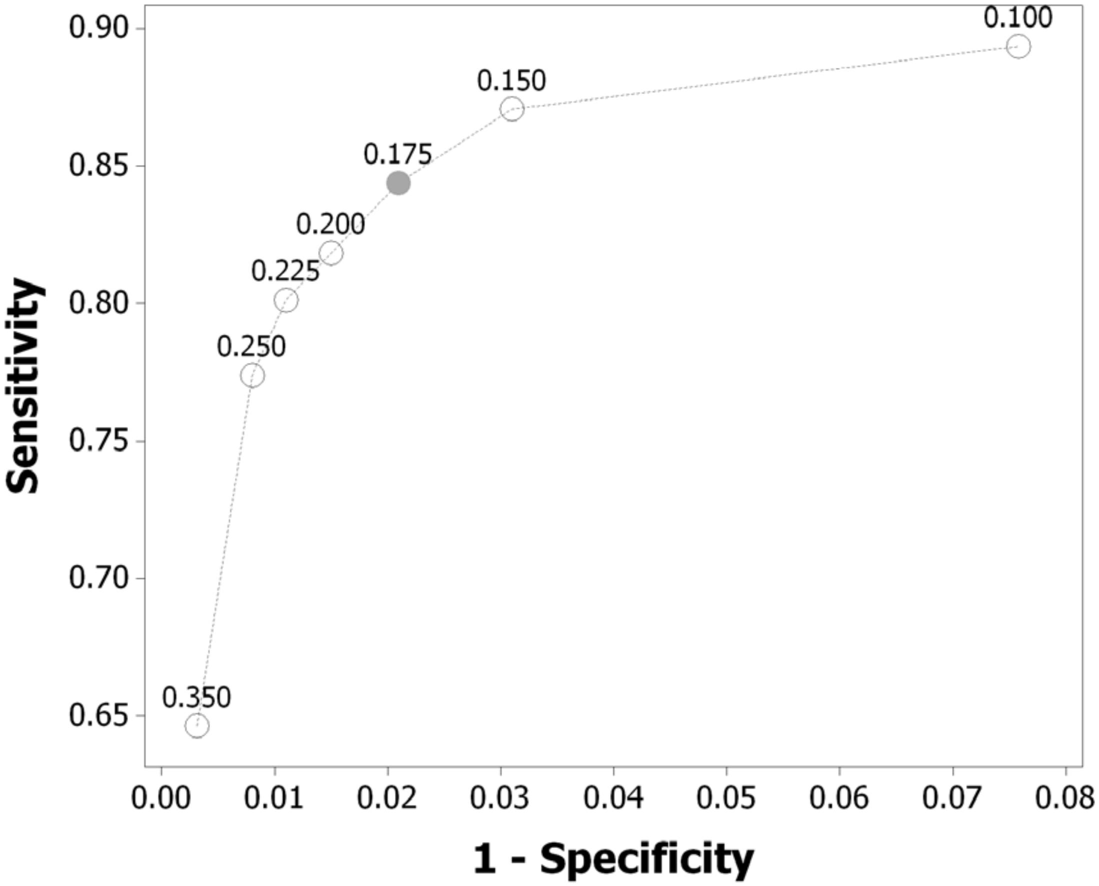

Supplementary Figure 7A.

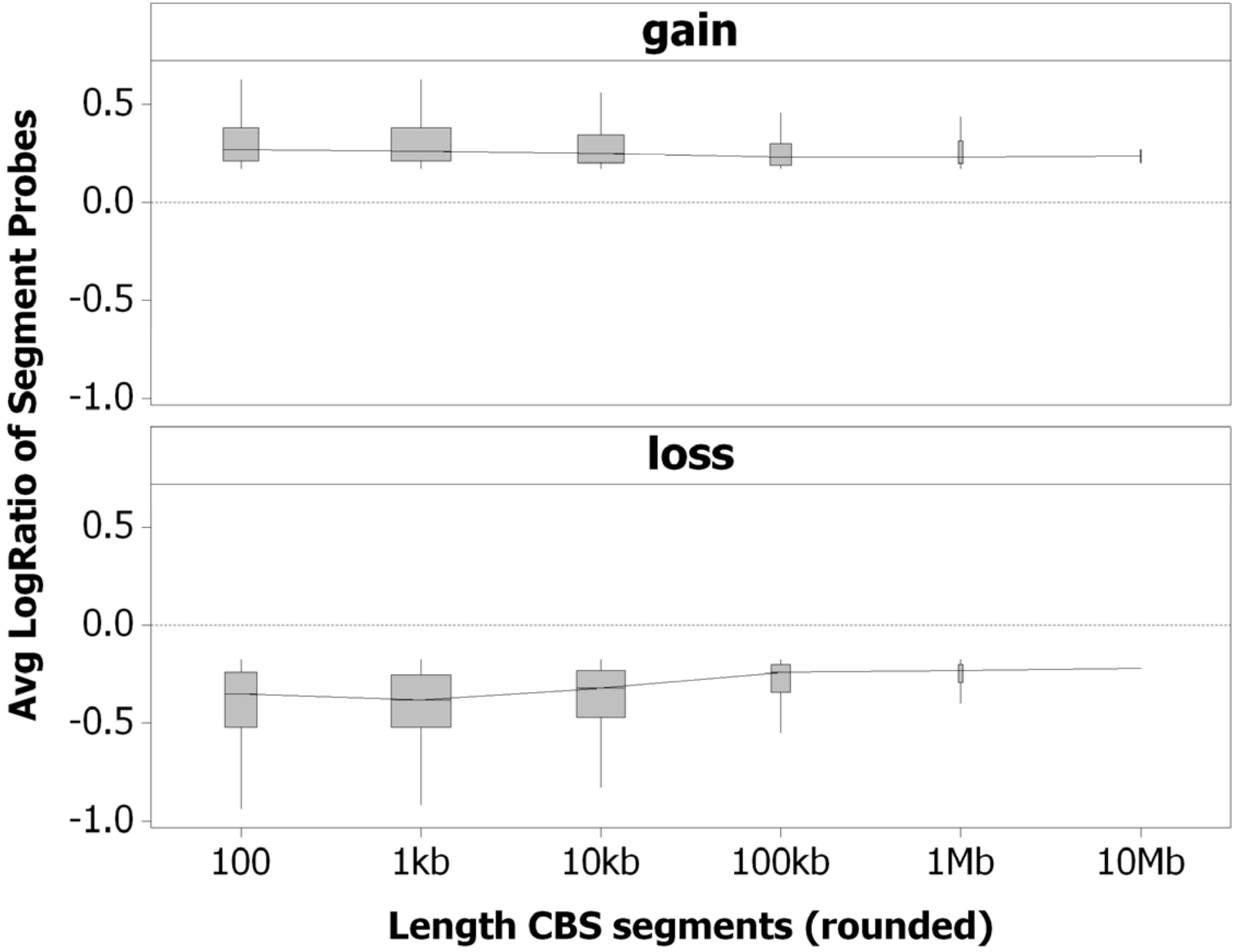

Supplementary Figure 7B.

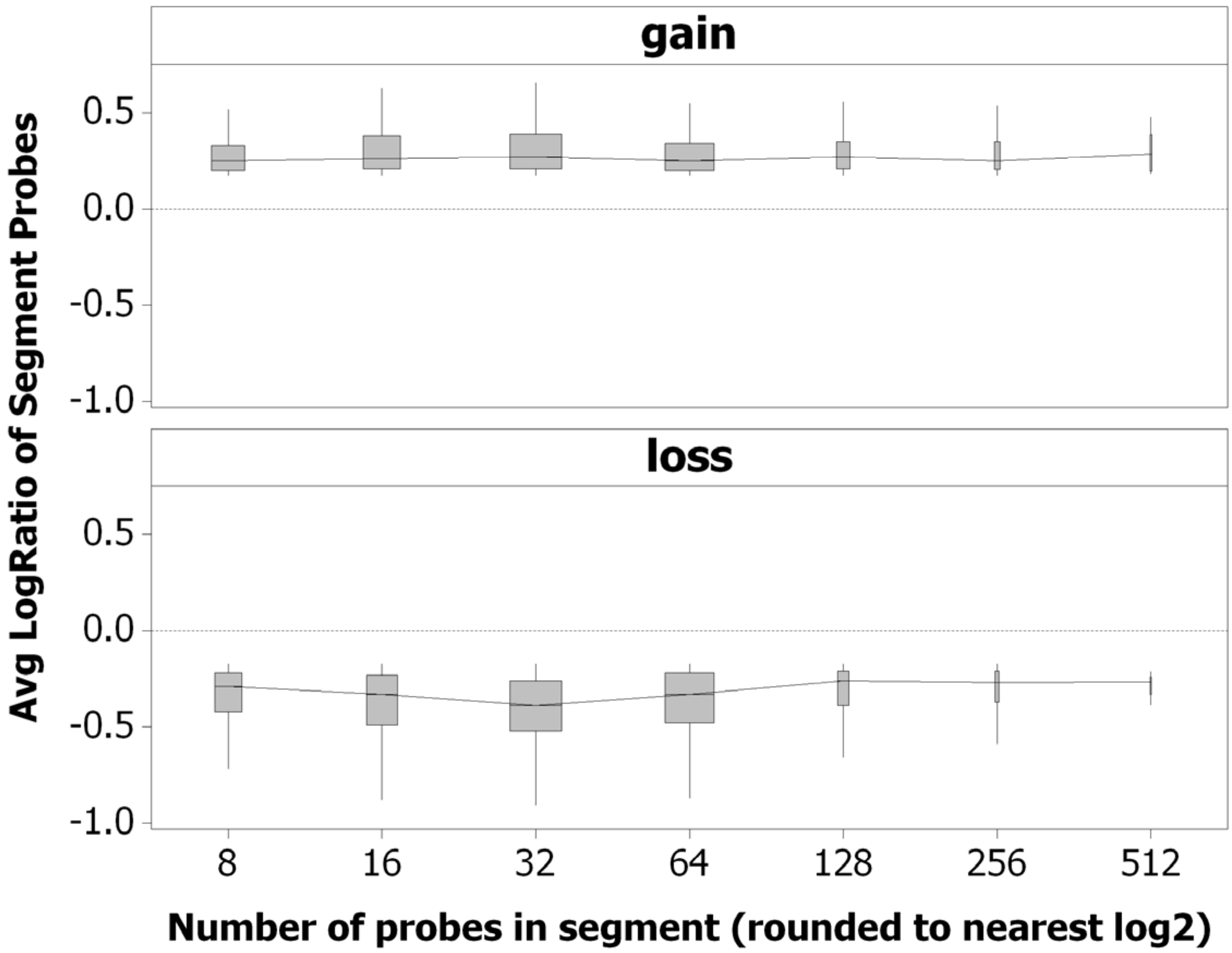

Supplementary Table 3A.

LocusID	DNA	Upstream of Breakpoint	len	Downstream of Breakpoint
756	NA18515	TAAATTTGGCACAAGAGATAGAATAGAGGTAGAGACACAGCTTTAAGAGC	1460	CACACTCTGGAATGAACATGACTTGGATAAGTAGGTGAAATAAGGGTGTC
954	NA19132	AAGGAGAATTAATCAAGGTGACAAAGGTGGTGGGGCAAAAACAGTGGGAA	1821	GAATGGTAATCGGTTCTGTTCTATTAGCCTCAGTGGTGTGAACATAGAGT
1017	NA18857	AATGGAAATCAGTGTTGTAAGTATCATCTCCCAAACACTTTTGGGAGTAC	1863	CTCAGATCTTCACCTCATCATCTGGGTCCTCCAAGCACCCCTCTACCCAC
1105	NA18857	AGTATTAATGGAAGTATAATGGTGGAGCATTTCAACCTACTTATGAGGAG	1329	TAGCAAGGAGCAGTGGTGTGATTGTTCTCAGTAGGATGTAGAAAATGTCA
2745	NA19132	CGTGTGCATGCAACTATTTGAGGAAACGCTGGAGCTGGGAGAGTCCAAGC	1017	AATCATCAACTCATCATCCCTATTGTTAAACCAAGGGAGAGCATGGTCTT
2827	NA19132	GTATTGATTTCTGTTCAATTCTATCACATGTGTAGATGCATACGATCACC	1322	AGTGAATCTGTGAAACTGTGTGGAGGTTGGGGAGGGATCAAATGGGAGAG
3157	NA18871	GGCCAGGCTGGTCTCGAACTCTTCACCTCAGGTGATCTGCCCGCCTTGGC	1545	ATCATTTGGCAAAAGCAAAACAgATCTTCTAAGGATTCCCCAAATCTTAg
3262	NA19101	CTACAAAAAGGATATATGTCATCATCCTATGATACAAAGTAAGACTCAAG	815	ACTGTACATTCTCAGGATGCATTCACAACACAGGCCATAGCTCCTGGAAT
3262	NA19132	CTACAAAAAGGAtATATGTCATCATCCTaTGATACAAAGTAAGACTCAAG	815	ACTGTACATTCTCAGGATGCATTCACAACACAGGCCATAGCTCCTGGAAT
3689	NA18508	tagagaAACGTGGAcaGCTCGCCaTATCCTATTCCTTTCACCTATATACG	1974	ATACTCTCTTTTTCTTTGATTAAACAATAAATTTATGCCATTCATCTATT
3689	NA18871	tagagaaACGTGGACaGCTCGCCATATCCTATTCCTTTCACCTATATACG	1974	ATACTCTCTTTTTCTTTGATTAAACAATAAATTTATGCCATTCATCTATT
3860	NA19132	GCCCCACTCGAGAACTCCTTCTTTCAGGAAGGACCTCACCCTCATAATAC	1484	GGGACTTCATACCTTCTACATCACTCCAGCTACTTCTATGCCTGCCAGAG
3907	NA18505	ACCTCAGCATTAGATTGGGCCTCTCCCTGAACTTGAACTGTAAAGTCAGC	1767	CTCAGTTTACTGCTCCGTAAAATAGGGATGGGATTGTTGTGATGGTGAGA
4292	NA18515	ATATAACATGTATTATATACTTTGTACAGTATCATGATGATTGCATAAGC	1499	CCATTTGAAGCAAATAGTTTCTTCTAATGCAGAAATGTGAGACCACCGGA
4582	NA18505	AGATTCAGGGCCAAAGGGGAGGTCAGAAGAGGAAGGCCCCCTGGtTTGCA	593	GCATTTGGTATGCTGTCCAGCTGAGTCCCCAaCCATGTTGTGAGTCAATG
4799	NA18505	GCTAAGATGATAAATACATCACCATTACCTAGATTGTCTTATCTCGTTGA	2085	TTATGAGTAAAAAATAAATGTCTGTGATATCAATTTCAGTGGAATCAAAT
5439	NA18515	TTTaTATCTGAAATGTAAGGTGAGGGGTTACATGATATTATCTATTAAGA	1280	CAGCCAACATGGTCACAAGtGGAGAGACTGCAGtGAAAAGAGTTTGGGTA
5439	NA18857	TTTaTATCTGAAATGTAAGGTGAGGGGTTACATGATATTATCTATTAAGA	1280	CAGCCAACATGGTCACAAGtGGAGAGACTGCAGtGAAAAGAGTTTGGGTA
6041	NA18508	tGAGTaAATGGCCCTCAAGAtTTGCTTTACTTTTCCTCCAAAATATTAGT	1381	TTTTCCCAGTGTGTATGGTGGGAAAAAAGGAATTCTATTCATAATGATTT

Supplementary Table 3B.

Locus ID	Breakpoint sequenced length	DGV length	DGV record	DGV Reference	DGV Method/platform
756	1460 bp				
954	1821 bp				
1017	1863 bp				
1105	1329 bp	1330 bp	Variation_39328	Wheeler et al. (2008)	Sequencing
2745	1017 bp				
2827	1322 bp	1047837 bp	Variation_7605	de Smith et al. (2007)	Agilent 185k CGH Arrays/Agilent Custom CGH Arrays
3157	1545 bp	1545 bp	Variation_6653	Mills et al. (2006)	Sequence trace read mapping
3262	815 bp				
3689	1974 bp	1975 bp	Variation_39055	Wheeler et al. (2008)	Sequencing
3860	1484 bp				
3907	1767 bp				
4292	1499 bp	144072 bp	Variation_4785	Wong et al. (2007)	BAC Array CGH
4582	593 bp	593 bp	Variation_11711	Mills et al. (2006)	Sequence trace read mapping
4799	2085 bp	2085 bp	Variation_5776	Mills et al. (2006)	Sequence trace read mapping
5439	1280 bp	1277 bp	Variation_43976	Bentley et al. (2008)	Illumina DNA sequencing
6041	1381 bp				

Supplementary Table 5.

LocusID	Event	EventDNA	LoCt_DNA	HiCt_DNA	**status**	Scaled_Diff_Ct
217	Gain	NA19101	NA19101	NA19132	**maybe**	+
1231	Gain	NA19192	NA19192	NA18505	**LOSS**	++++++++++++++++++++
1302	Gain	NA18505	NA18505	NA19192	**confirm**	+++
1701	Gain	NA19132	NA19132	NA19101	**confirm**	+++
1932	Gain	NA18857	NA18857	NA18515	**LOSS**	+++++++++++++++++++++++++
1950	Gain	NA18505	NA18505	NA19192	**confirm**	+++
2012	Gain	NA19101	NA19101	NA19132	**confirm**	++
2065	Gain	NA18505	NA18505	NA19192	**maybe**	+
2590	Gain	NA18505	NA18505	NA19192	**LOSS**	+++++++++++++++++++++++
2595	Gain	NA18515	NA18515	NA18857	**LOSS**	++++++++++++++++++++++++++++
2757	Gain	NA18508	NA18508	NA18871	**confirm**	+++
2854	Gain	NA19132	NA19132	NA19101	**confirm**	++
4018	Gain	NA18857	NA18857	NA18515	**maybe**	+
4799	Gain	NA19132	NA19132	NA19101	**confirm**	+++
4998	Gain	NA19132	NA19132	NA19101	**confirm**	++
5235	Gain	NA18508	NA18508	NA18871	**confirm**	++++

Supplementary Table 6.

DGV_reference	DGV_method	Classification	NumLoci
Bentley et al. (2008)	Illumina DNA sequencing	Sequencing	312
Conrad et al. (2005)	Mendelian inconsistencies	SNP_Array_Early	45
Cooper et al. (2008)	Illumina Human 1M BeadChip	SNP_Array_New	67
de Smith et al. (2007)	Agilent 185k CGH Arrays/Agilent Custom CGH Arrays	HiRes_aCGH	187
Gusev et al. (2009)	SNP genotyping analysis	SNP_Array_Early	30
Hinds et al. (2005)	Oligo arrays	SNP_Array_Early	14
Iafrate et al. (2004)	BAC Array CGH	ArrayCGH	31
Jakobsson et al. (2008)	Illumina HumanHap550 BeadChip	SNP_Array_Early	65
Kidd et al. (2008)	Paired End Mapping	Seq_Mapping	214
Korbel et al. (2007)	Paired End Mapping	Seq_Mapping	152
Levy et al. (2007)			125
	Sequencing	Sequencing	115
	Illumina HumanHap650Y BeadChip	SNP_Array_Early	7
	Agilent 244k CGH Arrays	HiRes_aCGH	2
	Affymetrix 500K SNP Mapping Array	SNP_Array_Early	1
Locke et al. (2006)	BAC Array CGH	ArrayCGH	35
McCarroll et al. (2006)			37
	Null genotypes	SNP_Array_Early	22
	Mendelian inconsistencies	SNP_Array_Early	8
	Mendelian inconsistencies/Null genotypes	SNP_Array_Early	7
McCarroll et al. (2008)	Affymetrix Human SNP Array 6.0	SNP_Array_New	385
Mills et al. (2006)	Sequence trace read mapping	Seq_Mapping	119
Perry et al. (2008)	Agilent Custom CGH Arrays	HiRes_aCGH	625
Pinto et al. (2007)	Affymetrix 500K SNP Mapping Array	SNP_Array_Early	199
Redon et al. (2006)			302
	BAC Array CGH	ArrayCGH	184
	Affymetrix 500K EA SNP Mapping Array	SNP_Array_Early	118
Sebat et al. (2004)	ROMA	ArrayCGH	13
Sharp et al. (2005)	BAC Array CGH	ArrayCGH	26
Simon-Sanchez et al. (2007)	Illumina HumanHap300 BeadChip	SNP_Array_Early	45
Tuzun et al. (2005)	Paired End Mapping	Seq_Mapping	13
Wang et al. (2007)	Illumina HumanHap550 BeadChip	SNP_Array_Early	161
Wang et al. (2008)	Illumina DNA sequencing	Sequencing	132
Wheeler et al. (2008)	Sequencing	Sequencing	96
Wong et al. (2007)	BAC Array CGH	ArrayCGH	216
Zogopoulos et al. (2007)	Affymetrix 500K and 100K SNP Mapping Arrays	SNP_Array_Early	32

Supplementary Table 7.

Study 1	Study 2	Common Yoruba	% Agreement	Events Compared	Events in Study 1	% Study 1 Compared	Events in Study 2	% Study 2 Compared
Bentley_2008	Kidd_2008	1	94.4%	71	4103	1.7%		
(Sequencing)	(Seq_Mapping)		93.0%	57			168	33.9%
Bentley_2008	McCarroll_2008	1	93.9%	49	4103	1.2%		
(Sequencing)	(SNP_Array)		95.8%	48			98	49.0%
Kidd_2008	McCarroll_2008	4	100.0%	126	938	13.4%		
(Seq_Mapping)	(SNP_Array)		100.0%	125			349	35.8%
Kidd_2008	Perry_2008	2	91.9%	99	460	21.5%		
(Seq_Mapping)	(HiRes_aCGH)		91.5%	106			1310	8.1%
Kidd_2008	Wang_2007	2	90.5%	21	460	4.6%		
(Seq_Mapping)	(SNP_Array_Early)		90.5%	21			55	38.2%
Korbel_2007	McCarroll_2008	1	88.7%	62	727	8.5%		
(Seq_Mapping)	(SNP_Array)		93.2%	59			102	57.8%
McCarroll_2008	Perry_2008	10	94.9%	455	843	54.0%		
(SNP_Array)	(HiRes_aCGH)		93.3%	511			6675	7.7%
McCarroll_2008	Wang_2007	36	100.0%	626	3087	20.3%		
(SNP_Array)	(SNP_Array_Early)		100.0%	602			1141	52.8%
Perry_2008	Wang_2007	5	100.0%	99	3063	3.2%		
(HiRes_aCGH)	(SNP_Array_Early)		100.0%	95			161	59.0%
