# Supplementary material for: High resolution discovery and confirmation of copy number variants in 90 Yoruba Nigerians
Source: Genome Biol. 2009 Nov 9;10(11):R125. doi: 10.1186/gb-2009-10-11-r125 (PMC3091319; doi:10.1186/gb-2009-10-11-r125)
Supplement: Additional data file 2 — Each putative CNV identified in the genome scan was assigned a unique identifier (locus_id). CNVs with locus_id numbers starting at 100,000 were from the smoothed segmentation analysis. Chromosome locations are on genome build 36. Confirmed CNVs had at least one Yoruba with an event on the CNV-typing array. For confirmed CNVs that overlapped at least one DGV record (March 2009), the closest matching record (variation_id) is listed along with its build 36 coordinates, length, cited reference, and discovery method. Regions were flagged as 'Complex' if both a loss and gain event were observed in the same individual. [file gb-2009-10-11-r125-S2.PDF]

locus_id	chrom	start	end	length	Yoruba w/ event	putative chr	putative start	putative end	putative len	putative type	variation_id	DGV_chr	DGV_start	DGV_end	DGV_len	Reference	Method/platform	complex
1	chr1	1379	240156	238777	15	chr1	1348	245025	243677	HiConf	Variation_0675	chr1	17845	167209	149364	Sharp et al. (2005)	BAC Array CGH	
2	chr1	346486	678853	332367	6	chr1	346455	685633	339178	HiConf	Variation_31594	chr1	346077	623339	277262	Perry et al. (2008)	Agilent Custom CGH Arrays	
3	chr1	745493	749315	3822	24	chr1	745462	749382	3920	HiConf	Variation_6833	chr1	744068	769619	25551	de Smith et al. (2007)	Agilent 185k CGH Arrays/Agilent Custom CGH Arrays	
4	chr1	803607	820076	16469	10	chr1	803576	820481	16905	single	Variation_2294	chr1	77822	1008567	930745	Redon et al. (2006)	Affymetrix 500K EA SNP Mapping Array	
5	chr1	1018374	1037386	19012	4	chr1	1018343	1037747	19404	HiConf	Variation_34671	chr1	1020714	1049928	29214	Kidd et al. (2008)	Paired End Mapping	
6	chr1	1077664	1078840	1176	40	chr1	1077633	1078858	1225	HiConf	Variation_30362	chr1	702445	1697636	995191	Perry et al. (2008)	Agilent Custom CGH Arrays	
7	chr1	1117697	1118007	310	57	chr1	1117666	1118009	343	single	Variation_0001	chr1	1083805	1283805	200000	Iafrate et al. (2004)	BAC Array CGH	
100002	chr1	1559807	1672045	112238	3	chr1	1553521	1673914	120393	HiConf	Variation_31596	chr1	1610047	1674335	64288	Perry et al. (2008)	Agilent Custom CGH Arrays	
8	chr1	1761949	1766359	4410	3	chr1	1761918	1766426	4508	single	Variation_3276	chr1	1314392	2014198	699806	Redon et al. (2006)	BAC Array CGH	
9	chr1	2041396	2045573	4177	24	chr1	2041365	2045579	4214	single	Variation_43449	chr1	2043376	2045793	2417	Wang et al. (2008)	Illumina DNA sequencing	
10	chr1	2758266	2759099	833	56	chr1	2758235	2759117	882	HiConf								
11	chr1	3205636	3206322	686	42	chr1	3205605	3206340	735	single	Variation_44568	chr1	3205492	3206251	759	Bentley et al. (2008)	Illumina DNA sequencing	
12	chr1	3646048	3646498	450	40	chr1	3646017	3646507	490	single	Variation_4197	chr1	3567200	3710699	143499	Wong et al. (2007)	BAC Array CGH	
13	chr1	3738413	3738952	539	45	chr1	3738382	3738970	588	single	Variation_44573	chr1	3738819	3738966	147	Bentley et al. (2008)	Illumina DNA sequencing	
100003	chr1	5095204	5148024	52820	22	chr1	5091738	5151126	59388	single								
14	chr1	5201896	5204101	2205	5	chr1	5201865	5204119	2254	HiConf	Variation_3279	chr1	5189627	5459265	269638	Redon et al. (2006)	BAC Array CGH	
15	chr1	5259275	5261529	2254	2	chr1	5259244	5261547	2303	single	Variation_3279	chr1	5189627	5459265	269638	Redon et al. (2006)	BAC Array CGH	
16	chr1	5505647	5512850	7203	19	chr1	5505616	5512966	7350	single								
17	chr1	5648629	5658674	10045	13	chr1	5648598	5658888	10290	HiConf								
18	chr1	5791611	5792297	686	10	chr1	5791580	5792315	735	HiConf	Variation_29826	chr1	5733711	5836208	102497	Jakobsson et al. (2008)	Illumina HumanHap550 BeadChip	
19	chr1	6417439	6446434	28995	3	chr1	6417408	6446808	29400	HiConf	Variation_31598	chr1	6421414	6437921	16507	Perry et al. (2008)	Agilent Custom CGH Arrays	
20	chr1	6543224	6543319	95	24	chr1	6543191	6543681	490	single	Variation_4202	chr1	6508456	6570177	61721	Wong et al. (2007)	BAC Array CGH	
21	chr1	6710263	6724032	13769	4	chr1	6710232	6724295	14063	HiConf	Variation_0992	chr1	6717817	6720271	2454	Conrad et al. (2005)	Mendelian inconsistencies	
22	chr1	8104313	8113672	9359	8	chr1	8104282	8113837	9555	HiConf	Variation_37859	chr1	8105171	8111872	6701	McCarroll et al. (2008)	Affymetrix Human SNP Array 6.0	
23	chr1	8473136	8473626	490	39	chr1	8473105	8473644	539	HiConf								y
24	chr1	8593333	8607445	14112	35	chr1	8593302	8607708	14406	HiConf								
25	chr1	8689079	8697164	8085	2	chr1	8689048	8697329	8281	HiConf	Variation_0003	chr1	8668859	8812827	143968	Iafrate et al. (2004)	BAC Array CGH	
26	chr1	10288341	10301056	12715	20	chr1	10288310	10301442	13132	HiConf	Variation_22833	chr1	10291453	10301596	10143	Korbel et al. (2007)	Paired End Mapping	
27	chr1	10405180	10406039	859	40	chr1	10405175	10406057	882	HiConf	Variation_33499	chr1	10405335	10406033	698	Perry et al. (2008)	Agilent Custom CGH Arrays	
28	chr1	10760407	10760807	400	10	chr1	10760376	10760817	441	single	Variation_4205	chr1	10627398	10782506	155108	Wong et al. (2007)	BAC Array CGH	
29	chr1	11325867	11330767	4900	6	chr1	11325836	11330883	5047	HiConf								
30	chr1	12666017	12668663	2646	8	chr1	12665986	12668730	2744	HiConf	Variation_43735	chr1	12665988	12669219	3231	Bentley et al. (2008)	Illumina DNA sequencing	
31	chr1	12771612	12783660	12048	5	chr1	12771581	12784174	12593	single	Variation_1537	chr1	12783336	12790824	7488	McCarroll et al. (2005)	Mendelian inconsistencies	
32	chr1	12938114	12946850	8736	26	chr1	12938083	12947197	9114	single	Variation_31602	chr1	12939392	13064453	125061	Perry et al. (2008)	Agilent Custom CGH Arrays	
33	chr1	12959968	12960175	207	3	chr1	12959937	12960182	245	single	Variation_31602	chr1	12939392	13064453	125061	Perry et al. (2008)	Agilent Custom CGH Arrays	
34	chr1	12960285	12972343	12058	34	chr1	12960280	12973069	12789	HiConf	Variation_31602	chr1	12939392	13064453	125061	Perry et al. (2008)	Agilent Custom CGH Arrays	
35	chr1	13045081	13046894	1813	11	chr1	13045050	13046912	1862	single	Variation_31602	chr1	12939392	13064453	125061	Perry et al. (2008)	Agilent Custom CGH Arrays	
36	chr1	13069429	13134947	65518	7	chr1	13069354	13136288	66934	HiConf	Variation_31603	chr1	13066951	13093155	26204	Perry et al. (2008)	Agilent Custom CGH Arrays	
37	chr1	13140360	13205433	65073	4	chr1	13140012	13207093	67081	HiConf	Variation_31604	chr1	13101084	13223981	122897	Perry et al. (2008)	Agilent Custom CGH Arrays	
38	chr1	13211355	13232158	20803	20	chr1	13210964	13232475	21511	HiConf	Variation_6779	chr1	13101085	13226052	124967	de Smith et al. (2007)	Agilent 185k CGH Arrays/Agilent Custom CGH Arrays	
39	chr1	13316688	13343491	26803	10	chr1	13316657	13344048	27391	single	Variation_38812	chr1	13317495	13340609	23114	McCarroll et al. (2008)	Affymetrix Human SNP Array 6.0	
40	chr1	13345794	13347607	1813	20	chr1	13345763	13347625	1862	HiConf	Variation_31605	chr1	13345302	13366853	21551	Perry et al. (2008)	Agilent Custom CGH Arrays	
41	chr1	13543166	13548703	5537	10	chr1	13543135	13548819	5684	single	Variation_30371	chr1	13420643	13621823	201180	Perry et al. (2008)	Agilent Custom CGH Arrays	
42	chr1	13566098	13568646	2548	23	chr1	13566067	13568713	2646	single	Variation_31607	chr1	13566138	13587686	21548	Perry et al. (2008)	Agilent Custom CGH Arrays	
43	chr1	13646688	13649300	2612	19	chr1	13645790	13649367	3577	single	Variation_31609	chr1	13646732	13649228	2496	Perry et al. (2008)	Agilent Custom CGH Arrays	
44	chr1	14035714	14039438	3724	21	chr1	14035683	14039505	3822	HiConf								
100005	chr1	14181636	14243131	61495	3	chr1	14181605	14244399	62794	HiConf	Variation_2299	chr1	14177669	14244987	67318	Redon et al. (2006)	Affymetrix 500K EA SNP Mapping Array	y
46	chr1	14308987	14311887	2900	46	chr1	14308956	14311896	2940	HiConf	Variation_43404	chr1	14309627	14311615	1988	Wang et al. (2008)	Illumina DNA sequencing	
47	chr1	14852054	14855386	3332	19	chr1	14852023	14855453	3430	single								
48	chr1	15359792	15364692	4900	4	chr1	15359761	15364808	5047	HiConf	Variation_37850	chr1	15361772	15362873	1101	McCarroll et al. (2008)	Affymetrix Human SNP Array 6.0	
49	chr1	16684115	16719640	35525	11	chr1	16684084	16720344	36260	HiConf	Variation_30374	chr1	16705187	16720593	15406	Perry et al. (2008)	Agilent Custom CGH Arrays	
50	chr1	16720695	16746443	25748	19	chr1	16720687	16746951	26264	single	Variation_4208	chr1	16738731	16782708	43977	Wong et al. (2007)	BAC Array CGH	y
51	chr1	16798824	16916925	118101	32	chr1	16798793	16917814	119021	HiConf	Variation_8321	chr1	16767863	16918233	150370	Pinto et al. (2007)	Affymetrix 500K SNP Mapping Array	
52	chr1	16920050	16996539	76489	38	chr1	16920019	16998076	78057	HiConf	Variation_0005	chr1	16880179	16998245	118066	Iafrate et al. (2004)	BAC Array CGH	y
53	chr1	17048332	17152568	104236	16	chr1	17048301	17153112	104811	HiConf	Variation_4210	chr1	17049946	17153536	103590	Wong et al. (2007)	BAC Array CGH	
54						chr1	17168792	17182414	13622	HiConf								
55	chr1	18555964	18556324	360	51	chr1	18555933	18556325	392	single								
56	chr1	19000541	19001129	588	14	chr1	19000510	19001147	637	single								y
57	chr1	20279343	20283704	4361	12	chr1	20279312	20283771	4459	HiConf	Variation_43756	chr1	20279673	20283272	3599	Bentley et al. (2008)	Illumina DNA sequencing	
58	chr1	21181482	21191233	9751	19	chr1	21181451	21191447	9996	HiConf								
100007	chr1	21216073	21260250	44177	1	chr1	21207054	21261346	54292	HiConf								
59	chr1	21610379	21627455	17076	14	chr1	21610348	21627645	17297	HiConf	Variation_30378	chr1	21610807	21671935	61128	Perry et al. (2008)	Agilent Custom CGH Arrays	y
60	chr1	21640220	21678001	37781	37	chr1	21640189	21678409	38220	HiConf	Variation_31611	chr1	21654568	21666231	11663	Perry et al. (2008)	Agilent Custom CGH Arrays	
100009						chr1	21771338	21846479	75141	HiConf								
100010	chr1	22165495	22210530	45035	4	chr1	22165175	22211725	46550	HiConf	Variation_31612	chr1	22182702	22213247	30545	Perry et al. (2008)	Agilent Custom CGH Arrays	
62	chr1	22785129	22788369	3240	2	chr1	22772138	22790170	18032	HiConf								
63	chr1	23965025	23972032	7007	5	chr1	23964994	23972148	7154	HiConf								
64	chr1	24081449	24084389	2940	3	chr1	24081418	24084456	3038	single								
65	chr1	24677926	24679396	1470	17	chr1	24677895	24679414	1519	HiConf	Variation_34800	chr1	24667583	24687729	20146	Kidd et al. (2008)	Paired End Mapping	
66	chr1	24765538	24774505	8967	11	chr1	24765507	24774670	9163	HiConf	Variation_22693	chr1	24768045	24774830	6785	Korbel et al. (2007)	Paired End Mapping	
67	chr1	25106725	25107313	588	41	chr1	25106694	25107331	637	HiConf								
68	chr1	25358193	25372011	13818	1	chr1	25358162	25372274	14112	single								y
69	chr1	25458055	25537247	79192	18	chr1	25458024	25537257	79233	HiConf	Variation_6821	chr1	25457932	25537445	79513	de Smith et al. (2007)	Agilent 185k CGH Arrays/Agilent Custom CGH Arrays	
70	chr1	25561156	25623878	62722	4	chr1	25561120	25625261	64141	HiConf	Variation_31615	chr1	25561644	25566191	4547	Perry et al. (2008)	Agilent Custom CGH Arrays	
71	chr1	26141458	26141566	108	19	chr1	26141427	26141574	147	single								
72	chr1	26334812	26336821	2009	37	chr1	26334781	26336839	2058	HiConf	Variation_34804	chr1	26328474	26341412	12938	Kidd et al. (2008)	Paired End Mapping	
73	chr1	26762876	26764444	1568	22	chr1	26762845	26764462	1617	single								
100012						chr1	27708055	27840355	132300	HiConf								
74	chr1	28108073	28126026	17953	4	chr1	28108042	28126123	18081	single								
75	chr1	29177253	29178625	1372	18	chr1	29177222	29178643	1421	HiConf								
76	chr1	29223901	29224587	686	6	chr1	29223870	29224605	735	single	Variation_38989	chr1	29205353	29235370	30017	Wheeler et al. (2008)	Sequencing	
77	chr1	29710961	29712044	1083	4	chr1	29710930	29713821	2891	single	Variation_34811	chr1	29708903	29740480	31577	Kidd et al. (2008)	Paired End Mapping	
78	chr1	29714083	29714321	238	3	chr1	29713968	29714703	735	single	Variation_34811	chr1	29708903	29740480	31577	Kidd et al. (2008)	Paired End Mapping	
100013	chr1	30866759	30873050	6291	2	chr1	30853561	30888229	34668	single								
79	chr1	30942429	30943752	1323	22	chr1	30942398	30943770	1372	HiConf								
80	chr1	31047485	31057530	10045	3	chr1	31047454	31057744	10290	HiConf								

locus_id	chrom	start	end	length	Yoruba w/ event	putative chr	putative start	putative end	putative len	putative type	variation_id	DGV_chr	DGV_start	DGV_end	DGV_len	Reference	Method/platform	complex
81	chr1	31632251	31633245	994	18	chr1	31632220	31633249	1029	HiConf	Variation_24184	chr1	31632360	31632734	374	Levy et al. (2007)	Sequencing	
82	chr1	31677135	31690855	13720	19	chr1	31677104	31691118	14014	HiConf	Variation_3287	chr1	31494727	32129437	634710	Redon et al. (2006)	BAC Array CGH	
100014						chr1	31870189	32026425	156236	HiConf								
83	chr1	32823147	32828880	5733	4	chr1	32823116	32828996	5880	HiConf								
84	chr1	34869975	34884430	14455	21	chr1	34869944	34884742	14798	HiConf	Variation_6823	chr1	34867574	34883404	15830	de Smith et al. (2007)	Agilent 185k CGH Arrays/Agilent Custom CGH Arrays	y
100015	chr1	35133767	35213455	79688	6	chr1	35133736	35215076	81340	single								
85	chr1	36565081	36574244	9163	4	chr1	36565050	36574409	9359	HiConf	Variation_3288	chr1	36392726	36659675	266949	Redon et al. (2006)	BAC Array CGH	
86	chr1	38414929	38417526	2597	7	chr1	38414898	38417593	2695	HiConf	Variation_38178	chr1	38414624	38417452	2828	McCarroll et al. (2008)	Affymetrix Human SNP Array 6.0	
87	chr1	38642093	38661889	19796	3	chr1	38642062	38662299	20237	single								
88	chr1	38857154	38858085	931	9	chr1	38857123	38858103	980	HiConf								
100016	chr1	39042279	39111840	69561	5	chr1	39032666	39113197	80531	single								
89	chr1	39307317	39309228	1911	5	chr1	39307286	39309246	1960	HiConf								
90	chr1	40350968	40359396	8428	3	chr1	40350937	40359561	8624	HiConf	Variation_4219	chr1	40311292	40504667	193375	Wong et al. (2007)	BAC Array CGH	
91	chr1	40794712	40800984	6272	12	chr1	40794681	40801100	6419	HiConf	Variation_5488	chr1	40794272	40801210	6938	Mills et al. (2006)	Sequence trace read mapping	
92	chr1	41534661	41537797	3136	3	chr1	41534630	41537864	3234	HiConf								y
93	chr1	41764618	41769812	5194	34	chr1	41764587	41769928	5341	HiConf	Variation_44579	chr1	41766663	41766930	267	Bentley et al. (2008)	Illumina DNA sequencing	
94	chr1	41844110	41845076	966	9	chr1	41844016	41845094	1078	HiConf								
95	chr1	42421955	42424942	2987	2	chr1	42421922	42425009	3087	single								
96	chr1	43466731	43467858	1127	20	chr1	43466700	43467876	1176	HiConf	Variation_43776	chr1	43466652	43468133	1481	Bentley et al. (2008)	Illumina DNA sequencing	
97	chr1	43533205	43534789	1584	2	chr1	43530204	43535202	4998	single								
98	chr1	43794890	43796663	1773	1	chr1	43793677	43803575	9898	HiConf								
99						chr1	43803771	43812052	8281	HiConf								
100	chr1	43817950	43820550	2600	1	chr1	43812199	43825233	13034	single								
101	chr1	43834084	43843982	9898	1	chr1	43834053	43844196	10143	HiConf								
102	chr1	43864709	43882045	17336	5	chr1	43864678	43882808	18130	HiConf	Variation_37648	chr1	43868287	43878235	9948	McCarroll et al. (2008)	Affymetrix Human SNP Array 6.0	
103	chr1	44109562	44109872	310	51	chr1	44109531	44109874	343	single								
104	chr1	44228926	44233189	4263	2	chr1	44223211	44233403	10192	HiConf								y
105	chr1	44246074	44251716	5642	8	chr1	44233550	44253738	20188	single								
106	chr1	44461284	44473289	12005	1	chr1	44461253	44473503	12250	single								
107	chr1	44480302	44483606	3304	2	chr1	44478501	44484430	5929	single								
108	chr1	46016936	46024691	7755	5	chr1	46016905	46025186	8281	HiConf	Variation_22817	chr1	46015346	46024503	9157	Korbel et al. (2007)	Paired End Mapping	
109	chr1	46486699	46487336	637	20	chr1	46486668	46487354	686	single								
110	chr1	47423040	47430390	7350	25	chr1	47423009	47430555	7546	HiConf								y
111	chr1	47974731	47987716	12985	13	chr1	47974700	47987979	13279	single								
112	chr1	48241732	48243545	1813	3	chr1	48241701	48243563	1862	HiConf								
100018	chr1	48414299	48428586	14287	1	chr1	48382111	48441670	59559	HiConf								
113	chr1	48494866	48496924	2058	21	chr1	48494835	48496942	2107	HiConf	Variation_43777	chr1	48494706	48496264	1558	Bentley et al. (2008)	Illumina DNA sequencing	
100019	chr1	48746775	48798172	51397	8	chr1	48746744	48799150	52406	single								
114	chr1	49392105	49392555	450	3	chr1	49392074	49392564	490	single								
115	chr1	51058632	51089808	31176	4	chr1	51058564	51090463	31899	single								
116	chr1	51687902	51690044	2142	8	chr1	51687871	51691056	3185	HiConf								
100020	chr1	51947088	51979575	32487	3	chr1	51947057	51980254	33197	single								
118	chr1	52042760	52043210	450	5	chr1	52042729	52043219	490	single								
100021	chr1	52104917	52202770	97853	41	chr1	52104886	52204748	99862	HiConf	Variation_4223	chr1	52078423	52187326	108903	Wong et al. (2007)	BAC Array CGH	
120	chr1	53333861	53334121	260	13	chr1	53333830	53334124	294	HiConf								
121	chr1	53334351	53340065	5714	3	chr1	53334320	53340102	5782	HiConf	Variation_39601	chr1	53336468	53336664	196	Wheeler et al. (2008)	Sequencing	
122	chr1	53367034	53367124	90	22	chr1	53367003	53367493	490	single								
123						chr1	53371462	53375725	4263	single								
124	chr1	54124427	54125848	1421	9	chr1	54124396	54125866	1470	single								
125	chr1	54140352	54141773	1421	12	chr1	54140321	54141791	1470	single								
126	chr1	54384813	54387753	2940	7	chr1	54384782	54387820	3038	HiConf								
127	chr1	54864866	54866951	2085	17	chr1	54864835	54866991	2156	HiConf	Variation_38995	chr1	54864862	54868555	3693	Wheeler et al. (2008)	Sequencing	
128	chr1	55073851	55079094	5243	8	chr1	55073820	55079210	5390	HiConf	Variation_38182	chr1	55074411	55078004	3593	McCarroll et al. (2008)	Affymetrix Human SNP Array 6.0	
129	chr1	55255342	55268038	12696	13	chr1	55240959	55268595	27636	HiConf								y
130	chr1	55608980	55633431	24451	34	chr1	55608949	55633939	24990	HiConf	Variation_31623	chr1	55620561	55743716	123155	Perry et al. (2008)	Agilent Custom CGH Arrays	
131	chr1	56864899	56871563	6664	3	chr1	56864868	56871679	6811	single								
132	chr1	56871906	56873180	1274	5	chr1	56871875	56873198	1323	single								
133	chr1	56873376	56878962	5586	5	chr1	56873345	56879078	5733	single								
134	chr1	58420208	58421580	1372	8	chr1	58420177	58421598	1421	single								
135	chr1	58516757	58517326	569	52	chr1	58516756	58517344	588	HiConf	Variation_44597	chr1	58516499	58517408	909	Bentley et al. (2008)	Illumina DNA sequencing	
136	chr1	59810289	59822105	11816	23	chr1	59810258	59831377	21119	HiConf	Variation_37396	chr1	59819356	59822221	2865	Cooper et al. (2008)	Illumina Human 1M BeadChip	
100023						chr1	61278470	61338740	60270	single								
137	chr1	61855500	61856235	735	52	chr1	61855469	61856253	784	HiConf	Variation_44599	chr1	61855454	61856293	839	Bentley et al. (2008)	Illumina DNA sequencing	
138	chr1	62208643	62224522	15879	9	chr1	62208612	62225125	16513	HiConf	Variation_31625	chr1	62209141	62223645	14504	Perry et al. (2008)	Agilent Custom CGH Arrays	
139	chr1	63387289	63388656	1367	9	chr1	63387258	63388679	1421	HiConf								
140	chr1	63507094	63508198	1104	3	chr1	63507063	63508288	1225	HiConf								y
141	chr1	64609594	64623479	13885	37	chr1	64609563	64623724	14161	HiConf	Variation_37913	chr1	64615315	64624133	8818	McCarroll et al. (2008)	Affymetrix Human SNP Array 6.0	
142	chr1	64624931	64627194	2263	6	chr1	64624900	64628575	3675	HiConf								
143	chr1	64628753	64628816	63	12	chr1	64628722	64628820	98	single								
144	chr1	65619925	65628500	8575	3	chr1	65619894	65628665	8771	HiConf								
145	chr1	65811172	65815974	4802	12	chr1	65811141	65816041	4900	HiConf	Variation_43779	chr1	65813532	65816494	2962	Bentley et al. (2008)	Illumina DNA sequencing	
146	chr1	67781070	67781330	260	54	chr1	67781039	67781333	294	HiConf	Variation_44611	chr1	67780558	67781440	882	Bentley et al. (2008)	Illumina DNA sequencing	
147	chr1	69769049	69774145	5096	4	chr1	69769018	69774261	5243	HiConf	Variation_22673	chr1	69768614	69775243	6629	Korbel et al. (2007)	Paired End Mapping	
148	chr1	71259769	71262226	2457	5	chr1	71259108	71262293	3185	HiConf								
149	chr1	72132662	72133642	980	29	chr1	72132631	72133660	1029	HiConf								y
150	chr1	72527994	72536873	8879	33	chr1	72527963	72536881	8918	HiConf	Variation_38997	chr1	72528280	72536662	8382	Wheeler et al. (2008)	Sequencing	y
151	chr1	72537650	72583658	46008	48	chr1	72537616	72584607	46991	HiConf	Variation_6832	chr1	72539082	72584229	45147	de Smith et al. (2007)	Agilent 185k CGH Arrays/Agilent Custom CGH Arrays	
100025	chr1	73451179	73483023	31844	9	chr1	73451148	73483684	32536	HiConf	Variation_30389	chr1	73452929	73484611	31682	Perry et al. (2008)	Agilent Custom CGH Arrays	
100026	chr1	75843163	75902899	59736	3	chr1	75843132	75904235	61103	HiConf	Variation_31628	chr1	75859509	75891203	31694	Perry et al. (2008)	Agilent Custom CGH Arrays	
100027	chr1	76941911	76964185	22274	4	chr1	76936861	76964742	27881	HiConf								
155	chr1	77245812	77257670	11858	6	chr1	77245781	77257884	12103	HiConf								
156	chr1	77546868	77553973	7105	4	chr1	77546837	77554089	7252	HiConf	Variation_38184	chr1	77547460	77552420	4960	McCarroll et al. (2008)	Affymetrix Human SNP Array 6.0	
157	chr1	78123647	78123809	162	4	chr1	78123616	78123812	196	single								
158	chr1	78421175	78424087	2912	29	chr1	78421144	78430258	9114	HiConf	Variation_34835	chr1	78421079	78433560	12481	Kidd et al. (2008)	Paired End Mapping	y
159	chr1	78606983	78607963	980	24	chr1	78606952	78607981	1029	HiConf								
160	chr1	79166073	79169111	3038	10	chr1	79166042	79169178	3136	HiConf								

locus_id	chrom	start	end	length	Yoruba w/ event	putative chr	putative start	putative end	putative len	putative type	variation_id	DGV_chr	DGV_start	DGV_end	DGV_len	Reference	Method/platform	complex
100028	chr1	79218895	79252656	33761	8	chr1	79218864	79253336	34472	single								
162	chr1	79670381	79670691	310	13	chr1	79670350	79670693	343	single								
163	chr1	79889166	79889852	686	6	chr1	79889135	79889870	735	HiConf	Variation_8918	chr1	79841670	81616474	1774804	Pinto et al. (2007)	Affymetrix 500K SNP Mapping Array	
164	chr1	79991233	79995479	4246	28	chr1	79991202	79995514	4312	HiConf	Variation_23370	chr1	79994370	79995618	1248	Levy et al. (2007)	Sequencing	
100029						chr1	80248771	80316881	68110	single								
165	chr1	81189870	81201876	12006	12	chr1	81176267	81202384	26117	HiConf	Variation_34836	chr1	81154388	81195935	41547	Kidd et al. (2008)	Paired End Mapping	
166	chr1	81209030	81217017	7987	14	chr1	81208999	81217182	8183	HiConf	Variation_8918	chr1	79841670	81616474	1774804	Pinto et al. (2007)	Affymetrix 500K SNP Mapping Array	
100030	chr1	83357876	83723220	365344	4	chr1	83357845	83730686	372841	HiConf	Variation_2313	chr1	83375945	83716816	340871	Redon et al. (2006)	Affymetrix 500K EA SNP Mapping Array	
168	chr1	84161182	84161380	198	18	chr1	84161151	84161396	245	single								
169	chr1	84484631	84488208	3577	78	chr1	84484600	84488275	3675	HiConf	Variation_43783	chr1	84484595	84488456	3861	Bentley et al. (2008)	Illumina DNA sequencing	
170	chr1	84506142	84510013	3871	5	chr1	84506111	84510080	3969	single								
171	chr1	84668626	84671566	2940	3	chr1	84668595	84671633	3038	HiConf	Variation_38185	chr1	84668621	84670569	1948	McCarroll et al. (2008)	Affymetrix Human SNP Array 6.0	y
172	chr1	84742714	84744723	2009	30	chr1	84742683	84744741	2058	single								
173	chr1	85753094	85777998	24904	1	chr1	85753063	85778494	25431	single								
174	chr1	86173563	86177042	3479	13	chr1	86173532	86177109	3577	HiConf	Variation_31631	chr1	86173636	86176916	3280	Perry et al. (2008)	Agilent Custom CGH Arrays	
175						chr1	86819401	86826555	7154	single								
176	chr1	86933357	86934778	1421	4	chr1	86933326	86934796	1470	single								
177	chr1	86935072	86951879	16807	2	chr1	86935041	86952191	17150	single								
178	chr1	87296300	87296888	588	9	chr1	87296269	87296906	637	single								
179	chr1	88109553	88110925	1372	3	chr1	88109522	88110943	1421	single								
180	chr1	88866848	88867877	1029	13	chr1	88866817	88867895	1078	HiConf								
181	chr1	88905466	88926971	21505	5	chr1	88905429	88927381	21952	single								
182	chr1	89249558	89250812	1254	3	chr1	89249556	89250830	1274	HiConf	Variation_30391	chr1	89249037	89251025	1988	Perry et al. (2008)	Agilent Custom CGH Arrays	
183	chr1	89357632	89367873	10241	3	chr1	89357601	89368087	10486	HiConf								
184	chr1	89941810	89946612	4802	6	chr1	89941779	89946679	4900	HiConf								
100031						chr1	90025202	90096227	71025	HiConf								
185	chr1	91108108	91115703	7595	4	chr1	91108077	91115868	7791	single								
186	chr1	91625548	91625611	63	31	chr1	91625517	91625615	98	single								
187	chr1	92004680	92005886	1206	55	chr1	92004679	92005904	1225	HiConf	Variation_39002	chr1	92004649	92005921	1272	Wheeler et al. (2008)	Sequencing	
188	chr1	92304884	92313459	8575	13	chr1	92304853	92313624	8771	HiConf	Variation_8323	chr1	92129369	92352263	222894	Pinto et al. (2007)	Affymetrix 500K SNP Mapping Array	
189	chr1	92352207	92357020	4813	5	chr1	92351844	92357136	5292	HiConf	Variation_38187	chr1	92351832	92357103	5271	McCarroll et al. (2008)	Affymetrix Human SNP Array 6.0	
190	chr1	93985633	93986033	400	19	chr1	93985602	93986043	441	single								
191	chr1	94061066	94063053	1987	3	chr1	94061062	94063071	2009	HiConf	Variation_6838	chr1	94060954	94063617	2663	de Smith et al. (2007)	Agilent 185k CGH Arrays/Agilent Custom CGH Arrays	
100032	chr1	94890933	94927487	36554	8	chr1	94890902	94928240	37338	HiConf	Variation_2314	chr1	94892378	94931191	38813	Redon et al. (2006)	Affymetrix 500K EA SNP Mapping Array	
193	chr1	95664324	95667754	3430	7	chr1	95664293	95667821	3528	HiConf								
194	chr1	96725419	96727820	2401	1	chr1	96725388	96727838	2450	single								
195	chr1	97628342	97634859	6517	7	chr1	97628311	97634975	6664	single								
196	chr1	97810573	97814983	4410	1	chr1	97810542	97815050	4508	single	Variation_4236	chr1	97680297	97873732	193435	Wong et al. (2007)	BAC Array CGH	
197	chr1	99602846	99606668	3822	1	chr1	99602815	99606735	3920	single								
198	chr1	99663557	99664096	539	33	chr1	99663526	99664114	588	HiConf								
199	chr1	100892624	100897230	4606	33	chr1	100892593	100897297	4704	HiConf								
100033	chr1	101461367	101468443	7076	1	chr1	101461312	101496812	35500	single								
201	chr1	101675448	101679025	3577	2	chr1	101675417	101679092	3675	single								
202	chr1	101751104	101759091	7987	3	chr1	101751073	101759256	8183	HiConf								
203	chr1	101869341	101871448	2107	6	chr1	101869310	101871466	2156	HiConf								
204	chr1	102094104	102099053	4949	4	chr1	102094073	102099169	5096	HiConf	Variation_38139	chr1	102096209	102098748	2539	McCarroll et al. (2008)	Affymetrix Human SNP Array 6.0	
205	chr1	102174513	102184264	9751	4	chr1	102174482	102184478	9996	HiConf	Variation_2315	chr1	102144927	102189681	44754	Redon et al. (2006)	Affymetrix 500K EA SNP Mapping Array	
206	chr1	102756339	102757319	980	26	chr1	102756308	102757337	1029	HiConf								
207	chr1	103905340	104102075	196735	23	chr1	103905309	104106062	200753	HiConf	Variation_30393	chr1	103905304	104102680	197376	Perry et al. (2008)	Agilent Custom CGH Arrays	
208	chr1	104108347	104110846	2499	5	chr1	104108316	104110913	2597	HiConf	Variation_0012	chr1	103956056	104113271	157215	Iafrate et al. (2004)	BAC Array CGH	
209	chr1	104213942	104221337	7395	5	chr1	104213911	104221506	7595	HiConf	Variation_31638	chr1	104219709	104221299	1590	Perry et al. (2008)	Agilent Custom CGH Arrays	
210	chr1	104244763	104245213	450	30	chr1	104244732	104245222	490	HiConf	Variation_44426	chr1	104244722	104245250	528	Bentley et al. (2008)	Illumina DNA sequencing	
211	chr1	104471094	104471496	402	38	chr1	104471063	104471504	441	single	Variation_34326	chr1	104471447	104471695	248	Levy et al. (2007)	Sequencing	
212	chr1	104508285	104509314	1029	16	chr1	104508254	104509332	1078	HiConf								
213	chr1	104840456	104844229	3773	2	chr1	104840425	104844296	3871	single								y
214	chr1	105309876	105310856	980	31	chr1	105309845	105310874	1029	single								
215	chr1	105426839	105431998	5159	6	chr1	105426808	105432100	5292	HiConf								y
216	chr1	105469322	105469910	588	63	chr1	105469291	105469928	637	single								y
217	chr1	105816051	105825021	8970	53	chr1	105815966	105825031	9065	HiConf	Variation_43645	chr1	105817308	105825002	7694	Wang et al. (2008)	Illumina DNA sequencing	
100035	chr1	105960841	106018563	57722	6	chr1	105960810	106036809	75999	HiConf	Variation_38142	chr1	105969819	106016548	46729	McCarroll et al. (2008)	Affymetrix Human SNP Array 6.0	
219	chr1	106108968	106119096	10128	23	chr1	106108937	106119227	10290	HiConf	Variation_38143	chr1	106110789	106116611	5822	McCarroll et al. (2008)	Affymetrix Human SNP Array 6.0	
220	chr1	106759786	106763265	3479	1	chr1	106759755	106763332	3577	single								
221	chr1	106779925	106783110	3185	21	chr1	106779894	106783177	3283	HiConf								
222	chr1	106898603	106907913	9310	12	chr1	106898572	106908078	9506	HiConf								
223	chr1	107022083	107026297	4214	8	chr1	107022052	107026364	4312	HiConf	Variation_37865	chr1	107023241	107025005	1764	McCarroll et al. (2008)	Affymetrix Human SNP Array 6.0	
100036	chr1	108112781	108126053	13272	1	chr1	108102796	108126537	23741	single	Variation_4240	chr1	108013196	108216400	203204	Wong et al. (2007)	BAC Array CGH	
225	chr1	108457832	108459253	1421	2	chr1	108457801	108459271	1470	single	Variation_3305	chr1	108420397	108898201	477804	Redon et al. (2006)	BAC Array CGH	
226	chr1	108534419	108539285	4866	23	chr1	108534388	108539288	4900	HiConf	Variation_43558	chr1	108534810	108538892	4082	Wang et al. (2008)	Illumina DNA sequencing	
227						chr1	108572020	108590885	18865	single								
228	chr1	108605224	108654126	48902	4	chr1	108605193	108655124	49931	HiConf	Variation_30397	chr1	108604493	108654504	50011	Perry et al. (2008)	Agilent Custom CGH Arrays	
229	chr1	108728214	108747520	19306	8	chr1	108728183	108747930	19747	HiConf	Variation_30398	chr1	108727420	108778174	50754	Perry et al. (2008)	Agilent Custom CGH Arrays	
230	chr1	108748078	108788974	40896	9	chr1	108748077	108789825	41748	HiConf	Variation_38853	chr1	108729983	108787720	57737	McCarroll et al. (2008)	Affymetrix Human SNP Array 6.0	
231	chr1	108994970	109000164	5194	15	chr1	108994939	109000280	5341	single								
232	chr1	109298231	109305434	7203	11	chr1	109298200	109305599	7399	HiConf	Variation_37882	chr1	109298377	109299668	1291	McCarroll et al. (2008)	Affymetrix Human SNP Array 6.0	
233	chr1	109375063	109376337	1274	28	chr1	109375032	109376355	1323	single	Variation_43417	chr1	109374706	109376814	2108	Wang et al. (2008)	Illumina DNA sequencing	
234	chr1	109988298	109992561	4263	43	chr1	109988267	109992628	4361	HiConf	Variation_22533	chr1	109988334	109993086	4752	Korbel et al. (2007)	Paired End Mapping	
100038	chr1	110002410	110059130	56720	26	chr1	110002379	110060322	57943	HiConf	Variation_30400	chr1	110019656	110058541	38885	Perry et al. (2008)	Agilent Custom CGH Arrays	
236	chr1	110527151	110532051	4900	6	chr1	110527120	110532167	5047	single								
237	chr1	110639655	110641027	1372	7	chr1	110639624	110641045	1421	single								
238	chr1	111176352	111190464	14112	34	chr1	111176321	111190727	14406	HiConf	Variation_9890	chr1	111176350	111189246	12896	Wang et al. (2007)	Illumina HumanHap550 BeadChip	
239	chr1	111972406	111985178	12772	5	chr1	111972375	111985360	12985	single								
240	chr1	113021692	113025759	4067	8	chr1	113021661	113025826	4165	HiConf	Variation_3306	chr1	112958658	116542895	3584237	Redon et al. (2006)	BAC Array CGH	
241	chr1	113331323	113333675	2352	25	chr1	113331292	113333693	2401	HiConf	Variation_2322	chr1	113048153	116500791	3452638	Redon et al. (2006)	Affymetrix 500K EA SNP Mapping Array	
242	chr1	113466122	113467347	1225	2	chr1	113466091	113467365	1274	single	Variation_2322	chr1	113048153	116500791	3452638	Redon et al. (2006)	Affymetrix 500K EA SNP Mapping Array	
243	chr1	113691130	113703037	11907	2	chr1	113691099	113703251	12152	single	Variation_2322	chr1	113048153	116500791	3452638	Redon et al. (2006)	Affymetrix 500K EA SNP Mapping Array	
244	chr1	114809310	114857232	47922	4	chr1	114809279	114858181	48902	HiConf	Variation_44434	chr1	114845056	114845351	295	Bentley et al. (2008)	Illumina DNA sequencing	

locus_id	chrom	start	end	length	Yoruba w/ event	putative chr	putative start	putative end	putative len	putative type	variation_id	DGV_chr	DGV_start	DGV_end	DGV_len	Reference	Method/platform	complex
245	chr1	116031223	116034261	3038	60	chr1	116031192	116034328	3136	HiConf	Variation_2322	chr1	113048153	116500791	3452638	Redon et al. (2006)	Affymetrix 500K EA SNP Mapping Array	
100040	chr1	116915395	117004096	88701	24	chr1	116914956	117005729	90773	HiConf	Variation_30403	chr1	116961521	117004368	42847	Perry et al. (2008)	Agilent Custom CGH Arrays	
247	chr1	117682768	117682876	108	18	chr1	117682737	117682884	147	single								
248	chr1	117772732	117778122	5390	16	chr1	117772701	117778238	5537	single								y
249	chr1	117778416	117784149	5733	2	chr1	117778385	117784265	5880	single								
250	chr1	119129493	119132335	2842	1	chr1	119129462	119132402	2940	single	Variation_8326	chr1	119030780	119183750	152970	Pinto et al. (2007)	Affymetrix 500K SNP Mapping Array	
251	chr1	119284046	119285117	1071	7	chr1	119284008	119285135	1127	HiConf								
252	chr1	119904036	119926870	22834	5	chr1	119904005	119927329	23324	HiConf	Variation_9370	chr1	119892957	119928513	35556	Wang et al. (2007)	Illumina HumanHap550 BeadChip	
253	chr1	119927531	119943383	15852	2	chr1	119927525	119943695	16170	single	Variation_1015	chr1	119924945	119929020	4075	Conrad et al. (2005)	Mendelian inconsistencies	
100041	chr1	120314803	120414030	99227	4	chr1	120314772	120861612	546840	HiConf	Variation_30405	chr1	120332743	120432023	99280	Perry et al. (2008)	Agilent Custom CGH Arrays	
257	chr1	142375289	142375739	450	15	chr1	142375258	142375748	490	single								
258	chr1	142500925	142545956	45031	7	chr1	142500894	142546856	45962	HiConf								
259	chr1	143085103	143085691	588	58	chr1	143085072	143085709	637	single	Variation_4248	chr1	143026080	143111804	85724	Wong et al. (2007)	BAC Array CGH	
260	chr1	143388217	143407964	19747	10	chr1	143388186	143408374	20188	single	Variation_31651	chr1	143327178	143527303	200125	Perry et al. (2008)	Agilent Custom CGH Arrays	
261	chr1	143789369	143811997	22628	5	chr1	143789300	143812526	23226	HiConf	Variation_34696	chr1	143782866	143815877	33011	Kidd et al. (2008)	Paired End Mapping	
262	chr1	143955049	143978961	23912	2	chr1	143955018	143979420	24402	HiConf	Variation_4251	chr1	143921702	144020182	98480	Wong et al. (2007)	BAC Array CGH	
263	chr1	143979721	144087463	107742	6	chr1	143979714	144089572	109858	HiConf	Variation_37561	chr1	144005888	144097032	91144	Cooper et al. (2008)	Illumina Human 1M BeadChip	
264	chr1	144092690	144093458	768	28	chr1	144092659	144093492	833	single	Variation_37561	chr1	144005888	144097032	91144	Cooper et al. (2008)	Illumina Human 1M BeadChip	
100043	chr1	144651882	144805457	153575	7	chr1	144651749	144805756	154007	HiConf	Variation_31654	chr1	144692908	144701906	8998	Perry et al. (2008)	Agilent Custom CGH Arrays	
267	chr1	145049660	145054511	4851	9	chr1	145049629	145054627	4998	single	Variation_3312	chr1	144594476	145164166	569690	Redon et al. (2006)	BAC Array CGH	
268	chr1	145952319	145954499	2180	1	chr1	145950200	145963283	13083	HiConf	Variation_34420	chr1	145708347	147521567	1813220	Zogopoulos et al. (2007)	Affymetrix 500K and 100K SNP Mapping Arrays	
269	chr1	145963522	146001163	37641	4	chr1	145963479	146001944	38465	HiConf	Variation_34420	chr1	145708347	147521567	1813220	Zogopoulos et al. (2007)	Affymetrix 500K and 100K SNP Mapping Arrays	
270	chr1	146002171	146022996	20825	4	chr1	146002140	146023406	21266	HiConf	Variation_34420	chr1	145708347	147521567	1813220	Zogopoulos et al. (2007)	Affymetrix 500K and 100K SNP Mapping Arrays	
271	chr1	146039705	146074152	34447	10	chr1	146039674	146074856	35182	single	Variation_34420	chr1	145708347	147521567	1813220	Zogopoulos et al. (2007)	Affymetrix 500K and 100K SNP Mapping Arrays	
272	chr1	146080620	146099724	19104	6	chr1	146080589	146099748	19159	single	Variation_39011	chr1	146097096	146106370	9274	Wheeler et al. (2008)	Sequencing	
273	chr1	146445866	146466502	20636	10	chr1	146445835	146466562	20727	single	Variation_39012	chr1	146442922	146462600	19678	Wheeler et al. (2008)	Sequencing	
274	chr1	146562486	146596345	33859	3	chr1	146562455	146597049	34594	single	Variation_2051	chr1	146578326	146719169	140843	Locke et al. (2006)	BAC Array CGH	
275	chr1	146866059	146888973	22914	9	chr1	146865961	146889432	23471	single	Variation_30428	chr1	146862821	146905437	42616	Perry et al. (2008)	Agilent Custom CGH Arrays	
276	chr1	147032396	147044499	12103	10	chr1	147032365	147044762	12397	single	Variation_30430	chr1	147024865	147067757	42892	Perry et al. (2008)	Agilent Custom CGH Arrays	
277	chr1	147045203	147049546	4343	9	chr1	147045203	147049613	4410	single	Variation_30430	chr1	147024865	147067757	42892	Perry et al. (2008)	Agilent Custom CGH Arrays	
278	chr1	147131915	147137158	5243	11	chr1	147131884	147137274	5390	single	Variation_37600	chr1	147017157	147217833	200676	Cooper et al. (2008)	Illumina Human 1M BeadChip	
279	chr1	147157395	147210714	53319	8	chr1	147157364	147211803	54439	single	Variation_30431	chr1	147162330	147169636	7306	Perry et al. (2008)	Agilent Custom CGH Arrays	
280	chr1	147302533	147311255	8722	13	chr1	147302502	147311420	8918	single	Variation_9372	chr1	147305744	147311729	5985	Wang et al. (2007)	Illumina HumanHap550 BeadChip	
281	chr1	147776363	147784007	7644	8	chr1	147776332	147784172	7840	HiConf	Variation_30433	chr1	147302889	147858378	555489	Perry et al. (2008)	Agilent Custom CGH Arrays	
282	chr1	147784613	147859418	74805	16	chr1	147784613	147860955	76342	HiConf	Variation_30433	chr1	147302889	147858378	555489	Perry et al. (2008)	Agilent Custom CGH Arrays	
283	chr1	148748557	148749454	897	1	chr1	148746042	148749521	3479	single								
284	chr1	149557954	149560159	2205	1	chr1	149557923	149560177	2254	single	Variation_8332	chr1	149507166	149678259	171093	Pinto et al. (2007)	Affymetrix 500K SNP Mapping Array	
285	chr1	150346756	150351816	5060	17	chr1	150346725	150351870	5145	HiConf	Variation_1557	chr1	150346613	150350663	4050	McCarroll et al. (2005)	Mendelian inconsistencies	
286	chr1	150452596	150459992	7396	16	chr1	150452565	150460062	7497	HiConf	Variation_30437	chr1	150452906	150458231	5325	Perry et al. (2008)	Agilent Custom CGH Arrays	
287	chr1	150542511	150547072	4561	5	chr1	150542480	150547086	4606	HiConf	Variation_23973	chr1	150545285	150546257	972	Levy et al. (2007)	Sequencing	
288	chr1	150761982	150763795	1813	7	chr1	150761951	150763813	1862	single	Variation_8333	chr1	150720293	150927551	207258	Pinto et al. (2007)	Affymetrix 500K SNP Mapping Array	
100046	chr1	150819667	150853776	34109	60	chr1	150798652	150858163	59511	HiConf	Variation_22907	chr1	150821592	150854502	32910	Korbel et al. (2007)	Paired End Mapping	
100047	chr1	150930640	151013940	83300	1	chr1	150930609	151015624	85015	single	Variation_8334	chr1	150938537	151023430	84893	Pinto et al. (2007)	Affymetrix 500K SNP Mapping Array	
291	chr1	151026190	151041037	14847	25	chr1	151026159	151041349	15190	HiConf	Variation_34716	chr1	151025776	151041038	15262	Kidd et al. (2008)	Paired End Mapping	y
292	chr1	151479440	151493526	14086	25	chr1	151479409	151493717	14308	single								
293	chr1	151853947	151855074	1127	26	chr1	151853916	151855092	1176	single								
294	chr1	151941275	151959361	18086	5	chr1	151940303	151959854	19551	HiConf	Variation_37780	chr1	151940298	151959872	19574	McCarroll et al. (2008)	Affymetrix Human SNP Array 6.0	
295	chr1	152192678	152196570	3892	1	chr1	152190840	152197798	6958	single								
296	chr1	152422739	152427826	5087	18	chr1	152422708	152427902	5194	HiConf								y
100048	chr1	153257832	153261512	3680	3	chr1	153242601	153316419	73818	single								
100049	chr1	153444505	153481819	37314	11	chr1	153438184	153482701	44517	HiConf	Variation_30443	chr1	153451033	153470618	19585	Perry et al. (2008)	Agilent Custom CGH Arrays	
298	chr1	153814094	153819995	5901	3	chr1	153814063	153820090	6027	single	Variation_6789	chr1	153489907	154184585	694678	de Smith et al. (2007)	Agilent 185k CGH Arrays/Agilent Custom CGH Arrays	
299	chr1	153925324	153931057	5733	27	chr1	153925293	153931173	5880	HiConf	Variation_38770	chr1	153927851	153929981	2130	McCarroll et al. (2008)	Affymetrix Human SNP Array 6.0	
300	chr1	155440747	155441825	1078	6	chr1	155440716	155441843	1127	HiConf								
301	chr1	155852102	155858766	6664	11	chr1	155852071	155858882	6811	HiConf								
302	chr1	156416190	156418632	2442	1	chr1	156416159	156436543	20384	single								
303	chr1	156782824	156788394	5570	9	chr1	156782777	156788510	5733	HiConf	Variation_38148	chr1	156783620	156787041	3421	McCarroll et al. (2008)	Affymetrix Human SNP Array 6.0	
304	chr1	156993753	156994488	735	10	chr1	156993722	156994506	784	single								y
305	chr1	157133699	157136646	2947	65	chr1	157133666	157136655	2989	HiConf	Variation_43486	chr1	157133963	157136708	2745	Wang et al. (2008)	Illumina DNA sequencing	
306	chr1	157228414	157232677	4263	15	chr1	157228383	157232744	4361	HiConf	Variation_43744	chr1	157227938	157232827	4889	Bentley et al. (2008)	Illumina DNA sequencing	
307	chr1	157279717	157285891	6174	3	chr1	157279686	157286007	6321	single								
308	chr1	157387615	157390801	3186	9	chr1	157387584	157393513	5929	HiConf								
309	chr1	157877027	157885006	7979	24	chr1	157876996	157885179	8183	single								y
310	chr1	157914904	157916244	1340	48	chr1	157914873	157916245	1372	HiConf	Variation_39663	chr1	157915331	157916281	950	Wheeler et al. (2008)	Sequencing	
311	chr1	158961005	158961167	162	23	chr1	158960974	158961170	196	HiConf								y
312	chr1	159654551	159711712	57161	25	chr1	159654520	159712046	57526	HiConf	Variation_34724	chr1	159658059	159690724	32665	Kidd et al. (2008)	Paired End Mapping	
313	chr1	159882450	159912516	30066	17	chr1	159882419	159913093	30674	HiConf	Variation_1339	chr1	159887552	159901749	14197	Conrad et al. (2005)	Mendelian inconsistencies	
314	chr1	160848534	160850396	1862	5	chr1	160848503	160850414	1911	single								
100051						chr1	161632846	161669033	36187	single								
100052	chr1	163396069	163438011	41942	8	chr1	163396038	163438888	42850	HiConf								
316	chr1	163688476	163690778	2302	5	chr1	163688445	163690797	2352	HiConf	Variation_37853	chr1	163688498	163690794	2296	McCarroll et al. (2008)	Affymetrix Human SNP Array 6.0	
317	chr1	164451326	164458609	7283	18	chr1	164451326	164458774	7448	single	Variation_37782	chr1	164451105	164460994	9889	McCarroll et al. (2008)	Affymetrix Human SNP Array 6.0	
318	chr1	164818171	164819053	882	28	chr1	164818140	164819071	931	HiConf	Variation_44465	chr1	164818209	164819151	942	Bentley et al. (2008)	Illumina DNA sequencing	
319	chr1	164946061	164951990	5929	4	chr1	164946030	164952106	6076	HiConf								
320	chr1	165266423	165270000	3577	5	chr1	165266392	165270067	3675	single	Variation_8338	chr1	165220490	165446966	226476	Pinto et al. (2007)	Affymetrix 500K SNP Mapping Array	
321	chr1	165592910	165600093	7183	2	chr1	165592879	165600768	7889	single								
100053	chr1	166408809	166468981	60172	6	chr1	166408778	166470224	61446	single	Variation_4261	chr1	166445800	166619686	173886	Wong et al. (2007)	BAC Array CGH	
322	chr1	166555956	166556740	784	3	chr1	166555925	166556758	833	single	Variation_4261	chr1	166445800	166619686	173886	Wong et al. (2007)	BAC Array CGH	
323	chr1	166645038	166646410	1372	36	chr1	166645007	166646428	1421	single								
324	chr1	167493878	167509319	15441	15	chr1	167486827	167521960	35133	HiConf	Variation_6796	chr1	167493797	167507911	14114	de Smith et al. (2007)	Agilent 185k CGH Arrays/Agilent Custom CGH Arrays	
325	chr1	168149926	168191331	41405	1	chr1	168149895	168192182	42287	single								
326	chr1	168633899	168655851	21952	5	chr1	168633868	168656310	22442	HiConf	Variation_38151	chr1	168634270	168653829	19559	McCarroll et al. (2008)	Affymetrix Human SNP Array 6.0	
327	chr1	169618211	169624630	6419	7	chr1	169618180	169624746	6566	single								
328	chr1	170456748	170460766	4018	1	chr1	170456717	170460833	4116	single								
329	chr1	170949345	170950913	1568	1	chr1	170949314	170950931	1617	single								
330	chr1	171025981	171028382	2401	9	chr1	171025950	171028449	2499	HiConf								

locus_id	chrom	start	end	length	Yoruba w/ event	putative chr	putative start	putative end	putative len	putative type	variation_id	DGV_chr	DGV_start	DGV_end	DGV_len	Reference	Method/platform	complex
331	chr1	172196542	172199041	2499	2	chr1	172196511	172199108	2597	single	Variation_3320	chr1	172077265	172222566	145301	Redon et al. (2006)	BAC Array CGH	
332	chr1	172230744	172234370	3626	1	chr1	172230713	172234437	3724	single								
333	chr1	172879063	172880484	1421	1	chr1	172879032	172880502	1470	single								
334	chr1	173063210	173068355	5145	17	chr1	173060773	173068466	7693	HiConf	Variation_38057	chr1	173063179	173068463	5284	McCarroll et al. (2008)	Affymetrix Human SNP Array 6.0	
335	chr1	173289095	173292265	3170	5	chr1	173289064	173292347	3283	HiConf								
336	chr1	173461477	173466224	4747	1	chr1	173461446	173466542	5096	single								
337	chr1	173678253	173678703	450	10	chr1	173678222	173678712	490	HiConf								
338	chr1	173717649	173719217	1568	3	chr1	173717618	173719235	1617	single	Variation_34421	chr1	173695580	174027375	331795	Zogopoulos et al. (2007)	Affymetrix 500K and 100K SNP Mapping Arrays	
339	chr1	173886601	173886911	310	13	chr1	173886570	173886913	343	HiConf	Variation_34421	chr1	173695580	174027375	331795	Zogopoulos et al. (2007)	Affymetrix 500K and 100K SNP Mapping Arrays	
340	chr1	174075692	174080690	4998	14	chr1	174075661	174080806	5145	single	Variation_6798	chr1	173713206	174176624	463418	de Smith et al. (2007)	Agilent 185k CGH Arrays/Agilent Custom CGH Arrays	
341	chr1	174270712	174286784	16072	7	chr1	174270681	174287096	16415	single								
342	chr1	174857389	174878935	21546	2	chr1	174857358	174879359	22001	single	Variation_9901	chr1	174857914	174871187	13273	Wang et al. (2007)	Illumina HumanHap550 BeadChip	
343	chr1	175601013	175607775	6762	8	chr1	175600982	175607891	6909	single								
344	chr1	176486492	176489212	2720	2	chr1	176486461	176489891	3430	HiConf								
345	chr1	176762019	176762082	63	66	chr1	176761988	176762086	98	HiConf	Variation_43114	chr1	176761860	176762603	743	Wang et al. (2008)	Illumina DNA sequencing	
346	chr1	176925091	176942584	17493	3	chr1	176925060	176942945	17885	HiConf	Variation_2334	chr1	176924205	176940697	16492	Redon et al. (2006)	Affymetrix 500K EA SNP Mapping Array	
347	chr1	177158870	177163101	4231	3	chr1	177158839	177163151	4312	single								y
348	chr1	177596538	177599870	3332	36	chr1	177596507	177599937	3430	HiConf	Variation_38015	chr1	177597151	177599938	2787	McCarroll et al. (2008)	Affymetrix Human SNP Array 6.0	
349	chr1	177723105	177724134	1029	24	chr1	177723074	177724152	1078	single								
100055	chr1	177815702	177845262	29560	3	chr1	177815635	177845868	30233	single	Variation_34739	chr1	177836523	177850224	13701	Kidd et al. (2008)	Paired End Mapping	
350	chr1	177873976	177874613	637	72	chr1	177873945	177874631	686	HiConf	Variation_44473	chr1	177873912	177874684	772	Bentley et al. (2008)	Illumina DNA sequencing	
100056						chr1	177984857	178011170	26313	HiConf								
352	chr1	178192182	178194681	2499	3	chr1	178192151	178194748	2597	HiConf								
353	chr1	179475835	179478180	2345	2	chr1	179475804	179479185	3381	single								
354	chr1	179661888	179663309	1421	21	chr1	179661857	179663327	1470	HiConf								y
355	chr1	179970392	179970792	400	25	chr1	179970361	179970802	441	single								
356	chr1	181135024	181136102	1078	2	chr1	181134993	181136120	1127	single								
357	chr1	181366469	181400751	34282	28	chr1	181366420	181401455	35035	HiConf								
358	chr1	183228353	183236830	8477	2	chr1	183228322	183236995	8673	single								
359	chr1	183276324	183278333	2009	18	chr1	183276293	183278351	2058	HiConf								
360	chr1	184050818	184051994	1176	47	chr1	184050787	184052012	1225	HiConf	Variation_43748	chr1	184050339	184051921	1582	Bentley et al. (2008)	Illumina DNA sequencing	
361	chr1	184181354	184183314	1960	10	chr1	184181323	184183332	2009	single								
362	chr1	184543954	184544354	400	34	chr1	184543923	184544364	441	single								
363	chr1	185562664	185567417	4753	3	chr1	185562633	185567484	4851	HiConf	Variation_38154	chr1	185564993	185567314	2321	McCarroll et al. (2008)	Affymetrix Human SNP Array 6.0	
364	chr1	185666495	185671542	5047	7	chr1	185666464	185671658	5194	HiConf	Variation_8926	chr1	185336978	186256217	919239	Pinto et al. (2007)	Affymetrix 500K SNP Mapping Array	
365	chr1	185731665	185733233	1568	31	chr1	185731634	185733251	1617	HiConf	Variation_8926	chr1	185336978	186256217	919239	Pinto et al. (2007)	Affymetrix 500K SNP Mapping Array	
366	chr1	185839517	185848910	9393	28	chr1	185833407	185849381	15974	HiConf	Variation_9904	chr1	185839646	185847926	8280	Wang et al. (2007)	Illumina HumanHap550 BeadChip	
367	chr1	185860290	185863573	3283	11	chr1	185860259	185863640	3381	HiConf	Variation_43749	chr1	185860265	185863517	3252	Bentley et al. (2008)	Illumina DNA sequencing	
368	chr1	185982808	185989366	6558	9	chr1	185982808	185989374	6566	HiConf	Variation_39023	chr1	185982727	185989155	6428	Wheeler et al. (2008)	Sequencing	
369	chr1	186806360	186806742	382	68	chr1	186806351	186806743	392	HiConf	Variation_44479	chr1	186806082	186806850	768	Bentley et al. (2008)	Illumina DNA sequencing	
100058	chr1	187057927	187097175	39248	3	chr1	187020432	187109637	89205	HiConf								
370	chr1	187225724	187233417	7693	1	chr1	187225693	187233582	7889	single								
371	chr1	187353516	187359935	6419	6	chr1	187353485	187360051	6566	HiConf	Variation_38156	chr1	187353623	187359115	5492	McCarroll et al. (2008)	Affymetrix Human SNP Array 6.0	
372	chr1	187510592	187513452	2860	13	chr1	187510236	187513519	3283	HiConf	Variation_31671	chr1	187510791	187513215	2424	Perry et al. (2008)	Agilent Custom CGH Arrays	
373	chr1	187683335	187702984	19649	3	chr1	187683304	187703394	20090	single	Variation_38157	chr1	187593758	187719766	126008	McCarroll et al. (2008)	Affymetrix Human SNP Array 6.0	
374	chr1	188038389	188044132	5743	6	chr1	188038358	188052715	14357	HiConf	Variation_9907	chr1	188037441	188043864	6423	Wang et al. (2007)	Illumina HumanHap550 BeadChip	
375	chr1	188120023	188120473	450	14	chr1	188119992	188120482	490	HiConf								
376	chr1	189140252	189142457	2205	4	chr1	189140221	189142475	2254	HiConf								
377	chr1	189346738	189347048	310	4	chr1	189346707	189347050	343	single								
378	chr1	189983861	189993688	9827	6	chr1	189983854	189993703	9849	HiConf	Variation_39025	chr1	189983668	189993306	9638	Wheeler et al. (2008)	Sequencing	
100060	chr1	190092224	190135589	43365	9	chr1	190092193	190136465	44272	HiConf	Variation_38160	chr1	190095692	190133873	38181	McCarroll et al. (2008)	Affymetrix Human SNP Array 6.0	
380	chr1	190183805	190184932	1127	45	chr1	190183774	190184950	1176	HiConf	Variation_43751	chr1	190183066	190185283	2217	Bentley et al. (2008)	Illumina DNA sequencing	
381	chr1	190941933	190949871	7938	1	chr1	190941902	190950036	8134	single								
382	chr1	191127447	191128476	1029	69	chr1	191127416	191128494	1078	HiConf								
100061	chr1	191923942	191977891	53949	3	chr1	191923911	191978987	55076	HiConf	Variation_38161	chr1	191927641	191976999	49358	McCarroll et al. (2008)	Affymetrix Human SNP Array 6.0	
384	chr1	192454367	192455739	1372	8	chr1	192454336	192455757	1421	HiConf								
100062						chr1	192576812	192624856	48044	single								
386	chr1	192717693	192723279	5586	47	chr1	192716731	192723395	6664	HiConf								
387	chr1	193026148	193026458	310	3	chr1	193026117	193026460	343	single								
388	chr1	193069350	193074511	5161	4	chr1	193069286	193074627	5341	HiConf	Variation_43752	chr1	193069200	193074564	5364	Bentley et al. (2008)	Illumina DNA sequencing	
389	chr1	193202793	193212740	9947	3	chr1	193202762	193212954	10192	HiConf	Variation_38162	chr1	193204702	193213877	9175	McCarroll et al. (2008)	Affymetrix Human SNP Array 6.0	
390	chr1	193309956	193310118	162	34	chr1	193309925	193310121	196	single								
391	chr1	193625173	193631347	6174	4	chr1	193625142	193631463	6321	HiConf	Variation_3323	chr1	193522550	193846835	324285	Redon et al. (2006)	BAC Array CGH	
100063	chr1	193667681	193728484	60803	5	chr1	193667650	193741836	74186	HiConf	Variation_38814	chr1	193676328	193682368	6040	McCarroll et al. (2008)	Affymetrix Human SNP Array 6.0	
100064	chr1	193955548	193971251	15703	2	chr1	193955500	194007930	52430	single								y
393	chr1	194978455	195184967	206512	40	chr1	194978424	195185351	206927	HiConf	Variation_31680	chr1	194977757	195193635	215878	Perry et al. (2008)	Agilent Custom CGH Arrays	
394	chr1	195228600	195242919	14319	2	chr1	195228569	195243073	14504	HiConf	Variation_34583	chr1	195006454	195274102	267648	Zogopoulos et al. (2007)	Affymetrix 500K and 100K SNP Mapping Arrays	
395	chr1	195585957	195599187	13230	8	chr1	195585926	195599450	13524	single								
396	chr1	195988198	195989129	931	5	chr1	195988167	195989147	980	single								
397	chr1	196160699	196160790	91	53	chr1	196160696	196160794	98	single	Variation_44485	chr1	196160644	196160936	292	Bentley et al. (2008)	Illumina DNA sequencing	
398	chr1	196571200	196576735	5535	18	chr1	196571169	196576902	5733	HiConf	Variation_34753	chr1	196569006	196582349	13343	Kidd et al. (2008)	Paired End Mapping	
399	chr1	197040592	197041355	763	12	chr1	197040589	197041373	784	single	Variation_39028	chr1	197040372	197042037	1665	Wheeler et al. (2008)	Sequencing	
400	chr1	197375682	197380386	4704	57	chr1	197375651	197380453	4802	HiConf	Variation_38098	chr1	197378049	197380463	2414	McCarroll et al. (2008)	Affymetrix Human SNP Array 6.0	
401	chr1	198522233	198535365	13132	15	chr1	198522202	198535628	13426	HiConf								
402	chr1	199441767	199447627	5860	36	chr1	199441736	199447665	5929	HiConf								
403	chr1	200567983	200576803	8820	1	chr1	200567952	200576968	9016	single								
100066	chr1	200613920	200798347	184427	28	chr1	200613743	200802099	188356	HiConf	Variation_44491	chr1	200627346	200627872	526	Bentley et al. (2008)	Illumina DNA sequencing	
405	chr1	201951645	201952527	882	23	chr1	201951614	201952545	931	HiConf								
406	chr1	202161267	202161657	390	55	chr1	202161236	202162020	784	HiConf								
407	chr1	202171312	202179152	7840	6	chr1	202171281	202179317	8036	single								
408	chr1	202204926	202211845	6919	4	chr1	202204601	202212000	7399	single	Variation_38165	chr1	202204481	202212021	7540	McCarroll et al. (2008)	Affymetrix Human SNP Array 6.0	
409	chr1	202648964	202651169	2205	29	chr1	202648933	202651187	2254	HiConf								
100067	chr1	204187319	204356614	169295	3	chr1	204187288	204360038	172750	HiConf	Variation_4268	chr1	204186230	204407370	221140	Wong et al. (2007)	BAC Array CGH	
412	chr1	204367149	204374845	7696	24	chr1	204367118	204375252	8134	HiConf	Variation_4268	chr1	204186230	204407370	221140	Wong et al. (2007)	BAC Array CGH	
100068	chr1	204579728	204646645	66917	3	chr1	204497801	204649652	151851	HiConf								
413	chr1	205107297	205111508	4211	2	chr1	205107263	205111575	4312	single	Variation_2091	chr1	204922611	205124381	201770	Locke et al. (2006)	BAC Array CGH	

locus_id	chrom	start	end	length	Yoruba w/ event	putative chr	putative start	putative end	putative len	putative type	variation_id	DGV_chr	DGV_start	DGV_end	DGV_len	Reference	Method/platform	complex
414	chr1	205359105	205359791	686	84	chr1	205359074	205359809	735	HiConf								
100069	chr1	205386815	205413079	26264	3	chr1	205386784	205413587	26803	single								
416	chr1	205533986	205536975	2989	5	chr1	205533955	205537042	3087	single								
417	chr1	205541287	205560719	19432	3	chr1	205541256	205561101	19845	single								
418	chr1	205609054	205612092	3038	45	chr1	205609023	205612159	3136	HiConf	Variation_23348	chr1	205608658	205612259	3601	Levy et al. (2007)	Sequencing	
419	chr1	205763453	205817964	54511	25	chr1	205763422	205818253	54831	HiConf	Variation_31682	chr1	205764096	205817763	53667	Perry et al. (2008)	Agilent Custom CGH Arrays	y
420	chr1	205908395	205911972	3577	26	chr1	205908364	205912039	3675	HiConf	Variation_31683	chr1	205908611	205913262	4651	Perry et al. (2008)	Agilent Custom CGH Arrays	
421	chr1	205922507	205927456	4949	32	chr1	205922476	205927572	5096	HiConf	Variation_3328	chr1	205624502	205991690	367188	Redon et al. (2006)	BAC Array CGH	
422	chr1	205928286	205931278	2992	18	chr1	205927915	205931345	3430	HiConf	Variation_3328	chr1	205624502	205991690	367188	Redon et al. (2006)	BAC Array CGH	
423	chr1	206082835	206087245	4410	3	chr1	206082804	206087312	4508	HiConf								
424	chr1	208144608	208153388	8780	45	chr1	208144577	208153544	8967	HiConf	Variation_43758	chr1	208144677	208152598	7921	Bentley et al. (2008)	Illumina DNA sequencing	y
425	chr1	208668173	208680517	12344	10	chr1	208668142	208680735	12593	HiConf	Variation_9913	chr1	208670753	208680239	9486	Wang et al. (2007)	Illumina HumanHap550 BeadChip	
426	chr1	208789007	208791555	2548	17	chr1	208788976	208791622	2646	HiConf	Variation_43487	chr1	208788882	208791631	2749	Wang et al. (2008)	Illumina DNA sequencing	
427	chr1	210392042	210394149	2107	2	chr1	210392011	210394167	2156	single								
428	chr1	211638406	211643551	5145	2	chr1	211638375	211643667	5292	single								
429	chr1	212919952	212922122	2170	4	chr1	212919921	212922518	2597	HiConf	Variation_38167	chr1	212919913	212922145	2232	McCarroll et al. (2008)	Affymetrix Human SNP Array 6.0	
430	chr1	213657598	213665389	7791	2	chr1	213657567	213665554	7987	single								
431	chr1	214298224	214298332	108	40	chr1	214298193	214298340	147	single								
432	chr1	215766166	215769841	3675	4	chr1	215766135	215769908	3773	single	Variation_4270	chr1	215701253	215854656	153403	Wong et al. (2007)	BAC Array CGH	
433	chr1	216484555	216488132	3577	8	chr1	216484524	216488199	3675	HiConf	Variation_38170	chr1	216484875	216489763	4888	McCarroll et al. (2008)	Affymetrix Human SNP Array 6.0	y
434	chr1	219014376	219015062	686	26	chr1	219014345	219015080	735	single								
435	chr1	219391872	219392901	1029	3	chr1	219391841	219392919	1078	single								
436	chr1	219810675	219821700	11025	10	chr1	219810644	219821914	11270	HiConf	Variation_38171	chr1	219812673	219820232	7559	McCarroll et al. (2008)	Affymetrix Human SNP Array 6.0	
437	chr1	219871631	219872415	784	3	chr1	219871600	219872433	833	single								
438	chr1	220440178	220449047	8869	43	chr1	220440147	220449212	9065	HiConf	Variation_30455	chr1	220441317	220449363	8046	Perry et al. (2008)	Agilent Custom CGH Arrays	
439	chr1	220715215	220758384	43169	5	chr1	220715184	220759235	44051	single								
440	chr1	221793068	221801986	8918	1	chr1	221793037	221802151	9114	single								
100072	chr1	222136990	222290235	153245	10	chr1	222136895	222293352	156457	HiConf	Variation_31689	chr1	222155539	222159164	3625	Perry et al. (2008)	Agilent Custom CGH Arrays	
442	chr1	222391946	222393461	1515	7	chr1	222391915	222393483	1568	HiConf	Variation_3331	chr1	222001843	222429786	427943	Redon et al. (2006)	BAC Array CGH	
443	chr1	223059816	223062658	2842	7	chr1	223059785	223062725	2940	HiConf								
444	chr1	223191528	223192753	1225	7	chr1	223191497	223192771	1274	single	Variation_3332	chr1	223154547	223319897	165350	Redon et al. (2006)	BAC Array CGH	
445	chr1	225232378	225233162	784	30	chr1	225232347	225233180	833	HiConf	Variation_42858	chr1	225232825	225233388	563	Wang et al. (2008)	Illumina DNA sequencing	
446	chr1	225509718	225515990	6272	39	chr1	225509687	225516106	6419	HiConf								
447	chr1	226316160	226326695	10535	45	chr1	226316129	226326909	10780	HiConf	Variation_6812	chr1	225946739	226872832	926093	de Smith et al. (2007)	Agilent 185k CGH Arrays/Agilent Custom CGH Arrays	
448	chr1	226493736	226495451	1715	17	chr1	226493705	226495469	1764	single	Variation_6812	chr1	225946739	226872832	926093	de Smith et al. (2007)	Agilent 185k CGH Arrays/Agilent Custom CGH Arrays	
449	chr1	226550207	226555832	5625	4	chr1	226546576	226557846	11270	single	Variation_29848	chr1	226530871	226612865	81994	Jakobsson et al. (2008)	Illumina HumanHap550 BeadChip	
450	chr1	226637791	226644854	7063	2	chr1	226635168	226647810	12642	single	Variation_39695	chr1	226638783	226639112	329	Wheeler et al. (2008)	Sequencing	
451	chr1	226849378	226872506	23128	8	chr1	226849347	226872965	23618	single	Variation_6812	chr1	225946739	226872832	926093	de Smith et al. (2007)	Agilent 185k CGH Arrays/Agilent Custom CGH Arrays	
452	chr1	227477950	227480008	2058	20	chr1	227476939	227480075	3136	HiConf								
453	chr1	227878280	227883866	5586	34	chr1	227878249	227883982	5733	HiConf	Variation_31691	chr1	227879571	227882341	2770	Perry et al. (2008)	Agilent Custom CGH Arrays	
454	chr1	228199622	228200357	735	44	chr1	228199591	228200375	784	HiConf	Variation_43763	chr1	228199169	228200503	1334	Bentley et al. (2008)	Illumina DNA sequencing	
455						chr1	228598500	228598598	98	single								
456	chr1	228599462	228608037	8575	17	chr1	228599431	228608202	8771	HiConf								
457	chr1	228646845	228655812	8967	8	chr1	228646814	228655977	9163	HiConf								
458	chr1	229386843	229388264	1421	16	chr1	229386812	229388282	1470	single	Variation_9914	chr1	229387089	229388566	1477	Wang et al. (2007)	Illumina HumanHap550 BeadChip	
100074	chr1	229480801	229504615	23814	2	chr1	229480770	229505074	24304	HiConf								
460	chr1	229759341	229761301	1960	2	chr1	229759310	229761319	2009	single	Variation_3334	chr1	229691383	229982619	291236	Redon et al. (2006)	BAC Array CGH	
461	chr1	230525309	230526828	1519	25	chr1	230525278	230526846	1568	HiConf	Variation_43764	chr1	230525218	230526873	1655	Bentley et al. (2008)	Illumina DNA sequencing	
462	chr1	230920935	230933479	12544	23	chr1	230920904	230933742	12838	HiConf	Variation_8348	chr1	230661638	231445842	784204	Pinto et al. (2007)	Affymetrix 500K SNP Mapping Array	
463	chr1	231503104	231518686	15582	8	chr1	231503073	231518998	15925	single								
464	chr1	232400539	232411809	11270	5	chr1	232400508	232412023	11515	single	Variation_4271	chr1	232330965	232487226	156261	Wong et al. (2007)	BAC Array CGH	
100075	chr1	232953258	233020953	67695	23	chr1	232937965	233022661	84696	HiConf	Variation_30459	chr1	232979290	233023542	44252	Perry et al. (2008)	Agilent Custom CGH Arrays	
466	chr1	233939433	233939833	400	23	chr1	233939402	233939843	441	single								
100076	chr1	234310887	234363388	52501	3	chr1	234297396	234364722	67326	HiConf	Variation_34782	chr1	234325435	234344750	19315	Kidd et al. (2008)	Paired End Mapping	
468	chr1	235188149	235199125	10976	4	chr1	235188118	235199339	11221	HiConf	Variation_9390	chr1	235190394	235199343	8949	Wang et al. (2007)	Illumina HumanHap550 BeadChip	
469	chr1	235409286	235410021	735	3	chr1	235409255	235410039	784	HiConf	Variation_39039	chr1	235408376	235439423	31047	Wheeler et al. (2008)	Sequencing	
470	chr1	235630423	235636074	5651	2	chr1	235630392	235636223	5831	single								
471	chr1	236106017	236111456	5439	5	chr1	236105986	236111572	5586	single	Variation_8350	chr1	236079393	236792582	713189	Pinto et al. (2007)	Affymetrix 500K SNP Mapping Array	
472	chr1	236383406	236384680	1274	11	chr1	236383375	236384698	1323	HiConf	Variation_8350	chr1	236079393	236792582	713189	Pinto et al. (2007)	Affymetrix 500K SNP Mapping Array	
473	chr1	236550986	236553142	2156	3	chr1	236550955	236553160	2205	HiConf	Variation_31695	chr1	236550923	236552911	1988	Perry et al. (2008)	Agilent Custom CGH Arrays	
474	chr1	236817644	236818575	931	11	chr1	236817613	236818593	980	HiConf	Variation_0982	chr1	236817115	236819235	2120	Hinds et al. (2005)	Oligo arrays	
475	chr1	238394268	238429597	35329	28	chr1	238394237	238430301	36064	HiConf	Variation_0262	chr1	238429482	238461503	32021	Sebat et al. (2004)	ROMA	
476	chr1	238460347	238461398	1051	36	chr1	238460338	238461416	1078	HiConf	Variation_31696	chr1	238459926	238461331	1405	Perry et al. (2008)	Agilent Custom CGH Arrays	y
477	chr1	239653372	239662878	9506	11	chr1	239653341	239663043	9702	HiConf								
100078	chr1	239871762	239920605	48843	5	chr1	239859068	239949522	90454	HiConf								
100079	chr1	241102555	241342439	239884	8	chr1	241102222	241347271	245049	HiConf	Variation_2355	chr1	241103290	241347508	244218	Redon et al. (2006)	Affymetrix 500K EA SNP Mapping Array	
481	chr1	241752875	241761891	9016	16	chr1	241752844	241762056	9212	HiConf								
482	chr1	242672066	242673830	1764	5	chr1	242672035	242673848	1813	single								
100080	chr1	243053972	243097009	43037	8	chr1	243053941	243098164	44223	HiConf								
483	chr1	243160449	243175578	15129	6	chr1	243160418	243176294	15876	single								
484	chr1	243848605	243849055	450	10	chr1	243848574	243849064	490	single								
485	chr1	243912501	243912609	108	70	chr1	243912470	243912617	147	HiConf								
486	chr1	244435919	244455029	19110	3	chr1	244435888	244455390	19502	HiConf								
487	chr1	244542543	244568611	26068	19	chr1	244542512	244569119	26607	HiConf	Variation_3338	chr1	244473769	244807567	333798	Redon et al. (2006)	BAC Array CGH	
488	chr1	244806289	244808221	1932	12	chr1	244806279	244808239	1960	HiConf	Variation_31699	chr1	244806258	244808278	2020	Perry et al. (2008)	Agilent Custom CGH Arrays	
489	chr1	245336196	245336833	637	22	chr1	245336165	245336851	686	single	Variation_6816	chr1	245141053	246395458	1254405	de Smith et al. (2007)	Agilent 185k CGH Arrays/Agilent Custom CGH Arrays	y
490	chr1	245342615	245360369	17754	43	chr1	245342584	245360371	17787	HiConf	Variation_24154	chr1	245358948	245359505	557	Levy et al. (2007)	Sequencing	
491	chr1	245779156	245796502	17346	1	chr1	245779125	245796863	17738	single	Variation_9917	chr1	245782621	245795585	12964	Wang et al. (2007)	Illumina HumanHap550 BeadChip	
492	chr1	246211238	246212169	931	1	chr1	246211207	246212187	980	single	Variation_31701	chr1	246210114	246213302	3188	Perry et al. (2008)	Agilent Custom CGH Arrays	
493	chr1	246221234	246221920	686	34	chr1	246221203	246221938	735	HiConf	Variation_3339	chr1	245781208	246343769	562561	Redon et al. (2006)	BAC Array CGH	
494	chr1	246246126	246357404	111278	1	chr1	246246095	246359089	112994	single	Variation_43064	chr1	246274275	246274951	676	Wang et al. (2008)	Illumina DNA sequencing	y
495	chr1	246613185	246614917	1732	51	chr1	246613154	246614918	1764	HiConf	Variation_31703	chr1	246613167	246614980	1813	Perry et al. (2008)	Agilent Custom CGH Arrays	y
496	chr1	246623204	246650735	27531	17	chr1	246623150	246651227	28077	HiConf	Variation_34796	chr1	246635008	246646934	11926	Kidd et al. (2008)	Paired End Mapping	
497	chr1	246713357	246723389	10032	1	chr1	246702040	246764760	62720	single	Variation_6817	chr1	246676880	246713386	36506	de Smith et al. (2007)	Agilent 185k CGH Arrays/Agilent Custom CGH Arrays	
498	chr1	246765007	246769348	4341	2	chr1	246765005	246769415	4410	single	Variation_5571	chr1	246760996	246767346	6350	Mills et al. (2006)	Sequence trace read mapping	y
499	chr1	246769816	246861928	92112	46	chr1	246769807	246863299	93492	HiConf	Variation_6818	chr1	246794551	246863305	68754	de Smith et al. (2007)	Agilent 185k CGH Arrays/Agilent Custom CGH Arrays	

locus_id	chrom	start	end	length	Yoruba w/ event	putative chr	putative start	putative end	putative len	putative type	variation_id	DGV_chr	DGV_start	DGV_end	DGV_len	Reference	Method/platform	complex
500	chr1	246865780	246867936	2156	14	chr1	246865749	246867954	2205	single	Variation_30468	chr1	246865937	246869166	3229	Perry et al. (2008)	Agilent Custom CGH Arrays	y
501	chr1	246933547	246960987	27440	42	chr1	246933516	246961544	28028	HiConf	Variation_37402	chr1	246933722	246942061	8339	Cooper et al. (2008)	Illumina Human 1M BeadChip	y
502	chr1	247056341	247056978	637	24	chr1	247056310	247056996	686	HiConf	Variation_8355	chr1	246589303	247124862	535559	Pinto et al. (2007)	Affymetrix 500K SNP Mapping Array	
3524	chr10	50531	118437	67906	14	chr10	50446	119193	68747	HiConf	Variation_30470	chr10	84352	123437	39085	Perry et al. (2008)	Agilent Custom CGH Arrays	
3525	chr10	384902	393036	8134	15	chr10	384871	393201	8330	single								
3526	chr10	415870	416360	490	6	chr10	415839	416378	539	single								
3527	chr10	584773	605687	20914	18	chr10	584742	606106	21364	HiConf	Variation_4664	chr10	456002	621099	165097	Wong et al. (2007)	BAC Array CGH	y
100576	chr10	689961	726422	36461	46	chr10	685609	726622	41013	single								y
3529	chr10	773619	793570	19951	37	chr10	773588	793580	19992	HiConf	Variation_24567	chr10	793296	793499	203	Levy et al. (2007)	Sequencing	y
3530	chr10	1263815	1283417	19602	52	chr10	1263784	1283629	19845	HiConf	Variation_24361	chr10	1271639	1271822	183	Levy et al. (2007)	Sequencing	
3531	chr10	1415519	1424339	8820	9	chr10	1415488	1424504	9016	single								
100577	chr10	1511296	1545492	34196	5	chr10	1511210	1546171	34961	single								
3532	chr10	2417716	2434768	17052	16	chr10	2417685	2435129	17444	HiConf								y
3533	chr10	2550163	2556460	6297	34	chr10	2550132	2556502	6370	single								
3534	chr10	2557366	2557816	450	33	chr10	2557335	2557825	490	single								y
3535	chr10	2558150	2558640	490	30	chr10	2558119	2558658	539	single								
3536	chr10	2612344	2613961	1617	6	chr10	2612313	2613979	1666	HiConf								
3537	chr10	2864596	2868222	3626	4	chr10	2864565	2868289	3724	HiConf	Variation_29582	chr10	2682656	3123648	440992	Jakobsson et al. (2008)	Illumina HumanHap550 BeadChip	
3539	chr10	3262966	3263029	63	18	chr10	3262935	3263033	98	single	Variation_6858	chr10	3198223	3292032	93809	de Smith et al. (2007)	Agilent 185k CGH Arrays/Agilent Custom CGH Arrays	
3540	chr10	3405556	3405956	400	21	chr10	3405525	3405966	441	HiConf								
3541	chr10	3488758	3490767	2009	11	chr10	3488727	3490785	2058	single								
3542	chr10	3564414	3565296	882	26	chr10	3564383	3565314	931	HiConf	Variation_43793	chr10	3564227	3565396	1169	Bentley et al. (2008)	Illumina DNA sequencing	y
3543	chr10	4279765	4282231	2466	51	chr10	4279734	4282233	2499	HiConf	Variation_43368	chr10	4280012	4281758	1746	Wang et al. (2008)	Illumina DNA sequencing	
3544	chr10	4364682	4366201	1519	3	chr10	4364651	4366219	1568	single								
3545	chr10	4427794	4431273	3479	17	chr10	4427763	4431340	3577	HiConf	Variation_31709	chr10	4427898	4430742	2844	Perry et al. (2008)	Agilent Custom CGH Arrays	
3546	chr10	4698929	4699891	962	56	chr10	4698929	4699909	980	HiConf	Variation_39043	chr10	4698518	4700524	2006	Wheeler et al. (2008)	Sequencing	
3547	chr10	4847822	4848034	212	48	chr10	4847791	4848036	245	single	Variation_24501	chr10	4847883	4848103	220	Levy et al. (2007)	Sequencing	
3548	chr10	5239969	5240557	588	20	chr10	5239938	5240575	637	HiConf	Variation_43699	chr10	5192052	5314829	122777	Wang et al. (2008)	Illumina DNA sequencing	
100579	chr10	5442406	5499106	56700	3	chr10	5396175	5501182	105007	single								
100580	chr10	5623590	5771227	147637	1	chr10	5623559	5774234	150675	HiConf	Variation_30473	chr10	5689917	5757181	67264	Perry et al. (2008)	Agilent Custom CGH Arrays	
3551	chr10	5930407	5932290	1883	54	chr10	5930397	5932308	1911	HiConf	Variation_5574	chr10	5929629	5932883	3254	Mills et al. (2006)	Sequence trace read mapping	
3552	chr10	6679197	6681451	2254	17	chr10	6679166	6681469	2303	single								
3553	chr10	6717844	6742619	24775	10	chr10	6717729	6743013	25284	HiConf								
3554	chr10	7117453	7118237	784	6	chr10	7117422	7118255	833	HiConf	Variation_11039	chr10	7117477	7117975	498	de Smith et al. (2007)	Agilent 185k CGH Arrays/Agilent Custom CGH Arrays	
100581	chr10	7132069	7178941	46872	1	chr10	7132000	7179873	47873	single	Variation_30476	chr10	7134485	7179277	44792	Perry et al. (2008)	Agilent Custom CGH Arrays	
3556	chr10	8035811	8037093	1282	13	chr10	8035780	8037103	1323	HiConf	Variation_34302	chr10	8036281	8036756	475	Levy et al. (2007)	Sequencing	
3557	chr10	8876749	8902131	25382	18	chr10	8876718	8902639	25921	HiConf								
100582						chr10	10548304	10576847	28543	single								
3558	chr10	10696119	10700480	4361	2	chr10	10696088	10700547	4459	single								
3559	chr10	11144567	11150888	6321	11	chr10	11144536	11151004	6468	HiConf	Variation_1217	chr10	11144870	11148911	4041	Conrad et al. (2005)	Mendelian inconsistencies	y
3560	chr10	11961054	11973004	11950	27	chr10	11961023	11973126	12103	single								
100583	chr10	12134835	12203454	68619	2	chr10	12134802	12204994	70192	single	Variation_29583	chr10	12140741	12205253	64512	Jakobsson et al. (2008)	Illumina HumanHap550 BeadChip	
3562	chr10	12585657	12588940	3283	1	chr10	12585626	12589007	3381	single								
3563	chr10	12693175	12716585	23410	25	chr10	12693132	12717044	23912	HiConf								
3564	chr10	13553995	13556690	2695	8	chr10	13553964	13556757	2793	HiConf								
100584	chr10	14091525	14109312	17787	2	chr10	14091494	14109796	18302	single								
3566	chr10	14525273	14526535	1262	2	chr10	14525242	14526565	1323	single	Variation_47859	chr10	14426301	14833838	407537	Gusev et al. (2009)	SNP genotyping analysis	
3567	chr10	14574126	14575645	1519	4	chr10	14574095	14575663	1568	single	Variation_47859	chr10	14426301	14833838	407537	Gusev et al. (2009)	SNP genotyping analysis	
3568	chr10	15711073	15714062	2989	2	chr10	15711042	15714129	3087	HiConf								
3569	chr10	15761249	15771441	10192	30	chr10	15761218	15771655	10437	HiConf	Variation_4667	chr10	15765869	15914974	149105	Wong et al. (2007)	BAC Array CGH	
3570	chr10	17909850	17914211	4361	18	chr10	17909819	17914278	4459	HiConf								
3571	chr10	18156663	18162004	5341	17	chr10	18156632	18162120	5488	HiConf								
3572	chr10	18460505	18461093	588	1	chr10	18459550	18463764	4214	single								
3573	chr10	18788224	18800817	12593	4	chr10	18788193	18801080	12887	HiConf								
3574	chr10	18869193	18915669	46476	48	chr10	18869141	18916181	47040	HiConf	Variation_2852	chr10	18875331	18946513	71182	Redon et al. (2006)	Affymetrix 500K EA SNP Mapping Array	
3575	chr10	19218052	19226295	8243	4	chr10	19218021	19226449	8428	HiConf	Variation_34866	chr10	19203838	19239139	35301	Kidd et al. (2008)	Paired End Mapping	
100585	chr10	19744421	19767965	23544	1	chr10	19733599	19782697	49098	single	Variation_10319	chr10	19750338	19778819	28481	Wang et al. (2007)	Illumina HumanHap550 BeadChip	
3577						chr10	20269022	20269071	49	single								
3578	chr10	20890667	20897311	6644	4	chr10	20890636	20897398	6762	HiConf	Variation_31713	chr10	20890946	20897367	6421	Perry et al. (2008)	Agilent Custom CGH Arrays	
3579	chr10	22192303	22200559	8256	3	chr10	22192272	22200896	8624	HiConf								
3580	chr10	23055634	23056663	1029	31	chr10	23055603	23056681	1078	HiConf	Variation_4670	chr10	22958107	23109682	151575	Wong et al. (2007)	BAC Array CGH	
3581	chr10	23740899	23748102	7203	12	chr10	23740868	23748267	7399	HiConf								
3582	chr10	24419010	24421899	2889	12	chr10	24418979	24424369	5390	HiConf								
3583	chr10	25442571	25446589	4018	9	chr10	25442540	25446656	4116	single								
3584	chr10	26272230	26273202	972	1	chr10	26269121	26277255	8134	single								
3585	chr10	26649245	26651058	1813	6	chr10	26649214	26651076	1862	HiConf								
3586	chr10	26724901	26729605	4704	18	chr10	26724870	26729672	4802	HiConf	Variation_43791	chr10	26724560	26729853	5293	Bentley et al. (2008)	Illumina DNA sequencing	
3587	chr10	27039628	27041833	2205	36	chr10	27039597	27041851	2254	HiConf	Variation_5588	chr10	27038983	27042120	3137	Mills et al. (2006)	Sequence trace read mapping	
3588	chr10	27651877	27676481	24604	11	chr10	27651803	27676989	25186	HiConf	Variation_34869	chr10	27662539	27694593	32054	Kidd et al. (2008)	Paired End Mapping	
3589	chr10	27678343	27681969	3626	11	chr10	27678312	27682036	3724	HiConf	Variation_43555	chr10	27678193	27682182	3989	Wang et al. (2008)	Illumina DNA sequencing	
3590	chr10	28078036	28088522	10486	6	chr10	28078005	28088736	10731	single								
3591	chr10	28401436	28401975	539	8	chr10	28401405	28401993	588	single								
3592	chr10	28543438	28550886	7448	3	chr10	28543407	28551051	7644	HiConf	Variation_34870	chr10	28529082	28554074	24992	Kidd et al. (2008)	Paired End Mapping	
3593	chr10	28604737	28612871	8134	4	chr10	28604706	28613036	8330	HiConf	Variation_10321	chr10	28602653	28611866	9213	Wang et al. (2007)	Illumina HumanHap550 BeadChip	
3594	chr10	28704060	28704991	931	14	chr10	28704029	28705009	980	HiConf								
3595	chr10	29314992	29315923	931	3	chr10	29314961	29315941	980	single								
3596	chr10	29369725	29375360	5635	4	chr10	29369694	29375476	5782	single								
3597	chr10	30662198	30665236	3038	2	chr10	30662167	30665303	3136	single								
3598	chr10	31239516	31244514	4998	2	chr10	31239485	31244630	5145	single								
3599	chr10	31539053	31539788	735	12	chr10	31539022	31539806	784	HiConf								
3600	chr10	31592071	31593492	1421	2	chr10	31592040	31593510	1470	HiConf								
3601	chr10	31617208	31621373	4165	3	chr10	31617177	31621440	4263	single								
3602	chr10	32084276	32088147	3871	4	chr10	32084245	32088214	3969	single								
3603	chr10	32225445	32253326	27881	11	chr10	32225414	32253883	28469	HiConf								
3604						chr10	32666365	32667982	1617	single								
3605	chr10	34555689	34563088	7399	5	chr10	34555658	34563253	7595	HiConf	Variation_38195	chr10	34557071	34562360	5289	McCarroll et al. (2008)	Affymetrix Human SNP Array 6.0	

locus_id	chrom	start	end	length	Yoruba w/ event	putative chr	putative start	putative end	putative len	putative type	variation_id	DGV_chr	DGV_start	DGV_end	DGV_len	Reference	Method/platform	complex
3606	chr10	34794760	34796181	1421	6	chr10	34794729	34796199	1470	HiConf								
3607	chr10	35996877	36001728	4851	14	chr10	35996846	36001844	4998	HiConf								
3608	chr10	36260252	36260791	539	9	chr10	36260221	36260809	588	single	Variation_6859	chr10	36259824	36260926	1102	de Smith et al. (2007)	Agilent 185k CGH Arrays/Agilent Custom CGH Arrays	
3609	chr10	36432428	36434888	2460	1	chr10	36431917	36434955	3038	single	Variation_4676	chr10	36343125	36519689	176564	Wong et al. (2007)	BAC Array CGH	
3610	chr10	36454635	36455174	539	5	chr10	36454604	36455192	588	single	Variation_4676	chr10	36343125	36519689	176564	Wong et al. (2007)	BAC Array CGH	
3611	chr10	37060598	37061500	902	10	chr10	37059460	37061518	2058	single								
100588	chr10	37441520	37545890	104370	19	chr10	37441489	37548015	106526	HiConf	Variation_31715	chr10	37483539	37529500	45961	Perry et al. (2008)	Agilent Custom CGH Arrays	
3614	chr10	37856231	37863679	7448	19	chr10	37856200	37863844	7644	HiConf	Variation_31716	chr10	37856644	37861684	5040	Perry et al. (2008)	Agilent Custom CGH Arrays	
3615	chr10	38055171	38055621	450	17	chr10	38055140	38055630	490	single								
3616	chr10	38814832	38922575	107743	39	chr10	38814787	38924204	109417	HiConf	Variation_24486	chr10	38843201	38843311	110	Levy et al. (2007)	Sequencing	
3617						chr10	38953065	39115745	162680	HiConf								
3618	chr10	41774173	41850466	76293	14	chr10	41774142	41852003	77861	HiConf	Variation_4771	chr10	41709405	41918048	208643	Wong et al. (2007)	BAC Array CGH	
3619	chr10	41926612	41928817	2205	39	chr10	41926581	41928835	2254	HiConf	Variation_2861	chr10	41753546	42249122	495576	Redon et al. (2006)	Affymetrix 500K EA SNP Mapping Array	
3620	chr10	42099484	42101983	2499	16	chr10	42099453	42102050	2597	HiConf	Variation_3796	chr10	42004899	42199692	194793	Redon et al. (2006)	BAC Array CGH	
3621	chr10	42348208	42348992	784	9	chr10	42348177	42349010	833	single	Variation_2152	chr10	42285138	42498329	213191	Locke et al. (2006)	BAC Array CGH	
3622	chr10	42503195	42506478	3283	13	chr10	42503164	42506545	3381	HiConf	Variation_6862	chr10	42503824	42506478	2654	de Smith et al. (2007)	Agilent 185k CGH Arrays/Agilent Custom CGH Arrays	
3623	chr10	42559647	42564837	5190	8	chr10	42559612	42564953	5341	single	Variation_31723	chr10	42555924	42576703	20779	Perry et al. (2008)	Agilent Custom CGH Arrays	
3624	chr10	43524551	43525580	1029	47	chr10	43524520	43525598	1078	HiConf								
3625	chr10	43604519	43605548	1029	26	chr10	43604488	43605566	1078	single								y
3626	chr10	44475543	44486674	11131	47	chr10	44475512	44486831	11319	HiConf	Variation_44744	chr10	44478129	44478533	404	Bentley et al. (2008)	Illumina DNA sequencing	
3627	chr10	44730833	44733675	2842	4	chr10	44730802	44733742	2940	HiConf								
3628	chr10	44747150	44751658	4508	17	chr10	44747119	44751725	4606	single	Variation_10324	chr10	44740082	44751327	11245	Wang et al. (2007)	Illumina HumanHap550 BeadChip	
3629	chr10	45003175	45011750	8575	11	chr10	45003144	45011915	8771	single	Variation_9148	chr10	44870151	45140142	269991	Pinto et al. (2007)	Affymetrix 500K SNP Mapping Array	
3630	chr10	45049039	45051440	2401	20	chr10	45049008	45051507	2499	HiConf	Variation_43797	chr10	45048776	45051648	2872	Bentley et al. (2008)	Illumina DNA sequencing	
3631	chr10	45494305	45600877	106572	9	chr10	45494271	45603051	108780	HiConf	Variation_6866	chr10	45525725	45554746	29021	de Smith et al. (2007)	Agilent 185k CGH Arrays/Agilent Custom CGH Arrays	
3632	chr10	45603199	45631703	28504	5	chr10	45603198	45632255	29057	HiConf	Variation_33522	chr10	45625486	45626243	757	Perry et al. (2008)	Agilent Custom CGH Arrays	
3633	chr10	45669624	45735382	65758	11	chr10	45669593	45736723	67130	HiConf	Variation_31727	chr10	45688966	45729628	40662	Perry et al. (2008)	Agilent Custom CGH Arrays	
3634	chr10	46121208	46131988	10780	10	chr10	46121177	46132202	11025	single	Variation_8651	chr10	45530757	46557002	1026245	Pinto et al. (2007)	Affymetrix 500K SNP Mapping Array	
3635	chr10	46573723	46590775	17052	9	chr10	46573692	46591136	17444	HiConf	Variation_4685	chr10	46562221	46744982	182761	Wong et al. (2007)	BAC Array CGH	y
3636	chr10	46849054	47123375	274321	37	chr10	46849023	47123570	274547	HiConf	Variation_29592	chr10	47013327	47122505	109178	Jakobsson et al. (2008)	Illumina HumanHap550 BeadChip	
3637	chr10	47180833	47237736	56903	9	chr10	47180802	47238916	58114	HiConf	Variation_1483	chr10	47167032	47219448	52416	Conrad et al. (2005)	Mendelian inconsistencies	
100592	chr10	47450835	47477259	26424	2	chr10	47261432	47481662	220230	single	Variation_34520	chr10	47030100	47485249	455149	Zogopoulos et al. (2007)	Affymetrix 500K and 100K SNP Mapping Arrays	y
3639	chr10	47485907	47488455	2548	36	chr10	47485876	47488522	2646	single	Variation_30488	chr10	47483536	47488691	5155	Perry et al. (2008)	Agilent Custom CGH Arrays	
3640	chr10	47726007	47810532	84525	10	chr10	47725976	47812265	86289	HiConf	Variation_4690	chr10	47795721	48000264	204543	Wong et al. (2007)	BAC Array CGH	
3641	chr10	47813423	47902210	88787	9	chr10	47813392	47903944	90552	HiConf	Variation_4690	chr10	47795721	48000264	204543	Wong et al. (2007)	BAC Array CGH	
3642	chr10	48210421	48214243	3822	8	chr10	48210390	48214310	3920	HiConf	Variation_43799	chr10	48210395	48214378	3983	Bentley et al. (2008)	Illumina DNA sequencing	
100594	chr10	48531904	49038693	506789	2	chr10	48367509	49052406	684897	HiConf	Variation_2155	chr10	48865543	49010893	145350	Locke et al. (2006)	BAC Array CGH	
3647	chr10	50724757	50751904	27147	1	chr10	50724678	50752461	27783	HiConf	Variation_31731	chr10	50726630	50761350	34720	Perry et al. (2008)	Agilent Custom CGH Arrays	
3648	chr10	50877099	50887732	10633	5	chr10	50877068	50887946	10878	single	Variation_33524	chr10	50879118	50879874	756	Perry et al. (2008)	Agilent Custom CGH Arrays	
3649	chr10	50895817	50941422	45605	6	chr10	50895786	50942238	46452	HiConf	Variation_31732	chr10	50882690	50922691	40001	Perry et al. (2008)	Agilent Custom CGH Arrays	
3650	chr10	50970787	50982057	11270	5	chr10	50970756	50982271	11515	single	Variation_31733	chr10	50942165	50983081	40916	Perry et al. (2008)	Agilent Custom CGH Arrays	
3651	chr10	51119023	51154341	35318	9	chr10	51118981	51155045	36064	HiConf	Variation_4686	chr10	51121375	51195127	73752	Wong et al. (2007)	BAC Array CGH	
3652	chr10	51186779	51188151	1372	34	chr10	51186748	51188169	1421	HiConf	Variation_43800	chr10	51186726	51188228	1502	Bentley et al. (2008)	Illumina DNA sequencing	
3653	chr10	51269883	51367491	97608	4	chr10	51269852	51369469	99617	HiConf	Variation_31736	chr10	51314334	51316502	2168	Perry et al. (2008)	Agilent Custom CGH Arrays	
3654	chr10	51370382	51401840	31458	5	chr10	51370351	51402495	32144	HiConf	Variation_31737	chr10	51351656	51392585	40929	Perry et al. (2008)	Agilent Custom CGH Arrays	
3655	chr10	51402820	51404780	1960	19	chr10	51402789	51404798	2009	single	Variation_31738	chr10	51402785	51441420	38635	Perry et al. (2008)	Agilent Custom CGH Arrays	
3656	chr10	51405289	51446805	41516	9	chr10	51405288	51447673	42385	HiConf	Variation_31738	chr10	51402785	51441420	38635	Perry et al. (2008)	Agilent Custom CGH Arrays	
3657	chr10	51464140	51571625	107485	5	chr10	51464039	51573799	109760	single	Variation_3800	chr10	50641980	51595172	953192	Redon et al. (2006)	BAC Array CGH	y
3658	chr10	51579710	51585443	5733	27	chr10	51579679	51585559	5880	HiConf	Variation_30499	chr10	51577380	51583991	6611	Perry et al. (2008)	Agilent Custom CGH Arrays	
3659	chr10	51653259	51666832	13573	1	chr10	51653228	51667095	13867	single								
100597	chr10	52077531	52166158	88627	1	chr10	52077250	52171844	94594	single	Variation_30502	chr10	52089664	52131068	41404	Perry et al. (2008)	Agilent Custom CGH Arrays	
3660	chr10	52388651	52389876	1225	9	chr10	52388620	52389894	1274	HiConf								
3661	chr10	53369533	53371113	1580	6	chr10	53369502	53377440	7938	HiConf								
3662	chr10	53685828	53688033	2205	9	chr10	53685797	53688051	2254	HiConf	Variation_43690	chr10	53654230	53704026	49796	Wang et al. (2008)	Illumina DNA sequencing	
3663	chr10	53914021	53918578	4557	1	chr10	53913990	53918645	4655	single								
3664	chr10	54196751	54199103	2352	4	chr10	54196720	54199121	2401	HiConf								
3665	chr10	54231443	54232423	980	17	chr10	54231412	54232441	1029	single								
3666	chr10	54598992	54608106	9114	8	chr10	54598961	54608271	9310	HiConf	Variation_9155	chr10	54599860	54607500	7640	Pinto et al. (2007)	Affymetrix 500K SNP Mapping Array	
3667	chr10	54787887	54810137	22250	5	chr10	54787856	54810592	22736	HiConf	Variation_31741	chr10	54789442	54810431	20989	Perry et al. (2008)	Agilent Custom CGH Arrays	
100598	chr10	55038792	55129001	90209	2	chr10	55038761	55130832	92071	HiConf	Variation_31742	chr10	55042646	55128207	85561	Perry et al. (2008)	Agilent Custom CGH Arrays	
3669	chr10	56190316	56200851	10535	3	chr10	56190285	56201065	10780	HiConf	Variation_3802	chr10	56078033	56236143	158110	Redon et al. (2006)	BAC Array CGH	
100599	chr10	56620596	56667258	46662	1	chr10	56612788	56668378	55590	single	Variation_3803	chr10	56503549	56781399	277850	Redon et al. (2006)	BAC Array CGH	
3671	chr10	56701386	56705747	4361	2	chr10	56701355	56705814	4459	HiConf	Variation_29857	chr10	56705391	56962466	257075	Jakobsson et al. (2008)	Illumina HumanHap550 BeadChip	
3672	chr10	56952413	56954520	2107	3	chr10	56952382	56954538	2156	HiConf	Variation_34883	chr10	56887586	56983615	96029	Kidd et al. (2008)	Paired End Mapping	y
3673	chr10	57307222	57312122	4900	12	chr10	57307191	57312238	5047	HiConf	Variation_8655	chr10	57304818	57449236	144418	Pinto et al. (2007)	Affymetrix 500K SNP Mapping Array	
3674	chr10	58182411	58196817	14406	17	chr10	58182380	58197080	14700	HiConf	Variation_38202	chr10	58186381	58196843	10462	McCarroll et al. (2008)	Affymetrix Human SNP Array 6.0	
100600	chr10	58258933	58286709	27776	1	chr10	58244022	58293659	49637	single	Variation_2871	chr10	58237729	58314518	76789	Redon et al. (2006)	Affymetrix 500K EA SNP Mapping Array	
3676	chr10	58472883	58473667	784	4	chr10	58472852	58473685	833	single								
3677	chr10	58516199	58520952	4753	3	chr10	58516168	58521019	4851	HiConf	Variation_37476	chr10	58516063	58520455	4392	Cooper et al. (2008)	Illumina Human 1M BeadChip	
3678	chr10	58622970	58623230	260	13	chr10	58622939	58623233	294	single								
3679	chr10	58879898	58881739	1841	38	chr10	58879895	58881757	1862	HiConf	Variation_31750	chr10	58879849	58881863	2014	Perry et al. (2008)	Agilent Custom CGH Arrays	
100601	chr10	59507812	59561565	53753	3	chr10	59507781	59562661	54880	single	Variation_3805	chr10	59214388	59554620	340232	Redon et al. (2006)	BAC Array CGH	
100602	chr10	60523166	60578634	55468	3	chr10	60523135	60579754	56619	HiConf	Variation_37577	chr10	60527948	60571609	43661	Cooper et al. (2008)	Illumina Human 1M BeadChip	
100603	chr10	60981273	61002459	21186	1	chr10	60970970	61003090	32120	single	Variation_2875	chr10	60994141	61003145	9004	Redon et al. (2006)	Affymetrix 500K EA SNP Mapping Array	y
3683	chr10	61032006	61036714	4708	29	chr10	61031975	61036728	4753	HiConf	Variation_37988	chr10	61034136	61035270	1134	McCarroll et al. (2008)	Affymetrix Human SNP Array 6.0	y
3684	chr10	61306181	61328453	22272	51	chr10	61305787	61328768	22981	HiConf								
3685	chr10	62097344	62098197	853	17	chr10	62097333	62098215	882	HiConf	Variation_0988	chr10	62097209	62098334	1125	Hinds et al. (2005)	Oligo arrays	
3686	chr10	62288464	62289199	735	3	chr10	62288433	62289217	784	single								
100605	chr10	63300398	63313092	12694	1	chr10	63284211	63341958	57747	single								
3687	chr10	64427755	64432753	4998	2	chr10	64427724	64432869	5145	HiConf								
3688	chr10	65179366	65181081	1715	35	chr10	65179335	65181099	1764	HiConf								
3689	chr10	66068888	66070676	1788	78	chr10	66068881	66070694	1813	HiConf	Variation_39055	chr10	66068776	66070750	1974	Wheeler et al. (2008)	Sequencing	
3690	chr10	66899643	66913378	13735	13	chr10	66899578	66913641	14063	HiConf								
3691	chr10	66966396	66996286	29890	2	chr10	66966365	66996892	30527	HiConf	Variation_34887	chr10	66971127	66999598	28471	Kidd et al. (2008)	Paired End Mapping	
3692	chr10	67001137	67007556	6419	17	chr10	67001106	67007672	6566	HiConf								
3693	chr10	67013436	67014073	637	36	chr10	67013405	67014091	686	HiConf	Variation_37921	chr10	67013460	67013961	501	McCarroll et al. (2008)	Affymetrix Human SNP Array 6.0	

locus_id	chrom	start	end	length	Yoruba w/ event	putative chr	putative start	putative end	putative len	putative type	variation_id	DGV_chr	DGV_start	DGV_end	DGV_len	Reference	Method/platform	complex
3694	chr10	67942231	67948895	6664	4	chr10	67942200	67949011	6811	HiConf	Variation_38206	chr10	67944731	67948321	3590	McCarroll et al. (2008)	Affymetrix Human SNP Array 6.0	
3695	chr10	68709522	68710649	1127	17	chr10	68709491	68710667	1176	HiConf								
3696	chr10	69516993	69522972	5979	1	chr10	69516962	69523087	6125	single								
3697	chr10	70230286	70232344	2058	3	chr10	70230255	70232362	2107	HiConf	Variation_3808	chr10	70127725	70301318	173593	Redon et al. (2006)	BAC Array CGH	
3698	chr10	70283087	70292957	9870	7	chr10	70282636	70293171	10535	HiConf	Variation_38207	chr10	70282650	70292123	9473	McCarroll et al. (2008)	Affymetrix Human SNP Array 6.0	
3699	chr10	70456470	70456960	490	57	chr10	70456439	70456978	539	HiConf								
3700	chr10	70710640	70723079	12439	2	chr10	70710602	70723342	12740	single	Variation_2877	chr10	70708723	70722894	14171	Redon et al. (2006)	Affymetrix 500K EA SNP Mapping Array	
3701	chr10	70728518	70728916	398	16	chr10	70728487	70728928	441	single								
3702	chr10	70951664	70963474	11810	6	chr10	70951633	70963687	12054	HiConf	Variation_37478	chr10	70950995	70961085	10090	Cooper et al. (2008)	Illumina Human 1M BeadChip	
3703	chr10	71467879	71471075	3196	5	chr10	71467848	71471131	3283	HiConf	Variation_6879	chr10	70933992	72216609	1282617	de Smith et al. (2007)	Agilent 185k CGH Arrays/Agilent Custom CGH Arrays	
3704	chr10	71632764	71635557	2793	14	chr10	71632733	71635624	2891	single	Variation_6879	chr10	70933992	72216609	1282617	de Smith et al. (2007)	Agilent 185k CGH Arrays/Agilent Custom CGH Arrays	
3705	chr10	73532102	73535875	3773	3	chr10	73532071	73535942	3871	HiConf	Variation_38208	chr10	73532154	73535907	3753	McCarroll et al. (2008)	Affymetrix Human SNP Array 6.0	y
3706	chr10	74794538	74796939	2401	26	chr10	74794507	74797006	2499	HiConf								
3707	chr10	74855641	74855749	108	10	chr10	74855610	74855757	147	single								
3708	chr10	75042282	75059258	16976	35	chr10	75042251	75059744	17493	HiConf	Variation_22788	chr10	75043199	75051439	8240	Korbel et al. (2007)	Paired End Mapping	
3709	chr10	75089567	75091968	2401	2	chr10	75089536	75092035	2499	single								
3710	chr10	75095501	75160322	64821	8	chr10	75095416	75161762	66346	HiConf	Variation_31755	chr10	75096563	75126882	30319	Perry et al. (2008)	Agilent Custom CGH Arrays	
3711	chr10	76429276	76432020	2744	4	chr10	76429245	76432087	2842	HiConf								
3712	chr10	77073038	77074606	1568	3	chr10	77073007	77074624	1617	single	Variation_4703	chr10	77020206	77190752	170546	Wong et al. (2007)	BAC Array CGH	y
3713	chr10	77919464	77931056	11592	69	chr10	77919433	77934476	15043	HiConf	Variation_6881	chr10	77917922	77928698	10776	de Smith et al. (2007)	Agilent 185k CGH Arrays/Agilent Custom CGH Arrays	
3714	chr10	80274502	80275531	1029	5	chr10	80274471	80275549	1078	single								
100607	chr10	81090627	81243907	153280	10	chr10	81079100	81270715	191615	HiConf	Variation_31758	chr10	81139981	81171386	31405	Perry et al. (2008)	Agilent Custom CGH Arrays	
3716	chr10	81445945	81453540	7595	12	chr10	81445914	81453705	7791	single	Variation_33228	chr10	81450597	81451286	689	Perry et al. (2008)	Agilent Custom CGH Arrays	
3717	chr10	81463340	81539976	76636	12	chr10	81463309	81541513	78204	HiConf	Variation_31761	chr10	81460179	81491562	31383	Perry et al. (2008)	Agilent Custom CGH Arrays	
3718	chr10	81541661	81588547	46886	5	chr10	81541660	81589533	47873	HiConf	Variation_31762	chr10	81535281	81588507	53226	Perry et al. (2008)	Agilent Custom CGH Arrays	y
3719	chr10	84116886	84120218	3332	45	chr10	84116855	84120285	3430	HiConf	Variation_43808	chr10	84117803	84120346	2543	Bentley et al. (2008)	Illumina DNA sequencing	
3720	chr10	84701603	84707385	5782	11	chr10	84701572	84707501	5929	HiConf	Variation_38858	chr10	84702878	84707152	4274	McCarroll et al. (2008)	Affymetrix Human SNP Array 6.0	
3721	chr10	85539013	85542198	3185	10	chr10	85538982	85542265	3283	HiConf								
3722	chr10	86728684	86738455	9771	6	chr10	86728653	86738649	9996	HiConf	Variation_38211	chr10	86728739	86737527	8788	McCarroll et al. (2008)	Affymetrix Human SNP Array 6.0	
3723	chr10	87791004	87798060	7056	3	chr10	87790973	87798176	7203	HiConf	Variation_38212	chr10	87791026	87798355	7329	McCarroll et al. (2008)	Affymetrix Human SNP Array 6.0	
3724	chr10	88865118	88957471	92353	8	chr10	88864759	88959329	94570	HiConf	Variation_31770	chr10	88863954	89014572	150618	Perry et al. (2008)	Agilent Custom CGH Arrays	
3725	chr10	89002921	89103228	100307	20	chr10	89002890	89103291	100401	single	Variation_34898	chr10	88977696	89123516	145820	Kidd et al. (2008)	Paired End Mapping	
3726	chr10	89180154	89236450	56296	10	chr10	89180123	89237208	57085	single	Variation_6893	chr10	89174470	89199792	25322	de Smith et al. (2007)	Agilent 185k CGH Arrays/Agilent Custom CGH Arrays	
3727	chr10	89238128	89249737	11609	9	chr10	89238090	89250193	12103	HiConf	Variation_31773	chr10	89222875	89249436	26561	Perry et al. (2008)	Agilent Custom CGH Arrays	y
3728	chr10	89265978	89266402	424	87	chr10	89265971	89266412	441	HiConf	Variation_39773	chr10	89265777	89266548	771	Wheeler et al. (2008)	Sequencing	
3729	chr10	89608610	89609394	784	4	chr10	89608579	89609412	833	single								
3730	chr10	91988467	91991921	3454	20	chr10	91988460	91991988	3528	HiConf	Variation_38214	chr10	91988396	91992471	4075	McCarroll et al. (2008)	Affymetrix Human SNP Array 6.0	
3731	chr10	92451884	92456000	4116	18	chr10	92451853	92456067	4214	HiConf	Variation_5268	chr10	91930522	92537538	607016	Simon-Sanchez et al. (2007)	Illumina HumanHap300 BeadChip	y
3732	chr10	92914640	92924685	10045	10	chr10	92914609	92924899	10290	HiConf	Variation_38215	chr10	92915179	92923075	7896	McCarroll et al. (2008)	Affymetrix Human SNP Array 6.0	
3733	chr10	93223977	93245635	21658	1	chr10	93223946	93246094	22148	single								
3734	chr10	93623425	93624503	1078	44	chr10	93623394	93624521	1127	HiConf	Variation_43809	chr10	93623317	93624579	1262	Bentley et al. (2008)	Illumina DNA sequencing	
3735	chr10	94809519	94811675	2156	17	chr10	94809488	94811693	2205	single								y
3736	chr10	95535552	95536091	539	65	chr10	95535521	95536109	588	HiConf	Variation_43286	chr10	95535310	95536625	1315	Wang et al. (2008)	Illumina DNA sequencing	
3737	chr10	96861296	96862521	1225	46	chr10	96861265	96862539	1274	HiConf	Variation_10682	chr10	96862132	96862601	469	Conrad et al. (2005)	Mendelian inconsistencies	
3738	chr10	99825227	99827413	2186	2	chr10	99825226	99827431	2205	HiConf	Variation_38217	chr10	99824936	99827390	2454	McCarroll et al. (2008)	Affymetrix Human SNP Array 6.0	
3739	chr10	99847258	99847658	400	12	chr10	99847227	99847668	441	single	Variation_2887	chr10	99824786	99883943	59157	Redon et al. (2006)	Affymetrix 500K EA SNP Mapping Array	
3740	chr10	100366021	100368961	2940	2	chr10	100365990	100369028	3038	single	Variation_4710	chr10	100246424	100373804	127380	Wong et al. (2007)	BAC Array CGH	
3741	chr10	100678935	100692949	14014	11	chr10	100678904	100693212	14308	HiConf	Variation_34843	chr10	100673057	100695436	22379	Kidd et al. (2008)	Paired End Mapping	
3742	chr10	101412220	101437553	25333	2	chr10	101412189	101438061	25872	single								
3743	chr10	102344935	102351844	6909	4	chr10	102344904	102351960	7056	HiConf	Variation_38188	chr10	102345103	102352103	7000	McCarroll et al. (2008)	Affymetrix Human SNP Array 6.0	
100610						chr10	102434011	102492811	58800	single								
100611	chr10	102512407	102524790	12383	6	chr10	102503836	102525224	21388	single	Variation_3819	chr10	102368626	102555726	187100	Redon et al. (2006)	BAC Array CGH	
3745	chr10	103384176	103384666	490	43	chr10	103384145	103384684	539	HiConf	Variation_8666	chr10	103044972	103442635	397663	Pinto et al. (2007)	Affymetrix 500K SNP Mapping Array	
3746	chr10	104136032	104138052	2020	1	chr10	104136001	104141097	5096	single								
3747	chr10	104153412	104157174	3762	1	chr10	104147320	104157806	10486	HiConf								
3748						chr10	104162510	104173388	10878	single								
3749	chr10	104738732	104740496	1764	7	chr10	104738701	104740514	1813	single								
3750	chr10	104742750	104746572	3822	6	chr10	104742719	104746639	3920	single								
3751	chr10	106094562	106097796	3234	9	chr10	106094531	106097863	3332	HiConf								
3752	chr10	107047367	107056873	9506	37	chr10	107047336	107057038	9702	HiConf	Variation_22755	chr10	107045210	107052721	7511	Korbel et al. (2007)	Paired End Mapping	
3753	chr10	107243099	107243284	185	49	chr10	107243091	107243287	196	HiConf	Variation_44661	chr10	107242993	107243342	349	Bentley et al. (2008)	Illumina DNA sequencing	
3754	chr10	107940804	107941762	958	34	chr10	107940802	107942076	1274	HiConf	Variation_43233	chr10	107940631	107941705	1074	Wang et al. (2008)	Illumina DNA sequencing	
3755	chr10	109628736	109629569	833	4	chr10	109628705	109629587	882	single								
3756	chr10	109831008	109842950	11942	3	chr10	109830977	109843178	12201	HiConf								
3757	chr10	110249170	110253878	4708	1	chr10	110248604	110253994	5390	single								
3758	chr10	110292392	110303809	11417	8	chr10	110292361	110304023	11662	HiConf								y
3759	chr10	110622456	110623975	1519	4	chr10	110622425	110624875	2450	single	Variation_3821	chr10	110448977	110633486	184509	Redon et al. (2006)	BAC Array CGH	
3760	chr10	110906999	110909214	2215	2	chr10	110906968	110909222	2254	HiConf								
3761	chr10	112832601	112835002	2401	4	chr10	112832570	112835069	2499	single								
3762	chr10	112898653	112899878	1225	4	chr10	112898622	112899896	1274	single								
3763	chr10	112992782	113010569	17787	10	chr10	112992751	113010930	18179	HiConf								
3764	chr10	113129688	113132187	2499	3	chr10	113129657	113132254	2597	HiConf								
3765	chr10	113148553	113163351	14798	4	chr10	113148522	113163663	15141	single								y
3766	chr10	114101995	114107091	5096	48	chr10	114101964	114107207	5243	HiConf	Variation_38754	chr10	114102065	114106829	4764	McCarroll et al. (2008)	Affymetrix Human SNP Array 6.0	
3767	chr10	114136834	114139270	2436	2	chr10	114136803	114141164	4361	HiConf								
100614						chr10	114278438	114370043	91605	single								
3768	chr10	116531660	116534943	3283	20	chr10	116531629	116535010	3381	HiConf								
3769	chr10	117701682	117701889	207	70	chr10	117701651	117701896	245	HiConf	Variation_11504	chr10	117701607	117701992	385	Mills et al. (2006)	Sequence trace read mapping	
3770	chr10	118841373	118844950	3577	2	chr10	118841342	118845017	3675	single								
3771	chr10	120215284	120216264	980	6	chr10	120215253	120216282	1029	HiConf								
3772	chr10	120885947	120887074	1127	41	chr10	120885916	120887092	1176	HiConf								
3773	chr10	122216346	122218600	2254	49	chr10	122216315	122218618	2303	HiConf	Variation_22727	chr10	122214392	122221501	7109	Korbel et al. (2007)	Paired End Mapping	
100615	chr10	122754640	122786976	32336	28	chr10	122754556	122787631	33075	HiConf	Variation_29870	chr10	122759878	122779808	19930	Jakobsson et al. (2008)	Illumina HumanHap550 BeadChip	y
100616	chr10	124319813	124370522	50709	32	chr10	124319812	124370674	50862	HiConf	Variation_30519	chr10	124332451	124370524	38073	Perry et al. (2008)	Agilent Custom CGH Arrays	
3776	chr10	126059122	126063581	4459	2	chr10	126059091	126063648	4557	HiConf	Variation_38191	chr10	126060139	126064222	4083	McCarroll et al. (2008)	Affymetrix Human SNP Array 6.0	
3777	chr10	126185346	126186147	801	46	chr10	126185315	126186148	833	HiConf	Variation_24354	chr10	126185401	126185788	387	Levy et al. (2007)	Sequencing	

locus_id	chrom	start	end	length	Yoruba w/ event	putative chr	putative start	putative end	putative len	putative type	variation_id	DGV_chr	DGV_start	DGV_end	DGV_len	Reference	Method/platform	complex
3778	chr10	126610323	126616497	6174	6	chr10	126609263	126616613	7350	HiConf								
3779	chr10	127022658	127023295	637	6	chr10	127022627	127023313	686	single								
3780	chr10	127503446	127503653	207	45	chr10	127503415	127503660	245	HiConf	Variation_43724	chr10	127503397	127503677	280	Wang et al. (2008)	Illumina DNA sequencing	
100617	chr10	127566457	127605064	38607	5	chr10	127553910	127606070	52160	HiConf	Variation_31781	chr10	127562666	127598453	35787	Perry et al. (2008)	Agilent Custom CGH Arrays	
3782	chr10	128578947	128582083	3136	19	chr10	128578916	128582150	3234	HiConf	Variation_22563	chr10	128577403	128582702	5299	Korbel et al. (2007)	Paired End Mapping	
3783	chr10	128595509	128595572	63	18	chr10	128595478	128595576	98	single	Variation_44682	chr10	128595444	128595634	190	Bentley et al. (2008)	Illumina DNA sequencing	
3784	chr10	129184244	129187527	3283	15	chr10	129184213	129187594	3381	single								
100618	chr10	129463716	129509531	45815	12	chr10	129463685	129510455	46770	HiConf								
3786	chr10	132072226	132072656	430	28	chr10	132072224	132072665	441	single	Variation_41398	chr10	132072175	132072412	237	Wang et al. (2008)	Illumina DNA sequencing	
3787	chr10	132147715	132148115	400	28	chr10	132147684	132148125	441	single								
3788	chr10	132200831	132201958	1127	20	chr10	132200800	132201976	1176	single								
3789	chr10	132207985	132210925	2940	12	chr10	132207954	132210992	3038	HiConf	Variation_38192	chr10	132208544	132210099	1555	McCarroll et al. (2008)	Affymetrix Human SNP Array 6.0	
3790	chr10	132989094	132994239	5145	7	chr10	132989063	132994355	5292	HiConf								
3791	chr10	133023443	133033194	9751	2	chr10	133023412	133033408	9996	single								
3792	chr10	133033901	133036673	2772	23	chr10	133033555	133036740	3185	single								
3793	chr10	133037114	133041965	4851	14	chr10	133037083	133042081	4998	single								
3794	chr10	133494333	133515158	20825	10	chr10	133494302	133515568	21266	HiConf	Variation_38193	chr10	133497123	133508705	11582	McCarroll et al. (2008)	Affymetrix Human SNP Array 6.0	
3795	chr10	133779758	133780978	1220	8	chr10	133779727	133782814	3087	single	Variation_4717	chr10	133776459	133922089	145630	Wong et al. (2007)	BAC Array CGH	
3796	chr10	133854532	133855422	890	49	chr10	133854501	133855432	931	HiConf	Variation_24389	chr10	133855331	133855440	109	Levy et al. (2007)	Sequencing	
3797	chr10	134087338	134090614	3276	4	chr10	134086761	134090681	3920	single	Variation_29872	chr10	134063887	134140516	76629	Jakobsson et al. (2008)	Illumina HumanHap550 BeadChip	
3798	chr10	134440819	134442238	1419	2	chr10	134440100	134442256	2156	single	Variation_29874	chr10	134362510	134477872	115362	Jakobsson et al. (2008)	Illumina HumanHap550 BeadChip	
3799	chr10	134548078	134551606	3528	14	chr10	134548047	134551673	3626	single	Variation_9646	chr10	134544675	134641871	97196	Wang et al. (2007)	Illumina HumanHap550 BeadChip	
3800	chr10	134657397	134667148	9751	7	chr10	134657366	134667362	9996	single	Variation_3828	chr10	134515692	134683356	167664	Redon et al. (2006)	BAC Array CGH	
3801	chr10	134997555	134998094	539	32	chr10	134997524	134998112	588	single	Variation_30526	chr10	134995742	135016856	21114	Perry et al. (2008)	Agilent Custom CGH Arrays	
3802	chr10	135000936	135003239	2303	18	chr10	135000905	135003257	2352	HiConf	Variation_30526	chr10	134995742	135016856	21114	Perry et al. (2008)	Agilent Custom CGH Arrays	
3803	chr10	135067409	135068311	902	6	chr10	135067398	135068329	931	single	Variation_29601	chr10	135028601	135075655	47054	Jakobsson et al. (2008)	Illumina HumanHap550 BeadChip	y
3804	chr10	135088842	135098124	9282	64	chr10	135088811	135098170	9359	single	Variation_24423	chr10	135094763	135095447	684	Levy et al. (2007)	Sequencing	y
3805	chr10	135113440	135281678	168238	39	chr10	135099248	135284566	185318	HiConf	Variation_34564	chr10	135119945	135284541	164596	Zogopoulos et al. (2007)	Affymetrix 500K and 100K SNP Mapping Arrays	
3806	chr10	135284808	135372532	87724	27	chr10	135284762	135374285	89523	HiConf	Variation_37652	chr10	135250509	135356694	106185	McCarroll et al. (2008)	Affymetrix Human SNP Array 6.0	
3807	chr11	65881	155000	89119	9	chr11	65783	156923	91140	HiConf	Variation_31791	chr11	50126	89349	39223	Perry et al. (2008)	Agilent Custom CGH Arrays	
3808	chr11	163373	180425	17052	18	chr11	163342	180786	17444	HiConf								
3809	chr11	464919	465507	588	58	chr11	464888	465525	637	HiConf	Variation_29881	chr11	399815	557391	157576	Jakobsson et al. (2008)	Illumina HumanHap550 BeadChip	
3810	chr11	487165	487851	686	24	chr11	487134	487869	735	HiConf	Variation_29881	chr11	399815	557391	157576	Jakobsson et al. (2008)	Illumina HumanHap550 BeadChip	
3811	chr11	732802	734027	1225	8	chr11	732771	734045	1274	single	Variation_6940	chr11	732745	734077	1332	de Smith et al. (2007)	Agilent 185k CGH Arrays/Agilent Custom CGH Arrays	y
3812	chr11	1125194	1129432	4238	5	chr11	1125163	1133346	8183	single	Variation_29887	chr11	1017811	1270604	252793	Jakobsson et al. (2008)	Illumina HumanHap550 BeadChip	y
3813	chr11	1264158	1264942	784	29	chr11	1264127	1264960	833	HiConf	Variation_29888	chr11	1193004	1280140	87136	Jakobsson et al. (2008)	Illumina HumanHap550 BeadChip	
3814	chr11	1469223	1469623	400	17	chr11	1469192	1469633	441	single	Variation_4729	chr11	1341557	1478016	136459	Wong et al. (2007)	BAC Array CGH	
3815	chr11	1649690	1650523	833	25	chr11	1649659	1650541	882	single								
3816	chr11	1650964	1657068	6104	7	chr11	1650933	1657450	6517	single								
3817	chr11	1862595	1865192	2597	18	chr11	1862564	1865259	2695	HiConf	Variation_0410	chr11	1855962	1897796	41834	Tuzun et al. (2005)	Paired End Mapping	
3818	chr11	1872444	1881362	8918	9	chr11	1872413	1881527	9114	single	Variation_43677	chr11	1871796	1893599	21803	Wang et al. (2008)	Illumina DNA sequencing	
100621	chr11	2017656	2076430	58774	9	chr11	2017625	2077699	60074	single	Variation_6911	chr11	2021347	2048176	26829	de Smith et al. (2007)	Agilent 185k CGH Arrays/Agilent Custom CGH Arrays	
3820	chr11	2915703	2916585	882	37	chr11	2915672	2916603	931	single	Variation_4730	chr11	2846346	2941793	95447	Wong et al. (2007)	BAC Array CGH	
3821	chr11	3071474	3080490	9016	19	chr11	3071443	3080655	9212	HiConf	Variation_31795	chr11	3002762	3179993	177231	Perry et al. (2008)	Agilent Custom CGH Arrays	
3822	chr11	3187020	3191916	4896	13	chr11	3185221	3192032	6811	HiConf								
3823	chr11	3224893	3314040	89147	15	chr11	3224862	3315904	91042	HiConf	Variation_30535	chr11	3225514	3315778	90264	Perry et al. (2008)	Agilent Custom CGH Arrays	
3824	chr11	3362240	3579457	217217	10	chr11	3362209	3583885	221676	HiConf	Variation_2897	chr11	3376078	3569739	193661	Redon et al. (2006)	Affymetrix 500K EA SNP Mapping Array	
3825	chr11	3673390	3706954	33564	2	chr11	3673359	3707610	34251	single	Variation_3833	chr11	3193088	3758005	564917	Redon et al. (2006)	BAC Array CGH	
100623	chr11	4159928	4350154	190226	9	chr11	4159709	4354043	194334	HiConf	Variation_30537	chr11	4123217	4334702	211485	Perry et al. (2008)	Agilent Custom CGH Arrays	
3827	chr11	4499432	4500020	588	18	chr11	4499401	4500038	637	single	Variation_31801	chr11	4499106	4515720	16614	Perry et al. (2008)	Agilent Custom CGH Arrays	
3828	chr11	4800243	4800831	588	7	chr11	4800212	4800849	637	single								
3829	chr11	4923919	4936591	12672	58	chr11	4923888	4937118	13230	HiConf	Variation_31803	chr11	4924403	4935219	10816	Perry et al. (2008)	Agilent Custom CGH Arrays	
3830	chr11	4950869	4954593	3724	2	chr11	4950838	4954660	3822	HiConf	Variation_34971	chr11	4907535	4954345	46810	Kidd et al. (2008)	Paired End Mapping	
3831	chr11	5226053	5232615	6562	2	chr11	5226022	5232735	6713	HiConf	Variation_37787	chr11	5228251	5230232	1981	McCarroll et al. (2008)	Affymetrix Human SNP Array 6.0	
3832	chr11	5594239	5595170	931	22	chr11	5594208	5595188	980	HiConf	Variation_34458	chr11	5462340	6151051	688711	Zogopoulos et al. (2007)	Affymetrix 500K and 100K SNP Mapping Arrays	
3833	chr11	5716712	5718895	2183	50	chr11	5716708	5718913	2205	HiConf	Variation_43821	chr11	5716667	5718943	2276	Bentley et al. (2008)	Illumina DNA sequencing	
3834	chr11	5738593	5766233	27640	34	chr11	5738562	5766835	28273	HiConf	Variation_37479	chr11	5740206	5765860	25654	Cooper et al. (2008)	Illumina Human 1M BeadChip	
3835	chr11	5826450	5840513	14063	58	chr11	5826419	5840776	14357	HiConf	Variation_31805	chr11	5828206	5839952	11746	Perry et al. (2008)	Agilent Custom CGH Arrays	
3836	chr11	6146322	6149899	3577	10	chr11	6146291	6149966	3675	HiConf	Variation_34458	chr11	5462340	6151051	688711	Zogopoulos et al. (2007)	Affymetrix 500K and 100K SNP Mapping Arrays	
3837	chr11	7759066	7786856	27790	44	chr11	7758979	7799943	40964	HiConf	Variation_37481	chr11	7769976	7786612	16636	Cooper et al. (2008)	Illumina Human 1M BeadChip	
3838	chr11	7803173	7804205	1032	10	chr11	7802540	7804696	2156	HiConf	Variation_35017	chr11	7762902	7808602	45700	Kidd et al. (2008)	Paired End Mapping	
3839	chr11	9460781	9464758	3977	16	chr11	9460749	9465404	4655	HiConf	Variation_35030	chr11	9457471	9471326	13855	Kidd et al. (2008)	Paired End Mapping	
3840	chr11	9895312	9896145	833	15	chr11	9895281	9896163	882	HiConf	Variation_43835	chr11	9894965	9896312	1347	Bentley et al. (2008)	Illumina DNA sequencing	
3841	chr11	10198720	10201121	2401	7	chr11	10198689	10201139	2450	single	Variation_0143	chr11	10149870	10309473	159603	Iafrate et al. (2004)	BAC Array CGH	
3842	chr11	10249551	10249942	391	35	chr11	10249551	10249943	392	single	Variation_43812	chr11	10249323	10250421	1098	Bentley et al. (2008)	Illumina DNA sequencing	
3843	chr11	10486154	10488310	2156	49	chr11	10486123	10488328	2205	HiConf								
3844	chr11	10920819	10941321	20502	6	chr11	10920753	10941627	20874	HiConf	Variation_37663	chr11	10922331	10938872	16541	McCarroll et al. (2008)	Affymetrix Human SNP Array 6.0	
3845	chr11	11258247	11262020	3773	3	chr11	11258216	11262087	3871	HiConf	Variation_38220	chr11	11258385	11262056	3671	McCarroll et al. (2008)	Affymetrix Human SNP Array 6.0	
3846	chr11	11336423	11337431	1008	31	chr11	11336420	11337449	1029	HiConf	Variation_23396	chr11	11336197	11337606	1409	Levy et al. (2007)	Sequencing	
3847	chr11	11779558	11781469	1911	14	chr11	11779527	11781487	1960	HiConf	Variation_43399	chr11	11779461	11781411	1950	Wang et al. (2008)	Illumina DNA sequencing	
3848	chr11	11890102	11918277	28175	1	chr11	11890071	11918834	28763	single								
3849	chr11	12358395	12358795	400	24	chr11	12358364	12358805	441	HiConf	Variation_44823	chr11	12358305	12358924	619	Bentley et al. (2008)	Illumina DNA sequencing	
3850	chr11	12603885	12620839	16954	29	chr11	12603854	12621200	17346	HiConf								
3851	chr11	13585061	13586090	1029	11	chr11	13585030	13586108	1078	HiConf								
3852	chr11	15800057	15800219	162	1	chr11	15800026	15800222	196	single								
3853	chr11	17164119	17178118	13999	21	chr11	17164088	17178396	14308	HiConf								
3854	chr11	18045874	18057193	11319	3	chr11	18045843	18057407	11564	HiConf								
100626	chr11	18905666	18923636	17970	20	chr11	18894254	18924217	29963	HiConf	Variation_38835	chr11	18905648	18918564	12916	McCarroll et al. (2008)	Affymetrix Human SNP Array 6.0	
3856	chr11	19137790	19139799	2009	5	chr11	19137759	19139817	2058	HiConf								
3857	chr11	19448990	19451733	2743	2	chr11	19448958	19451800	2842	HiConf	Variation_2908	chr11	19439936	19450983	11047	Redon et al. (2006)	Affymetrix 500K EA SNP Mapping Array	
100627	chr11	21138351	21171731	33380	9	chr11	21138282	21172386	34104	HiConf	Variation_31806	chr11	21144962	21172404	27442	Perry et al. (2008)	Agilent Custom CGH Arrays	
3859	chr11	21189567	21196672	7105	7	chr11	21189536	21196788	7252	single	Variation_2909	chr11	21112669	21194273	81604	Redon et al. (2006)	Affymetrix 500K EA SNP Mapping Array	
3860	chr11	21297024	21298102	1078	9	chr11	21296993	21298120	1127	single								
3861	chr11	21379736	21381941	2205	3	chr11	21379705	21381959	2254	HiConf								
3862	chr11	21666484	21674128	7644	2	chr11	21666453	21675567	9114	single	Variation_37947	chr11	21671128	21679716	8588	McCarroll et al. (2008)	Affymetrix Human SNP Array 6.0	
100628	chr11	21755248	21799078	43830	2	chr11	21727458	21800542	73084	HiConf	Variation_2910	chr11	21740206	21805311	65105	Redon et al. (2006)	Affymetrix 500K EA SNP Mapping Array	

locus_id	chrom	start	end	length	Yoruba w/ event	putative chr	putative start	putative end	putative len	putative type	variation_id	DGV_chr	DGV_start	DGV_end	DGV_len	Reference	Method/platform	complex
3864	chr11	21803929	21805938	2009	20	chr11	21803898	21805956	2058	HiConf	Variation_31808	chr11	21803918	21805682	1764	Perry et al. (2008)	Agilent Custom CGH Arrays	
3865	chr11	22206807	22211217	4410	2	chr11	22206776	22211284	4508	single								
100629	chr11	22589497	22615740	26243	3	chr11	22589466	22655886	66420	single								
3866	chr11	23407503	23418136	10633	3	chr11	23407472	23418350	10878	single								
3867	chr11	23706501	23709588	3087	29	chr11	23706470	23709655	3185	HiConf								
100630	chr11	23908253	23921828	13575	5	chr11	23904602	23949853	45251	single								
3868	chr11	24240944	24244570	3626	40	chr11	24240913	24244637	3724	HiConf								
3869	chr11	24615010	24620743	5733	10	chr11	24614979	24620859	5880	HiConf	Variation_22623	chr11	24614861	24620959	6098	Korbel et al. (2007)	Paired End Mapping	
3870	chr11	24859667	24860451	784	12	chr11	24859636	24860469	833	single	Variation_47942	chr11	24787750	25047350	259600	Gusev et al. (2009)	SNP genotyping analysis	
100631	chr11	25077840	25254044	176204	7	chr11	25077809	25257639	179830	HiConf	Variation_38224	chr11	25090419	25250126	159707	McCarroll et al. (2008)	Affymetrix Human SNP Array 6.0	
3874	chr11	25333812	25334291	479	11	chr11	25333809	25334299	490	single	Variation_11547	chr11	25333781	25334449	668	Mills et al. (2006)	Sequence trace read mapping	
3875	chr11	25389406	25390092	686	4	chr11	25389375	25390110	735	single	Variation_38225	chr11	25384338	25390482	6144	McCarroll et al. (2008)	Affymetrix Human SNP Array 6.0	
100632	chr11	25556004	25583002	26998	8	chr11	25555926	25586012	30086	HiConf	Variation_31809	chr11	25563651	25581052	17401	Perry et al. (2008)	Agilent Custom CGH Arrays	
3877	chr11	25658908	25677428	18520	16	chr11	25658875	25677789	18914	HiConf	Variation_43817	chr11	25658848	25677487	18639	Bentley et al. (2008)	Illumina DNA sequencing	
3878						chr11	26061116	26061165	49	single								
3879	chr11	28963784	28968856	5072	42	chr11	28963729	28968972	5243	HiConf	Variation_43818	chr11	28963777	28969472	5695	Bentley et al. (2008)	Illumina DNA sequencing	
3880	chr11	29096060	29100225	4165	39	chr11	29096029	29100292	4263	HiConf	Variation_43288	chr11	29095733	29097058	1325	Wang et al. (2008)	Illumina DNA sequencing	
3881	chr11	29768538	29782256	13718	2	chr11	29764536	29782617	18081	HiConf								
3882	chr11	29924258	29924846	588	78	chr11	29924227	29924864	637	HiConf	Variation_43216	chr11	29924108	29925114	1006	Wang et al. (2008)	Illumina DNA sequencing	
3883	chr11	30128490	30133341	4851	10	chr11	30128459	30133457	4998	HiConf	Variation_31812	chr11	30129364	30133432	4068	Perry et al. (2008)	Agilent Custom CGH Arrays	
3884	chr11	31350452	31353980	3528	14	chr11	31350421	31354047	3626	HiConf	Variation_34958	chr11	31337783	31359097	21314	Kidd et al. (2008)	Paired End Mapping	
3885	chr11	31923850	31925271	1421	38	chr11	31923819	31925289	1470	HiConf	Variation_5622	chr11	31923749	31925874	2125	Mills et al. (2006)	Sequence trace read mapping	
100633	chr11	32987395	33022138	34743	2	chr11	32987364	33022840	35476	single	Variation_3843	chr11	32671219	33006516	335297	Redon et al. (2006)	BAC Array CGH	
3886	chr11	34891045	34925002	33957	3	chr11	34891014	34925706	34692	single	Variation_2917	chr11	34869891	34920304	50413	Redon et al. (2006)	Affymetrix 500K EA SNP Mapping Array	
100634	chr11	35024541	35193260	168719	2	chr11	35024490	35196652	172162	single	Variation_2918	chr11	35024058	35195154	171096	Redon et al. (2006)	Affymetrix 500K EA SNP Mapping Array	
100635	chr11	36252412	36340220	87808	17	chr11	36252381	36342027	89646	HiConf	Variation_11050	chr11	36299628	36300413	785	de Smith et al. (2007)	Agilent 185k CGH Arrays/Agilent Custom CGH Arrays	
3890	chr11	36604134	36611288	7154	24	chr11	36604103	36611404	7301	HiConf	Variation_5274	chr11	35906274	36945491	1039217	Simon-Sanchez et al. (2007)	Illumina HumanHap300 BeadChip	
3891	chr11	37167536	37167936	400	5	chr11	37167505	37167946	441	single								
3892	chr11	37537878	37537986	108	53	chr11	37537847	37537994	147	single								
3893	chr11	37983582	37985836	2254	6	chr11	37983551	37985854	2303	HiConf								
3894	chr11	38243527	38282678	39151	5	chr11	38243496	38283480	39984	single	Variation_29604	chr11	38242077	38282959	40882	Jakobsson et al. (2008)	Illumina HumanHap550 BeadChip	
3895	chr11	38413998	38420368	6370	6	chr11	38413967	38420484	6517	HiConf	Variation_38230	chr11	38414524	38419763	5239	McCarroll et al. (2008)	Affymetrix Human SNP Array 6.0	
3896	chr11	38900071	38917081	17010	2	chr11	38893236	38917540	24304	HiConf								
100636	chr11	39923149	39956420	33271	3	chr11	39923118	39957075	33957	single								
3897	chr11	41293924	41304557	10633	5	chr11	41293893	41304771	10878	HiConf	Variation_37667	chr11	41295844	41303860	8016	McCarroll et al. (2008)	Affymetrix Human SNP Array 6.0	y
3898	chr11	41774614	41775295	681	57	chr11	41774583	41775318	735	HiConf	Variation_39830	chr11	41774774	41775600	826	Wheeler et al. (2008)	Sequencing	
3899	chr11	41903876	41904464	588	6	chr11	41903845	41904482	637	single								
3900	chr11	42630889	42635789	4900	5	chr11	42630858	42635905	5047	HiConf								
3901	chr11	42770784	42775299	4515	35	chr11	42770753	42777221	6468	HiConf	Variation_37483	chr11	42770873	42774780	3907	Cooper et al. (2008)	Illumina Human 1M BeadChip	
3902	chr11	42925085	42929887	4802	4	chr11	42925054	42929954	4900	HiConf	Variation_9656	chr11	42925451	42929829	4378	Wang et al. (2007)	Illumina HumanHap550 BeadChip	
3903	chr11	43124123	43124383	260	19	chr11	43124092	43124386	294	single								
3904	chr11	43320662	43322573	1911	3	chr11	43320631	43322591	1960	single								
3905	chr11	43501227	43506029	4802	6	chr11	43501196	43506096	4900	single								
100637						chr11	44900734	44942262	41528	single								
3906	chr11	45376800	45377200	400	21	chr11	45376769	45377210	441	single								
3907	chr11	45386502	45387923	1421	29	chr11	45386471	45387941	1470	HiConf								
3908	chr11	45677367	45692458	15091	8	chr11	45677335	45692770	15435	HiConf								
3909	chr11	45693368	45695594	2226	3	chr11	45692966	45695661	2695	HiConf								
100638	chr11	45860861	45872068	11207	1	chr11	45848566	45899697	51131	HiConf								
3911	chr11	47335085	47336310	1225	6	chr11	47335054	47336328	1274	single								
3912	chr11	48284536	48285538	1002	4	chr11	48284527	48285556	1029	single	Variation_30549	chr11	48284285	48285336	1051	Perry et al. (2008)	Agilent Custom CGH Arrays	
3913	chr11	48557537	48560673	3136	36	chr11	48557506	48560740	3234	HiConf	Variation_43820	chr11	48557434	48560857	3423	Bentley et al. (2008)	Illumina DNA sequencing	
100639	chr11	49007586	49076031	68445	5	chr11	49007547	49077421	69874	single	Variation_31819	chr11	49038687	49054705	16018	Perry et al. (2008)	Agilent Custom CGH Arrays	
100640	chr11	49261226	49324877	63651	4	chr11	49261195	49326194	64999	HiConf	Variation_38234	chr11	49264380	49317267	52887	McCarroll et al. (2008)	Affymetrix Human SNP Array 6.0	
3916	chr11	49450856	49461048	10192	3	chr11	49450825	49461262	10437	HiConf								
100641	chr11	49662683	49716542	53859	2	chr11	49662652	49731718	69066	HiConf	Variation_38235	chr11	49667437	49714078	46641	McCarroll et al. (2008)	Affymetrix Human SNP Array 6.0	
100642	chr11	49779610	49879139	99529	4	chr11	49779517	49881119	101602	HiConf	Variation_1889	chr11	49805757	49811283	5526	McCarroll et al. (2005)	Null genotypes	
100643	chr11	50174295	50257684	83389	3	chr11	50162624	50259668	97044	single	Variation_9659	chr11	50205110	50249096	43986	Wang et al. (2007)	Illumina HumanHap550 BeadChip	
3919	chr11	51199764	51223137	23373	11	chr11	51199733	51223596	23863	HiConf	Variation_43678	chr11	51197691	51219710	22019	Wang et al. (2008)	Illumina DNA sequencing	y
3920	chr11	55122283	55266538	144255	35	chr11	55119390	55267762	148372	HiConf	Variation_0419	chr11	55115601	55226935	111334	Tuzun et al. (2005)	Paired End Mapping	
3921	chr11	55267964	55268730	766	9	chr11	55267958	55268742	784	single	Variation_38236	chr11	55217259	55303668	86409	McCarroll et al. (2008)	Affymetrix Human SNP Array 6.0	
3922	chr11	55268890	55270915	2025	7	chr11	55268889	55270947	2058	single	Variation_38236	chr11	55217259	55303668	86409	McCarroll et al. (2008)	Affymetrix Human SNP Array 6.0	
3923	chr11	55277733	55279489	1756	7	chr11	55271486	55279669	8183	single	Variation_38236	chr11	55217259	55303668	86409	McCarroll et al. (2008)	Affymetrix Human SNP Array 6.0	
3924	chr11	55279817	55315372	35555	8	chr11	55279816	55316076	36260	single	Variation_1244	chr11	55231932	55320476	88544	Conrad et al. (2005)	Mendelian inconsistencies	
3925	chr11	55316646	55359374	42728	7	chr11	55316615	55360225	43610	HiConf	Variation_5629	chr11	55347300	55357168	9868	Mills et al. (2006)	Sequence trace read mapping	
100645	chr11	55439685	55590066	150381	2	chr11	55439654	55593122	153468	HiConf	Variation_9189	chr11	55442375	55599088	156713	Pinto et al. (2007)	Affymetrix 500K SNP Mapping Array	y
3927	chr11	55786654	55804578	17924	44	chr11	55786623	55804802	18179	single								
3928	chr11	56829229	56831605	2376	2	chr11	56825766	56832381	6615	single								
3929						chr11	56973109	56973158	49	single								
3930	chr11	57516147	57517775	1628	6	chr11	57510492	57517940	7448	HiConf	Variation_34988	chr11	57508225	57529794	21569	Kidd et al. (2008)	Paired End Mapping	
3931	chr11	57609601	57611512	1911	15	chr11	57609570	57611530	1960	single	Variation_38237	chr11	57608407	57612191	3784	McCarroll et al. (2008)	Affymetrix Human SNP Array 6.0	
3932	chr11	58214482	58218230	3748	45	chr11	58214475	58218297	3822	HiConf	Variation_37485	chr11	58214361	58218268	3907	Cooper et al. (2008)	Illumina Human 1M BeadChip	
100646	chr11	58545880	58634939	89059	7	chr11	58534788	58636341	101553	HiConf	Variation_34460	chr11	58560700	58610700	50000	Zogopoulos et al. (2007)	Affymetrix 500K and 100K SNP Mapping Arrays	
100647	chr11	59068037	59232286	164249	2	chr11	59068006	59235684	167678	HiConf	Variation_4746	chr11	59152983	59187691	34708	Wong et al. (2007)	BAC Array CGH	
3934						chr11	59523265	59525715	2450	single								
100648	chr11	59954594	60178475	223881	4	chr11	59954563	60183050	228487	HiConf								
100649	chr11	60700278	60823707	123429	26	chr11	60700245	60826200	125955	HiConf	Variation_3854	chr11	60671936	60837233	165297	Redon et al. (2006)	BAC Array CGH	
100650	chr11	61061729	61066662	4933	2	chr11	61061547	61112360	50813	HiConf								
100651	chr11	62018115	62024956	6841	1	chr11	62012882	62056247	43365	HiConf								
3943						chr11	62566900	62580473	13573	single								
3944	chr11	62593930	62595888	1958	10	chr11	62593899	62596251	2352	HiConf	Variation_10363	chr11	62594645	62596243	1598	Wang et al. (2007)	Illumina HumanHap550 BeadChip	
3945	chr11	62796643	62797721	1078	10	chr11	62796612	62797739	1127	HiConf								
3946	chr11	62949278	62950797	1519	14	chr11	62949247	62950815	1568	single	Variation_0421	chr11	62936696	62949999	13303	Tuzun et al. (2005)	Paired End Mapping	
100652						chr11	64121964	64320463	198499	HiConf								
3948	chr11	65330580	65336313	5733	2	chr11	65330549	65336429	5880	single	Variation_3855	chr11	65316469	65478939	162470	Redon et al. (2006)	BAC Array CGH	
3949	chr11	65660546	65661183	637	7	chr11	65660515	65661201	686	single								

locus_id	chrom	start	end	length	Yoruba w/ event	putative chr	putative start	putative end	putative len	putative type	variation_id	DGV_chr	DGV_start	DGV_end	DGV_len	Reference	Method/platform	complex
3950	chr11	65690534	65695973	5439	20	chr11	65690503	65696089	5586	HiConf	Variation_37486	chr11	65690430	65695863	5433	Cooper et al. (2008)	Illumina Human 1M BeadChip	
3951	chr11	66468820	66469732	912	42	chr11	66468819	66469750	931	HiConf	Variation_11058	chr11	66468753	66469671	918	de Smith et al. (2007)	Agilent 185k CGH Arrays/Agilent Custom CGH Arrays	
100653	chr11	67204795	67522537	317742	2	chr11	67204677	67529032	324355	HiConf	Variation_10366	chr11	67258202	67505393	247191	Wang et al. (2007)	Illumina HumanHap550 BeadChip	
3953	chr11	67913027	67913762	735	37	chr11	67912996	67913780	784	HiConf								
3954	chr11	67998630	68005637	7007	5	chr11	67998599	68005753	7154	single								
3955	chr11	68177627	68217283	39656	11	chr11	68177596	68217776	40180	HiConf								
3956	chr11	68606524	68607014	490	34	chr11	68606493	68607032	539	single	Variation_29915	chr11	68561027	68656390	95363	Jakobsson et al. (2008)	Illumina HumanHap550 BeadChip	
3957	chr11	68803357	68804092	735	27	chr11	68803326	68804110	784	single								
3958	chr11	69143515	69149591	6076	4	chr11	69143484	69149707	6223	single	Variation_4755	chr11	69132864	69297659	164795	Wong et al. (2007)	BAC Array CGH	y
3959	chr11	69202560	69205893	3333	14	chr11	69202529	69205959	3430	single	Variation_4755	chr11	69132864	69297659	164795	Wong et al. (2007)	BAC Array CGH	
3960	chr11	69752536	69760964	8428	1	chr11	69752505	69761129	8624	single	Variation_37487	chr11	69749776	69761724	11948	Cooper et al. (2008)	Illumina Human 1M BeadChip	
3961	chr11	70776097	70776881	784	31	chr11	70776066	70776899	833	single	Variation_4756	chr11	70752897	70809016	56119	Wong et al. (2007)	BAC Array CGH	
100654	chr11	70970064	71291602	321538	8	chr11	70970033	71298137	328104	HiConf	Variation_3857	chr11	71041287	71335224	293937	Redon et al. (2006)	BAC Array CGH	
100655						chr11	71972818	72131406	158588	HiConf								
100656	chr11	72734247	72769982	35735	2	chr11	72734106	72770905	36799	HiConf								
3964	chr11	72985458	72986242	784	13	chr11	72985427	72986260	833	single								
3965	chr11	73064593	73066014	1421	9	chr11	73064562	73066032	1470	single								
3966	chr11	73073609	73075520	1911	1	chr11	73073578	73075538	1960	single								
3967	chr11	73468696	73469186	490	3	chr11	73468665	73469204	539	single								
100657	chr11	73676285	73798442	122157	4	chr11	73676254	73800934	124680	HiConf	Variation_43482	chr11	73702527	73705237	2710	Wang et al. (2008)	Illumina DNA sequencing	
3969	chr11	74108230	74108655	425	6	chr11	74107674	74108948	1274	single	Variation_8692	chr11	73556336	74211100	654764	Pinto et al. (2007)	Affymetrix 500K SNP Mapping Array	
3970	chr11	75144202	75145378	1176	8	chr11	75144171	75145396	1225	single								
3971	chr11	75529930	75531155	1225	2	chr11	75529899	75531173	1274	single								
3972	chr11	75644880	75656644	11764	2	chr11	75644363	75656907	12544	single								
3973	chr11	75817854	75826184	8330	17	chr11	75817823	75826349	8526	HiConf	Variation_38239	chr11	75820665	75825933	5268	McCarroll et al. (2008)	Affymetrix Human SNP Array 6.0	
3974	chr11	76932653	76932860	207	15	chr11	76932622	76932867	245	single								
100658	chr11	77285631	77346311	60680	2	chr11	77273760	77347799	74039	HiConf	Variation_31830	chr11	77328780	77345509	16729	Perry et al. (2008)	Agilent Custom CGH Arrays	
3976	chr11	79020445	79026570	6125	1	chr11	79020414	79026686	6272	single								
3977	chr11	79068034	79073316	5282	5	chr11	79066474	79073432	6958	HiConf								
3978	chr11	79648037	79662443	14406	9	chr11	79648006	79662706	14700	HiConf	Variation_37861	chr11	79650579	79659825	9246	McCarroll et al. (2008)	Affymetrix Human SNP Array 6.0	
100659						chr11	79663368	79727190	63822	single								
3979	chr11	80549294	80556693	7399	3	chr11	80549263	80556858	7595	HiConf	Variation_38240	chr11	80550138	80556840	6702	McCarroll et al. (2008)	Affymetrix Human SNP Array 6.0	
3980	chr11	80576587	80583300	6713	4	chr11	80576556	80583416	6860	HiConf	Variation_37669	chr11	80575854	80583265	7411	McCarroll et al. (2008)	Affymetrix Human SNP Array 6.0	
3981	chr11	80639454	80640826	1372	3	chr11	80639423	80640844	1421	single								
3982	chr11	80661063	80694764	33701	3	chr11	80661032	80695479	34447	HiConf	Variation_29916	chr11	80661868	80692622	30754	Jakobsson et al. (2008)	Illumina HumanHap550 BeadChip	
3983	chr11	80835062	80836385	1323	3	chr11	80835031	80836403	1372	single								
3984	chr11	80865687	80866961	1274	17	chr11	80865656	80866979	1323	HiConf								
3985	chr11	80910081	80916598	6517	6	chr11	80910050	80916714	6664	single								
3986	chr11	80944332	80945851	1519	4	chr11	80944301	80945869	1568	HiConf								
3987	chr11	81178895	81194575	15680	11	chr11	81178864	81194887	16023	HiConf	Variation_10372	chr11	81181640	81194909	13269	Wang et al. (2007)	Illumina HumanHap550 BeadChip	
3988	chr11	81353385	81354413	1028	13	chr11	81353353	81354431	1078	HiConf	Variation_33545	chr11	81353672	81353989	317	Perry et al. (2008)	Agilent Custom CGH Arrays	
3989	chr11	81533557	81540760	7203	5	chr11	81533526	81540876	7350	HiConf	Variation_35023	chr11	81519610	81551863	32253	Kidd et al. (2008)	Paired End Mapping	
3990	chr11	82138658	82140814	2156	3	chr11	82138627	82140832	2205	HiConf	Variation_43827	chr11	82138208	82140953	2745	Bentley et al. (2008)	Illumina DNA sequencing	y
3991	chr11	82800893	82813339	12446	26	chr11	82800862	82813602	12740	HiConf	Variation_6945	chr11	81909575	83194350	1284775	de Smith et al. (2007)	Agilent 185k CGH Arrays/Agilent Custom CGH Arrays	
3992	chr11	83142668	83145412	2744	19	chr11	83142637	83145479	2842	HiConf	Variation_43828	chr11	83142305	83145561	3256	Bentley et al. (2008)	Illumina DNA sequencing	
3993	chr11	83210552	83216462	5910	2	chr11	83210502	83216578	6076	single								
3994	chr11	84733208	84737961	4753	1	chr11	84733177	84738028	4851	single								
3995	chr11	84795781	84800926	5145	8	chr11	84795750	84801042	5292	single								
3996	chr11	85024807	85025177	370	4	chr11	85024776	85025217	441	single	Variation_3860	chr11	84858128	85281636	423508	Redon et al. (2006)	BAC Array CGH	
3997	chr11	85332162	85332762	600	1	chr11	85330781	85333329	2548	single								
3998	chr11	85963647	85964235	588	44	chr11	85963616	85964253	637	HiConf	Variation_10871	chr11	85963510	85964435	925	Hinds et al. (2005)	Oligo arrays	
3999	chr11	85981924	85984325	2401	16	chr11	85981893	85984392	2499	HiConf	Variation_43459	chr11	85981842	85984292	2450	Wang et al. (2008)	Illumina DNA sequencing	
4000	chr11	86010344	86011569	1225	3	chr11	86010313	86011587	1274	HiConf								
4001	chr11	86431450	86434194	2744	3	chr11	86431419	86434261	2842	HiConf								
100661	chr11	87292681	87306520	13839	1	chr11	87249156	87348038	98882	HiConf	Variation_2942	chr11	87287865	87304787	16922	Redon et al. (2006)	Affymetrix 500K EA SNP Mapping Array	
4003	chr11	87562174	87562484	310	44	chr11	87562143	87562486	343	HiConf								
100662						chr11	88250887	88306600	55713	single								
4004	chr11	88605041	88607099	2058	36	chr11	88605010	88607117	2107	HiConf	Variation_43540	chr11	88604103	88607660	3557	Wang et al. (2008)	Illumina DNA sequencing	
4005	chr11	89104596	89107830	3234	7	chr11	89104565	89107897	3332	single	Variation_2943	chr11	89105700	89525014	419314	Redon et al. (2006)	Affymetrix 500K EA SNP Mapping Array	
4006	chr11	89129194	89145266	16072	16	chr11	89129163	89145578	16415	HiConf	Variation_31837	chr11	89116348	89154457	38109	Perry et al. (2008)	Agilent Custom CGH Arrays	
4007	chr11	89185005	89189366	4361	41	chr11	89184974	89189433	4459	HiConf	Variation_30563	chr11	89185554	89189177	3623	Perry et al. (2008)	Agilent Custom CGH Arrays	
4008	chr11	89249146	89257476	8330	11	chr11	89249115	89257641	8526	HiConf	Variation_31838	chr11	89215176	89374958	159782	Perry et al. (2008)	Agilent Custom CGH Arrays	
4009	chr11	89260416	89265855	5439	26	chr11	89260385	89265971	5586	HiConf	Variation_31838	chr11	89215176	89374958	159782	Perry et al. (2008)	Agilent Custom CGH Arrays	
4010	chr11	89324165	89329751	5586	27	chr11	89324134	89329867	5733	HiConf	Variation_30564	chr11	89323665	89327396	3731	Perry et al. (2008)	Agilent Custom CGH Arrays	
4011	chr11	89333132	89340972	7840	13	chr11	89333101	89341137	8036	HiConf	Variation_31838	chr11	89215176	89374958	159782	Perry et al. (2008)	Agilent Custom CGH Arrays	
4012	chr11	89423733	89431769	8036	9	chr11	89423702	89431934	8232	single	Variation_31839	chr11	89427786	89482222	54436	Perry et al. (2008)	Agilent Custom CGH Arrays	
4013	chr11	89439462	89449360	9898	17	chr11	89439431	89449574	10143	HiConf	Variation_31839	chr11	89427786	89482222	54436	Perry et al. (2008)	Agilent Custom CGH Arrays	
4014	chr11	89475036	89478123	3087	2	chr11	89475005	89478190	3185	single	Variation_31839	chr11	89427786	89482222	54436	Perry et al. (2008)	Agilent Custom CGH Arrays	
4015	chr11	89491108	89493362	2254	7	chr11	89491077	89493380	2303	HiConf	Variation_2943	chr11	89105700	89525014	419314	Redon et al. (2006)	Affymetrix 500K EA SNP Mapping Array	
100664	chr11	89526976	89593224	66248	1	chr11	89526945	89594565	67620	single	Variation_42370	chr11	89572086	89572560	474	Wang et al. (2008)	Illumina DNA sequencing	
4016	chr11	89833471	89835774	2303	16	chr11	89833440	89835792	2352	HiConf	Variation_43461	chr11	89833278	89835759	2481	Wang et al. (2008)	Illumina DNA sequencing	
4017						chr11	89989309	89989554	245	single								
4018	chr11	90207782	90210624	2842	63	chr11	90207751	90210691	2940	HiConf	Variation_38764	chr11	90207835	90210585	2750	McCarroll et al. (2008)	Affymetrix Human SNP Array 6.0	
4019	chr11	91010549	91011137	588	22	chr11	91010518	91011155	637	HiConf								
4020	chr11	91428176	91428284	108	6	chr11	91428145	91428292	147	single								
4021	chr11	92344280	92346730	2450	4	chr11	92344249	92346797	2548	single	Variation_37670	chr11	92344805	92346836	2031	McCarroll et al. (2008)	Affymetrix Human SNP Array 6.0	y
4022	chr11	92660330	92662868	2538	58	chr11	92660299	92662896	2597	HiConf	Variation_43831	chr11	92660784	92661808	1024	Bentley et al. (2008)	Illumina DNA sequencing	
4023	chr11	92792140	92792540	400	5	chr11	92792109	92792550	441	HiConf	Variation_22784	chr11	92792265	92800397	8132	Korbel et al. (2007)	Paired End Mapping	
4024	chr11	93317322	93317812	490	11	chr11	93317291	93317830	539	single	Variation_2947	chr11	93315872	93354598	38726	Redon et al. (2006)	Affymetrix 500K EA SNP Mapping Array	
4025	chr11	93323007	93328684	5677	18	chr11	93322975	93328708	5733	HiConf	Variation_39083	chr11	93322976	93328212	5236	Wheeler et al. (2008)	Sequencing	
4026	chr11	93330209	93330748	539	20	chr11	93330178	93330766	588	single	Variation_2947	chr11	93315872	93354598	38726	Redon et al. (2006)	Affymetrix 500K EA SNP Mapping Array	
4027	chr11	93334962	93341577	6615	18	chr11	93334931	93341693	6762	HiConf	Variation_31843	chr11	93334923	93341681	6758	Perry et al. (2008)	Agilent Custom CGH Arrays	
4028	chr11	93541056	93550219	9163	9	chr11	93541025	93550384	9359	HiConf								
100665	chr11	93866661	93913064	46403	1	chr11	93866630	93913989	47359	single								
4029						chr11	94674052	94682137	8085	HiConf								
4030	chr11	94764880	94769633	4753	4	chr11	94764849	94769700	4851	single								

locus_id	chrom	start	end	length	Yoruba w/ event	putative chr	putative start	putative end	putative len	putative type	variation_id	DGV_chr	DGV_start	DGV_end	DGV_len	Reference	Method/platform	complex
4031	chr11	95471999	95472449	450	4	chr11	95471968	95472458	490	HiConf	Variation_5424	chr11	95454157	95472813	18656	Simon-Sanchez et al. (2007)	Illumina HumanHap300 BeadChip	
4032	chr11	95641802	95642862	1060	56	chr11	95641802	95642880	1078	HiConf	Variation_5641	chr11	95641573	95643042	1469	Mills et al. (2006)	Sequence trace read mapping	
4033	chr11	96253304	96258057	4753	10	chr11	96253273	96258124	4851	HiConf								
100666	chr11	96480403	96554918	74515	1	chr11	96472916	96566163	93247	single	Variation_9195	chr11	96479400	96549641	70241	Pinto et al. (2007)	Affymetrix 500K SNP Mapping Array	
4035	chr11	97023241	97027259	4018	26	chr11	97023210	97027326	4116	HiConf	Variation_22564	chr11	97022422	97027735	5313	Korbel et al. (2007)	Paired End Mapping	
4036	chr11	97978986	97981436	2450	51	chr11	97978955	97981503	2548	HiConf								
4037	chr11	98389753	98412501	22748	6	chr11	98389722	98415594	25872	single								
100667	chr11	98607215	98677971	70756	1	chr11	98607184	98679386	72202	single	Variation_31844	chr11	98617823	98623108	5285	Perry et al. (2008)	Agilent Custom CGH Arrays	
4038	chr11	100882062	100888704	6642	2	chr11	100882009	100888820	6811	single								
4039	chr11	100950003	100950591	588	25	chr11	100949972	100950609	637	HiConf								
4040	chr11	101394041	101399926	5885	2	chr11	101394010	101400037	6027	HiConf								
4041						chr11	101643763	101648124	4361	single								
100668	chr11	102096236	102116816	20580	11	chr11	102096205	102120754	24549	single								
4043	chr11	102256735	102257911	1176	72	chr11	102256704	102257929	1225	HiConf								
4044	chr11	102464985	102500073	35088	4	chr11	102464954	102500822	35868	single	Variation_0155	chr11	102438737	102497333	58596	Iafrate et al. (2004)	BAC Array CGH	
4045	chr11	103357177	103361538	4361	7	chr11	103357146	103361605	4459	single	Variation_8694	chr11	102587768	103430000	842232	Pinto et al. (2007)	Affymetrix 500K SNP Mapping Array	
4046	chr11	103421759	103422543	784	22	chr11	103421728	103422561	833	HiConf	Variation_8694	chr11	102587768	103430000	842232	Pinto et al. (2007)	Affymetrix 500K SNP Mapping Array	y
100669	chr11	103533948	103589078	55130	6	chr11	103513530	103590558	77028	HiConf	Variation_8695	chr11	103470000	104851268	1381268	Pinto et al. (2007)	Affymetrix 500K SNP Mapping Array	
4048	chr11	103764416	103769022	4606	4	chr11	103764385	103769089	4704	single	Variation_34906	chr11	103768103	103781179	13076	Kidd et al. (2008)	Paired End Mapping	y
4049	chr11	103772893	103778740	5847	45	chr11	103772862	103778742	5880	HiConf	Variation_22591	chr11	103772864	103778661	5797	Korbel et al. (2007)	Paired End Mapping	y
4050	chr11	103822040	103826401	4361	18	chr11	103822009	103826468	4459	HiConf	Variation_31845	chr11	103822422	103825876	3454	Perry et al. (2008)	Agilent Custom CGH Arrays	
100670	chr11	104233766	104264066	30300	1	chr11	104228685	104279204	50519	single	Variation_31846	chr11	104230355	104264796	34441	Perry et al. (2008)	Agilent Custom CGH Arrays	
4052	chr11	104428660	104436402	7742	21	chr11	104428629	104436567	7938	HiConf	Variation_37906	chr11	104429072	104436443	7371	McCarroll et al. (2008)	Affymetrix Human SNP Array 6.0	
4053	chr11	104447917	104478395	30478	3	chr11	104447886	104479001	31115	HiConf	Variation_30566	chr11	104447500	104477533	30033	Perry et al. (2008)	Agilent Custom CGH Arrays	
4054	chr11	104554516	104574190	19674	8	chr11	104554510	104574600	20090	HiConf	Variation_38218	chr11	104555015	104572108	17093	McCarroll et al. (2008)	Affymetrix Human SNP Array 6.0	
4055	chr11	104798585	104804245	5660	36	chr11	104798579	104804361	5782	HiConf	Variation_22607	chr11	104798500	104804436	5936	Korbel et al. (2007)	Paired End Mapping	
4056	chr11	104918611	104921698	3087	3	chr11	104918580	104921765	3185	HiConf								
4057	chr11	106291591	106294678	3087	8	chr11	106291560	106294745	3185	HiConf	Variation_34909	chr11	106262230	106300356	38126	Kidd et al. (2008)	Paired End Mapping	
4058	chr11	106489110	106507632	18522	8	chr11	106489079	106507993	18914	HiConf								
4059	chr11	106744158	106757238	13080	47	chr11	106735843	106757648	21805	HiConf	Variation_37488	chr11	106743994	106748586	4592	Cooper et al. (2008)	Illumina Human 1M BeadChip	
4060	chr11	106842269	106853180	10911	5	chr11	106842173	106853394	11221	HiConf	Variation_38219	chr11	106843163	106852442	9279	McCarroll et al. (2008)	Affymetrix Human SNP Array 6.0	
4061	chr11	107287712	107293420	5708	20	chr11	107287681	107293512	5831	HiConf	Variation_38051	chr11	107289717	107292923	3206	McCarroll et al. (2008)	Affymetrix Human SNP Array 6.0	
4062	chr11	107955729	107979396	23667	48	chr11	107955698	107979855	24157	HiConf	Variation_4761	chr11	107954824	108128930	174106	Wong et al. (2007)	BAC Array CGH	
4063	chr11	108307745	108309460	1715	8	chr11	108307714	108309478	1764	HiConf								
4064	chr11	108392279	108392432	153	60	chr11	108392239	108392435	196	single								
4065	chr11	108901086	108907071	5985	9	chr11	108901055	108907180	6125	HiConf								
4066	chr11	110533815	110538421	4606	5	chr11	110533784	110538488	4704	HiConf								y
4067	chr11	110804992	110807480	2488	11	chr11	110804950	110807547	2597	single								
100673	chr11	110872626	110916224	43598	3	chr11	110872595	110917381	44786	single								
4068	chr11	111555955	111561051	5096	2	chr11	111555924	111561167	5243	single								
4069	chr11	112492688	112495089	2401	8	chr11	112492657	112495107	2450	HiConf								
4070	chr11	115165099	115165932	833	4	chr11	115165068	115165950	882	single	Variation_8698	chr11	114988378	115246794	258416	Pinto et al. (2007)	Affymetrix 500K SNP Mapping Array	
4071	chr11	115393439	115395889	2450	3	chr11	115393408	115395956	2548	HiConf								
4072	chr11	116173742	116178713	4971	16	chr11	116173733	116178829	5096	HiConf	Variation_37489	chr11	116173701	116179119	5418	Cooper et al. (2008)	Illumina Human 1M BeadChip	
4073	chr11	116585142	116586255	1113	2	chr11	116582197	116586901	4704	single								
4074	chr11	117787334	117787694	360	12	chr11	117787303	117787695	392	single								
100674	chr11	118269813	118298037	28224	12	chr11	118269782	118298618	28836	single								
4075	chr11	119026936	119041342	14406	1	chr11	119026905	119041605	14700	single								y
4076	chr11	119733908	119734888	980	13	chr11	119733877	119734906	1029	single								
4077	chr11	123184096	123187134	3038	3	chr11	123184065	123187201	3136	HiConf								
4078	chr11	123413367	123425870	12503	1	chr11	123413336	123426125	12789	single								
100675	chr11	124034859	124107477	72618	5	chr11	124034828	124108940	74112	HiConf								
4079	chr11	124187665	124187975	310	6	chr11	124187634	124187977	343	HiConf								
4080	chr11	124792227	124793060	833	5	chr11	124792196	124793078	882	single	Variation_5037	chr11	124705792	124839738	133946	Wong et al. (2007)	BAC Array CGH	
100676	chr11	124949480	125018681	69201	4	chr11	124949217	125020071	70854	single	Variation_6905	chr11	124870175	125421492	551317	de Smith et al. (2007)	Agilent 185k CGH Arrays/Agilent Custom CGH Arrays	
4082	chr11	126463323	126472388	9065	11	chr11	126463292	126472553	9261	HiConf								
4083	chr11	126780108	126785057	4949	3	chr11	126780077	126785173	5096	HiConf								
4084	chr11	127817046	127819202	2156	56	chr11	127817015	127819220	2205	HiConf	Variation_34922	chr11	127810163	127822396	12233	Kidd et al. (2008)	Paired End Mapping	
4085	chr11	128188025	128188425	400	27	chr11	128187994	128188435	441	single	Variation_11591	chr11	128187928	128188620	692	Mills et al. (2006)	Sequence trace read mapping	y
100677	chr11	129329701	129478125	148424	10	chr11	129329327	129478360	149033	single	Variation_5652	chr11	129341195	129344745	3550	Mills et al. (2006)	Sequence trace read mapping	
4086	chr11	130426443	130427129	686	3	chr11	130426412	130427147	735	single								
4087	chr11	130707164	130710251	3087	2	chr11	130707133	130710318	3185	HiConf								
4088	chr11	131101565	131102300	735	12	chr11	131101534	131102318	784	HiConf	Variation_3870	chr11	130938011	131254195	316184	Redon et al. (2006)	BAC Array CGH	
4089	chr11	132729737	132732922	3185	22	chr11	132729706	132732989	3283	single	Variation_8700	chr11	132316000	132797586	481586	Pinto et al. (2007)	Affymetrix 500K SNP Mapping Array	
4090	chr11	132808676	132819505	10829	23	chr11	132808645	132819719	11074	HiConf	Variation_34461	chr11	132245168	132876984	631816	Zogopoulos et al. (2007)	Affymetrix 500K and 100K SNP Mapping Arrays	
4091	chr11	133078911	133086996	8085	4	chr11	133078880	133087161	8281	single								
4092	chr11	133298676	133319158	20482	9	chr11	133298645	133319568	20923	HiConf								
4093	chr11	133512757	133513492	735	21	chr11	133512726	133513510	784	single								
100679	chr11	133665176	133702363	37187	12	chr11	133664896	133703140	38244	HiConf	Variation_29921	chr11	133654072	133715739	61667	Jakobsson et al. (2008)	Illumina HumanHap550 BeadChip	y
100680	chr11	134078642	134114862	36220	14	chr11	134077647	134114912	37265	HiConf	Variation_30570	chr11	134080796	134111845	31049	Perry et al. (2008)	Agilent Custom CGH Arrays	y
4096	chr11	134202187	134226436	24249	47	chr11	134202156	134226656	24500	HiConf	Variation_44841	chr11	134214522	134215430	908	Bentley et al. (2008)	Illumina DNA sequencing	
4097	chr11	134226883	134227243	360	48	chr11	134226852	134227244	392	HiConf	Variation_8702	chr11	133816000	134263730	447730	Pinto et al. (2007)	Affymetrix 500K SNP Mapping Array	
4098	chr11	134238251	134239231	980	30	chr11	134238220	134239249	1029	HiConf	Variation_8702	chr11	133816000	134263730	447730	Pinto et al. (2007)	Affymetrix 500K SNP Mapping Array	
4099	chr11	134247806	134253294	5488	1	chr11	134247775	134253410	5635	single	Variation_8702	chr11	133816000	134263730	447730	Pinto et al. (2007)	Affymetrix 500K SNP Mapping Array	y
100681	chr12	17451	59493	42042	16	chr12	17420	60320	42900	HiConf	Variation_30571	chr12	18651	46748	28097	Perry et al. (2008)	Agilent Custom CGH Arrays	y
100682	chr12	297388	415156	117768	53	chr12	297357	415472	118115	HiConf	Variation_37675	chr12	303069	414108	111039	McCarroll et al. (2008)	Affymetrix Human SNP Array 6.0	y
4102	chr12	482902	494138	11236	33	chr12	482871	494484	11613	HiConf	Variation_25023	chr12	491153	491866	713	Levy et al. (2007)	Sequencing	
4103	chr12	570220	570335	115	40	chr12	570189	570336	147	single	Variation_25042	chr12	570234	570482	248	Levy et al. (2007)	Sequencing	
100683	chr12	690704	843052	152348	5	chr12	690435	845030	154595	HiConf	Variation_2966	chr12	656499	834812	178313	Redon et al. (2006)	Affymetrix 500K EA SNP Mapping Array	
4104	chr12	939778	948898	9120	4	chr12	939747	949253	9506	HiConf	Variation_10384	chr12	945143	948337	3194	Wang et al. (2007)	Illumina HumanHap550 BeadChip	
4105	chr12	1577709	1585157	7448	7	chr12	1577678	1585322	7644	single	Variation_4765	chr12	1580511	1738903	158392	Wong et al. (2007)	BAC Array CGH	
4106	chr12	1585500	1588293	2793	11	chr12	1585469	1588360	2891	single	Variation_4765	chr12	1580511	1738903	158392	Wong et al. (2007)	BAC Array CGH	
4107	chr12	1588930	1599367	10437	5	chr12	1588899	1599581	10682	single	Variation_4765	chr12	1580511	1738903	158392	Wong et al. (2007)	BAC Array CGH	
4108	chr12	1631364	1642536	11172	19	chr12	1631333	1642750	11417	HiConf	Variation_42644	chr12	1640462	1640977	515	Wang et al. (2008)	Illumina DNA sequencing	y
4109	chr12	1788605	1795514	6909	6	chr12	1788574	1795630	7056	HiConf								
4110	chr12	2230193	2235387	5194	7	chr12	2230162	2235503	5341	single	Variation_0424	chr12	2229492	2241937	12445	Tuzun et al. (2005)	Paired End Mapping	

locus_id	chrom	start	end	length	Yoruba w/ event	putative chr	putative start	putative end	putative len	putative type	variation_id	DGV_chr	DGV_start	DGV_end	DGV_len	Reference	Method/platform	complex
4111	chr12	2738715	2741165	2450	2	chr12	2738684	2741232	2548	single	Variation_4766	chr12	2705497	2879935	174438	Wong et al. (2007)	BAC Array CGH	
4112	chr12	3242239	3247384	5145	5	chr12	3242208	3247500	5292	single	Variation_0161	chr12	3099613	3265128	165515	Iafrate et al. (2004)	BAC Array CGH	y
4113	chr12	4598069	4616693	18624	18	chr12	4598038	4616952	18914	HiConf								
4114	chr12	5092136	5093655	1519	19	chr12	5092105	5093673	1568	HiConf	Variation_43856	chr12	5092084	5093714	1630	Bentley et al. (2008)	Illumina DNA sequencing	
4115	chr12	5410195	5413184	2989	28	chr12	5410164	5413251	3087	single								
4116	chr12	5596346	5597032	686	19	chr12	5596315	5597050	735	HiConf								
4117	chr12	5643337	5654803	11466	9	chr12	5643306	5655017	11711	HiConf								
4118	chr12	5878528	5904345	25817	19	chr12	5878457	5904868	26411	single								
4119	chr12	6444438	6453601	9163	5	chr12	6444407	6453766	9359	single	Variation_30577	chr12	6410536	6452546	42010	Perry et al. (2008)	Agilent Custom CGH Arrays	
4120						chr12	6902998	6930340	27342	HiConf								
4121	chr12	7171843	7188356	16513	1	chr12	7171812	7188668	16856	HiConf	Variation_9680	chr12	7172584	7193249	20665	Wang et al. (2007)	Illumina HumanHap550 BeadChip	
100684	chr12	7854055	7969563	115508	4	chr12	7853255	7971933	118678	single	Variation_29613	chr12	7876208	7960148	83940	Jakobsson et al. (2008)	Illumina HumanHap550 BeadChip	
4122	chr12	8211280	8268349	57069	21	chr12	8211249	8269510	58261	HiConf	Variation_30580	chr12	8206659	8271580	64921	Perry et al. (2008)	Agilent Custom CGH Arrays	
4123	chr12	8270325	8330879	60554	8	chr12	8270294	8332181	61887	HiConf	Variation_37620	chr12	8237875	8322268	84393	Cooper et al. (2008)	Illumina Human 1M BeadChip	
4124	chr12	8333584	8491571	157987	11	chr12	8333553	8492215	158662	HiConf	Variation_35121	chr12	8442228	8494779	52551	Kidd et al. (2008)	Paired End Mapping	
4125	chr12	8711374	8713432	2058	3	chr12	8711343	8713450	2107	HiConf								
4126	chr12	8786148	8790068	3920	12	chr12	8786117	8790135	4018	HiConf	Variation_22595	chr12	8785058	8790881	5823	Korbel et al. (2007)	Paired End Mapping	
4127	chr12	8901249	8905169	3920	4	chr12	8901218	8905236	4018	HiConf	Variation_38271	chr12	8902508	8905023	2515	McCarroll et al. (2008)	Affymetrix Human SNP Array 6.0	
4128	chr12	8908520	8908658	138	75	chr12	8908519	8908666	147	HiConf	Variation_5654	chr12	8908348	8909373	1025	Mills et al. (2006)	Sequence trace read mapping	
4129	chr12	9369490	9369934	444	2	chr12	9368090	9369952	1862	single	Variation_3874	chr12	9262862	9814796	551934	Redon et al. (2006)	BAC Array CGH	y
4130	chr12	9437799	9453105	15306	39	chr12	9437768	9453154	15386	HiConf	Variation_30582	chr12	9439574	9446000	6426	Perry et al. (2008)	Agilent Custom CGH Arrays	
4131	chr12	9524235	9622529	98294	50	chr12	9524204	9623282	99078	HiConf	Variation_30584	chr12	9524510	9623223	98713	Perry et al. (2008)	Agilent Custom CGH Arrays	
4132	chr12	9747185	9766834	19649	5	chr12	9747154	9767244	20090	HiConf	Variation_38275	chr12	9753345	9766191	12846	McCarroll et al. (2008)	Affymetrix Human SNP Array 6.0	
100687	chr12	10456093	10491814	35721	7	chr12	10456062	10492542	36480	HiConf	Variation_31857	chr12	10459687	10488400	28713	Perry et al. (2008)	Agilent Custom CGH Arrays	
4134	chr12	10967481	10967589	108	5	chr12	10967450	10967597	147	single	Variation_3875	chr12	10860887	11606188	745301	Redon et al. (2006)	BAC Array CGH	
4135	chr12	11028682	11033005	4323	2	chr12	11028651	11033061	4410	single	Variation_4769	chr12	10968945	11098224	129279	Wong et al. (2007)	BAC Array CGH	
100688	chr12	11106298	11140500	34202	29	chr12	11106267	11141204	34937	HiConf	Variation_38772	chr12	11113633	11132799	19166	McCarroll et al. (2008)	Affymetrix Human SNP Array 6.0	
4137	chr12	11348995	11358698	9703	2	chr12	11348964	11364644	15680	HiConf	Variation_9683	chr12	11350073	11356963	6890	Wang et al. (2007)	Illumina HumanHap550 BeadChip	
4138	chr12	11395055	11457530	62475	23	chr12	11395024	11458822	63798	HiConf	Variation_31861	chr12	11393207	11459990	66783	Perry et al. (2008)	Agilent Custom CGH Arrays	
4139	chr12	11473308	11474288	980	6	chr12	11473277	11474306	1029	HiConf	Variation_3875	chr12	10860887	11606188	745301	Redon et al. (2006)	BAC Array CGH	y
4140	chr12	11917836	11918236	400	53	chr12	11917805	11918246	441	HiConf	Variation_44976	chr12	11917630	11918424	794	Bentley et al. (2008)	Illumina DNA sequencing	
4141	chr12	12099577	12099685	108	9	chr12	12099546	12099693	147	single								
4142	chr12	12144216	12145833	1617	6	chr12	12144185	12145851	1666	HiConf								
4143	chr12	12351486	12382552	31066	14	chr12	12351455	12383158	31703	HiConf								
4144	chr12	12421164	12433824	12660	8	chr12	12421133	12434167	13034	HiConf	Variation_10389	chr12	12421985	12435533	13548	Wang et al. (2007)	Illumina HumanHap550 BeadChip	
4145	chr12	13183800	13185368	1568	34	chr12	13183769	13185386	1617	HiConf								
4146	chr12	14104510	14107793	3283	18	chr12	14104479	14107860	3381	HiConf								
100690	chr12	14765888	14866632	100744	2	chr12	14765857	14868659	102802	single								
4147	chr12	15088675	15102591	13916	15	chr12	15088644	15102854	14210	HiConf								
100692	chr12	15348569	15399090	50521	1	chr12	15348491	15400137	51646	single	Variation_6971	chr12	15323654	15398874	75220	de Smith et al. (2007)	Agilent 185k CGH Arrays/Agilent Custom CGH Arrays	
4149	chr12	15459477	15465877	6400	13	chr12	15459476	15465993	6517	HiConf	Variation_38256	chr12	15459186	15464906	5720	McCarroll et al. (2008)	Affymetrix Human SNP Array 6.0	
4150	chr12	15790649	15797803	7154	2	chr12	15790618	15797919	7301	single								
4151	chr12	15910062	15913051	2989	19	chr12	15910031	15913118	3087	HiConf	Variation_22705	chr12	15906760	15913605	6845	Korbel et al. (2007)	Paired End Mapping	
4152	chr12	16198280	16198487	207	11	chr12	16198249	16198494	245	single	Variation_3877	chr12	16156359	16337645	181286	Redon et al. (2006)	BAC Array CGH	
4153	chr12	16311470	16311902	432	50	chr12	16311439	16312272	833	HiConf	Variation_3877	chr12	16156359	16337645	181286	Redon et al. (2006)	BAC Array CGH	
4154	chr12	17336275	17336638	363	78	chr12	17336274	17337009	735	HiConf	Variation_39099	chr12	17336094	17337206	1112	Wheeler et al. (2008)	Sequencing	
4155	chr12	17480806	17492419	11613	5	chr12	17480775	17492633	11858	HiConf	Variation_38257	chr12	17481711	17491817	10106	McCarroll et al. (2008)	Affymetrix Human SNP Array 6.0	
4156	chr12	18274067	18288326	14259	9	chr12	18274036	18288589	14553	HiConf	Variation_1249	chr12	18278058	18284911	6853	Conrad et al. (2005)	Mendelian inconsistencies	
4157	chr12	18614813	18616185	1372	18	chr12	18614782	18616203	1421	HiConf								
4158	chr12	19840009	19840319	310	9	chr12	19839978	19840321	343	single								
100693						chr12	20130965	20165951	34986	single								
4160	chr12	20597010	20598088	1078	15	chr12	20596979	20598106	1127	single								
4161						chr12	21752987	21753036	49	single								
4162	chr12	22020754	22022421	1667	11	chr12	22020723	22022438	1715	HiConf	Variation_24976	chr12	22021655	22022477	822	Levy et al. (2007)	Sequencing	
4163	chr12	22214796	22217734	2938	4	chr12	22214420	22217801	3381	HiConf	Variation_0163	chr12	22210387	22369559	159172	Iafrate et al. (2004)	BAC Array CGH	
4164	chr12	22235570	22236305	735	3	chr12	22235539	22236323	784	single	Variation_0163	chr12	22210387	22369559	159172	Iafrate et al. (2004)	BAC Array CGH	y
4165	chr12	22462881	22475881	13000	53	chr12	22462850	22475933	13083	HiConf	Variation_30589	chr12	22463304	22474599	11295	Perry et al. (2008)	Agilent Custom CGH Arrays	
4166	chr12	22757763	22763007	5244	3	chr12	22757732	22763465	5733	single								
4167	chr12	23830834	23831313	479	76	chr12	23830832	23831322	490	HiConf	Variation_11066	chr12	23830794	23831195	401	de Smith et al. (2007)	Agilent 185k CGH Arrays/Agilent Custom CGH Arrays	
100694	chr12	24558930	24652809	93879	3	chr12	24558899	24654718	95819	single	Variation_5294	chr12	24410659	25112953	702294	Simon-Sanchez et al. (2007)	Illumina HumanHap300 BeadChip	
100695	chr12	24884608	25005155	120547	3	chr12	24884577	25007641	123064	single	Variation_5294	chr12	24410659	25112953	702294	Simon-Sanchez et al. (2007)	Illumina HumanHap300 BeadChip	
4168	chr12	25525675	25528566	2891	3	chr12	25525644	25528633	2989	HiConf								y
100696	chr12	26806143	26887040	80897	13	chr12	26806112	26887869	81757	HiConf								
4169	chr12	27539575	27546582	7007	20	chr12	27539544	27546698	7154	HiConf	Variation_37490	chr12	27539233	27546356	7123	Cooper et al. (2008)	Illumina Human 1M BeadChip	
4170	chr12	27766788	27773991	7203	7	chr12	27766757	27774107	7350	HiConf								
4171	chr12	27987386	27989444	2058	9	chr12	27987355	27989462	2107	HiConf	Variation_37994	chr12	27986760	27988911	2151	McCarroll et al. (2008)	Affymetrix Human SNP Array 6.0	
4172	chr12	28656629	28658980	2351	3	chr12	28656597	28658998	2401	single	Variation_38259	chr12	28656614	28658887	2273	McCarroll et al. (2008)	Affymetrix Human SNP Array 6.0	
4173	chr12	28902363	28908488	6125	5	chr12	28902332	28908604	6272	HiConf								
4174	chr12	29053234	29055978	2744	21	chr12	29053203	29056045	2842	HiConf	Variation_3882	chr12	29002049	29181116	179067	Redon et al. (2006)	BAC Array CGH	
4175	chr12	29735853	29737372	1519	21	chr12	29735822	29737390	1568	HiConf								
4176	chr12	30043181	30046807	3626	2	chr12	30043150	30046874	3724	single	Variation_3883	chr12	29914434	30066499	152065	Redon et al. (2006)	BAC Array CGH	
4177	chr12	30128492	30134664	6172	11	chr12	30128410	30134780	6370	HiConf	Variation_38016	chr12	30128768	30135200	6432	McCarroll et al. (2008)	Affymetrix Human SNP Array 6.0	
4178	chr12	30369493	30371790	2297	4	chr12	30369392	30371842	2450	single	Variation_23440	chr12	30369554	30372260	2706	Levy et al. (2007)	Sequencing	y
100697	chr12	31098788	31292537	193749	36	chr12	31098757	31295076	196319	HiConf	Variation_9687	chr12	31101381	31298174	196793	Wang et al. (2007)	Illumina HumanHap550 BeadChip	
4180	chr12	31531213	31548265	17052	6	chr12	31531182	31548626	17444	single	Variation_8717	chr12	31065500	31576000	510500	Pinto et al. (2007)	Affymetrix 500K SNP Mapping Array	
4181	chr12	31796254	31801654	5400	20	chr12	31796223	31802005	5782	HiConf	Variation_10392	chr12	31795038	31803855	8817	Wang et al. (2007)	Illumina HumanHap550 BeadChip	
4182	chr12	32294192	32298504	4312	16	chr12	32294161	32298571	4410	single	Variation_8721	chr12	32171607	32594051	422444	Pinto et al. (2007)	Affymetrix 500K SNP Mapping Array	
4183	chr12	32299435	32303431	3996	3	chr12	32299404	32304990	5586	single	Variation_8721	chr12	32171607	32594051	422444	Pinto et al. (2007)	Affymetrix 500K SNP Mapping Array	
100698	chr12	33068850	33113287	44437	1	chr12	33053441	33138897	85456	HiConf								
4185	chr12	33192313	33199320	7007	9	chr12	33192282	33199436	7154	HiConf	Variation_37987	chr12	33192673	33198641	5968	McCarroll et al. (2008)	Affymetrix Human SNP Array 6.0	
4186	chr12	33271327	33289520	18193	58	chr12	33271319	33289890	18571	HiConf	Variation_37823	chr12	33270866	33286685	15819	McCarroll et al. (2008)	Affymetrix Human SNP Array 6.0	
4187	chr12	33605824	33626943	21119	17	chr12	33605793	33627353	21560	HiConf	Variation_10394	chr12	33611229	33626202	14973	Wang et al. (2007)	Illumina HumanHap550 BeadChip	
4188	chr12	33990376	34000568	10192	12	chr12	33990345	34000782	10437	HiConf	Variation_31872	chr12	33990189	34000127	9938	Perry et al. (2008)	Agilent Custom CGH Arrays	
4189	chr12	34098715	34108319	9604	47	chr12	34098684	34108484	9800	HiConf	Variation_37525	chr12	34098698	34108391	9693	Cooper et al. (2008)	Illumina Human 1M BeadChip	
4190	chr12	34143060	34153644	10584	18	chr12	34143029	34153858	10829	HiConf	Variation_31875	chr12	34143040	34152928	9888	Perry et al. (2008)	Agilent Custom CGH Arrays	
4191	chr12	34169863	34171235	1372	2	chr12	34169832	34171253	1421	single	Variation_2170	chr12	34086087	34188854	102767	Locke et al. (2006)	BAC Array CGH	

locus_id	chrom	start	end	length	Yoruba w/ event	putative chr	putative start	putative end	putative len	putative type	variation_id	DGV_chr	DGV_start	DGV_end	DGV_len	Reference	Method/platform	complex
4192	chr12	34223959	34242369	18410	1	chr12	34223928	34289686	65758	HiConf	Variation_8722	chr12	34237698	34651600	413902	Pinto et al. (2007)	Affymetrix 500K SNP Mapping Array	y
4194	chr12	34341781	34399409	57628	8	chr12	34341724	34400524	58800	HiConf	Variation_31878	chr12	34377077	34649713	272636	Perry et al. (2008)	Agilent Custom CGH Arrays	
100700						chr12	35433273	36190788	757515	HiConf								
4196	chr12	36516081	36687042	170961	16	chr12	36516050	36690539	174489	HiConf	Variation_10395	chr12	36528296	36667312	139016	Wang et al. (2007)	Illumina HumanHap550 BeadChip	
4197	chr12	37030581	37038470	7889	14	chr12	37030550	37038635	8085	single	Variation_31885	chr12	37034789	37041755	6966	Perry et al. (2008)	Agilent Custom CGH Arrays	
100702	chr12	37742833	37764577	21744	2	chr12	37742349	37765011	22662	single	Variation_35081	chr12	37752064	37775663	23599	Kidd et al. (2008)	Paired End Mapping	
4199	chr12	38588022	38601766	13744	4	chr12	38588015	38602029	14014	HiConf	Variation_22865	chr12	38587871	38602453	14582	Korbel et al. (2007)	Paired End Mapping	
4200	chr12	39101370	39107740	6370	11	chr12	39101339	39107856	6517	HiConf	Variation_37935	chr12	39103864	39107744	3880	McCarroll et al. (2008)	Affymetrix Human SNP Array 6.0	
4201	chr12	39161297	39162179	882	22	chr12	39161266	39162197	931	HiConf	Variation_43852	chr12	39161026	39162695	1669	Bentley et al. (2008)	Illumina DNA sequencing	
4202	chr12	39162620	39171391	8771	9	chr12	39162589	39171556	8967	HiConf	Variation_30598	chr12	39163813	39168374	4561	Perry et al. (2008)	Agilent Custom CGH Arrays	
4203	chr12	39314471	39315206	735	19	chr12	39314440	39315224	784	HiConf	Variation_45046	chr12	39314310	39315293	983	Bentley et al. (2008)	Illumina DNA sequencing	
4204	chr12	40985340	40985388	48	29	chr12	40984997	40985389	392	HiConf								
4205	chr12	41306909	41315337	8428	7	chr12	41306878	41315502	8624	HiConf	Variation_6984	chr12	41306841	41313692	6851	de Smith et al. (2007)	Agilent 185k CGH Arrays/Agilent Custom CGH Arrays	
100704	chr12	42466365	42551607	85242	3	chr12	42466316	42553316	87000	HiConf								
4206	chr12	42978299	42979034	735	25	chr12	42978268	42979052	784	HiConf								
4207	chr12	43559537	43569533	9996	6	chr12	43559506	43569747	10241	HiConf								
4208	chr12	44189285	44195723	6438	46	chr12	44189254	44195869	6615	HiConf	Variation_37492	chr12	44189465	44195940	6475	Cooper et al. (2008)	Illumina Human 1M BeadChip	
100705	chr12	44666175	44680075	13900	1	chr12	44648041	44717597	69556	single								
4209	chr12	44759498	44759858	360	16	chr12	44759467	44759859	392	single								
4210	chr12	44917425	44918454	1029	15	chr12	44917394	44918472	1078	HiConf								
4211	chr12	45315158	45319127	3969	29	chr12	45315127	45319194	4067	HiConf	Variation_35092	chr12	45312128	45326117	13989	Kidd et al. (2008)	Paired End Mapping	
4212	chr12	45963722	45966711	2989	6	chr12	45963691	45966778	3087	HiConf	Variation_9205	chr12	45827657	46032424	204767	Pinto et al. (2007)	Affymetrix 500K SNP Mapping Array	
4213	chr12	46380810	46392227	11417	1	chr12	46380779	46392441	11662	single	Variation_8728	chr12	46227125	46413152	186027	Pinto et al. (2007)	Affymetrix 500K SNP Mapping Array	
4214	chr12	47686268	47694794	8526	3	chr12	47686237	47694959	8722	single	Variation_4777	chr12	47544827	47744822	199995	Wong et al. (2007)	BAC Array CGH	
4215	chr12	48450325	48458116	7791	3	chr12	48450294	48458281	7987	single								
4216						chr12	48562014	48573039	11025	HiConf								
4217	chr12	49113099	49132945	19846	8	chr12	49113068	49133403	20335	single								
4218	chr12	49260050	49261716	1666	14	chr12	49260019	49261734	1715	HiConf	Variation_43397	chr12	49259944	49261881	1937	Wang et al. (2008)	Illumina DNA sequencing	
4219	chr12	50235640	50236914	1274	6	chr12	50235609	50236932	1323	single								
4220	chr12	50787184	50787870	686	21	chr12	50787153	50787888	735	single								
4221	chr12	51662373	51664578	2205	25	chr12	51662342	51664596	2254	HiConf								y
100706	chr12	52618768	52791459	172691	15	chr12	52599369	52792625	193256	HiConf	Variation_3890	chr12	52633336	52820913	187577	Redon et al. (2006)	BAC Array CGH	
4222	chr12	53480558	53481155	597	5	chr12	53479458	53481173	1715	single								
4223	chr12	53897263	53900466	3203	4	chr12	53897232	53900515	3283	HiConf								
4224	chr12	54288626	54290439	1813	66	chr12	54288595	54290457	1862	HiConf	Variation_43857	chr12	54288540	54290512	1972	Bentley et al. (2008)	Illumina DNA sequencing	
4225	chr12	54318271	54320672	2401	11	chr12	54318240	54320739	2499	HiConf								y
4226	chr12	54347622	54348504	882	25	chr12	54347591	54348522	931	HiConf								
4227	chr12	55395095	55399799	4704	45	chr12	55395064	55399866	4802	HiConf	Variation_45060	chr12	55397743	55398007	264	Bentley et al. (2008)	Illumina DNA sequencing	
4228	chr12	55619051	55662760	43709	3	chr12	55618945	55663731	44786	single	Variation_22920	chr12	55618247	55664031	45784	Korbel et al. (2007)	Paired End Mapping	y
4229	chr12	56314580	56314740	160	36	chr12	56314549	56314892	343	single	Variation_43858	chr12	56314314	56315556	1242	Bentley et al. (2008)	Illumina DNA sequencing	y
4230	chr12	56795596	56799778	4182	53	chr12	56787693	56800041	12348	HiConf	Variation_43859	chr12	56795894	56800008	4114	Bentley et al. (2008)	Illumina DNA sequencing	
4231	chr12	57071875	57076530	4655	4	chr12	57071844	57076597	4753	HiConf								
100707	chr12	57298015	57334638	36623	13	chr12	57297955	57335391	37436	single	Variation_3892	chr12	57308996	57609312	300316	Redon et al. (2006)	BAC Array CGH	
4233	chr12	58173983	58176531	2548	5	chr12	58173952	58176598	2646	single								
4234	chr12	58224159	58234057	9898	24	chr12	58224128	58234271	10143	HiConf	Variation_1502	chr12	58224199	58230898	6699	Conrad et al. (2005)	Mendelian inconsistencies	
4235	chr12	58388799	58390269	1470	14	chr12	58388768	58390287	1519	HiConf								
4236	chr12	58730917	58733514	2597	2	chr12	58730886	58733581	2695	single								
4237	chr12	58808161	58811179	3018	7	chr12	58808159	58811246	3087	HiConf	Variation_23447	chr12	58808112	58811308	3196	Levy et al. (2007)	Sequencing	
4238	chr12	59039323	59043978	4655	10	chr12	59039292	59044045	4753	HiConf	Variation_31890	chr12	59039839	59044045	4206	Perry et al. (2008)	Agilent Custom CGH Arrays	
4239	chr12	59432107	59438741	6634	5	chr12	59432076	59438838	6762	HiConf								
100708	chr12	59798480	59884279	85799	2	chr12	59798449	59886037	87588	single	Variation_2986	chr12	59797263	59898818	101555	Redon et al. (2006)	Affymetrix 500K EA SNP Mapping Array	
4241	chr12	60173232	60176221	2989	7	chr12	60173201	60176288	3087	HiConf								
4242	chr12	60379179	60382805	3626	3	chr12	60379148	60382872	3724	HiConf								
4243	chr12	60415831	60417448	1617	5	chr12	60415800	60417466	1666	single								
4244	chr12	60488645	60497024	8379	10	chr12	60488614	60497189	8575	HiConf	Variation_37873	chr12	60488837	60497087	8250	McCarroll et al. (2008)	Affymetrix Human SNP Array 6.0	
4245	chr12	60759909	60760889	980	3	chr12	60759878	60760907	1029	single								
4246	chr12	61781559	61784387	2828	4	chr12	61781528	61784468	2940	single	Variation_8733	chr12	61732200	61790219	58019	Pinto et al. (2007)	Affymetrix 500K SNP Mapping Array	
100709	chr12	62322432	62351806	29374	1	chr12	62231936	62354191	122255	HiConf	Variation_30606	chr12	62226339	62347043	120704	Perry et al. (2008)	Agilent Custom CGH Arrays	
4247	chr12	62685854	62691587	5733	3	chr12	62685823	62691703	5880	HiConf	Variation_38264	chr12	62687505	62691702	4197	McCarroll et al. (2008)	Affymetrix Human SNP Array 6.0	
4248	chr12	64256549	64264807	8258	8	chr12	64256518	64264897	8379	HiConf								
4249	chr12	65042656	65043056	400	8	chr12	65042625	65043066	441	single								
4250	chr12	65078671	65080288	1617	3	chr12	65078640	65080306	1666	single								
4251						chr12	65210401	65210450	49	single								
4252	chr12	67542195	67542645	450	74	chr12	67542164	67542654	490	single	Variation_11626	chr12	67542043	67542784	741	Mills et al. (2006)	Sequence trace read mapping	
4253	chr12	67818653	67820319	1666	16	chr12	67818622	67820337	1715	single								
100710	chr12	68394771	68535352	140581	4	chr12	68394740	68538236	143496	single								
4254	chr12	68613678	68615442	1764	10	chr12	68613647	68615460	1813	single								
4255	chr12	69158460	69164340	5880	13	chr12	69158429	69164456	6027	HiConf	Variation_38019	chr12	69158942	69164294	5352	McCarroll et al. (2008)	Affymetrix Human SNP Array 6.0	
4256	chr12	69549382	69557320	7938	2	chr12	69549351	69557485	8134	single								
4257	chr12	69815773	69818735	2962	33	chr12	69815715	69818802	3087	single								
4258	chr12	70052579	70055835	3256	7	chr12	70052532	70056207	3675	single								
4259	chr12	70172270	70175504	3234	31	chr12	70172239	70175571	3332	HiConf	Variation_22596	chr12	70172816	70178644	5828	Korbel et al. (2007)	Paired End Mapping	
4260	chr12	70475531	70477099	1568	58	chr12	70475500	70477117	1617	HiConf	Variation_43336	chr12	70475389	70476927	1538	Wang et al. (2008)	Illumina DNA sequencing	
4261	chr12	71044372	71051364	6992	6	chr12	71044341	71053553	9212	single	Variation_5311	chr12	70772200	71461848	689648	Simon-Sanchez et al. (2007)	Illumina HumanHap300 BeadChip	y
100711	chr12	71638273	71691948	53675	17	chr12	71632243	71715004	82761	single	Variation_2992	chr12	71660953	71703530	42577	Redon et al. (2006)	Affymetrix 500K EA SNP Mapping Array	
4263	chr12	72931019	72933469	2450	74	chr12	72930988	72933536	2548	HiConf								
4264	chr12	73398528	73401615	3087	5	chr12	73398497	73401682	3185	HiConf	Variation_43867	chr12	73397845	73401738	3893	Bentley et al. (2008)	Illumina DNA sequencing	
4265	chr12	73977365	74022641	45276	4	chr12	73977334	74023541	46207	single								
4266	chr12	75425021	75425228	207	6	chr12	75424990	75425235	245	single								
4267	chr12	75639837	75641945	2108	19	chr12	75639806	75641962	2156	single								
4268	chr12	76198192	76198780	588	48	chr12	76198161	76198798	637	HiConf								
4269	chr12	76623414	76624845	1431	27	chr12	76623383	76624853	1470	HiConf								
4270	chr12	77714105	77716555	2450	5	chr12	77714074	77716622	2548	single								
4271	chr12	77721063	77723026	1963	22	chr12	77721032	77723041	2009	HiConf								
4272	chr12	78203664	78215375	11711	2	chr12	78203633	78215589	11956	single								
4273	chr12	78808177	78809549	1372	16	chr12	78808146	78809567	1421	single								

locus_id	chrom	start	end	length	Yoruba w/ event	putative chr	putative start	putative end	putative len	putative type	variation_id	DGV_chr	DGV_start	DGV_end	DGV_len	Reference	Method/platform	complex
4274	chr12	78810039	78812636	2597	12	chr12	78810008	78812703	2695	single								
4275	chr12	78960224	78962233	2009	6	chr12	78960193	78962251	2058	HiConf	Variation_10696	chr12	78961464	78962049	585	Conrad et al. (2005)	Mendelian inconsistencies	
4276	chr12	79348010	79352224	4214	5	chr12	79347979	79352291	4312	HiConf	Variation_37851	chr12	79349306	79352242	2936	McCarroll et al. (2008)	Affymetrix Human SNP Array 6.0	
4277	chr12	80731133	80749067	17934	14	chr12	80731102	80749428	18326	HiConf	Variation_35119	chr12	80730274	80757053	26779	Kidd et al. (2008)	Paired End Mapping	
4278	chr12	81518563	81518671	108	19	chr12	81518532	81518679	147	HiConf								
100713	chr12	81565652	81654440	88788	1	chr12	81565621	81656247	90626	single	Variation_41669	chr12	81607144	81607473	329	Wang et al. (2008)	Illumina DNA sequencing	
4279	chr12	81763427	81769394	5967	5	chr12	81763385	81769510	6125	HiConf	Variation_1265	chr12	81764273	81766799	2526	Conrad et al. (2005)	Mendelian inconsistencies	
4280	chr12	82776442	82784870	8428	2	chr12	82776411	82785035	8624	single	Variation_4783	chr12	82690977	82845136	154159	Wong et al. (2007)	BAC Array CGH	
4281	chr12	83117384	83120520	3136	18	chr12	83117353	83120587	3234	HiConf	Variation_6994	chr12	83117333	83120576	3243	de Smith et al. (2007)	Agilent 185k CGH Arrays/Agilent Custom CGH Arrays	
4282	chr12	83136887	83138209	1322	6	chr12	83136855	83138227	1372	single								
4283	chr12	84817096	84818272	1176	4	chr12	84817065	84818290	1225	single								
4284	chr12	85177795	85234331	56536	4	chr12	85177754	85235476	57722	HiConf	Variation_35125	chr12	85173069	85200842	27773	Kidd et al. (2008)	Paired End Mapping	
4285	chr12	85648381	85652889	4508	5	chr12	85648350	85652956	4606	HiConf								
4286	chr12	85934590	85951789	17199	4	chr12	85934559	85952150	17591	HiConf	Variation_38269	chr12	85937514	85950402	12888	McCarroll et al. (2008)	Affymetrix Human SNP Array 6.0	
4287	chr12	86054689	86057580	2891	12	chr12	86054658	86057647	2989	HiConf	Variation_38270	chr12	86055044	86057392	2348	McCarroll et al. (2008)	Affymetrix Human SNP Array 6.0	
4288	chr12	87536596	87539144	2548	4	chr12	87536565	87539211	2646	HiConf	Variation_31899	chr12	87537338	87539234	1896	Perry et al. (2008)	Agilent Custom CGH Arrays	
4289	chr12	89012231	89016102	3871	22	chr12	89012200	89016169	3969	HiConf	Variation_10697	chr12	89014390	89015075	685	Conrad et al. (2005)	Mendelian inconsistencies	
4290	chr12	89413737	89423145	9408	14	chr12	89413706	89423310	9604	HiConf	Variation_38272	chr12	89416448	89422039	5591	McCarroll et al. (2008)	Affymetrix Human SNP Array 6.0	
4291	chr12	89766243	89768889	2646	11	chr12	89766212	89768956	2744	HiConf								
4292	chr12	90182547	90183968	1421	26	chr12	90182516	90183986	1470	HiConf	Variation_4785	chr12	90057414	90201485	144071	Wong et al. (2007)	BAC Array CGH	
4293	chr12	91161077	91174405	13328	5	chr12	91161046	91174668	13622	HiConf	Variation_38273	chr12	91161059	91171445	10386	McCarroll et al. (2008)	Affymetrix Human SNP Array 6.0	
4294	chr12	91344578	91357224	12646	13	chr12	91344502	91357487	12985	HiConf								
4295	chr12	92028279	92029504	1225	28	chr12	92028248	92029522	1274	HiConf	Variation_43869	chr12	92028424	92029514	1090	Bentley et al. (2008)	Illumina DNA sequencing	
4296	chr12	92309686	92310969	1283	20	chr12	92309655	92311321	1666	single	Variation_39111	chr12	92296330	92312517	16187	Wheeler et al. (2008)	Sequencing	
4297	chr12	92968832	92971382	2550	4	chr12	92967186	92971449	4263	HiConf	Variation_30609	chr12	92807138	93053349	246211	Perry et al. (2008)	Agilent Custom CGH Arrays	
4298	chr12	94534825	94536295	1470	44	chr12	94534794	94536313	1519	HiConf	Variation_43871	chr12	94534578	94536547	1969	Bentley et al. (2008)	Illumina DNA sequencing	
4299	chr12	94561236	94561971	735	33	chr12	94561205	94561989	784	HiConf								
4300	chr12	95591608	95596865	5257	4	chr12	95591577	95596967	5390	single								
4301	chr12	96516875	96517414	539	22	chr12	96516844	96517432	588	single	Variation_43873	chr12	96516384	96517699	1315	Bentley et al. (2008)	Illumina DNA sequencing	
4302	chr12	96962776	96964931	2155	8	chr12	96962744	96964949	2205	single								
4303	chr12	96965127	96967430	2303	1	chr12	96965096	96967448	2352	single								
4304	chr12	97194594	97196015	1421	25	chr12	97194563	97196033	1470	HiConf	Variation_10827	chr12	97194992	97195652	660	McCarroll et al. (2005)	Null genotypes	
4305	chr12	97483601	97484870	1269	14	chr12	97483516	97484888	1372	single	Variation_22732	chr12	97480519	97487678	7159	Korbel et al. (2007)	Paired End Mapping	
4306	chr12	98088403	98088803	400	4	chr12	98088372	98088813	441	single								
4307	chr12	98318164	98334236	16072	28	chr12	98318133	98334548	16415	HiConf	Variation_1928	chr12	98319623	98326639	7016	McCarroll et al. (2005)	Null genotypes	
4308	chr12	100626897	100631013	4116	4	chr12	100626866	100631080	4214	HiConf	Variation_39113	chr12	100626537	100631217	4680	Wheeler et al. (2008)	Sequencing	
4309	chr12	102363506	102370856	7350	3	chr12	102363475	102371021	7546	HiConf								
4310	chr12	102651626	102652655	1029	19	chr12	102651595	102652673	1078	single	Variation_47868	chr12	102638407	102822676	184269	Gusev et al. (2009)	SNP genotyping analysis	
4311	chr12	102684554	102693815	9261	4	chr12	102684523	102693980	9457	single	Variation_47868	chr12	102638407	102822676	184269	Gusev et al. (2009)	SNP genotyping analysis	
100715	chr12	103171946	103249391	77445	3	chr12	103171755	103250547	78792	single	Variation_5690	chr12	103220416	103225907	5491	Mills et al. (2006)	Sequence trace read mapping	
4312	chr12	103893727	103897451	3724	22	chr12	103893696	103897518	3822	HiConf	Variation_35035	chr12	103892254	103908916	16662	Kidd et al. (2008)	Paired End Mapping	
4313	chr12	104205416	104205676	260	13	chr12	104205385	104205679	294	single	Variation_43171	chr12	104205039	104205878	839	Wang et al. (2008)	Illumina DNA sequencing	
4314	chr12	107387476	107390416	2940	26	chr12	107387445	107390483	3038	HiConf	Variation_43838	chr12	107388012	107390369	2357	Bentley et al. (2008)	Illumina DNA sequencing	
4315	chr12	107765854	107766254	400	42	chr12	107765823	107766264	441	single	Variation_5312	chr12	107710583	108040548	329965	Simon-Sanchez et al. (2007)	Illumina HumanHap300 BeadChip	
4316						chr12	107816293	107827073	10780	single								
4317	chr12	109530902	109533382	2480	4	chr12	109530313	109533449	3136	single								
4318	chr12	109633636	109637409	3773	5	chr12	109633605	109637476	3871	HiConf								
4319	chr12	109711350	109723446	12096	3	chr12	109711319	109723961	12642	HiConf	Variation_38244	chr12	109718337	109720108	1771	McCarroll et al. (2008)	Affymetrix Human SNP Array 6.0	
4320	chr12	111958113	111962998	4885	9	chr12	111958067	111963114	5047	HiConf	Variation_35044	chr12	111950113	111976035	25922	Kidd et al. (2008)	Paired End Mapping	
4321	chr12	113784132	113809416	25284	13	chr12	113784101	113809924	25823	HiConf								
100716	chr12	114258232	114356722	98490	27	chr12	114258201	114358724	100523	HiConf								
4323	chr12	114576168	114580823	4655	3	chr12	114576137	114580890	4753	single								
4324	chr12	115174773	115176516	1743	3	chr12	115174476	115176534	2058	single								
4325	chr12	115369150	115375456	6306	2	chr12	115368663	115375572	6909	single								y
4326	chr12	118377392	118379548	2156	38	chr12	118377361	118379566	2205	single								
4327	chr12	118388172	118393366	5194	6	chr12	118388141	118393482	5341	single								
4328	chr12	120020264	120026291	6027	5	chr12	120020233	120026407	6174	HiConf	Variation_38248	chr12	120020329	120022886	2557	McCarroll et al. (2008)	Affymetrix Human SNP Array 6.0	
4329	chr12	120436911	120437450	539	27	chr12	120436880	120437468	588	HiConf								
100717	chr12	120695043	120764301	69258	2	chr12	120694302	120765621	71319	single								
4330	chr12	123062331	123065957	3626	6	chr12	123062300	123066024	3724	HiConf								
4331	chr12	123291553	123292337	784	17	chr12	123291522	123292355	833	single								
4332	chr12	123373824	123374363	539	39	chr12	123373793	123374381	588	single	Variation_9699	chr12	123306943	123442086	135143	Wang et al. (2007)	Illumina HumanHap550 BeadChip	
4333	chr12	125387038	125395172	8134	3	chr12	125387007	125395337	8330	HiConf	Variation_31904	chr12	125387274	125394874	7600	Perry et al. (2008)	Agilent Custom CGH Arrays	
4334	chr12	125873314	125884192	10878	16	chr12	125873283	125884406	11123	HiConf	Variation_4793	chr12	125862923	126017694	154771	Wong et al. (2007)	BAC Array CGH	
4335	chr12	126054663	126060837	6174	4	chr12	126054632	126060953	6321	single	Variation_31905	chr12	126054518	126151180	96662	Perry et al. (2008)	Agilent Custom CGH Arrays	
4336	chr12	126145068	126169519	24451	6	chr12	126145037	126170027	24990	HiConf	Variation_38252	chr12	126158640	126170328	11688	McCarroll et al. (2008)	Affymetrix Human SNP Array 6.0	
4337	chr12	126422261	126424907	2646	11	chr12	126422230	126424974	2744	HiConf								
100718	chr12	126593908	126647839	53931	27	chr12	126593877	126648733	54856	HiConf								
4339	chr12	126991004	126996982	5978	7	chr12	126990973	126997098	6125	HiConf	Variation_10406	chr12	126991688	126995596	3908	Wang et al. (2007)	Illumina HumanHap550 BeadChip	
4340	chr12	127377565	127377628	63	37	chr12	127377534	127377632	98	HiConf	Variation_6965	chr12	127373650	127381167	7517	de Smith et al. (2007)	Agilent 185k CGH Arrays/Agilent Custom CGH Arrays	
4341	chr12	127758687	127759226	539	35	chr12	127758656	127759244	588	single	Variation_43194	chr12	127758429	127759347	918	Wang et al. (2008)	Illumina DNA sequencing	
4342	chr12	127969387	127970220	833	6	chr12	127969356	127970238	882	single								
4343	chr12	128239671	128246825	7154	20	chr12	128239640	128246941	7301	HiConf								
4344	chr12	128322628	128324784	2156	18	chr12	128322597	128324802	2205	HiConf								y
4345	chr12	128436798	128440121	3323	23	chr12	128436767	128440148	3381	HiConf	Variation_44995	chr12	128438738	128439179	441	Bentley et al. (2008)	Illumina DNA sequencing	y
4346	chr12	128553859	128557926	4067	14	chr12	128553828	128557993	4165	single	Variation_3901	chr12	128512417	129681529	1169112	Redon et al. (2006)	BAC Array CGH	
4347	chr12	128622508	128628137	5629	15	chr12	128622477	128628161	5684	HiConf	Variation_22644	chr12	128624064	128630378	6314	Korbel et al. (2007)	Paired End Mapping	
4348	chr12	128746772	128757405	10633	12	chr12	128746741	128757619	10878	single	Variation_9700	chr12	128578866	129296225	717359	Wang et al. (2007)	Illumina HumanHap550 BeadChip	
4349						chr12	128910744	128915301	4557	single								
4350	chr12	129077662	129078992	1330	1	chr12	129071513	129084841	13328	single	Variation_9700	chr12	128578866	129296225	717359	Wang et al. (2007)	Illumina HumanHap550 BeadChip	
4351	chr12	129442474	129444385	1911	45	chr12	129442443	129444403	1960	HiConf	Variation_31914	chr12	129443038	129444095	1057	Perry et al. (2008)	Agilent Custom CGH Arrays	y
4352	chr12	129674423	129713809	39386	43	chr12	129674360	129714148	39788	HiConf	Variation_35063	chr12	129687121	129718560	31439	Kidd et al. (2008)	Paired End Mapping	
4353	chr12	129932768	129933258	490	43	chr12	129932737	129933276	539	HiConf								
4354	chr12	130080111	130081189	1078	31	chr12	130080080	130081207	1127	single								y
4355	chr12	130367323	130376960	9637	19	chr12	130367171	130381528	14357	HiConf	Variation_10409	chr12	130366012	130380887	14875	Wang et al. (2007)	Illumina HumanHap550 BeadChip	
4356	chr12	130694473	130710545	16072	12	chr12	130694442	130710857	16415	HiConf	Variation_1940	chr12	130696856	130701708	4852	McCarroll et al. (2005)	Mendelian inconsistencies/Null genotypes	

locus_id	chrom	start	end	length	Yoruba w/ event	putative chr	putative start	putative end	putative len	putative type	variation_id	DGV_chr	DGV_start	DGV_end	DGV_len	Reference	Method/platform	complex
4357	chr12	131086081	131086865	784	11	chr12	131086050	131086883	833	single								
4358	chr12	131384099	131386794	2695	25	chr12	131384068	131386861	2793	HiConf								
4359	chr12	131467693	131470902	3209	47	chr12	131467662	131470945	3283	single	Variation_45011	chr12	131469216	131470064	848	Bentley et al. (2008)	Illumina DNA sequencing	
4360	chr12	131818631	131820297	1666	17	chr12	131818600	131820315	1715	HiConf								
4361	chr12	131953038	131974941	21903	10	chr12	131953007	131975400	22393	HiConf								
4362	chr12	132004929	132044119	39190	13	chr12	132004898	132045127	40229	HiConf	Variation_30625	chr12	132039744	132043113	3369	Perry et al. (2008)	Agilent Custom CGH Arrays	y
100719	chr12	132145020	132288853	143833	47	chr12	132144965	132289000	144035	HiConf	Variation_3002	chr12	132149424	132244186	94762	Redon et al. (2006)	Affymetrix 500K EA SNP Mapping Array	
100720	chr13	18038249	18271309	233060	9	chr13	17918100	18371644	453544	HiConf	Variation_8743	chr13	17960300	18252675	292375	Pinto et al. (2007)	Affymetrix 500K SNP Mapping Array	
4368	chr13	18376134	18377066	932	14	chr13	18376103	18377083	980	HiConf								
4369	chr13	18589039	18591734	2695	43	chr13	18589008	18591801	2793	HiConf								
4370	chr13	18622212	18626573	4361	16	chr13	18622181	18626640	4459	single								
4371	chr13	18825513	18825913	400	60	chr13	18825482	18825923	441	single								
100721	chr13	18843631	19027001	183370	4	chr13	18843563	19030743	187180	HiConf								
4372	chr13	19358702	19368287	9585	13	chr13	19358259	19368598	10339	HiConf	Variation_1942	chr13	19361867	19368071	6204	McCarroll et al. (2005)	Mendelian inconsistencies	
4373	chr13	19548312	19549733	1421	19	chr13	19548281	19549751	1470	HiConf	Variation_43876	chr13	19548108	19549785	1677	Bentley et al. (2008)	Illumina DNA sequencing	
4374	chr13	19662090	19662297	207	11	chr13	19662059	19662304	245	single								
4375	chr13	19864950	19865636	686	31	chr13	19864919	19865654	735	single	Variation_9214	chr13	19690082	20018804	328722	Pinto et al. (2007)	Affymetrix 500K SNP Mapping Array	y
4376	chr13	20432762	20433072	310	24	chr13	20432731	20433074	343	single								
4377	chr13	20436682	20438593	1911	8	chr13	20436651	20438611	1960	single	Variation_30632	chr13	20436712	20446049	9337	Perry et al. (2008)	Agilent Custom CGH Arrays	
4378	chr13	20578488	20579811	1323	31	chr13	20578457	20579829	1372	single								
100722						chr13	20732611	20811991	79380	single								
4379	chr13	20850046	20854309	4263	9	chr13	20850015	20854376	4361	single								
4380	chr13	20902721	20907229	4508	4	chr13	20902690	20907296	4606	single								
4381	chr13	21316327	21320152	3825	21	chr13	21315956	21320219	4263	HiConf								
4382	chr13	22298829	22318282	19453	14	chr13	22298798	22318692	19894	HiConf	Variation_9707	chr13	22300993	22310923	9930	Wang et al. (2007)	Illumina HumanHap550 BeadChip	
4383	chr13	23187640	23187748	108	35	chr13	23187609	23187756	147	single	Variation_4899	chr13	23106479	23257571	151092	Wong et al. (2007)	BAC Array CGH	
4384	chr13	23353162	23367813	14651	1	chr13	23353131	23368125	14994	single	Variation_9216	chr13	22454400	23865515	1411115	Pinto et al. (2007)	Affymetrix 500K SNP Mapping Array	
4385	chr13	23368365	23396625	28260	3	chr13	23368321	23397182	28861	single	Variation_9216	chr13	22454400	23865515	1411115	Pinto et al. (2007)	Affymetrix 500K SNP Mapping Array	
4386	chr13	23426662	23427984	1322	47	chr13	23426631	23428003	1372	single	Variation_9216	chr13	22454400	23865515	1411115	Pinto et al. (2007)	Affymetrix 500K SNP Mapping Array	y
4387	chr13	23796955	23801414	4459	22	chr13	23796924	23801481	4557	single	Variation_8747	chr13	23625026	23854056	229030	Pinto et al. (2007)	Affymetrix 500K SNP Mapping Array	
4388	chr13	23877413	23889663	12250	7	chr13	23877382	23889926	12544	single	Variation_8748	chr13	23829981	23906630	76649	Pinto et al. (2007)	Affymetrix 500K SNP Mapping Array	
4389	chr13	24041759	24071208	29449	1	chr13	24041728	24071814	30086	HiConf	Variation_22877	chr13	24057990	24075240	17250	Korbel et al. (2007)	Paired End Mapping	
100725	chr13	24415561	24434612	19051	1	chr13	24402319	24462418	60099	HiConf								
4392	chr13	24470019	24477957	7938	3	chr13	24469988	24478122	8134	single	Variation_10413	chr13	24469233	24477336	8103	Wang et al. (2007)	Illumina HumanHap550 BeadChip	
100726	chr13	24544235	24559241	15006	1	chr13	24493655	24587319	93664	single								
4394	chr13	25667432	25671744	4312	8	chr13	25667401	25671811	4410	HiConf								
4395	chr13	26536937	26537819	882	42	chr13	26536906	26537837	931	single								
4396	chr13	27592201	27593867	1666	18	chr13	27592170	27593885	1715	HiConf	Variation_5702	chr13	27588116	27594207	6091	Mills et al. (2006)	Sequence trace read mapping	
4397	chr13	28649131	28649338	207	22	chr13	28649100	28649345	245	single								
4398	chr13	29020638	29021386	748	1	chr13	29020471	29022186	1715	single								y
4399	chr13	29022364	29039382	17018	34	chr13	29022333	29039532	17199	HiConf								
4400	chr13	29039710	29047697	7987	2	chr13	29039679	29047862	8183	single								
100727						chr13	29718942	29796656	77714	HiConf								
4401	chr13	30110997	30113153	2156	2	chr13	30110966	30113171	2205	single								
4402	chr13	30324245	30324505	260	46	chr13	30324214	30324508	294	HiConf	Variation_42701	chr13	30324137	30324662	525	Wang et al. (2008)	Illumina DNA sequencing	
4403	chr13	31050131	31050441	310	14	chr13	31050100	31050443	343	single								y
4404	chr13	31426157	31429538	3381	1	chr13	31426126	31429605	3479	single	Variation_5704	chr13	31425584	31426794	1210	Mills et al. (2006)	Sequence trace read mapping	
4405	chr13	31429685	31437868	8183	58	chr13	31429654	31438033	8379	HiConf	Variation_37494	chr13	31430427	31436698	6271	Cooper et al. (2008)	Illumina Human 1M BeadChip	
4406	chr13	31569972	31572177	2205	4	chr13	31569941	31572195	2254	HiConf								
4407	chr13	32037824	32038224	400	7	chr13	32037793	32038234	441	HiConf								
4408	chr13	33030907	33042912	12005	31	chr13	33030876	33043175	12299	HiConf	Variation_7006	chr13	33033770	33042587	8817	de Smith et al. (2007)	Agilent 185k CGH Arrays/Agilent Custom CGH Arrays	
4409	chr13	34394479	34401780	7301	6	chr13	34394448	34401945	7497	HiConf								
100728	chr13	34567351	34603219	35868	7	chr13	34567320	34603923	36603	single	Variation_7009	chr13	34588252	34598671	10419	de Smith et al. (2007)	Agilent 185k CGH Arrays/Agilent Custom CGH Arrays	
4410	chr13	35200382	35202832	2450	4	chr13	35200351	35202899	2548	single								
100729	chr13	35587370	35632464	45094	6	chr13	35587206	35633364	46158	single								
4411	chr13	36770508	36772981	2473	3	chr13	36770507	36773055	2548	single	Variation_39129	chr13	36769056	36781282	12226	Wheeler et al. (2008)	Sequencing	
4412	chr13	36967396	37017524	50128	13	chr13	36967340	37018643	51303	HiConf	Variation_29936	chr13	36968549	37017097	48548	Jakobsson et al. (2008)	Illumina HumanHap550 BeadChip	
4413	chr13	37323552	37329873	6321	2	chr13	37323521	37329989	6468	single								
4414	chr13	37955376	37958151	2775	26	chr13	37955376	37958218	2842	HiConf	Variation_43880	chr13	37955323	37958201	2878	Bentley et al. (2008)	Illumina DNA sequencing	
4415	chr13	38426297	38428355	2058	7	chr13	38426266	38428373	2107	HiConf								
4416	chr13	38644275	38649884	5609	65	chr13	38644218	38650000	5782	HiConf	Variation_37958	chr13	38645763	38649136	3373	McCarroll et al. (2008)	Affymetrix Human SNP Array 6.0	
4417	chr13	38832605	38833193	588	81	chr13	38832574	38833211	637	HiConf	Variation_5707	chr13	38832219	38833515	1296	Mills et al. (2006)	Sequence trace read mapping	
4418	chr13	38847942	38848922	980	2	chr13	38847911	38848940	1029	HiConf								
100731	chr13	40421895	40428527	6632	2	chr13	40356866	40441538	84672	single								
4419	chr13	40901385	40905795	4410	9	chr13	40901354	40905862	4508	HiConf								
4420	chr13	40921524	40923533	2009	25	chr13	40921493	40923551	2058	single								
100732						chr13	41105635	41145154	39519	HiConf								
100733	chr13	41357545	41373567	16022	2	chr13	41334514	41384543	50029	single	Variation_9217	chr13	41291824	41480595	188771	Pinto et al. (2007)	Affymetrix 500K SNP Mapping Array	
4422	chr13	41540561	41545343	4782	1	chr13	41540510	41545459	4949	single								
100734	chr13	42098529	42126645	28116	5	chr13	42098424	42127212	28788	HiConf	Variation_3012	chr13	42098764	42130996	32232	Redon et al. (2006)	Affymetrix 500K EA SNP Mapping Array	
4424	chr13	42435134	42437584	2450	9	chr13	42435103	42437651	2548	single	Variation_7014	chr13	42280242	42771921	491679	de Smith et al. (2007)	Agilent 185k CGH Arrays/Agilent Custom CGH Arrays	
4425	chr13	42496874	42507605	10731	5	chr13	42496843	42507819	10976	HiConf	Variation_9710	chr13	42501093	42505143	4050	Wang et al. (2007)	Illumina HumanHap550 BeadChip	
4426	chr13	43270927	43285627	14700	14	chr13	43270896	43285939	15043	HiConf	Variation_45170	chr13	43278326	43278656	330	Bentley et al. (2008)	Illumina DNA sequencing	
100735	chr13	45483060	45561408	78348	2	chr13	45482830	45563018	80188	single								
4427	chr13	45882152	45931137	48985	4	chr13	45882106	45932135	50029	HiConf	Variation_35165	chr13	45918884	45946900	28016	Kidd et al. (2008)	Paired End Mapping	
4428	chr13	46877572	46877832	260	64	chr13	46877541	46877835	294	HiConf								
4429	chr13	47679428	47683680	4252	21	chr13	47672174	47683836	11662	HiConf	Variation_35167	chr13	47664511	47688173	23662	Kidd et al. (2008)	Paired End Mapping	
4430	chr13	48431607	48435037	3430	27	chr13	48431576	48435104	3528	HiConf	Variation_35168	chr13	48424762	48437669	12907	Kidd et al. (2008)	Paired End Mapping	
4431	chr13	49373289	49375028	1739	18	chr13	49373258	49375218	1960	single								
4432	chr13	49411215	49412685	1470	7	chr13	49411184	49412703	1519	HiConf	Variation_43882	chr13	49411108	49412930	1822	Bentley et al. (2008)	Illumina DNA sequencing	y
4433	chr13	49967335	49973898	6563	66	chr13	49967334	49973900	6566	HiConf	Variation_23091	chr13	49967295	49973805	6510	Korbel et al. (2007)	Paired End Mapping	
4434	chr13	50342607	50344273	1666	21	chr13	50342576	50344291	1715	HiConf								
4435	chr13	51780414	51793056	12642	12	chr13	51780383	51793319	12936	HiConf	Variation_38866	chr13	51756793	51788011	31218	McCarroll et al. (2008)	Affymetrix Human SNP Array 6.0	
4436	chr13	51926140	51926347	207	7	chr13	51926109	51926354	245	single								
4437	chr13	53132863	53138106	5243	17	chr13	53132832	53138222	5390	HiConf								
100737	chr13	53682900	53719589	36689	1	chr13	53682857	53720342	37485	single	Variation_10419	chr13	53686843	53710499	23656	Wang et al. (2007)	Illumina HumanHap550 BeadChip	

locus_id	chrom	start	end	length	Yoruba w/ event	putative chr	putative start	putative end	putative len	putative type	variation_id	DGV_chr	DGV_start	DGV_end	DGV_len	Reference	Method/platform	complex
4439	chr13	54550937	54556901	5964	12	chr13	54549912	54557017	7105	single								
4440						chr13	54839306	54839355	49	HiConf								y
4441	chr13	56610638	56787610	176972	50	chr13	56610607	56788183	177576	HiConf	Variation_4805	chr13	56601346	56773819	172473	Wong et al. (2007)	BAC Array CGH	
4442	chr13	56807863	56809431	1568	5	chr13	56807832	56809449	1617	HiConf	Variation_31932	chr13	56807761	56809552	1791	Perry et al. (2008)	Agilent Custom CGH Arrays	
4443	chr13	56879256	56881608	2352	3	chr13	56879225	56881626	2401	single	Variation_29939	chr13	56743090	56938460	195370	Jakobsson et al. (2008)	Illumina HumanHap550 BeadChip	
4444	chr13	57348921	57352302	3381	5	chr13	57348890	57352369	3479	single								
4445						chr13	57942084	57945073	2989	single								
4446	chr13	57945349	57974063	28714	1	chr13	57945318	57974620	29302	single								
4447	chr13	58289231	58293445	4214	8	chr13	58289200	58293512	4312	single								
4448	chr13	60359285	60360265	980	14	chr13	60359254	60360283	1029	HiConf								
4449	chr13	60404147	60405051	904	5	chr13	60404089	60405069	980	single								
4450	chr13	60451111	60451219	108	12	chr13	60451080	60451227	147	single	Variation_45187	chr13	60451038	60451867	829	Bentley et al. (2008)	Illumina DNA sequencing	
4451	chr13	60610018	60616633	6615	3	chr13	60609987	60616749	6762	HiConf	Variation_38284	chr13	60611470	60616344	4874	McCarroll et al. (2008)	Affymetrix Human SNP Array 6.0	
4452	chr13	60691505	60700178	8673	18	chr13	60691474	60700343	8869	HiConf								
4453	chr13	61186650	61188904	2254	1	chr13	61186619	61188922	2303	single								
100739	chr13	63186651	63327514	140863	8	chr13	63186407	63330394	143987	single	Variation_3017	chr13	63201508	63285508	84000	Redon et al. (2006)	Affymetrix 500K EA SNP Mapping Array	
4455	chr13	63755939	63759738	3799	2	chr13	63755885	63759805	3920	HiConf								
4456	chr13	64240575	64242388	1813	9	chr13	64240544	64242406	1862	HiConf	Variation_0312	chr13	64056794	65087468	1030674	Sebat et al. (2004)	ROMA	
100740	chr13	64382504	64437335	54831	3	chr13	64382473	64438455	55982	single	Variation_31937	chr13	64420002	64428574	8572	Perry et al. (2008)	Agilent Custom CGH Arrays	
4458	chr13	64535947	64536154	207	38	chr13	64535916	64536161	245	HiConf	Variation_42621	chr13	64535763	64536275	512	Wang et al. (2008)	Illumina DNA sequencing	
4459	chr13	65090774	65105131	14357	2	chr13	65090743	65105394	14651	HiConf	Variation_38286	chr13	65094528	65103708	9180	McCarroll et al. (2008)	Affymetrix Human SNP Array 6.0	
4460	chr13	65352140	65354247	2107	8	chr13	65352109	65354265	2156	HiConf								
4461	chr13	65619435	65621934	2499	16	chr13	65619404	65622001	2597	single								
4462	chr13	66073126	66075331	2205	6	chr13	66073095	66075349	2254	HiConf	Variation_5426	chr13	65939891	66193481	253590	Simon-Sanchez et al. (2007)	Illumina HumanHap300 BeadChip	
4463	chr13	66234091	66239579	5488	2	chr13	66234060	66239695	5635	HiConf								
4464	chr13	66944885	66952145	7260	5	chr13	66944854	66953135	8281	HiConf	Variation_38287	chr13	66945140	66954900	9760	McCarroll et al. (2008)	Affymetrix Human SNP Array 6.0	
4465	chr13	67061367	67070766	9399	3	chr13	67060298	67070980	10682	HiConf								
4466	chr13	67219775	67228130	8355	4	chr13	67219744	67228270	8526	HiConf								
4467	chr13	67875907	67876096	189	71	chr13	67875903	67876099	196	HiConf	Variation_39982	chr13	67875868	67876192	324	Wheeler et al. (2008)	Sequencing	
4468	chr13	68097561	68100893	3332	8	chr13	68097530	68100960	3430	HiConf	Variation_4480	chr13	68040160	68199579	159419	Wong et al. (2007)	BAC Array CGH	
4469	chr13	68142200	68166215	24015	3	chr13	68142169	68166669	24500	HiConf	Variation_7022	chr13	68138941	68163552	24611	de Smith et al. (2007)	Agilent 185k CGH Arrays/Agilent Custom CGH Arrays	
4470	chr13	68834962	68839715	4753	1	chr13	68834931	68839782	4851	single								
4471	chr13	69300070	69300707	637	27	chr13	69300039	69300725	686	HiConf	Variation_43886	chr13	69299965	69304875	4910	Bentley et al. (2008)	Illumina DNA sequencing	
4472	chr13	69646157	69648313	2156	2	chr13	69646126	69648331	2205	HiConf	Variation_34653	chr13	69642500	69670400	27900	Zogopoulos et al. (2007)	Affymetrix 500K and 100K SNP Mapping Arrays	y
4473	chr13	71375269	71378726	3457	32	chr13	71375238	71378913	3675	HiConf	Variation_39137	chr13	71375266	71378578	3312	Wheeler et al. (2008)	Sequencing	
4474	chr13	71420741	71422946	2205	1	chr13	71420710	71422964	2254	single								y
4475	chr13	71705744	71710282	4538	55	chr13	71705743	71710349	4606	HiConf	Variation_39138	chr13	71705623	71710359	4736	Wheeler et al. (2008)	Sequencing	
4476	chr13	71744092	71744402	310	46	chr13	71744061	71744404	343	HiConf	Variation_23034	chr13	71739535	71745246	5711	Korbel et al. (2007)	Paired End Mapping	
4477	chr13	74525675	74528027	2352	2	chr13	74525644	74528045	2401	single								
4478	chr13	75001955	75028415	26460	16	chr13	75001924	75028972	27048	HiConf	Variation_37791	chr13	75007535	75015769	8234	McCarroll et al. (2008)	Affymetrix Human SNP Array 6.0	
4479	chr13	75507194	75510771	3577	5	chr13	75507163	75510838	3675	HiConf	Variation_43888	chr13	75506827	75510757	3930	Bentley et al. (2008)	Illumina DNA sequencing	
100742						chr13	76442769	76507841	65072	single								
4480	chr13	76863710	76870864	7154	2	chr13	76863679	76870980	7301	HiConf								
4481	chr13	77069461	77069821	360	8	chr13	77069430	77069822	392	single								
4482	chr13	77354053	77358120	4067	23	chr13	77354022	77358187	4165	HiConf								
4483	chr13	77662998	77665595	2597	5	chr13	77662967	77665662	2695	HiConf								
4484	chr13	77896042	77896105	63	10	chr13	77896011	77896109	98	single	Variation_5718	chr13	77895642	77896751	1109	Mills et al. (2006)	Sequence trace read mapping	
4485	chr13	80449236	80468542	19306	11	chr13	80449205	80468952	19747	HiConf								
4486	chr13	80701978	80712709	10731	63	chr13	80701947	80712923	10976	HiConf	Variation_22838	chr13	80702843	80713407	10564	Korbel et al. (2007)	Paired End Mapping	
4487	chr13	81271995	81274298	2303	3	chr13	81271964	81274316	2352	single								
4488	chr13	82063658	82070605	6947	40	chr13	82063314	82070664	7350	HiConf	Variation_22776	chr13	82062793	82070760	7967	Korbel et al. (2007)	Paired End Mapping	
4489	chr13	82108033	82112590	4557	4	chr13	82108002	82112657	4655	HiConf	Variation_3916	chr13	82035430	82280653	245223	Redon et al. (2006)	BAC Array CGH	
4490	chr13	82319762	82321134	1372	25	chr13	82319731	82321152	1421	single								
4491	chr13	82947942	82953381	5439	7	chr13	82947911	82953497	5586	HiConf								
4492	chr13	83022569	83030213	7644	3	chr13	83022538	83030378	7840	HiConf	Variation_43891	chr13	83025645	83028126	2481	Bentley et al. (2008)	Illumina DNA sequencing	
4493	chr13	84174118	84180733	6615	7	chr13	84174087	84180849	6762	HiConf								
4494	chr13	84312690	84317511	4821	2	chr13	84312659	84317559	4900	single								
100743	chr13	84368004	84378253	10249	1	chr13	84356220	84414163	57943	single								
4495	chr13	84611100	84611786	686	6	chr13	84611069	84611804	735	single	Variation_0313	chr13	84446447	84867020	420573	Sebat et al. (2004)	ROMA	
4496	chr13	84698908	84701260	2352	2	chr13	84698877	84701278	2401	single	Variation_5428	chr13	84616436	84869540	253104	Simon-Sanchez et al. (2007)	Illumina HumanHap300 BeadChip	
4497	chr13	85571549	85573754	2205	57	chr13	85571518	85573772	2254	HiConf	Variation_43892	chr13	85571787	85574248	2461	Bentley et al. (2008)	Illumina DNA sequencing	
4498	chr13	86481577	86483292	1715	3	chr13	86481546	86483310	1764	HiConf								
4499	chr13	86628234	86635940	7706	5	chr13	86628203	86636092	7889	HiConf								
4500	chr13	86719423	86724274	4851	2	chr13	86719392	86724390	4998	single								
4501	chr13	88220097	88220832	735	28	chr13	88220066	88220850	784	HiConf	Variation_3920	chr13	88209082	88673975	464893	Redon et al. (2006)	BAC Array CGH	
100744	chr13	88795446	88843059	47613	3	chr13	88795106	88844008	48902	single								y
4502	chr13	88965387	88987045	21658	26	chr13	88965356	88987504	22148	HiConf								
4503	chr13	89151930	89153351	1421	4	chr13	89151899	89153369	1470	HiConf	Variation_5429	chr13	89078620	89597586	518966	Simon-Sanchez et al. (2007)	Illumina HumanHap300 BeadChip	
4504	chr13	89660844	89662657	1813	44	chr13	89660813	89662675	1862	HiConf	Variation_43893	chr13	89660856	89662832	1976	Bentley et al. (2008)	Illumina DNA sequencing	
4505	chr13	89709158	89710502	1344	7	chr13	89709127	89711969	2842	HiConf								
4506	chr13	90700134	90702339	2205	9	chr13	90700103	90702357	2254	HiConf								
4507	chr13	90708366	90709493	1127	7	chr13	90708335	90709511	1176	single								
4508	chr13	91184536	91190575	6039	25	chr13	91181577	91190740	9163	HiConf	Variation_37498	chr13	91184589	91190243	5654	Cooper et al. (2008)	Illumina Human 1M BeadChip	
4509	chr13	91807679	91816599	8920	8	chr13	91805641	91816813	11172	HiConf								
4510	chr13	91854987	91855123	136	12	chr13	91854984	91855131	147	single	Variation_40002	chr13	91854848	91855423	575	Wheeler et al. (2008)	Sequencing	
4511	chr13	92042587	92043812	1225	5	chr13	92042556	92043830	1274	HiConf								
4512	chr13	92260490	92269359	8869	6	chr13	92260459	92269524	9065	HiConf								
4513	chr13	93456825	93456951	126	3	chr13	93456794	93456990	196	single								
4514	chr13	93801442	93802275	833	4	chr13	93801411	93802293	882	single	Variation_47869	chr13	93704538	94190645	486107	Gusev et al. (2009)	SNP genotyping analysis	
4515	chr13	94819221	94821867	2646	34	chr13	94819190	94821934	2744	HiConf	Variation_35200	chr13	94805858	94828927	23069	Kidd et al. (2008)	Paired End Mapping	
4516	chr13	95686570	95687122	552	4	chr13	95686539	95687764	1225	single								
4517	chr13	95717979	95723761	5782	2	chr13	95717948	95723877	5929	HiConf								
4518	chr13	96901889	96906523	4634	6	chr13	96901837	96906590	4753	HiConf								
4519	chr13	97299748	97303178	3430	23	chr13	97299717	97303245	3528	single								
4520						chr13	97616600	97618119	1519	single								
100745	chr13	97700201	97740283	40082	2	chr13	97700170	97741109	40939	HiConf	Variation_10435	chr13	97711062	97740231	29169	Wang et al. (2007)	Illumina HumanHap550 BeadChip	

locus_id	chrom	start	end	length	Yoruba w/ event	putative chr	putative start	putative end	putative len	putative type	variation_id	DGV_chr	DGV_start	DGV_end	DGV_len	Reference	Method/platform	complex
4522	chr13	98555373	98562772	7399	19	chr13	98555342	98562937	7595	single								
4523	chr13	98901754	98932448	30694	5	chr13	98901723	98933083	31360	HiConf	Variation_48033	chr13	98901377	99086958	185581	Gusev et al. (2009)	SNP genotyping analysis	
4524	chr13	99501857	99526063	24206	10	chr13	99501826	99526571	24745	HiConf								
4525	chr13	99896650	99914633	17983	15	chr13	99896619	99914994	18375	HiConf								
4526	chr13	100674182	100674344	162	14	chr13	100674151	100674347	196	single								
4527	chr13	100690401	100694383	3982	49	chr13	100690370	100694437	4067	HiConf	Variation_5722	chr13	100692145	100694425	2280	Mills et al. (2006)	Sequence trace read mapping	
4528	chr13	101656632	101661679	5047	2	chr13	101656601	101661795	5194	HiConf								
4529	chr13	102397618	102401285	3667	4	chr13	102397579	102401352	3773	HiConf								
4530	chr13	102889932	102890222	290	32	chr13	102889931	102890225	294	HiConf	Variation_45107	chr13	102889769	102890247	478	Bentley et al. (2008)	Illumina DNA sequencing	
4531	chr13	103393976	103396720	2744	10	chr13	103393945	103396787	2842	HiConf								
4532	chr13	103404413	103405491	1078	10	chr13	103404382	103405509	1127	single								
4533	chr13	103605607	103607910	2303	4	chr13	103605576	103607928	2352	HiConf	Variation_22570	chr13	103605412	103610830	5418	Korbel et al. (2007)	Paired End Mapping	
4534	chr13	105247475	105247759	284	39	chr13	105247468	105247762	294	HiConf	Variation_40013	chr13	105247324	105247922	598	Wheeler et al. (2008)	Sequencing	
4535	chr13	107358860	107359350	490	23	chr13	107358829	107359368	539	single	Variation_4817	chr13	107309198	107481871	172673	Wong et al. (2007)	BAC Array CGH	
4536	chr13	107659475	107669177	9702	20	chr13	107659444	107669391	9947	single								
4537	chr13	107745029	107749635	4606	30	chr13	107744998	107749702	4704	HiConf								
4538	chr13	108159777	108160365	588	57	chr13	108159734	108160371	637	HiConf	Variation_40015	chr13	108159746	108160438	692	Wheeler et al. (2008)	Sequencing	
100747	chr13	109191493	109213437	21944	3	chr13	109191454	109213896	22442	single								
4539	chr13	109648238	109662412	14174	26	chr13	109648207	109662662	14455	HiConf								
4540	chr13	109705078	109714437	9359	16	chr13	109705047	109714602	9555	HiConf								
4541	chr13	110760440	110761420	980	19	chr13	110760409	110761438	1029	single								
4542	chr13	111008576	111018523	9947	18	chr13	111008545	111018737	10192	HiConf	Variation_42071	chr13	111018199	111018639	440	Wang et al. (2008)	Illumina DNA sequencing	
4543	chr13	111137985	111138818	833	34	chr13	111137954	111138836	882	single	Variation_3926	chr13	110872855	111151995	279140	Redon et al. (2006)	BAC Array CGH	
4544	chr13	111717410	111717473	63	44	chr13	111717379	111717477	98	single	Variation_43227	chr13	111717422	111718473	1051	Wang et al. (2008)	Illumina DNA sequencing	
4545	chr13	111850862	111851482	620	28	chr13	111850855	111851492	637	single	Variation_25142	chr13	111851141	111851592	451	Levy et al. (2007)	Sequencing	
4546	chr13	111992153	112021521	29368	32	chr13	111992122	112021522	29400	single	Variation_35147	chr13	112011370	112012860	1490	Kidd et al. (2008)	Paired End Mapping	
4547	chr13	112312172	112315063	2891	13	chr13	112312141	112315130	2989	HiConf								y
4548	chr13	112488278	112493175	4897	65	chr13	112488247	112493245	4998	HiConf	Variation_25155	chr13	112488264	112488633	369	Levy et al. (2007)	Sequencing	
4549	chr13	112498176	112517087	18911	52	chr13	112498145	112517353	19208	HiConf	Variation_25157	chr13	112503577	112503728	151	Levy et al. (2007)	Sequencing	
4550	chr13	112547029	112548156	1127	62	chr13	112546998	112548174	1176	HiConf	Variation_43202	chr13	112547413	112548367	954	Wang et al. (2008)	Illumina DNA sequencing	
4551	chr13	112814577	112815402	825	3	chr13	112813754	112815420	1666	HiConf	Variation_11703	chr13	112814875	112815362	487	Mills et al. (2006)	Sequence trace read mapping	
4552	chr13	112833483	112838628	5145	9	chr13	112833452	112838744	5292	single	Variation_4819	chr13	112775902	112852253	76351	Wong et al. (2007)	BAC Array CGH	
100750	chr13	112907890	112965060	57170	9	chr13	112907859	112966193	58334	HiConf								
4555	chr13	113620080	113621305	1225	37	chr13	113620049	113621323	1274	HiConf								
4556	chr13	113856505	113857142	637	30	chr13	113856474	113857160	686	HiConf	Variation_45137	chr13	113856440	113856567	127	Bentley et al. (2008)	Illumina DNA sequencing	
4557	chr13	114124305	114125155	850	1	chr13	114117644	114126170	8526	single								
100751	chr14	18071109	18103988	32879	14	chr14	18071078	18104643	33565	HiConf								
4559	chr14	18346342	18371479	25137	7	chr14	18346311	18371987	25676	single	Variation_7028	chr14	18187183	19494445	1307262	de Smith et al. (2007)	Agilent 185k CGH Arrays/Agilent Custom CGH Arrays	
4560	chr14	18426947	18566254	139307	14	chr14	18426916	18569114	142198	HiConf	Variation_38838	chr14	18521076	18539520	18444	McCarroll et al. (2008)	Affymetrix Human SNP Array 6.0	
4561	chr14	18569316	18569601	285	19	chr14	18569310	18569604	294	single	Variation_2174	chr14	18544515	18732465	187950	Locke et al. (2006)	BAC Array CGH	
4562	chr14	18580366	18620665	40299	22	chr14	18580335	18621446	41111	HiConf	Variation_2174	chr14	18544515	18732465	187950	Locke et al. (2006)	BAC Array CGH	
4563	chr14	18698113	18704336	6223	27	chr14	18698082	18704452	6370	single	Variation_4823	chr14	18672780	18819775	146995	Wong et al. (2007)	BAC Array CGH	
4564	chr14	18826052	18831834	5782	14	chr14	18826021	18831950	5929	single	Variation_30646	chr14	18824823	18850824	26001	Perry et al. (2008)	Agilent Custom CGH Arrays	
4565	chr14	18862202	18907177	44975	24	chr14	18862134	18908194	46060	HiConf	Variation_33268	chr14	18873704	18874433	729	Perry et al. (2008)	Agilent Custom CGH Arrays	
4566	chr14	18979716	18987556	7840	31	chr14	18979685	18987721	8036	HiConf	Variation_4822	chr14	18662310	18987252	324942	Wong et al. (2007)	BAC Array CGH	
4567	chr14	18990398	19028360	37962	16	chr14	18990367	19029126	38759	single	Variation_37621	chr14	18740757	19400957	660200	Cooper et al. (2008)	Illumina Human 1M BeadChip	
4568	chr14	19092220	19132400	40180	18	chr14	19092189	19133202	41013	HiConf	Variation_37621	chr14	18740757	19400957	660200	Cooper et al. (2008)	Illumina Human 1M BeadChip	
4569	chr14	19148619	19216876	68257	17	chr14	19148588	19218266	69678	single	Variation_33269	chr14	19195986	19196533	547	Perry et al. (2008)	Agilent Custom CGH Arrays	
4570	chr14	19222462	19489414	266952	33	chr14	19222431	19494871	272440	HiConf	Variation_30648	chr14	19247074	19494444	247370	Perry et al. (2008)	Agilent Custom CGH Arrays	
4571	chr14	19535140	19553457	18317	24	chr14	19535100	19553818	18718	single	Variation_2175	chr14	19440982	19574860	133878	Locke et al. (2006)	BAC Array CGH	
4572	chr14	19562865	19566344	3479	3	chr14	19562834	19566411	3577	single	Variation_2175	chr14	19440982	19574860	133878	Locke et al. (2006)	BAC Array CGH	
4573	chr14	19590060	19610222	20162	8	chr14	19590029	19610560	20531	HiConf	Variation_30649	chr14	19590285	19610807	20522	Perry et al. (2008)	Agilent Custom CGH Arrays	y
4574	chr14	19621714	19624899	3185	39	chr14	19621683	19624966	3283	HiConf	Variation_23482	chr14	19620757	19625436	4679	Levy et al. (2007)	Sequencing	
4575	chr14	20219085	20245421	26336	17	chr14	20219042	20245943	26901	single								
4576	chr14	20418307	20494727	76420	10	chr14	20418276	20496431	78155	HiConf	Variation_31955	chr14	20414091	20492145	78054	Perry et al. (2008)	Agilent Custom CGH Arrays	
4577	chr14	20991411	20991811	400	15	chr14	20991380	20991821	441	single								
4578	chr14	21024217	21024437	220	45	chr14	21023916	21024455	539	HiConf	Variation_43899	chr14	21022353	21024500	2147	Bentley et al. (2008)	Illumina DNA sequencing	
4579	chr14	21096805	21107051	10246	15	chr14	21096338	21107265	10927	HiConf	Variation_5318	chr14	21001464	21239342	237878	Simon-Sanchez et al. (2007)	Illumina HumanHap300 BeadChip	
4580	chr14	21120085	21128226	8141	16	chr14	21120054	21128580	8526	HiConf	Variation_37792	chr14	21119983	21125597	5614	McCarroll et al. (2008)	Affymetrix Human SNP Array 6.0	
4581	chr14	21652715	21653401	686	22	chr14	21652684	21653419	735	HiConf	Variation_4829	chr14	21628027	21807284	179257	Wong et al. (2007)	BAC Array CGH	
4582	chr14	21951615	21952065	450	47	chr14	21951584	21952074	490	HiConf	Variation_11711	chr14	21951507	21952099	592	Mills et al. (2006)	Sequence trace read mapping	
4583	chr14	22164931	22168042	3111	25	chr14	22164930	22168115	3185	HiConf	Variation_39146	chr14	22164710	22168524	3814	Wheeler et al. (2008)	Sequencing	
4584	chr14	22992248	22992385	137	81	chr14	22992246	22992393	147	HiConf	Variation_45251	chr14	22992094	22992487	393	Bentley et al. (2008)	Illumina DNA sequencing	
100754	chr14	23491052	23592642	101590	9	chr14	23465170	23593623	128453	HiConf	Variation_7029	chr14	23499453	23574945	75492	de Smith et al. (2007)	Agilent 185k CGH Arrays/Agilent Custom CGH Arrays	
4586	chr14	24296265	24301067	4802	2	chr14	24296234	24301183	4949	HiConf								y
4587	chr14	24332329	24332819	490	46	chr14	24332298	24332837	539	single								
4588	chr14	25354194	25354431	237	18	chr14	25354193	25354438	245	single	Variation_40023	chr14	25353713	25354639	926	Wheeler et al. (2008)	Sequencing	
4589	chr14	25660829	25662005	1176	30	chr14	25660786	25662011	1225	HiConf	Variation_39148	chr14	25660798	25662231	1433	Wheeler et al. (2008)	Sequencing	y
4590	chr14	26134304	26139890	5586	22	chr14	26134273	26140006	5733	single								
4591	chr14	26540262	26542278	2016	5	chr14	26539895	26542296	2401	HiConf								
100755	chr14	27276258	27399974	123716	1	chr14	27276218	27402491	126273	HiConf	Variation_30655	chr14	27286696	27399142	112446	Perry et al. (2008)	Agilent Custom CGH Arrays	
4593	chr14	27736555	27743023	6468	10	chr14	27736524	27743139	6615	HiConf	Variation_22728	chr14	27736578	27743723	7145	Korbel et al. (2007)	Paired End Mapping	
4594	chr14	27939679	27940885	1206	31	chr14	27939678	27940903	1225	single	Variation_43903	chr14	27939602	27941911	2309	Bentley et al. (2008)	Illumina DNA sequencing	
4595	chr14	32023347	32023957	610	53	chr14	32023338	32023975	637	HiConf	Variation_33584	chr14	32023276	32023749	473	Perry et al. (2008)	Agilent Custom CGH Arrays	y
4596	chr14	33011650	33011857	207	26	chr14	33011619	33011864	245	single	Variation_47870	chr14	32905671	33260245	354574	Gusev et al. (2009)	SNP genotyping analysis	
4597	chr14	33583921	33584029	108	35	chr14	33583890	33584037	147	HiConf	Variation_45266	chr14	33583777	33584586	809	Bentley et al. (2008)	Illumina DNA sequencing	
4598	chr14	33738663	33745180	6517	30	chr14	33738632	33745296	6664	HiConf	Variation_47953	chr14	33550265	33908046	357781	Gusev et al. (2009)	SNP genotyping analysis	
4599	chr14	34082349	34097245	14896	3	chr14	34082318	34097557	15239	single	Variation_31960	chr14	34086445	34089057	2612	Perry et al. (2008)	Agilent Custom CGH Arrays	y
4600	chr14	34180888	34192599	11711	16	chr14	34180857	34192813	11956	HiConf	Variation_22785	chr14	34184561	34192732	8171	Korbel et al. (2007)	Paired End Mapping	
4601	chr14	34374389	34375763	1374	9	chr14	34374358	34375779	1421	HiConf	Variation_42020	chr14	34374996	34375426	430	Wang et al. (2008)	Illumina DNA sequencing	
4602	chr14	34772220	34772480	260	9	chr14	34772189	34772483	294	HiConf								
4603	chr14	35050834	35051194	360	6	chr14	35050803	35051195	392	single								
4604	chr14	35415051	35426027	10976	1	chr14	35415020	35426241	11221	single								
4605	chr14	35742330	35745066	2736	18	chr14	35742242	35745133	2891	HiConf	Variation_38297	chr14	35741780	35744784	3004	McCarroll et al. (2008)	Affymetrix Human SNP Array 6.0	
4606	chr14	36839873	36840657	784	51	chr14	36839842	36840675	833	HiConf								
100756	chr14	37124965	37139163	14198	1	chr14	37074209	37175688	101479	HiConf								

locus_id	chrom	start	end	length	Yoruba w/ event	putative chr	putative start	putative end	putative len	putative type	variation_id	DGV_chr	DGV_start	DGV_end	DGV_len	Reference	Method/platform	complex
4607	chr14	38074399	38074688	289	37	chr14	38074397	38074691	294	HiConf	Variation_40035	chr14	38074269	38074778	509	Wheeler et al. (2008)	Sequencing	
4608	chr14	38808130	38831478	23348	4	chr14	38789013	38832329	43316	single								
4609	chr14	39679178	39687165	7987	46	chr14	39679147	39687330	8183	HiConf	Variation_23164	chr14	39679507	39687925	8418	Korbel et al. (2007)	Paired End Mapping	
4610	chr14	39939662	39943043	3381	3	chr14	39939631	39943110	3479	HiConf	Variation_38298	chr14	39940733	39942282	1549	McCarroll et al. (2008)	Affymetrix Human SNP Array 6.0	
4611	chr14	40541872	40555837	13965	3	chr14	40541841	40556100	14259	HiConf	Variation_38299	chr14	40541848	40555277	13429	McCarroll et al. (2008)	Affymetrix Human SNP Array 6.0	
100757	chr14	40883085	40928556	45471	2	chr14	40866638	40929799	63161	HiConf	Variation_38300	chr14	40885215	40927856	42641	McCarroll et al. (2008)	Affymetrix Human SNP Array 6.0	
4613	chr14	42057316	42062743	5427	13	chr14	42057313	42062752	5439	HiConf	Variation_39151	chr14	42057276	42062682	5406	Wheeler et al. (2008)	Sequencing	y
4614	chr14	42251094	42254765	3671	8	chr14	42251059	42254832	3773	HiConf								
100758	chr14	44885649	45093825	208176	3	chr14	44885618	45098106	212488	HiConf	Variation_38304	chr14	44895806	45085468	189662	McCarroll et al. (2008)	Affymetrix Human SNP Array 6.0	
4616	chr14	45362933	45373174	10241	11	chr14	45362902	45373388	10486	HiConf	Variation_4834	chr14	45348804	45497520	148716	Wong et al. (2007)	BAC Array CGH	y
4617	chr14	45658256	45660657	2401	19	chr14	45658225	45660675	2450	HiConf								
4618	chr14	45671633	45678738	7105	8	chr14	45671602	45678854	7252	HiConf	Variation_10444	chr14	45671842	45677824	5982	Wang et al. (2007)	Illumina HumanHap550 BeadChip	
4619	chr14	46331222	46348176	16954	3	chr14	46331191	46348537	17346	single	Variation_10445	chr14	46317149	46436090	118941	Wang et al. (2007)	Illumina HumanHap550 BeadChip	
100759	chr14	47149669	47208754	59085	5	chr14	47149638	47239774	90136	HiConf	Variation_39152	chr14	47127218	47158069	30851	Wheeler et al. (2008)	Sequencing	
4621	chr14	47833949	47834248	299	6	chr14	47833580	47834266	686	single	Variation_42107	chr14	47833521	47833966	445	Wang et al. (2008)	Illumina DNA sequencing	
4622	chr14	48402912	48403432	520	4	chr14	48401441	48403450	2009	single								
4623	chr14	48451991	48454539	2548	15	chr14	48451960	48454606	2646	HiConf								
4624	chr14	49010199	49012845	2646	9	chr14	49010168	49012912	2744	HiConf								
4625	chr14	51285134	51309377	24243	9	chr14	51285091	51309885	24794	single								
4626	chr14	51875572	51875972	400	9	chr14	51875541	51875982	441	HiConf								
100760						chr14	53401156	53466130	64974	single								
4627	chr14	55272203	55275486	3283	2	chr14	55272172	55275553	3381	HiConf								
4628	chr14	56514647	56534737	20090	13	chr14	56514616	56535147	20531	HiConf								
4629	chr14	57207328	57212296	4968	1	chr14	57203409	57214238	10829	single	Variation_22870	chr14	57203060	57218526	15466	Korbel et al. (2007)	Paired End Mapping	
4630	chr14	57343874	57344903	1029	13	chr14	57343843	57344921	1078	HiConf								
4631	chr14	57766631	57766709	78	2	chr14	57766223	57766909	686	single								
4632	chr14	58110626	58111900	1274	4	chr14	58110595	58111918	1323	HiConf								
4633	chr14	59409322	59409910	588	9	chr14	59409291	59409928	637	single								
100762						chr14	59442538	59530591	88053	single								
4634	chr14	60220699	60226518	5819	8	chr14	60220290	60226611	6321	HiConf								
4635	chr14	60900343	60912740	12397	6	chr14	60900312	60913003	12691	HiConf	Variation_38305	chr14	60901611	60909127	7516	McCarroll et al. (2008)	Affymetrix Human SNP Array 6.0	
100763	chr14	61032521	61061774	29253	3	chr14	61032490	61062355	29865	HiConf								
4637	chr14	61723886	61734568	10682	3	chr14	61723855	61734782	10927	HiConf	Variation_38306	chr14	61725259	61734091	8832	McCarroll et al. (2008)	Affymetrix Human SNP Array 6.0	
4638	chr14	62131272	62135535	4263	7	chr14	62131241	62135602	4361	HiConf								
4639	chr14	62581379	62583717	2338	4	chr14	62571996	62588705	16709	single								
4640	chr14	63108381	63112938	4557	2	chr14	63108350	63113005	4655	single								
100764	chr14	64395342	64444566	49224	13	chr14	64395311	64445021	49710	single	Variation_23486	chr14	64430149	64433303	3154	Levy et al. (2007)	Sequencing	
4641	chr14	64500128	64509670	9542	4	chr14	64500097	64510142	10045	HiConf	Variation_7038	chr14	64491244	64504182	12938	de Smith et al. (2007)	Agilent 185k CGH Arrays/Agilent Custom CGH Arrays	
4642	chr14	64659329	64662465	3136	6	chr14	64659298	64662532	3234	single								
4643	chr14	65109051	65115470	6419	4	chr14	65109020	65115586	6566	HiConf								
100765						chr14	65146505	65274322	127817	single								
4644	chr14	67443950	67444210	260	6	chr14	67443919	67444213	294	single								
4645	chr14	67970957	67975365	4408	10	chr14	67964936	67975569	10633	HiConf	Variation_4838	chr14	67944394	68130025	185631	Wong et al. (2007)	BAC Array CGH	
4646	chr14	68879062	68883766	4704	2	chr14	68879031	68883833	4802	single								
4647	chr14	69086675	69092114	5439	22	chr14	69086644	69092230	5586	HiConf	Variation_31972	chr14	69087395	69092239	4844	Perry et al. (2008)	Agilent Custom CGH Arrays	
4648	chr14	69422717	69425706	2989	4	chr14	69422686	69425773	3087	HiConf								
4649	chr14	69642531	69644491	1960	5	chr14	69642500	69644509	2009	HiConf								
4650	chr14	69757730	69759053	1323	4	chr14	69757699	69759071	1372	single								
4651	chr14	72040493	72048529	8036	3	chr14	72040462	72048694	8232	single								
4652	chr14	72338756	72340226	1470	2	chr14	72338725	72340244	1519	single								y
100766	chr14	73058689	73149737	91048	20	chr14	73058658	73150459	91801	HiConf	Variation_30658	chr14	73064476	73122227	57751	Perry et al. (2008)	Agilent Custom CGH Arrays	
4654	chr14	73640784	73665455	24671	2	chr14	73640753	73665890	25137	HiConf	Variation_30659	chr14	73641855	73658431	16576	Perry et al. (2008)	Agilent Custom CGH Arrays	
4655	chr14	73994711	73996769	2058	3	chr14	73994680	73996787	2107	single								
4656						chr14	74469049	74472087	3038	single								
4657	chr14	74591090	74593442	2352	10	chr14	74591059	74593460	2401	single								
4658	chr14	74854465	74855739	1274	15	chr14	74854434	74855757	1323	HiConf								
4659	chr14	75160764	75163459	2695	9	chr14	75160733	75163526	2793	HiConf								
4660	chr14	76921726	76927836	6110	2	chr14	76921695	76928212	6517	single								
100767	chr14	77166065	77279451	113386	2	chr14	77166034	77281772	115738	single	Variation_3056	chr14	77174650	77282215	107565	Redon et al. (2006)	Affymetrix 500K EA SNP Mapping Array	
4662	chr14	77857479	77861399	3920	5	chr14	77857448	77861466	4018	single	Variation_38308	chr14	77856088	77861755	5667	McCarroll et al. (2008)	Affymetrix Human SNP Array 6.0	
4663	chr14	78624133	78624493	360	34	chr14	78624102	78624494	392	HiConf								
4664	chr14	79051070	79051430	360	25	chr14	79051039	79051431	392	HiConf								
4665	chr14	79168817	79169217	400	5	chr14	79168786	79169227	441	single								
4666	chr14	79295384	79300039	4655	12	chr14	79295353	79300106	4753	HiConf								
100768	chr14	80471262	80515950	44688	2	chr14	80471231	80516874	45643	single	Variation_0178	chr14	80497720	80650761	153041	Iafrate et al. (2004)	BAC Array CGH	
100769	chr14	80746979	80763414	16435	2	chr14	80740853	80815554	74701	single								
4667	chr14	80948007	80950212	2205	56	chr14	80947976	80950230	2254	HiConf								y
4668	chr14	81568814	81573214	4400	60	chr14	81568806	81573216	4410	HiConf	Variation_43572	chr14	81568706	81573198	4492	Wang et al. (2008)	Illumina DNA sequencing	
4669	chr14	82064325	82067118	2793	24	chr14	82064294	82067185	2891	HiConf	Variation_35277	chr14	82057311	82102846	45535	Kidd et al. (2008)	Paired End Mapping	
4670	chr14	82224261	82224996	735	15	chr14	82224230	82225014	784	HiConf	Variation_9247	chr14	82135791	82273410	137619	Pinto et al. (2007)	Affymetrix 500K SNP Mapping Array	
4671	chr14	82505276	82506844	1568	7	chr14	82505245	82506862	1617	HiConf								
4672	chr14	82868709	82869346	637	6	chr14	82868678	82869364	686	single								
4673						chr14	83710988	83712801	1813	single								
4674	chr14	84182497	84185584	3087	2	chr14	84182466	84185651	3185	HiConf	Variation_9248	chr14	83885083	84773800	888717	Pinto et al. (2007)	Affymetrix 500K SNP Mapping Array	
4675	chr14	84209080	84209212	132	52	chr14	84209073	84209220	147	HiConf	Variation_11751	chr14	84208899	84209378	479	Mills et al. (2006)	Sequence trace read mapping	
4676	chr14	84346549	84348264	1715	6	chr14	84346518	84348282	1764	HiConf	Variation_35278	chr14	84347252	84373770	26518	Kidd et al. (2008)	Paired End Mapping	
4677	chr14	84367031	84372002	4971	3	chr14	84367000	84372096	5096	single	Variation_23059	chr14	84365867	84372038	6171	Korbel et al. (2007)	Paired End Mapping	
4678	chr14	84474390	84476644	2254	4	chr14	84474359	84476662	2303	HiConf	Variation_9248	chr14	83885083	84773800	888717	Pinto et al. (2007)	Affymetrix 500K SNP Mapping Array	
4679	chr14	85219435	85223012	3577	3	chr14	85219404	85223079	3675	single	Variation_5761	chr14	85213147	85220123	6976	Mills et al. (2006)	Sequence trace read mapping	
4680	chr14	85638189	85641421	3232	12	chr14	85638158	85648301	10143	HiConf								
4681	chr14	85717631	85717961	330	4	chr14	85717391	85717979	588	single								
4682	chr14	85824585	85825222	637	5	chr14	85824554	85825240	686	HiConf								
4683	chr14	86122897	86123583	686	9	chr14	86122866	86123601	735	single								
4684	chr14	86435517	86460409	24892	5	chr14	86435486	86460917	25431	single								
100770	chr14	86530896	86566421	35525	2	chr14	86530865	86567125	36260	single								
4686	chr14	86836827	86852850	16023	10	chr14	86836796	86853162	16366	HiConf								

locus_id	chrom	start	end	length	Yoruba w/ event	putative chr	putative start	putative end	putative len	putative type	variation_id	DGV_chr	DGV_start	DGV_end	DGV_len	Reference	Method/platform	complex
4687	chr14	88414823	88428249	13426	2	chr14	88414792	88428512	13720	HiConf								
4688	chr14	88767819	88769779	1960	4	chr14	88767788	88769797	2009	HiConf								
4689	chr14	89212053	89216757	4704	4	chr14	89212022	89216824	4802	HiConf								
4690	chr14	91738052	91740453	2401	4	chr14	91738021	91740471	2450	single								
4691	chr14	91752311	91774348	22037	9	chr14	91752280	91774820	22540	HiConf								
4692	chr14	92909838	92924979	15141	8	chr14	92909807	92925291	15484	HiConf	Variation_38315	chr14	92909950	92924155	14205	McCarroll et al. (2008)	Affymetrix Human SNP Array 6.0	
100771						chr14	93254302	93310701	56399	HiConf								
4694	chr14	93385622	93392684	7062	2	chr14	93382167	93392898	10731	HiConf	Variation_3065	chr14	93382087	93414055	31968	Redon et al. (2006)	Affymetrix 500K EA SNP Mapping Array	
4695	chr14	93392982	93403170	10188	2	chr14	93392947	93403384	10437	HiConf	Variation_3065	chr14	93382087	93414055	31968	Redon et al. (2006)	Affymetrix 500K EA SNP Mapping Array	
4696	chr14	94055017	94061632	6615	7	chr14	94054986	94062336	7350	single								
4697	chr14	94485535	94488373	2838	8	chr14	94485108	94488440	3332	HiConf								
100773	chr14	94643314	94697358	54044	7	chr14	94643280	94698454	55174	single								
4699	chr14	95753896	95758943	5047	1	chr14	95753865	95759059	5194	single								
4700	chr14	95903836	95913538	9702	2	chr14	95903805	95913752	9947	single								
4701	chr14	97199788	97210127	10339	2	chr14	97199757	97210341	10584	HiConf	Variation_3067	chr14	97199755	97224576	24821	Redon et al. (2006)	Affymetrix 500K EA SNP Mapping Array	
4702	chr14	97223357	97225611	2254	5	chr14	97223326	97225629	2303	HiConf	Variation_31977	chr14	97223407	97225740	2333	Perry et al. (2008)	Agilent Custom CGH Arrays	
4703	chr14	97290879	97292545	1666	6	chr14	97290848	97292563	1715	HiConf								
4704	chr14	97447385	97450668	3283	1	chr14	97447354	97450735	3381	single	Variation_4846	chr14	97332528	97493465	160937	Wong et al. (2007)	BAC Array CGH	y
4705	chr14	99483041	99495442	12401	26	chr14	99483010	99495848	12838	HiConf								
4706	chr14	99710626	99714811	4185	7	chr14	99710223	99714878	4655	single								
4707	chr14	100218335	100221422	3087	18	chr14	100218304	100221489	3185	single	Variation_8776	chr14	100138042	100389000	250958	Pinto et al. (2007)	Affymetrix 500K SNP Mapping Array	
4708	chr14	100555847	100556337	490	15	chr14	100555816	100556355	539	single								y
4709	chr14	101244052	101255665	11613	22	chr14	101244021	101255879	11858	HiConf								
4710	chr14	101320394	101321423	1029	25	chr14	101320363	101321441	1078	single								
4711	chr14	101453184	101455683	2499	17	chr14	101453153	101455750	2597	single								
4712	chr14	101630809	101634141	3332	4	chr14	101630778	101634208	3430	single								
4713						chr14	101635090	101643959	8869	single								
4714	chr14	102412751	102413878	1127	27	chr14	102412720	102413896	1176	HiConf	Variation_0180	chr14	102319877	102479478	159601	Iafrate et al. (2004)	BAC Array CGH	
4715	chr14	102454205	102458419	4214	30	chr14	102454174	102458486	4312	HiConf	Variation_30662	chr14	102444175	102467664	23489	Perry et al. (2008)	Agilent Custom CGH Arrays	
4716	chr14	102616885	102617424	539	10	chr14	102616854	102617442	588	single	Variation_4848	chr14	102506700	102678934	172234	Wong et al. (2007)	BAC Array CGH	
4717	chr14	102729977	102733750	3773	5	chr14	102729946	102733817	3871	HiConf								
4718	chr14	102849880	102853555	3675	2	chr14	102849849	102853622	3773	single	Variation_31978	chr14	102759088	102908688	149600	Perry et al. (2008)	Agilent Custom CGH Arrays	y
4719	chr14	102862865	102864629	1764	15	chr14	102862834	102864647	1813	single	Variation_31978	chr14	102759088	102908688	149600	Perry et al. (2008)	Agilent Custom CGH Arrays	
4720	chr14	103634076	103634238	162	36	chr14	103634045	103634241	196	single	Variation_29961	chr14	103615037	103650825	35788	Jakobsson et al. (2008)	Illumina HumanHap550 BeadChip	
4721	chr14	103823216	103840660	17444	5	chr14	103823185	103841021	17836	HiConf								
4722	chr14	104033720	104035876	2156	14	chr14	104033689	104035894	2205	single	Variation_35202	chr14	104028535	104037753	9218	Kidd et al. (2008)	Paired End Mapping	
4723	chr14	105141855	105171402	29547	1	chr14	105141824	105172008	30184	HiConf	Variation_35210	chr14	105142320	105195403	53083	Kidd et al. (2008)	Paired End Mapping	
4724	chr14	105182665	105196637	13972	4	chr14	105172155	105197145	24990	HiConf	Variation_23230	chr14	105160389	105205439	45050	Korbel et al. (2007)	Paired End Mapping	
4725	chr14	105197293	105198205	912	6	chr14	105197292	105198223	931	single	Variation_23230	chr14	105160389	105205439	45050	Korbel et al. (2007)	Paired End Mapping	
4726	chr14	105198425	105205653	7228	11	chr14	105198419	105205769	7350	single	Variation_23230	chr14	105160389	105205439	45050	Korbel et al. (2007)	Paired End Mapping	y
4727	chr14	105206120	105206543	423	26	chr14	105206112	105206553	441	single	Variation_23242	chr14	105187250	105281514	94264	Korbel et al. (2007)	Paired End Mapping	y
4728	chr14	105270872	105395630	124758	25	chr14	105270841	105397702	126861	HiConf	Variation_22946	chr14	105280556	105399039	118483	Korbel et al. (2007)	Paired End Mapping	y
4729	chr14	105456974	105518273	61299	18	chr14	105456943	105519516	62573	HiConf	Variation_4853	chr14	105480026	105600462	120436	Wong et al. (2007)	BAC Array CGH	y
4730	chr14	105519718	105659687	139969	26	chr14	105519712	105662547	142835	HiConf	Variation_0772	chr14	105512458	105672588	160130	Sharp et al. (2005)	BAC Array CGH	y
4731	chr14	105662794	105990148	327354	33	chr14	105662792	105990210	327418	HiConf	Variation_4856	chr14	105779689	105964048	184359	Wong et al. (2007)	BAC Array CGH	
4732	chr14	105990259	106057028	66769	30	chr14	105990259	106058369	68110	HiConf	Variation_10455	chr14	105997070	106049356	52286	Wang et al. (2007)	Illumina HumanHap550 BeadChip	
4733	chr14	106058517	106064182	5665	9	chr14	106058516	106064298	5782	single	Variation_10457	chr14	106047919	106088226	40307	Wang et al. (2007)	Illumina HumanHap550 BeadChip	
4734	chr14	106154538	106167866	13328	17	chr14	106154507	106168129	13622	HiConf	Variation_45243	chr14	106163675	106163781	106	Bentley et al. (2008)	Illumina DNA sequencing	y
4735	chr14	106218336	106253510	35174	34	chr14	106218305	106253781	35476	HiConf	Variation_34503	chr14	106223861	106251269	27408	Zogopoulos et al. (2007)	Affymetrix 500K and 100K SNP Mapping Arrays	
4736	chr15	18413031	18457621	44590	14	chr15	18413000	18458521	45521	HiConf	Variation_38316	chr15	18415652	18454752	39100	McCarroll et al. (2008)	Affymetrix Human SNP Array 6.0	y
4737	chr15	18458624	18965208	506584	51	chr15	18458619	18967533	508914	HiConf	Variation_35284	chr15	18515594	19109786	594192	Kidd et al. (2008)	Paired End Mapping	
4738	chr15	19044935	19080920	35985	23	chr15	19044904	19081801	36897	HiConf	Variation_4866	chr15	18982301	19133581	151280	Wong et al. (2007)	BAC Array CGH	
4739	chr15	19210457	19296060	85603	17	chr15	19210426	19297793	87367	single	Variation_4868	chr15	19138466	19296842	158376	Wong et al. (2007)	BAC Array CGH	
4740	chr15	19298608	19317620	19012	7	chr15	19298577	19317981	19404	HiConf	Variation_0775	chr15	19203509	19367828	164319	Sharp et al. (2005)	BAC Array CGH	y
4741	chr15	19318943	19954789	635846	55	chr15	19318912	19955177	636265	HiConf	Variation_9742	chr15	19361783	19950423	588640	Wang et al. (2007)	Illumina HumanHap550 BeadChip	
4742	chr15	19955226	20062301	107075	37	chr15	19955226	20063271	108045	HiConf	Variation_4875	chr15	19970520	20094181	123661	Wong et al. (2007)	BAC Array CGH	y
4743	chr15	20067614	20296640	229026	18	chr15	20067583	20301313	233730	HiConf	Variation_4877	chr15	20218069	20299811	81742	Wong et al. (2007)	BAC Array CGH	
4744	chr15	20305460	20342259	36799	2	chr15	20305429	20343012	37583	single	Variation_2183	chr15	20197683	20382314	184631	Locke et al. (2006)	BAC Array CGH	
4745	chr15	20624303	20633221	8918	1	chr15	20624272	20633386	9114	single	Variation_38872	chr15	20232440	20646852	414412	McCarroll et al. (2008)	Affymetrix Human SNP Array 6.0	y
4746	chr15	20852057	21161196	309139	25	chr15	20852024	21167486	315462	HiConf	Variation_30674	chr15	20854468	21155028	300560	Perry et al. (2008)	Agilent Custom CGH Arrays	y
4747	chr15	21223279	21235528	12249	25	chr15	21223248	21235547	12299	HiConf	Variation_23494	chr15	21222917	21226724	3807	Levy et al. (2007)	Sequencing	
4748	chr15	21542073	21546875	4802	18	chr15	21542042	21546991	4949	HiConf	Variation_43554	chr15	21543150	21547137	3987	Wang et al. (2008)	Illumina DNA sequencing	
4749	chr15	21606557	21612535	5978	4	chr15	21606526	21612651	6125	HiConf	Variation_38318	chr15	21606534	21610186	3652	McCarroll et al. (2008)	Affymetrix Human SNP Array 6.0	
100776	chr15	21999782	22327347	327565	5	chr15	21999751	22334005	334254	HiConf	Variation_30678	chr15	22038902	22329388	290486	Perry et al. (2008)	Agilent Custom CGH Arrays	
4753	chr15	22589399	22591310	1911	6	chr15	22589368	22591328	1960	single	Variation_10469	chr15	22589304	22598069	8765	Wang et al. (2007)	Illumina HumanHap550 BeadChip	
4754	chr15	22617349	22635704	18355	3	chr15	22617347	22636065	18718	HiConf	Variation_31996	chr15	22617318	22633426	16108	Perry et al. (2008)	Agilent Custom CGH Arrays	
4755	chr15	22658270	22662213	3943	31	chr15	22658262	22662280	4018	HiConf	Variation_10471	chr15	22658176	22659931	1755	Wang et al. (2007)	Illumina HumanHap550 BeadChip	
4756	chr15	23058152	23064356	6204	4	chr15	23050507	23064619	14112	single	Variation_35298	chr15	23020941	23059757	38816	Kidd et al. (2008)	Paired End Mapping	
100777	chr15	23284905	23345910	61005	26	chr15	23284874	23347153	62279	HiConf	Variation_0183	chr15	23337062	23504662	167600	Iafrate et al. (2004)	BAC Array CGH	
4758	chr15	23482326	23483404	1078	44	chr15	23482295	23483422	1127	HiConf	Variation_45323	chr15	23482491	23483473	982	Bentley et al. (2008)	Illumina DNA sequencing	
4759	chr15	23873885	23875110	1225	7	chr15	23873854	23875128	1274	HiConf	Variation_47908	chr15	23802940	23972678	169738	Gusev et al. (2009)	SNP genotyping analysis	
4760	chr15	24300332	24303370	3038	2	chr15	24300301	24303437	3136	HiConf	Variation_43532	chr15	24300212	24303612	3400	Wang et al. (2008)	Illumina DNA sequencing	
4761	chr15	25294033	25312825	18792	11	chr15	25291522	25312935	21413	HiConf	Variation_25494	chr15	25306938	25307110	172	Levy et al. (2007)	Sequencing	
4762	chr15	25323011	25323844	833	8	chr15	25322980	25323862	882	single	Variation_7052	chr15	25301194	25457050	155856	de Smith et al. (2007)	Agilent 185k CGH Arrays/Agilent Custom CGH Arrays	
4763	chr15	25592098	25601184	9086	31	chr15	25592088	25601349	9261	HiConf	Variation_43910	chr15	25592463	25601461	8998	Bentley et al. (2008)	Illumina DNA sequencing	
4764	chr15	25602262	25609202	6940	1	chr15	25602231	25609287	7056	single	Variation_35299	chr15	25584526	25607437	22911	Kidd et al. (2008)	Paired End Mapping	
4765	chr15	26067468	26069379	1911	1	chr15	26067437	26069397	1960	HiConf								
4766	chr15	26111813	26116125	4312	10	chr15	26111782	26116192	4410	single	Variation_3954	chr15	26086062	26763830	677768	Redon et al. (2006)	BAC Array CGH	
4767	chr15	26135137	26136313	1176	10	chr15	26135106	26136331	1225	single	Variation_32000	chr15	26135591	26137216	1625	Perry et al. (2008)	Agilent Custom CGH Arrays	y
4768	chr15	26329030	26343190	14160	36	chr15	26328999	26343797	14798	HiConf	Variation_30685	chr15	26318572	26331265	12693	Perry et al. (2008)	Agilent Custom CGH Arrays	
4769	chr15	26538456	26542131	3675	38	chr15	26538425	26542198	3773	HiConf	Variation_30687	chr15	26538129	26542214	4085	Perry et al. (2008)	Agilent Custom CGH Arrays	
4770	chr15	26608183	26642317	34134	25	chr15	26608152	26643089	34937	HiConf	Variation_30689	chr15	26609197	26610755	1558	Perry et al. (2008)	Agilent Custom CGH Arrays	
4771	chr15	26643237	26708878	65641	9	chr15	26643236	26710219	66983	HiConf	Variation_30690	chr15	26634685	26698391	63706	Perry et al. (2008)	Agilent Custom CGH Arrays	
4772	chr15	26742100	26794726	52626	7	chr15	26742069	26795773	53704	single	Variation_32008	chr15	26757185	26780238	23053	Perry et al. (2008)	Agilent Custom CGH Arrays	
4773	chr15	26865531	26868471	2940	18	chr15	26865500	26868538	3038	single	Variation_9262	chr15	26768706	28766520	1997814	Pinto et al. (2007)	Affymetrix 500K SNP Mapping Array	y
4774	chr15	27188490	27208290	19800	13	chr15	27188459	27208451	19992	single	Variation_32012	chr15	27198522	27201948	3426	Perry et al. (2008)	Agilent Custom CGH Arrays	y

locus_id	chrom	start	end	length	Yoruba w/ event	putative chr	putative start	putative end	putative len	putative type	variation_id	DGV_chr	DGV_start	DGV_end	DGV_len	Reference	Method/platform	complex
4775	chr15	27208972	27212843	3871	15	chr15	27208941	27212910	3969	single	Variation_9262	chr15	26768706	28766520	1997814	Pinto et al. (2007)	Affymetrix 500K SNP Mapping Array	
4776	chr15	27688535	27690299	1764	11	chr15	27688504	27690317	1813	HiConf	Variation_37501	chr15	27688638	27690333	1695	Cooper et al. (2008)	Illumina Human 1M BeadChip	
4777	chr15	28158115	28279475	121360	7	chr15	28158071	28281943	123872	HiConf	Variation_4885	chr15	28106102	28294834	188732	Wong et al. (2007)	BAC Array CGH	
4778	chr15	28456953	28485618	28665	30	chr15	28456922	28486175	29253	single	Variation_37842	chr15	28377089	28536721	159632	McCarroll et al. (2008)	Affymetrix Human SNP Array 6.0	
4779	chr15	28638400	28680736	42336	24	chr15	28638369	28681587	43218	HiConf	Variation_30697	chr15	28645333	28654500	9167	Perry et al. (2008)	Agilent Custom CGH Arrays	
4780	chr15	28694750	28758156	63406	4	chr15	28694719	28759448	64729	HiConf	Variation_7056	chr15	28719165	28758851	39686	de Smith et al. (2007)	Agilent 185k CGH Arrays/Agilent Custom CGH Arrays	y
4781	chr15	30233105	30304335	71230	18	chr15	30233074	30304467	71393	HiConf	Variation_29639	chr15	30172759	30302218	129459	Jakobsson et al. (2008)	Illumina HumanHap550 BeadChip	
4782	chr15	30304664	30352714	48050	12	chr15	30304663	30353663	49000	single	Variation_0781	chr15	30179962	30339503	159541	Sharp et al. (2005)	BAC Array CGH	
4783	chr15	30353811	30365307	11496	12	chr15	30353810	30365521	11711	single	Variation_4892	chr15	30316092	30502594	186502	Wong et al. (2007)	BAC Array CGH	
4784	chr15	30365669	30418766	53097	28	chr15	30365668	30419862	54194	single	Variation_4892	chr15	30316092	30502594	186502	Wong et al. (2007)	BAC Array CGH	
4785	chr15	30420064	30420873	809	18	chr15	30420058	30420891	833	single	Variation_4892	chr15	30316092	30502594	186502	Wong et al. (2007)	BAC Array CGH	
4786	chr15	30453605	30455124	1519	17	chr15	30453574	30455142	1568	single	Variation_4894	chr15	30453948	30621426	167478	Wong et al. (2007)	BAC Array CGH	
4787	chr15	30468409	30518775	50366	36	chr15	30468274	30519822	51548	HiConf	Variation_30701	chr15	30499505	30520735	21230	Perry et al. (2008)	Agilent Custom CGH Arrays	
4788	chr15	30626036	30687482	61446	5	chr15	30626005	30688725	62720	HiConf	Variation_2191	chr15	30609233	30773871	164638	Locke et al. (2006)	BAC Array CGH	
4789	chr15	30691725	30711933	20208	2	chr15	30691273	30712343	21070	single	Variation_32019	chr15	30699095	30712376	13281	Perry et al. (2008)	Agilent Custom CGH Arrays	
4790	chr15	32375924	32379844	3920	2	chr15	32375893	32379911	4018	HiConf	Variation_3957	chr15	32330313	32775539	445226	Redon et al. (2006)	BAC Array CGH	y
4791	chr15	32462458	32496758	34300	13	chr15	32462427	32497462	35035	HiConf	Variation_32020	chr15	32469858	32483260	13402	Perry et al. (2008)	Agilent Custom CGH Arrays	
4792	chr15	32497863	32539786	41923	24	chr15	32497854	32540239	42385	single	Variation_32021	chr15	32495955	32506908	10953	Perry et al. (2008)	Agilent Custom CGH Arrays	
4793	chr15	32540680	32550560	9880	31	chr15	32540680	32550774	10094	single	Variation_43683	chr15	32528621	32556540	27919	Wang et al. (2008)	Illumina DNA sequencing	
4794	chr15	32550976	32589501	38525	25	chr15	32550970	32590317	39347	single	Variation_37540	chr15	32529325	32599965	70640	Cooper et al. (2008)	Illumina Human 1M BeadChip	y
4795	chr15	32590668	32598095	7427	32	chr15	32590660	32598451	7791	single	Variation_37540	chr15	32529325	32599965	70640	Cooper et al. (2008)	Illumina Human 1M BeadChip	
4796	chr15	32604901	32660089	55188	9	chr15	32604870	32661171	56301	HiConf	Variation_32023	chr15	32615284	32632382	17098	Perry et al. (2008)	Agilent Custom CGH Arrays	
4797	chr15	32984259	32985043	784	4	chr15	32984228	32985061	833	HiConf								
100782	chr15	33153656	33225045	71389	3	chr15	33153523	33226484	72961	single								
4798	chr15	33323682	33326867	3185	20	chr15	33323651	33326934	3283	single								
4799	chr15	33947354	33950098	2744	52	chr15	33947323	33950165	2842	HiConf	Variation_5776	chr15	33947423	33949507	2084	Mills et al. (2006)	Sequence trace read mapping	
4800	chr15	37159892	37160480	588	68	chr15	37159861	37160498	637	HiConf								
4801	chr15	37531722	37532063	341	50	chr15	37531722	37532065	343	HiConf	Variation_25515	chr15	37531683	37532152	469	Levy et al. (2007)	Sequencing	
4802	chr15	37931250	37933504	2254	17	chr15	37931219	37933522	2303	HiConf								
4803	chr15	39102713	39122489	19776	14	chr15	39102270	39122899	20629	HiConf								
4804	chr15	39634637	39635715	1078	18	chr15	39634606	39635733	1127	single								
4805	chr15	40178047	40178733	686	11	chr15	40178016	40178751	735	single	Variation_0785	chr15	40121667	40290274	168607	Sharp et al. (2005)	BAC Array CGH	
4806	chr15	40191499	40195148	3649	9	chr15	40191491	40195215	3724	HiConf	Variation_38326	chr15	40191008	40194976	3968	McCarroll et al. (2008)	Affymetrix Human SNP Array 6.0	
4807	chr15	40373704	40374243	539	18	chr15	40373673	40374261	588	single								
4808	chr15	40508986	40509679	693	5	chr15	40508619	40509697	1078	single								
4809	chr15	40776386	40781662	5276	4	chr15	40776355	40782039	5684	single								
4810	chr15	40950777	40962341	11564	2	chr15	40950746	40962555	11809	single								
100783						chr15	41222108	41308079	85971	single								
100784	chr15	41621274	41826922	205648	4	chr15	41620748	41831105	210357	HiConf	Variation_3079	chr15	41619215	41845926	226711	Redon et al. (2006)	Affymetrix 500K EA SNP Mapping Array	
4811	chr15	42919450	42932827	13377	6	chr15	42919419	42933090	13671	HiConf	Variation_32032	chr15	42919602	42932699	13097	Perry et al. (2008)	Agilent Custom CGH Arrays	
4812	chr15	43119370	43125668	6298	3	chr15	43119339	43126052	6713	single	Variation_32033	chr15	43122783	43127827	5044	Perry et al. (2008)	Agilent Custom CGH Arrays	
4813						chr15	43203129	43204844	1715	single								
4814	chr15	43206737	43207137	400	14	chr15	43206706	43207147	441	single	Variation_3961	chr15	42756160	43210134	453974	Redon et al. (2006)	BAC Array CGH	
100785	chr15	43236999	43240966	3967	1	chr15	43229173	43259871	30698	single								
4815	chr15	43993616	43994030	414	2	chr15	43993401	43994577	1176	single								
4816	chr15	44042432	44044637	2205	64	chr15	44042401	44044655	2254	HiConf	Variation_43912	chr15	44042373	44044785	2412	Bentley et al. (2008)	Illumina DNA sequencing	
4817	chr15	44389107	44396996	7889	1	chr15	44389076	44397161	8085	single								
4818	chr15	44648192	44648381	189	46	chr15	44648188	44648384	196	HiConf	Variation_40095	chr15	44647999	44648460	461	Wheeler et al. (2008)	Sequencing	
4819	chr15	44847110	44849462	2352	8	chr15	44847030	44849529	2499	single								
100786						chr15	45125228	45190202	64974	HiConf								
4822	chr15	46048220	46053735	5515	5	chr15	46048167	46053851	5684	HiConf								
4823	chr15	46127823	46130126	2303	2	chr15	46127792	46130144	2352	single								
4824	chr15	46477389	46488708	11319	1	chr15	46477358	46488922	11564	single	Variation_10484	chr15	46481503	46488520	7017	Wang et al. (2007)	Illumina HumanHap550 BeadChip	
4825	chr15	47332243	47335012	2769	15	chr15	47332212	47335103	2891	HiConf								
4826	chr15	47558819	47570334	11515	2	chr15	47558788	47570548	11760	HiConf	Variation_30712	chr15	47558891	47566666	7775	Perry et al. (2008)	Agilent Custom CGH Arrays	
4827	chr15	48955811	48957132	1321	3	chr15	48955778	48957150	1372	single	Variation_29644	chr15	48558160	48971875	413715	Jakobsson et al. (2008)	Illumina HumanHap550 BeadChip	
100787						chr15	49669414	49706630	37216	single								
4828	chr15	51987586	51989350	1764	3	chr15	51987555	51989368	1813	single	Variation_39168	chr15	51987210	51990457	3247	Wheeler et al. (2008)	Sequencing	
4829	chr15	52737678	52741500	3822	1	chr15	52737647	52741567	3920	HiConf								
4830	chr15	54070520	54082434	11914	1	chr15	54057021	54082942	25921	single								
4831	chr15	54459881	54545435	85554	5	chr15	54459850	54547168	87318	HiConf	Variation_32038	chr15	54506299	54547227	40928	Perry et al. (2008)	Agilent Custom CGH Arrays	
4832	chr15	54547316	54680871	133555	2	chr15	54547315	54683584	136269	HiConf	Variation_9265	chr15	54465022	54683596	218574	Pinto et al. (2007)	Affymetrix 500K SNP Mapping Array	
4833	chr15	56702170	56725298	23128	3	chr15	56702139	56725757	23618	single								y
100789	chr15	58399604	58468927	69323	27	chr15	58399573	58470329	70756	HiConf								
4835	chr15	60247663	60256189	8526	5	chr15	60247632	60256354	8722	HiConf	Variation_38331	chr15	60248389	60254287	5898	McCarroll et al. (2008)	Affymetrix Human SNP Array 6.0	
4836	chr15	60493496	60495309	1813	18	chr15	60493465	60495327	1862	HiConf	Variation_43395	chr15	60493342	60495245	1903	Wang et al. (2008)	Illumina DNA sequencing	
4837	chr15	60742906	60743739	833	8	chr15	60742875	60743757	882	HiConf								
100790	chr15	61535726	61593546	57820	2	chr15	61535695	61594740	59045	single								
4838	chr15	62781208	62793017	11809	13	chr15	62781177	62793231	12054	HiConf								
4839	chr15	62799632	62803160	3528	2	chr15	62799601	62803227	3626	single								
4840	chr15	62803503	62803665	162	17	chr15	62803472	62803668	196	single								y
4841	chr15	62921544	62922524	980	8	chr15	62921513	62922542	1029	single								
4842	chr15	63375823	63378616	2793	23	chr15	63375792	63378683	2891	HiConf								
100791						chr15	63454829	63499860	45031	single								y
4843	chr15	63604016	63606074	2058	50	chr15	63603985	63606092	2107	HiConf								
4844	chr15	63885031	63889804	4773	6	chr15	63885000	63890586	5586	HiConf								
4845						chr15	64709131	64713835	4704	single								
4846	chr15	65655891	65656098	207	15	chr15	65655860	65656105	245	single								
100792	chr15	68926519	69015601	89082	4	chr15	68926488	69017407	90919	HiConf								
4848	chr15	69493816	69498144	4328	16	chr15	69493785	69498195	4410	single	Variation_39173	chr15	69495663	69500744	5081	Wheeler et al. (2008)	Sequencing	
100793	chr15	70144600	70211152	66552	3	chr15	70127576	70212836	85260	single								
4849	chr15	70355334	70355441	107	42	chr15	70355303	70355450	147	HiConf	Variation_45364	chr15	70355289	70355480	191	Bentley et al. (2008)	Illumina DNA sequencing	
4850	chr15	70359303	70362537	3234	25	chr15	70359272	70362604	3332	HiConf								
4851	chr15	70725921	70749343	23422	7	chr15	70725890	70749802	23912	HiConf	Variation_32042	chr15	70726254	70749416	23162	Perry et al. (2008)	Agilent Custom CGH Arrays	
4852	chr15	70800303	70810887	10584	7	chr15	70800272	70811101	10829	HiConf	Variation_3967	chr15	70606839	70907613	300774	Redon et al. (2006)	BAC Array CGH	
100794						chr15	70848096	70899718	51622	single								

locus_id	chrom	start	end	length	Yoruba w/ event	putative chr	putative start	putative end	putative len	putative type	variation_id	DGV_chr	DGV_start	DGV_end	DGV_len	Reference	Method/platform	complex
4853	chr15	71270899	71271007	108	24	chr15	71270868	71271015	147	single	Variation_45365	chr15	71270740	71271019	279	Bentley et al. (2008)	Illumina DNA sequencing	
4854	chr15	71428385	71430051	1666	1	chr15	71428354	71430069	1715	single								
4855	chr15	71711654	71711816	162	14	chr15	71711623	71711819	196	single								
4856	chr15	72138068	72169224	31156	14	chr15	72138021	72169871	31850	HiConf	Variation_32043	chr15	72142415	72169771	27356	Perry et al. (2008)	Agilent Custom CGH Arrays	
4857	chr15	73330592	73379663	49071	14	chr15	73330534	73380661	50127	HiConf	Variation_35341	chr15	73333181	73374031	40850	Kidd et al. (2008)	Paired End Mapping	
100797	chr15	73726510	73771897	45387	1	chr15	73726479	73839693	113214	HiConf								
4858	chr15	74117358	74133258	15900	23	chr15	74117327	74133448	16121	HiConf	Variation_35342	chr15	74119158	74140173	21015	Kidd et al. (2008)	Paired End Mapping	
4859	chr15	74671744	74682034	10290	15	chr15	74671713	74682248	10535	HiConf	Variation_30713	chr15	74671654	74680518	8864	Perry et al. (2008)	Agilent Custom CGH Arrays	
100798	chr15	74981003	74990045	9042	1	chr15	74934255	75009642	75387	single	Variation_3969	chr15	74957605	75160503	202898	Redon et al. (2006)	BAC Array CGH	y
4860	chr15	75115292	75134500	19208	13	chr15	75115261	75134910	19649	HiConf	Variation_35348	chr15	75113906	75129464	15558	Kidd et al. (2008)	Paired End Mapping	
4861	chr15	75376364	75376903	539	9	chr15	75376333	75376921	588	single								
100799						chr15	76574898	76651191	76293	single								
100800						chr15	76795275	76875341	80066	single								
4862	chr15	77014189	77019641	5452	2	chr15	77014158	77019989	5831	single	Variation_7073	chr15	76840539	77080936	240397	de Smith et al. (2007)	Agilent 185k CGH Arrays/Agilent Custom CGH Arrays	
4863	chr15	77335482	77342783	7301	3	chr15	77335451	77342948	7497	HiConf	Variation_38332	chr15	77335797	77340887	5090	McCarroll et al. (2008)	Affymetrix Human SNP Array 6.0	
4864	chr15	77773885	77775257	1372	3	chr15	77773854	77775275	1421	single	Variation_47973	chr15	77743844	77775197	31353	Gusev et al. (2009)	SNP genotyping analysis	
4865	chr15	77954058	77957906	3848	2	chr15	77954027	77961475	7448	single								
4866	chr15	77992278	77995022	2744	12	chr15	77992247	77995089	2842	single								
4867	chr15	80184146	80188213	4067	7	chr15	80184115	80188280	4165	HiConf	Variation_5326	chr15	80080119	80213455	133336	Simon-Sanchez et al. (2007)	Illumina HumanHap300 BeadChip	
4868	chr15	80440416	80449187	8771	7	chr15	80440385	80449352	8967	single	Variation_32051	chr15	80440972	80451464	10492	Perry et al. (2008)	Agilent Custom CGH Arrays	
4869	chr15	80504753	80584133	79380	16	chr15	80504722	80585768	81046	HiConf	Variation_0788	chr15	80499440	80610425	110985	Sharp et al. (2005)	BAC Array CGH	
4870	chr15	80730986	80797969	66983	19	chr15	80730955	80799310	68355	HiConf	Variation_30716	chr15	80509891	80849830	339939	Perry et al. (2008)	Agilent Custom CGH Arrays	
4871	chr15	80827418	80836434	9016	11	chr15	80827387	80836599	9212	single	Variation_32052	chr15	80827184	80836451	9267	Perry et al. (2008)	Agilent Custom CGH Arrays	y
4872	chr15	80908415	80972801	64386	23	chr15	80908384	80974093	65709	HiConf	Variation_30717	chr15	80896416	80982275	85859	Perry et al. (2008)	Agilent Custom CGH Arrays	
4873	chr15	81862641	81863001	360	12	chr15	81862610	81863002	392	HiConf								
4874	chr15	81877782	81879448	1666	20	chr15	81877751	81879466	1715	HiConf								
4875	chr15	82332502	82334756	2254	46	chr15	82332471	82334774	2303	HiConf	Variation_38089	chr15	82331742	82334554	2812	McCarroll et al. (2008)	Affymetrix Human SNP Array 6.0	
4876	chr15	82396251	82398551	2300	28	chr15	82396220	82398572	2352	HiConf								
4877	chr15	82611753	82624444	12691	44	chr15	82611722	82624707	12985	HiConf	Variation_30718	chr15	82610331	82860467	250136	Perry et al. (2008)	Agilent Custom CGH Arrays	
4878	chr15	82638058	82683195	45137	19	chr15	82637790	82684095	46305	HiConf	Variation_4907	chr15	82630622	82775276	144654	Wong et al. (2007)	BAC Array CGH	
4879	chr15	82713526	82731068	17542	17	chr15	82713495	82731429	17934	single	Variation_4907	chr15	82630622	82775276	144654	Wong et al. (2007)	BAC Array CGH	
4880	chr15	82748904	82850040	101136	4	chr15	82748873	82852116	103243	HiConf	Variation_0791	chr15	82753026	82861713	108687	Sharp et al. (2005)	BAC Array CGH	
4881	chr15	83532935	83597535	64600	13	chr15	83532677	83598827	66150	HiConf	Variation_30719	chr15	83530747	83581731	50984	Perry et al. (2008)	Agilent Custom CGH Arrays	
4882	chr15	83685049	83696319	11270	49	chr15	83685018	83696533	11515	HiConf	Variation_33609	chr15	83687238	83687705	467	Perry et al. (2008)	Agilent Custom CGH Arrays	
4883						chr15	83717211	83784978	67767	HiConf								
4884	chr15	83858117	83860028	1911	58	chr15	83858086	83860046	1960	HiConf	Variation_43437	chr15	83857975	83860318	2343	Wang et al. (2008)	Illumina DNA sequencing	
4885	chr15	84140357	84150598	10241	6	chr15	84140326	84150812	10486	HiConf	Variation_10490	chr15	84142286	84150824	8538	Wang et al. (2007)	Illumina HumanHap550 BeadChip	
4886	chr15	84265307	84276528	11221	8	chr15	84265276	84276742	11466	HiConf								
4887	chr15	84974631	84989527	14896	15	chr15	84974600	84989839	15239	HiConf								
4888	chr15	85326059	85328553	2494	2	chr15	85326028	85328968	2940	single								
4889	chr15	85472422	85490697	18275	2	chr15	85472391	85493657	21266	HiConf	Variation_35350	chr15	85473257	85478416	5159	Kidd et al. (2008)	Paired End Mapping	
4890	chr15	85706691	85711542	4851	5	chr15	85706660	85711658	4998	HiConf	Variation_3093	chr15	85706849	85711119	4270	Redon et al. (2006)	Affymetrix 500K EA SNP Mapping Array	
4891	chr15	86511271	86512692	1421	8	chr15	86511240	86512710	1470	single								
4892	chr15	87234805	87236324	1519	5	chr15	87234774	87236342	1568	single								
4893	chr15	87266067	87267684	1617	13	chr15	87266036	87267702	1666	single								
4894	chr15	87350445	87351835	1390	10	chr15	87350414	87352178	1764	HiConf								
4895	chr15	87992625	87996167	3542	3	chr15	87988247	87996332	8085	single								
100803	chr15	88424764	88587594	162830	2	chr15	88424764	88591217	166453	HiConf	Variation_8803	chr15	88423508	88593510	170002	Pinto et al. (2007)	Affymetrix 500K SNP Mapping Array	
4897	chr15	89020476	89023403	2927	5	chr15	89020089	89023470	3381	HiConf								
4898	chr15	89864096	89865370	1274	2	chr15	89864065	89865388	1323	single								
4900	chr15	90389768	90407310	17542	3	chr15	90389737	90407671	17934	HiConf	Variation_3095	chr15	90382555	90416250	33695	Redon et al. (2006)	Affymetrix 500K EA SNP Mapping Array	
4901	chr15	90476008	90477576	1568	17	chr15	90475977	90477594	1617	HiConf								
4902	chr15	90797350	90798820	1470	2	chr15	90797319	90798838	1519	single								
4903	chr15	91507801	91515690	7889	8	chr15	91507770	91515855	8085	single								
4904	chr15	91604674	91623408	18734	2	chr15	91604643	91623900	19257	single	Variation_38878	chr15	91605927	91621304	15377	McCarroll et al. (2008)	Affymetrix Human SNP Array 6.0	
4905	chr15	91658427	91666953	8526	3	chr15	91658396	91667118	8722	HiConf	Variation_38338	chr15	91656315	91667141	10826	McCarroll et al. (2008)	Affymetrix Human SNP Array 6.0	
4906	chr15	92687485	92689485	2000	13	chr15	92687445	92689503	2058	HiConf								
4907	chr15	93493134	93493296	162	49	chr15	93493103	93493299	196	HiConf								
4908	chr15	93791250	93795758	4508	2	chr15	93791219	93795825	4606	HiConf								
4909	chr15	93844562	93847019	2457	10	chr15	93844531	93847716	3185	HiConf	Variation_7083	chr15	93845138	93846566	1428	de Smith et al. (2007)	Agilent 185k CGH Arrays/Agilent Custom CGH Arrays	
4910	chr15	94382876	94383980	1104	12	chr15	94382845	94384021	1176	HiConf	Variation_43917	chr15	94382679	94384011	1332	Bentley et al. (2008)	Illumina DNA sequencing	
4911	chr15	94689357	94690547	1190	5	chr15	94688801	94690565	1764	single								y
4912	chr15	96019917	96036550	16633	27	chr15	96019886	96036693	16807	HiConf								
100806	chr15	96138742	96167750	29008	8	chr15	96138711	96168356	29645	single								
4914	chr15	96333713	96339005	5292	34	chr15	96333682	96339121	5439	HiConf								
4915	chr15	96598362	96601939	3577	2	chr15	96598331	96602006	3675	single								
4916	chr15	96659612	96661131	1519	37	chr15	96659581	96661149	1568	HiConf	Variation_43918	chr15	96659847	96661329	1482	Bentley et al. (2008)	Illumina DNA sequencing	
4917	chr15	97392336	97392946	610	62	chr15	97392327	97392964	637	HiConf	Variation_23504	chr15	97392035	97393161	1126	Levy et al. (2007)	Sequencing	
4918	chr15	98234276	98239274	4998	3	chr15	98234245	98239390	5145	single	Variation_30721	chr15	98234327	98239350	5023	Perry et al. (2008)	Agilent Custom CGH Arrays	
4919	chr15	98276514	98298857	22343	48	chr15	98276483	98299121	22638	HiConf	Variation_25590	chr15	98284484	98285414	930	Levy et al. (2007)	Sequencing	
4920	chr15	98368914	98415888	46974	6	chr15	98368848	98416868	48020	single	Variation_9754	chr15	98177859	98414814	236955	Wang et al. (2007)	Illumina HumanHap550 BeadChip	y
4921	chr15	98610694	98611968	1274	28	chr15	98610663	98611986	1323	single	Variation_29649	chr15	98563203	98622819	59616	Jakobsson et al. (2008)	Illumina HumanHap550 BeadChip	
4922	chr15	98869218	98869771	553	42	chr15	98869187	98869775	588	HiConf	Variation_43135	chr15	98869194	98869970	776	Wang et al. (2008)	Illumina DNA sequencing	
4923	chr15	98912044	98936544	24500	46	chr15	98912013	98937052	25039	HiConf	Variation_35358	chr15	98887659	98922862	35203	Kidd et al. (2008)	Paired End Mapping	y
100808	chr15	99063871	99097291	33420	41	chr15	99063840	99109165	45325	single								y
4924	chr15	99159053	99159837	784	34	chr15	99159022	99159855	833	HiConf	Variation_11818	chr15	99159014	99159896	882	Mills et al. (2006)	Sequence trace read mapping	
4925	chr15	100092111	100128077	35966	20	chr15	100092080	100128781	36701	HiConf	Variation_30725	chr15	100091062	100125853	34791	Perry et al. (2008)	Agilent Custom CGH Arrays	
100809	chr15	100223387	100333345	109958	28	chr15	100213576	100335855	122279	HiConf	Variation_30727	chr15	100262624	100336759	74135	Perry et al. (2008)	Agilent Custom CGH Arrays	
4927						chr16	466	22565	22099	HiConf								
4928	chr16	156856	164658	7802	1	chr16	156825	165155	8330	single	Variation_37503	chr16	161924	166830	4906	Cooper et al. (2008)	Illumina Human 1M BeadChip	y
4929	chr16	167783	187958	20175	15	chr16	167752	188381	20629	HiConf	Variation_35361	chr16	159147	185217	26070	Kidd et al. (2008)	Paired End Mapping	
4930	chr16	257747	258147	400	8	chr16	257716	258157	441	single	Variation_43110	chr16	257438	258174	736	Wang et al. (2008)	Illumina DNA sequencing	
4931	chr16	321741	325563	3822	5	chr16	321710	325630	3920	HiConf	Variation_30731	chr16	322248	324905	2657	Perry et al. (2008)	Agilent Custom CGH Arrays	
4932	chr16	604079	605206	1127	45	chr16	604048	605224	1176	HiConf	Variation_29985	chr16	595687	686885	91198	Jakobsson et al. (2008)	Illumina HumanHap550 BeadChip	
4933	chr16	809242	811714	2472	2	chr16	809211	815728	6517	single	Variation_3987	chr16	266993	1111069	844076	Redon et al. (2006)	BAC Array CGH	
4934	chr16	836584	837172	588	49	chr16	836553	837190	637	HiConf	Variation_3987	chr16	266993	1111069	844076	Redon et al. (2006)	BAC Array CGH	

locus_id	chrom	start	end	length	Yoruba w/ event	putative chr	putative start	putative end	putative len	putative type	variation_id	DGV_chr	DGV_start	DGV_end	DGV_len	Reference	Method/platform	complex
4935	chr16	1266160	1267196	1036	1	chr16	1259913	1267361	7448	single	Variation_38879	chr16	1260595	1289926	29331	McCarroll et al. (2008)	Affymetrix Human SNP Array 6.0	
4936	chr16	2120580	2134545	13965	11	chr16	2120549	2134808	14259	HiConf	Variation_32073	chr16	2122769	2125012	2243	Perry et al. (2008)	Agilent Custom CGH Arrays	
100810						chr16	2430229	2757966	327737	HiConf								
4938	chr16	3143112	3144386	1274	33	chr16	3143081	3144404	1323	single	Variation_4921	chr16	3142783	3249414	106631	Wong et al. (2007)	BAC Array CGH	
4939	chr16	3341611	3341921	310	7	chr16	3341580	3341923	343	single								
4940	chr16	3360525	3364935	4410	7	chr16	3360494	3365002	4508	HiConf								
100811	chr16	5065684	5153484	87800	8	chr16	5065645	5155266	89621	HiConf	Variation_9755	chr16	4996296	5299354	303058	Wang et al. (2007)	Illumina HumanHap550 BeadChip	
4942	chr16	5168491	5183900	15409	4	chr16	5156295	5196867	40572	single	Variation_9755	chr16	4996296	5299354	303058	Wang et al. (2007)	Illumina HumanHap550 BeadChip	
4943	chr16	5234677	5283722	49045	1	chr16	5234646	5284724	50078	single	Variation_9755	chr16	4996296	5299354	303058	Wang et al. (2007)	Illumina HumanHap550 BeadChip	
4944	chr16	5366585	5369574	2989	30	chr16	5366554	5369641	3087	HiConf	Variation_32080	chr16	5367907	5369326	1419	Perry et al. (2008)	Agilent Custom CGH Arrays	
4945	chr16	6243440	6255200	11760	2	chr16	6243409	6255708	12299	single	Variation_30003	chr16	6079141	6653778	574637	Jakobsson et al. (2008)	Illumina HumanHap550 BeadChip	
4946	chr16	6624072	6631226	7154	1	chr16	6624041	6631342	7301	single	Variation_8812	chr16	6598310	6632981	34671	Pinto et al. (2007)	Affymetrix 500K SNP Mapping Array	
100812	chr16	6719976	6743601	23625	3	chr16	6712070	6755851	43781	HiConf	Variation_9270	chr16	6713809	6742380	28571	Pinto et al. (2007)	Affymetrix 500K SNP Mapping Array	
4948	chr16	6942033	6946039	4006	9	chr16	6942002	6946069	4067	HiConf	Variation_34611	chr16	6865782	7021094	155312	Zogopoulos et al. (2007)	Affymetrix 500K and 100K SNP Mapping Arrays	
4949	chr16	8061928	8064721	2793	7	chr16	8061897	8064788	2891	HiConf	Variation_35414	chr16	8052809	8078101	25292	Kidd et al. (2008)	Paired End Mapping	
4950	chr16	8308980	8310456	1476	3	chr16	8308661	8310474	1813	HiConf								
4951	chr16	8663653	8664187	534	48	chr16	8663274	8664205	931	HiConf								
4952						chr16	8849866	8855991	6125	single								
4953	chr16	9681966	9693579	11613	7	chr16	9681935	9693793	11858	single								
4954	chr16	10640161	10640798	637	11	chr16	10640130	10640816	686	single								
4955	chr16	12204535	12204795	260	12	chr16	12204504	12204798	294	single								
4956	chr16	12599138	12618193	19055	11	chr16	12599101	12618554	19453	HiConf	Variation_32084	chr16	12599300	12614734	15434	Perry et al. (2008)	Agilent Custom CGH Arrays	
4957	chr16	13016073	13025334	9261	8	chr16	13016042	13025499	9457	single								
4958	chr16	13200950	13203890	2940	45	chr16	13200919	13203957	3038	HiConf	Variation_43920	chr16	13201971	13203994	2023	Bentley et al. (2008)	Illumina DNA sequencing	
4959	chr16	14351568	14356419	4851	14	chr16	14351537	14356535	4998	single	Variation_35360	chr16	14339141	14379988	40847	Kidd et al. (2008)	Paired End Mapping	
4960	chr16	14407673	14412377	4704	2	chr16	14407642	14412444	4802	single	Variation_43921	chr16	14407233	14412760	5527	Bentley et al. (2008)	Illumina DNA sequencing	
4961	chr16	14687757	14759201	71444	10	chr16	14687726	14760638	72912	HiConf	Variation_4923	chr16	14631546	14759652	128106	Wong et al. (2007)	BAC Array CGH	
4962	chr16	14771106	14816741	45635	2	chr16	14771075	14817527	46452	single	Variation_3992	chr16	14529556	15274051	744495	Redon et al. (2006)	BAC Array CGH	y
4963	chr16	14896007	15029526	133519	20	chr16	14895976	15030138	134162	HiConf	Variation_37843	chr16	14897364	15016088	118724	McCarroll et al. (2008)	Affymetrix Human SNP Array 6.0	
100814	chr16	15146860	15351285	204425	3	chr16	15146807	15355572	208765	HiConf	Variation_4924	chr16	15202458	15407666	205208	Wong et al. (2007)	BAC Array CGH	
4966	chr16	15884092	15930503	46411	6	chr16	15884061	15931493	47432	HiConf	Variation_37692	chr16	15888009	15931537	43528	McCarroll et al. (2008)	Affymetrix Human SNP Array 6.0	
4967	chr16	15979136	15985277	6141	7	chr16	15978974	15985393	6419	HiConf	Variation_38341	chr16	15979352	15985377	6025	McCarroll et al. (2008)	Affymetrix Human SNP Array 6.0	
4968	chr16	16296623	16317350	20727	7	chr16	16296592	16317760	21168	HiConf	Variation_32090	chr16	16295041	16314647	19606	Perry et al. (2008)	Agilent Custom CGH Arrays	
4969	chr16	16634086	16638055	3969	5	chr16	16634055	16638122	4067	HiConf	Variation_30749	chr16	16634437	16639854	5417	Perry et al. (2008)	Agilent Custom CGH Arrays	
4970	chr16	16716847	16745772	28925	1	chr16	16716816	16763660	46844	single	Variation_4929	chr16	16627631	16816757	189126	Wong et al. (2007)	BAC Array CGH	
4971	chr16	17098067	17113461	15394	1	chr16	17098036	17113765	15729	single	Variation_8814	chr16	16824638	18688438	1863800	Pinto et al. (2007)	Affymetrix 500K SNP Mapping Array	
4972	chr16	17571897	17571960	63	26	chr16	17571866	17571964	98	single	Variation_9756	chr16	17516975	18072544	555569	Wang et al. (2007)	Illumina HumanHap550 BeadChip	
4973	chr16	18079537	18117757	38220	3	chr16	18079506	18118510	39004	HiConf	Variation_8815	chr16	17499663	18171589	671926	Pinto et al. (2007)	Affymetrix 500K SNP Mapping Array	
4974	chr16	18397714	18438854	41140	8	chr16	18393008	18439803	46795	HiConf	Variation_32092	chr16	18397779	18427110	29331	Perry et al. (2008)	Agilent Custom CGH Arrays	
100819	chr16	18536322	18677642	141320	2	chr16	18509530	18700728	191198	single	Variation_7091	chr16	18577196	18687445	110249	de Smith et al. (2007)	Agilent 185k CGH Arrays/Agilent Custom CGH Arrays	
4977	chr16	18904501	18918154	13653	6	chr16	18904470	18918337	13867	single	Variation_9757	chr16	18856267	18968449	112182	Wang et al. (2007)	Illumina HumanHap550 BeadChip	
4978	chr16	19161702	19169829	8127	1	chr16	19161671	19170001	8330	single								
100820	chr16	19837976	19872864	34888	14	chr16	19837945	19873519	35574	HiConf	Variation_37504	chr16	19853145	19872019	18874	Cooper et al. (2008)	Illumina Human 1M BeadChip	
4980	chr16	19895869	19900132	4263	8	chr16	19895838	19900199	4361	HiConf	Variation_43924	chr16	19895981	19900041	4060	Bentley et al. (2008)	Illumina DNA sequencing	
100821	chr16	20409232	20451592	42360	13	chr16	20392257	20462891	70634	HiConf	Variation_37505	chr16	20409060	20452031	42971	Cooper et al. (2008)	Illumina Human 1M BeadChip	
100822	chr16	21395376	21462986	67610	4	chr16	21267985	21466950	198965	HiConf	Variation_32100	chr16	21394708	21501101	106393	Perry et al. (2008)	Agilent Custom CGH Arrays	
100823						chr16	21470331	21853633	383302	HiConf								
4985	chr16	22351994	22395586	43592	6	chr16	22351963	22396455	44492	HiConf	Variation_32103	chr16	22356849	22381925	25076	Perry et al. (2008)	Agilent Custom CGH Arrays	
4986						chr16	22399395	22462213	62818	HiConf								
4987	chr16	22604442	22615614	11172	2	chr16	22604411	22615828	11417	single	Variation_29663	chr16	22581355	22651533	70178	Jakobsson et al. (2008)	Illumina HumanHap550 BeadChip	
100825	chr16	22719249	22751491	32242	11	chr16	22719218	22752146	32928	HiConf								
4989	chr16	22897168	22904714	7546	7	chr16	22897137	22904879	7742	HiConf								
4990	chr16	22955357	22956948	1591	43	chr16	22955349	22956966	1617	HiConf	Variation_43925	chr16	22955280	22957031	1751	Bentley et al. (2008)	Illumina DNA sequencing	
4991	chr16	24051020	24055479	4459	2	chr16	24050989	24055546	4557	single	Variation_4935	chr16	23996676	24177670	180994	Wong et al. (2007)	BAC Array CGH	
4992	chr16	24113495	24121433	7938	5	chr16	24113464	24121598	8134	HiConf	Variation_4935	chr16	23996676	24177670	180994	Wong et al. (2007)	BAC Array CGH	
4993	chr16	24448410	24455809	7399	29	chr16	24448379	24455974	7595	HiConf								
100827	chr16	24970701	25043154	72453	1	chr16	24970670	25044636	73966	HiConf	Variation_43696	chr16	24973555	25045087	71532	Wang et al. (2008)	Illumina DNA sequencing	
4994	chr16	25135978	25145631	9653	3	chr16	25135947	25145845	9898	single								
4995	chr16	25247698	25250344	2646	38	chr16	25247667	25250411	2744	HiConf	Variation_43926	chr16	25247615	25250656	3041	Bentley et al. (2008)	Illumina DNA sequencing	
4996	chr16	26787131	26790071	2940	3	chr16	26787100	26790138	3038	HiConf								
4997	chr16	26824420	26825988	1568	57	chr16	26824389	26826006	1617	HiConf	Variation_5817	chr16	26824422	26826998	2576	Mills et al. (2006)	Sequence trace read mapping	y
4998	chr16	27053936	27061814	7878	33	chr16	27053905	27061892	7987	HiConf								
4999	chr16	27787172	27789818	2646	13	chr16	27787141	27789885	2744	HiConf	Variation_43927	chr16	27787939	27789904	1965	Bentley et al. (2008)	Illumina DNA sequencing	
100829	chr16	27811663	27882747	71084	2	chr16	27811274	27884210	72936	single								
5000	chr16	28252525	28364142	111617	15	chr16	28252494	28366370	113876	HiConf	Variation_32106	chr16	28283717	28366478	82761	Perry et al. (2008)	Agilent Custom CGH Arrays	
5001	chr16	28516733	28533785	17052	5	chr16	28516702	28534146	17444	HiConf	Variation_23183	chr16	28520221	28530344	10123	Korbel et al. (2007)	Paired End Mapping	
5002	chr16	28559050	28726159	167109	8	chr16	28558989	28729558	170569	HiConf	Variation_35381	chr16	28569859	28715781	145922	Kidd et al. (2008)	Paired End Mapping	y
5003	chr16	29039563	29041376	1813	24	chr16	29039532	29041394	1862	single	Variation_0971	chr16	29039165	29041643	2478	Hinds et al. (2005)	Oligo arrays	
5004	chr16	29156300	29160299	3999	20	chr16	29155711	29160366	4655	single								
5005	chr16	29160544	29164252	3708	15	chr16	29160513	29164286	3773	single								
100831	chr16	29491272	29547817	56545	1	chr16	29239477	29554057	314580	HiConf	Variation_7102	chr16	29500313	29559994	59681	de Smith et al. (2007)	Agilent 185k CGH Arrays/Agilent Custom CGH Arrays	
5007	chr16	29759079	29763783	4704	10	chr16	29759048	29763850	4802	single	Variation_35383	chr16	29357274	30120928	763654	Kidd et al. (2008)	Paired End Mapping	
5008	chr16	29898778	29899709	931	14	chr16	29898747	29899727	980	single	Variation_35383	chr16	29357274	30120928	763654	Kidd et al. (2008)	Paired End Mapping	
100832	chr16	30102251	30274465	172214	9	chr16	30102220	30289424	187204	HiConf	Variation_4001	chr16	29981456	30290781	309325	Redon et al. (2006)	BAC Array CGH	y
5011	chr16	32034639	32048250	13611	12	chr16	32034608	32048426	13818	single	Variation_39180	chr16	32038634	32040281	1647	Wheeler et al. (2008)	Sequencing	
5012	chr16	32126710	32144613	17903	21	chr16	32126679	32144956	18277	single	Variation_4945	chr16	32095829	32201223	105394	Wong et al. (2007)	BAC Array CGH	
5013	chr16	32145203	32216233	71030	21	chr16	32145201	32217672	72471	HiConf	Variation_35385	chr16	32198944	32220115	21171	Kidd et al. (2008)	Paired End Mapping	
5014	chr16	32219510	32362124	142614	8	chr16	32218946	32364917	145971	HiConf	Variation_32118	chr16	32151724	32364027	212303	Perry et al. (2008)	Agilent Custom CGH Arrays	
5015	chr16	32387586	32388125	539	11	chr16	32387555	32388143	588	single	Variation_2213	chr16	32301596	32438326	136730	Locke et al. (2006)	BAC Array CGH	
5016	chr16	32392241	32509106	116865	16	chr16	32392210	32511476	119266	HiConf	Variation_22839	chr16	32410543	32421217	10674	Korbel et al. (2007)	Paired End Mapping	
5017	chr16	32550315	32691386	141071	23	chr16	32550284	32694246	143962	HiConf	Variation_4946	chr16	32541844	32719609	177765	Wong et al. (2007)	BAC Array CGH	
5018	chr16	32734604	32754400	19796	3	chr16	32734573	32754810	20237	single	Variation_2212	chr16	32646445	32828220	181775	Locke et al. (2006)	BAC Array CGH	
5019	chr16	32771697	32780961	9264	23	chr16	32771666	32781319	9653	single	Variation_32120	chr16	32766052	32931075	165023	Perry et al. (2008)	Agilent Custom CGH Arrays	
5020	chr16	32948979	32955496	6517	4	chr16	32948948	32955612	6664	single	Variation_0324	chr16	32082890	33533065	1450175	Sebat et al. (2004)	ROMA	
5021	chr16	33081279	33222497	141218	17	chr16	33081248	33225357	144109	HiConf	Variation_35388	chr16	33146315	33228951	82636	Kidd et al. (2008)	Paired End Mapping	
5022	chr16	33271693	33279386	7693	12	chr16	33271662	33279551	7889	single	Variation_32121	chr16	33090654	33405980	315326	Perry et al. (2008)	Agilent Custom CGH Arrays	

locus_id	chrom	start	end	length	Yoruba w/ event	putative chr	putative start	putative end	putative len	putative type	variation_id	DGV_chr	DGV_start	DGV_end	DGV_len	Reference	Method/platform	complex
5023	chr16	33288255	33352837	64582	9	chr16	33288224	33354129	65905	HiConf	Variation_23084	chr16	33334452	33340871	6419	Korbel et al. (2007)	Paired End Mapping	
5024	chr16	33392527	33398505	5978	28	chr16	33392496	33398572	6076	HiConf	Variation_45423	chr16	33393432	33393771	339	Bentley et al. (2008)	Illumina DNA sequencing	
5025	chr16	33447407	33457769	10362	6	chr16	33447376	33457960	10584	single	Variation_32122	chr16	33443968	33479777	35809	Perry et al. (2008)	Agilent Custom CGH Arrays	
5026	chr16	33458401	33459384	983	6	chr16	33458401	33461243	2842	single	Variation_32122	chr16	33443968	33479777	35809	Perry et al. (2008)	Agilent Custom CGH Arrays	
5027	chr16	33475631	33477885	2254	11	chr16	33475600	33477903	2303	single	Variation_32122	chr16	33443968	33479777	35809	Perry et al. (2008)	Agilent Custom CGH Arrays	
5028	chr16	33725384	33761309	35925	2	chr16	33725010	33762103	37093	HiConf	Variation_37633	chr16	33463813	33867424	403611	Cooper et al. (2008)	Illumina Human 1M BeadChip	
5029	chr16	33842934	34034113	191179	15	chr16	33829233	34036846	207613	HiConf	Variation_30782	chr16	33852389	33874106	21717	Perry et al. (2008)	Agilent Custom CGH Arrays	
5030	chr16	34275997	34280260	4263	20	chr16	34275966	34280327	4361	single	Variation_0485	chr16	34248297	34573297	325000	Tuzun et al. (2005)	Paired End Mapping	
100834	chr16	34646437	34650864	4427	1	chr16	34573445	34664071	90626	single	Variation_4953	chr16	34515401	34713569	198168	Wong et al. (2007)	BAC Array CGH	
5031	chr16	34831020	34842290	11270	5	chr16	34830989	34842504	11515	HiConf	Variation_30783	chr16	34734046	34882794	148748	Perry et al. (2008)	Agilent Custom CGH Arrays	
5032	chr16	44943333	45018333	75000	36	chr16	44940000	45019902	79902	HiConf	Variation_37634	chr16	44943958	45037126	93168	Cooper et al. (2008)	Illumina Human 1M BeadChip	
5033	chr16	45020472	45052273	31801	4	chr16	45020441	45052928	32487	HiConf	Variation_22780	chr16	45039596	45047612	8016	Korbel et al. (2007)	Paired End Mapping	
5034	chr16	46371313	46377625	6312	8	chr16	46371273	46377741	6468	HiConf								
5035	chr16	46445784	46446234	450	31	chr16	46445753	46446243	490	single								y
5036	chr16	47067202	47069165	1963	24	chr16	47067171	47069180	2009	HiConf								
5037	chr16	47462926	47463236	310	31	chr16	47462895	47463238	343	single	Variation_7112	chr16	47462888	47464131	1243	de Smith et al. (2007)	Agilent 185k CGH Arrays/Agilent Custom CGH Arrays	
5038	chr16	47523539	47523746	207	22	chr16	47523508	47523753	245	single								
5039	chr16	47845910	47846449	539	34	chr16	47845879	47846467	588	HiConf								
5040	chr16	49733488	49740124	6636	10	chr16	49733457	49740268	6811	single								
5041	chr16	50213296	50213458	162	53	chr16	50213265	50213461	196	HiConf								
5042	chr16	50447124	50451485	4361	3	chr16	50447093	50451552	4459	single	Variation_47872	chr16	50436066	50874031	437965	Gusev et al. (2009)	SNP genotyping analysis	
5043						chr16	52747545	52751416	3871	single								
5044	chr16	54615554	54617269	1715	12	chr16	54615523	54617287	1764	single								
5045	chr16	54918213	54920453	2240	1	chr16	54910062	54924076	14014	HiConf	Variation_4957	chr16	54850768	55038253	187485	Wong et al. (2007)	BAC Array CGH	
5046	chr16	55172831	55174693	1862	19	chr16	55172800	55174711	1911	single	Variation_5828	chr16	55172686	55174527	1841	Mills et al. (2006)	Sequence trace read mapping	y
5047	chr16	55884262	55884622	360	26	chr16	55884231	55884623	392	HiConf								
5048	chr16	56062573	56062833	260	5	chr16	56062542	56062836	294	single								
5049	chr16	56606630	56608418	1788	6	chr16	56605854	56613351	7497	single								
5050	chr16	57204786	57207115	2329	67	chr16	57204781	57207133	2352	HiConf	Variation_30785	chr16	57204913	57206938	2025	Perry et al. (2008)	Agilent Custom CGH Arrays	
5051	chr16	57232218	57233722	1504	20	chr16	57231927	57233740	1813	single	Variation_33634	chr16	57232599	57233543	944	Perry et al. (2008)	Agilent Custom CGH Arrays	
5052	chr16	57480682	57486072	5390	29	chr16	57480651	57486188	5537	HiConf								
5053	chr16	57486415	57487297	882	24	chr16	57486384	57487315	931	single								
5054	chr16	57502732	57505672	2940	19	chr16	57502701	57512550	9849	HiConf	Variation_43651	chr16	57503245	57511892	8647	Wang et al. (2008)	Illumina DNA sequencing	
5055	chr16	58026486	58028838	2352	20	chr16	58025825	58029353	3528	HiConf								
5056	chr16	58544717	58551430	6713	5	chr16	58544686	58551546	6860	HiConf								
5057	chr16	59024378	59024638	260	23	chr16	59024347	59024641	294	single								
5058	chr16	61099479	61108054	8575	21	chr16	61099448	61108219	8771	HiConf	Variation_35391	chr16	61086020	61109247	23227	Kidd et al. (2008)	Paired End Mapping	
100835	chr16	62629235	62670346	41111	4	chr16	62629204	62671172	41968	HiConf	Variation_38345	chr16	62636133	62671716	35583	McCarroll et al. (2008)	Affymetrix Human SNP Array 6.0	y
5060	chr16	63146895	63148316	1421	46	chr16	63146864	63148334	1470	HiConf								
5061	chr16	63207802	63209419	1617	1	chr16	63207771	63209437	1666	single								
5062	chr16	63843969	63844904	935	4	chr16	63843938	63844918	980	single	Variation_47974	chr16	63694595	63936997	242402	Gusev et al. (2009)	SNP genotyping analysis	
5063	chr16	64037372	64044722	7350	12	chr16	64037341	64044887	7546	HiConf	Variation_4961	chr16	63973728	64146306	172578	Wong et al. (2007)	BAC Array CGH	
5064	chr16	65292507	65296800	4293	18	chr16	65292476	65297572	5096	HiConf								
100836	chr16	65765129	65770097	4968	1	chr16	65739038	65787523	48485	single								
5065	chr16	66003644	66006059	2415	1	chr16	66003613	66009395	5782	single								
5066	chr16	67345117	67348065	2948	10	chr16	67345086	67348467	3381	single								
5067	chr16	67397988	67426849	28861	1	chr16	67397957	67427455	29498	single	Variation_9761	chr16	67414790	67420815	6025	Wang et al. (2007)	Illumina HumanHap550 BeadChip	y
5068	chr16	68534076	68600241	66165	15	chr16	68533532	68601397	67865	HiConf	Variation_32128	chr16	68534434	68563823	29389	Perry et al. (2008)	Agilent Custom CGH Arrays	y
5069	chr16	68602114	68605740	3626	12	chr16	68602083	68605807	3724	HiConf	Variation_32129	chr16	68602755	68666385	63630	Perry et al. (2008)	Agilent Custom CGH Arrays	
5070	chr16	68702717	68839148	136431	38	chr16	68701700	68841007	139307	HiConf	Variation_32131	chr16	68702955	68789873	86918	Perry et al. (2008)	Agilent Custom CGH Arrays	
5071	chr16	69258126	69265182	7056	2	chr16	69258095	69265298	7203	HiConf								
5072	chr16	69535534	69537083	1549	20	chr16	69535533	69537101	1568	HiConf	Variation_43930	chr16	69535299	69537390	2091	Bentley et al. (2008)	Illumina DNA sequencing	y
100838	chr16	70627799	70682454	54655	44	chr16	70627768	70683603	55835	HiConf	Variation_32132	chr16	70643022	70667434	24412	Perry et al. (2008)	Agilent Custom CGH Arrays	
5074	chr16	70876547	70878164	1617	7	chr16	70876516	70878182	1666	HiConf								
100839	chr16	71190049	71233703	43654	3	chr16	71190018	71234731	44713	single	Variation_8828	chr16	71190800	71209415	18615	Pinto et al. (2007)	Affymetrix 500K SNP Mapping Array	
5076	chr16	72172107	72174214	2107	4	chr16	72172076	72174232	2156	single								
5077	chr16	72257024	72258151	1127	8	chr16	72256993	72258169	1176	HiConf								
5078	chr16	72459198	72468655	9457	7	chr16	72459167	72468820	9653	single								y
100840	chr16	72910586	73026948	116362	35	chr16	72910555	73028351	117796	HiConf	Variation_32133	chr16	72919818	73021701	101883	Perry et al. (2008)	Agilent Custom CGH Arrays	
5080	chr16	73408916	73412341	3425	3	chr16	73408885	73415745	6860	single								
5081	chr16	73430770	73431456	686	5	chr16	73430739	73431474	735	single								
5082	chr16	74019603	74020191	588	4	chr16	74019572	74020209	637	single	Variation_4013	chr16	73977115	74134472	157357	Redon et al. (2006)	BAC Array CGH	
5083	chr16	74089477	74132989	43512	4	chr16	74089446	74139377	49931	single	Variation_30791	chr16	74095995	74132305	36310	Perry et al. (2008)	Agilent Custom CGH Arrays	
100841						chr16	74092509	74133865	41356	single								
5084	chr16	75091870	75101557	9687	22	chr16	75091839	75102717	10878	HiConf	Variation_43580	chr16	75096554	75101642	5088	Wang et al. (2008)	Illumina DNA sequencing	
5085	chr16	75156354	75158951	2597	15	chr16	75156323	75159018	2695	HiConf	Variation_43933	chr16	75156418	75159063	2645	Bentley et al. (2008)	Illumina DNA sequencing	
5086	chr16	75219221	75228384	9163	14	chr16	75219190	75228549	9359	HiConf	Variation_37936	chr16	75224186	75226991	2805	McCarroll et al. (2008)	Affymetrix Human SNP Array 6.0	
5087	chr16	75648657	75650274	1617	17	chr16	75648626	75650292	1666	HiConf	Variation_5438	chr16	75101609	75683063	581454	Simon-Sanchez et al. (2007)	Illumina HumanHap300 BeadChip	
5088	chr16	76617191	76632827	15636	2	chr16	76617160	76633085	15925	HiConf	Variation_9765	chr16	76619660	76629008	9348	Wang et al. (2007)	Illumina HumanHap550 BeadChip	
5089	chr16	76851166	76853321	2155	12	chr16	76851135	76853340	2205	single	Variation_4014	chr16	76811430	76929139	117709	Redon et al. (2006)	BAC Array CGH	
5090	chr16	76857242	76919766	62524	4	chr16	76857211	76921058	63847	HiConf	Variation_4014	chr16	76811430	76929139	117709	Redon et al. (2006)	BAC Array CGH	
5091	chr16	76923784	76943402	19618	46	chr16	76923753	76943647	19894	HiConf	Variation_22861	chr16	76928828	76942620	13792	Korbel et al. (2007)	Paired End Mapping	
5092	chr16	77189119	77198282	9163	6	chr16	77189088	77198447	9359	single								
5093	chr16	77616399	77620417	4018	12	chr16	77616368	77620484	4116	HiConf	Variation_37886	chr16	77617649	77620658	3009	McCarroll et al. (2008)	Affymetrix Human SNP Array 6.0	
5094	chr16	78148795	78149094	299	5	chr16	78148753	78149096	343	single								
100843	chr16	78407431	78439531	32100	3	chr16	78407400	78440156	32756	single	Variation_35413	chr16	78404074	78432787	28713	Kidd et al. (2008)	Paired End Mapping	
5096	chr16	79461151	79470867	9716	3	chr16	79461120	79471067	9947	HiConf	Variation_38348	chr16	79460358	79471170	10812	McCarroll et al. (2008)	Affymetrix Human SNP Array 6.0	
5097	chr16	79532201	79540580	8379	11	chr16	79532170	79540745	8575	HiConf	Variation_32137	chr16	79536443	79540299	3856	Perry et al. (2008)	Agilent Custom CGH Arrays	
5098	chr16	80289398	80298610	9212	2	chr16	80289367	80298775	9408	single								
5099	chr16	80312477	80313849	1372	5	chr16	80312446	80313867	1421	single								y
5100	chr16	80395581	80398570	2989	7	chr16	80395550	80398637	3087	single	Variation_37696	chr16	80395345	80398185	2840	McCarroll et al. (2008)	Affymetrix Human SNP Array 6.0	
5101	chr16	81329472	81330011	539	4	chr16	81329441	81330029	588	single	Variation_8831	chr16	81272197	81409797	137600	Pinto et al. (2007)	Affymetrix 500K SNP Mapping Array	
5102						chr16	82509459	82510978	1519	single								
5103	chr16	82537469	82542369	4900	9	chr16	82537438	82542485	5047	single	Variation_45473	chr16	82541946	82542165	219	Bentley et al. (2008)	Illumina DNA sequencing	
5104	chr16	82635371	82641398	6027	5	chr16	82635340	82641514	6174	single								
5105	chr16	83058780	83061328	2548	54	chr16	83058749	83061395	2646	HiConf	Variation_7135	chr16	83056817	83061318	4501	de Smith et al. (2007)	Agilent 185k CGH Arrays/Agilent Custom CGH Arrays	

locus_id	chrom	start	end	length	Yoruba w/ event	putative chr	putative start	putative end	putative len	putative type	variation_id	DGV_chr	DGV_start	DGV_end	DGV_len	Reference	Method/platform	complex
5106	chr16	84236936	84240415	3479	29	chr16	84236905	84240482	3577	HiConf								
5107	chr16	85473353	85477714	4361	3	chr16	85473322	85477781	4459	HiConf	Variation_4015	chr16	85399263	85564336	165073	Redon et al. (2006)	BAC Array CGH	
5108	chr16	85569466	85592238	22772	22	chr16	85569411	85592686	23275	HiConf								
5109	chr16	86133922	86137891	3969	4	chr16	86133891	86137958	4067	single	Variation_4971	chr16	86025064	86192948	167884	Wong et al. (2007)	BAC Array CGH	
100845	chr16	86292021	86307727	15706	8	chr16	86207514	86307988	100474	HiConf								y
5110	chr16	86385684	86388820	3136	30	chr16	86385653	86388887	3234	HiConf								
5111	chr16	86591337	86592431	1094	41	chr16	86591306	86592433	1127	single	Variation_25717	chr16	86591392	86592190	798	Levy et al. (2007)	Sequencing	
5112	chr16	86836141	86846921	10780	6	chr16	86836110	86847135	11025	HiConf								
5113	chr16	86867893	86872891	4998	35	chr16	86867862	86873007	5145	HiConf								
5114	chr16	86926742	86927820	1078	37	chr16	86926711	86927838	1127	HiConf	Variation_30010	chr16	86889141	87022477	133336	Jakobsson et al. (2008)	Illumina HumanHap550 BeadChip	
5115	chr16	87004064	87004603	539	18	chr16	87004033	87004621	588	single	Variation_30009	chr16	86976186	87082408	106222	Jakobsson et al. (2008)	Illumina HumanHap550 BeadChip	
5116	chr16	87523464	87524346	882	52	chr16	87523433	87524364	931	HiConf	Variation_30016	chr16	87404930	87529899	124969	Jakobsson et al. (2008)	Illumina HumanHap550 BeadChip	
5117	chr16	87555363	87556108	745	42	chr16	87555332	87556116	784	HiConf	Variation_25730	chr16	87555812	87555960	148	Levy et al. (2007)	Sequencing	
5118	chr16	88659774	88668192	8418	6	chr16	88659743	88668906	9163	single	Variation_43938	chr16	88658478	88664654	6176	Bentley et al. (2008)	Illumina DNA sequencing	y
5119	chr16	88683686	88817554	133868	14	chr16	88683655	88820267	136612	HiConf	Variation_30799	chr16	88690880	88719132	28252	Perry et al. (2008)	Agilent Custom CGH Arrays	
5120	chr17	146272	167391	21119	15	chr17	146241	167801	21560	HiConf	Variation_35435	chr17	161772	168534	6762	Kidd et al. (2008)	Paired End Mapping	
5121	chr17	358638	360402	1764	40	chr17	358607	360420	1813	single	Variation_4020	chr17	343377	440727	97350	Redon et al. (2006)	BAC Array CGH	y
5122	chr17	495495	496736	1241	46	chr17	495464	496738	1274	HiConf	Variation_25878	chr17	495779	496459	680	Levy et al. (2007)	Sequencing	
5123	chr17	620249	627158	6909	14	chr17	620218	627274	7056	single	Variation_3136	chr17	595817	897708	301891	Redon et al. (2006)	Affymetrix 500K EA SNP Mapping Array	
5124	chr17	665280	668465	3185	12	chr17	665249	668532	3283	single	Variation_9767	chr17	642766	797292	154526	Wang et al. (2007)	Illumina HumanHap550 BeadChip	y
5125	chr17	790818	802040	11222	48	chr17	790787	802155	11368	HiConf	Variation_23538	chr17	791635	792946	1311	Levy et al. (2007)	Sequencing	
5126	chr17	1009309	1009759	450	76	chr17	1009278	1009768	490	HiConf	Variation_45496	chr17	1009591	1009807	216	Bentley et al. (2008)	Illumina DNA sequencing	
5127	chr17	1013621	1014079	458	46	chr17	1013590	1014080	490	HiConf	Variation_25768	chr17	1014012	1014319	307	Levy et al. (2007)	Sequencing	
5128	chr17	1477798	1480918	3120	8	chr17	1477767	1483794	6027	single								
5129	chr17	2855581	2857050	1469	9	chr17	2855549	2857068	1519	single								
5130	chr17	3204852	3208331	3479	7	chr17	3204821	3208398	3577	HiConf	Variation_22843	chr17	3203952	3214925	10973	Korbel et al. (2007)	Paired End Mapping	
5131	chr17	3516786	3526341	9555	9	chr17	3516755	3526506	9751	single	Variation_4982	chr17	3476232	3656125	179893	Wong et al. (2007)	BAC Array CGH	
100847	chr17	3674173	3739070	64897	8	chr17	3674119	3811907	137788	single	Variation_45544	chr17	3718019	3718309	290	Bentley et al. (2008)	Illumina DNA sequencing	
5132	chr17	4508007	4509820	1813	8	chr17	4507976	4509838	1862	single	Variation_4985	chr17	4496300	4710353	214053	Wong et al. (2007)	BAC Array CGH	
5133	chr17	4702341	4705820	3479	23	chr17	4702310	4705887	3577	single	Variation_4987	chr17	4703112	4835308	132196	Wong et al. (2007)	BAC Array CGH	
5134	chr17	5535733	5538134	2401	43	chr17	5535702	5538201	2499	HiConf								
5135	chr17	5594582	5595954	1372	1	chr17	5594551	5595972	1421	single								
5136	chr17	6058416	6074472	16056	4	chr17	6058385	6074702	16317	HiConf	Variation_38361	chr17	6047214	6073498	26284	McCarroll et al. (2008)	Affymetrix Human SNP Array 6.0	y
5137	chr17	6145195	6146175	980	12	chr17	6145164	6146193	1029	single								
5138	chr17	6365842	6371379	5537	4	chr17	6365811	6371495	5684	single								
5139	chr17	6811840	6817753	5913	5	chr17	6811809	6817787	5978	HiConf	Variation_42751	chr17	6815298	6815833	535	Wang et al. (2008)	Illumina DNA sequencing	
5140	chr17	7018865	7020041	1176	27	chr17	7018834	7020059	1225	single								
5141	chr17	10702914	10707144	4230	5	chr17	10702507	10707211	4704	HiConf								
5142	chr17	10831114	10836357	5243	15	chr17	10831083	10836473	5390	HiConf	Variation_5852	chr17	10827584	10836460	8876	Mills et al. (2006)	Sequence trace read mapping	
5143	chr17	10969049	10977232	8183	12	chr17	10969018	10977397	8379	HiConf	Variation_1287	chr17	10970310	10973815	3505	Conrad et al. (2005)	Mendelian inconsistencies	
5144	chr17	11985358	11986975	1617	8	chr17	11985327	11986993	1666	single								
5145	chr17	12080320	12082282	1962	1	chr17	12080289	12082298	2009	single								
5146	chr17	13472900	13473350	450	76	chr17	13472869	13473359	490	HiConf	Variation_0798	chr17	13415317	13594294	178977	Sharp et al. (2005)	BAC Array CGH	
5147	chr17	14131558	14132195	637	54	chr17	14131527	14132213	686	HiConf	Variation_23517	chr17	14130636	14132281	1645	Levy et al. (2007)	Sequencing	
5148	chr17	15124739	15126160	1421	1	chr17	15124708	15126178	1470	single								
5149	chr17	15530949	15535849	4900	12	chr17	15530918	15535965	5047	single								
5150	chr17	15588328	15600418	12090	5	chr17	15588297	15600547	12250	single	Variation_37697	chr17	15591355	15614730	23375	McCarroll et al. (2008)	Affymetrix Human SNP Array 6.0	
5151	chr17	15615971	15629047	13076	6	chr17	15615443	15629310	13867	HiConf	Variation_38349	chr17	15617726	15635510	17784	McCarroll et al. (2008)	Affymetrix Human SNP Array 6.0	
5152	chr17	15730428	15734299	3871	45	chr17	15730397	15734366	3969	HiConf	Variation_23029	chr17	15729200	15734745	5545	Korbel et al. (2007)	Paired End Mapping	
5153	chr17	16007425	16014294	6869	1	chr17	16007394	16014303	6909	HiConf								
5154	chr17	16654617	16665152	10535	23	chr17	16654586	16665366	10780	HiConf	Variation_32153	chr17	16657486	16665353	7867	Perry et al. (2008)	Agilent Custom CGH Arrays	
5155	chr17	16666151	16685924	19773	8	chr17	16666101	16686387	20286	HiConf	Variation_30804	chr17	16658768	16717750	58982	Perry et al. (2008)	Agilent Custom CGH Arrays	
5156	chr17	16733458	16738162	4704	2	chr17	16733427	16738229	4802	single	Variation_4023	chr17	16467781	16758162	290381	Redon et al. (2006)	BAC Array CGH	
5157	chr17	17468066	17468997	931	3	chr17	17468035	17469015	980	single								
100849	chr17	17563017	17576115	13098	1	chr17	17530241	17639413	109172	single	Variation_30806	chr17	17564707	17569002	4295	Perry et al. (2008)	Agilent Custom CGH Arrays	
5158	chr17	17804952	17808714	3762	6	chr17	17803832	17808830	4998	single								
5159						chr17	17973029	17976851	3822	HiConf								
100850	chr17	18279353	18521993	242640	4	chr17	18209087	18528371	319284	HiConf	Variation_38841	chr17	18296117	18405946	109829	McCarroll et al. (2008)	Affymetrix Human SNP Array 6.0	
5162	chr17	18534649	18538961	4312	13	chr17	18534618	18539028	4410	single	Variation_4993	chr17	18503340	18688049	184709	Wong et al. (2007)	BAC Array CGH	
5163	chr17	18696937	18697247	310	5	chr17	18696906	18697249	343	single	Variation_30808	chr17	18234217	19081776	847559	Perry et al. (2008)	Agilent Custom CGH Arrays	
100851	chr17	18859973	19113927	253954	12	chr17	18810390	19120119	309729	HiConf	Variation_32155	chr17	18873538	19078459	204921	Perry et al. (2008)	Agilent Custom CGH Arrays	
100852	chr17	19424951	19507590	82639	12	chr17	19424777	19508077	83300	HiConf	Variation_3140	chr17	19426327	19504358	78031	Redon et al. (2006)	Affymetrix 500K EA SNP Mapping Array	
5167	chr17	19956384	19962362	5978	4	chr17	19956353	19962478	6125	HiConf								
100853	chr17	20162692	20380014	217322	12	chr17	20162619	20384442	221823	HiConf	Variation_4995	chr17	20185621	20373865	188244	Wong et al. (2007)	BAC Array CGH	
5169	chr17	20553792	20555066	1274	25	chr17	20553761	20555084	1323	single	Variation_43943	chr17	20551585	20555188	3603	Bentley et al. (2008)	Illumina DNA sequencing	
5170	chr17	20574274	20577214	2940	16	chr17	20574243	20577281	3038	single	Variation_30812	chr17	20571582	20577452	5870	Perry et al. (2008)	Agilent Custom CGH Arrays	
5171	chr17	20763953	20766305	2352	17	chr17	20763922	20766323	2401	HiConf	Variation_8836	chr17	20403500	20854748	451248	Pinto et al. (2007)	Affymetrix 500K SNP Mapping Array	
100854	chr17	21446401	21607621	161220	10	chr17	21446370	21609834	163464	HiConf	Variation_39198	chr17	21497312	21498663	1351	Wheeler et al. (2008)	Sequencing	y
5174	chr17	21827351	21831467	4116	18	chr17	21827320	21831534	4214	HiConf	Variation_35446	chr17	21828753	21830118	1365	Kidd et al. (2008)	Paired End Mapping	
5175	chr17	21947355	21947612	257	44	chr17	21947321	21947615	294	single	Variation_32165	chr17	21941754	21955191	13437	Perry et al. (2008)	Agilent Custom CGH Arrays	
5176	chr17	22560783	22562106	1323	55	chr17	22560752	22562124	1372	HiConf	Variation_43947	chr17	22560683	22564508	3825	Bentley et al. (2008)	Illumina DNA sequencing	y
5177	chr17	22644916	22652805	7889	26	chr17	22644885	22652970	8085	single								
5178	chr17	22770650	22771010	360	9	chr17	22770619	22771011	392	single	Variation_22804	chr17	22766823	22775461	8638	Korbel et al. (2007)	Paired End Mapping	
5179	chr17	25203549	25205754	2205	3	chr17	25203518	25205772	2254	single	Variation_38353	chr17	25204962	25206809	1847	McCarroll et al. (2008)	Affymetrix Human SNP Array 6.0	
100855	chr17	25731169	25759479	28310	2	chr17	25717406	25760330	42924	HiConf								
5180	chr17	26039626	26048456	8830	8	chr17	26039262	26048621	9359	single	Variation_38354	chr17	26040482	26043732	3250	McCarroll et al. (2008)	Affymetrix Human SNP Array 6.0	
5181	chr17	26127199	26129698	2499	12	chr17	26127168	26129765	2597	HiConf								
5182	chr17	26854751	26855011	260	27	chr17	26854720	26855014	294	single								
5183	chr17	27013560	27015079	1519	10	chr17	27013529	27015097	1568	HiConf								
5184	chr17	27333922	27335931	2009	11	chr17	27333891	27335949	2058	single	Variation_4030	chr17	27245834	27562095	316261	Redon et al. (2006)	BAC Array CGH	
100856						chr17	28597601	28621097	23496	single								
100857						chr17	28689011	28755259	66248	single								
5186	chr17	28924217	28927451	3234	1	chr17	28924186	28927518	3332	single								
5187	chr17	29088514	29088577	63	23	chr17	29088483	29088581	98	single								
5188	chr17	29897014	29898680	1666	11	chr17	29896983	29898698	1715	HiConf	Variation_43370	chr17	29897059	29898829	1770	Wang et al. (2008)	Illumina DNA sequencing	
100858	chr17	30187421	30189748	2327	4	chr17	30134878	30226655	91777	single	Variation_34366	chr17	30189627	30189766	139	Levy et al. (2007)	Sequencing	

locus_id	chrom	start	end	length	Yoruba w/ event	putative chr	putative start	putative end	putative len	putative type	variation_id	DGV_chr	DGV_start	DGV_end	DGV_len	Reference	Method/platform	complex
5189	chr17	30378439	30388386	9947	6	chr17	30378408	30388600	10192	HiConf								
5190	chr17	31055080	31055480	400	3	chr17	31055049	31055490	441	single								y
5191	chr17	31431890	31882117	450227	49	chr17	31431859	31889274	457415	HiConf	Variation_30824	chr17	31429658	31888786	459128	Perry et al. (2008)	Agilent Custom CGH Arrays	
5192	chr17	32066244	32069331	3087	2	chr17	32066213	32069398	3185	single	Variation_3143	chr17	32018706	32104672	85966	Redon et al. (2006)	Affymetrix 500K EA SNP Mapping Array	
5193	chr17	32212509	32221159	8650	1	chr17	32212478	32221200	8722	single	Variation_3144	chr17	32210529	32219667	9138	Redon et al. (2006)	Affymetrix 500K EA SNP Mapping Array	
5194	chr17	32830203	32832604	2401	14	chr17	32830172	32832622	2450	HiConf	Variation_32174	chr17	32830175	32832670	2495	Perry et al. (2008)	Agilent Custom CGH Arrays	y
5195	chr17	33288108	33657274	369166	21	chr17	33288077	33664789	376712	HiConf	Variation_30827	chr17	33324930	33658997	334067	Perry et al. (2008)	Agilent Custom CGH Arrays	
100861	chr17	33825271	33930487	105216	3	chr17	33825240	34154079	328839	HiConf	Variation_2224	chr17	33803800	33955396	151596	Locke et al. (2006)	BAC Array CGH	
100862	chr17	34996758	35103237	106479	4	chr17	34995654	35105757	110103	single								y
5199	chr17	36457330	36463651	6321	8	chr17	36457299	36463767	6468	single	Variation_38355	chr17	36457235	36463665	6430	McCarroll et al. (2008)	Affymetrix Human SNP Array 6.0	
5200	chr17	36509074	36541904	32830	9	chr17	36509043	36542559	33516	HiConf	Variation_38356	chr17	36510770	36528661	17891	McCarroll et al. (2008)	Affymetrix Human SNP Array 6.0	
5201	chr17	36637033	36659410	22377	31	chr17	36637031	36659963	22932	HiConf	Variation_35452	chr17	36636267	36653666	17399	Kidd et al. (2008)	Paired End Mapping	
5202	chr17	36674939	36685273	10334	46	chr17	36674908	36685492	10584	HiConf	Variation_7159	chr17	36675647	36685241	9594	de Smith et al. (2007)	Agilent 185k CGH Arrays/Agilent Custom CGH Arrays	
100864	chr17	36759121	36782298	23177	32	chr17	36759090	36782782	23692	HiConf	Variation_35456	chr17	36758317	36785944	27627	Kidd et al. (2008)	Paired End Mapping	
5204	chr17	36785924	36792637	6713	15	chr17	36785893	36792753	6860	HiConf	Variation_37796	chr17	36786395	36790200	3805	McCarroll et al. (2008)	Affymetrix Human SNP Array 6.0	
5205	chr17	36991773	36995448	3675	15	chr17	36991742	36995515	3773	HiConf	Variation_32180	chr17	36992630	36995333	2703	Perry et al. (2008)	Agilent Custom CGH Arrays	
5206	chr17	37013823	37025191	11368	2	chr17	37013792	37025405	11613	HiConf	Variation_30833	chr17	36999462	37014738	15276	Perry et al. (2008)	Agilent Custom CGH Arrays	
5207						chr17	38737612	38737661	49	single								
5208	chr17	38781230	38797424	16194	44	chr17	38781222	38797784	16562	HiConf	Variation_35461	chr17	38777604	38804687	27083	Kidd et al. (2008)	Paired End Mapping	
5209	chr17	38873079	38873142	63	25	chr17	38873048	38873146	98	single	Variation_5007	chr17	38864523	39045455	180932	Wong et al. (2007)	BAC Array CGH	
5210	chr17	39354504	39361364	6860	2	chr17	39354473	39361480	7007	single								
5211						chr17	39634998	39646023	11025	HiConf								
5212	chr17	39752678	39758288	5610	4	chr17	39752647	39758380	5733	single								
5213	chr17	39987437	40012142	24705	3	chr17	39987406	40012445	25039	single								
5214	chr17	40196569	40201420	4851	3	chr17	40196538	40201536	4998	single	Variation_5009	chr17	40072682	40258767	186085	Wong et al. (2007)	BAC Array CGH	
5215						chr17	40667477	40684235	16758	HiConf								
100866	chr17	40845343	41054243	208900	10	chr17	40845298	41058203	212905	HiConf	Variation_34654	chr17	40848884	41022689	173805	Zogopoulos et al. (2007)	Affymetrix 500K and 100K SNP Mapping Arrays	y
5217	chr17	41656622	41664952	8330	14	chr17	41656591	41665117	8526	single	Variation_5012	chr17	41590929	41719579	128650	Wong et al. (2007)	BAC Array CGH	
5218	chr17	41727359	41778190	50831	14	chr17	41727004	41779238	52234	HiConf	Variation_35469	chr17	41714303	41922542	208239	Kidd et al. (2008)	Paired End Mapping	
5219	chr17	41780445	41807640	27195	41	chr17	41780414	41808197	27783	HiConf	Variation_32184	chr17	41770281	41975625	205344	Perry et al. (2008)	Agilent Custom CGH Arrays	
5220	chr17	41942893	42006166	63273	19	chr17	41942800	42007921	65121	HiConf	Variation_37589	chr17	41922658	42005250	82592	Cooper et al. (2008)	Illumina Human 1M BeadChip	
5221	chr17	42008123	42063835	55712	35	chr17	42008117	42065055	56938	single	Variation_37635	chr17	41936430	42092850	156420	Cooper et al. (2008)	Illumina Human 1M BeadChip	
5222	chr17	42066507	42099043	32536	46	chr17	42066476	42099698	33222	single	Variation_32185	chr17	41985210	42141946	156736	Perry et al. (2008)	Agilent Custom CGH Arrays	
5223	chr17	42100268	42100628	360	50	chr17	42100237	42100629	392	single	Variation_32185	chr17	41985210	42141946	156736	Perry et al. (2008)	Agilent Custom CGH Arrays	
5224	chr17	42100831	42137263	36432	43	chr17	42100825	42138016	37191	single	Variation_32185	chr17	41985210	42141946	156736	Perry et al. (2008)	Agilent Custom CGH Arrays	
5225	chr17	42441749	42444248	2499	3	chr17	42441718	42444315	2597	HiConf	Variation_5015	chr17	42418934	42607623	188689	Wong et al. (2007)	BAC Array CGH	
5226	chr17	42446159	42494110	47951	10	chr17	42446128	42495079	48951	HiConf	Variation_30838	chr17	42446148	42487442	41294	Perry et al. (2008)	Agilent Custom CGH Arrays	
5227	chr17	42497952	42508487	10535	2	chr17	42497921	42508701	10780	HiConf	Variation_5015	chr17	42418934	42607623	188689	Wong et al. (2007)	BAC Array CGH	
100869	chr17	42746828	42871283	124455	7	chr17	42746449	42873800	127351	HiConf								
100870	chr17	42958638	43032157	73519	31	chr17	42875123	43035647	160524	HiConf	Variation_32186	chr17	42970185	43025568	55383	Perry et al. (2008)	Agilent Custom CGH Arrays	
5230	chr17	43150289	43150649	360	5	chr17	43150258	43150650	392	single								
5231	chr17	43502550	43503628	1078	33	chr17	43502519	43503646	1127	single								
100871	chr17	43968336	43982860	14524	2	chr17	43964883	44078955	114072	single	Variation_39203	chr17	43970802	43972222	1420	Wheeler et al. (2008)	Sequencing	
5232	chr17	44259992	44262137	2145	23	chr17	44259961	44262754	2793	HiConf								
100872	chr17	45529803	45655831	126028	5	chr17	45529772	45658421	128649	HiConf								
100873						chr17	45958424	46051965	93541	HiConf								
5234	chr17	46889234	46893889	4655	3	chr17	46889203	46893956	4753	HiConf								y
5235	chr17	46978806	46981942	3136	43	chr17	46978775	46982009	3234	HiConf	Variation_43951	chr17	46978997	46982056	3059	Bentley et al. (2008)	Illumina DNA sequencing	y
5236	chr17	47253157	47254137	980	18	chr17	47253126	47254155	1029	HiConf								
5237	chr17	47847446	47849781	2335	3	chr17	47846369	47849848	3479	HiConf								
5238	chr17	49513545	49517888	4343	13	chr17	49513545	49517955	4410	HiConf	Variation_23181	chr17	49513414	49523124	9710	Korbel et al. (2007)	Paired End Mapping	
5239	chr17	50816388	50820210	3822	27	chr17	50816357	50820277	3920	HiConf								
5240	chr17	50898855	50904918	6063	9	chr17	50898824	50905292	6468	single								
5241						chr17	50946501	50949980	3479	single								
5242	chr17	51170707	51172774	2067	1	chr17	51170676	51172783	2107	single								
5243	chr17	51515275	51527329	12054	3	chr17	51515244	51527592	12348	HiConf	Variation_10517	chr17	51515464	51527276	11812	Wang et al. (2007)	Illumina HumanHap550 BeadChip	
5244	chr17	51954511	51958480	3969	3	chr17	51954480	51958547	4067	HiConf								y
5245	chr17	52060155	52061086	931	15	chr17	52060124	52061104	980	HiConf								
5246	chr17	52306282	52307556	1274	2	chr17	52306251	52307574	1323	single	Variation_7166	chr17	52115894	52434492	318598	de Smith et al. (2007)	Agilent 185k CGH Arrays/Agilent Custom CGH Arrays	
5247	chr17	52445167	52447500	2333	27	chr17	52445166	52447518	2352	HiConf	Variation_35479	chr17	52443666	52490415	46749	Kidd et al. (2008)	Paired End Mapping	
5248	chr17	53042899	53044761	1862	47	chr17	53042868	53044779	1911	HiConf	Variation_7167	chr17	52445066	53148466	703400	de Smith et al. (2007)	Agilent 185k CGH Arrays/Agilent Custom CGH Arrays	
5249	chr17	54358059	54368790	10731	1	chr17	54358028	54369004	10976	single								
5250	chr17	54604039	54609233	5194	2	chr17	54604008	54609349	5341	single	Variation_4043	chr17	54437361	54780416	343055	Redon et al. (2006)	BAC Array CGH	
5251	chr17	55372163	55375544	3381	16	chr17	55372132	55375611	3479	single	Variation_30842	chr17	55369406	55373396	3990	Perry et al. (2008)	Agilent Custom CGH Arrays	
5252	chr17	55434834	55436500	1666	32	chr17	55434803	55436518	1715	single	Variation_32190	chr17	55435332	55454017	18685	Perry et al. (2008)	Agilent Custom CGH Arrays	y
5253	chr17	55531315	55542928	11613	38	chr17	55531284	55543142	11858	HiConf	Variation_30844	chr17	55514303	55558252	43949	Perry et al. (2008)	Agilent Custom CGH Arrays	
5254	chr17	55764898	55768426	3528	49	chr17	55764867	55768493	3626	HiConf								
5255	chr17	55803951	55813702	9751	21	chr17	55803920	55813916	9996	HiConf	Variation_33653	chr17	55808452	55808996	544	Perry et al. (2008)	Agilent Custom CGH Arrays	
5256	chr17	55934683	55936006	1323	65	chr17	55934652	55936024	1372	HiConf								
5257	chr17	56009065	56009947	882	22	chr17	56009034	56009965	931	HiConf								
5258	chr17	57652917	57668325	15408	21	chr17	57652886	57668958	16072	HiConf	Variation_30845	chr17	57653396	57667259	13863	Perry et al. (2008)	Agilent Custom CGH Arrays	
5259	chr17	57683444	57708679	25235	23	chr17	57683413	57709187	25774	HiConf	Variation_32193	chr17	57683473	57709158	25685	Perry et al. (2008)	Agilent Custom CGH Arrays	
5260	chr17	58535897	58541924	6027	10	chr17	58535866	58542040	6174	HiConf								
5261						chr17	59377049	59386898	9849	single								
5262	chr17	60267606	60343458	75852	11	chr17	60267575	60344995	77420	HiConf	Variation_30847	chr17	60269648	60360559	90911	Perry et al. (2008)	Agilent Custom CGH Arrays	
5263	chr17	61717663	61719280	1617	47	chr17	61717632	61719298	1666	HiConf								
5264	chr17	63998515	64005914	7399	16	chr17	63998484	64006079	7595	HiConf								
5265	chr17	64074857	64076670	1813	14	chr17	64074826	64076688	1862	HiConf								
5266	chr17	64597295	64605777	8482	2	chr17	64597264	64605888	8624	single								
5267	chr17	64853908	64860327	6419	24	chr17	64853877	64860443	6566	HiConf	Variation_43953	chr17	64855962	64860429	4467	Bentley et al. (2008)	Illumina DNA sequencing	
5268	chr17	67389962	67395202	5240	1	chr17	67386001	67399133	13132	single								
5269	chr17	67570027	67600750	30723	2	chr17	67569996	67601356	31360	single	Variation_5028	chr17	67541685	67740709	199024	Wong et al. (2007)	BAC Array CGH	
5270	chr17	68301597	68306595	4998	4	chr17	68301566	68306711	5145	HiConf	Variation_38362	chr17	68304153	68306056	1903	McCarroll et al. (2008)	Affymetrix Human SNP Array 6.0	
5271	chr17	68632200	68663382	31182	14	chr17	68632169	68663529	31360	HiConf								
5272						chr17	69841048	69844429	3381	single								
100875						chr17	70354764	70386320	31556	single								

locus_id	chrom	start	end	length	Yoruba w/ event	putative chr	putative start	putative end	putative len	putative type	variation_id	DGV_chr	DGV_start	DGV_end	DGV_len	Reference	Method/platform	complex
5273	chr17	70445886	70448679	2793	16	chr17	70445855	70448746	2891	single								y
5274	chr17	70619542	70620179	637	38	chr17	70619511	70620197	686	single								
5275	chr17	71527463	71528590	1127	29	chr17	71527432	71528608	1176	single								
5276	chr17	71875039	71876000	961	22	chr17	71875038	71876018	980	single	Variation_39210	chr17	71873832	71876903	3071	Wheeler et al. (2008)	Sequencing	
5277	chr17	72298772	72301516	2744	13	chr17	72298741	72301583	2842	HiConf								
5278	chr17	72744045	72748056	4011	1	chr17	72727050	72749492	22442	HiConf	Variation_25923	chr17	72747559	72747901	342	Levy et al. (2007)	Sequencing	
100876						chr17	72898109	72984447	86338	single								y
5279	chr17	73032498	73037251	4753	7	chr17	73032467	73037318	4851	single								
5280						chr17	73656531	73659765	3234	single								
5281	chr17	73980874	73982298	1424	1	chr17	73975325	73984243	8918	HiConf								
5282	chr17	73985385	73990056	4671	18	chr17	73984390	73990172	5782	single								
5283	chr17	74040575	74051845	11270	23	chr17	74040544	74052059	11515	HiConf								
5284	chr17	74518325	74518487	162	11	chr17	74518294	74518490	196	single	Variation_30028	chr17	74481477	74599400	117923	Jakobsson et al. (2008)	Illumina HumanHap550 BeadChip	
5285	chr17	74573254	74573793	539	23	chr17	74573223	74573811	588	single	Variation_30029	chr17	74544687	74599400	54713	Jakobsson et al. (2008)	Illumina HumanHap550 BeadChip	
5286	chr17	74751516	74755436	3920	7	chr17	74751485	74755503	4018	single	Variation_3153	chr17	74743681	74774728	31047	Redon et al. (2006)	Affymetrix 500K EA SNP Mapping Array	
5287	chr17	74851427	74855445	4018	5	chr17	74851396	74855512	4116	single	Variation_5035	chr17	74849625	75033343	183718	Wong et al. (2007)	BAC Array CGH	
5288	chr17	75294289	75315738	21449	12	chr17	75294258	75316210	21952	HiConf	Variation_30853	chr17	75295256	75303042	7786	Perry et al. (2008)	Agilent Custom CGH Arrays	
5289	chr17	75607399	75614896	7497	15	chr17	75607368	75615061	7693	HiConf								y
5290	chr17	76217008	76218233	1225	17	chr17	76216977	76218251	1274	single								
5291	chr17	76270075	76270183	108	64	chr17	76270044	76270191	147	single								
5292	chr17	76309128	76313440	4312	13	chr17	76309097	76313507	4410	HiConf								y
5293	chr17	76332568	76333351	783	64	chr17	76332568	76333352	784	single	Variation_25941	chr17	76332501	76333203	702	Levy et al. (2007)	Sequencing	
5294	chr17	76347838	76348328	490	10	chr17	76347807	76348346	539	single								
5295	chr17	76941623	76947203	5580	6	chr17	76941344	76947567	6223	HiConf	Variation_4327	chr17	76896216	77057694	161478	Wong et al. (2007)	BAC Array CGH	
5296	chr17	77303039	77303583	544	1	chr17	77301984	77303601	1617	single	Variation_30034	chr17	77202218	77307788	105570	Jakobsson et al. (2008)	Illumina HumanHap550 BeadChip	
5297	chr17	77999775	77999988	213	29	chr17	77999744	77999989	245	single	Variation_34352	chr17	77999897	78000059	162	Levy et al. (2007)	Sequencing	
5298	chr17	78243942	78247372	3430	17	chr17	78243911	78247439	3528	HiConf								
100877	chr18	32591	104634	72043	7	chr18	858	106625	105767	HiConf	Variation_38883	chr18	38133	68539	30406	McCarroll et al. (2008)	Affymetrix Human SNP Array 6.0	
5300	chr18	283080	294056	10976	5	chr18	283049	294270	11221	HiConf	Variation_38366	chr18	283522	294262	10740	McCarroll et al. (2008)	Affymetrix Human SNP Array 6.0	
5301	chr18	496622	498533	1911	7	chr18	496591	498551	1960	single								
5302	chr18	500346	504423	4077	21	chr18	500315	504480	4165	single								
5303	chr18	2364404	2369794	5390	25	chr18	2364373	2369910	5537	HiConf								
5304	chr18	3468815	3472490	3675	3	chr18	3468784	3472557	3773	single								
5305	chr18	3625615	3625925	310	2	chr18	3625584	3625927	343	single								
5306	chr18	3756074	3761002	4928	2	chr18	3755483	3761118	5635	single	Variation_4049	chr18	3667422	4160252	492830	Redon et al. (2006)	BAC Array CGH	
5307	chr18	3962424	3962784	360	1	chr18	3959225	3962851	3626	single	Variation_8854	chr18	3783886	4096164	312278	Pinto et al. (2007)	Affymetrix 500K SNP Mapping Array	
5308	chr18	4319406	4325482	6076	8	chr18	4319375	4325598	6223	HiConf	Variation_10525	chr18	4320954	4325455	4501	Wang et al. (2007)	Illumina HumanHap550 BeadChip	y
5309	chr18	4490269	4524471	34202	23	chr18	4490238	4525175	34937	HiConf								
5310	chr18	4525745	4526676	931	12	chr18	4525714	4526694	980	HiConf								
5311	chr18	4695530	4696853	1323	1	chr18	4695499	4696871	1372	single								
5312	chr18	4803869	4803932	63	34	chr18	4803838	4803936	98	single	Variation_45661	chr18	4803797	4804480	683	Bentley et al. (2008)	Illumina DNA sequencing	
5313	chr18	5200230	5200590	360	55	chr18	5200199	5200591	392	HiConf								
5314	chr18	5914307	5923862	9555	2	chr18	5914276	5924027	9751	HiConf	Variation_38374	chr18	5918109	5923771	5662	McCarroll et al. (2008)	Affymetrix Human SNP Array 6.0	
5315	chr18	5987121	5987229	108	28	chr18	5987090	5987237	147	single								
5316	chr18	6140834	6140888	54	1	chr18	6140803	6140950	147	single	Variation_10526	chr18	6114882	6174092	59210	Wang et al. (2007)	Illumina HumanHap550 BeadChip	
5317	chr18	6144344	6150977	6633	5	chr18	6141097	6151191	10094	single	Variation_10526	chr18	6114882	6174092	59210	Wang et al. (2007)	Illumina HumanHap550 BeadChip	
5318	chr18	6151369	6153672	2303	15	chr18	6151338	6153690	2352	single	Variation_10526	chr18	6114882	6174092	59210	Wang et al. (2007)	Illumina HumanHap550 BeadChip	
5319	chr18	6262550	6263443	893	49	chr18	6262519	6263450	931	HiConf	Variation_26091	chr18	6262554	6262788	234	Levy et al. (2007)	Sequencing	
5320	chr18	6902784	6917876	15092	2	chr18	6902753	6918188	15435	single	Variation_33657	chr18	6903552	6904056	504	Perry et al. (2008)	Agilent Custom CGH Arrays	
5321	chr18	7098735	7104130	5395	7	chr18	7098704	7104241	5537	single								
100879	chr18	9084359	9145767	61408	2	chr18	9084160	9158689	74529	HiConf								
5322	chr18	9418493	9424090	5597	1	chr18	9418462	9428115	9653	single	Variation_5045	chr18	9391631	9587291	195660	Wong et al. (2007)	BAC Array CGH	
5323	chr18	10446366	10451707	5341	5	chr18	10446335	10451823	5488	single	Variation_11131	chr18	10448243	10448572	329	de Smith et al. (2007)	Agilent 185k CGH Arrays/Agilent Custom CGH Arrays	
5324	chr18	10745413	10747814	2401	49	chr18	10745382	10747881	2499	HiConf								
5325	chr18	11500013	11500797	784	52	chr18	11499982	11500815	833	HiConf	Variation_5888	chr18	11499706	11501474	1768	Mills et al. (2006)	Sequence trace read mapping	
5326	chr18	11549503	11555334	5831	2	chr18	11549472	11555450	5978	single								
5327	chr18	11617006	11639467	22461	2	chr18	11600089	11640269	40180	single	Variation_23224	chr18	11601345	11635370	34025	Korbel et al. (2007)	Paired End Mapping	
5328	chr18	11855214	11856831	1617	24	chr18	11855183	11856849	1666	HiConf								
5329	chr18	12892544	12897052	4508	5	chr18	12892513	12897119	4606	single								
5330	chr18	13117699	13123775	6076	3	chr18	13117668	13123891	6223	single	Variation_10529	chr18	13120681	13123577	2896	Wang et al. (2007)	Illumina HumanHap550 BeadChip	
5331	chr18	13813107	13813744	637	42	chr18	13813076	13813762	686	HiConf								
5332	chr18	14190064	14192073	2009	1	chr18	14190033	14192091	2058	single	Variation_4053	chr18	13988748	15367893	1379145	Redon et al. (2006)	BAC Array CGH	
100880	chr18	14322854	14548156	225302	13	chr18	14322823	14552756	229933	HiConf	Variation_3159	chr18	14198563	14548050	349487	Redon et al. (2006)	Affymetrix 500K EA SNP Mapping Array	
5335	chr18	14979209	14986212	7003	3	chr18	14979178	14986675	7497	single	Variation_7179	chr18	14919347	15042868	123521	de Smith et al. (2007)	Agilent 185k CGH Arrays/Agilent Custom CGH Arrays	
5336	chr18	14988911	14996114	7203	16	chr18	14988880	14996279	7399	single	Variation_7179	chr18	14919347	15042868	123521	de Smith et al. (2007)	Agilent 185k CGH Arrays/Agilent Custom CGH Arrays	
100881	chr18	15370678	15399559	28881	18	chr18	15018231	16067689	1049458	HiConf								y
5338	chr18	16944795	16946608	1813	24	chr18	16944764	16946626	1862	single								
5339	chr18	16990267	16993256	2989	20	chr18	16990236	16993323	3087	single								
5340	chr18	18308224	18321107	12883	7	chr18	18308189	18321370	13181	HiConf	Variation_10530	chr18	18308209	18320153	11944	Wang et al. (2007)	Illumina HumanHap550 BeadChip	
5341	chr18	18773965	18776905	2940	6	chr18	18773934	18776972	3038	HiConf								
100882	chr18	20216901	20263429	46528	2	chr18	20206106	20271227	65121	single								
5343	chr18	22001959	22005074	3115	17	chr18	22001956	22005141	3185	HiConf	Variation_23542	chr18	22001811	22005328	3517	Levy et al. (2007)	Sequencing	
5344	chr18	22018176	22019872	1696	11	chr18	22018175	22019890	1715	HiConf	Variation_23543	chr18	22016969	22020126	3157	Levy et al. (2007)	Sequencing	
5345	chr18	22662850	22663879	1029	3	chr18	22662819	22663897	1078	HiConf								
5346	chr18	22825748	22826175	427	28	chr18	22825744	22826185	441	HiConf	Variation_40217	chr18	22825618	22826339	721	Wheeler et al. (2008)	Sequencing	
5347	chr18	23045687	23045795	108	38	chr18	23045656	23045803	147	HiConf	Variation_45633	chr18	23045589	23045867	278	Bentley et al. (2008)	Illumina DNA sequencing	
5348	chr18	24227175	24235358	8183	7	chr18	24227144	24235523	8379	HiConf								
5349	chr18	25317033	25320022	2989	4	chr18	25317002	25320089	3087	HiConf								
5350	chr18	25340602	25342758	2156	1	chr18	25340571	25342776	2205	single								
5351	chr18	25493531	25495197	1666	6	chr18	25493500	25495215	1715	HiConf	Variation_43961	chr18	25492798	25495419	2621	Bentley et al. (2008)	Illumina DNA sequencing	
5352	chr18	26533654	26537525	3871	5	chr18	26533623	26537592	3969	HiConf								
5353	chr18	27025418	27029730	4312	4	chr18	27025387	27029797	4410	HiConf								y
5354	chr18	28142177	28143794	1617	18	chr18	28142146	28143812	1666	HiConf	Variation_43962	chr18	28142163	28143489	1326	Bentley et al. (2008)	Illumina DNA sequencing	
5355	chr18	28480620	28481698	1078	3	chr18	28480589	28481716	1127	single								
5356	chr18	28606452	28606942	490	16	chr18	28606421	28606960	539	single								
5357	chr18	30013438	30014761	1323	3	chr18	30013407	30014779	1372	single								

locus_id	chrom	start	end	length	Yoruba w/ event	putative chr	putative start	putative end	putative len	putative type	variation_id	DGV_chr	DGV_start	DGV_end	DGV_len	Reference	Method/platform	complex
5358	chr18	31014018	31021662	7644	2	chr18	31013987	31021827	7840	HiConf								
5359	chr18	32289451	32291687	2236	3	chr18	32289212	32291858	2646	HiConf								
5360	chr18	32507146	32513663	6517	6	chr18	32507115	32513779	6664	HiConf	Variation_22773	chr18	32506084	32513933	7849	Korbel et al. (2007)	Paired End Mapping	
5361	chr18	32763416	32765817	2401	4	chr18	32763385	32765884	2499	HiConf								
5362	chr18	33003712	33011258	7546	1	chr18	33003681	33011423	7742	single								y
5363	chr18	33560156	33560556	400	73	chr18	33560125	33560566	441	HiConf	Variation_43121	chr18	33560020	33560768	748	Wang et al. (2008)	Illumina DNA sequencing	
5364	chr18	33784233	33785948	1715	10	chr18	33784202	33785966	1764	single								
5365	chr18	33786389	33787253	864	17	chr18	33786358	33787289	931	single								
5366	chr18	33804421	33805891	1470	8	chr18	33804390	33805909	1519	HiConf								
5367	chr18	34217736	34221705	3969	9	chr18	34217705	34221772	4067	HiConf								
100883	chr18	34367456	34424590	57134	6	chr18	34367425	34425735	58310	single								
5368	chr18	34470870	34471270	400	8	chr18	34470839	34471280	441	single								
5369	chr18	34831706	34838811	7105	3	chr18	34831675	34838927	7252	HiConf								
5370	chr18	34910057	34920690	10633	6	chr18	34910026	34920904	10878	HiConf	Variation_38368	chr18	34911440	34920221	8781	McCarroll et al. (2008)	Affymetrix Human SNP Array 6.0	
100884	chr18	35265337	35288932	23595	2	chr18	35262042	35289458	27416	single								
5372	chr18	36260595	36260757	162	69	chr18	36260564	36260760	196	HiConf	Variation_10876	chr18	36260200	36261019	819	Hinds et al. (2005)	Oligo arrays	
5373	chr18	36513729	36520638	6909	27	chr18	36513698	36520754	7056	HiConf	Variation_32212	chr18	36514820	36520512	5692	Perry et al. (2008)	Agilent Custom CGH Arrays	y
5374	chr18	37119173	37122211	3038	41	chr18	37119142	37122278	3136	HiConf	Variation_43963	chr18	37118778	37122343	3565	Bentley et al. (2008)	Illumina DNA sequencing	
5375	chr18	38287627	38295369	7742	5	chr18	38287596	38295534	7938	HiConf								
5376	chr18	38308305	38311490	3185	4	chr18	38308274	38311557	3283	HiConf	Variation_38369	chr18	38310567	38311469	902	McCarroll et al. (2008)	Affymetrix Human SNP Array 6.0	
5377	chr18	38418322	38418632	310	11	chr18	38418181	38418720	539	single								
5378	chr18	38506461	38511606	5145	2	chr18	38506430	38511722	5292	HiConf								
100885	chr18	39135341	39156005	20664	3	chr18	39127848	39156587	28739	HiConf	Variation_38370	chr18	39135060	39157329	22269	McCarroll et al. (2008)	Affymetrix Human SNP Array 6.0	
5380	chr18	40230624	40235867	5243	9	chr18	40230593	40235983	5390	HiConf	Variation_43964	chr18	40230708	40236070	5362	Bentley et al. (2008)	Illumina DNA sequencing	
5381	chr18	40282858	40283818	960	4	chr18	40282827	40283856	1029	single								
5382	chr18	40284083	40284191	108	3	chr18	40284052	40284199	147	single								
5383	chr18	41092266	41092779	513	56	chr18	41092258	41092797	539	HiConf	Variation_11952	chr18	41092106	41092807	701	Mills et al. (2006)	Sequence trace read mapping	
5384	chr18	42205716	42208215	2499	1	chr18	42205685	42208282	2597	single								
5385	chr18	42532105	42532505	400	33	chr18	42532074	42532515	441	HiConf								
5386	chr18	42741874	42746431	4557	5	chr18	42741843	42746498	4655	single	Variation_5054	chr18	42660422	42808151	147729	Wong et al. (2007)	BAC Array CGH	
5387	chr18	42795823	42813022	17199	31	chr18	42795792	42813383	17591	HiConf	Variation_35554	chr18	42805538	42807663	2125	Kidd et al. (2008)	Paired End Mapping	
5388	chr18	43349082	43349964	882	21	chr18	43349051	43349982	931	HiConf								
5389	chr18	44001615	44002105	490	35	chr18	44001584	44002123	539	HiConf								
5390	chr18	44293949	44294586	637	1	chr18	44293918	44294604	686	single								
5391	chr18	45251703	45259102	7399	45	chr18	45251672	45259267	7595	HiConf	Variation_37993	chr18	45251724	45257350	5626	McCarroll et al. (2008)	Affymetrix Human SNP Array 6.0	
5392	chr18	45536148	45542665	6517	24	chr18	45536117	45542781	6664	single								y
5393	chr18	45948608	45953040	4432	48	chr18	45948599	45953107	4508	HiConf	Variation_43537	chr18	45948935	45952472	3537	Wang et al. (2008)	Illumina DNA sequencing	
5394	chr18	46078627	46083037	4410	14	chr18	46078596	46083496	4900	HiConf	Variation_35576	chr18	46075696	46091043	15347	Kidd et al. (2008)	Paired End Mapping	
5395	chr18	46149873	46152519	2646	9	chr18	46149842	46152586	2744	HiConf	Variation_47955	chr18	46138509	46524501	385992	Gusev et al. (2009)	SNP genotyping analysis	
5396	chr18	47442101	47444306	2205	8	chr18	47442070	47444324	2254	single	Variation_43965	chr18	47442024	47444559	2535	Bentley et al. (2008)	Illumina DNA sequencing	
5397	chr18	47459433	47465874	6441	2	chr18	47450498	47475292	24794	HiConf								
5398	chr18	47612278	47613209	931	3	chr18	47612247	47613227	980	single								
5399						chr18	47698732	47698781	49	single								
5400	chr18	48166027	48168575	2548	2	chr18	48165996	48168642	2646	HiConf								
5401	chr18	48610114	48614524	4410	2	chr18	48610083	48614591	4508	HiConf								
5402	chr18	48716666	48716752	86	52	chr18	48716658	48716756	98	HiConf	Variation_11961	chr18	48716564	48717028	464	Mills et al. (2006)	Sequence trace read mapping	
5403	chr18	49390537	49391615	1078	13	chr18	49390506	49391633	1127	HiConf								
5404	chr18	49459986	49466487	6501	6	chr18	49459694	49466603	6909	HiConf	Variation_37910	chr18	49461207	49464634	3427	McCarroll et al. (2008)	Affymetrix Human SNP Array 6.0	
5405	chr18	49690172	49690280	108	48	chr18	49690141	49690288	147	single	Variation_35580	chr18	49677318	49691438	14120	Kidd et al. (2008)	Paired End Mapping	y
100887	chr18	50119701	50196612	76911	21	chr18	50119651	50198173	78522	HiConf								
5407	chr18	50542282	50544977	2695	4	chr18	50542251	50545044	2793	single								
5408	chr18	50653218	50655178	1960	4	chr18	50653187	50655196	2009	HiConf								
5409	chr18	51514785	51516500	1715	17	chr18	51514754	51516518	1764	HiConf								
5411	chr18	52842783	52843287	504	85	chr18	52842752	52843291	539	HiConf	Variation_40234	chr18	52842839	52843184	345	Wheeler et al. (2008)	Sequencing	y
5412	chr18	53097806	53099796	1990	48	chr18	53097797	53099806	2009	HiConf	Variation_43968	chr18	53097736	53099717	1981	Bentley et al. (2008)	Illumina DNA sequencing	
5413	chr18	54368545	54368995	450	26	chr18	54368514	54369004	490	HiConf								
5414	chr18	55788271	55790373	2102	10	chr18	55788240	55790396	2156	single								
5415	chr18	55803167	55815368	12201	3	chr18	55803136	55815631	12495	HiConf	Variation_30871	chr18	55803812	55815432	11620	Perry et al. (2008)	Agilent Custom CGH Arrays	
5416	chr18	56067473	56076440	8967	6	chr18	56067442	56076605	9163	HiConf	Variation_22798	chr18	56067675	56076233	8558	Korbel et al. (2007)	Paired End Mapping	
5417	chr18	56193991	56198646	4655	2	chr18	56193960	56198713	4753	HiConf								
5418	chr18	56268039	56271901	3862	1	chr18	56267999	56271968	3969	single	Variation_3171	chr18	56251349	56278547	27198	Redon et al. (2006)	Affymetrix 500K EA SNP Mapping Array	
100889	chr18	59747006	59874936	127930	2	chr18	59746975	59877535	130560	HiConf								
5419	chr18	60193077	60193337	260	7	chr18	60193046	60193340	294	single	Variation_5337	chr18	60125616	60303316	177700	Simon-Sanchez et al. (2007)	Illumina HumanHap300 BeadChip	
5420	chr18	60582627	60594632	12005	24	chr18	60582596	60594895	12299	HiConf	Variation_3173	chr18	60558805	60600790	41985	Redon et al. (2006)	Affymetrix 500K EA SNP Mapping Array	
5421	chr18	61351388	61357905	6517	18	chr18	61351357	61358021	6664	HiConf	Variation_32215	chr18	61351802	61357912	6110	Perry et al. (2008)	Agilent Custom CGH Arrays	
5422	chr18	61485354	61489274	3920	4	chr18	61485323	61489341	4018	HiConf								
5423	chr18	61786606	61791359	4753	8	chr18	61786575	61791426	4851	HiConf	Variation_38375	chr18	61788030	61791107	3077	McCarroll et al. (2008)	Affymetrix Human SNP Array 6.0	
5424	chr18	61862997	61869212	6215	3	chr18	61862966	61869336	6370	HiConf	Variation_35584	chr18	61858938	61894678	35740	Kidd et al. (2008)	Paired End Mapping	
5425	chr18	61874022	61886621	12599	14	chr18	61873991	61886878	12887	HiConf	Variation_23186	chr18	61874764	61885039	10275	Korbel et al. (2007)	Paired End Mapping	
5426	chr18	61887026	61888232	1206	4	chr18	61887025	61888250	1225	single	Variation_35584	chr18	61858938	61894678	35740	Kidd et al. (2008)	Paired End Mapping	
5427	chr18	61917884	61920793	2909	68	chr18	61917846	61920835	2989	HiConf	Variation_43454	chr18	61917819	61920243	2424	Wang et al. (2008)	Illumina DNA sequencing	
5428	chr18	61980903	61985791	4888	10	chr18	61980664	61985907	5243	HiConf	Variation_43974	chr18	61980917	61985908	4991	Bentley et al. (2008)	Illumina DNA sequencing	
5429	chr18	62281359	62283907	2548	2	chr18	62281328	62283974	2646	single	Variation_4059	chr18	62157007	62341492	184485	Redon et al. (2006)	BAC Array CGH	
5430	chr18	62369706	62371813	2107	58	chr18	62369675	62371831	2156	HiConf								
5431	chr18	63107499	63118034	10535	27	chr18	63107468	63118248	10780	HiConf	Variation_22801	chr18	63109678	63118284	8606	Korbel et al. (2007)	Paired End Mapping	
5432	chr18	63130333	63130949	616	16	chr18	63130302	63131037	735	HiConf								
100890	chr18	63447337	63468100	20763	15	chr18	63443461	63481951	38490	HiConf	Variation_37510	chr18	63447152	63468024	20872	Cooper et al. (2008)	Illumina Human 1M BeadChip	
5434	chr18	63946575	63965538	18963	13	chr18	63946544	63965899	19355	HiConf	Variation_37703	chr18	63949433	63959430	9997	McCarroll et al. (2008)	Affymetrix Human SNP Array 6.0	
5435	chr18	64156834	64211238	54404	2	chr18	64156803	64212271	55468	HiConf	Variation_1296	chr18	64195345	64211988	16643	Conrad et al. (2005)	Mendelian inconsistencies	
5436	chr18	64212672	64214801	2129	3	chr18	64212663	64214819	2156	HiConf	Variation_1984	chr18	64173660	64272543	98883	McCarroll et al. (2005)	Mendelian inconsistencies/Null genotypes	
5437	chr18	64215120	64272327	57207	2	chr18	64215113	64273472	58359	HiConf	Variation_1297	chr18	64224850	64243196	18346	Conrad et al. (2005)	Mendelian inconsistencies	
5438	chr18	64380048	64380240	192	40	chr18	64380047	64380243	196	HiConf	Variation_11973	chr18	64379938	64380315	377	Mills et al. (2006)	Sequence trace read mapping	
100892	chr18	64682653	64725185	42532	1	chr18	64682622	64726061	43439	single								
5439	chr18	65143841	65145017	1176	52	chr18	65143810	65145035	1225	HiConf	Variation_43976	chr18	65143799	65145075	1276	Bentley et al. (2008)	Illumina DNA sequencing	
5440	chr18	65300690	65302460	1770	11	chr18	65300659	65302472	1813	HiConf	Variation_7188	chr18	64953366	67496827	2543461	de Smith et al. (2007)	Agilent 185k CGH Arrays/Agilent Custom CGH Arrays	
5441	chr18	68917674	68920124	2450	6	chr18	68917643	68920191	2548	HiConf	Variation_43977	chr18	68917648	68920239	2591	Bentley et al. (2008)	Illumina DNA sequencing	

locus_id	chrom	start	end	length	Yoruba w/ event	putative chr	putative start	putative end	putative len	putative type	variation_id	DGV_chr	DGV_start	DGV_end	DGV_len	Reference	Method/platform	complex
5442	chr18	70015240	70035999	20759	26	chr18	70015194	70036411	21217	HiConf								y
5443	chr18	72212679	72245509	32830	17	chr18	72212648	72246164	33516	HiConf								y
5444	chr18	72415098	72432774	17676	42	chr18	72415067	72432903	17836	HiConf								
5445	chr18	72735901	72738841	2940	10	chr18	72735870	72738908	3038	HiConf								
5446	chr18	73083997	73102747	18750	26	chr18	73083966	73103125	19159	HiConf	Variation_45693	chr18	73093807	73093987	180	Bentley et al. (2008)	Illumina DNA sequencing	y
5447	chr18	73396197	73396960	763	50	chr18	73396194	73396978	784	single	Variation_39222	chr18	73395986	73397148	1162	Wheeler et al. (2008)	Sequencing	
5448	chr18	73798466	73798673	207	24	chr18	73798435	73798680	245	HiConf	Variation_42717	chr18	73798394	73798922	528	Wang et al. (2008)	Illumina DNA sequencing	
5449	chr18	74074385	74109084	34699	31	chr18	74074354	74109291	34937	HiConf								
5450	chr18	74299540	74309267	9727	25	chr18	74299509	74309505	9996	HiConf	Variation_43304	chr18	74303080	74304470	1390	Wang et al. (2008)	Illumina DNA sequencing	
5451	chr18	74334428	74336780	2352	12	chr18	74334397	74336798	2401	HiConf	Variation_4064	chr18	74125373	74378820	253447	Redon et al. (2006)	BAC Array CGH	
5452	chr18	74393473	74396854	3381	4	chr18	74393442	74396921	3479	single	Variation_1519	chr18	74394310	74397540	3230	Conrad et al. (2005)	Mendelian inconsistencies	
5453	chr18	74402930	74404302	1372	10	chr18	74402899	74404320	1421	single								
5454	chr18	74404498	74408712	4214	15	chr18	74404467	74408779	4312	HiConf								y
5455	chr18	74409006	74415768	6762	22	chr18	74408975	74415884	6909	single								
5456	chr18	74494119	74494326	207	51	chr18	74494088	74494333	245	single	Variation_35599	chr18	74479797	74497358	17561	Kidd et al. (2008)	Paired End Mapping	
5457	chr18	74613483	74617060	3577	18	chr18	74613452	74617127	3675	single								
5458	chr18	74696195	74696832	637	48	chr18	74696164	74696850	686	HiConf	Variation_37574	chr18	74693156	74702191	9035	Cooper et al. (2008)	Illumina Human 1M BeadChip	y
5459	chr18	74715746	74717118	1372	60	chr18	74715715	74717136	1421	HiConf	Variation_42808	chr18	74715287	74715835	548	Wang et al. (2008)	Illumina DNA sequencing	y
5460	chr18	74870831	74898753	27922	46	chr18	74870800	74898779	27979	HiConf	Variation_35602	chr18	74872901	74882050	9149	Kidd et al. (2008)	Paired End Mapping	y
5461	chr18	74899153	74905180	6027	20	chr18	74899122	74905296	6174	single	Variation_35603	chr18	74891685	74906441	14756	Kidd et al. (2008)	Paired End Mapping	
100898	chr18	75041376	75088220	46844	16	chr18	75041345	75089169	47824	HiConf								
5463	chr18	75158804	75159833	1029	25	chr18	75158773	75159851	1078	HiConf	Variation_5927	chr18	75158735	75159972	1237	Mills et al. (2006)	Sequence trace read mapping	y
5464	chr18	75186195	75186783	588	27	chr18	75186164	75186801	637	single								
5465	chr18	75334665	75336625	1960	46	chr18	75334634	75336643	2009	single	Variation_11998	chr18	75334659	75335534	875	Mills et al. (2006)	Sequence trace read mapping	y
5466	chr18	75482750	75508843	26093	16	chr18	75482712	75509123	26411	HiConf	Variation_35604	chr18	75479132	75491762	12630	Kidd et al. (2008)	Paired End Mapping	
5467	chr18	75675460	75679345	3885	2	chr18	75675429	75680721	5292	single								
100899	chr19	11179	217665	206486	15	chr19	11148	221848	210700	HiConf	Variation_30879	chr19	11354	119972	108618	Perry et al. (2008)	Agilent Custom CGH Arrays	
5469	chr19	267057	275338	8281	7	chr19	267026	275503	8477	HiConf	Variation_30884	chr19	274569	276173	1604	Perry et al. (2008)	Agilent Custom CGH Arrays	
5470	chr19	379316	381423	2107	46	chr19	379285	381441	2156	HiConf	Variation_30050	chr19	352714	681297	328583	Jakobsson et al. (2008)	Illumina HumanHap550 BeadChip	y
5471	chr19	2860921	2861371	450	56	chr19	2860890	2861380	490	HiConf	Variation_23559	chr19	2860347	2861527	1180	Levy et al. (2007)	Sequencing	y
5472	chr19	3265367	3285358	19991	14	chr19	3265336	3285867	20531	HiConf	Variation_5072	chr19	3181780	3316288	134508	Wong et al. (2007)	BAC Array CGH	
5473	chr19	3788295	3789275	980	27	chr19	3788264	3789293	1029	HiConf	Variation_30067	chr19	3344464	3806977	462513	Jakobsson et al. (2008)	Illumina HumanHap550 BeadChip	y
5474	chr19	4151434	4151973	539	28	chr19	4151403	4151991	588	single	Variation_5074	chr19	4072257	4225851	153594	Wong et al. (2007)	BAC Array CGH	
5475	chr19	4152169	4173043	20874	2	chr19	4152138	4173453	21315	HiConf	Variation_7202	chr19	4170533	4175602	5069	de Smith et al. (2007)	Agilent 185k CGH Arrays/Agilent Custom CGH Arrays	
5476	chr19	5437733	5438664	931	14	chr19	5437702	5438682	980	HiConf								
5477	chr19	6744955	6746572	1617	3	chr19	6744924	6746590	1666	single	Variation_5342	chr19	6689841	6925438	235597	Simon-Sanchez et al. (2007)	Illumina HumanHap300 BeadChip	
5478	chr19	6803853	6834533	30680	2	chr19	6803822	6835329	31507	HiConf	Variation_37710	chr19	6805266	6833431	28165	McCarroll et al. (2008)	Affymetrix Human SNP Array 6.0	
100900	chr19	6963384	7012627	49243	26	chr19	6963342	7013469	50127	HiConf	Variation_34616	chr19	6902567	7034629	132062	Zogopoulos et al. (2007)	Affymetrix 500K and 100K SNP Mapping Arrays	
5480	chr19	7209769	7209832	63	23	chr19	7209738	7209836	98	single	Variation_8863	chr19	6847380	7787030	939650	Pinto et al. (2007)	Affymetrix 500K SNP Mapping Array	
5481	chr19	7418947	7423747	4800	9	chr19	7416420	7426024	9604	HiConf	Variation_8863	chr19	6847380	7787030	939650	Pinto et al. (2007)	Affymetrix 500K SNP Mapping Array	
5482	chr19	7661945	7664077	2132	8	chr19	7660930	7665046	4116	HiConf	Variation_8863	chr19	6847380	7787030	939650	Pinto et al. (2007)	Affymetrix 500K SNP Mapping Array	
5483	chr19	7714224	7715645	1421	7	chr19	7714193	7715663	1470	single	Variation_8863	chr19	6847380	7787030	939650	Pinto et al. (2007)	Affymetrix 500K SNP Mapping Array	
5484	chr19	8234506	8264634	30128	9	chr19	8234475	8265149	30674	HiConf	Variation_22876	chr19	8241693	8258798	17105	Korbel et al. (2007)	Paired End Mapping	
5485	chr19	8269345	8271060	1715	6	chr19	8269314	8271078	1764	single	Variation_37640	chr19	8256197	8271067	14870	McCarroll et al. (2008)	Affymetrix Human SNP Array 6.0	
5486	chr19	8512924	8519049	6125	13	chr19	8512893	8519165	6272	single								
5487	chr19	8665706	8666882	1176	28	chr19	8665675	8666900	1225	single	Variation_5079	chr19	8665784	8736725	70941	Wong et al. (2007)	BAC Array CGH	y
100901	chr19	8708232	8765499	57267	53	chr19	8695737	8784378	88641	HiConf	Variation_30888	chr19	8708296	8763911	55615	Perry et al. (2008)	Agilent Custom CGH Arrays	
100902	chr19	8854660	8955628	100968	7	chr19	8854570	8957740	103170	single	Variation_35699	chr19	8875808	8909190	33382	Kidd et al. (2008)	Paired End Mapping	
5490	chr19	9084166	9086028	1862	9	chr19	9084135	9086046	1911	HiConf	Variation_5936	chr19	9083945	9086121	2176	Mills et al. (2006)	Sequence trace read mapping	
5491	chr19	9134783	9145122	10339	16	chr19	9134752	9145336	10584	HiConf	Variation_37960	chr19	9135672	9139121	3449	McCarroll et al. (2008)	Affymetrix Human SNP Array 6.0	
5492	chr19	9278112	9281832	3720	13	chr19	9277734	9281899	4165	single								
5493	chr19	9724694	9735082	10388	11	chr19	9724663	9735296	10633	HiConf	Variation_35702	chr19	9715828	9742451	26623	Kidd et al. (2008)	Paired End Mapping	y
5494	chr19	9934659	9947893	13234	23	chr19	9934628	9948005	13377	HiConf	Variation_45822	chr19	9943491	9943630	139	Bentley et al. (2008)	Illumina DNA sequencing	
5495	chr19	10045105	10058874	13769	6	chr19	10045074	10059137	14063	single	Variation_5085	chr19	10049332	10223589	174257	Wong et al. (2007)	BAC Array CGH	y
5496	chr19	11179854	11182836	2982	16	chr19	11178836	11182903	4067	HiConf								
5497	chr19	11900931	11906174	5243	22	chr19	11900900	11906290	5390	HiConf								
100903	chr19	12349408	12445506	96098	6	chr19	12349348	12446050	96702	HiConf	Variation_3180	chr19	12350726	12410655	59929	Redon et al. (2006)	Affymetrix 500K EA SNP Mapping Array	
5499	chr19	12468529	12471389	2860	37	chr19	12468173	12471456	3283	HiConf	Variation_22646	chr19	12466738	12473066	6328	Korbel et al. (2007)	Paired End Mapping	
5500	chr19	12556404	12558413	2009	53	chr19	12556373	12558431	2058	HiConf	Variation_22582	chr19	12554413	12560035	5622	Korbel et al. (2007)	Paired End Mapping	y
5501	chr19	13637197	13637647	450	50	chr19	13637166	13637656	490	HiConf	Variation_47866	chr19	13275594	13727866	452272	Gusev et al. (2009)	SNP genotyping analysis	y
5502	chr19	13908461	13910274	1813	19	chr19	13908430	13910292	1862	HiConf	Variation_5088	chr19	13697718	13923800	226082	Wong et al. (2007)	BAC Array CGH	y
5503	chr19	14120582	14121513	931	14	chr19	14120551	14121531	980	single	Variation_5089	chr19	13991013	14149003	157990	Wong et al. (2007)	BAC Array CGH	
5504	chr19	14153809	14155849	2040	1	chr19	14143238	14160241	17003	single								
5505	chr19	14555604	14575605	20001	10	chr19	14555573	14576006	20433	HiConf								
5506	chr19	14776398	14783748	7350	5	chr19	14776367	14783913	7546	HiConf	Variation_38379	chr19	14776641	14783883	7242	McCarroll et al. (2008)	Affymetrix Human SNP Array 6.0	y
5507	chr19	14906248	14910412	4164	50	chr19	14906217	14910480	4263	HiConf	Variation_43982	chr19	14907385	14910474	3089	Bentley et al. (2008)	Illumina DNA sequencing	
5508	chr19	15007531	15039283	31752	7	chr19	15007500	15039938	32438	HiConf	Variation_4068	chr19	14957950	15120179	162229	Redon et al. (2006)	BAC Array CGH	
5509	chr19	15525706	15537734	12028	6	chr19	15525675	15538072	12397	HiConf	Variation_10544	chr19	15526528	15529140	2612	Wang et al. (2007)	Illumina HumanHap550 BeadChip	
100905	chr19	15638800	15696111	57311	50	chr19	15632936	15697028	64092	HiConf	Variation_30890	chr19	15639244	15694434	55190	Perry et al. (2008)	Agilent Custom CGH Arrays	y
5511	chr19	15892569	15921989	29420	44	chr19	15892538	15922330	29792	HiConf								
5512	chr19	15964452	15966314	1862	4	chr19	15964421	15966332	1911	HiConf								
5513						chr19	18102340	18113120	10780	single								
5514	chr19	19699379	19700555	1176	59	chr19	19699348	19700573	1225	HiConf	Variation_7194	chr19	19699073	19700649	1576	de Smith et al. (2007)	Agilent 185k CGH Arrays/Agilent Custom CGH Arrays	
100907	chr19	20372937	20535487	162550	4	chr19	20372657	20538767	166110	HiConf	Variation_35617	chr19	20370872	20521031	150159	Kidd et al. (2008)	Paired End Mapping	
5516	chr19	20593255	20593686	431	46	chr19	20593255	20593696	441	HiConf	Variation_33320	chr19	20592814	20593683	869	Perry et al. (2008)	Agilent Custom CGH Arrays	
100908	chr19	20836106	20903060	66954	6	chr19	20836075	20904405	68330	HiConf								
5518	chr19	20949026	20957827	8801	2	chr19	20948995	20958207	9212	HiConf	Variation_10548	chr19	20949974	20956239	6265	Wang et al. (2007)	Illumina HumanHap550 BeadChip	
5519	chr19	21541338	21548669	7331	36	chr19	21541307	21548853	7546	HiConf	Variation_5094	chr19	21455378	21637562	182184	Wong et al. (2007)	BAC Array CGH	
5520	chr19	22207159	22239000	31841	4	chr19	22207119	22239655	32536	HiConf	Variation_30892	chr19	22201569	22239807	38238	Perry et al. (2008)	Agilent Custom CGH Arrays	
5521	chr19	22796179	22929508	133329	5	chr19	22796148	22932221	136073	HiConf	Variation_9792	chr19	22792585	23412472	619887	Wang et al. (2007)	Illumina HumanHap550 BeadChip	
5522	chr19	22932840	22934800	1960	53	chr19	22932809	22934818	2009	HiConf	Variation_22537	chr19	22930973	22935770	4797	Korbel et al. (2007)	Paired End Mapping	
5523	chr19	23167305	23170374	3069	23	chr19	23167274	23170655	3381	HiConf	Variation_9792	chr19	22792585	23412472	619887	Wang et al. (2007)	Illumina HumanHap550 BeadChip	y
5524	chr19	23253888	23258078	4190	26	chr19	23253857	23258120	4263	HiConf	Variation_5946	chr19	23257033	23258677	1644	Mills et al. (2006)	Sequence trace read mapping	
5525	chr19	23598015	23598123	108	25	chr19	23597984	23598131	147	single	Variation_32241	chr19	23596541	23599600	3059	Perry et al. (2008)	Agilent Custom CGH Arrays	
5526	chr19	23648907	23659333	10426	8	chr19	23648552	23659528	10976	HiConf	Variation_4072	chr19	22774966	23732307	957341	Redon et al. (2006)	BAC Array CGH	
100910	chr19	32435420	32766842	331422	28	chr19	28399837	32769118	4369281	single	Variation_5096	chr19	32535227	32599962	64735	Wong et al. (2007)	BAC Array CGH	

locus_id	chrom	start	end	length	Yoruba w/ event	putative chr	putative start	putative end	putative len	putative type	variation_id	DGV_chr	DGV_start	DGV_end	DGV_len	Reference	Method/platform	complex
5527	chr19	32942413	32944814	2401	4	chr19	32942382	32944832	2450	single	Variation_22529	chr19	32942106	32946759	4653	Korbel et al. (2007)	Paired End Mapping	
5528	chr19	33053496	33059229	5733	3	chr19	33053465	33059345	5880	HiConf								
5529	chr19	33096665	33102398	5733	6	chr19	33096634	33102514	5880	HiConf								
5530	chr19	33268116	33270300	2184	3	chr19	33268085	33276513	8428	HiConf								y
5531	chr19	33463185	33467791	4606	36	chr19	33463154	33467858	4704	HiConf								y
5532	chr19	34029282	34031928	2646	13	chr19	34029251	34031995	2744	HiConf	Variation_37864	chr19	34029192	34031422	2230	McCarroll et al. (2008)	Affymetrix Human SNP Array 6.0	
5533	chr19	34648495	34650063	1568	5	chr19	34648464	34650081	1617	single	Variation_23132	chr19	34641983	34649280	7297	Korbel et al. (2007)	Paired End Mapping	
5534	chr19	34877913	34879285	1372	5	chr19	34877882	34879303	1421	single								
5535	chr19	35606200	35608013	1813	29	chr19	35606169	35608031	1862	single								
5536	chr19	35979433	35981246	1813	44	chr19	35979402	35981264	1862	HiConf	Variation_43986	chr19	35979316	35981595	2279	Bentley et al. (2008)	Illumina DNA sequencing	
5537	chr19	36007608	36010352	2744	8	chr19	36007577	36010419	2842	HiConf								
5538	chr19	38160714	38211432	50718	6	chr19	38160343	38212479	52136	HiConf								
100912	chr19	38607757	39208452	600695	3	chr19	38607713	39211687	603974	HiConf	Variation_8871	chr19	38589007	39215623	626616	Pinto et al. (2007)	Affymetrix 500K SNP Mapping Array	
5541	chr19	39832156	39839751	7595	19	chr19	39832125	39839916	7791	HiConf	Variation_32245	chr19	39832668	39839521	6853	Perry et al. (2008)	Agilent Custom CGH Arrays	
5542	chr19	40031929	40034281	2352	3	chr19	40031898	40034299	2401	HiConf								
5543	chr19	40353075	40356701	3626	8	chr19	40353044	40356768	3724	single	Variation_32246	chr19	40353026	40356667	3641	Perry et al. (2008)	Agilent Custom CGH Arrays	y
5544	chr19	40548566	40553411	4845	13	chr19	40541106	40557276	16170	single	Variation_37514	chr19	40542993	40553524	10531	Cooper et al. (2008)	Illumina Human 1M BeadChip	
5545	chr19	41496533	41498548	2015	15	chr19	41496165	41498566	2401	single	Variation_35638	chr19	41496309	41541512	45203	Kidd et al. (2008)	Paired End Mapping	
5546	chr19	41532923	41539325	6402	19	chr19	41532572	41539334	6762	HiConf	Variation_39233	chr19	41532593	41538914	6321	Wheeler et al. (2008)	Sequencing	
5547	chr19	41650989	41651771	782	13	chr19	41650613	41651789	1176	single								
5548	chr19	42314300	42315819	1519	1	chr19	42314269	42315837	1568	single								y
100913	chr19	42433909	42509271	75362	48	chr19	42433878	42510808	76930	HiConf	Variation_30903	chr19	42453000	42486487	33487	Perry et al. (2008)	Agilent Custom CGH Arrays	
5550	chr19	43830654	43831242	588	42	chr19	43830623	43831260	637	single								
5551						chr19	44619180	44619229	49	single								
100914	chr19	45055567	45108060	52493	7	chr19	45055354	45109131	53777	HiConf	Variation_35641	chr19	45060871	45083094	22223	Kidd et al. (2008)	Paired End Mapping	
5553	chr19	45345440	45372540	27100	20	chr19	45345409	45373094	27685	HiConf	Variation_7204	chr19	45355279	45361177	5898	de Smith et al. (2007)	Agilent 185k CGH Arrays/Agilent Custom CGH Arrays	
5554	chr19	45448639	45461031	12392	20	chr19	45447917	45461294	13377	single	Variation_4080	chr19	45304260	45531360	227100	Redon et al. (2006)	BAC Array CGH	
100915	chr19	46021702	46092467	70765	10	chr19	46021609	46093100	71491	HiConf	Variation_7205	chr19	46032469	46092475	60006	de Smith et al. (2007)	Agilent 185k CGH Arrays/Agilent Custom CGH Arrays	y
100916	chr19	46137777	46219502	81725	3	chr19	46104713	46221848	117135	HiConf	Variation_3191	chr19	46123775	46220507	96732	Redon et al. (2006)	Affymetrix 500K EA SNP Mapping Array	y
5558	chr19	46384289	46389105	4816	14	chr19	46384258	46389207	4949	HiConf								y
5559	chr19	46397078	46402762	5684	4	chr19	46397047	46402878	5831	single								
100917	chr19	46658560	46700584	42024	8	chr19	46658511	46701460	42949	HiConf	Variation_38383	chr19	46668500	46699711	31211	McCarroll et al. (2008)	Affymetrix Human SNP Array 6.0	
5561	chr19	46879728	46883452	3724	1	chr19	46879697	46883519	3822	single	Variation_37707	chr19	46879180	46883135	3955	McCarroll et al. (2008)	Affymetrix Human SNP Array 6.0	
5562	chr19	47134115	47136047	1932	3	chr19	47131508	47147629	16121	single								
5563	chr19	47727379	47728947	1568	11	chr19	47727348	47728965	1617	HiConf	Variation_5446	chr19	47683129	48360867	677738	Simon-Sanchez et al. (2007)	Illumina HumanHap300 BeadChip	
100918	chr19	47896316	48489146	592830	15	chr19	47896276	48493439	597163	HiConf	Variation_30908	chr19	47906372	48490564	584192	Perry et al. (2008)	Agilent Custom CGH Arrays	
5565	chr19	48537055	48539554	2499	4	chr19	48537024	48539621	2597	single	Variation_2244	chr19	48405049	48578186	173137	Locke et al. (2006)	BAC Array CGH	
5566	chr19	48542445	48542553	108	21	chr19	48542414	48542561	147	single	Variation_2244	chr19	48405049	48578186	173137	Locke et al. (2006)	BAC Array CGH	
5567	chr19	49095704	49096341	637	10	chr19	49095673	49096359	686	single								
5568	chr19	49584675	49629657	44982	7	chr19	49584644	49630557	45913	HiConf	Variation_32260	chr19	49585124	49630732	45608	Perry et al. (2008)	Agilent Custom CGH Arrays	
100920	chr19	49648591	49688114	39523	6	chr19	49648491	49688916	40425	HiConf								
5571	chr19	51175068	51183153	8085	2	chr19	51175037	51183318	8281	HiConf	Variation_35650	chr19	51171349	51176350	5001	Kidd et al. (2008)	Paired End Mapping	
5572	chr19	51314620	51320010	5390	15	chr19	51314589	51320126	5537	HiConf								
5573	chr19	51640715	51654969	14254	8	chr19	51640684	51655090	14406	HiConf	Variation_38386	chr19	51650847	51653422	2575	McCarroll et al. (2008)	Affymetrix Human SNP Array 6.0	
5574	chr19	51831619	51832452	833	7	chr19	51831588	51832470	882	single								
5575	chr19	51865911	51868649	2738	13	chr19	51865496	51868730	3234	single								
5576	chr19	53509673	53512417	2744	11	chr19	53509642	53512484	2842	single	Variation_5106	chr19	53445212	53619236	174024	Wong et al. (2007)	BAC Array CGH	
5577	chr19	53920099	53926516	6417	6	chr19	53920066	53926632	6566	single	Variation_32261	chr19	53097718	55337070	2239352	Perry et al. (2008)	Agilent Custom CGH Arrays	
5578						chr19	54325051	54338379	13328	single								y
5579	chr19	54633249	54639646	6397	18	chr19	54620570	54648108	27538	HiConf	Variation_26309	chr19	54639198	54639343	145	Levy et al. (2007)	Sequencing	
5580	chr19	54979134	54986631	7497	11	chr19	54979103	54986796	7693	single	Variation_5114	chr19	54928889	55138293	209404	Wong et al. (2007)	BAC Array CGH	
5581	chr19	55200222	55200908	686	40	chr19	55200191	55200926	735	single	Variation_8874	chr19	55190200	55477198	286998	Pinto et al. (2007)	Affymetrix 500K SNP Mapping Array	
5582	chr19	55246723	55252897	6174	47	chr19	55246692	55253013	6321	HiConf	Variation_7209	chr19	55250216	55252940	2724	de Smith et al. (2007)	Agilent 185k CGH Arrays/Agilent Custom CGH Arrays	
5583	chr19	55589778	55592842	3064	1	chr19	55581558	55600962	19404	single								
5584	chr19	55702178	55702488	310	18	chr19	55702147	55702490	343	HiConf								
5585	chr19	55720095	55723983	3888	3	chr19	55716014	55724148	8134	single								
5586	chr19	55769063	55773865	4802	46	chr19	55769032	55773932	4900	HiConf								
5587	chr19	55798708	55803363	4655	3	chr19	55798677	55803430	4753	HiConf	Variation_7211	chr19	55802063	55816357	14294	de Smith et al. (2007)	Agilent 185k CGH Arrays/Agilent Custom CGH Arrays	
5588						chr19	55877861	55886632	8771	single								
100921						chr19	56502562	56547397	44835	single								
5589	chr19	56569821	56569965	144	12	chr19	56569790	56569986	196	HiConf								
5590	chr19	56822710	56841722	19012	57	chr19	56822679	56842083	19404	HiConf	Variation_37516	chr19	56824337	56840796	16459	Cooper et al. (2008)	Illumina Human 1M BeadChip	
5591	chr19	56901600	56918162	16562	2	chr19	56901569	56918474	16905	single	Variation_30915	chr19	56901393	56912374	10981	Perry et al. (2008)	Agilent Custom CGH Arrays	
5592	chr19	57454211	57463133	8922	5	chr19	57453799	57463207	9408	HiConf								
5593	chr19	57580740	57582406	1666	56	chr19	57580709	57582424	1715	HiConf	Variation_7216	chr19	57580834	57582921	2087	de Smith et al. (2007)	Agilent 185k CGH Arrays/Agilent Custom CGH Arrays	
5594	chr19	58014047	58050905	36858	5	chr19	58014016	58051599	37583	single	Variation_30916	chr19	58016405	58048790	32385	Perry et al. (2008)	Agilent Custom CGH Arrays	y
5595	chr19	58197699	58197906	207	14	chr19	58197668	58197913	245	single	Variation_9311	chr19	58169921	58201500	31579	Pinto et al. (2007)	Affymetrix 500K SNP Mapping Array	
5596	chr19	58619108	58696911	77803	1	chr19	58619068	58698497	79429	single	Variation_29695	chr19	58624107	58705989	81882	Jakobsson et al. (2008)	Illumina HumanHap550 BeadChip	
5597	chr19	58872135	58872495	360	22	chr19	58872104	58872496	392	single								
5598	chr19	59183759	59188244	4485	4	chr19	59180608	59190359	9751	HiConf	Variation_5115	chr19	59041022	59190183	149161	Wong et al. (2007)	BAC Array CGH	
5599	chr19	59247671	59251888	4217	29	chr19	59247640	59252246	4606	HiConf	Variation_30919	chr19	59245616	59252269	6653	Perry et al. (2008)	Agilent Custom CGH Arrays	
5600	chr19	59417848	59439849	22001	40	chr19	59417817	59440308	22491	HiConf	Variation_37528	chr19	59418356	59439332	20976	Cooper et al. (2008)	Illumina Human 1M BeadChip	
5601	chr19	59488212	59488512	300	23	chr19	59488181	59488524	343	single	Variation_35683	chr19	59487830	59501472	13642	Kidd et al. (2008)	Paired End Mapping	
5602	chr19	59495072	59498820	3748	8	chr19	59495041	59499157	4116	single	Variation_39241	chr19	59492668	59499432	6764	Wheeler et al. (2008)	Sequencing	
5603	chr19	59641190	59641729	539	26	chr19	59641159	59641747	588	single	Variation_5116	chr19	59464633	59721885	257252	Wong et al. (2007)	BAC Array CGH	
5604	chr19	59644179	59651088	6909	20	chr19	59644148	59651204	7056	single	Variation_5116	chr19	59464633	59721885	257252	Wong et al. (2007)	BAC Array CGH	
5605	chr19	59653538	59653940	402	8	chr19	59653507	59653948	441	single	Variation_5116	chr19	59464633	59721885	257252	Wong et al. (2007)	BAC Array CGH	
100923	chr19	59934676	60068847	134171	4	chr19	59934645	60071551	136906	HiConf	Variation_8876	chr19	59939919	60074863	134944	Pinto et al. (2007)	Affymetrix 500K SNP Mapping Array	y
5608	chr19	60236148	60236687	539	54	chr19	60236117	60236705	588	single	Variation_3201	chr19	60229274	60342499	113225	Redon et al. (2006)	Affymetrix 500K EA SNP Mapping Array	
5609	chr19	60829736	60836712	6976	1	chr19	60829703	60887866	58163	single								
100924	chr19	60958310	60981795	23485	21	chr19	60958279	60982240	23961	HiConf	Variation_23204	chr19	60961580	60974440	12860	Korbel et al. (2007)	Paired End Mapping	
5611	chr19	61173773	61218818	45045	41	chr19	61173732	61219743	46011	HiConf								
5612						chr19	61295154	61315440	20286	HiConf								
5613	chr19	61405337	61413520	8183	9	chr19	61405306	61413685	8379	HiConf	Variation_38388	chr19	61405760	61414571	8811	McCarroll et al. (2008)	Affymetrix Human SNP Array 6.0	
5614	chr19	61558217	61561157	2940	23	chr19	61558186	61561224	3038	HiConf	Variation_43991	chr19	61558263	61561734	3471	Bentley et al. (2008)	Illumina DNA sequencing	
5615	chr19	61894308	61903569	9261	11	chr19	61894277	61903734	9457	HiConf	Variation_38390	chr19	61896070	61903319	7249	McCarroll et al. (2008)	Affymetrix Human SNP Array 6.0	

locus_id	chrom	start	end	length	Yoruba w/ event	putative chr	putative start	putative end	putative len	putative type	variation_id	DGV_chr	DGV_start	DGV_end	DGV_len	Reference	Method/platform	complex
5616	chr19	62219276	62220305	1029	26	chr19	62219245	62220323	1078	single								
5617	chr19	62308309	62310318	2009	23	chr19	62308278	62310336	2058	single								
5618	chr19	62401507	62405966	4459	16	chr19	62401476	62406033	4557	single								
5619						chr19	62469145	62469537	392	single								
5620	chr19	62469617	62471087	1470	10	chr19	62469586	62471105	1519	HiConf								
5621	chr19	63231714	63234703	2989	2	chr19	63231683	63234770	3087	single	Variation_5118	chr19	63182937	63368540	185603	Wong et al. (2007)	BAC Array CGH	
503	chr2	296310	299326	3016	51	chr2	296279	299366	3087	single	Variation_32277	chr2	296699	298661	1962	Perry et al. (2008)	Agilent Custom CGH Arrays	y
504	chr2	299544	300132	588	42	chr2	299513	300150	637	single	Variation_8356	chr2	286852	836164	549312	Pinto et al. (2007)	Affymetrix 500K SNP Mapping Array	
505	chr2	411242	415870	4628	5	chr2	411233	415937	4704	single	Variation_30931	chr2	305360	846457	541097	Perry et al. (2008)	Agilent Custom CGH Arrays	
506	chr2	426454	427336	882	28	chr2	426423	427354	931	single	Variation_30931	chr2	305360	846457	541097	Perry et al. (2008)	Agilent Custom CGH Arrays	
507	chr2	714819	738829	24010	5	chr2	714788	739288	24500	HiConf	Variation_46034	chr2	715530	715667	137	Bentley et al. (2008)	Illumina DNA sequencing	
508	chr2	827274	852362	25088	29	chr2	827243	852870	25627	HiConf	Variation_41899	chr2	849620	850030	410	Wang et al. (2008)	Illumina DNA sequencing	
509	chr2	994903	995442	539	43	chr2	994872	995460	588	HiConf	Variation_42241	chr2	995135	995595	460	Wang et al. (2008)	Illumina DNA sequencing	
510	chr2	1097852	1101501	3649	34	chr2	1097821	1101594	3773	HiConf								y
511	chr2	1405278	1452226	46948	50	chr2	1405247	1452483	47236	HiConf	Variation_2362	chr2	1410597	1484084	73487	Redon et al. (2006)	Affymetrix 500K EA SNP Mapping Array	
513	chr2	1505532	1520279	14747	8	chr2	1505501	1520495	14994	HiConf	Variation_43574	chr2	1512357	1517129	4772	Wang et al. (2008)	Illumina DNA sequencing	
514	chr2	1538166	1538901	735	46	chr2	1538135	1538919	784	single								y
515	chr2	1547770	1550122	2352	34	chr2	1547739	1550140	2401	HiConf								
516	chr2	1689331	1690269	938	50	chr2	1689300	1690280	980	single	Variation_26486	chr2	1689388	1689518	130	Levy et al. (2007)	Sequencing	y
517	chr2	1700503	1701679	1176	23	chr2	1700472	1701697	1225	HiConf								
518	chr2	1930117	1936072	5955	16	chr2	1930086	1936162	6076	single	Variation_3342	chr2	1875438	2155707	280269	Redon et al. (2006)	BAC Array CGH	
519	chr2	1987643	1988868	1225	15	chr2	1987612	1988886	1274	single	Variation_45927	chr2	1987848	1988418	570	Bentley et al. (2008)	Illumina DNA sequencing	
520	chr2	2157526	2158457	931	9	chr2	2157495	2158475	980	single								
521	chr2	2563050	2564128	1078	11	chr2	2563019	2564146	1127	HiConf								
522	chr2	2890566	2890674	108	37	chr2	2890535	2890682	147	single								
523	chr2	2892036	2892771	735	40	chr2	2892005	2892789	784	single								
524	chr2	3200981	3204558	3577	17	chr2	3200950	3204625	3675	single								
525	chr2	3258507	3260075	1568	7	chr2	3258476	3260093	1617	single								
526	chr2	3292415	3294816	2401	11	chr2	3292384	3294834	2450	single								
527	chr2	3697498	3697858	360	41	chr2	3697467	3697859	392	single	Variation_9394	chr2	3623304	3713701	90397	Wang et al. (2007)	Illumina HumanHap550 BeadChip	
528	chr2	4422992	4442298	19306	32	chr2	4422961	4442708	19747	HiConf								
529	chr2	4629089	4632075	2986	7	chr2	4629055	4632142	3087	HiConf	Variation_44027	chr2	4629530	4631946	2416	Bentley et al. (2008)	Illumina DNA sequencing	
530	chr2	4983258	5098653	115395	4	chr2	4983227	5101023	117796	single								
531	chr2	5168086	5170095	2009	2	chr2	5168055	5170113	2058	single								y
532	chr2	5367075	5367372	297	40	chr2	5367044	5367387	343	HiConf								
100088						chr2	5679052	5714503	35451	HiConf								
534	chr2	6215853	6225947	10094	18	chr2	6215822	6226161	10339	HiConf	Variation_38434	chr2	6215763	6225305	9542	McCarroll et al. (2008)	Affymetrix Human SNP Array 6.0	
535	chr2	6240205	6240277	72	1	chr2	6240126	6240763	637	single	Variation_35845	chr2	6208099	6254823	46724	Kidd et al. (2008)	Paired End Mapping	
536	chr2	6252310	6253654	1344	2	chr2	6249975	6254777	4802	single	Variation_35845	chr2	6208099	6254823	46724	Kidd et al. (2008)	Paired End Mapping	y
537	chr2	6317283	6319920	2637	58	chr2	6317252	6319947	2695	HiConf	Variation_5967	chr2	6317960	6319644	1684	Mills et al. (2006)	Sequence trace read mapping	
538	chr2	7147441	7153027	5586	8	chr2	7147410	7153143	5733	HiConf	Variation_1026	chr2	7148439	7149929	1490	Conrad et al. (2005)	Mendelian inconsistencies	y
539	chr2	7531454	7562602	31148	59	chr2	7531423	7562930	31507	HiConf								
540	chr2	8898211	8901396	3185	5	chr2	8898180	8901463	3283	single								
541	chr2	9359301	9367141	7840	34	chr2	9359270	9367306	8036	HiConf								
542	chr2	9463671	9464071	400	54	chr2	9463640	9464081	441	HiConf								
543	chr2	9777467	9799899	22432	18	chr2	9777436	9800368	22932	HiConf	Variation_29698	chr2	9774469	9876222	101753	Jakobsson et al. (2008)	Illumina HumanHap550 BeadChip	y
544	chr2	9842637	9846388	3751	39	chr2	9842606	9846428	3822	HiConf	Variation_44040	chr2	9842929	9846502	3573	Bentley et al. (2008)	Illumina DNA sequencing	
546	chr2	11157160	11180484	23324	20	chr2	11157129	11180943	23814	HiConf								
547	chr2	11312851	11317586	4735	10	chr2	11312851	11317653	4802	HiConf	Variation_22655	chr2	11312440	11318844	6404	Korbel et al. (2007)	Paired End Mapping	
548	chr2	11503198	11503982	784	37	chr2	11503167	11504000	833	HiConf								
549	chr2	11911417	11912642	1225	40	chr2	11911386	11912660	1274	HiConf								y
550	chr2	13426497	13485444	58947	17	chr2	13426466	13486638	60172	HiConf	Variation_30936	chr2	13433391	13478875	45484	Perry et al. (2008)	Agilent Custom CGH Arrays	
551	chr2	13497204	13514648	17444	10	chr2	13497173	13515009	17836	HiConf	Variation_30937	chr2	13499719	13515172	15453	Perry et al. (2008)	Agilent Custom CGH Arrays	
552	chr2	14107793	14118916	11123	4	chr2	14107762	14119130	11368	HiConf	Variation_30086	chr2	14095364	14204168	108804	Jakobsson et al. (2008)	Illumina HumanHap550 BeadChip	
553	chr2	14369894	14372694	2800	4	chr2	14369863	14373391	3528	HiConf								
554	chr2	14623910	14627144	3234	2	chr2	14623879	14627211	3332	single	Variation_38406	chr2	14623720	14627544	3824	McCarroll et al. (2008)	Affymetrix Human SNP Array 6.0	
555	chr2	14770371	14773850	3479	11	chr2	14770340	14773917	3577	HiConf								
556	chr2	15202649	15205393	2744	7	chr2	15202618	15205460	2842	HiConf	Variation_3345	chr2	15119640	15275310	155670	Redon et al. (2006)	BAC Array CGH	
557	chr2	15583526	15610280	26754	10	chr2	15583495	15610837	27342	single								
558	chr2	16424219	16424529	310	30	chr2	16424188	16424531	343	HiConf	Variation_47944	chr2	16383293	16737520	354227	Gusev et al. (2009)	SNP genotyping analysis	
559	chr2	16800490	16801225	735	6	chr2	16800459	16801243	784	single								y
560	chr2	17450916	17453856	2940	64	chr2	17450885	17453923	3038	HiConf	Variation_39244	chr2	17448608	17454091	5483	Wheeler et al. (2008)	Sequencing	
561	chr2	17470026	17471349	1323	3	chr2	17469995	17471367	1372	single								
100093	chr2	17545658	17611171	65513	5	chr2	17545627	17612512	66885	single								
562	chr2	17982615	17983065	450	38	chr2	17982584	17983074	490	HiConf	Variation_44009	chr2	17982350	17983496	1146	Bentley et al. (2008)	Illumina DNA sequencing	
563	chr2	17987809	17994865	7056	14	chr2	17987778	17994981	7203	single								
564	chr2	18035157	18056213	21056	3	chr2	18034230	18056672	22442	HiConf	Variation_1567	chr2	18035293	18056322	21029	McCarroll et al. (2005)	Mendelian inconsistencies/Null genotypes	
565	chr2	18300331	18302534	2203	3	chr2	18300300	18302554	2254	HiConf	Variation_8365	chr2	18285494	18876595	591101	Pinto et al. (2007)	Affymetrix 500K SNP Mapping Array	
100094	chr2	18597709	18633204	35495	1	chr2	18588518	18645775	57257	HiConf	Variation_45914	chr2	18616413	18616545	132	Bentley et al. (2008)	Illumina DNA sequencing	
567	chr2	19271462	19273079	1617	5	chr2	19271431	19273097	1666	HiConf								
568	chr2	19631122	19633964	2842	49	chr2	19631091	19634031	2940	HiConf	Variation_44011	chr2	19631059	19634044	2985	Bentley et al. (2008)	Illumina DNA sequencing	
569	chr2	20192123	20197066	4943	3	chr2	20192092	20197139	5047	HiConf	Variation_35777	chr2	20180002	20212669	32667	Kidd et al. (2008)	Paired End Mapping	
570	chr2	20197317	20200269	2952	2	chr2	20197286	20200324	3038	HiConf	Variation_35777	chr2	20180002	20212669	32667	Kidd et al. (2008)	Paired End Mapping	
571	chr2	20878172	20886012	7840	2	chr2	20878141	20886177	8036	single								
572	chr2	22182160	22184904	2744	3	chr2	22182129	22184971	2842	HiConf	Variation_7250	chr2	22181860	22185387	3527	de Smith et al. (2007)	Agilent 185k CGH Arrays/Agilent Custom CGH Arrays	
573	chr2	22400161	22404032	3871	3	chr2	22400130	22404099	3969	HiConf								
574	chr2	22542996	22543256	260	24	chr2	22542965	22543259	294	single								
575	chr2	23319940	23328368	8428	1	chr2	23319909	23328533	8624	single								
100095	chr2	23686439	23690663	4224	1	chr2	23686380	23728937	42557	single								
576	chr2	24453202	24465966	12764	8	chr2	24452887	24466362	13475	HiConf	Variation_37406	chr2	24453419	24464972	11553	Cooper et al. (2008)	Illumina Human 1M BeadChip	
577	chr2	25940509	25940999	490	8	chr2	25940478	25941017	539	single	Variation_7261	chr2	25516390	26749172	1232782	de Smith et al. (2007)	Agilent 185k CGH Arrays/Agilent Custom CGH Arrays	
578	chr2	26375629	26381313	5684	1	chr2	26375598	26381429	5831	single	Variation_7261	chr2	25516390	26749172	1232782	de Smith et al. (2007)	Agilent 185k CGH Arrays/Agilent Custom CGH Arrays	
100096	chr2	26409905	26489115	79210	1	chr2	26409874	26490797	80923	HiConf	Variation_35804	chr2	26417396	26436915	19519	Kidd et al. (2008)	Paired End Mapping	
580	chr2	27614055	27632687	18632	3	chr2	27614024	27633036	19012	single	Variation_9400	chr2	27510671	27700107	189436	Wang et al. (2007)	Illumina HumanHap550 BeadChip	
581	chr2	27701863	27703970	2107	22	chr2	27701832	27703988	2156	single								
582	chr2	29495214	29498693	3479	7	chr2	29495183	29498760	3577	HiConf	Variation_32289	chr2	29495154	29498246	3092	Perry et al. (2008)	Agilent Custom CGH Arrays	

locus_id	chrom	start	end	length	Yoruba w/ event	putative chr	putative start	putative end	putative len	putative type	variation_id	DGV_chr	DGV_start	DGV_end	DGV_len	Reference	Method/platform	complex
583	chr2	29811705	29816752	5047	1	chr2	29811674	29816868	5194	single	Variation_3348	chr2	29207771	30392933	1185162	Redon et al. (2006)	BAC Array CGH	
584	chr2	29898755	29917006	18251	6	chr2	29898698	29917367	18669	HiConf	Variation_3348	chr2	29207771	30392933	1185162	Redon et al. (2006)	BAC Array CGH	
585	chr2	30334939	30335119	180	1	chr2	30334308	30335827	1519	single	Variation_3348	chr2	29207771	30392933	1185162	Redon et al. (2006)	BAC Array CGH	
586	chr2	31563455	31564141	686	4	chr2	31563424	31564159	735	single	Variation_35808	chr2	31563921	31587266	23345	Kidd et al. (2008)	Paired End Mapping	
587	chr2	31580409	31580669	260	49	chr2	31580378	31580672	294	single	Variation_35808	chr2	31563921	31587266	23345	Kidd et al. (2008)	Paired End Mapping	
588	chr2	31658515	31661994	3479	3	chr2	31658484	31662061	3577	single								
589	chr2	33076624	33080691	4067	17	chr2	33076593	33080758	4165	HiConf	Variation_43507	chr2	33077923	33080880	2957	Wang et al. (2008)	Illumina DNA sequencing	
590	chr2	33364548	33372241	7693	1	chr2	33364517	33372406	7889	single								y
591	chr2	34377280	34379338	2058	79	chr2	34377249	34379356	2107	HiConf	Variation_43427	chr2	34377127	34379374	2247	Wang et al. (2008)	Illumina DNA sequencing	
592	chr2	34680835	34682657	1822	23	chr2	34680804	34682666	1862	HiConf	Variation_44023	chr2	34680713	34682821	2108	Bentley et al. (2008)	Illumina DNA sequencing	
593	chr2	34914075	34921915	7840	4	chr2	34914044	34922080	8036	HiConf	Variation_38425	chr2	34917577	34920174	2597	McCarroll et al. (2008)	Affymetrix Human SNP Array 6.0	
594	chr2	34932450	34946366	13916	9	chr2	34932419	34946629	14210	single								
595	chr2	35182644	35185237	2593	4	chr2	35182613	35192658	10045	HiConf	Variation_3349	chr2	35091247	35258488	167241	Redon et al. (2006)	BAC Array CGH	
100097	chr2	35424970	35469879	44909	9	chr2	35412962	35487859	74897	HiConf	Variation_30088	chr2	35433195	35467948	34753	Jakobsson et al. (2008)	Illumina HumanHap550 BeadChip	
597	chr2	35826749	35845908	19159	48	chr2	35826718	35846269	19551	HiConf	Variation_37409	chr2	35829787	35845226	15439	Cooper et al. (2008)	Illumina Human 1M BeadChip	
598	chr2	36189839	36192983	3144	14	chr2	36189808	36193042	3234	HiConf	Variation_38427	chr2	36190123	36193058	2935	McCarroll et al. (2008)	Affymetrix Human SNP Array 6.0	y
599	chr2	36252804	36267749	14945	20	chr2	36252773	36268061	15288	HiConf	Variation_8939	chr2	36236970	36275853	38883	Pinto et al. (2007)	Affymetrix 500K SNP Mapping Array	
600	chr2	36690227	36695274	5047	4	chr2	36690196	36695390	5194	HiConf	Variation_35824	chr2	36676494	36701675	25181	Kidd et al. (2008)	Paired End Mapping	
601	chr2	37000544	37016910	16366	15	chr2	37000513	37017222	16709	HiConf								
602	chr2	37231285	37264622	33337	15	chr2	37231254	37265211	33957	HiConf								
100100	chr2	37812346	37853316	40970	3	chr2	37794240	37854510	60270	HiConf	Variation_30940	chr2	37810930	37854438	43508	Perry et al. (2008)	Agilent Custom CGH Arrays	
100101						chr2	37990583	38125039	134456	HiConf								
604	chr2	38154249	38154411	162	11	chr2	38154218	38154414	196	single								
605	chr2	38318534	38319134	600	5	chr2	38318368	38319152	784	HiConf	Variation_32298	chr2	38299071	38810308	511237	Perry et al. (2008)	Agilent Custom CGH Arrays	
606	chr2	38726128	38735340	9212	8	chr2	38726097	38735505	9408	HiConf	Variation_9927	chr2	38727681	38734610	6929	Wang et al. (2007)	Illumina HumanHap550 BeadChip	
607	chr2	39198145	39200301	2156	2	chr2	39198114	39200319	2205	single								
608	chr2	39820347	39823856	3509	2	chr2	39820316	39823942	3626	HiConf								
609	chr2	40465628	40468666	3038	15	chr2	40465597	40468733	3136	HiConf	Variation_32299	chr2	40466052	40467549	1497	Perry et al. (2008)	Agilent Custom CGH Arrays	
610	chr2	40618116	40624731	6615	5	chr2	40618085	40624847	6762	HiConf	Variation_38429	chr2	40617671	40622406	4735	McCarroll et al. (2008)	Affymetrix Human SNP Array 6.0	
611	chr2	40715430	40716214	784	3	chr2	40715399	40716232	833	single	Variation_30094	chr2	40688789	40716539	27750	Jakobsson et al. (2008)	Illumina HumanHap550 BeadChip	
612	chr2	40921769	40922651	882	4	chr2	40921738	40922669	931	HiConf								
613	chr2	41091897	41104931	13034	44	chr2	41091866	41105194	13328	HiConf	Variation_32301	chr2	41092324	41101967	9643	Perry et al. (2008)	Agilent Custom CGH Arrays	
614	chr2	41629525	41630554	1029	19	chr2	41629494	41630572	1078	HiConf	Variation_44024	chr2	41629468	41634785	5317	Bentley et al. (2008)	Illumina DNA sequencing	
615	chr2	41775545	41776231	686	1	chr2	41775514	41776249	735	single								
616	chr2	41823271	41836995	13724	17	chr2	41823240	41837156	13916	single	Variation_43493	chr2	41826625	41829441	2816	Wang et al. (2008)	Illumina DNA sequencing	
617	chr2	41859384	41863059	3675	6	chr2	41859353	41863126	3773	HiConf								
618	chr2	42200130	42200440	310	76	chr2	42200099	42200442	343	HiConf	Variation_44026	chr2	42199762	42200927	1165	Bentley et al. (2008)	Illumina DNA sequencing	
619	chr2	42916020	42918715	2695	4	chr2	42915989	42918782	2793	HiConf								
620	chr2	43754900	43759996	5096	12	chr2	43754869	43760112	5243	single	Variation_8942	chr2	43656932	43848995	192063	Pinto et al. (2007)	Affymetrix 500K SNP Mapping Array	
621	chr2	43934730	43951243	16513	18	chr2	43934699	43951555	16856	HiConf								
622	chr2	44678795	44683548	4753	9	chr2	44678764	44683615	4851	HiConf	Variation_38432	chr2	44680343	44682239	1896	McCarroll et al. (2008)	Affymetrix Human SNP Array 6.0	
623	chr2	45390961	45391745	784	46	chr2	45390930	45391763	833	HiConf	Variation_8368	chr2	45222194	45853323	631129	Pinto et al. (2007)	Affymetrix 500K SNP Mapping Array	
624	chr2	45599309	45600926	1617	3	chr2	45599278	45600944	1666	single	Variation_8368	chr2	45222194	45853323	631129	Pinto et al. (2007)	Affymetrix 500K SNP Mapping Array	
625	chr2	45897866	45902031	4165	1	chr2	45897835	45902098	4263	single								
626	chr2	46378311	46380271	1960	21	chr2	46378280	46380289	2009	single								
627	chr2	46629485	46643254	13769	2	chr2	46629454	46643517	14063	HiConf	Variation_30941	chr2	46630035	46643541	13506	Perry et al. (2008)	Agilent Custom CGH Arrays	
628	chr2	46821467	46832100	10633	1	chr2	46821436	46832314	10878	single								y
629	chr2	48704475	48711340	6865	8	chr2	48699116	48712738	13622	HiConf	Variation_39248	chr2	48704694	48711456	6762	Wheeler et al. (2008)	Sequencing	
630	chr2	49881223	49881673	450	19	chr2	49881192	49881682	490	HiConf								
100104						chr2	49882956	49954374	71418	single								
631	chr2	49988484	49988794	310	3	chr2	49988453	49988796	343	single								
632	chr2	51077813	51079028	1215	48	chr2	51077674	51079046	1372	HiConf								
633	chr2	51287131	51289091	1960	7	chr2	51287100	51289109	2009	HiConf								
634	chr2	51358230	51361121	2891	5	chr2	51358199	51361188	2989	single								
635	chr2	51827454	51827616	162	33	chr2	51827423	51827619	196	HiConf								
636	chr2	51862685	51900993	38308	24	chr2	51862654	51901756	39102	HiConf								
100106	chr2	52601728	52638429	36701	13	chr2	52601697	52639157	37460	HiConf	Variation_43685	chr2	52603158	52638854	35696	Wang et al. (2008)	Illumina DNA sequencing	
638	chr2	53102826	53105472	2646	6	chr2	53102795	53105539	2744	HiConf								
639	chr2	53412016	53414074	2058	61	chr2	53411985	53414092	2107	HiConf	Variation_5984	chr2	53411588	53413281	1693	Mills et al. (2006)	Sequence trace read mapping	
640	chr2	53531086	53532115	1029	6	chr2	53531055	53532133	1078	single								
641	chr2	53698519	53700035	1516	11	chr2	53698488	53700056	1568	HiConf	Variation_9936	chr2	53698406	53700051	1645	Wang et al. (2007)	Illumina HumanHap550 BeadChip	
642	chr2	54366879	54371142	4263	7	chr2	54366848	54371209	4361	HiConf								
643	chr2	54419139	54420926	1787	56	chr2	54419131	54420993	1862	HiConf	Variation_39251	chr2	54418997	54420977	1980	Wheeler et al. (2008)	Sequencing	
100107	chr2	54535868	54571543	35675	9	chr2	54527103	54572011	44908	single	Variation_9412	chr2	54546708	54571685	24977	Wang et al. (2007)	Illumina HumanHap550 BeadChip	
644	chr2	54807928	54807991	63	33	chr2	54807897	54807995	98	single	Variation_46020	chr2	54807815	54808048	233	Bentley et al. (2008)	Illumina DNA sequencing	
645	chr2	55177388	55189834	12446	2	chr2	55177357	55190097	12740	HiConf	Variation_9937	chr2	55178464	55188549	10085	Wang et al. (2007)	Illumina HumanHap550 BeadChip	
646	chr2	55763918	55792240	28322	17	chr2	55763887	55792797	28910	HiConf	Variation_30945	chr2	55764794	55792267	27473	Perry et al. (2008)	Agilent Custom CGH Arrays	
647	chr2	56179438	56181839	2401	3	chr2	56179407	56181906	2499	HiConf	Variation_32308	chr2	56179549	56181812	2263	Perry et al. (2008)	Agilent Custom CGH Arrays	
648	chr2	56507983	56509502	1519	33	chr2	56507952	56509520	1568	HiConf	Variation_33335	chr2	56508430	56508888	458	Perry et al. (2008)	Agilent Custom CGH Arrays	
100108	chr2	56580736	56611741	31005	3	chr2	56572755	56612518	39763	HiConf	Variation_30947	chr2	56573855	56604252	30397	Perry et al. (2008)	Agilent Custom CGH Arrays	
650	chr2	57096473	57097012	539	24	chr2	57096442	57097030	588	HiConf	Variation_3360	chr2	57016787	57341900	325113	Redon et al. (2006)	BAC Array CGH	
651	chr2	57404242	57406055	1813	3	chr2	57404211	57406073	1862	single	Variation_8372	chr2	57241283	57519133	277850	Pinto et al. (2007)	Affymetrix 500K SNP Mapping Array	
652	chr2	57435014	57435414	400	22	chr2	57434983	57435424	441	single	Variation_8372	chr2	57241283	57519133	277850	Pinto et al. (2007)	Affymetrix 500K SNP Mapping Array	
653	chr2	58058294	58059176	882	28	chr2	58058263	58059194	931	single	Variation_44032	chr2	58058133	58060268	2135	Bentley et al. (2008)	Illumina DNA sequencing	
100109	chr2	58127749	58195562	67813	1	chr2	58127525	58196958	69433	single								
655	chr2	58298345	58300648	2303	3	chr2	58298314	58300666	2352	single	Variation_9940	chr2	58244042	58304073	60031	Wang et al. (2007)	Illumina HumanHap550 BeadChip	
100110	chr2	60539273	60569761	30488	10	chr2	60524458	60570665	46207	HiConf	Variation_3361	chr2	58251801	61136244	2884443	Redon et al. (2006)	BAC Array CGH	
657	chr2	61135249	61138630	3381	7	chr2	61135218	61138697	3479	single	Variation_8373	chr2	58264453	61148977	2884524	Pinto et al. (2007)	Affymetrix 500K SNP Mapping Array	
658	chr2	61244666	61248243	3577	2	chr2	61244635	61248310	3675	single								
659	chr2	62336876	62336984	108	9	chr2	62336845	62336992	147	single								
660	chr2	62465354	62474125	8771	6	chr2	62465323	62474290	8967	HiConf								
661	chr2	63594706	63597401	2695	3	chr2	63594675	63597468	2793	HiConf								
662	chr2	63941920	63943488	1568	3	chr2	63941889	63943506	1617	HiConf								
100111	chr2	64190448	64365425	174977	4	chr2	64190417	64369022	178605	HiConf	Variation_30951	chr2	64196012	64370706	174694	Perry et al. (2008)	Agilent Custom CGH Arrays	
664	chr2	65048977	65052064	3087	9	chr2	65048946	65052131	3185	single								
665	chr2	65229346	65234883	5537	1	chr2	65229315	65234999	5684	single								

locus_id	chrom	start	end	length	Yoruba w/ event	putative chr	putative start	putative end	putative len	putative type	variation_id	DGV_chr	DGV_start	DGV_end	DGV_len	Reference	Method/platform	complex
666	chr2	65276463	65291723	15260	4	chr2	65264987	65292280	27293	single								
667	chr2	65886180	65889672	3492	3	chr2	65885082	65889933	4851	HiConf								
668	chr2	66359237	66361001	1764	9	chr2	66359206	66361019	1813	HiConf	Variation_32313	chr2	66359137	66360918	1781	Perry et al. (2008)	Agilent Custom CGH Arrays	y
669	chr2	66930038	66930398	360	7	chr2	66930007	66930399	392	single								
670	chr2	67466098	67466686	588	4	chr2	67466067	67466704	637	HiConf								
671	chr2	67612608	67619125	6517	12	chr2	67612577	67619241	6664	HiConf	Variation_38435	chr2	67614459	67619396	4937	McCarroll et al. (2008)	Affymetrix Human SNP Array 6.0	
100112	chr2	68509627	68616202	106575	3	chr2	68509596	68618376	108780	single								
673	chr2	69069378	69070938	1560	9	chr2	69069347	69071356	2009	HiConf								
674	chr2	69343876	69356469	12593	2	chr2	69343845	69356732	12887	HiConf	Variation_7279	chr2	69195924	69869677	673753	de Smith et al. (2007)	Agilent 185k CGH Arrays/Agilent Custom CGH Arrays	
675	chr2	69756995	69759543	2548	2	chr2	69756964	69759610	2646	single	Variation_7279	chr2	69195924	69869677	673753	de Smith et al. (2007)	Agilent 185k CGH Arrays/Agilent Custom CGH Arrays	
676	chr2	69978698	69978828	130	65	chr2	69978689	69978836	147	single	Variation_46033	chr2	69978559	69979067	508	Bentley et al. (2008)	Illumina DNA sequencing	
677	chr2	70842982	70845666	2684	9	chr2	70842951	70846038	3087	HiConf								
678	chr2	71173732	71174614	882	11	chr2	71173701	71174632	931	single								
679	chr2	71195586	71201221	5635	41	chr2	71195555	71201337	5782	HiConf	Variation_22568	chr2	71195896	71201301	5405	Korbel et al. (2007)	Paired End Mapping	y
680	chr2	72902942	72910243	7301	5	chr2	72902911	72910408	7497	single								
681	chr2	73124536	73133193	8657	3	chr2	73124489	73133358	8869	single								
682	chr2	73862117	73891975	29858	41	chr2	73862086	73892466	30380	HiConf	Variation_30953	chr2	73864075	73889913	25838	Perry et al. (2008)	Agilent Custom CGH Arrays	
683	chr2	75322366	75326580	4214	6	chr2	75322335	75326647	4312	HiConf								
100113	chr2	75626926	75666869	39943	10	chr2	75626895	75674474	47579	HiConf	Variation_9418	chr2	75631476	75664263	32787	Wang et al. (2007)	Illumina HumanHap550 BeadChip	
685	chr2	76190499	76191479	980	6	chr2	76190468	76191497	1029	single	Variation_4289	chr2	76129119	76304236	175117	Wong et al. (2007)	BAC Array CGH	
686	chr2	76627187	76629073	1886	78	chr2	76627156	76629459	2303	HiConf								
687	chr2	76954213	76955438	1225	4	chr2	76954182	76955456	1274	HiConf								
688	chr2	77511637	77512323	686	35	chr2	77511606	77512341	735	HiConf	Variation_9948	chr2	77509668	77516772	7104	Wang et al. (2007)	Illumina HumanHap550 BeadChip	
689	chr2	78092582	78187662	95080	5	chr2	78092550	78189619	97069	single	Variation_32320	chr2	78152015	78154109	2094	Perry et al. (2008)	Agilent Custom CGH Arrays	
690	chr2	78193129	78235563	42434	3	chr2	78193098	78236414	43316	single	Variation_8950	chr2	78104653	78288353	183700	Pinto et al. (2007)	Affymetrix 500K SNP Mapping Array	
691	chr2	78830227	78831991	1764	2	chr2	78830196	78832009	1813	single								
692	chr2	78845711	78848553	2842	15	chr2	78845680	78848620	2940	HiConf								
693	chr2	78928962	78935038	6076	19	chr2	78928931	78935154	6223	HiConf	Variation_23053	chr2	78928658	78934727	6069	Korbel et al. (2007)	Paired End Mapping	
694	chr2	79185232	79186325	1093	9	chr2	79185201	79193090	7889	HiConf	Variation_9421	chr2	79185591	79192437	6846	Wang et al. (2007)	Illumina HumanHap550 BeadChip	
100115	chr2	80372453	80412535	40082	1	chr2	80372422	80413337	40915	single	Variation_3371	chr2	80254829	80695972	441143	Redon et al. (2006)	BAC Array CGH	
695	chr2	80532879	80533443	564	25	chr2	80532848	80533485	637	HiConf	Variation_3371	chr2	80254829	80695972	441143	Redon et al. (2006)	BAC Array CGH	
696	chr2	80741521	80741881	360	8	chr2	80741490	80741882	392	single								
697	chr2	81665661	81670169	4508	4	chr2	81665630	81670236	4606	HiConf	Variation_38437	chr2	81665692	81669805	4113	McCarroll et al. (2008)	Affymetrix Human SNP Array 6.0	
698	chr2	82889307	82889446	139	30	chr2	82889307	82889454	147	single	Variation_40339	chr2	82889159	82889927	768	Wheeler et al. (2008)	Sequencing	
699	chr2	83331285	83332292	1007	1	chr2	83331238	83335893	4655	single	Variation_8379	chr2	83273184	83451071	177887	Pinto et al. (2007)	Affymetrix 500K SNP Mapping Array	
700	chr2	83955676	83958371	2695	4	chr2	83955645	83958438	2793	single								
701	chr2	84032704	84044721	12017	4	chr2	84032673	84044923	12250	single	Variation_5987	chr2	84028371	84033479	5108	Mills et al. (2006)	Sequence trace read mapping	
702	chr2	86065469	86068213	2744	7	chr2	86065438	86068280	2842	HiConf								
100116	chr2	86945509	87150623	205114	4	chr2	86945478	87154782	209304	HiConf	Variation_7283	chr2	86975626	87178841	203215	de Smith et al. (2007)	Agilent 185k CGH Arrays/Agilent Custom CGH Arrays	
703	chr2	87199231	87208639	9408	5	chr2	87199200	87208804	9604	single	Variation_30960	chr2	87199452	87428932	229480	Perry et al. (2008)	Agilent Custom CGH Arrays	
704	chr2	87413949	87501708	87759	4	chr2	87413918	87503490	89572	HiConf	Variation_35865	chr2	87434678	87441810	7132	Kidd et al. (2008)	Paired End Mapping	
705	chr2	87521896	87532333	10437	7	chr2	87521865	87532547	10682	HiConf	Variation_35867	chr2	87501600	87546743	45143	Kidd et al. (2008)	Paired End Mapping	
706	chr2	87906007	87920315	14308	18	chr2	87905976	87920578	14602	HiConf	Variation_30964	chr2	87906365	88038902	132537	Perry et al. (2008)	Agilent Custom CGH Arrays	
707	chr2	87944962	88038759	93797	29	chr2	87944931	88039795	94864	single	Variation_30964	chr2	87906365	88038902	132537	Perry et al. (2008)	Agilent Custom CGH Arrays	
708	chr2	88043256	88058856	15600	8	chr2	88043225	88059493	16268	HiConf	Variation_4294	chr2	87828344	88056043	227699	Wong et al. (2007)	BAC Array CGH	
709	chr2	88061288	88074861	13573	4	chr2	88061257	88075124	13867	single	Variation_30965	chr2	88061043	88070498	9455	Perry et al. (2008)	Agilent Custom CGH Arrays	
710	chr2	88603277	88603637	360	22	chr2	88603246	88603638	392	single	Variation_46060	chr2	88603072	88603619	547	Bentley et al. (2008)	Illumina DNA sequencing	
711	chr2	88608667	88611558	2891	6	chr2	88608636	88611625	2989	single								y
712	chr2	88913419	89088890	175471	49	chr2	88913367	89090796	177429	HiConf	Variation_4295	chr2	88921447	89083570	162123	Wong et al. (2007)	BAC Array CGH	
713	chr2	89091366	89091676	310	27	chr2	89091335	89091678	343	single	Variation_35876	chr2	89031296	89106564	75268	Kidd et al. (2008)	Paired End Mapping	
714	chr2	89091826	89106556	14730	20	chr2	89091825	89106868	15043	single	Variation_35876	chr2	89031296	89106564	75268	Kidd et al. (2008)	Paired End Mapping	
715	chr2	89220481	89268256	47775	11	chr2	89220450	89269205	48755	single	Variation_7287	chr2	89233647	89256485	22838	de Smith et al. (2007)	Agilent 185k CGH Arrays/Agilent Custom CGH Arrays	
716	chr2	89269353	89341707	72354	7	chr2	89269352	89343195	73843	HiConf	Variation_35877	chr2	89239325	89279044	39719	Kidd et al. (2008)	Paired End Mapping	
717	chr2	89385170	89410993	25823	17	chr2	89385139	89411501	26362	HiConf	Variation_12085	chr2	89388980	89389116	136	Mills et al. (2006)	Sequence trace read mapping	
718	chr2	89682747	89697496	14749	19	chr2	89682716	89697808	15092	single	Variation_2057	chr2	89561558	89717426	155868	Locke et al. (2006)	BAC Array CGH	
719	chr2	89715675	91166872	1451197	23	chr2	89715644	91193141	1477497	HiConf	Variation_2400	chr2	89682553	91638754	1956201	Redon et al. (2006)	Affymetrix 500K EA SNP Mapping Array	
720						chr2	91261447	91267866	6419	single								
721	chr2	91326207	91333361	7154	11	chr2	91326176	91333477	7301	HiConf	Variation_32329	chr2	91325040	91334084	9044	Perry et al. (2008)	Agilent Custom CGH Arrays	
722	chr2	91397992	91435281	37289	6	chr2	91397961	91436034	38073	HiConf	Variation_30974	chr2	91391901	91410114	18213	Perry et al. (2008)	Agilent Custom CGH Arrays	
723	chr2	91463085	91470043	6958	2	chr2	91444168	91493903	49735	single	Variation_32330	chr2	91431705	91538262	106557	Perry et al. (2008)	Agilent Custom CGH Arrays	
724	chr2	91599039	91624177	25138	3	chr2	91599008	91624684	25676	single	Variation_3375	chr2	90958831	91619119	660288	Redon et al. (2006)	BAC Array CGH	
725	chr2	91651322	91678223	26901	21	chr2	91651291	91678780	27489	single								
726	chr2	94890565	94898307	7742	9	chr2	94890534	94898472	7938	single	Variation_30978	chr2	94890189	94919429	29240	Perry et al. (2008)	Agilent Custom CGH Arrays	
100119						chr2	95310489	95369901	59412	single								
727	chr2	95395951	95400655	4704	2	chr2	95395920	95400722	4802	single	Variation_3377	chr2	95333865	95516286	182421	Redon et al. (2006)	BAC Array CGH	
728	chr2	95451215	95471486	20271	5	chr2	95451094	95471870	20776	HiConf	Variation_3377	chr2	95333865	95516286	182421	Redon et al. (2006)	BAC Array CGH	
729	chr2	95683728	95685884	2156	3	chr2	95683697	95685902	2205	single								
730						chr2	95797132	95804874	7742	single								
100120						chr2	95870044	96094807	224763	HiConf								
732	chr2	96154128	96160890	6762	3	chr2	96154097	96161006	6909	HiConf								
100121	chr2	97056292	97587893	531601	2	chr2	97056261	97598715	542454	HiConf	Variation_2402	chr2	97072338	97640959	568621	Redon et al. (2006)	Affymetrix 500K EA SNP Mapping Array	
736	chr2	97911268	97914477	3209	15	chr2	97911237	97914569	3332	HiConf								
737						chr2	98153101	98163538	10437	single								
100122	chr2	98221950	98247170	25220	6	chr2	98207369	98255879	48510	HiConf	Variation_9959	chr2	98221929	98238471	16542	Wang et al. (2007)	Illumina HumanHap550 BeadChip	
100123	chr2	99066738	99113740	47002	19	chr2	99066706	99114677	47971	HiConf	Variation_4301	chr2	98900265	99076431	176166	Wong et al. (2007)	BAC Array CGH	y
740	chr2	99469762	99471638	1876	45	chr2	99469731	99471642	1911	HiConf	Variation_43318	chr2	99470109	99471559	1450	Wang et al. (2008)	Illumina DNA sequencing	
741	chr2	101599057	101599743	686	23	chr2	101599026	101599761	735	HiConf								y
742	chr2	101760267	101769381	9114	34	chr2	101760236	101769399	9163	HiConf	Variation_38393	chr2	101763391	101766764	3373	McCarroll et al. (2008)	Affymetrix Human SNP Array 6.0	
743	chr2	102476794	102477054	260	13	chr2	102476763	102477057	294	single								
744	chr2	103682978	103685771	2793	7	chr2	103682947	103685838	2891	single								
745	chr2	103759614	103773138	13524	2	chr2	103759583	103773401	13818	HiConf								
100125	chr2	104804147	104834625	30478	7	chr2	104804116	104835231	31115	single								
746	chr2	105788214	105794682	6468	6	chr2	105788183	105794798	6615	single	Variation_0021	chr2	105711636	105852218	140582	Iafrate et al. (2004)	BAC Array CGH	
747	chr2	105794976	105801542	6566	6	chr2	105794945	105801658	6713	single	Variation_0021	chr2	105711636	105852218	140582	Iafrate et al. (2004)	BAC Array CGH	
748	chr2	105805315	105807079	1764	11	chr2	105805284	105807097	1813	HiConf	Variation_0021	chr2	105711636	105852218	140582	Iafrate et al. (2004)	BAC Array CGH	
749	chr2	105918848	105919387	539	19	chr2	105918817	105919405	588	HiConf								

locus_id	chrom	start	end	length	Yoruba w/ event	putative chr	putative start	putative end	putative len	putative type	variation_id	DGV_chr	DGV_start	DGV_end	DGV_len	Reference	Method/platform	complex
750	chr2	106061661	106061943	282	46	chr2	106061652	106061946	294	HiConf	Variation_12091	chr2	106061511	106062327	816	Mills et al. (2006)	Sequence trace read mapping	
751	chr2	106231811	106236466	4655	1	chr2	106231780	106236533	4753	single	Variation_0529	chr2	106217623	106264339	46716	Tuzun et al. (2005)	Paired End Mapping/Sequencing/PCR	
752	chr2	106247001	106252685	5684	39	chr2	106246970	106252801	5831	HiConf	Variation_38769	chr2	106247145	106251789	4644	McCarroll et al. (2008)	Affymetrix Human SNP Array 6.0	
100127	chr2	106422021	106463342	41321	2	chr2	106350752	106465682	114930	HiConf								
754	chr2	107265609	107270072	4463	6	chr2	107265533	107270139	4606	HiConf								
755	chr2	107383232	107385761	2529	14	chr2	107383231	107385828	2597	HiConf	Variation_22497	chr2	107383121	107385739	2618	Korbel et al. (2007)	Paired End Mapping	
756	chr2	107549764	107551234	1470	4	chr2	107549733	107551252	1519	HiConf								
757	chr2	107641786	107646931	5145	27	chr2	107641755	107647047	5292	HiConf	Variation_43993	chr2	107641745	107645537	3792	Bentley et al. (2008)	Illumina DNA sequencing	
758	chr2	107808974	107886323	77349	9	chr2	107808943	107887833	78890	HiConf	Variation_23576	chr2	107810312	107811812	1500	Levy et al. (2007)	Sequencing	
759	chr2	107903740	107903902	162	4	chr2	107903709	107903905	196	single	Variation_3382	chr2	107816309	108345086	528777	Redon et al. (2006)	BAC Array CGH	
760	chr2	107907023	107909032	2009	7	chr2	107906992	107909050	2058	single	Variation_3382	chr2	107816309	108345086	528777	Redon et al. (2006)	BAC Array CGH	
761	chr2	108221917	108222681	764	61	chr2	108221915	108222699	784	HiConf	Variation_40351	chr2	108221850	108222713	863	Wheeler et al. (2008)	Sequencing	
100129	chr2	108674915	108693473	18558	17	chr2	108628787	108753051	124264	single	Variation_23577	chr2	108676986	108678542	1556	Levy et al. (2007)	Sequencing	
762	chr2	109905831	109922331	16500	2	chr2	109905800	109960827	55027	HiConf	Variation_8954	chr2	109814503	110337635	523132	Pinto et al. (2007)	Affymetrix 500K SNP Mapping Array	
763	chr2	110044417	110089506	45089	27	chr2	110044372	110090383	46011	HiConf	Variation_32342	chr2	110045080	110088373	43293	Perry et al. (2008)	Agilent Custom CGH Arrays	
764	chr2	110324977	110334287	9310	5	chr2	110324946	110334452	9506	HiConf	Variation_48026	chr2	110212141	110330690	118549	Gusev et al. (2009)	SNP genotyping analysis	
765						chr2	110340724	110500366	159642	HiConf								
766	chr2	110588156	110634803	46647	12	chr2	110588125	110635900	47775	HiConf	Variation_32347	chr2	110590096	110633406	43310	Perry et al. (2008)	Agilent Custom CGH Arrays	
767	chr2	111008579	111050741	42162	2	chr2	110858507	111054605	196098	HiConf	Variation_23253	chr2	110208911	111170310	961399	Korbel et al. (2007)	Paired End Mapping	
100132	chr2	111725586	112080427	354841	2	chr2	111725538	112087672	362134	HiConf	Variation_30996	chr2	111754603	112090736	336133	Perry et al. (2008)	Agilent Custom CGH Arrays	
100133	chr2	112192147	112363913	171766	2	chr2	112192116	112367413	175297	HiConf	Variation_30997	chr2	112194592	112320073	125481	Perry et al. (2008)	Agilent Custom CGH Arrays	
771	chr2	112754544	112758562	4018	13	chr2	112754513	112758629	4116	single								
100134	chr2	112895376	112910030	14654	1	chr2	112818311	112922436	104125	HiConf								
773	chr2	113194907	113202012	7105	25	chr2	113194876	113202128	7252	HiConf	Variation_2405	chr2	113185706	113195360	9654	Redon et al. (2006)	Affymetrix 500K EA SNP Mapping Array	
100135	chr2	113883428	114082214	198786	6	chr2	113883302	114086162	202860	HiConf	Variation_30998	chr2	113888688	113970862	82174	Perry et al. (2008)	Agilent Custom CGH Arrays	
776	chr2	114243409	114247186	3777	2	chr2	114243378	114247543	4165	HiConf	Variation_35709	chr2	114224359	114251669	27310	Kidd et al. (2008)	Paired End Mapping	
777	chr2	114558528	114559018	490	30	chr2	114558497	114559036	539	HiConf								
778	chr2	115105711	115118941	13230	18	chr2	115105680	115119204	13524	HiConf	Variation_1034	chr2	115108946	115118854	9908	Conrad et al. (2005)	Mendelian inconsistencies	
779	chr2	115168208	115171371	3163	14	chr2	115168155	115171438	3283	HiConf								y
780	chr2	115344635	115345125	490	67	chr2	115344604	115345143	539	HiConf	Variation_45841	chr2	115344580	115345314	734	Bentley et al. (2008)	Illumina DNA sequencing	
781	chr2	116378682	116379042	360	20	chr2	116378651	116379043	392	single	Variation_10713	chr2	116378779	116379089	310	Conrad et al. (2005)	Mendelian inconsistencies	
782	chr2	117422676	117424685	2009	5	chr2	117422645	117424703	2058	single	Variation_3388	chr2	117261003	117467903	206900	Redon et al. (2006)	BAC Array CGH	
783	chr2	117444971	117452419	7448	7	chr2	117444940	117452584	7644	HiConf	Variation_32351	chr2	117445424	117452682	7258	Perry et al. (2008)	Agilent Custom CGH Arrays	
784	chr2	117485886	117499949	14063	11	chr2	117485855	117500212	14357	HiConf	Variation_37711	chr2	117489208	117508223	19015	McCarroll et al. (2008)	Affymetrix Human SNP Array 6.0	
785	chr2	118597941	118598676	735	5	chr2	118597910	118598694	784	HiConf								
786	chr2	119150024	119152719	2695	2	chr2	119149993	119152786	2793	single								
787	chr2	120674309	120684508	10199	1	chr2	120673844	120684722	10878	single	Variation_4304	chr2	120606832	120778568	171736	Wong et al. (2007)	BAC Array CGH	
788	chr2	120688918	120691270	2352	22	chr2	120688887	120691288	2401	single	Variation_4304	chr2	120606832	120778568	171736	Wong et al. (2007)	BAC Array CGH	
789	chr2	123000983	123010636	9653	2	chr2	123000952	123010850	9898	HiConf								
790	chr2	123193968	123198845	4877	14	chr2	123193963	123198961	4998	HiConf	Variation_32352	chr2	123193361	123198997	5636	Perry et al. (2008)	Agilent Custom CGH Arrays	
791	chr2	124144888	124156746	11858	9	chr2	124144857	124156960	12103	HiConf								
792	chr2	125368369	125375327	6958	6	chr2	125368338	125375443	7105	HiConf	Variation_37857	chr2	125368402	125374828	6426	McCarroll et al. (2008)	Affymetrix Human SNP Array 6.0	
793	chr2	125824902	125827352	2450	3	chr2	125824871	125827419	2548	HiConf								
794	chr2	126159313	126168294	8981	89	chr2	126159247	126168459	9212	HiConf	Variation_5997	chr2	126159718	126168301	8583	Mills et al. (2006)	Sequence trace read mapping	
795	chr2	126195636	126198674	3038	3	chr2	126195605	126198741	3136	HiConf								
796	chr2	127113672	127113945	273	1	chr2	127112003	127113963	1960	single								y
797	chr2	127391187	127395098	3911	42	chr2	127391156	127395125	3969	HiConf	Variation_43483	chr2	127391183	127393897	2714	Wang et al. (2008)	Illumina DNA sequencing	
798	chr2	127438325	127438585	260	44	chr2	127438294	127438588	294	single								
799	chr2	127820286	127829296	9010	8	chr2	127820249	127829461	9212	HiConf								
800	chr2	127937586	127938811	1225	9	chr2	127937555	127938829	1274	single								
801	chr2	127940183	127942535	2352	11	chr2	127940152	127942553	2401	HiConf								
802	chr2	128269512	128271080	1568	4	chr2	128269481	128271098	1617	HiConf								
803	chr2	128541413	128543275	1862	4	chr2	128541382	128543293	1911	single	Variation_38400	chr2	128538342	128543406	5064	McCarroll et al. (2008)	Affymetrix Human SNP Array 6.0	
804	chr2	129728977	129730202	1225	34	chr2	129728946	129730220	1274	HiConf	Variation_3392	chr2	129582094	129866515	284421	Redon et al. (2006)	BAC Array CGH	
805	chr2	129959816	129965794	5978	8	chr2	129959785	129965910	6125	HiConf	Variation_32357	chr2	129959100	129966390	7290	Perry et al. (2008)	Agilent Custom CGH Arrays	
806	chr2	130072467	130072629	162	27	chr2	130072436	130072632	196	HiConf	Variation_45855	chr2	130072317	130072691	374	Bentley et al. (2008)	Illumina DNA sequencing	
807	chr2	130154150	130160156	6006	18	chr2	130154119	130161861	7742	HiConf	Variation_22794	chr2	130153649	130162049	8400	Korbel et al. (2007)	Paired End Mapping	
808	chr2	130261411	130274739	13328	17	chr2	130261380	130275002	13622	HiConf	Variation_32358	chr2	130265994	130274110	8116	Perry et al. (2008)	Agilent Custom CGH Arrays	y
809	chr2	130406010	130409685	3675	14	chr2	130405979	130409752	3773	HiConf	Variation_32360	chr2	130405412	130484564	79152	Perry et al. (2008)	Agilent Custom CGH Arrays	
810	chr2	130426622	130489898	63276	5	chr2	130426265	130491190	64925	HiConf	Variation_32360	chr2	130405412	130484564	79152	Perry et al. (2008)	Agilent Custom CGH Arrays	
811	chr2	130496513	130497003	490	17	chr2	130496482	130497021	539	single	Variation_32361	chr2	130495524	130502748	7224	Perry et al. (2008)	Agilent Custom CGH Arrays	
812	chr2	130497322	130506019	8697	7	chr2	130497315	130506184	8869	single	Variation_32361	chr2	130495524	130502748	7224	Perry et al. (2008)	Agilent Custom CGH Arrays	
813	chr2	130516603	130524443	7840	2	chr2	130516572	130524608	8036	single	Variation_37826	chr2	130510353	130539705	29352	McCarroll et al. (2008)	Affymetrix Human SNP Array 6.0	
814	chr2	130548110	130587479	39369	3	chr2	130548079	130588112	40033	HiConf	Variation_32364	chr2	130549454	130562946	13492	Perry et al. (2008)	Agilent Custom CGH Arrays	
815	chr2	130885396	130895912	10516	5	chr2	130885395	130896126	10731	single	Variation_32367	chr2	130884837	130897955	13118	Perry et al. (2008)	Agilent Custom CGH Arrays	
816	chr2	130915169	130917815	2646	11	chr2	130915138	130917882	2744	single	Variation_31008	chr2	130917352	130919186	1834	Perry et al. (2008)	Agilent Custom CGH Arrays	
817	chr2	130921686	130930225	8539	9	chr2	130921655	130930328	8673	single	Variation_40362	chr2	130925277	130925686	409	Wheeler et al. (2008)	Sequencing	
818	chr2	130930898	130979457	48559	2	chr2	130930867	130980455	49588	HiConf	Variation_32368	chr2	130917352	130982483	65131	Perry et al. (2008)	Agilent Custom CGH Arrays	
819						chr2	131092077	131131522	39445	HiConf								
821	chr2	131329807	131337837	8030	37	chr2	131329776	131337910	8134	HiConf	Variation_23583	chr2	131334474	131337950	3476	Levy et al. (2007)	Sequencing	
822	chr2	131355581	131363274	7693	4	chr2	131355550	131363439	7889	HiConf	Variation_32373	chr2	131355716	131361706	5990	Perry et al. (2008)	Agilent Custom CGH Arrays	
823	chr2	131633411	131675474	42063	8	chr2	131633380	131676255	42875	HiConf	Variation_32374	chr2	131650772	131840170	189398	Perry et al. (2008)	Agilent Custom CGH Arrays	
824						chr2	131697717	131739465	41748	single								
825	chr2	131754448	131755361	913	1	chr2	131746227	131755537	9310	single	Variation_32374	chr2	131650772	131840170	189398	Perry et al. (2008)	Agilent Custom CGH Arrays	
826	chr2	131831224	131836418	5194	11	chr2	131831193	131836534	5341	single	Variation_9431	chr2	131793849	131951456	157607	Wang et al. (2007)	Illumina HumanHap550 BeadChip	
827	chr2	131879293	131904136	24843	2	chr2	131879262	131904644	25382	single	Variation_1597	chr2	131903442	131943533	40091	McCarroll et al. (2005)	Null genotypes	
828	chr2	131938044	131941033	2989	34	chr2	131938013	131941100	3087	HiConf	Variation_38115	chr2	131938478	131942838	4360	McCarroll et al. (2008)	Affymetrix Human SNP Array 6.0	y
100141	chr2	132127490	132743328	615838	36	chr2	132024474	132832998	808524	HiConf	Variation_7231	chr2	132489782	132765542	275760	de Smith et al. (2007)	Agilent 185k CGH Arrays/Agilent Custom CGH Arrays	
833	chr2	132855961	132856843	882	18	chr2	132855930	132856861	931	single	Variation_3393	chr2	130063308	132914473	2851165	Redon et al. (2006)	BAC Array CGH	y
834	chr2	132857970	132867182	9212	14	chr2	132857939	132867347	9408	HiConf	Variation_35730	chr2	132864232	132893330	29098	Kidd et al. (2008)	Paired End Mapping	y
835	chr2	132867476	132874141	6665	37	chr2	132867445	132874256	6811	HiConf	Variation_35730	chr2	132864232	132893330	29098	Kidd et al. (2008)	Paired End Mapping	
836	chr2	132997179	133024988	27809	16	chr2	132997148	133025519	28371	HiConf	Variation_37542	chr2	132997360	133025106	27746	Cooper et al. (2008)	Illumina Human 1M BeadChip	
837						chr2	133661637	133666341	4704	single								
838	chr2	134140006	134141770	1764	8	chr2	134139975	134141788	1813	HiConf								
839	chr2	134621333	134625106	3773	3	chr2	134621302	134625173	3871	single								
840	chr2	135267104	135267311	207	10	chr2	135267073	135267318	245	HiConf								

locus_id	chrom	start	end	length	Yoruba w/ event	putative chr	putative start	putative end	putative len	putative type	variation_id	DGV_chr	DGV_start	DGV_end	DGV_len	Reference	Method/platform	complex
841	chr2	135537386	135539103	1717	6	chr2	135537112	135539121	2009	single								
842	chr2	135542190	135559830	17640	6	chr2	135542159	135560191	18032	HiConf								
843	chr2	135688651	135692718	4067	3	chr2	135688620	135692785	4165	HiConf								
844	chr2	135816492	135817864	1372	53	chr2	135816461	135817882	1421	HiConf	Variation_37413	chr2	135814811	135818043	3232	Cooper et al. (2008)	Illumina Human 1M BeadChip	
845	chr2	136510773	136516898	6125	3	chr2	136510742	136517014	6272	HiConf								
846	chr2	137957498	137971018	13520	32	chr2	137957467	137971187	13720	HiConf								
847	chr2	139692147	139692507	360	8	chr2	139692116	139692508	392	HiConf								
848	chr2	139832917	139833757	840	5	chr2	139832550	139833775	1225	single								
849	chr2	140276227	140280490	4263	2	chr2	140276196	140280557	4361	single								
850	chr2	140737317	140745206	7889	3	chr2	140737286	140745371	8085	HiConf								
851	chr2	141900626	141955751	55125	3	chr2	141900595	141956847	56252	HiConf	Variation_8390	chr2	141897738	141955738	58000	Pinto et al. (2007)	Affymetrix 500K SNP Mapping Array	
852	chr2	142112649	142112856	207	77	chr2	142112618	142112863	245	single	Variation_45869	chr2	142112365	142113081	716	Bentley et al. (2008)	Illumina DNA sequencing	
853	chr2	142364509	142369801	5292	7	chr2	142364478	142369917	5439	single								
854	chr2	143278849	143312254	33405	4	chr2	143278818	143312922	34104	single								
855	chr2	143331769	143332406	637	16	chr2	143331738	143332424	686	HiConf								
100143						chr2	143568310	143594795	26485	single								
856	chr2	145197444	145201756	4312	7	chr2	145197413	145201823	4410	HiConf								
857	chr2	146579120	146594060	14940	19	chr2	146579115	146594207	15092	HiConf	Variation_43673	chr2	146579036	146593475	14439	Wang et al. (2008)	Illumina DNA sequencing	
858	chr2	147268821	147270977	2156	1	chr2	147268790	147270995	2205	single	Variation_4309	chr2	147220848	147399344	178496	Wong et al. (2007)	BAC Array CGH	
859	chr2	147662683	147663222	539	52	chr2	147662652	147663240	588	HiConf	Variation_23173	chr2	147656248	147665206	8958	Korbel et al. (2007)	Paired End Mapping	
100145	chr2	148385605	148418337	32732	4	chr2	148385574	148418992	33418	single								
861	chr2	148916397	148918994	2597	21	chr2	148916366	148919061	2695	HiConf	Variation_8391	chr2	148867828	149813849	946021	Pinto et al. (2007)	Affymetrix 500K SNP Mapping Array	
862	chr2	148965201	148967063	1862	3	chr2	148965170	148967081	1911	HiConf	Variation_8391	chr2	148867828	149813849	946021	Pinto et al. (2007)	Affymetrix 500K SNP Mapping Array	
100146	chr2	149120392	149150992	30600	2	chr2	149110161	149161195	51034	single	Variation_8391	chr2	148867828	149813849	946021	Pinto et al. (2007)	Affymetrix 500K SNP Mapping Array	
864	chr2	149567167	149567274	107	7	chr2	149567135	149567282	147	single	Variation_8391	chr2	148867828	149813849	946021	Pinto et al. (2007)	Affymetrix 500K SNP Mapping Array	y
100147	chr2	151428854	153413162	1984308	13	chr2	151428768	153423558	1994790	HiConf	Variation_8961	chr2	151423126	153424992	2001866	Pinto et al. (2007)	Affymetrix 500K SNP Mapping Array	
867	chr2	153503924	153507849	3925	9	chr2	153503893	153507911	4018	HiConf	Variation_39269	chr2	153504241	153507989	3748	Wheeler et al. (2008)	Sequencing	
868						chr2	154116540	154122420	5880	single								
869	chr2	155182174	155191043	8869	2	chr2	155182143	155191208	9065	HiConf	Variation_9965	chr2	155182601	155191183	8582	Wang et al. (2007)	Illumina HumanHap550 BeadChip	
100148	chr2	157002697	157024917	22220	1	chr2	156807130	157029345	222215	HiConf								
870	chr2	158693710	158694298	588	17	chr2	158693679	158694316	637	single								
871	chr2	158989964	158993198	3234	3	chr2	158989933	158993265	3332	single								
872	chr2	159370351	159370939	588	12	chr2	159370320	159370957	637	HiConf	Variation_12102	chr2	159370243	159371120	877	Mills et al. (2006)	Sequence trace read mapping	
873	chr2	159667383	159670193	2810	51	chr2	159667015	159670200	3185	HiConf	Variation_23586	chr2	159667423	159669692	2269	Levy et al. (2007)	Sequencing	
874	chr2	159806647	159808068	1421	42	chr2	159806616	159808086	1470	HiConf	Variation_11160	chr2	159806675	159807502	827	de Smith et al. (2007)	Agilent 185k CGH Arrays/Agilent Custom CGH Arrays	
875						chr2	160355465	160368156	12691	single								y
876	chr2	161843773	161843980	207	60	chr2	161843742	161843987	245	single								y
877	chr2	162041733	162042043	310	44	chr2	162041702	162042045	343	HiConf	Variation_23588	chr2	162040517	162042291	1774	Levy et al. (2007)	Sequencing	
878	chr2	162406832	162408645	1813	1	chr2	162406801	162408663	1862	single								
879	chr2	164146626	164151526	4900	2	chr2	164146595	164151642	5047	HiConf								
880	chr2	164294655	164307346	12691	2	chr2	164294624	164307609	12985	single	Variation_4315	chr2	164211895	164391872	179977	Wong et al. (2007)	BAC Array CGH	
881	chr2	164349584	164353847	4263	6	chr2	164349553	164353914	4361	HiConf	Variation_38409	chr2	164351734	164353370	1636	McCarroll et al. (2008)	Affymetrix Human SNP Array 6.0	y
882	chr2	164852079	164866338	14259	9	chr2	164852048	164866601	14553	single								
100149	chr2	165535556	165563339	27783	10	chr2	165535525	165563896	28371	HiConf	Variation_7240	chr2	165552585	165573244	20659	de Smith et al. (2007)	Agilent 185k CGH Arrays/Agilent Custom CGH Arrays	
884	chr2	166353831	166354958	1127	2	chr2	166353800	166354976	1176	single								
885	chr2	166857355	166866959	9604	21	chr2	166857324	166867124	9800	HiConf								
886	chr2	167652968	167657770	4802	1	chr2	167652937	167657837	4900	single								
887	chr2	169820287	169825172	4885	6	chr2	169820256	169825303	5047	single	Variation_35758	chr2	169812078	169836110	24032	Kidd et al. (2008)	Paired End Mapping	
100150	chr2	170350323	170434461	84138	6	chr2	170333458	170436137	102679	single	Variation_23590	chr2	170349764	170352208	2444	Levy et al. (2007)	Sequencing	
888	chr2	171553662	171561747	8085	4	chr2	171553631	171561912	8281	single								
889	chr2	172541110	172549685	8575	18	chr2	172541079	172549850	8771	HiConf	Variation_22832	chr2	172539681	172549763	10082	Korbel et al. (2007)	Paired End Mapping	
890	chr2	172617942	172618149	207	16	chr2	172617911	172618156	245	single								
891	chr2	172714913	172715363	450	40	chr2	172714882	172715372	490	single	Variation_23100	chr2	172710102	172716776	6674	Korbel et al. (2007)	Paired End Mapping	
892	chr2	173437516	173438300	784	6	chr2	173437485	173438318	833	single								
893						chr2	173874075	173893332	19257	HiConf								
894	chr2	174249513	174257460	7947	2	chr2	174249513	174257598	8085	HiConf	Variation_38410	chr2	174249122	174254919	5797	McCarroll et al. (2008)	Affymetrix Human SNP Array 6.0	
895	chr2	174427610	174430109	2499	2	chr2	174427579	174430176	2597	HiConf	Variation_44006	chr2	174427462	174430338	2876	Bentley et al. (2008)	Illumina DNA sequencing	
100151	chr2	176870432	176912180	41748	2	chr2	176870401	176913006	42605	single								y
896	chr2	176973846	176980573	6727	47	chr2	176973815	176980577	6762	HiConf	Variation_23593	chr2	176973891	176980290	6399	Levy et al. (2007)	Sequencing	
897	chr2	177507701	177514120	6419	9	chr2	177507670	177514236	6566	HiConf								
898	chr2	177995907	177996297	390	47	chr2	177995906	177996298	392	HiConf	Variation_45905	chr2	177995881	177996308	427	Bentley et al. (2008)	Illumina DNA sequencing	
899	chr2	178449383	178449783	400	60	chr2	178449352	178449793	441	HiConf								
900	chr2	178689614	178689875	261	1	chr2	178688423	178689893	1470	single								
100152	chr2	178863335	178890160	26825	4	chr2	178863304	178899564	36260	single								
902						chr2	179225120	179237076	11956	HiConf								
903	chr2	179775152	179782477	7325	43	chr2	179768089	179782740	14651	HiConf	Variation_38074	chr2	179775437	179787389	11952	McCarroll et al. (2008)	Affymetrix Human SNP Array 6.0	
904	chr2	179935259	179935519	260	25	chr2	179935228	179935522	294	HiConf								
905	chr2	180118328	180130867	12539	35	chr2	180118243	180131130	12887	HiConf	Variation_32384	chr2	180121299	180131307	10008	Perry et al. (2008)	Agilent Custom CGH Arrays	
906	chr2	181405896	181406206	310	26	chr2	181405865	181406208	343	single								
100153	chr2	181564338	181661582	97244	48	chr2	181564307	181670049	105742	HiConf								
908	chr2	182151662	182153342	1680	8	chr2	182151596	182153360	1764	HiConf								
909	chr2	182441952	182443814	1862	3	chr2	182441921	182443832	1911	HiConf								
910	chr2	182565187	182565775	588	59	chr2	182565156	182565793	637	HiConf	Variation_45912	chr2	182565164	182565841	677	Bentley et al. (2008)	Illumina DNA sequencing	
911	chr2	182798770	182799750	980	67	chr2	182798739	182799768	1029	HiConf	Variation_44010	chr2	182798628	182799875	1247	Bentley et al. (2008)	Illumina DNA sequencing	
912	chr2	183138572	183140398	1826	3	chr2	183132233	183140563	8330	HiConf								
100155	chr2	183236002	183548960	312958	1	chr2	183235917	183555324	319407	single	Variation_8394	chr2	183245644	183495008	249364	Pinto et al. (2007)	Affymetrix 500K SNP Mapping Array	
913	chr2	183698018	183698328	310	10	chr2	183697987	183698330	343	HiConf								
914	chr2	184289987	184291506	1519	3	chr2	184289956	184291524	1568	single								
915	chr2	184309881	184313164	3283	12	chr2	184309850	184313231	3381	HiConf	Variation_32387	chr2	184310574	184312932	2358	Perry et al. (2008)	Agilent Custom CGH Arrays	
916	chr2	184557086	184558605	1519	3	chr2	184557055	184558623	1568	HiConf								
917	chr2	184629214	184632718	3504	9	chr2	184629183	184640159	10976	HiConf	Variation_22852	chr2	184629181	184641045	11864	Korbel et al. (2007)	Paired End Mapping	
918	chr2	184807819	184846872	39053	2	chr2	184807788	184847674	39886	HiConf	Variation_30105	chr2	184807930	184842482	34552	Jakobsson et al. (2008)	Illumina HumanHap550 BeadChip	
919	chr2	184954966	184972557	17591	21	chr2	184954935	184972918	17983	HiConf	Variation_22885	chr2	184954893	184973428	18535	Korbel et al. (2007)	Paired End Mapping	
921	chr2	186449172	186455248	6076	1	chr2	186449141	186455364	6223	single								y
922	chr2	187382769	187384631	1862	9	chr2	187382738	187384649	1911	HiConf								
923	chr2	187804953	187813675	8722	1	chr2	187804922	187813840	8918	single	Variation_8963	chr2	187744192	187849935	105743	Pinto et al. (2007)	Affymetrix 500K SNP Mapping Array	y

locus_id	chrom	start	end	length	Yoruba w/ event	putative chr	putative start	putative end	putative len	putative type	variation_id	DGV_chr	DGV_start	DGV_end	DGV_len	Reference	Method/platform	complex
100158	chr2	188059459	188154600	95141	22	chr2	188059428	188155101	95673	HiConf	Variation_3404	chr2	188088287	188211430	123143	Redon et al. (2006)	BAC Array CGH	
925	chr2	188827583	188829396	1813	49	chr2	188827552	188829414	1862	HiConf								y
926	chr2	188975612	188983795	8183	2	chr2	188975581	188983960	8379	single								
927	chr2	189276129	189287889	11760	15	chr2	189276098	189288348	12250	HiConf	Variation_37938	chr2	189276201	189287574	11373	McCarroll et al. (2008)	Affymetrix Human SNP Array 6.0	
928	chr2	191703638	191707117	3479	6	chr2	191703607	191707184	3577	HiConf								
929	chr2	193003804	193010370	6566	2	chr2	193003773	193010486	6713	HiConf								
930	chr2	193130665	193133801	3136	6	chr2	193130634	193133868	3234	single								
931	chr2	194397805	194410055	12250	52	chr2	194397774	194410318	12544	HiConf	Variation_22842	chr2	194397088	194407968	10880	Korbel et al. (2007)	Paired End Mapping	
932	chr2	194519619	194521775	2156	6	chr2	194519588	194521793	2205	HiConf	Variation_29705	chr2	194452842	194613887	161045	Jakobsson et al. (2008)	Illumina HumanHap550 BeadChip	
100159	chr2	194623058	194665590	42532	2	chr2	194623027	194666466	43439	HiConf	Variation_2420	chr2	194628082	194665120	37038	Redon et al. (2006)	Affymetrix 500K EA SNP Mapping Array	y
934	chr2	195005846	195006296	450	24	chr2	195005815	195006305	490	single	Variation_30109	chr2	194884458	195024254	139796	Jakobsson et al. (2008)	Illumina HumanHap550 BeadChip	
935	chr2	195022751	195037956	15205	34	chr2	195022720	195037959	15239	HiConf	Variation_38416	chr2	195022965	195034089	11124	McCarroll et al. (2008)	Affymetrix Human SNP Array 6.0	
936	chr2	195397552	195399757	2205	1	chr2	195397521	195399775	2254	single								
937	chr2	195686848	195692385	5537	58	chr2	195686817	195692501	5684	HiConf	Variation_22567	chr2	195687498	195692885	5387	Korbel et al. (2007)	Paired End Mapping	
938	chr2	195871627	195872460	833	18	chr2	195871596	195872478	882	HiConf								
100160	chr2	197458051	197510165	52114	3	chr2	197458020	197511112	53092	single								
940	chr2	197850296	197851178	882	12	chr2	197850265	197851196	931	HiConf								
941	chr2	198876797	198878169	1372	4	chr2	198876766	198878187	1421	single								
942	chr2	199779818	199781081	1263	10	chr2	199779787	199781110	1323	HiConf								
943	chr2	199887373	199891538	4165	5	chr2	199887342	199891605	4263	HiConf	Variation_38417	chr2	199887215	199890802	3587	McCarroll et al. (2008)	Affymetrix Human SNP Array 6.0	
944	chr2	200028640	200029040	400	28	chr2	200028609	200029050	441	single								
100161	chr2	201078131	201167915	89784	1	chr2	201045678	201170407	124729	single								
945	chr2	201336352	201338606	2254	6	chr2	201336321	201338624	2303	single								
946	chr2	201849676	201858014	8338	39	chr2	201849645	201858024	8379	single	Variation_23598	chr2	201854903	201857695	2792	Levy et al. (2007)	Sequencing	
100162	chr2	202146592	202224982	78390	3	chr2	202146561	202226431	79870	single								
947	chr2	203002107	203020680	18573	37	chr2	203002076	203021039	18963	HiConf	Variation_37929	chr2	203004020	203020446	16426	McCarroll et al. (2008)	Affymetrix Human SNP Array 6.0	
948	chr2	203607845	203611667	3822	3	chr2	203607814	203611734	3920	HiConf	Variation_37952	chr2	203608045	203610291	2246	McCarroll et al. (2008)	Affymetrix Human SNP Array 6.0	
949	chr2	204012144	204015182	3038	43	chr2	204012113	204015249	3136	HiConf								
950	chr2	204223873	204225784	1911	3	chr2	204223842	204225802	1960	single								
951	chr2	205051111	205057118	6007	6	chr2	205051109	205057234	6125	HiConf	Variation_2421	chr2	205050972	205056969	5997	Redon et al. (2006)	Affymetrix 500K EA SNP Mapping Array	
952	chr2	205226808	205227393	585	8	chr2	205226431	205227411	980	HiConf								
953	chr2	205414377	205420161	5784	2	chr2	205414346	205420275	5929	HiConf	Variation_3405	chr2	205248077	205549419	301342	Redon et al. (2006)	BAC Array CGH	
954	chr2	205836316	205838080	1764	6	chr2	205836285	205838098	1813	HiConf								
955	chr2	207852617	207855459	2842	10	chr2	207852586	207855526	2940	HiConf								
956	chr2	208060426	208067482	7056	2	chr2	208060395	208067598	7203	HiConf	Variation_37416	chr2	208059459	208067309	7850	Cooper et al. (2008)	Illumina Human 1M BeadChip	
100163	chr2	208499127	208529013	29886	1	chr2	208499043	208529595	30552	single								
957	chr2	208942230	208954970	12740	3	chr2	208942199	208955233	13034	HiConf	Variation_32393	chr2	208945724	208954661	8937	Perry et al. (2008)	Agilent Custom CGH Arrays	
958	chr2	209638275	209650623	12348	10	chr2	209638244	209650886	12642	HiConf	Variation_35786	chr2	209623563	209666321	42758	Kidd et al. (2008)	Paired End Mapping	
959	chr2	209657483	209663853	6370	30	chr2	209657452	209663969	6517	HiConf	Variation_35786	chr2	209623563	209666321	42758	Kidd et al. (2008)	Paired End Mapping	
100164	chr2	209956674	210006234	49560	2	chr2	209941775	210024413	82638	HiConf								
960	chr2	211478819	211488564	9745	5	chr2	211478782	211488778	9996	HiConf	Variation_38418	chr2	211480395	211486620	6225	McCarroll et al. (2008)	Affymetrix Human SNP Array 6.0	
961	chr2	211787202	211788738	1536	4	chr2	211787139	211788756	1617	single								
962	chr2	212350327	212351601	1274	5	chr2	212350296	212351619	1323	single	Variation_47898	chr2	212289730	212565367	275637	Gusev et al. (2009)	SNP genotyping analysis	
963	chr2	213109435	213110317	882	11	chr2	213109404	213110335	931	single								
964	chr2	213174654	213183425	8771	2	chr2	213174623	213183590	8967	single								
100166	chr2	213678033	213742682	64649	6	chr2	213677951	213743954	66003	HiConf								
965	chr2	213852568	213867808	15240	3	chr2	213849108	213868169	19061	single								
966	chr2	213922100	213938801	16701	5	chr2	213917218	213939317	22099	HiConf	Variation_4325	chr2	213899523	214074098	174575	Wong et al. (2007)	BAC Array CGH	
967	chr2	214058614	214062485	3871	3	chr2	214058583	214062552	3969	HiConf	Variation_4325	chr2	213899523	214074098	174575	Wong et al. (2007)	BAC Array CGH	
968	chr2	214583453	214584188	735	74	chr2	214583422	214584206	784	HiConf	Variation_44013	chr2	214582952	214584454	1502	Bentley et al. (2008)	Illumina DNA sequencing	
969	chr2	215434011	215440414	6403	33	chr2	215433964	215440530	6566	HiConf	Variation_43421	chr2	215436976	215439129	2153	Wang et al. (2008)	Illumina DNA sequencing	
100167	chr2	216032603	216118713	86110	1	chr2	216024880	216120626	95746	single	Variation_8966	chr2	216026482	216118739	92257	Pinto et al. (2007)	Affymetrix 500K SNP Mapping Array	
971	chr2	216628517	216631506	2989	4	chr2	216628486	216631573	3087	HiConf								
972	chr2	217664181	217669130	4949	16	chr2	217664150	217669246	5096	HiConf								
973	chr2	218153201	218163344	10143	6	chr2	218153170	218163558	10388	single								
974	chr2	218562939	218563576	637	12	chr2	218562908	218563594	686	HiConf								
975	chr2	218734145	218735321	1176	24	chr2	218734114	218735339	1225	HiConf								y
976	chr2	219762067	219762557	490	42	chr2	219762036	219762575	539	HiConf	Variation_7248	chr2	219760483	219762359	1876	de Smith et al. (2007)	Agilent 185k CGH Arrays/Agilent Custom CGH Arrays	
100168						chr2	219994223	220157074	162851	HiConf								
977	chr2	221102658	221104871	2213	10	chr2	221102627	221104881	2254	HiConf	Variation_47935	chr2	220986970	221224908	237938	Gusev et al. (2009)	SNP genotyping analysis	
978	chr2	221406409	221408614	2205	1	chr2	221406378	221408632	2254	single								
100169	chr2	222076950	222110221	33271	2	chr2	222076919	222110876	33957	HiConf	Variation_9978	chr2	222082667	222108467	25800	Wang et al. (2007)	Illumina HumanHap550 BeadChip	
980	chr2	222207412	222207951	539	7	chr2	222207381	222207969	588	single								
981	chr2	222269054	222290663	21609	6	chr2	222269023	222291073	22050	single								
982	chr2	223121752	223140568	18816	5	chr2	223121721	223140929	19208	HiConf								
983	chr2	223573385	223577755	4370	22	chr2	223573354	223578107	4753	HiConf	Variation_37417	chr2	223573414	223580048	6634	Cooper et al. (2008)	Illumina Human 1M BeadChip	
984	chr2	224390705	224391015	310	7	chr2	224390674	224391017	343	single								
985	chr2	224875413	224885017	9604	3	chr2	224875382	224885231	9849	single								
986	chr2	224928529	224928637	108	16	chr2	224928498	224928645	147	single								
987	chr2	225001000	225002421	1421	41	chr2	225000969	225002439	1470	HiConf	Variation_45951	chr2	225001196	225002194	998	Bentley et al. (2008)	Illumina DNA sequencing	
988	chr2	226162104	226171071	8967	2	chr2	226162073	226171236	9163	single								
989	chr2	226663864	226670969	7105	10	chr2	226663833	226671085	7252	HiConf	Variation_44017	chr2	226663290	226670357	7067	Bentley et al. (2008)	Illumina DNA sequencing	
990	chr2	226871183	226879284	8101	15	chr2	226871152	226879678	8526	HiConf	Variation_43601	chr2	226873374	226879523	6149	Wang et al. (2008)	Illumina DNA sequencing	y
991	chr2	226937480	226938117	637	18	chr2	226937449	226938135	686	single								
992	chr2	227155824	227156134	310	5	chr2	227155793	227156136	343	single								
993	chr2	227369807	227371032	1225	32	chr2	227369776	227371050	1274	single								
994	chr2	227619413	227620246	833	10	chr2	227619382	227620264	882	HiConf								
995	chr2	227977652	227978102	450	21	chr2	227977621	227978111	490	HiConf								
996	chr2	228362645	228366173	3528	2	chr2	228362614	228366240	3626	single								
997	chr2	228972499	228976419	3920	24	chr2	228972468	228976486	4018	HiConf								
998	chr2	229011895	229015826	3931	3	chr2	229011864	229015882	4018	single								y
999	chr2	229156592	229160120	3528	73	chr2	229156561	229160187	3626	HiConf								
1000	chr2	229468379	229471662	3283	1	chr2	229468348	229471729	3381	single	Variation_7251	chr2	229467600	229472217	4617	de Smith et al. (2007)	Agilent 185k CGH Arrays/Agilent Custom CGH Arrays	
1001	chr2	230128507	230141835	13328	7	chr2	230128476	230142098	13622	single								
1002	chr2	230483757	230490519	6762	3	chr2	230483726	230490635	6909	single	Variation_8396	chr2	230283738	230537838	254100	Pinto et al. (2007)	Affymetrix 500K SNP Mapping Array	
1003						chr2	231133074	231146843	13769	single								

locus_id	chrom	start	end	length	Yoruba w/ event	putative chr	putative start	putative end	putative len	putative type	variation_id	DGV_chr	DGV_start	DGV_end	DGV_len	Reference	Method/platform	complex
1004	chr2	231250214	231271407	21193	5	chr2	231250135	231271695	21560	HiConf								
1005	chr2	231272853	231274029	1176	13	chr2	231272822	231274047	1225	HiConf								
1006	chr2	232438906	232441370	2464	16	chr2	232438875	232441717	2842	single	Variation_3408	chr2	232245069	232714102	469033	Redon et al. (2006)	BAC Array CGH	
1007	chr2	232728349	232748341	19992	1	chr2	232728318	232748751	20433	single								
1008						chr2	232915008	232919859	4851	HiConf								y
1009	chr2	233548756	233555322	6566	9	chr2	233548725	233555438	6713	single								
1010	chr2	233887983	233891217	3234	2	chr2	233887952	233891284	3332	single								
1011	chr2	233969960	233977849	7889	4	chr2	233969929	233978014	8085	single								
1012						chr2	234028533	234037304	8771	single								
1013	chr2	234313818	234331580	17762	8	chr2	234313762	234331941	18179	HiConf	Variation_37520	chr2	234313071	234324729	11658	Cooper et al. (2008)	Illumina Human 1M BeadChip	
1014	chr2	234607744	234625139	17395	4	chr2	234607713	234625500	17787	HiConf	Variation_1047	chr2	234608782	234620735	11953	Conrad et al. (2005)	Mendelian inconsistencies	
1015	chr2	234656695	234662624	5929	7	chr2	234656664	234662740	6076	HiConf	Variation_9980	chr2	234657651	234659626	1975	Wang et al. (2007)	Illumina HumanHap550 BeadChip	y
1016	chr2	235195297	235250255	54958	58	chr2	235195223	235250887	55664	HiConf								
1017	chr2	235379151	235380635	1484	4	chr2	235379120	235380835	1715	single								
1018	chr2	235675726	235675858	132	35	chr2	235675717	235675864	147	single	Variation_45967	chr2	235675668	235675854	186	Bentley et al. (2008)	Illumina DNA sequencing	y
1019	chr2	238108059	238135436	27377	40	chr2	238108028	238135762	27734	HiConf								y
1020	chr2	238164752	238177492	12740	25	chr2	238164721	238177853	13132	HiConf								
1021	chr2	238730359	238732760	2401	15	chr2	238730328	238732827	2499	single	Variation_30110	chr2	238678401	238747816	69415	Jakobsson et al. (2008)	Illumina HumanHap550 BeadChip	y
100172	chr2	239102157	239128449	26292	12	chr2	239101944	239128992	27048	HiConf								
1022	chr2	239147839	239149211	1372	4	chr2	239147808	239149229	1421	single								
1023	chr2	239165308	239166361	1053	48	chr2	239165301	239166379	1078	HiConf	Variation_23606	chr2	239165137	239166474	1337	Levy et al. (2007)	Sequencing	
1024	chr2	239672531	239673070	539	48	chr2	239672500	239673088	588	HiConf	Variation_35797	chr2	239663040	239673676	10636	Kidd et al. (2008)	Paired End Mapping	
1025	chr2	240231033	240231572	539	55	chr2	240231002	240231590	588	HiConf								
1026	chr2	240231915	240244067	12152	3	chr2	240231884	240244330	12446	single								
1027	chr2	240305268	240306689	1421	37	chr2	240305237	240306707	1470	HiConf								
1028	chr2	240326289	240327612	1323	10	chr2	240326258	240327630	1372	single								
1029	chr2	240476523	240498475	21952	3	chr2	240476492	240498934	22442	single								y
1030	chr2	240742754	240746775	4021	53	chr2	240740700	240746776	6076	HiConf								y
1031	chr2	240856763	240859801	3038	24	chr2	240856732	240859868	3136	single								y
1032	chr2	241509639	241514833	5194	33	chr2	241509608	241514949	5341	single	Variation_30111	chr2	241465995	241516747	50752	Jakobsson et al. (2008)	Illumina HumanHap550 BeadChip	
1033	chr2	241703630	241704169	539	67	chr2	241703599	241704187	588	HiConf								
1034	chr2	241954020	241957597	3577	5	chr2	241953989	241957664	3675	HiConf								
1035	chr2	242349744	242366747	17003	9	chr2	242349713	242367108	17395	single	Variation_11169	chr2	242359273	242360030	757	de Smith et al. (2007)	Agilent 185k CGH Arrays/Agilent Custom CGH Arrays	
1036	chr2	242662364	242688040	25676	4	chr2	242662333	242688548	26215	HiConf	Variation_32403	chr2	242668304	242692706	24402	Perry et al. (2008)	Agilent Custom CGH Arrays	
1037	chr2	242688696	242692842	4146	6	chr2	242688695	242692909	4214	single	Variation_1610	chr2	242675168	242695936	20768	McCarroll et al. (2005)	Null genotypes	
1038	chr2	242735913	242748849	12936	12	chr2	242735882	242749112	13230	single								
100925	chr20	187009	251965	64956	15	chr20	186960	253282	66322	HiConf	Variation_11179	chr20	203239	203844	605	de Smith et al. (2007)	Agilent 185k CGH Arrays/Agilent Custom CGH Arrays	
5623	chr20	975009	979811	4802	1	chr20	974978	979927	4949	single								
100926	chr20	1184750	1231647	46897	2	chr20	1157503	1233110	75607	single								
5624	chr20	1337805	1338736	931	43	chr20	1337774	1338754	980	HiConf	Variation_6042	chr20	1337144	1338814	1670	Mills et al. (2006)	Sequence trace read mapping	
5625	chr20	1500583	1507394	6811	14	chr20	1500552	1507510	6958	HiConf	Variation_37920	chr20	1505219	1508562	3343	McCarroll et al. (2008)	Affymetrix Human SNP Array 6.0	
5626	chr20	1508004	1542919	34915	27	chr20	1508000	1543623	35623	HiConf	Variation_22910	chr20	1508872	1542911	34039	Korbel et al. (2007)	Paired End Mapping	
5627	chr20	1618477	1622103	3626	7	chr20	1618446	1622170	3724	HiConf	Variation_32407	chr20	1618858	1622081	3223	Perry et al. (2008)	Agilent Custom CGH Arrays	
100928	chr20	1769887	1827315	57428	10	chr20	1769856	1828485	58629	HiConf	Variation_35908	chr20	1785670	1818094	32424	Kidd et al. (2008)	Paired End Mapping	
5629	chr20	2146403	2149147	2744	4	chr20	2146372	2149214	2842	single	Variation_4091	chr20	2078560	2423469	344909	Redon et al. (2006)	BAC Array CGH	
5630	chr20	2392971	2397871	4900	3	chr20	2392940	2397987	5047	single	Variation_4091	chr20	2078560	2423469	344909	Redon et al. (2006)	BAC Array CGH	
5631	chr20	3771243	3772468	1225	20	chr20	3771212	3772486	1274	single	Variation_44048	chr20	3769071	3773127	4056	Bentley et al. (2008)	Illumina DNA sequencing	
5632	chr20	3880072	3881689	1617	1	chr20	3880041	3881707	1666	single								
5633	chr20	4393445	4397708	4263	15	chr20	4393414	4397775	4361	HiConf	Variation_22540	chr20	4393121	4397992	4871	Korbel et al. (2007)	Paired End Mapping	
5634	chr20	4770059	4777899	7840	11	chr20	4770028	4778064	8036	single								
5635	chr20	5738005	5739034	1029	6	chr20	5737974	5739052	1078	single	Variation_47915	chr20	5643053	5764858	121805	Gusev et al. (2009)	SNP genotyping analysis	
5636	chr20	5974234	5975116	882	7	chr20	5974203	5975134	931	single								
5637	chr20	7193795	7202762	8967	2	chr20	7193764	7202927	9163	single								
5638	chr20	7346675	7351869	5194	20	chr20	7346644	7351985	5341	HiConf	Variation_5124	chr20	7288381	7476529	188148	Wong et al. (2007)	BAC Array CGH	
5639	chr20	7553014	7555415	2401	36	chr20	7552983	7555482	2499	HiConf	Variation_6057	chr20	7549037	7555572	6535	Mills et al. (2006)	Sequence trace read mapping	
5640	chr20	7984802	7988673	3871	2	chr20	7984771	7988740	3969	HiConf	Variation_5346	chr20	7897426	8392248	494822	Simon-Sanchez et al. (2007)	Illumina HumanHap300 BeadChip	
5641	chr20	8362151	8362313	162	39	chr20	8362120	8362316	196	HiConf	Variation_8879	chr20	8050867	8517610	466743	Pinto et al. (2007)	Affymetrix 500K SNP Mapping Array	
100929	chr20	10303874	10346873	42999	1	chr20	10303843	10383493	79650	single								
5642	chr20	11595563	11596234	671	12	chr20	11595532	11596610	1078	HiConf								y
100930	chr20	11749766	11931967	182201	4	chr20	11749735	11935764	186029	HiConf	Variation_31031	chr20	11768927	11770174	1247	Perry et al. (2008)	Agilent Custom CGH Arrays	
5649	chr20	12137650	12152644	14994	4	chr20	12137619	12152956	15337	HiConf	Variation_8880	chr20	11754600	12230700	476100	Pinto et al. (2007)	Affymetrix 500K SNP Mapping Array	
5650	chr20	12182299	12235013	52714	3	chr20	12182258	12236060	53802	HiConf	Variation_32410	chr20	12189781	12215934	26153	Perry et al. (2008)	Agilent Custom CGH Arrays	
5651	chr20	12274948	12284748	9800	9	chr20	12274917	12284962	10045	single	Variation_4095	chr20	11638021	12381393	743372	Redon et al. (2006)	BAC Array CGH	
5652	chr20	12531218	12531281	63	21	chr20	12531187	12531285	98	HiConf								
5653	chr20	12554689	12562083	7394	2	chr20	12554658	12562253	7595	HiConf								
5654	chr20	12597027	12607854	10827	3	chr20	12596994	12608068	11074	HiConf	Variation_3208	chr20	12581827	12609944	28117	Redon et al. (2006)	Affymetrix 500K EA SNP Mapping Array	
5655	chr20	12705364	12708794	3430	6	chr20	12705333	12708861	3528	HiConf								
5656	chr20	13153275	13154625	1350	1	chr20	13152164	13158926	6762	single								
5657	chr20	13547108	13553015	5907	4	chr20	13546761	13553131	6370	single								
100932	chr20	13649300	13723437	74137	3	chr20	13649269	13724950	75681	single								
5658	chr20	14220003	14226618	6615	8	chr20	14219972	14226734	6762	HiConf								
100933	chr20	14371107	14388219	17112	4	chr20	14367511	14403208	35697	HiConf	Variation_38439	chr20	14376080	14390471	14391	McCarroll et al. (2008)	Affymetrix Human SNP Array 6.0	
5660	chr20	14425362	14426734	1372	11	chr20	14425331	14426752	1421	HiConf	Variation_6062	chr20	14425294	14426955	1661	Mills et al. (2006)	Sequence trace read mapping	
5661	chr20	15082501	15089165	6664	3	chr20	15082470	15089281	6811	single								
5662	chr20	15249762	15251747	1985	12	chr20	15249756	15251765	2009	HiConf	Variation_6065	chr20	15249732	15251768	2036	Mills et al. (2006)	Sequence trace read mapping	
5663	chr20	15258999	15268456	9457	10	chr20	15258968	15268621	9653	HiConf	Variation_44042	chr20	15258901	15268654	9753	Bentley et al. (2008)	Illumina DNA sequencing	
5664	chr20	15924664	15941128	16464	9	chr20	15924633	15941440	16807	HiConf								
5665	chr20	16175348	16188872	13524	27	chr20	16175317	16189135	13818	HiConf	Variation_5127	chr20	16082347	16250763	168416	Wong et al. (2007)	BAC Array CGH	
100934	chr20	16517357	16534229	16872	3	chr20	16511702	16546860	35158	HiConf	Variation_38440	chr20	16516058	16534634	18576	McCarroll et al. (2008)	Affymetrix Human SNP Array 6.0	
5667	chr20	17950716	17951794	1078	31	chr20	17950685	17951812	1127	single								
5668	chr20	18197529	18201400	3871	1	chr20	18197498	18201467	3969	single	Variation_3213	chr20	18197877	18221632	23755	Redon et al. (2006)	Affymetrix 500K EA SNP Mapping Array	
5669	chr20	19790274	19790436	162	33	chr20	19790243	19790439	196	single								
5670	chr20	19815558	19822124	6566	4	chr20	19815527	19822240	6713	single	Variation_9821	chr20	19814572	19822222	7650	Wang et al. (2007)	Illumina HumanHap550 BeadChip	
100935	chr20	19842694	19857709	15015	3	chr20	19826283	19894540	68257	HiConf	Variation_6078	chr20	19856801	19866731	9930	Mills et al. (2006)	Sequence trace read mapping	
5672	chr20	20057475	20059676	2201	8	chr20	20057440	20059694	2254	single								
5673	chr20	20120436	20121906	1470	2	chr20	20120405	20121924	1519	single								

locus_id	chrom	start	end	length	Yoruba w/ event	putative chr	putative start	putative end	putative len	putative type	variation_id	DGV_chr	DGV_start	DGV_end	DGV_len	Reference	Method/platform	complex
100936	chr20	20843505	20873297	29792	9	chr20	20843474	20873903	30429	single								
5675	chr20	21234696	21236411	1715	50	chr20	21234665	21236429	1764	HiConf	Variation_39288	chr20	21234068	21236749	2681	Wheeler et al. (2008)	Sequencing	
5676	chr20	22272859	22278102	5243	6	chr20	22272828	22278218	5390	HiConf	Variation_32414	chr20	22274448	22277490	3042	Perry et al. (2008)	Agilent Custom CGH Arrays	
5677	chr20	22951215	22954792	3577	3	chr20	22951184	22954859	3675	single								
5678	chr20	23056075	23056761	686	33	chr20	23056044	23056779	735	single	Variation_4097	chr20	22977404	23121240	143836	Redon et al. (2006)	BAC Array CGH	
5679	chr20	23116510	23124692	8182	15	chr20	23116510	23126996	10486	HiConf	Variation_6082	chr20	23116344	23124309	7965	Mills et al. (2006)	Sequence trace read mapping	
5680	chr20	23619967	23622074	2107	17	chr20	23619936	23622092	2156	HiConf	Variation_44044	chr20	23619866	23622299	2433	Bentley et al. (2008)	Illumina DNA sequencing	
5681	chr20	23906617	23924061	17444	5	chr20	23906586	23924422	17836	HiConf	Variation_31033	chr20	23907810	23979343	71533	Perry et al. (2008)	Agilent Custom CGH Arrays	
5682	chr20	24359573	24360847	1274	24	chr20	24359542	24360865	1323	HiConf	Variation_32415	chr20	23907810	24584934	677124	Perry et al. (2008)	Agilent Custom CGH Arrays	
5683	chr20	24657836	24658522	686	19	chr20	24657805	24658540	735	single								
5684	chr20	25702271	25725938	23667	11	chr20	25702240	25726397	24157	HiConf	Variation_32416	chr20	25714237	25773787	59550	Perry et al. (2008)	Agilent Custom CGH Arrays	
5685	chr20	25774252	25781428	7176	2	chr20	25774221	25788039	13818	single	Variation_29708	chr20	25663507	26257255	593748	Jakobsson et al. (2008)	Illumina HumanHap550 BeadChip	
5686	chr20	25883767	25904200	20433	1	chr20	25883736	25904610	20874	single	Variation_29708	chr20	25663507	26257255	593748	Jakobsson et al. (2008)	Illumina HumanHap550 BeadChip	
5687	chr20	25997332	26018294	20962	5	chr20	25932736	26020005	87269	HiConf	Variation_31036	chr20	26002475	26010947	8472	Perry et al. (2008)	Agilent Custom CGH Arrays	
5688	chr20	26147681	26160519	12838	14	chr20	26147650	26160782	13132	HiConf	Variation_32420	chr20	26147451	26162148	14697	Perry et al. (2008)	Agilent Custom CGH Arrays	
5689	chr20	28034304	28095872	61568	2	chr20	28034297	28097115	62818	single	Variation_38441	chr20	28034013	28119552	85539	McCarroll et al. (2008)	Affymetrix Human SNP Array 6.0	
5690	chr20	28097416	28121009	23593	4	chr20	28097409	28121468	24059	single	Variation_38441	chr20	28034013	28119552	85539	McCarroll et al. (2008)	Affymetrix Human SNP Array 6.0	
5691	chr20	28134876	28159002	24126	15	chr20	28134845	28159198	24353	HiConf	Variation_31038	chr20	28128809	28149114	20305	Perry et al. (2008)	Agilent Custom CGH Arrays	
5692	chr20	29267952	29313738	45786	41	chr20	29267921	29326623	58702	HiConf	Variation_6084	chr20	29270756	29274157	3401	Mills et al. (2006)	Sequence trace read mapping	
5693	chr20	29941408	29945531	4123	13	chr20	29941377	29945885	4508	HiConf	Variation_9822	chr20	29941937	29945359	3422	Wang et al. (2007)	Illumina HumanHap550 BeadChip	
5694	chr20	30775166	30779798	4632	5	chr20	30770310	30779963	9653	single								
5695	chr20	31192966	31199826	6860	10	chr20	31192935	31199942	7007	single	Variation_4100	chr20	30959468	31211827	252359	Redon et al. (2006)	BAC Array CGH	
5696	chr20	31723489	31728829	5340	3	chr20	31723458	31732376	8918	single								
5697	chr20	32707190	32707270	80	32	chr20	32706839	32707280	441	HiConf	Variation_6093	chr20	32705521	32707945	2424	Mills et al. (2006)	Sequence trace read mapping	
5698	chr20	34096804	34101606	4802	14	chr20	34096773	34101722	4949	HiConf	Variation_5135	chr20	33989555	34143087	153532	Wong et al. (2007)	BAC Array CGH	
5699	chr20	35166621	35167356	735	4	chr20	35166590	35167374	784	single								
5700						chr20	35460590	35480043	19453	HiConf								
5701	chr20	35480760	35490952	10192	19	chr20	35480729	35491166	10437	HiConf								
5702	chr20	35908040	35910588	2548	3	chr20	35908009	35910655	2646	HiConf	Variation_5136	chr20	35879175	35971325	92150	Wong et al. (2007)	BAC Array CGH	
5703	chr20	36941548	36959972	18424	4	chr20	36941517	36960333	18816	HiConf								
5704	chr20	37289105	37296112	7007	1	chr20	37289074	37296228	7154	single								
5705	chr20	37296406	37310170	13764	1	chr20	37296375	37310438	14063	single								
5706	chr20	37336145	37338350	2205	3	chr20	37336114	37338368	2254	HiConf								
5707	chr20	37658075	37659790	1715	6	chr20	37658044	37659808	1764	single								
5708	chr20	37883426	37885827	2401	9	chr20	37883395	37885894	2499	single								
5709	chr20	38075408	38080258	4850	3	chr20	38075377	38080375	4998	HiConf	Variation_10581	chr20	38076193	38078497	2304	Wang et al. (2007)	Illumina HumanHap550 BeadChip	
5710	chr20	38450699	38458833	8134	1	chr20	38450668	38458998	8330	single								y
5711	chr20	38895815	38897040	1225	8	chr20	38895784	38897058	1274	single								
5712	chr20	38897285	38898559	1274	3	chr20	38897254	38898577	1323	single								
5713	chr20	38959907	38971044	11137	10	chr20	38959876	38971146	11270	HiConf								
5714	chr20	39340490	39340697	207	64	chr20	39340459	39340704	245	HiConf	Variation_35918	chr20	39313603	39346181	32578	Kidd et al. (2008)	Paired End Mapping	y
5715	chr20	40612285	40612685	400	24	chr20	40612254	40612695	441	single	Variation_8885	chr20	39856396	40702735	846339	Pinto et al. (2007)	Affymetrix 500K SNP Mapping Array	
100940	chr20	40689688	40711780	22092	4	chr20	40673088	40712582	39494	HiConf	Variation_10582	chr20	40688107	40711739	23632	Wang et al. (2007)	Illumina HumanHap550 BeadChip	y
5717	chr20	41127373	41139378	12005	8	chr20	41127342	41139592	12250	HiConf								y
5718	chr20	41163045	41163584	539	21	chr20	41163014	41163602	588	single	Variation_11185	chr20	41162911	41163648	737	de Smith et al. (2007)	Agilent 185k CGH Arrays/Agilent Custom CGH Arrays	
5719	chr20	41522656	41523685	1029	7	chr20	41522625	41523703	1078	single								
5720	chr20	41704642	41706406	1764	47	chr20	41704611	41706424	1813	HiConf								
5721	chr20	41914950	41917008	2058	27	chr20	41914919	41917369	2450	HiConf								
5722	chr20	42739522	42742070	2548	26	chr20	42739491	42742137	2646	HiConf	Variation_35920	chr20	42738760	42769954	31194	Kidd et al. (2008)	Paired End Mapping	
5723	chr20	42760906	42762748	1842	40	chr20	42760904	42762766	1862	HiConf	Variation_22498	chr20	42760299	42762978	2679	Korbel et al. (2007)	Paired End Mapping	
5724	chr20	45590734	45625672	34938	4	chr20	45590703	45626473	35770	single	Variation_7309	chr20	45205485	45713113	507628	de Smith et al. (2007)	Agilent 185k CGH Arrays/Agilent Custom CGH Arrays	
5725	chr20	45884783	45896788	12005	3	chr20	45884752	45897002	12250	HiConf	Variation_32425	chr20	45884294	45888652	4358	Perry et al. (2008)	Agilent Custom CGH Arrays	
5727	chr20	46183973	46184133	160	6	chr20	46183652	46184142	490	single								
5728	chr20	46532759	46537610	4851	18	chr20	46532728	46537726	4998	HiConf								
5729	chr20	46557455	46565638	8183	13	chr20	46557424	46565803	8379	HiConf	Variation_35930	chr20	46561525	46568448	6923	Kidd et al. (2008)	Paired End Mapping	
5730	chr20	47657309	47682201	24892	15	chr20	47657278	47682709	25431	HiConf								
5731	chr20	48377368	48378883	1515	3	chr20	48375863	48378950	3087	single								
5732	chr20	50193353	50193892	539	16	chr20	50193322	50193910	588	single	Variation_6120	chr20	50192598	50194335	1737	Mills et al. (2006)	Sequence trace read mapping	
5733	chr20	51765273	51770915	5642	18	chr20	51765242	51770975	5733	HiConf	Variation_35932	chr20	51748569	51773832	25263	Kidd et al. (2008)	Paired End Mapping	y
5734	chr20	51871603	51899380	27777	28	chr20	51871572	51899551	27979	HiConf	Variation_26852	chr20	51891483	51891656	173	Levy et al. (2007)	Sequencing	
5735	chr20	53037117	53037999	882	19	chr20	53037086	53038017	931	HiConf								y
5736	chr20	54450473	54452139	1666	21	chr20	54450442	54452157	1715	HiConf								
5737	chr20	55226976	55227845	869	3	chr20	55226945	55230914	3969	single								y
5738	chr20	55426014	55427729	1715	5	chr20	55425983	55427747	1764	single								
5739	chr20	55668858	55669258	400	30	chr20	55668827	55669268	441	single	Variation_31042	chr20	55665515	55674060	8545	Perry et al. (2008)	Agilent Custom CGH Arrays	
100942						chr20	56277971	56310041	32070	single								
5740	chr20	56594174	56594762	588	13	chr20	56594143	56594780	637	HiConf								
100943	chr20	56663881	56694974	31093	1	chr20	56658235	56698170	39935	single								
5741	chr20	57041740	57042818	1078	23	chr20	57041709	57042836	1127	single								
5742						chr20	57772005	57777052	5047	single								
5743	chr20	57823633	57838235	14602	14	chr20	57823602	57838547	14945	HiConf								
5744	chr20	57987538	58006256	18718	3	chr20	57987507	58006617	19110	single	Variation_10585	chr20	57998636	58005901	7265	Wang et al. (2007)	Illumina HumanHap550 BeadChip	
5745	chr20	58829771	58843666	13895	5	chr20	58829719	58843929	14210	HiConf	Variation_38447	chr20	58831790	58841969	10179	McCarroll et al. (2008)	Affymetrix Human SNP Array 6.0	
5746	chr20	59398395	59399081	686	20	chr20	59398364	59399099	735	single								
5747	chr20	59792012	59792796	784	53	chr20	59791981	59792814	833	single	Variation_12212	chr20	59792752	59792939	187	Mills et al. (2006)	Sequence trace read mapping	y
5748	chr20	59925635	59930752	5117	27	chr20	59925604	59930798	5194	HiConf	Variation_42557	chr20	59928278	59928781	503	Wang et al. (2008)	Illumina DNA sequencing	
5749	chr20	59979388	59980025	637	18	chr20	59979357	59980043	686	HiConf	Variation_33354	chr20	59979622	59979994	372	Perry et al. (2008)	Agilent Custom CGH Arrays	
5750	chr20	60218312	60223310	4998	13	chr20	60218281	60223426	5145	single	Variation_30129	chr20	60220455	60386791	166336	Jakobsson et al. (2008)	Illumina HumanHap550 BeadChip	
5751	chr20	61151733	61153397	1664	3	chr20	61149526	61155945	6419	single	Variation_5145	chr20	61041181	61192343	151162	Wong et al. (2007)	BAC Array CGH	
5752	chr20	61441254	61442332	1078	57	chr20	61441223	61442350	1127	single	Variation_31044	chr20	60877832	61701456	823624	Perry et al. (2008)	Agilent Custom CGH Arrays	
5753						chr20	61918189	61923579	5390	single								y
5754	chr20	62226306	62238953	12647	65	chr20	62226301	62239041	12740	HiConf	Variation_6130	chr20	62234272	62235717	1445	Mills et al. (2006)	Sequence trace read mapping	
5755	chr20	62280428	62280918	490	23	chr20	62280397	62280936	539	single	Variation_46136	chr20	62280224	62280918	694	Bentley et al. (2008)	Illumina DNA sequencing	
5756	chr20	62372695	62373185	490	46	chr20	62372664	62373203	539	HiConf	Variation_26919	chr20	62372854	62373178	324	Levy et al. (2007)	Sequencing	y
5757	chr20	62390199	62432812	42613	30	chr20	62387560	62432885	45325	HiConf	Variation_32432	chr20	62406565	62431887	25322	Perry et al. (2008)	Agilent Custom CGH Arrays	y
5758	chr21	9724204	9905083	180879	42	chr21	9724173	9905424	181251	HiConf	Variation_38955	chr21	9758742	9965425	206683	McCarroll et al. (2008)	Affymetrix Human SNP Array 6.0	

locus_id	chrom	start	end	length	Yoruba w/ event	putative chr	putative start	putative end	putative len	putative type	variation_id	DGV_chr	DGV_start	DGV_end	DGV_len	Reference	Method/platform	complex
5759	chr21	10033492	10125514	92022	6	chr21	10033461	10127394	93933	HiConf	Variation_5156	chr21	10037729	10121693	83964	Wong et al. (2007)	BAC Array CGH	
5760	chr21	10131002	10207181	76179	9	chr21	10130971	10207558	76587	single	Variation_32435	chr21	10173916	10201954	28038	Perry et al. (2008)	Agilent Custom CGH Arrays	
5761	chr21	13648908	13659541	10633	5	chr21	13648877	13659755	10878	single	Variation_31055	chr21	13638763	13663958	25195	Perry et al. (2008)	Agilent Custom CGH Arrays	
5762	chr21	13766165	13770591	4426	7	chr21	13766134	13770642	4508	single	Variation_0810	chr21	13677346	13829345	151999	Sharp et al. (2005)	BAC Array CGH	
5763	chr21	13837460	13838440	980	8	chr21	13837429	13838458	1029	single	Variation_0811	chr21	13831889	13995202	163313	Sharp et al. (2005)	BAC Array CGH	
100946	chr21	13926741	14134616	207875	6	chr21	13879863	14139906	260043	HiConf	Variation_5157	chr21	13879420	14043771	164351	Wong et al. (2007)	BAC Array CGH	
5765	chr21	14358624	14364357	5733	13	chr21	14358593	14364473	5880	HiConf								
5766	chr21	15510343	15511202	859	23	chr21	15510338	15511220	882	HiConf	Variation_23633	chr21	15510251	15513325	3074	Levy et al. (2007)	Sequencing	
5767	chr21	15546090	15550598	4508	3	chr21	15546059	15550665	4606	HiConf	Variation_38449	chr21	15546277	15550528	4251	McCarroll et al. (2008)	Affymetrix Human SNP Array 6.0	
5768	chr21	16865660	16866395	735	5	chr21	16865629	16866413	784	HiConf	Variation_46144	chr21	16865519	16866472	953	Bentley et al. (2008)	Illumina DNA sequencing	
5769						chr21	17996794	18017766	20972	single								
5770	chr21	18248538	18250946	2408	22	chr21	18248507	18251006	2499	HiConf	Variation_23636	chr21	18248710	18251219	2509	Levy et al. (2007)	Sequencing	
5771	chr21	19375461	19376567	1106	2	chr21	19372665	19376634	3969	single	Variation_9325	chr21	18329683	21294600	2964917	Pinto et al. (2007)	Affymetrix 500K SNP Mapping Array	
5772	chr21	19758718	19765382	6664	49	chr21	19758687	19765498	6811	HiConf	Variation_22670	chr21	19758856	19765477	6621	Korbel et al. (2007)	Paired End Mapping	
5773	chr21	19914195	19948740	34545	1	chr21	19914164	19949444	35280	single	Variation_9830	chr21	19919785	19947130	27345	Wang et al. (2007)	Illumina HumanHap550 BeadChip	
5774	chr21	20111322	20112302	980	1	chr21	20111291	20112320	1029	single	Variation_9325	chr21	18329683	21294600	2964917	Pinto et al. (2007)	Affymetrix 500K SNP Mapping Array	
100947	chr21	20131731	20189110	57379	1	chr21	20131700	20190279	58579	single	Variation_9325	chr21	18329683	21294600	2964917	Pinto et al. (2007)	Affymetrix 500K SNP Mapping Array	
5775	chr21	20534045	20542473	8428	3	chr21	20534014	20542638	8624	HiConf	Variation_9325	chr21	18329683	21294600	2964917	Pinto et al. (2007)	Affymetrix 500K SNP Mapping Array	
5776	chr21	20543992	20545707	1715	21	chr21	20543961	20545725	1764	HiConf	Variation_9325	chr21	18329683	21294600	2964917	Pinto et al. (2007)	Affymetrix 500K SNP Mapping Array	
100948	chr21	20734656	20771143	36487	4	chr21	20719773	20787369	67596	HiConf	Variation_32442	chr21	20724567	20766825	42258	Perry et al. (2008)	Agilent Custom CGH Arrays	
5778	chr21	20922125	20932856	10731	19	chr21	20922094	20933070	10976	HiConf	Variation_37939	chr21	20927716	20932562	4846	McCarroll et al. (2008)	Affymetrix Human SNP Array 6.0	
5779	chr21	22110228	22123703	13475	3	chr21	22110197	22123966	13769	single	Variation_0232	chr21	22059729	22195086	135357	Iafrate et al. (2004)	BAC Array CGH	
5780	chr21	22681225	22690192	8967	1	chr21	22681194	22690357	9163	single								
100949	chr21	23098367	23131682	33315	4	chr21	23066677	23149977	83300	HiConf	Variation_38451	chr21	23098732	23132654	33922	McCarroll et al. (2008)	Affymetrix Human SNP Array 6.0	
5782	chr21	23317245	23378054	60809	17	chr21	23317214	23379297	62083	HiConf	Variation_9326	chr21	23321000	23372706	51706	Pinto et al. (2007)	Affymetrix 500K SNP Mapping Array	
5783	chr21	24180478	24184839	4361	67	chr21	24180447	24184906	4459	HiConf	Variation_5349	chr21	24077624	24332553	254929	Simon-Sanchez et al. (2007)	Illumina HumanHap300 BeadChip	
5784	chr21	24210319	24211348	1029	5	chr21	24210288	24211366	1078	single	Variation_5349	chr21	24077624	24332553	254929	Simon-Sanchez et al. (2007)	Illumina HumanHap300 BeadChip	
100951	chr21	25253481	25311496	58015	22	chr21	25253351	25312690	59339	HiConf								
5786	chr21	25784003	25787188	3185	10	chr21	25783972	25787255	3283	HiConf								
5787	chr21	26096868	26101523	4655	6	chr21	26096837	26101590	4753	HiConf	Variation_38454	chr21	26097651	26100417	2766	McCarroll et al. (2008)	Affymetrix Human SNP Array 6.0	
5788	chr21	27116950	27124202	7252	13	chr21	27116919	27124367	7448	HiConf	Variation_10600	chr21	27117916	27122320	4404	Wang et al. (2007)	Illumina HumanHap550 BeadChip	
5789	chr21	27271349	27274632	3283	2	chr21	27271318	27274699	3381	HiConf								
5790	chr21	27372926	27380962	8036	1	chr21	27372895	27381127	8232	single	Variation_4113	chr21	27302101	27462314	160213	Redon et al. (2006)	BAC Array CGH	
5791	chr21	28205289	28206322	1033	2	chr21	28205258	28206336	1078	single								
5792	chr21	28635019	28635439	420	18	chr21	28634988	28635772	784	HiConf	Variation_44056	chr21	28634910	28635993	1083	Bentley et al. (2008)	Illumina DNA sequencing	
5793	chr21	29643439	29648094	4655	2	chr21	29643408	29648161	4753	HiConf								
5794	chr21	30214485	30214975	490	9	chr21	30214454	30214993	539	single								
5795	chr21	30490159	30492529	2370	2	chr21	30490128	30494538	4410	single								
5796	chr21	30561111	30566158	5047	10	chr21	30561080	30566519	5439	HiConf	Variation_44057	chr21	30560990	30566517	5527	Bentley et al. (2008)	Illumina DNA sequencing	
100952	chr21	31959544	32004793	45249	3	chr21	31935996	32026450	90454	single								
5797						chr21	32725459	32734573	9114	single								
5798	chr21	34452495	34454504	2009	30	chr21	34452464	34454522	2058	HiConf								
5799	chr21	34521781	34523692	1911	4	chr21	34521750	34523710	1960	single								
5800	chr21	34994827	34995137	310	45	chr21	34994796	34995139	343	HiConf								
5801	chr21	35482916	35487571	4655	5	chr21	35482885	35487638	4753	HiConf	Variation_47936	chr21	35304204	35704075	399871	Gusev et al. (2009)	SNP genotyping analysis	
5802	chr21	35523292	35523978	686	4	chr21	35523261	35523996	735	single	Variation_47936	chr21	35304204	35704075	399871	Gusev et al. (2009)	SNP genotyping analysis	y
5803	chr21	36287055	36289260	2205	31	chr21	36287024	36289278	2254	HiConf								
5804	chr21	36785973	36801336	15363	13	chr21	36785942	36801573	15631	HiConf								
5805	chr21	37071741	37078993	7252	5	chr21	37071710	37079158	7448	HiConf	Variation_35970	chr21	37069002	37086441	17439	Kidd et al. (2008)	Paired End Mapping	
5806	chr21	38573199	38576825	3626	4	chr21	38573168	38576892	3724	single	Variation_7324	chr21	38573989	38576625	2636	de Smith et al. (2007)	Agilent 185k CGH Arrays/Agilent Custom CGH Arrays	
5807	chr21	38896403	38898755	2352	4	chr21	38896372	38898773	2401	HiConf								
5808	chr21	39656393	39658800	2407	8	chr21	39656362	39658861	2499	HiConf	Variation_38456	chr21	39656792	39658882	2090	McCarroll et al. (2008)	Affymetrix Human SNP Array 6.0	
5809	chr21	39870229	39878314	8085	4	chr21	39870198	39878479	8281	HiConf	Variation_33358	chr21	39874611	39875064	453	Perry et al. (2008)	Agilent Custom CGH Arrays	
5810	chr21	40442745	40443725	980	12	chr21	40442714	40443743	1029	single	Variation_43189	chr21	40442966	40443873	907	Wang et al. (2008)	Illumina DNA sequencing	y
5811	chr21	40465578	40466706	1128	11	chr21	40465499	40466724	1225	HiConf								
5812	chr21	40466951	40489050	22099	21	chr21	40466920	40489509	22589	HiConf								
5813	chr21	40971112	40981364	10252	8	chr21	40971081	40981469	10388	HiConf								
5814	chr21	41910638	41911912	1274	11	chr21	41910607	41911930	1323	single								
5815	chr21	42123984	42136309	12325	6	chr21	42123953	42136546	12593	single								
5816	chr21	42641865	42642747	882	28	chr21	42641834	42642765	931	HiConf								y
5817	chr21	42770784	42773877	3093	14	chr21	42770753	42773938	3185	single								
5818	chr21	43076838	43080415	3577	14	chr21	43076807	43080482	3675	single								y
5819	chr21	43632400	43633674	1274	60	chr21	43632369	43633692	1323	HiConf								
5820	chr21	43794345	43799282	4937	50	chr21	43794314	43799312	4998	HiConf	Variation_43516	chr21	43794767	43797841	3074	Wang et al. (2008)	Illumina DNA sequencing	
5821	chr21	44079280	44089913	10633	11	chr21	44079249	44090127	10878	HiConf	Variation_5162	chr21	43979753	44118974	139221	Wong et al. (2007)	BAC Array CGH	y
5822	chr21	44205700	44209081	3381	11	chr21	44205669	44209148	3479	single	Variation_7327	chr21	43993840	44891970	898130	de Smith et al. (2007)	Agilent 185k CGH Arrays/Agilent Custom CGH Arrays	
100954	chr21	44782161	44939010	156849	1	chr21	44782130	44942188	160058	single	Variation_9329	chr21	44814874	44879100	64226	Pinto et al. (2007)	Affymetrix 500K SNP Mapping Array	
5823	chr21	46125388	46127578	2190	1	chr21	46120393	46127694	7301	single	Variation_3234	chr21	46067460	46144196	76736	Redon et al. (2006)	Affymetrix 500K EA SNP Mapping Array	
5824	chr21	46140906	46141306	400	25	chr21	46140875	46141316	441	single	Variation_3234	chr21	46067460	46144196	76736	Redon et al. (2006)	Affymetrix 500K EA SNP Mapping Array	
5825	chr21	46162809	46163209	400	34	chr21	46162778	46163219	441	single								
5826	chr21	46167030	46170460	3430	47	chr21	46164689	46170520	5831	HiConf	Variation_46190	chr21	46168065	46168478	413	Bentley et al. (2008)	Illumina DNA sequencing	
5827	chr21	46176382	46176832	450	32	chr21	46176351	46176841	490	single								
5828	chr21	46626545	46629361	2816	57	chr21	46626514	46629748	3234	HiConf	Variation_46196	chr21	46627164	46627362	198	Bentley et al. (2008)	Illumina DNA sequencing	
5829	chr22	14560896	14565257	4361	28	chr22	14560865	14565324	4459	HiConf	Variation_0331	chr22	14491957	14623461	131504	Sebat et al. (2004)	ROMA	
5830	chr22	14578585	14614551	35966	17	chr22	14578554	14615255	36701	single	Variation_0331	chr22	14491957	14623461	131504	Sebat et al. (2004)	ROMA	
5831	chr22	14672005	14707073	35068	21	chr22	14671850	14707767	35917	single	Variation_4821	chr22	14560644	14747014	186370	Wong et al. (2007)	BAC Array CGH	
5832	chr22	14726026	14841480	115454	17	chr22	14725995	14843889	117894	HiConf	Variation_0813	chr22	14759229	14899909	140680	Sharp et al. (2005)	BAC Array CGH	
5833	chr22	14904729	14930356	25627	12	chr22	14904698	14930864	26166	single	Variation_38459	chr22	14870976	15029985	159009	McCarroll et al. (2008)	Affymetrix Human SNP Array 6.0	
5834	chr22	14972496	14982345	9849	9	chr22	14972465	14982559	10094	single	Variation_38459	chr22	14870976	15029985	159009	McCarroll et al. (2008)	Affymetrix Human SNP Array 6.0	
5835	chr22	15258133	15417003	158870	27	chr22	15250687	15418610	167923	HiConf	Variation_32446	chr22	15280543	15393600	113057	Perry et al. (2008)	Agilent Custom CGH Arrays	
5836						chr22	15436299	15438161	1862	single								
5837	chr22	15729301	15771862	42561	6	chr22	15729270	15772292	43022	HiConf	Variation_32448	chr22	15733195	15767050	33855	Perry et al. (2008)	Agilent Custom CGH Arrays	
5838	chr22	15875419	15898057	22638	14	chr22	15875388	15898516	23128	HiConf	Variation_34559	chr22	14433758	15904203	1470445	Zogopoulos et al. (2007)	Affymetrix 500K and 100K SNP Mapping Arrays	
5839	chr22	16437988	16439066	1078	48	chr22	16437957	16439084	1127	HiConf	Variation_11196	chr22	16437998	16438709	711	de Smith et al. (2007)	Agilent 185k CGH Arrays/Agilent Custom CGH Arrays	
5840	chr22	16507960	16508968	1008	19	chr22	16507929	16509350	1421	single								y
100956	chr22	16717050	16778886	61836	53	chr22	16716914	16779659	62745	HiConf	Variation_46203	chr22	16739440	16739814	374	Bentley et al. (2008)	Illumina DNA sequencing	

locus_id	chrom	start	end	length	Yoruba w/ event	putative chr	putative start	putative end	putative len	putative type	variation_id	DGV_chr	DGV_start	DGV_end	DGV_len	Reference	Method/platform	complex
5843	chr22	16871197	16872912	1715	51	chr22	16871166	16872930	1764	HiConf								
5844	chr22	16999675	17004468	4793	8	chr22	16999644	17004789	5145	HiConf	Variation_31069	chr22	16999477	17001104	1627	Perry et al. (2008)	Agilent Custom CGH Arrays	
100957	chr22	17023868	17270866	246998	24	chr22	17023777	17275882	252105	HiConf	Variation_2259	chr22	17059974	17275750	215776	Locke et al. (2006)	BAC Array CGH	
5846	chr22	17393047	17402308	9261	5	chr22	17393016	17402473	9457	single	Variation_8899	chr22	17270946	17417535	146589	Pinto et al. (2007)	Affymetrix 500K SNP Mapping Array	y
5847	chr22	17937045	17938515	1470	47	chr22	17937014	17938533	1519	HiConf	Variation_8901	chr22	17803250	18146782	343532	Pinto et al. (2007)	Affymetrix 500K SNP Mapping Array	
5848	chr22	17951745	17952333	588	67	chr22	17951714	17952351	637	HiConf	Variation_6142	chr22	17951619	17953101	1482	Mills et al. (2006)	Sequence trace read mapping	y
100958	chr22	18650877	19089566	438689	46	chr22	18650846	19091626	440780	HiConf	Variation_7332	chr22	18691733	18989576	297843	de Smith et al. (2007)	Agilent 185k CGH Arrays/Agilent Custom CGH Arrays	
5850	chr22	19372513	19382033	9520	4	chr22	19368402	19402408	34006	HiConf	Variation_31071	chr22	17399088	19383198	1984110	Perry et al. (2008)	Agilent Custom CGH Arrays	
5851	chr22	19417384	19421886	4502	2	chr22	19417353	19422253	4900	single								
5852						chr22	19693370	19698172	4802	HiConf								
5853	chr22	19786354	19793802	7448	5	chr22	19786323	19793967	7644	HiConf	Variation_1310	chr22	19786518	19791274	4756	Conrad et al. (2005)	Mendelian inconsistencies	y
5854	chr22	19795811	20122788	326977	30	chr22	19795780	20129470	333690	HiConf	Variation_31072	chr22	19796080	20029903	233823	Perry et al. (2008)	Agilent Custom CGH Arrays	
5855	chr22	20136273	20243279	107006	6	chr22	20136183	20245453	109270	HiConf	Variation_5173	chr22	20105069	20280089	175020	Wong et al. (2007)	BAC Array CGH	
5856	chr22	20704075	20709104	5029	2	chr22	20704044	20709434	5390	HiConf	Variation_4118	chr22	20625103	20738604	113501	Redon et al. (2006)	BAC Array CGH	
5857	chr22	20710631	20746215	35584	3	chr22	20710561	20746919	36358	single	Variation_2011	chr22	20716434	20724227	7793	McCarroll et al. (2005)	Mendelian inconsistencies	
5858	chr22	20747067	20839044	91977	6	chr22	20747066	20839774	92708	HiConf	Variation_23322	chr22	20802998	20840725	37727	Levy et al. (2007)	Agilent 244k CGH Arrays	
5859	chr22	20934179	20988477	54298	4	chr22	20934148	20998828	64680	HiConf	Variation_2265	chr22	20885600	21052931	167331	Locke et al. (2006)	BAC Array CGH	
5860	chr22	20999545	21091730	92185	8	chr22	20999514	21092957	93443	HiConf	Variation_7333	chr22	21007308	21086336	79028	de Smith et al. (2007)	Agilent 185k CGH Arrays/Agilent Custom CGH Arrays	
5861	chr22	21093105	21094850	1745	12	chr22	21093104	21094868	1764	HiConf	Variation_23324	chr22	21031758	21160583	128825	Levy et al. (2007)	Agilent 244k CGH Arrays	y
5862	chr22	21094971	21102151	7180	11	chr22	21094966	21102267	7301	HiConf	Variation_9834	chr22	21100917	21191994	91077	Wang et al. (2007)	Illumina HumanHap550 BeadChip	
5863	chr22	21102370	21136469	34099	17	chr22	21102365	21137057	34692	HiConf	Variation_10616	chr22	21122400	21169559	47159	Wang et al. (2007)	Illumina HumanHap550 BeadChip	
5864	chr22	21137205	21203472	66267	14	chr22	21137204	21204481	67277	HiConf	Variation_9836	chr22	21163756	21196423	32667	Wang et al. (2007)	Illumina HumanHap550 BeadChip	
5865						chr22	21204628	21204677	49	single								
5866	chr22	21204879	21222348	17469	7	chr22	21204873	21222709	17836	single	Variation_36002	chr22	21205451	21213495	8044	Kidd et al. (2008)	Paired End Mapping	
5867	chr22	21222911	21242193	19282	20	chr22	21222905	21242603	19698	single	Variation_44063	chr22	21236062	21239456	3394	Bentley et al. (2008)	Illumina DNA sequencing	
5868	chr22	21243271	21243908	637	21	chr22	21243240	21243926	686	single	Variation_9835	chr22	21147621	21250212	102591	Wang et al. (2007)	Illumina HumanHap550 BeadChip	
5869	chr22	21244659	21297438	52779	12	chr22	21244563	21298561	53998	HiConf	Variation_9837	chr22	21270634	21295702	25068	Wang et al. (2007)	Illumina HumanHap550 BeadChip	
5870	chr22	21303639	21336567	32928	10	chr22	21303608	21337222	33614	HiConf	Variation_5176	chr22	21253838	21445636	191798	Wong et al. (2007)	BAC Array CGH	
5871	chr22	21337370	21337660	290	19	chr22	21337369	21337663	294	single	Variation_5176	chr22	21253838	21445636	191798	Wong et al. (2007)	BAC Array CGH	y
5872	chr22	21503461	21578549	75088	43	chr22	21503430	21578743	75313	HiConf	Variation_36007	chr22	21487340	21586552	99212	Kidd et al. (2008)	Paired End Mapping	
5873	chr22	21686721	21695345	8624	1	chr22	21686690	21695510	8820	single	Variation_48008	chr22	21546762	21829513	282751	Gusev et al. (2009)	SNP genotyping analysis	
5874	chr22	21983024	21989737	6713	4	chr22	21982993	21989853	6860	HiConf	Variation_32451	chr22	21983119	22145329	162210	Perry et al. (2008)	Agilent Custom CGH Arrays	
5875	chr22	21998900	22002673	3773	18	chr22	21998869	22002740	3871	single	Variation_32451	chr22	21983119	22145329	162210	Perry et al. (2008)	Agilent Custom CGH Arrays	
5876	chr22	22136396	22152319	15923	29	chr22	22136363	22152631	16268	HiConf	Variation_7336	chr22	22138362	22148276	9914	de Smith et al. (2007)	Agilent 185k CGH Arrays/Agilent Custom CGH Arrays	
5877	chr22	22153838	22155504	1666	23	chr22	22153807	22155522	1715	single	Variation_5179	chr22	22078703	22232492	153789	Wong et al. (2007)	BAC Array CGH	y
5878	chr22	22173095	22177897	4802	23	chr22	22173064	22177964	4900	single	Variation_5179	chr22	22078703	22232492	153789	Wong et al. (2007)	BAC Array CGH	
5879	chr22	22212001	22220478	8477	21	chr22	22211970	22220643	8673	single	Variation_5179	chr22	22078703	22232492	153789	Wong et al. (2007)	BAC Array CGH	
100961	chr22	22238849	22312897	74048	2	chr22	22238626	22314405	75779	HiConf	Variation_22929	chr22	22245900	22313362	67462	Korbel et al. (2007)	Paired End Mapping	
5881	chr22	22355767	22357384	1617	14	chr22	22355736	22357402	1666	single								
5882	chr22	22391684	22401199	9515	3	chr22	22391653	22401355	9702	single								y
5883	chr22	22604148	22625120	20972	44	chr22	22604117	22625530	21413	HiConf	Variation_22918	chr22	22603584	22641689	38105	Korbel et al. (2007)	Paired End Mapping	y
5884	chr22	22658244	22732053	73809	42	chr22	22658213	22732203	73990	HiConf	Variation_7338	chr22	22671397	22734855	63458	de Smith et al. (2007)	Agilent 185k CGH Arrays/Agilent Custom CGH Arrays	
5885	chr22	22962828	23003939	41111	14	chr22	22962797	23004790	41993	HiConf	Variation_32454	chr22	22963326	22991179	27853	Perry et al. (2008)	Agilent Custom CGH Arrays	
5886	chr22	23206622	23206912	290	44	chr22	23206621	23206915	294	HiConf	Variation_40495	chr22	23206431	23207008	577	Wheeler et al. (2008)	Sequencing	
100963	chr22	23330390	23396264	65874	26	chr22	23321134	23397574	76440	HiConf	Variation_32456	chr22	23335009	23386483	51474	Perry et al. (2008)	Agilent Custom CGH Arrays	
5888	chr22	23780442	23781618	1176	7	chr22	23780411	23781636	1225	HiConf	Variation_2269	chr22	23742887	23902859	159972	Locke et al. (2006)	BAC Array CGH	
100964	chr22	23950157	24250450	300293	7	chr22	23949755	24256569	306814	HiConf	Variation_9331	chr22	23971025	24255078	284053	Pinto et al. (2007)	Affymetrix 500K SNP Mapping Array	
100965	chr22	24798628	24864782	66154	13	chr22	24779472	24866570	87098	HiConf	Variation_2273	chr22	24843140	25024294	181154	Locke et al. (2006)	BAC Array CGH	
5894						chr22	25284858	25297647	12789	single								y
5895	chr22	25462073	25462612	539	31	chr22	25462042	25462630	588	single								
100966	chr22	25486059	25499739	13680	13	chr22	25486028	25517167	31139	single	Variation_44067	chr22	25498075	25499791	1716	Bentley et al. (2008)	Illumina DNA sequencing	
5897	chr22	27261330	27276462	15132	5	chr22	27254315	27283421	29106	HiConf								
5898	chr22	27409137	27421281	12144	10	chr22	27409106	27421797	12691	HiConf	Variation_32463	chr22	27402654	27421731	19077	Perry et al. (2008)	Agilent Custom CGH Arrays	
5899	chr22	27837936	27860917	22981	34	chr22	27837905	27861376	23471	HiConf								
5900	chr22	28008701	28011739	3038	12	chr22	28008670	28011806	3136	single								
5901	chr22	28302456	28304906	2450	15	chr22	28302425	28304973	2548	HiConf								
5902	chr22	28666624	28667024	400	51	chr22	28666593	28667034	441	single								
5903	chr22	29191120	29192198	1078	15	chr22	29191089	29192216	1127	single								
5904	chr22	29790096	29804404	14308	4	chr22	29790065	29804667	14602	HiConf	Variation_31089	chr22	29785329	29795665	10336	Perry et al. (2008)	Agilent Custom CGH Arrays	
5905	chr22	30629452	30629624	172	19	chr22	30629386	30629631	245	single								
5906	chr22	31041164	31043124	1960	1	chr22	31041133	31043142	2009	single	Variation_5358	chr22	30816448	31118258	301810	Simon-Sanchez et al. (2007)	Illumina HumanHap300 BeadChip	
5907	chr22	31486427	31487603	1176	30	chr22	31486396	31487621	1225	single								
5908	chr22	31992597	31994271	1674	10	chr22	31992566	31994281	1715	single								
5909	chr22	32066538	32066938	400	21	chr22	32066507	32066948	441	single								
5910	chr22	32086726	32088882	2156	4	chr22	32086695	32088900	2205	single	Variation_7344	chr22	32087443	32090021	2578	de Smith et al. (2007)	Agilent 185k CGH Arrays/Agilent Custom CGH Arrays	
5911	chr22	32110932	32113529	2597	3	chr22	32110901	32113596	2695	single								
5912	chr22	33117455	33122096	4641	4	chr22	33111236	33122310	11074	HiConf								
5913	chr22	33127685	33130181	2496	9	chr22	33127602	33130248	2646	single								
5914	chr22	33230288	33231121	833	20	chr22	33230257	33231139	882	HiConf								
5915	chr22	34083525	34091813	8288	2	chr22	34083494	34092216	8722	HiConf	Variation_36019	chr22	34072444	34086028	13584	Kidd et al. (2008)	Paired End Mapping	
5916	chr22	34807059	34811714	4655	7	chr22	34807028	34811781	4753	HiConf								
5917	chr22	34894475	34905108	10633	18	chr22	34894444	34905322	10878	HiConf	Variation_22425	chr22	34901862	34902461	599	Wang et al. (2007)	Illumina HumanHap550 BeadChip	
5918	chr22	34942936	34943965	1029	49	chr22	34942905	34943983	1078	HiConf	Variation_44070	chr22	34942950	34944265	1315	Bentley et al. (2008)	Illumina DNA sequencing	
5919	chr22	35273098	35281428	8330	11	chr22	35273067	35281593	8526	HiConf	Variation_32464	chr22	35273438	35279081	5643	Perry et al. (2008)	Agilent Custom CGH Arrays	
5920						chr22	35299870	35306975	7105	single								
100968	chr22	35319032	35370058	51026	4	chr22	35318980	35371116	52136	HiConf	Variation_4126	chr22	35236509	35371980	135471	Redon et al. (2006)	BAC Array CGH	y
5921	chr22	35473459	35476840	3381	28	chr22	35473428	35476907	3479	single	Variation_7347	chr22	35473736	35476971	3235	de Smith et al. (2007)	Agilent 185k CGH Arrays/Agilent Custom CGH Arrays	
100969	chr22	36045970	36065972	20002	3	chr22	36028206	36066769	38563	single	Variation_30163	chr22	36037950	36083945	45995	Jakobsson et al. (2008)	Illumina HumanHap550 BeadChip	
5923	chr22	36449858	36450699	841	44	chr22	36449851	36450733	882	single	Variation_33725	chr22	36449937	36450879	942	Perry et al. (2008)	Agilent Custom CGH Arrays	
5924	chr22	36912540	36919749	7209	18	chr22	36912509	36919908	7399	single	Variation_5186	chr22	36789449	36966456	177007	Wong et al. (2007)	BAC Array CGH	
5925						chr22	37091359	37107627	16268	single								
5926						chr22	37156039	37169612	13573	single								
5927	chr22	37177971	37182187	4216	1	chr22	37170004	37182450	12446	single	Variation_9847	chr22	37139431	37195809	56378	Wang et al. (2007)	Illumina HumanHap550 BeadChip	y
5928	chr22	39203535	39210542	7007	4	chr22	39203504	39210658	7154	single								
100970	chr22	39484232	39588504	104272	1	chr22	39484201	39590629	106428	single								
5929	chr22	39750326	39752972	2646	6	chr22	39750295	39753039	2744	HiConf								y

locus_id	chrom	start	end	length	Yoruba w/ event	putative chr	putative start	putative end	putative len	putative type	variation_id	DGV_chr	DGV_start	DGV_end	DGV_len	Reference	Method/platform	complex
100971	chr22	41199276	41308095	108819	22	chr22	41199201	41309549	110348	HiConf	Variation_7351	chr22	41201041	41300437	99396	de Smith et al. (2007)	Agilent 185k CGH Arrays/Agilent Custom CGH Arrays	
5932	chr22	41760012	41765745	5733	7	chr22	41759981	41765861	5880	HiConf								
100972	chr22	42000087	42010313	10226	45	chr22	41975949	42013091	37142	HiConf	Variation_43196	chr22	42006634	42007561	927	Wang et al. (2008)	Illumina DNA sequencing	
5934	chr22	42059206	42066703	7497	27	chr22	42059175	42066868	7693	HiConf								
5935	chr22	42155687	42169557	13870	5	chr22	42155656	42169817	14161	single								
5936	chr22	42306068	42307097	1029	17	chr22	42306037	42307115	1078	HiConf								
5937	chr22	42403284	42404999	1715	2	chr22	42403253	42405017	1764	single								
5938	chr22	42456351	42462819	6468	7	chr22	42456320	42462935	6615	HiConf								y
100974	chr22	42516076	42567838	51762	47	chr22	42503164	42577644	74480	single								
100975	chr22	43046906	43057833	10927	1	chr22	43004728	43082785	78057	HiConf								
5939	chr22	43308766	43312087	3321	13	chr22	43308724	43312154	3430	single								y
5940	chr22	43518097	43520680	2583	20	chr22	43517562	43520747	3185	single	Variation_7353	chr22	43514633	43526118	11485	de Smith et al. (2007)	Agilent 185k CGH Arrays/Agilent Custom CGH Arrays	
5941	chr22	43933358	43935367	2009	54	chr22	43933327	43935385	2058	HiConf	Variation_32475	chr22	43851470	43934563	83093	Perry et al. (2008)	Agilent Custom CGH Arrays	
5942	chr22	44913701	44916298	2597	4	chr22	44913670	44916365	2695	HiConf								
5943	chr22	45374301	45381219	6918	53	chr22	45374270	45381228	6958	HiConf	Variation_27073	chr22	45380573	45381058	485	Levy et al. (2007)	Sequencing	
5944	chr22	45651788	45657080	5292	14	chr22	45651757	45657196	5439	single								
5945	chr22	46152862	46159303	6441	2	chr22	46152831	46169785	16954	HiConf								
5946	chr22	46695880	46698477	2597	5	chr22	46695849	46698544	2695	single	Variation_5191	chr22	46649763	46893546	243783	Wong et al. (2007)	BAC Array CGH	
5947	chr22	46936911	46940292	3381	3	chr22	46936880	46940359	3479	single								
5948	chr22	47064778	47065159	381	40	chr22	47064770	47065162	392	single	Variation_27089	chr22	47064702	47064975	273	Levy et al. (2007)	Sequencing	
5949	chr22	47068133	47068340	207	8	chr22	47068102	47068347	245	single								
5950	chr22	47774566	47782896	8330	35	chr22	47774535	47783061	8526	HiConf	Variation_36036	chr22	47758216	47790090	31874	Kidd et al. (2008)	Paired End Mapping	
5951	chr22	48101863	48103258	1395	27	chr22	48101855	48103276	1421	single	Variation_39317	chr22	48101782	48103447	1665	Wheeler et al. (2008)	Sequencing	
5952	chr22	48799254	48806516	7262	55	chr22	48799223	48806622	7399	HiConf	Variation_27104	chr22	48801439	48801879	440	Levy et al. (2007)	Sequencing	
5953	chr22	49571298	49590506	19208	9	chr22	49571267	49590867	19600	HiConf								
1039	chr3	198751	207767	9016	28	chr3	198720	207932	9212	HiConf	Variation_38093	chr3	205022	207800	2778	McCarroll et al. (2008)	Affymetrix Human SNP Array 6.0	
1040	chr3	208035	209923	1888	31	chr3	208030	209941	1911	HiConf	Variation_31098	chr3	206383	209009	2626	Perry et al. (2008)	Agilent Custom CGH Arrays	
1041	chr3	288617	301308	12691	2	chr3	288586	301571	12985	HiConf	Variation_31099	chr3	289036	301050	12014	Perry et al. (2008)	Agilent Custom CGH Arrays	y
100173	chr3	588254	638488	50234	7	chr3	584007	639451	55444	HiConf	Variation_32480	chr3	587943	638149	50206	Perry et al. (2008)	Agilent Custom CGH Arrays	
1043	chr3	965846	971579	5733	4	chr3	965815	971695	5880	HiConf	Variation_7421	chr3	915435	1251928	336493	de Smith et al. (2007)	Agilent 185k CGH Arrays/Agilent Custom CGH Arrays	
1044	chr3	1007153	1014307	7154	5	chr3	1007122	1014423	7301	HiConf	Variation_38463	chr3	1009997	1014189	4192	McCarroll et al. (2008)	Affymetrix Human SNP Array 6.0	
1045	chr3	1019256	1021118	1862	5	chr3	1019225	1021136	1911	HiConf	Variation_32481	chr3	1008336	1020832	12496	Perry et al. (2008)	Agilent Custom CGH Arrays	
1046	chr3	1323889	1327682	3793	2	chr3	1323858	1327729	3871	HiConf	Variation_32482	chr3	1318446	1344365	25919	Perry et al. (2008)	Agilent Custom CGH Arrays	
1047	chr3	1340647	1341676	1029	33	chr3	1340616	1341694	1078	HiConf	Variation_44083	chr3	1340574	1341707	1133	Bentley et al. (2008)	Illumina DNA sequencing	
1048	chr3	1888516	1892534	4018	2	chr3	1888485	1892601	4116	HiConf	Variation_38485	chr3	1888098	1900851	12753	McCarroll et al. (2008)	Affymetrix Human SNP Array 6.0	
1049	chr3	1893224	1898022	4798	15	chr3	1893189	1898089	4900	HiConf	Variation_37418	chr3	1892992	1897569	4577	Cooper et al. (2008)	Illumina Human 1M BeadChip	
1050	chr3	1898490	1900668	2178	4	chr3	1898481	1900686	2205	single	Variation_38485	chr3	1888098	1900851	12753	McCarroll et al. (2008)	Affymetrix Human SNP Array 6.0	
1051	chr3	2003666	2003976	310	45	chr3	2003635	2003978	343	HiConf	Variation_5373	chr3	1917406	2649253	731847	Simon-Sanchez et al. (2007)	Illumina HumanHap300 BeadChip	
1052	chr3	2470859	2471322	463	36	chr3	2470850	2471340	490	single	Variation_27293	chr3	2470658	2470931	273	Levy et al. (2007)	Sequencing	
1053	chr3	2607248	2607934	686	4	chr3	2607217	2607952	735	single	Variation_8971	chr3	2187318	2877880	690562	Pinto et al. (2007)	Affymetrix 500K SNP Mapping Array	
1054	chr3	2926532	2926694	162	2	chr3	2926501	2926697	196	single	Variation_8407	chr3	2217441	3195570	978129	Pinto et al. (2007)	Affymetrix 500K SNP Mapping Array	
1055	chr3	2926875	2941232	14357	6	chr3	2926844	2941495	14651	single	Variation_8407	chr3	2217441	3195570	978129	Pinto et al. (2007)	Affymetrix 500K SNP Mapping Array	
1056	chr3	2989546	2990428	882	7	chr3	2989515	2990446	931	HiConf	Variation_8407	chr3	2217441	3195570	978129	Pinto et al. (2007)	Affymetrix 500K SNP Mapping Array	
1057	chr3	3456124	3456908	784	6	chr3	3456093	3456926	833	HiConf	Variation_8410	chr3	3398498	3687240	288742	Pinto et al. (2007)	Affymetrix 500K SNP Mapping Array	
1058	chr3	3563385	3568628	5243	7	chr3	3563354	3568744	5390	HiConf	Variation_44107	chr3	3563288	3568778	5490	Bentley et al. (2008)	Illumina DNA sequencing	
100174	chr3	4106036	4177674	71638	10	chr3	4106005	4179113	73108	HiConf	Variation_8976	chr3	4113369	4180623	67254	Pinto et al. (2007)	Affymetrix 500K SNP Mapping Array	
1060	chr3	5920726	5923719	2993	12	chr3	5920695	5923782	3087	HiConf								
1061	chr3	6191843	6214677	22834	7	chr3	6191812	6215136	23324	HiConf	Variation_32484	chr3	6194375	6215263	20888	Perry et al. (2008)	Agilent Custom CGH Arrays	
1062	chr3	6405264	6405543	279	48	chr3	6405256	6405550	294	HiConf	Variation_27347	chr3	6405197	6405485	288	Levy et al. (2007)	Sequencing	
1063	chr3	7375144	7377104	1960	48	chr3	7375113	7377122	2009	HiConf								
1064	chr3	7485002	7486325	1323	5	chr3	7484971	7486343	1372	single	Variation_9984	chr3	7484265	7486375	2110	Wang et al. (2007)	Illumina HumanHap550 BeadChip	
1065	chr3	7532519	7540568	8049	2	chr3	7531423	7540733	9310	HiConf	Variation_2440	chr3	7499552	7539303	39751	Redon et al. (2006)	Affymetrix 500K EA SNP Mapping Array	
1066	chr3	7686637	7686700	63	21	chr3	7686606	7686704	98	single	Variation_46444	chr3	7686553	7686760	207	Bentley et al. (2008)	Illumina DNA sequencing	
1067	chr3	8300730	8301636	906	3	chr3	8300723	8301654	931	single	Variation_23694	chr3	8300639	8301812	1173	Levy et al. (2007)	Sequencing	
1068	chr3	8428467	8429383	916	71	chr3	8428466	8429397	931	HiConf	Variation_27381	chr3	8428710	8429010	300	Levy et al. (2007)	Sequencing	
1069	chr3	9615032	9622921	7889	6	chr3	9615001	9623086	8085	single								
1070	chr3	10201366	10203767	2401	9	chr3	10201335	10203834	2499	HiConf	Variation_22725	chr3	10197769	10204815	7046	Korbel et al. (2007)	Paired End Mapping	
1071	chr3	10518739	10519139	400	11	chr3	10518708	10519149	441	single								
1072	chr3	10883734	10883968	234	1	chr3	10882533	10884493	1960	single								
1073	chr3	11083807	11088560	4753	10	chr3	11083776	11088627	4851	HiConf	Variation_36039	chr3	11071142	11088654	17512	Kidd et al. (2008)	Paired End Mapping	
1074	chr3	11385353	11389812	4459	6	chr3	11385322	11389879	4557	HiConf	Variation_10733	chr3	11387882	11388387	505	Conrad et al. (2005)	Mendelian inconsistencies	
1075	chr3	11615457	11628687	13230	14	chr3	11615426	11628950	13524	HiConf								
1076	chr3	11758243	11758880	637	40	chr3	11758212	11758898	686	HiConf	Variation_44080	chr3	11757214	11759338	2124	Bentley et al. (2008)	Illumina DNA sequencing	
1077	chr3	12640831	12641141	310	9	chr3	12640800	12641143	343	single	Variation_29726	chr3	12630689	12781123	150434	Jakobsson et al. (2008)	Illumina HumanHap550 BeadChip	
1078	chr3	13719425	13747258	27833	19	chr3	13719388	13753345	33957	HiConf								
1079	chr3	14266210	14272188	5978	6	chr3	14266179	14272304	6125	HiConf								
1080	chr3	14705201	14706083	882	4	chr3	14705170	14706101	931	single								
1081	chr3	15140587	15161146	20559	2	chr3	15140486	15161556	21070	HiConf								
1082	chr3	15819608	15823577	3969	35	chr3	15819577	15823644	4067	HiConf								
1083	chr3	16214475	16216067	1592	39	chr3	16214468	16216085	1617	HiConf	Variation_44090	chr3	16214413	16216211	1798	Bentley et al. (2008)	Illumina DNA sequencing	
1084	chr3	16585919	16594102	8183	43	chr3	16585888	16594267	8379	single								
1085	chr3	16623306	16629725	6419	9	chr3	16623275	16629841	6566	single								
100176	chr3	17036158	17104511	68353	26	chr3	17036125	17105901	69776	HiConf								
1087	chr3	17515106	17520545	5439	28	chr3	17515075	17520661	5586	HiConf	Variation_37925	chr3	17504952	17519017	14065	McCarroll et al. (2008)	Affymetrix Human SNP Array 6.0	
1088	chr3	17694789	17699885	5096	13	chr3	17694758	17700001	5243	HiConf								
1089	chr3	18448051	18454926	6875	11	chr3	18447986	18455042	7056	single								
1090	chr3	18921896	18928413	6517	26	chr3	18921865	18928529	6664	HiConf								y
100177	chr3	19161898	19175324	13426	1	chr3	19148441	19196412	47971	single								
1091	chr3	19291993	19294394	2401	4	chr3	19291962	19294412	2450	single								
1092	chr3	20349364	20351765	2401	3	chr3	20349333	20351832	2499	HiConf	Variation_3419	chr3	20240591	20515632	275041	Redon et al. (2006)	BAC Array CGH	
1093	chr3	21698726	21700980	2254	8	chr3	21698695	21700998	2303	HiConf								
1094	chr3	21781046	21784525	3479	15	chr3	21781015	21784592	3577	single	Variation_8418	chr3	21761897	22187361	425464	Pinto et al. (2007)	Affymetrix 500K SNP Mapping Array	
1095	chr3	22054711	22066912	12201	2	chr3	22054680	22067175	12495	HiConf	Variation_1616	chr3	22058240	22060813	2573	McCarroll et al. (2005)	Mendelian inconsistencies	
1096	chr3	22254484	22262030	7546	16	chr3	22254453	22262195	7742	HiConf	Variation_7405	chr3	22255614	22261196	5582	de Smith et al. (2007)	Agilent 185k CGH Arrays/Agilent Custom CGH Arrays	
1097	chr3	22882615	22885800	3185	7	chr3	22882584	22885867	3283	HiConf	Variation_44101	chr3	22882548	22885903	3355	Bentley et al. (2008)	Illumina DNA sequencing	
1098	chr3	23489088	23493547	4459	6	chr3	23489057	23493614	4557	single								

locus_id	chrom	start	end	length	Yoruba w/ event	putative chr	putative start	putative end	putative len	putative type	variation_id	DGV_chr	DGV_start	DGV_end	DGV_len	Reference	Method/platform	complex
100178	chr3	24487831	24553050	65219	2	chr3	24487800	24554366	66566	single								
1099	chr3	24756424	24760785	4361	11	chr3	24756393	24760852	4459	HiConf	Variation_44102	chr3	24755903	24760636	4733	Bentley et al. (2008)	Illumina DNA sequencing	
1100	chr3	24777984	24779209	1225	4	chr3	24777953	24779227	1274	HiConf	Variation_8979	chr3	24721300	24788964	67664	Pinto et al. (2007)	Affymetrix 500K SNP Mapping Array	
1101	chr3	25825114	25829851	4737	3	chr3	25825083	25829885	4802	HiConf	Variation_38488	chr3	25825784	25829901	4117	McCarroll et al. (2008)	Affymetrix Human SNP Array 6.0	
1102	chr3	25898173	25902828	4655	1	chr3	25898142	25902895	4753	single								
1103	chr3	26182177	26182863	686	8	chr3	26182146	26182881	735	single	Variation_3422	chr3	26040173	26194166	153993	Redon et al. (2006)	BAC Array CGH	
1104	chr3	26407822	26414290	6468	3	chr3	26407791	26414406	6615	single	Variation_38823	chr3	26409128	26414347	5219	McCarroll et al. (2008)	Affymetrix Human SNP Array 6.0	
1105	chr3	26426004	26427033	1029	2	chr3	26425970	26427048	1078	single	Variation_39328	chr3	26425973	26427302	1329	Wheeler et al. (2008)	Sequencing	
1106	chr3	26436585	26438006	1421	9	chr3	26436554	26438024	1470	HiConf								
1107	chr3	26930848	26933837	2989	6	chr3	26930817	26933904	3087	HiConf								
1108	chr3	27483470	27485283	1813	3	chr3	27483439	27485301	1862	single								
1109	chr3	28367136	28368067	931	16	chr3	28367105	28368085	980	HiConf	Variation_44105	chr3	28367093	28368316	1223	Bentley et al. (2008)	Illumina DNA sequencing	
1110	chr3	28612479	28614096	1617	2	chr3	28612448	28614114	1666	single								
1111	chr3	28614684	28619045	4361	2	chr3	28614653	28619112	4459	single								
1112	chr3	28645260	28646436	1176	11	chr3	28645229	28646454	1225	HiConf	Variation_8980	chr3	28635630	28680800	45170	Pinto et al. (2007)	Affymetrix 500K SNP Mapping Array	
1113	chr3	30042495	30046023	3528	8	chr3	30042464	30046090	3626	HiConf	Variation_38491	chr3	30042734	30046449	3715	McCarroll et al. (2008)	Affymetrix Human SNP Array 6.0	
1114						chr3	30840919	30840968	49	single								
1115	chr3	30885001	30895340	10339	5	chr3	30884970	30895554	10584	HiConf	Variation_38492	chr3	30886154	30888645	2491	McCarroll et al. (2008)	Affymetrix Human SNP Array 6.0	
1116	chr3	30969771	30974720	4949	14	chr3	30969740	30974836	5096	HiConf	Variation_38493	chr3	30967806	30973751	5945	McCarroll et al. (2008)	Affymetrix Human SNP Array 6.0	
1117	chr3	31084480	31084930	450	11	chr3	31084449	31084939	490	single								
1118	chr3	32077095	32082920	5825	26	chr3	32077091	32082922	5831	HiConf	Variation_23681	chr3	32077056	32082889	5833	Levy et al. (2007)	Sequencing	
1119	chr3	32162039	32191774	29735	22	chr3	32162008	32192143	30135	HiConf								
1120	chr3	32987395	32988718	1323	14	chr3	32987364	32988736	1372	single								
100180						chr3	33089872	33130983	41111	single								
1121	chr3	34094991	34095126	135	9	chr3	34094960	34095156	196	single								
1122	chr3	34221852	34225576	3724	41	chr3	34221821	34225643	3822	single								
1123	chr3	34239198	34239982	784	4	chr3	34239167	34240000	833	single								
1124	chr3	34810048	34810930	882	4	chr3	34810017	34810948	931	single								
1125	chr3	37415697	37416897	1200	2	chr3	37415641	37416915	1274	single								
1126	chr3	37428510	37430029	1519	20	chr3	37428479	37430047	1568	single	Variation_44108	chr3	37427869	37430589	2720	Bentley et al. (2008)	Illumina DNA sequencing	
1127	chr3	37953104	37961728	8624	4	chr3	37953073	37961893	8820	HiConf	Variation_32489	chr3	37954894	37961762	6868	Perry et al. (2008)	Agilent Custom CGH Arrays	y
1128	chr3	38088393	38106363	17970	25	chr3	38088362	38106884	18522	HiConf								
1129	chr3	38601913	38613722	11809	4	chr3	38601882	38613936	12054	single	Variation_36146	chr3	38587021	38616870	29849	Kidd et al. (2008)	Paired End Mapping	
1130	chr3	39000822	39002880	2058	32	chr3	39000791	39002898	2107	HiConf	Variation_3426	chr3	38908752	39071102	162350	Redon et al. (2006)	BAC Array CGH	
1131	chr3	39111212	39112360	1148	1	chr3	39108885	39117117	8232	single								
100181	chr3	39330642	39378123	47481	3	chr3	39307948	39395658	87710	HiConf								
100182	chr3	39783872	39832524	48652	2	chr3	39783787	39833522	49735	single								
1134	chr3	41737227	41737537	310	3	chr3	41737196	41737539	343	single	Variation_3427	chr3	41314156	41748757	434601	Redon et al. (2006)	BAC Array CGH	
1135	chr3	41754279	41764667	10388	6	chr3	41754248	41764881	10633	HiConf	Variation_38496	chr3	41755151	41763499	8348	McCarroll et al. (2008)	Affymetrix Human SNP Array 6.0	y
1136	chr3	41894712	41934795	40083	11	chr3	41894633	41935597	40964	HiConf								
1137	chr3	42333753	42348845	15092	2	chr3	42333722	42349157	15435	single	Variation_2449	chr3	42335110	42378384	43274	Redon et al. (2006)	Affymetrix 500K EA SNP Mapping Array	
1138	chr3	42868588	42868750	162	3	chr3	42868557	42868753	196	single								
1139	chr3	43028279	43034502	6223	29	chr3	43028248	43034618	6370	single								
100183	chr3	46757008	46845845	88837	34	chr3	46756977	46847627	90650	HiConf	Variation_36147	chr3	46753634	46836119	82485	Kidd et al. (2008)	Paired End Mapping	
1141	chr3	47465737	47468316	2579	48	chr3	47465737	47468383	2646	HiConf	Variation_43505	chr3	47465627	47468543	2916	Wang et al. (2008)	Illumina DNA sequencing	
100184	chr3	49697568	49720732	23164	13	chr3	49649716	49722163	72447	HiConf								
100185						chr3	50150031	50584073	434042	single								
100186	chr3	50814274	50830927	16653	4	chr3	50788623	50852715	64092	single								
1143	chr3	50922816	50925217	2401	3	chr3	50922785	50925235	2450	single								
1144	chr3	50927863	50929381	1518	1	chr3	50927832	50929498	1666	single								
1145						chr3	51696348	51698553	2205	single								
1146						chr3	52813989	52818203	4214	HiConf								
1147	chr3	53021878	53022564	686	15	chr3	53021847	53022582	735	HiConf	Variation_36155	chr3	52978699	53026103	47404	Kidd et al. (2008)	Paired End Mapping	
1148	chr3	53072446	53073867	1421	9	chr3	53072415	53073885	1470	single								
1149						chr3	53179382	53182322	2940	single								
1150	chr3	54216302	54216464	162	18	chr3	54216271	54216467	196	HiConf	Variation_46422	chr3	54216205	54216594	389	Bentley et al. (2008)	Illumina DNA sequencing	
1151	chr3	54613398	54616583	3185	1	chr3	54613367	54616650	3283	single	Variation_9995	chr3	54614249	54616608	2359	Wang et al. (2007)	Illumina HumanHap550 BeadChip	
1152	chr3	56598780	56608384	9604	7	chr3	56598749	56608549	9800	single								
1153	chr3	57447313	57452115	4802	7	chr3	57447282	57452182	4900	single								
1154	chr3	57603231	57608474	5243	17	chr3	57603200	57608590	5390	single								
1155	chr3	58773645	58775038	1393	5	chr3	58773614	58783659	10045	HiConf								
1157	chr3	58999241	59034472	35231	1	chr3	58999210	59035176	35966	single								
1158	chr3	59674559	59675343	784	26	chr3	59674528	59675361	833	HiConf								
1159	chr3	60052888	60053427	539	3	chr3	60052857	60053445	588	single	Variation_8983	chr3	60042436	60263468	221032	Pinto et al. (2007)	Affymetrix 500K SNP Mapping Array	
1160	chr3	60316018	60318419	2401	1	chr3	60315987	60318437	2450	single								
100188	chr3	60506253	60530674	24421	7	chr3	60484694	60531514	46820	single								
1161	chr3	60753735	60753995	260	4	chr3	60753704	60753998	294	single								
100189	chr3	60808215	60817215	9000	1	chr3	60799544	60817551	18007	single	Variation_1367	chr3	60801472	60878151	76679	Conrad et al. (2005)	Mendelian inconsistencies	
1163	chr3	62585992	62587070	1078	17	chr3	62585961	62587088	1127	HiConf	Variation_9998	chr3	62585665	62587053	1388	Wang et al. (2007)	Illumina HumanHap550 BeadChip	
100190	chr3	62638226	62671252	33026	4	chr3	62638195	62671907	33712	HiConf	Variation_22906	chr3	62639373	62671804	32431	Korbel et al. (2007)	Paired End Mapping	
1165	chr3	62686393	62690803	4410	11	chr3	62686362	62690870	4508	HiConf	Variation_32492	chr3	62687200	62690477	3277	Perry et al. (2008)	Agilent Custom CGH Arrays	
1166	chr3	63079373	63079773	400	3	chr3	63079342	63079783	441	single								
1167	chr3	63103138	63113379	10241	8	chr3	63103107	63113593	10486	HiConf	Variation_38900	chr3	63108165	63110809	2644	McCarroll et al. (2008)	Affymetrix Human SNP Array 6.0	
1168	chr3	63711718	63713335	1617	1	chr3	63711687	63713353	1666	single								
1169	chr3	64508507	64510222	1715	11	chr3	64508476	64510240	1764	HiConf								
1170	chr3	65092881	65094645	1764	5	chr3	65092850	65094663	1813	HiConf								
100191						chr3	65298552	65355980	57428	single								
1172	chr3	67579043	67579250	207	56	chr3	67579012	67579257	245	HiConf	Variation_44114	chr3	67576387	67579483	3096	Bentley et al. (2008)	Illumina DNA sequencing	
1173	chr3	68718489	68724026	5537	64	chr3	68718458	68724142	5684	HiConf	Variation_22714	chr3	68716816	68723738	6922	Korbel et al. (2007)	Paired End Mapping	
1174	chr3	68822125	68830398	8273	32	chr3	68819104	68830570	11466	HiConf	Variation_44116	chr3	68822299	68830548	8249	Bentley et al. (2008)	Illumina DNA sequencing	
1175	chr3	68979610	69008079	28469	4	chr3	68979579	69008636	29057	HiConf	Variation_2461	chr3	68971420	69018486	47066	Redon et al. (2006)	Affymetrix 500K EA SNP Mapping Array	
1176	chr3	69262586	69264104	1518	25	chr3	69261819	69264122	2303	HiConf								
1177	chr3	69400275	69400635	360	7	chr3	69400244	69400636	392	single								
1178	chr3	70142478	70147231	4753	2	chr3	70142447	70147298	4851	HiConf								
100193	chr3	71560636	71591555	30919	14	chr3	71560605	71592186	31581	HiConf								
1180	chr3	72863056	72868691	5635	3	chr3	72863025	72868807	5782	HiConf								

locus_id	chrom	start	end	length	Yoruba w/ event	putative chr	putative start	putative end	putative len	putative type	variation_id	DGV_chr	DGV_start	DGV_end	DGV_len	Reference	Method/platform	complex
1181	chr3	73145982	73146045	63	57	chr3	73145951	73146049	98	HiConf								
1182	chr3	74021857	74022019	162	20	chr3	74021826	74022022	196	single								
1183	chr3	74230009	74237604	7595	12	chr3	74229978	74237769	7791	HiConf	Variation_22767	chr3	74229997	74237786	7789	Korbel et al. (2007)	Paired End Mapping	
1184	chr3	74485740	74489709	3969	5	chr3	74485709	74489776	4067	HiConf								
1185	chr3	74891803	74895716	3913	5	chr3	74891772	74896329	4557	HiConf								
1186	chr3	75676244	75684084	7840	5	chr3	75676213	75684249	8036	HiConf	Variation_4341	chr3	75640941	75700637	59696	Wong et al. (2007)	BAC Array CGH	
1187	chr3	75707310	75741975	34665	12	chr3	75707279	75742510	35231	single	Variation_31108	chr3	75712495	75714604	2109	Perry et al. (2008)	Agilent Custom CGH Arrays	
1188	chr3	75782378	75798989	16611	18	chr3	75782347	75799301	16954	HiConf	Variation_32497	chr3	75745040	75900650	155610	Perry et al. (2008)	Agilent Custom CGH Arrays	
1189	chr3	75841815	75876765	34950	18	chr3	75841784	75877456	35672	HiConf	Variation_31109	chr3	75845474	75876105	30631	Perry et al. (2008)	Agilent Custom CGH Arrays	
1190	chr3	76166538	76178106	11568	1	chr3	76166507	76178561	12054	single	Variation_10003	chr3	76159833	76176911	17078	Wang et al. (2007)	Illumina HumanHap550 BeadChip	
1191	chr3	76273113	76274044	931	2	chr3	76273082	76274062	980	single	Variation_32499	chr3	76273485	76274835	1350	Perry et al. (2008)	Agilent Custom CGH Arrays	
1192	chr3	77428337	77433090	4753	3	chr3	77428306	77433157	4851	single								
1193	chr3	77998354	78001000	2646	2	chr3	77998323	78001067	2744	HiConf								
1194	chr3	78425487	78425694	207	11	chr3	78425456	78425701	245	single								
1195	chr3	78454201	78454601	400	14	chr3	78454170	78454611	441	HiConf								
1196	chr3	78543187	78558963	15776	5	chr3	78543154	78559275	16121	HiConf	Variation_2463	chr3	78536627	78579808	43181	Redon et al. (2006)	Affymetrix 500K EA SNP Mapping Array	
1197	chr3	78671075	78674113	3038	4	chr3	78671044	78674180	3136	HiConf								
1198	chr3	79299108	79306452	7344	6	chr3	79299077	79306770	7693	single	Variation_39337	chr3	79292081	79314382	22301	Wheeler et al. (2008)	Sequencing	
1199	chr3	79804249	79809363	5114	3	chr3	79804218	79809510	5292	HiConf	Variation_5368	chr3	79366217	81386046	2019829	Simon-Sanchez et al. (2007)	Illumina HumanHap300 BeadChip	
1200	chr3	80145730	80147102	1372	73	chr3	80145699	80147120	1421	HiConf	Variation_5368	chr3	79366217	81386046	2019829	Simon-Sanchez et al. (2007)	Illumina HumanHap300 BeadChip	
1201	chr3	80484761	80487897	3136	2	chr3	80484730	80487964	3234	HiConf	Variation_5368	chr3	79366217	81386046	2019829	Simon-Sanchez et al. (2007)	Illumina HumanHap300 BeadChip	
1202	chr3	81009404	81023075	13671	2	chr3	81009373	81023338	13965	single	Variation_5368	chr3	79366217	81386046	2019829	Simon-Sanchez et al. (2007)	Illumina HumanHap300 BeadChip	
100195						chr3	81150346	81213826	63480	single								
1203	chr3	82227544	82246752	19208	7	chr3	82227513	82247113	19600	single								
1204	chr3	82947501	82955292	7791	22	chr3	82947470	82955457	7987	HiConf	Variation_32500	chr3	82951723	82955245	3522	Perry et al. (2008)	Agilent Custom CGH Arrays	
1205	chr3	82992042	82993365	1323	7	chr3	82992011	82993383	1372	HiConf								
1206	chr3	83099940	83102684	2744	10	chr3	83099909	83102751	2842	HiConf								
1207	chr3	83633599	83648789	15190	6	chr3	83633568	83649101	15533	HiConf								
1208	chr3	83943132	83946317	3185	21	chr3	83943101	83946384	3283	HiConf								
1209	chr3	83974835	83976942	2107	3	chr3	83974804	83976960	2156	single								
1210	chr3	84309015	84315826	6811	3	chr3	84308984	84315942	6958	single								
1211	chr3	84554113	84559797	5684	16	chr3	84554082	84559913	5831	HiConf								
1212	chr3	84782747	84784952	2205	13	chr3	84782716	84784970	2254	HiConf	Variation_38024	chr3	84782483	84784826	2343	McCarroll et al. (2008)	Affymetrix Human SNP Array 6.0	
100196	chr3	85035365	85052404	17039	33	chr3	85024850	85105234	80384	single	Variation_46455	chr3	85040717	85040934	217	Bentley et al. (2008)	Illumina DNA sequencing	
1214	chr3	85294549	85295875	1326	4	chr3	85294178	85295893	1715	HiConf								
1215	chr3	86144261	86146760	2499	3	chr3	86144230	86146827	2597	single								
100197	chr3	86847722	86908710	60988	2	chr3	86847674	86909953	62279	single								
1216	chr3	86931005	86937600	6595	4	chr3	86930974	86937736	6762	HiConf								
1217	chr3	87388371	87389057	686	3	chr3	87388340	87389075	735	single								
1218						chr3	87434988	87435135	147	single								
100198	chr3	87907428	87944129	36701	4	chr3	87907397	87944858	37461	single								
1220	chr3	87973186	87984750	11564	4	chr3	87973155	87984964	11809	HiConf	Variation_38506	chr3	87974966	87984943	9977	McCarroll et al. (2008)	Affymetrix Human SNP Array 6.0	
1221	chr3	88540214	88547515	7301	1	chr3	88540183	88547680	7497	single								
1222	chr3	89109888	89112338	2450	2	chr3	89109857	89112405	2548	HiConf								
1223	chr3	89753038	89760608	7570	3	chr3	89753031	89760773	7742	HiConf	Variation_32502	chr3	89752823	89760716	7893	Perry et al. (2008)	Agilent Custom CGH Arrays	
1224	chr3	95777024	95781042	4018	9	chr3	95776993	95781109	4116	HiConf								
1225	chr3	95995074	95996005	931	1	chr3	95995043	95996023	980	single								
1226	chr3	96629918	96631633	1715	4	chr3	96629887	96631651	1764	HiConf								
1227	chr3	96949790	96950721	931	79	chr3	96949759	96950739	980	HiConf								y
1228	chr3	97819344	97819883	539	49	chr3	97819313	97819901	588	single								
1229	chr3	99389255	99391117	1862	17	chr3	99389224	99391135	1911	HiConf	Variation_37833	chr3	99370181	99402053	31872	McCarroll et al. (2008)	Affymetrix Human SNP Array 6.0	
1230	chr3	99893024	99897287	4263	10	chr3	99892993	99897354	4361	HiConf	Variation_7423	chr3	99893514	99897268	3754	de Smith et al. (2007)	Agilent 185k CGH Arrays/Agilent Custom CGH Arrays	y
1231	chr3	100379545	100385580	6035	69	chr3	100379514	100385590	6076	HiConf	Variation_43533	chr3	100381723	100385131	3408	Wang et al. (2008)	Illumina DNA sequencing	
1232	chr3	100725142	100727543	2401	21	chr3	100725111	100727561	2450	HiConf	Variation_44077	chr3	100725953	100727568	1615	Bentley et al. (2008)	Illumina DNA sequencing	
1233	chr3	101111533	101112242	709	12	chr3	101111525	101112260	735	HiConf	Variation_40544	chr3	101111472	101112321	849	Wheeler et al. (2008)	Sequencing	
1234	chr3	101405556	101407418	1862	27	chr3	101405525	101407436	1911	HiConf	Variation_43442	chr3	101405315	101407680	2365	Wang et al. (2008)	Illumina DNA sequencing	
1235	chr3	102151973	102153492	1519	9	chr3	102151942	102153510	1568	HiConf	Variation_44078	chr3	102151997	102153549	1552	Bentley et al. (2008)	Illumina DNA sequencing	
1236	chr3	102734485	102736790	2305	6	chr3	102734454	102736806	2352	HiConf								
1237	chr3	102839590	102842236	2646	10	chr3	102839559	102842303	2744	HiConf	Variation_43444	chr3	102840220	102842592	2372	Wang et al. (2008)	Illumina DNA sequencing	
1238	chr3	104290480	104292685	2205	5	chr3	104290449	104292703	2254	single								
100199	chr3	104409065	104469015	59950	10	chr3	104408956	104470083	61127	single	Variation_2465	chr3	104428032	104433385	5353	Redon et al. (2006)	Affymetrix 500K EA SNP Mapping Array	
1239	chr3	105405671	105407876	2205	7	chr3	105405640	105407894	2254	HiConf	Variation_31112	chr3	105296348	105516822	220474	Perry et al. (2008)	Agilent Custom CGH Arrays	
1240	chr3	106073639	106080303	6664	3	chr3	106073608	106080419	6811	single	Variation_8988	chr3	105586566	106261000	674434	Pinto et al. (2007)	Affymetrix 500K SNP Mapping Array	
1241	chr3	107061577	107069221	7644	2	chr3	107061546	107069386	7840	single								y
1242	chr3	108520552	108523541	2989	60	chr3	108520521	108523608	3087	HiConf	Variation_43468	chr3	108520599	108523158	2559	Wang et al. (2008)	Illumina DNA sequencing	
1243	chr3	108742081	108750607	8526	2	chr3	108742050	108750772	8722	HiConf								
1244	chr3	108900302	108901870	1568	4	chr3	108900271	108901888	1617	HiConf	Variation_4349	chr3	108852877	109039033	186156	Wong et al. (2007)	BAC Array CGH	
1245	chr3	109884516	109884723	207	25	chr3	109884485	109884730	245	HiConf								
1246	chr3	110066933	110069001	2068	2	chr3	110066667	110069019	2352	single								y
1247	chr3	110586882	110587421	539	34	chr3	110586851	110587439	588	single								
1248	chr3	110897934	110898767	833	4	chr3	110897903	110898785	882	single								
1249	chr3	112176981	112181979	4998	6	chr3	112176950	112182095	5145	HiConf								
1250	chr3	112727033	112729260	2227	15	chr3	112727024	112729278	2254	HiConf	Variation_44079	chr3	112726289	112731693	5404	Bentley et al. (2008)	Illumina DNA sequencing	
1251	chr3	113936081	113943088	7007	3	chr3	113936050	113943204	7154	single								
1252	chr3	114210971	114235531	24560	20	chr3	114210940	114235538	24598	single	Variation_23660	chr3	114226773	114234972	8199	Levy et al. (2007)	Sequencing	
1253	chr3	116140634	116152694	12060	53	chr3	116136248	116153006	16758	HiConf	Variation_22859	chr3	116139177	116152319	13142	Korbel et al. (2007)	Paired End Mapping	
1254	chr3	116826640	116830658	4018	1	chr3	116826609	116830725	4116	single								
1255	chr3	117581730	117592510	10780	2	chr3	117581699	117592724	11025	HiConf	Variation_1059	chr3	117585789	117591189	5400	Conrad et al. (2005)	Mendelian inconsistencies	
1256	chr3	117687325	117700080	12755	8	chr3	117687294	117700328	13034	HiConf	Variation_44081	chr3	117687148	117701012	13864	Bentley et al. (2008)	Illumina DNA sequencing	
1257	chr3	118006074	118008520	2446	6	chr3	118005990	118008587	2597	HiConf	Variation_10734	chr3	118007437	118008279	842	Conrad et al. (2005)	Mendelian inconsistencies	
1258						chr3	118047542	118053569	6027	single								
1259	chr3	121643732	121647750	4018	56	chr3	121643701	121647817	4116	HiConf	Variation_22594	chr3	121643307	121649129	5822	Korbel et al. (2007)	Paired End Mapping	
1260	chr3	122574585	122578088	3503	22	chr3	122574554	122580238	5684	single								
1261	chr3	122976581	123001522	24941	11	chr3	122976550	123002030	25480	single	Variation_7357	chr3	122983090	123574521	591431	de Smith et al. (2007)	Agilent 185k CGH Arrays/Agilent Custom CGH Arrays	
100200	chr3	123378532	123393333	14801	1	chr3	123348901	123422916	74015	HiConf	Variation_7357	chr3	122983090	123574521	591431	de Smith et al. (2007)	Agilent 185k CGH Arrays/Agilent Custom CGH Arrays	
100201	chr3	123920934	124002666	81732	6	chr3	123920903	124004325	83422	single								

locus_id	chrom	start	end	length	Yoruba w/ event	putative chr	putative start	putative end	putative len	putative type	variation_id	DGV_chr	DGV_start	DGV_end	DGV_len	Reference	Method/platform	complex
1263	chr3	124798205	124799332	1127	2	chr3	124798174	124799350	1176	single								
1264	chr3	125830831	125831664	833	14	chr3	125830800	125831682	882	single								
1265	chr3	126419151	126419574	423	38	chr3	126419143	126419584	441	HiConf	Variation_12329	chr3	126418986	126419853	867	Mills et al. (2006)	Sequence trace read mapping	
100202	chr3	126512691	126570168	57477	5	chr3	126512660	126571435	58775	HiConf	Variation_31116	chr3	126450312	126834131	383819	Perry et al. (2008)	Agilent Custom CGH Arrays	
1266	chr3	126899404	127127875	228471	12	chr3	126899343	127131064	231721	HiConf	Variation_3446	chr3	127024023	127185446	161423	Redon et al. (2006)	BAC Array CGH	y
1267	chr3	127155350	127160152	4802	34	chr3	127155319	127160219	4900	HiConf	Variation_32512	chr3	127153912	127160209	6297	Perry et al. (2008)	Agilent Custom CGH Arrays	
1268	chr3	127195108	127204546	9438	30	chr3	127195107	127204711	9604	HiConf	Variation_37964	chr3	127194960	127204552	9592	McCarroll et al. (2008)	Affymetrix Human SNP Array 6.0	
100204						chr3	128165626	128201102	35476	single								
1270	chr3	128363445	128364033	588	33	chr3	128363414	128364051	637	single								
1271	chr3	128674281	128675344	1063	1	chr3	128673388	128682159	8771	single								
1272	chr3	128761913	128762173	260	15	chr3	128761882	128762176	294	single								
1273	chr3	129012499	129012759	260	58	chr3	129012468	129012762	294	HiConf								
1274	chr3	129912629	129915422	2793	4	chr3	129912598	129915489	2891	single	Variation_1374	chr3	129913631	129917110	3479	Conrad et al. (2005)	Mendelian inconsistencies	
1275	chr3	130558939	130562467	3528	19	chr3	130558908	130562534	3626	HiConf	Variation_36058	chr3	130523337	130568598	45261	Kidd et al. (2008)	Paired End Mapping	y
1276	chr3	131140079	131140529	450	60	chr3	131140048	131140538	490	HiConf	Variation_46290	chr3	131139997	131140629	632	Bentley et al. (2008)	Illumina DNA sequencing	
1277	chr3	131200496	131294619	94123	3	chr3	131200465	131296505	96040	HiConf	Variation_36060	chr3	131224293	131294091	69798	Kidd et al. (2008)	Paired End Mapping	
1278	chr3	131298297	131412931	114634	11	chr3	131298171	131413272	115101	HiConf	Variation_37603	chr3	131260491	131392498	132007	Cooper et al. (2008)	Illumina Human 1M BeadChip	
1279	chr3	131524827	131528502	3675	2	chr3	131524796	131528569	3773	single								
1280	chr3	131819660	131830540	10880	42	chr3	131819629	131835652	16023	HiConf	Variation_44082	chr3	131830273	131835797	5524	Bentley et al. (2008)	Illumina DNA sequencing	y
1281	chr3	132081418	132089503	8085	5	chr3	132081387	132089668	8281	single								
1282	chr3	132089944	132111210	21266	2	chr3	132089913	132111620	21707	single								
1283	chr3	132369685	132372380	2695	2	chr3	132369654	132372447	2793	HiConf								
1284	chr3	133472370	133480569	8199	21	chr3	133471615	133480680	9065	HiConf	Variation_22717	chr3	133471299	133478251	6952	Korbel et al. (2007)	Paired End Mapping	
1285	chr3	133587449	133598801	11352	2	chr3	133571232	133599358	28126	HiConf								
1286	chr3	133765058	133784476	19418	1	chr3	133765027	133790605	25578	HiConf								
1287	chr3	133932099	133935039	2940	2	chr3	133932068	133935106	3038	single								
1288	chr3	134080618	134081353	735	10	chr3	134080587	134081371	784	HiConf								
1289	chr3	134291367	134303862	12495	1	chr3	134291336	134304125	12789	single								
1290	chr3	134498931	134507408	8477	3	chr3	134498900	134507573	8673	HiConf	Variation_36069	chr3	134498153	134515167	17014	Kidd et al. (2008)	Paired End Mapping	
1291	chr3	134615453	134619569	4116	28	chr3	134615422	134619636	4214	HiConf								y
1292	chr3	134621137	134642991	21854	26	chr3	134621106	134643450	22344	HiConf								
1293	chr3	135078797	135083305	4508	1	chr3	135078766	135083372	4606	single								
1294	chr3	135874459	135875145	686	3	chr3	135874428	135875163	735	single								
1295	chr3	136324783	136326925	2142	11	chr3	136323807	136326992	3185	HiConf	Variation_37881	chr3	136325315	136327533	2218	McCarroll et al. (2008)	Affymetrix Human SNP Array 6.0	
1296	chr3	136498180	136498387	207	12	chr3	136498149	136498394	245	single								
1297	chr3	137179917	137187806	7889	10	chr3	137179886	137187971	8085	HiConf								
1298	chr3	137504003	137508756	4753	4	chr3	137503972	137508823	4851	HiConf	Variation_36071	chr3	137500650	137518869	18219	Kidd et al. (2008)	Paired End Mapping	
1299	chr3	138513184	138526584	13400	7	chr3	138513176	138526847	13671	HiConf	Variation_38469	chr3	138510648	138525910	15262	McCarroll et al. (2008)	Affymetrix Human SNP Array 6.0	y
1300	chr3	139245267	139262875	17608	32	chr3	139245236	139263072	17836	HiConf	Variation_27167	chr3	139252447	139252652	205	Levy et al. (2007)	Sequencing	
100207	chr3	140290486	140333461	42975	7	chr3	140290455	140334335	43880	single								
1301	chr3	141750343	141753045	2702	19	chr3	141750312	141753056	2744	single	Variation_32520	chr3	141750620	141753174	2554	Perry et al. (2008)	Agilent Custom CGH Arrays	
1302	chr3	142027144	142029643	2499	70	chr3	142027113	142029710	2597	HiConf	Variation_44085	chr3	142027100	142029734	2634	Bentley et al. (2008)	Illumina DNA sequencing	
1303	chr3	143332700	143334513	1813	41	chr3	143332669	143334531	1862	HiConf	Variation_2476	chr3	143239231	143577321	338090	Redon et al. (2006)	Affymetrix 500K EA SNP Mapping Array	
1304						chr3	143651022	143652835	1813	single								
1305	chr3	143927560	143928785	1225	2	chr3	143927529	143928803	1274	single								
1306	chr3	144702465	144703818	1353	16	chr3	144702464	144703836	1372	HiConf	Variation_7369	chr3	144702354	144703946	1592	de Smith et al. (2007)	Agilent 185k CGH Arrays/Agilent Custom CGH Arrays	
1307	chr3	145764080	145775938	11858	10	chr3	145764049	145776152	12103	HiConf	Variation_22851	chr3	145763524	145775376	11852	Korbel et al. (2007)	Paired End Mapping	
1308	chr3	146472522	146485556	13034	15	chr3	146472491	146489690	17199	HiConf	Variation_36077	chr3	146468317	146482511	14194	Kidd et al. (2008)	Paired End Mapping	y
1309	chr3	146629322	146629529	207	26	chr3	146629291	146629536	245	HiConf								
1310	chr3	146962032	146962342	310	77	chr3	146962001	146962344	343	HiConf	Variation_8992	chr3	146955710	147240884	285174	Pinto et al. (2007)	Affymetrix 500K SNP Mapping Array	
100208	chr3	147107146	147144253	37107	3	chr3	147107115	147144967	37852	HiConf	Variation_10010	chr3	147109058	147143052	33994	Wang et al. (2007)	Illumina HumanHap550 BeadChip	
1312	chr3	147426797	147451495	24698	8	chr3	147426766	147451903	25137	single								
1313	chr3	147865690	147866817	1127	6	chr3	147865659	147866835	1176	single	Variation_23172	chr3	147864911	147873843	8932	Korbel et al. (2007)	Paired End Mapping	y
1314	chr3	147867552	147872958	5406	51	chr3	147867521	147873058	5537	HiConf	Variation_22616	chr3	147867331	147873340	6009	Korbel et al. (2007)	Paired End Mapping	
1315	chr3	147898275	147899990	1715	10	chr3	147898244	147900008	1764	HiConf								
100209	chr3	147947324	148013817	66493	3	chr3	147947293	148015183	67890	single								
1316	chr3	149075990	149113922	37932	45	chr3	149075959	149114424	38465	HiConf								
1317	chr3	149319765	149322509	2744	22	chr3	149319734	149322576	2842	HiConf								
1318	chr3	150751202	150752721	1519	23	chr3	150751171	150752739	1568	HiConf	Variation_44087	chr3	150751166	150752835	1669	Bentley et al. (2008)	Illumina DNA sequencing	y
1319	chr3	151869039	151869529	490	1	chr3	151869008	151869547	539	single								
1320	chr3	153253730	153258238	4508	1	chr3	153253699	153258305	4606	single								
1321	chr3	153698650	153709479	10829	6	chr3	153698619	153709693	11074	HiConf								
1322	chr3	154361130	154363384	2254	19	chr3	154361099	154363402	2303	single								
1323	chr3	154947807	154952756	4949	9	chr3	154947776	154952872	5096	HiConf								
1324	chr3	155229361	155230537	1176	5	chr3	155229330	155230555	1225	single								
100210	chr3	155771228	155926411	155183	2	chr3	155771197	155929565	158368	HiConf	Variation_3458	chr3	155740963	156041417	300454	Redon et al. (2006)	BAC Array CGH	
1326	chr3	156978318	156981258	2940	1	chr3	156978287	156981325	3038	single	Variation_7374	chr3	156963914	156981135	17221	de Smith et al. (2007)	Agilent 185k CGH Arrays/Agilent Custom CGH Arrays	
1327	chr3	157239880	157241497	1617	16	chr3	157239849	157241515	1666	HiConf								
1328	chr3	157574893	157576853	1960	53	chr3	157574862	157576871	2009	HiConf	Variation_6218	chr3	157574869	157576385	1516	Mills et al. (2006)	Sequence trace read mapping	
1329	chr3	158016040	158020891	4851	15	chr3	158016009	158021007	4998	single								
1330	chr3	158822158	158825128	2970	4	chr3	158822157	158825195	3038	HiConf	Variation_38473	chr3	158822082	158824376	2294	McCarroll et al. (2008)	Affymetrix Human SNP Array 6.0	
1331	chr3	159769897	159773278	3381	45	chr3	159769866	159773345	3479	HiConf								
1332	chr3	160291012	160295830	4818	13	chr3	160290981	160301957	10976	HiConf								
1333	chr3	160381172	160382495	1323	5	chr3	160381141	160382513	1372	HiConf								
100212	chr3	160744462	160790776	46314	2	chr3	160736710	160791908	55198	HiConf								
1336	chr3	163400650	163402806	2156	17	chr3	163400619	163402824	2205	HiConf	Variation_34430	chr3	163345992	163454992	109000	Zogopoulos et al. (2007)	Affymetrix 500K and 100K SNP Mapping Arrays	y
100213	chr3	163988437	164109399	120962	50	chr3	163985312	164135619	150307	HiConf	Variation_43698	chr3	163994788	164109092	114304	Wang et al. (2008)	Illumina DNA sequencing	
1338	chr3	164174605	164206945	32340	43	chr3	164174574	164207600	33026	HiConf	Variation_36097	chr3	164173810	164220330	46520	Kidd et al. (2008)	Paired End Mapping	
1339	chr3	164247370	164251682	4312	58	chr3	164247339	164251749	4410	HiConf	Variation_44091	chr3	164247596	164251780	4184	Bentley et al. (2008)	Illumina DNA sequencing	
1340	chr3	165785088	165794447	9359	7	chr3	165785057	165794612	9555	HiConf	Variation_36102	chr3	165783447	165811717	28270	Kidd et al. (2008)	Paired End Mapping	
1341	chr3	166702564	166706435	3871	2	chr3	166702533	166706502	3969	single								
100214	chr3	166743210	166788437	45227	10	chr3	166743179	166789337	46158	HiConf	Variation_10022	chr3	166747883	166797069	49186	Wang et al. (2007)	Illumina HumanHap550 BeadChip	
1343	chr3	167568749	167579429	10680	17	chr3	167568706	167580858	12152	HiConf	Variation_37724	chr3	167573295	167576883	3588	McCarroll et al. (2008)	Affymetrix Human SNP Array 6.0	
1344	chr3	168012138	168018116	5978	11	chr3	168012107	168018232	6125	single	Variation_9459	chr3	168005541	168016121	10580	Wang et al. (2007)	Illumina HumanHap550 BeadChip	
1345	chr3	168032571	168040068	7497	4	chr3	168032540	168040233	7693	single								
1346	chr3	168327124	168341369	14245	36	chr3	168327079	168341632	14553	HiConf								

locus_id	chrom	start	end	length	Yoruba w/ event	putative chr	putative start	putative end	putative len	putative type	variation_id	DGV_chr	DGV_start	DGV_end	DGV_len	Reference	Method/platform	complex
1347	chr3	169911672	169915200	3528	2	chr3	169911641	169915267	3626	HiConf	Variation_3463	chr3	169771708	169985804	214096	Redon et al. (2006)	BAC Array CGH	
1348	chr3	170221254	170221416	162	35	chr3	170221223	170221419	196	HiConf	Variation_43025	chr3	170221065	170221706	641	Wang et al. (2008)	Illumina DNA sequencing	
1349	chr3	170753394	170758588	5194	5	chr3	170753363	170758704	5341	single								
1350	chr3	171545675	171546165	490	33	chr3	171545644	171546183	539	single								
1351	chr3	174143116	174143704	588	17	chr3	174143085	174143722	637	single								
1352	chr3	174589604	174600188	10584	4	chr3	174589573	174600402	10829	single								
1353	chr3	174691876	174698383	6507	8	chr3	174689435	174701489	12054	HiConf	Variation_38480	chr3	174692475	174698428	5953	McCarroll et al. (2008)	Affymetrix Human SNP Array 6.0	
1354	chr3	175559804	175563141	3337	6	chr3	175559773	175563203	3430	HiConf	Variation_44092	chr3	175559773	175563332	3559	Bentley et al. (2008)	Illumina DNA sequencing	
1355	chr3	176560531	176566068	5537	16	chr3	176560500	176566184	5684	HiConf	Variation_32536	chr3	176563646	176565983	2337	Perry et al. (2008)	Agilent Custom CGH Arrays	
100215	chr3	177374595	177432609	58014	4	chr3	177374562	177433803	59241	HiConf	Variation_1654	chr3	177379272	177412368	33096	McCarroll et al. (2005)	Null genotypes	
1358	chr3	178175032	178192247	17215	3	chr3	178175001	178192739	17738	HiConf	Variation_38482	chr3	178176283	178191496	15213	McCarroll et al. (2008)	Affymetrix Human SNP Array 6.0	
1359	chr3	178866030	178866237	207	21	chr3	178865999	178866244	245	HiConf	Variation_7388	chr3	178866164	178872551	6387	de Smith et al. (2007)	Agilent 185k CGH Arrays/Agilent Custom CGH Arrays	
1360	chr3	179237254	179239655	2401	1	chr3	179237223	179239673	2450	single	Variation_36109	chr3	179231611	179244210	12599	Kidd et al. (2008)	Paired End Mapping	
1361	chr3	179262930	179264008	1078	10	chr3	179262899	179264026	1127	HiConf								
100216	chr3	179319991	179360495	40504	13	chr3	179319960	179361316	41356	HiConf								
100217	chr3	180239405	180364205	124800	2	chr3	180211294	180367310	156016	single								
1363	chr3	180719798	180726090	6292	17	chr3	180719767	180731870	12103	single								
1364	chr3	181032908	181033398	490	18	chr3	181032877	181033416	539	HiConf								
1365	chr3	181136788	181136896	108	3	chr3	181136757	181136904	147	single								
1366	chr3	181137066	181137442	376	54	chr3	181137051	181137443	392	HiConf	Variation_46339	chr3	181137037	181137496	459	Bentley et al. (2008)	Illumina DNA sequencing	
1367	chr3	181244539	181251105	6566	4	chr3	181244508	181251221	6713	single								
1368	chr3	181251399	181251654	255	1	chr3	181251368	181253965	2597	single								
1369	chr3	182228825	182235943	7118	1	chr3	182228673	182247734	19061	single								
100218	chr3	182880505	182887310	6805	1	chr3	182868589	182935376	66787	single								
1370	chr3	183123836	183125396	1560	2	chr3	183123805	183129832	6027	HiConf								
1371	chr3	183209194	183213856	4662	1	chr3	183209163	183213916	4753	single								
1372	chr3	183550430	183550969	539	9	chr3	183550399	183550987	588	single								
1373	chr3	185436244	185436352	108	35	chr3	185436213	185436360	147	single	Variation_4364	chr3	185337138	185502784	165646	Wong et al. (2007)	BAC Array CGH	
100219	chr3	185715016	185759571	44555	17	chr3	185714925	185760520	45595	HiConf	Variation_4365	chr3	185604555	185822597	218042	Wong et al. (2007)	BAC Array CGH	
1375	chr3	185820012	185824814	4802	4	chr3	185819981	185824881	4900	single	Variation_4365	chr3	185604555	185822597	218042	Wong et al. (2007)	BAC Array CGH	
1376	chr3	186483668	186486706	3038	2	chr3	186483637	186486773	3136	HiConf								
1377	chr3	186762331	186774679	12348	3	chr3	186762300	186774942	12642	HiConf								
1378	chr3	187048344	187049924	1580	6	chr3	187048313	187050273	1960	single								
1379	chr3	187577544	187581072	3528	19	chr3	187577513	187581139	3626	HiConf								
1380	chr3	187664862	187665842	980	12	chr3	187664831	187665860	1029	HiConf	Variation_44095	chr3	187664743	187665875	1132	Bentley et al. (2008)	Illumina DNA sequencing	y
1381	chr3	187854933	187855617	684	26	chr3	187854902	187855833	931	single	Variation_36114	chr3	187826188	187855437	29249	Kidd et al. (2008)	Paired End Mapping	
1382	chr3	190574482	190582420	7938	12	chr3	190574451	190582585	8134	HiConf	Variation_36118	chr3	190560809	190584710	23901	Kidd et al. (2008)	Paired End Mapping	
1383	chr3	190845667	190854126	8459	46	chr3	190845666	190854290	8624	HiConf	Variation_44097	chr3	190846013	190853601	7588	Bentley et al. (2008)	Illumina DNA sequencing	
1384	chr3	191147733	191149105	1372	6	chr3	191147702	191149123	1421	single								
1385	chr3	191220008	191223144	3136	66	chr3	191219977	191223211	3234	HiConf	Variation_44098	chr3	191220027	191223218	3191	Bentley et al. (2008)	Illumina DNA sequencing	
1386	chr3	191322761	191323643	882	25	chr3	191322730	191323661	931	HiConf								
100220	chr3	191634083	191778621	144538	3	chr3	191634052	191778724	144672	single								
1387	chr3	191961183	191961291	108	40	chr3	191961151	191961298	147	HiConf	Variation_46355	chr3	191961157	191961327	170	Bentley et al. (2008)	Illumina DNA sequencing	
1388	chr3	192064376	192092502	28126	4	chr3	192064345	192093059	28714	HiConf	Variation_47999	chr3	191836948	192071046	234098	Gusev et al. (2009)	SNP genotyping analysis	
1389	chr3	192196578	192200939	4361	3	chr3	192196547	192201006	4459	single								
1390	chr3	192269931	192271254	1323	37	chr3	192269900	192271272	1372	HiConf								
1391	chr3	192494155	192496311	2156	2	chr3	192494124	192496329	2205	HiConf	Variation_3469	chr3	192419528	192761801	342273	Redon et al. (2006)	BAC Array CGH	
1392	chr3	192547395	192565352	17957	19	chr3	192547387	192565713	18326	HiConf	Variation_23177	chr3	192547244	192556527	9283	Korbel et al. (2007)	Paired End Mapping	
1393	chr3	192639489	192646398	6909	5	chr3	192639458	192646514	7056	HiConf	Variation_3469	chr3	192419528	192761801	342273	Redon et al. (2006)	BAC Array CGH	
1394	chr3	192723230	192728816	5586	6	chr3	192723199	192728932	5733	HiConf	Variation_3469	chr3	192419528	192761801	342273	Redon et al. (2006)	BAC Array CGH	
1395	chr3	192806579	192814244	7665	4	chr3	192806548	192817524	10976	HiConf								
1396	chr3	192987487	192990084	2597	6	chr3	192987456	192990151	2695	HiConf								
1397	chr3	193264925	193271393	6468	3	chr3	193264894	193271509	6615	HiConf								
1398	chr3	193338210	193338630	420	3	chr3	193338149	193338639	490	single								
1399	chr3	193605475	193606063	588	28	chr3	193605444	193606081	637	HiConf								y
1400	chr3	194071759	194072749	990	11	chr3	194071728	194073100	1372	single								
1401	chr3	194081069	194092339	11270	2	chr3	194081038	194092553	11515	HiConf	Variation_1660	chr3	194082030	194092410	10380	McCarroll et al. (2005)	Null genotypes	
1402	chr3	194258743	194265113	6370	8	chr3	194258712	194265229	6517	HiConf	Variation_32545	chr3	194260142	194265043	4901	Perry et al. (2008)	Agilent Custom CGH Arrays	
1403	chr3	194358010	194367906	9896	46	chr3	194357790	194368080	10290	HiConf	Variation_31134	chr3	194358036	194367809	9773	Perry et al. (2008)	Agilent Custom CGH Arrays	
1404	chr3	194618893	194624773	5880	13	chr3	194618862	194624889	6027	HiConf	Variation_1662	chr3	194619170	194621399	2229	McCarroll et al. (2005)	Null genotypes	
1405	chr3	194675504	194678477	2973	2	chr3	194675457	194678544	3087	single								
1406	chr3	195373794	195374246	452	3	chr3	195371943	195374589	2646	single								
1407	chr3	195473257	195478304	5047	24	chr3	195473226	195478420	5194	HiConf								
1408	chr3	195480362	195480622	260	31	chr3	195480331	195480625	294	single								
1409	chr3	195571306	195592474	21168	1	chr3	195571275	195592884	21609	single								
1410	chr3	195880129	195881623	1494	37	chr3	195880122	195881641	1519	HiConf	Variation_23677	chr3	195880028	195881636	1608	Levy et al. (2007)	Sequencing	
1411	chr3	195923714	195925477	1763	3	chr3	195923683	195925839	2156	HiConf								
1412						chr3	196037216	196037265	49	single								
1413	chr3	196042022	196050967	8945	11	chr3	196042018	196051132	9114	HiConf	Variation_32546	chr3	196041982	196049198	7216	Perry et al. (2008)	Agilent Custom CGH Arrays	y
1414	chr3	196672581	196736477	63896	32	chr3	196672550	196737769	65219	HiConf	Variation_32548	chr3	196680964	196715761	34797	Perry et al. (2008)	Agilent Custom CGH Arrays	
1415	chr3	196822397	196960452	138055	28	chr3	196822392	196963120	140728	HiConf	Variation_32549	chr3	196767501	196963423	195922	Perry et al. (2008)	Agilent Custom CGH Arrays	
1416	chr3	197002792	197012396	9604	7	chr3	197002761	197012561	9800	single	Variation_0696	chr3	196902846	197038755	135909	Sharp et al. (2005)	BAC Array CGH	
1417	chr3	197091629	197092903	1274	44	chr3	197091598	197092921	1323	single	Variation_4367	chr3	197090272	197248100	157828	Wong et al. (2007)	BAC Array CGH	
1418	chr3	197105447	197107505	2058	1	chr3	197093068	197107817	14749	single	Variation_4367	chr3	197090272	197248100	157828	Wong et al. (2007)	BAC Array CGH	y
100223	chr3	197139441	197216383	76942	23	chr3	197139373	197217945	78572	HiConf	Variation_32551	chr3	197147483	197221646	74163	Perry et al. (2008)	Agilent Custom CGH Arrays	y
1420	chr3	197302770	197322480	19710	44	chr3	197302739	197323466	20727	HiConf	Variation_27273	chr3	197310459	197310764	305	Levy et al. (2007)	Sequencing	
1421	chr3	197539979	197553851	13872	11	chr3	197539948	197554403	14455	HiConf	Variation_43285	chr3	197540204	197541518	1314	Wang et al. (2008)	Illumina DNA sequencing	
1422	chr3	198006410	198042621	36211	21	chr3	198006379	198043374	36995	HiConf	Variation_31139	chr3	198039744	198042001	2257	Perry et al. (2008)	Agilent Custom CGH Arrays	
1423	chr3	198149637	198165660	16023	10	chr3	198149606	198165972	16366	HiConf								
1424	chr3	198595880	198602887	7007	22	chr3	198595849	198603003	7154	HiConf	Variation_6231	chr3	198602286	198603517	1231	Mills et al. (2006)	Sequence trace read mapping	y
100225	chr3	198650074	198710028	59954	54	chr3	198650043	198726826	76783	HiConf	Variation_31143	chr3	198670779	198672125	1346	Perry et al. (2008)	Agilent Custom CGH Arrays	
1426	chr3	198789577	198798926	9349	10	chr3	198789546	198799101	9555	HiConf	Variation_38486	chr3	198792686	198798533	5847	McCarroll et al. (2008)	Affymetrix Human SNP Array 6.0	
100226	chr3	198824451	198880742	56291	12	chr3	198824410	198881887	57477	HiConf	Variation_32558	chr3	198818911	198878259	59348	Perry et al. (2008)	Agilent Custom CGH Arrays	y
1428	chr3	199298148	199400438	102290	15	chr3	199298117	199402536	104419	HiConf	Variation_31148	chr3	199323028	199373344	50316	Perry et al. (2008)	Agilent Custom CGH Arrays	y
1429	chr4	1924	57435	55511	24	chr4	1838	58580	56742	HiConf	Variation_0273	chr4	4287	54931	50644	Sebat et al. (2004)	ROMA	
1430	chr4	235305	238588	3283	7	chr4	235274	238655	3381	HiConf	Variation_7441	chr4	34049	240916	206867	de Smith et al. (2007)	Agilent 185k CGH Arrays/Agilent Custom CGH Arrays	y

locus_id	chrom	start	end	length	Yoruba w/ event	putative chr	putative start	putative end	putative len	putative type	variation_id	DGV_chr	DGV_start	DGV_end	DGV_len	Reference	Method/platform	complex
1431	chr4	1075802	1080457	4655	60	chr4	1075771	1080475	4704	HiConf	Variation_27413	chr4	1079200	1079495	295	Levy et al. (2007)	Sequencing	
1432	chr4	2060702	2061976	1274	14	chr4	2060671	2061994	1323	single								
1433	chr4	3028158	3039281	11123	10	chr4	3028127	3039495	11368	HiConf								
1434	chr4	3310006	3317503	7497	23	chr4	3309975	3317668	7693	single								
1435	chr4	3506300	3540453	34153	8	chr4	3506269	3541157	34888	HiConf	Variation_32567	chr4	3535217	3536695	1478	Perry et al. (2008)	Agilent Custom CGH Arrays	
1436	chr4	3618244	3619784	1540	1	chr4	3616813	3623820	7007	single	Variation_2495	chr4	3593937	3684137	90200	Redon et al. (2006)	Affymetrix 500K EA SNP Mapping Array	
1437						chr4	3632199	3639794	7595	single								
1438	chr4	3718127	3718235	108	57	chr4	3718096	3718243	147	single	Variation_3477	chr4	3364142	4401887	1037745	Redon et al. (2006)	BAC Array CGH	
1439	chr4	3786482	3786545	63	52	chr4	3786451	3786549	98	single	Variation_3477	chr4	3364142	4401887	1037745	Redon et al. (2006)	BAC Array CGH	
1440	chr4	3852975	3997339	144364	5	chr4	3852944	4000287	147343	single	Variation_23239	chr4	3882047	3965213	83166	Korbel et al. (2007)	Paired End Mapping	
1441	chr4	4001053	4238861	237808	12	chr4	4001022	4241955	240933	HiConf	Variation_36272	chr4	4157433	4219778	62345	Kidd et al. (2008)	Paired End Mapping	
1442	chr4	4265359	4293773	28414	7	chr4	4265328	4294189	28861	HiConf	Variation_3477	chr4	3364142	4401887	1037745	Redon et al. (2006)	BAC Array CGH	
1443	chr4	5456402	5462625	6223	4	chr4	5456371	5462741	6370	single	Variation_8995	chr4	5213289	5485050	271761	Pinto et al. (2007)	Affymetrix 500K SNP Mapping Array	
1444	chr4	5477243	5482240	4997	3	chr4	5477196	5482341	5145	HiConf	Variation_8995	chr4	5213289	5485050	271761	Pinto et al. (2007)	Affymetrix 500K SNP Mapping Array	
1445	chr4	5539702	5542593	2891	1	chr4	5539671	5542660	2989	single								
1446	chr4	5812975	5814298	1323	5	chr4	5812944	5814316	1372	HiConf								
1447	chr4	5821158	5830125	8967	2	chr4	5821127	5830290	9163	single								
1448	chr4	5946108	5948460	2352	15	chr4	5946077	5948478	2401	single	Variation_37733	chr4	5946326	5948393	2067	McCarroll et al. (2008)	Affymetrix Human SNP Array 6.0	
1449	chr4	6733342	6736135	2793	26	chr4	6733311	6736202	2891	HiConf	Variation_22505	chr4	6733297	6736755	3458	Korbel et al. (2007)	Paired End Mapping	
1450	chr4	6947423	6950363	2940	19	chr4	6947392	6950430	3038	HiConf	Variation_6238	chr4	6948186	6951235	3049	Mills et al. (2006)	Sequence trace read mapping	
1451	chr4	7561736	7577705	15969	30	chr4	7561705	7577728	16023	single								
100230	chr4	7853972	7957220	103248	23	chr4	7853941	7959340	105399	HiConf	Variation_47968	chr4	7838836	8223391	384555	Gusev et al. (2009)	SNP genotyping analysis	y
1453	chr4	8026746	8027971	1225	17	chr4	8025539	8028038	2499	single	Variation_4373	chr4	8009599	8099960	90361	Wong et al. (2007)	BAC Array CGH	y
1454	chr4	8669597	8694335	24738	53	chr4	8669448	8694830	25382	HiConf	Variation_31158	chr4	8672878	8691387	18509	Perry et al. (2008)	Agilent Custom CGH Arrays	
1455	chr4	8783208	9080393	297185	26	chr4	8783177	9086438	303261	HiConf	Variation_4378	chr4	8979407	9088247	108840	Wong et al. (2007)	BAC Array CGH	
1456	chr4	9235307	9239447	4140	47	chr4	9234908	9239514	4606	HiConf	Variation_32576	chr4	9235364	9239428	4064	Perry et al. (2008)	Agilent Custom CGH Arrays	
1457	chr4	9288202	9303343	15141	18	chr4	9288171	9303655	15484	single	Variation_34433	chr4	8990916	9290749	299833	Zogopoulos et al. (2007)	Affymetrix 500K and 100K SNP Mapping Arrays	y
100232	chr4	9795352	9842773	47421	39	chr4	9795321	9843758	48437	HiConf	Variation_0571	chr4	9807721	9847657	39936	Tuzun et al. (2005)	Paired End Mapping	
1459	chr4	10000715	10010428	9713	7	chr4	10000484	10010578	10094	HiConf	Variation_38032	chr4	10001452	10009766	8314	McCarroll et al. (2008)	Affymetrix Human SNP Array 6.0	
1460	chr4	10650794	10651676	882	45	chr4	10650763	10651694	931	HiConf	Variation_44123	chr4	10650599	10651756	1157	Bentley et al. (2008)	Illumina DNA sequencing	
100233	chr4	10962647	11018358	55711	2	chr4	10962403	11052637	90234	single								
1461	chr4	11275691	11281375	5684	21	chr4	11275660	11281491	5831	HiConf	Variation_32577	chr4	11276182	11279300	3118	Perry et al. (2008)	Agilent Custom CGH Arrays	
1462	chr4	11935966	11936995	1029	4	chr4	11935935	11937013	1078	HiConf								
100234	chr4	11959929	11995893	35964	17	chr4	11948234	11996842	48608	HiConf	Variation_30203	chr4	11974561	11993045	18484	Jakobsson et al. (2008)	Illumina HumanHap550 BeadChip	
100235	chr4	13886142	13907761	21619	8	chr4	13886111	13908210	22099	single								
100236	chr4	15207133	15323340	116207	1	chr4	15207102	15325657	118555	HiConf	Variation_6242	chr4	15247398	15249228	1830	Mills et al. (2006)	Sequence trace read mapping	
1467	chr4	15369600	15377726	8126	5	chr4	15369561	15377891	8330	HiConf	Variation_32580	chr4	15369563	15377431	7868	Perry et al. (2008)	Agilent Custom CGH Arrays	
1468	chr4	16645258	16649080	3822	3	chr4	16645227	16649147	3920	HiConf	Variation_38535	chr4	16645288	16648472	3184	McCarroll et al. (2008)	Affymetrix Human SNP Array 6.0	
100237	chr4	18203629	18248097	44468	26	chr4	18203476	18248997	45521	HiConf								
1470	chr4	19345011	19348686	3675	7	chr4	19344980	19348753	3773	HiConf								
1471	chr4	19432378	19433750	1372	6	chr4	19432347	19433768	1421	HiConf	Variation_8998	chr4	19397361	19735902	338541	Pinto et al. (2007)	Affymetrix 500K SNP Mapping Array	
1472						chr4	19498644	19498693	49	single								
1473	chr4	19597115	19605681	8566	1	chr4	19595860	19606101	10241	single	Variation_8998	chr4	19397361	19735902	338541	Pinto et al. (2007)	Affymetrix 500K SNP Mapping Array	
1474	chr4	19693744	19697811	4067	2	chr4	19693713	19697878	4165	single	Variation_8998	chr4	19397361	19735902	338541	Pinto et al. (2007)	Affymetrix 500K SNP Mapping Array	
1475	chr4	20165840	20174924	9084	8	chr4	20165828	20175089	9261	HiConf	Variation_10039	chr4	20166557	20174959	8402	Wang et al. (2007)	Illumina HumanHap550 BeadChip	
1476	chr4	20690257	20695468	5211	2	chr4	20690226	20695567	5341	HiConf								
1477	chr4	20978083	20988618	10535	45	chr4	20978052	20988832	10780	HiConf	Variation_22799	chr4	20978178	20986741	8563	Korbel et al. (2007)	Paired End Mapping	
1478	chr4	21066626	21067459	833	57	chr4	21066595	21067477	882	HiConf								
1479	chr4	22272173	22275505	3332	18	chr4	22272142	22275572	3430	HiConf	Variation_32584	chr4	22272489	22275269	2780	Perry et al. (2008)	Agilent Custom CGH Arrays	
1480	chr4	22312513	22344938	32425	5	chr4	22312469	22345593	33124	HiConf	Variation_4381	chr4	22275424	22451615	176191	Wong et al. (2007)	BAC Array CGH	
1481	chr4	22531922	22532804	882	6	chr4	22531891	22532822	931	HiConf	Variation_4382	chr4	22474812	22640333	165521	Wong et al. (2007)	BAC Array CGH	
1482	chr4	24575663	24575973	310	22	chr4	24575632	24575975	343	HiConf	Variation_46621	chr4	24575554	24576229	675	Bentley et al. (2008)	Illumina DNA sequencing	
1483	chr4	25049945	25075866	25921	11	chr4	25049903	25076363	26460	HiConf								
1484	chr4	25111204	25117946	6742	5	chr4	25111202	25118062	6860	HiConf	Variation_38546	chr4	25111151	25117108	5957	McCarroll et al. (2008)	Affymetrix Human SNP Array 6.0	
1485	chr4	25814999	25827711	12712	17	chr4	25814646	25827974	13328	HiConf	Variation_41294	chr4	25820208	25820427	219	Wang et al. (2008)	Illumina DNA sequencing	
1486	chr4	25864510	25865196	686	39	chr4	25864479	25865214	735	HiConf	Variation_4383	chr4	25779052	25947411	168359	Wong et al. (2007)	BAC Array CGH	y
1487	chr4	25899643	25904048	4405	11	chr4	25899612	25904414	4802	HiConf	Variation_4383	chr4	25779052	25947411	168359	Wong et al. (2007)	BAC Array CGH	
1488	chr4	26051984	26062911	10927	6	chr4	26051953	26063125	11172	single	Variation_32586	chr4	26054536	26062977	8441	Perry et al. (2008)	Agilent Custom CGH Arrays	
1489	chr4	26248915	26249023	108	12	chr4	26248884	26249031	147	single								
1490	chr4	26565161	26566484	1323	69	chr4	26565130	26566502	1372	HiConf	Variation_44153	chr4	26565054	26566548	1494	Bentley et al. (2008)	Illumina DNA sequencing	
1491	chr4	28030702	28030962	260	67	chr4	28030671	28030965	294	HiConf	Variation_12425	chr4	28030689	28031038	349	Mills et al. (2006)	Sequence trace read mapping	
1492	chr4	28121107	28121793	686	16	chr4	28121076	28121811	735	HiConf								
1493	chr4	28129633	28131446	1813	11	chr4	28129602	28131464	1862	single								
1494	chr4	28553091	28561567	8476	4	chr4	28553060	28561733	8673	HiConf	Variation_38549	chr4	28553137	28561579	8442	McCarroll et al. (2008)	Affymetrix Human SNP Array 6.0	
1495	chr4	28643119	28649327	6208	9	chr4	28643073	28649443	6370	HiConf								
1496	chr4	28835772	28846694	10922	14	chr4	28835741	28848481	12740	HiConf	Variation_32589	chr4	28835895	28850559	14664	Perry et al. (2008)	Agilent Custom CGH Arrays	
1497	chr4	29760794	29761194	400	15	chr4	29760763	29761204	441	single	Variation_3487	chr4	29737882	29896542	158660	Redon et al. (2006)	BAC Array CGH	
1498	chr4	30966782	30972270	5488	1	chr4	30966751	30972386	5635	single								
1499	chr4	31965451	31967901	2450	5	chr4	31965420	31967968	2548	HiConf								
1500	chr4	32741856	32749696	7840	4	chr4	32741825	32749861	8036	HiConf								
1501	chr4	32761701	32761908	207	3	chr4	32761670	32761915	245	single								
1502	chr4	33054378	33063443	9065	16	chr4	33054347	33063608	9261	HiConf	Variation_38550	chr4	33055531	33062581	7050	McCarroll et al. (2008)	Affymetrix Human SNP Array 6.0	
1503	chr4	33852196	33862976	10780	3	chr4	33852165	33863190	11025	single	Variation_44156	chr4	33851459	33863317	11858	Bentley et al. (2008)	Illumina DNA sequencing	
100240	chr4	33995276	34081271	85995	5	chr4	33995245	34083029	87784	HiConf	Variation_8444	chr4	34012429	34080429	68000	Pinto et al. (2007)	Affymetrix 500K SNP Mapping Array	
100241	chr4	34453909	34504144	50235	18	chr4	34440998	34505409	64411	HiConf	Variation_36271	chr4	34453911	34507529	53618	Kidd et al. (2008)	Paired End Mapping	
1506	chr4	34633158	34634530	1372	21	chr4	34633127	34634548	1421	HiConf	Variation_0275	chr4	34485505	34770739	285234	Sebat et al. (2004)	ROMA	y
1507	chr4	34916525	34920739	4214	21	chr4	34916494	34920806	4312	HiConf	Variation_6257	chr4	34916651	34920821	4170	Mills et al. (2006)	Sequence trace read mapping	
1508						chr4	35367784	35368127	343	single								
1509	chr4	35370755	35374185	3430	2	chr4	35370724	35374252	3528	single								y
1510	chr4	35466305	35467775	1470	37	chr4	35466274	35467793	1519	single								
1511	chr4	35523537	35532896	9359	13	chr4	35523506	35533061	9555	HiConf	Variation_37890	chr4	35524746	35527802	3056	McCarroll et al. (2008)	Affymetrix Human SNP Array 6.0	
1512	chr4	35549948	35551663	1715	7	chr4	35549917	35551681	1764	HiConf								
1513	chr4	35696861	35697536	675	39	chr4	35696280	35697554	1274	HiConf								
1514	chr4	36021181	36023582	2401	4	chr4	36021150	36023600	2450	single								
1515	chr4	36045926	36078119	32193	3	chr4	36045895	36078774	32879	HiConf								
1516	chr4	36348158	36350182	2024	11	chr4	36348127	36350430	2303	HiConf								

locus_id	chrom	start	end	length	Yoruba w/ event	putative chr	putative start	putative end	putative len	putative type	variation_id	DGV_chr	DGV_start	DGV_end	DGV_len	Reference	Method/platform	complex
1517	chr4	37139655	37140015	360	13	chr4	37139624	37140016	392	HiConf								
1518	chr4	37258333	37276681	18348	17	chr4	37258302	37277069	18767	HiConf								
1519	chr4	37582223	37586731	4508	9	chr4	37582192	37586798	4606	single								
1520	chr4	37941148	37941638	490	38	chr4	37941117	37941656	539	single								
1521	chr4	38531892	38536878	4986	5	chr4	38531861	38537006	5145	single	Variation_9472	chr4	38524312	38535155	10843	Wang et al. (2007)	Illumina HumanHap550 BeadChip	
1522	chr4	39260277	39265153	4876	7	chr4	39260246	39265587	5341	HiConf	Variation_38551	chr4	39262956	39265557	2601	McCarroll et al. (2008)	Affymetrix Human SNP Array 6.0	
1523	chr4	39671779	39673114	1335	6	chr4	39671748	39673120	1372	single	Variation_5217	chr4	39504050	39725836	221786	Simon-Sanchez et al. (2007)	Illumina HumanHap300 BeadChip	
1524	chr4	39701767	39703286	1519	37	chr4	39701736	39703304	1568	HiConf	Variation_5217	chr4	39504050	39725836	221786	Simon-Sanchez et al. (2007)	Illumina HumanHap300 BeadChip	
1525	chr4	39968131	39971691	3560	17	chr4	39968100	39971922	3822	single								
1526	chr4	40085584	40092101	6517	2	chr4	40085553	40092217	6664	single								
1527	chr4	42400916	42403872	2956	8	chr4	42400901	42403939	3038	HiConf	Variation_10043	chr4	42400885	42403451	2566	Wang et al. (2007)	Illumina HumanHap550 BeadChip	
1528	chr4	42456205	42471198	14993	58	chr4	42456173	42471510	15337	HiConf	Variation_22808	chr4	42456406	42465095	8689	Korbel et al. (2007)	Paired End Mapping	
1529	chr4	42478842	42479577	735	2	chr4	42478811	42479595	784	single								
1530	chr4	43434244	43437086	2842	3	chr4	43434213	43437153	2940	HiConf	Variation_38555	chr4	43433755	43436627	2872	McCarroll et al. (2008)	Affymetrix Human SNP Array 6.0	
1531	chr4	43446611	43446749	138	32	chr4	43446610	43446757	147	HiConf	Variation_40620	chr4	43446562	43446886	324	Wheeler et al. (2008)	Sequencing	
1532	chr4	43712810	43721727	8917	15	chr4	43712778	43721892	9114	HiConf	Variation_38556	chr4	43716642	43720503	3861	McCarroll et al. (2008)	Affymetrix Human SNP Array 6.0	y
100243	chr4	43882704	43892918	10214	33	chr4	43842285	43893049	50764	single								
1534	chr4	44000733	44005192	4459	4	chr4	44000702	44005259	4557	HiConf								
1535	chr4	44020382	44025915	5533	3	chr4	44020351	44026035	5684	HiConf	Variation_38557	chr4	44018589	44025731	7142	McCarroll et al. (2008)	Affymetrix Human SNP Array 6.0	
1536	chr4	45217298	45222058	4760	4	chr4	45216196	45222174	5978	HiConf								
1537	chr4	45307367	45309327	1960	7	chr4	45307336	45309345	2009	HiConf								
1538	chr4	45897594	45899728	2134	23	chr4	45897590	45899746	2156	HiConf	Variation_6263	chr4	45897555	45899785	2230	Mills et al. (2006)	Sequence trace read mapping	
100244						chr4	46057575	46104174	46599	single								
100245	chr4	47065046	47186664	121618	1	chr4	47065015	47189157	124142	single	Variation_4392	chr4	47130896	47373200	242304	Wong et al. (2007)	BAC Array CGH	
1540	chr4	47367278	47372962	5684	2	chr4	47367247	47373078	5831	single	Variation_4392	chr4	47130896	47373200	242304	Wong et al. (2007)	BAC Array CGH	
100246						chr4	47956178	48087106	130928	HiConf								
1541	chr4	48573266	48582429	9163	5	chr4	48573235	48582594	9359	HiConf								y
1542	chr4	48771036	48804550	33514	55	chr4	48770999	48804760	33761	HiConf	Variation_23726	chr4	48789201	48790380	1179	Levy et al. (2007)	Sequencing	
1543	chr4	48854869	48901430	46561	3	chr4	48854838	48911629	56791	HiConf	Variation_31171	chr4	48856018	48857212	1194	Perry et al. (2008)	Agilent Custom CGH Arrays	
1544	chr4	48933857	49321077	387220	16	chr4	48933826	49327149	393323	HiConf	Variation_31173	chr4	48975570	49213260	237690	Perry et al. (2008)	Agilent Custom CGH Arrays	
100248	chr4	52585264	52595496	10232	1	chr4	52585233	52636462	51229	single	Variation_4395	chr4	52478916	52634436	155520	Wong et al. (2007)	BAC Array CGH	
100249	chr4	53512849	53529151	16302	3	chr4	53508099	53529585	21486	single								
1545	chr4	53764571	53774322	9751	4	chr4	53764540	53774536	9996	HiConf	Variation_38559	chr4	53765718	53774999	9281	McCarroll et al. (2008)	Affymetrix Human SNP Array 6.0	
1546	chr4	54704489	54706449	1960	9	chr4	54704458	54706467	2009	single								
1547	chr4	54914605	54915001	396	17	chr4	54914570	54915011	441	single								
1548	chr4	55625591	55625987	396	10	chr4	55625560	55626001	441	single								
100250						chr4	55677745	55718856	41111	single								
1549	chr4	55961731	55963250	1519	31	chr4	55961700	55963268	1568	HiConf								
100251						chr4	56094466	56207950	113484	single								
1550	chr4	57232077	57232987	910	6	chr4	57232074	57233005	931	HiConf	Variation_33759	chr4	57232102	57233077	975	Perry et al. (2008)	Agilent Custom CGH Arrays	
1551	chr4	57234555	57239014	4459	15	chr4	57234524	57239081	4557	HiConf	Variation_37428	chr4	57234948	57239195	4247	Cooper et al. (2008)	Illumina Human 1M BeadChip	
1552	chr4	58332988	58337055	4067	1	chr4	58332957	58337122	4165	single	Variation_3495	chr4	57748174	58349780	601606	Redon et al. (2006)	BAC Array CGH	
1553	chr4	58509437	58519139	9702	14	chr4	58509406	58519353	9947	single	Variation_23179	chr4	58506344	58515900	9556	Korbel et al. (2007)	Paired End Mapping	
1554	chr4	58579164	58584122	4958	10	chr4	58579133	58589374	10241	HiConf	Variation_43260	chr4	58582000	58583166	1166	Wang et al. (2008)	Illumina DNA sequencing	
1555	chr4	59821118	59826263	5145	3	chr4	59821087	59826379	5292	HiConf								
1556	chr4	59974488	59975027	539	9	chr4	59974457	59975045	588	single								y
1557	chr4	60052055	60085259	33204	42	chr4	60052024	60085638	33614	HiConf								
1558	chr4	60961005	60977757	16752	18	chr4	60960974	60978467	17493	HiConf	Variation_3496	chr4	60813753	60999844	186091	Redon et al. (2006)	BAC Array CGH	
1559	chr4	61012847	61014268	1421	31	chr4	61012816	61014286	1470	HiConf	Variation_6271	chr4	61012702	61015780	3078	Mills et al. (2006)	Sequence trace read mapping	
1560	chr4	61183024	61184053	1029	16	chr4	61182993	61184071	1078	single								
1561	chr4	61621672	61624701	3029	24	chr4	61621641	61624777	3136	HiConf	Variation_39372	chr4	61621784	61624838	3054	Wheeler et al. (2008)	Sequencing	
1562	chr4	61682040	61682971	931	83	chr4	61682009	61682989	980	HiConf								
1563	chr4	61711018	61711111	93	61	chr4	61711017	61711115	98	HiConf	Variation_27685	chr4	61710907	61711190	283	Levy et al. (2007)	Sequencing	
1564	chr4	61966779	61967808	1029	25	chr4	61966748	61967826	1078	HiConf	Variation_43316	chr4	61966506	61967945	1439	Wang et al. (2008)	Illumina DNA sequencing	
1565	chr4	62181497	62182227	730	57	chr4	62181466	62182250	784	HiConf	Variation_27686	chr4	62181755	62182309	554	Levy et al. (2007)	Sequencing	y
1566	chr4	63352132	63358526	6394	18	chr4	63352125	63358642	6517	HiConf	Variation_39373	chr4	63352021	63358721	6700	Wheeler et al. (2008)	Sequencing	
1567	chr4	63816934	63836583	19649	3	chr4	63816890	63836980	20090	HiConf	Variation_39374	chr4	63816903	63837131	20228	Wheeler et al. (2008)	Sequencing	
1568	chr4	63925848	63937797	11949	2	chr4	63925817	63938018	12201	HiConf	Variation_38561	chr4	63926525	63935689	9164	McCarroll et al. (2008)	Affymetrix Human SNP Array 6.0	
100253	chr4	64443313	64513352	70039	19	chr4	64443282	64514307	71025	single								
1569	chr4	64779820	64780359	539	39	chr4	64779789	64780377	588	HiConf								
1570	chr4	65502638	65523248	20610	6	chr4	65502637	65523658	21021	HiConf	Variation_38562	chr4	65502562	65522613	20051	McCarroll et al. (2008)	Affymetrix Human SNP Array 6.0	
1571	chr4	66644368	66653308	8940	6	chr4	66644337	66653696	9359	HiConf	Variation_10047	chr4	66632070	66652993	20923	Wang et al. (2007)	Illumina HumanHap550 BeadChip	
1572	chr4	68035576	68036115	539	6	chr4	68035545	68036133	588	single								
1573	chr4	68064388	68065123	735	8	chr4	68064357	68065141	784	single	Variation_44167	chr4	68063491	68065949	2458	Bentley et al. (2008)	Illumina DNA sequencing	
100254	chr4	68420177	68688599	268422	1	chr4	68420146	68694081	273935	HiConf	Variation_3498	chr4	68459601	68695959	236358	Redon et al. (2006)	BAC Array CGH	
100255	chr4	69035372	69227883	192511	10	chr4	69035341	69229136	193795	HiConf	Variation_31177	chr4	69055784	69219241	163457	Perry et al. (2008)	Agilent Custom CGH Arrays	
100256	chr4	69515327	69570330	55003	5	chr4	69515272	69571426	56154	HiConf	Variation_31178	chr4	69524193	69571251	47058	Perry et al. (2008)	Agilent Custom CGH Arrays	
1578	chr4	69714463	69751507	37044	2	chr4	69714432	69752260	37828	HiConf	Variation_37736	chr4	69713484	69751804	38320	McCarroll et al. (2008)	Affymetrix Human SNP Array 6.0	
1579	chr4	70060646	70082355	21709	23	chr4	70060568	70082814	22246	HiConf	Variation_31180	chr4	70058699	70327768	269069	Perry et al. (2008)	Agilent Custom CGH Arrays	
1580	chr4	70119840	70265954	146114	27	chr4	70119809	70267544	147735	HiConf	Variation_22939	chr4	70162456	70260011	97555	Korbel et al. (2007)	Paired End Mapping	
1581	chr4	70469651	70470876	1225	5	chr4	70469620	70470894	1274	HiConf								
1582	chr4	70500245	70508862	8617	46	chr4	70500245	70509800	9555	HiConf	Variation_7451	chr4	70500160	70508927	8767	de Smith et al. (2007)	Agilent 185k CGH Arrays/Agilent Custom CGH Arrays	
100259	chr4	70638925	70677840	38915	2	chr4	70618605	70696515	77910	HiConf								
1584						chr4	71197417	71199034	1617	single								
1585	chr4	71407168	71407528	360	17	chr4	71407137	71407529	392	single								
1586	chr4	71520946	71521153	207	14	chr4	71520915	71521160	245	single								
1587	chr4	72164455	72168775	4320	4	chr4	72164040	72168842	4802	HiConf	Variation_38566	chr4	72164818	72167328	2510	McCarroll et al. (2008)	Affymetrix Human SNP Array 6.0	
1588	chr4	73638285	73644216	5931	10	chr4	73638254	73644330	6076	HiConf	Variation_38567	chr4	73639702	73644176	4474	McCarroll et al. (2008)	Affymetrix Human SNP Array 6.0	
1589	chr4	73773133	73777200	4067	24	chr4	73773102	73777267	4165	HiConf								
1590	chr4	73780483	73785677	5194	7	chr4	73780452	73785793	5341	single								
1591	chr4	74332223	74340063	7840	8	chr4	74332192	74340228	8036	single								
1592	chr4	74774399	74775575	1176	5	chr4	74774368	74775593	1225	HiConf	Variation_10765	chr4	74774902	74775136	234	Conrad et al. (2005)	Mendelian inconsistencies	
1593	chr4	75300316	75302325	2009	6	chr4	75300285	75302343	2058	HiConf	Variation_38568	chr4	75299976	75301070	1094	McCarroll et al. (2008)	Affymetrix Human SNP Array 6.0	
100260						chr4	75433982	75493860	59878	single								y
1595	chr4	76962396	76963278	882	41	chr4	76962365	76963296	931	HiConf								
1596	chr4	77664958	77671916	6958	2	chr4	77664927	77672032	7105	HiConf	Variation_2532	chr4	77611116	77674429	63313	Redon et al. (2006)	Affymetrix 500K EA SNP Mapping Array	

locus_id	chrom	start	end	length	Yoruba w/ event	putative chr	putative start	putative end	putative len	putative type	variation_id	DGV_chr	DGV_start	DGV_end	DGV_len	Reference	Method/platform	complex
1597	chr4	78001980	78008203	6223	10	chr4	78001949	78008319	6370	HiConf	Variation_32619	chr4	78002131	78006365	4234	Perry et al. (2008)	Agilent Custom CGH Arrays	
100261						chr4	79114470	79204850	90380	single								
1599	chr4	80481625	80484026	2401	2	chr4	80481594	80484093	2499	single								
1600	chr4	80580605	80581839	1234	14	chr4	80580574	80581848	1274	HiConf	Variation_43422	chr4	80579625	80581811	2186	Wang et al. (2008)	Illumina DNA sequencing	
100262	chr4	81063910	81125154	61244	17	chr4	81059084	81125381	66297	HiConf	Variation_23128	chr4	81106900	81114109	7209	Korbel et al. (2007)	Paired End Mapping	
1602	chr4	81728136	81728918	782	16	chr4	81728105	81729281	1176	HiConf								
1603	chr4	81750333	81754792	4459	4	chr4	81750302	81754859	4557	HiConf								
100263	chr4	83204611	83223567	18956	2	chr4	83157190	83224933	67743	HiConf								
1604	chr4	83584697	83586657	1960	4	chr4	83584666	83586675	2009	single								
1605	chr4	83854834	83860227	5393	4	chr4	83854803	83860291	5488	HiConf	Variation_10050	chr4	83855747	83859356	3609	Wang et al. (2007)	Illumina HumanHap550 BeadChip	
1606	chr4	84808521	84810726	2205	3	chr4	84808490	84810744	2254	single								
1607	chr4	85078881	85080737	1856	4	chr4	85075834	85087447	11613	single								
1608	chr4	85273384	85276079	2695	1	chr4	85273353	85276146	2793	single								y
1609	chr4	86790424	86791796	1372	41	chr4	86790393	86791814	1421	HiConf	Variation_44172	chr4	86790325	86791940	1615	Bentley et al. (2008)	Illumina DNA sequencing	
1610	chr4	87137148	87145331	8183	23	chr4	87137117	87145496	8379	single								
1611	chr4	87195478	87199084	3606	21	chr4	87195476	87199151	3675	HiConf	Variation_0981	chr4	87195411	87198976	3565	Hinds et al. (2005)	Oligo arrays	
1612	chr4	89061966	89069879	7913	14	chr4	89061935	89071147	9212	single								
1613	chr4	89334945	89338865	3920	2	chr4	89334914	89338932	4018	single	Variation_2536	chr4	89177516	89348280	170764	Redon et al. (2006)	Affymetrix 500K EA SNP Mapping Array	
1614	chr4	90646822	90647508	686	24	chr4	90646791	90647526	735	single	Variation_10893	chr4	90646818	90647687	869	Hinds et al. (2005)	Oligo arrays	
1615	chr4	90799552	90810985	11433	6	chr4	90799475	90811235	11760	single	Variation_29745	chr4	90791345	90837287	45942	Jakobsson et al. (2008)	Illumina HumanHap550 BeadChip	
1616	chr4	92105062	92118635	13573	3	chr4	92105031	92118898	13867	single	Variation_10052	chr4	92107906	92117088	9182	Wang et al. (2007)	Illumina HumanHap550 BeadChip	
1617	chr4	92148968	92154891	5923	16	chr4	92147661	92155011	7350	HiConf	Variation_39384	chr4	92150580	92154860	4280	Wheeler et al. (2008)	Sequencing	
1618	chr4	92179101	92185716	6615	11	chr4	92179070	92185832	6762	HiConf	Variation_36321	chr4	92184304	92193865	9561	Kidd et al. (2008)	Paired End Mapping	
1619	chr4	92186451	92199044	12593	11	chr4	92186420	92199307	12887	HiConf	Variation_36321	chr4	92184304	92193865	9561	Kidd et al. (2008)	Paired End Mapping	
1620	chr4	92379364	92381375	2011	24	chr4	92379333	92381391	2058	HiConf	Variation_23740	chr4	92380266	92381517	1251	Levy et al. (2007)	Sequencing	
1621	chr4	92691837	92697227	5390	2	chr4	92691806	92697343	5537	HiConf	Variation_38577	chr4	92692337	92697344	5007	McCarroll et al. (2008)	Affymetrix Human SNP Array 6.0	
1622	chr4	92975890	92979418	3528	1	chr4	92975859	92979485	3626	single								
1623	chr4	93786938	93788947	2009	34	chr4	93786907	93788965	2058	HiConf	Variation_4406	chr4	93729981	93904958	174977	Wong et al. (2007)	BAC Array CGH	
1624	chr4	95853856	95856306	2450	5	chr4	95853825	95856373	2548	single								
1625	chr4	95856894	95857531	637	15	chr4	95856863	95857549	686	HiConf								
100264						chr4	97244004	97319660	75656	single								
1626	chr4	97536614	97544552	7938	8	chr4	97536583	97544717	8134	HiConf	Variation_38579	chr4	97539253	97541426	2173	McCarroll et al. (2008)	Affymetrix Human SNP Array 6.0	
1627	chr4	98574091	98577227	3136	5	chr4	98574060	98579009	4949	HiConf	Variation_7460	chr4	98574748	98576965	2217	de Smith et al. (2007)	Agilent 185k CGH Arrays/Agilent Custom CGH Arrays	
100265	chr4	98834330	98890925	56595	3	chr4	98834299	98892095	57796	single	Variation_29748	chr4	98817016	98926069	109053	Jakobsson et al. (2008)	Illumina HumanHap550 BeadChip	
1629	chr4	99209357	99209964	607	3	chr4	99209296	99209982	686	single								
1630	chr4	99472555	99502317	29762	2	chr4	99472524	99502904	30380	single								
1631	chr4	99899345	99901599	2254	26	chr4	99899314	99901617	2303	HiConf	Variation_44176	chr4	99899839	99901958	2119	Bentley et al. (2008)	Illumina DNA sequencing	
1632	chr4	101818773	101821517	2744	49	chr4	101818742	101821584	2842	HiConf	Variation_44121	chr4	101818681	101820623	1942	Bentley et al. (2008)	Illumina DNA sequencing	
1633	chr4	102179237	102179854	617	15	chr4	102179235	102179872	637	single	Variation_39387	chr4	102179179	102180213	1034	Wheeler et al. (2008)	Sequencing	
1634	chr4	102745804	102757280	11476	8	chr4	102745773	102757484	11711	HiConf								
1635						chr4	102923398	102936285	12887	single								
100266	chr4	103006974	103100858	93884	10	chr4	103006943	103102787	95844	HiConf	Variation_2537	chr4	102991884	103152972	161088	Redon et al. (2006)	Affymetrix 500K EA SNP Mapping Array	
1637	chr4	103816258	103823020	6762	7	chr4	103816227	103823136	6909	single								
100267	chr4	104022426	104117443	95017	5	chr4	104022395	104117896	95501	HiConf	Variation_32626	chr4	104039295	104090622	51327	Perry et al. (2008)	Agilent Custom CGH Arrays	
100268	chr4	104417317	104477146	59829	13	chr4	104417286	104478340	61054	HiConf	Variation_32627	chr4	104419680	104468207	48527	Perry et al. (2008)	Agilent Custom CGH Arrays	
1640	chr4	105001862	105002312	450	3	chr4	105001831	105002321	490	HiConf								
100269	chr4	105394452	105428324	33872	14	chr4	105383909	105442317	58408	HiConf								
100270	chr4	106243825	106287505	43680	2	chr4	106235994	106314027	78033	HiConf								
1642	chr4	106927513	106936382	8869	6	chr4	106927482	106936547	9065	HiConf	Variation_22840	chr4	106926305	106937012	10707	Korbel et al. (2007)	Paired End Mapping	
1643	chr4	107159773	107162664	2891	26	chr4	107159742	107162731	2989	HiConf	Variation_38042	chr4	107162376	107165884	3508	McCarroll et al. (2008)	Affymetrix Human SNP Array 6.0	
1644	chr4	107276148	107282665	6517	10	chr4	107276117	107282781	6664	HiConf	Variation_43638	chr4	107275973	107282873	6900	Wang et al. (2008)	Illumina DNA sequencing	
1645	chr4	108374091	108381147	7056	14	chr4	108374060	108381263	7203	HiConf								
100271	chr4	109279058	109321972	42914	7	chr4	109277155	109353301	76146	single								
1646	chr4	111059634	111065122	5488	10	chr4	111059603	111065238	5635	HiConf	Variation_6305	chr4	111060190	111065187	4997	Mills et al. (2006)	Sequence trace read mapping	
1647	chr4	111223539	111224323	784	16	chr4	111223508	111224341	833	HiConf	Variation_10741	chr4	111223542	111223994	452	Conrad et al. (2005)	Mendelian inconsistencies	
1648	chr4	111516167	111524448	8281	8	chr4	111516136	111524613	8477	HiConf	Variation_38515	chr4	111517080	111519963	2883	McCarroll et al. (2008)	Affymetrix Human SNP Array 6.0	
1649	chr4	111601115	111614774	13659	5	chr4	111601053	111615018	13965	single								y
100272	chr4	111708646	111790038	81392	51	chr4	111704198	111791149	86951	single								
1650	chr4	112516845	112529144	12299	10	chr4	112516814	112529407	12593	HiConf	Variation_44126	chr4	112516763	112529060	12297	Bentley et al. (2008)	Illumina DNA sequencing	
1651	chr4	112896791	112900735	3944	3	chr4	112896760	112900778	4018	HiConf	Variation_38517	chr4	112896236	112900044	3808	McCarroll et al. (2008)	Affymetrix Human SNP Array 6.0	
1652	chr4	113450344	113451618	1274	4	chr4	113450313	113451636	1323	HiConf								
100273	chr4	114893961	114938887	44926	4	chr4	114879447	114951918	72471	HiConf								
1653	chr4	115385256	115401558	16302	44	chr4	115385225	115406148	20923	HiConf	Variation_36179	chr4	115391467	115403744	12277	Kidd et al. (2008)	Paired End Mapping	
1654	chr4	115726688	115733009	6321	44	chr4	115726657	115733125	6468	HiConf	Variation_38022	chr4	115727615	115729903	2288	McCarroll et al. (2008)	Affymetrix Human SNP Array 6.0	
1655	chr4	116148284	116148684	400	69	chr4	116148253	116148694	441	HiConf	Variation_23042	chr4	116147379	116153212	5833	Korbel et al. (2007)	Paired End Mapping	
1656	chr4	116200567	116204193	3626	4	chr4	116200536	116204260	3724	HiConf								
100274	chr4	116318588	116347313	28725	2	chr4	116294959	116360521	65562	single	Variation_2541	chr4	116301040	116402149	101109	Redon et al. (2006)	Affymetrix 500K EA SNP Mapping Array	
1657	chr4	116386375	116396861	10486	10	chr4	116386344	116397075	10731	HiConf	Variation_32633	chr4	116386643	116396229	9586	Perry et al. (2008)	Agilent Custom CGH Arrays	
1658	chr4	116485698	116489961	4263	1	chr4	116485667	116490028	4361	single	Variation_3509	chr4	116483697	116671246	187549	Redon et al. (2006)	BAC Array CGH	
1659	chr4	117866616	117869213	2597	3	chr4	117866585	117869280	2695	HiConf	Variation_38519	chr4	117866565	117869001	2436	McCarroll et al. (2008)	Affymetrix Human SNP Array 6.0	
1660	chr4	119344799	119349552	4753	57	chr4	119344768	119349619	4851	HiConf	Variation_6311	chr4	119344860	119349647	4787	Mills et al. (2006)	Sequence trace read mapping	
1661	chr4	119402668	119413791	11123	4	chr4	119402637	119414005	11368	single	Variation_47878	chr4	119254572	119674839	420267	Gusev et al. (2009)	SNP genotyping analysis	
100275	chr4	119558194	119609572	51378	16	chr4	119558163	119610446	52283	HiConf	Variation_4411	chr4	119557416	119715366	157950	Wong et al. (2007)	BAC Array CGH	
1663	chr4	119738202	119810250	72048	7	chr4	119737993	119811738	73745	HiConf	Variation_8453	chr4	118783787	119807559	1023772	Pinto et al. (2007)	Affymetrix 500K SNP Mapping Array	
1664	chr4	120246840	120246948	108	45	chr4	120246809	120246956	147	HiConf								
100276	chr4	120455769	120463992	8223	2	chr4	120394936	120465668	70732	HiConf								
100277	chr4	120550665	120609269	58604	3	chr4	120498792	120611492	112700	HiConf								
1665	chr4	120772316	120775451	3135	9	chr4	120772285	120775519	3234	single								
1666	chr4	121084938	121085818	880	9	chr4	121084709	121085836	1127	HiConf								
1667	chr4	121140453	121152391	11938	6	chr4	121140422	121152623	12201	HiConf								
1668	chr4	121183328	121184357	1029	14	chr4	121183297	121184375	1078	HiConf								
100278	chr4	121742327	121764153	21826	3	chr4	121733518	121764878	31360	HiConf	Variation_38522	chr4	121743029	121762869	19840	McCarroll et al. (2008)	Affymetrix Human SNP Array 6.0	
1670	chr4	122501477	122509603	8126	19	chr4	122501446	122509972	8526	HiConf	Variation_44128	chr4	122501703	122509401	7698	Bentley et al. (2008)	Illumina DNA sequencing	
1671	chr4	122635100	122650621	15521	11	chr4	122635069	122650896	15827	HiConf								
1672	chr4	124240311	124269740	29429	1	chr4	124240260	124270346	30086	single								
1673	chr4	125155317	125166195	10878	10	chr4	125155286	125166409	11123	HiConf	Variation_44129	chr4	125163009	125164635	1626	Bentley et al. (2008)	Illumina DNA sequencing	

locus_id	chrom	start	end	length	Yoruba w/ event	putative chr	putative start	putative end	putative len	putative type	variation_id	DGV_chr	DGV_start	DGV_end	DGV_len	Reference	Method/platform	complex
100279	chr4	126780840	126896728	115888	1	chr4	126780763	126899098	118335	single	Variation_37727	chr4	126826946	126843898	16952	McCarroll et al. (2008)	Affymetrix Human SNP Array 6.0	
1674	chr4	127120560	127124628	4068	2	chr4	127120529	127124694	4165	single								
1675	chr4	127337533	127340668	3135	4	chr4	127335982	127340735	4753	single								
1676	chr4	127585325	127590519	5194	2	chr4	127585294	127590635	5341	HiConf								
1677	chr4	128078657	128079196	539	8	chr4	128078626	128079214	588	single								
1678	chr4	128376087	128376626	539	10	chr4	128376056	128376644	588	single								
1679	chr4	128605407	128609915	4508	2	chr4	128605376	128609982	4606	single								
1680	chr4	129184489	129184799	310	32	chr4	129184458	129184801	343	HiConf								
1681	chr4	129332420	129332483	63	2	chr4	129332389	129332487	98	single								
1682	chr4	129381033	129384948	3915	19	chr4	129380654	129385015	4361	single								
1683	chr4	130285372	130287822	2450	33	chr4	130285341	130287889	2548	HiConf	Variation_7428	chr4	130286209	130287853	1644	de Smith et al. (2007)	Agilent 185k CGH Arrays/Agilent Custom CGH Arrays	
1684	chr4	130500286	130502540	2254	66	chr4	130500255	130502558	2303	HiConf	Variation_44131	chr4	130500285	130502385	2100	Bentley et al. (2008)	Illumina DNA sequencing	
1685	chr4	130804772	130807320	2548	5	chr4	130804741	130807387	2646	HiConf								
1686	chr4	130845981	130846044	63	58	chr4	130845950	130846048	98	HiConf	Variation_46508	chr4	130845823	130846100	277	Bentley et al. (2008)	Illumina DNA sequencing	
1687	chr4	132709892	132713322	3430	56	chr4	132709861	132713389	3528	HiConf	Variation_44132	chr4	132709861	132713086	3225	Bentley et al. (2008)	Illumina DNA sequencing	
100280	chr4	132852526	133107086	254560	10	chr4	132795219	133113450	318231	HiConf	Variation_31191	chr4	132906454	133114707	208253	Perry et al. (2008)	Agilent Custom CGH Arrays	
1688	chr4	133221648	133225225	3577	4	chr4	133221617	133225292	3675	HiConf	Variation_30209	chr4	133156764	133266750	109986	Jakobsson et al. (2008)	Illumina HumanHap550 BeadChip	
1689	chr4	133398146	133402262	4116	53	chr4	133398115	133402329	4214	HiConf	Variation_32647	chr4	133401103	133402307	1204	Perry et al. (2008)	Agilent Custom CGH Arrays	
1690	chr4	134392944	134397696	4752	2	chr4	134392913	134397764	4851	single	Variation_4415	chr4	134334527	134478079	143552	Wong et al. (2007)	BAC Array CGH	
1691	chr4	135652563	135655642	3079	51	chr4	135652556	135655643	3087	HiConf	Variation_43498	chr4	135652408	135655243	2835	Wang et al. (2008)	Illumina DNA sequencing	
1692	chr4	136037607	136056788	19181	3	chr4	136037598	136057149	19551	HiConf	Variation_22893	chr4	136037048	136059319	22271	Korbel et al. (2007)	Paired End Mapping	
1693	chr4	136727892	136730930	3038	3	chr4	136727861	136730997	3136	HiConf								
1694	chr4	136863132	136869628	6496	3	chr4	136863101	136869716	6615	HiConf								
1695	chr4	137578777	137583971	5194	17	chr4	137578746	137584087	5341	HiConf	Variation_38523	chr4	137578497	137583646	5149	McCarroll et al. (2008)	Affymetrix Human SNP Array 6.0	
1696	chr4	138311304	138324753	13449	34	chr4	138310953	138325016	14063	HiConf	Variation_32649	chr4	138311975	138320136	8161	Perry et al. (2008)	Agilent Custom CGH Arrays	
1697	chr4	138543734	138552505	8771	2	chr4	138543703	138552670	8967	HiConf	Variation_38525	chr4	138543995	138549443	5448	McCarroll et al. (2008)	Affymetrix Human SNP Array 6.0	
1698	chr4	139186075	139186565	490	64	chr4	139186044	139186583	539	HiConf	Variation_6323	chr4	139185662	139186667	1005	Mills et al. (2006)	Sequence trace read mapping	
1699	chr4	140452186	140455175	2989	4	chr4	140452155	140455242	3087	single	Variation_37872	chr4	140452973	140457810	4837	McCarroll et al. (2008)	Affymetrix Human SNP Array 6.0	
1700	chr4	140747558	140749912	2354	14	chr4	140747527	140750320	2793	HiConf								y
1701	chr4	142449791	142453052	3261	39	chr4	142449738	142453119	3381	HiConf	Variation_6328	chr4	142450229	142452512	2283	Mills et al. (2006)	Sequence trace read mapping	
100283						chr4	142733473	142833286	99813	HiConf								
1702	chr4	143835630	143840438	4808	2	chr4	143835556	143840554	4998	HiConf	Variation_38526	chr4	143835324	143839399	4075	McCarroll et al. (2008)	Affymetrix Human SNP Array 6.0	
1703	chr4	143914575	143915800	1225	2	chr4	143914544	143915818	1274	single								
1704	chr4	144014388	144020415	6027	10	chr4	144014357	144020531	6174	HiConf	Variation_1102	chr4	144014721	144015937	1216	Conrad et al. (2005)	Mendelian inconsistencies	
1705	chr4	144077402	144078039	637	36	chr4	144077371	144078057	686	HiConf								
1706	chr4	144261985	144262245	260	16	chr4	144261954	144262248	294	single								
1707	chr4	144482240	144489051	6811	1	chr4	144482209	144489167	6958	single								
100284	chr4	144922211	145287310	365099	2	chr4	144922180	145294776	372596	HiConf	Variation_34437	chr4	144941110	145251291	310181	Zogopoulos et al. (2007)	Affymetrix 500K and 100K SNP Mapping Arrays	
1711	chr4	145538533	145539219	686	53	chr4	145538502	145539237	735	HiConf	Variation_36202	chr4	145526100	145554666	28566	Kidd et al. (2008)	Paired End Mapping	
1712	chr4	146152025	146154561	2536	3	chr4	146147180	146154726	7546	HiConf								
1713	chr4	147121380	147123781	2401	12	chr4	147121349	147123848	2499	HiConf								
1714	chr4	147145439	147145547	108	57	chr4	147145408	147145555	147	single	Variation_23706	chr4	147139031	147147193	8162	Levy et al. (2007)	Sequencing	
1715	chr4	147549395	147552041	2646	2	chr4	147549364	147552108	2744	single								
1716	chr4	149645566	149649878	4312	15	chr4	149645535	149649945	4410	HiConf								y
1717	chr4	151389329	151398051	8722	34	chr4	151389298	151398216	8918	HiConf	Variation_0578	chr4	151379647	151409247	29600	Tuzun et al. (2005)	Paired End Mapping	
1718	chr4	152091989	152106122	14133	55	chr4	152091958	152106511	14553	HiConf	Variation_37986	chr4	152095801	152103748	7947	McCarroll et al. (2008)	Affymetrix Human SNP Array 6.0	
100285	chr4	152346373	152393980	47607	5	chr4	152346342	152394950	48608	single								
1719	chr4	152472033	152473797	1764	33	chr4	152472002	152473815	1813	HiConf	Variation_44136	chr4	152471990	152473620	1630	Bentley et al. (2008)	Illumina DNA sequencing	
1720	chr4	153009497	153014267	4770	7	chr4	153009042	153014383	5341	HiConf	Variation_32657	chr4	153009840	153013203	3363	Perry et al. (2008)	Agilent Custom CGH Arrays	
1721	chr4	153118735	153120254	1519	8	chr4	153118704	153120272	1568	single								
1722	chr4	153209679	153212833	3154	5	chr4	153209648	153212882	3234	HiConf	Variation_39401	chr4	153209764	153213399	3635	Wheeler et al. (2008)	Sequencing	
100286	chr4	154239071	154296002	56931	24	chr4	154239040	154297179	58139	HiConf								
1724	chr4	154452123	154454132	2009	9	chr4	154452092	154454150	2058	HiConf								
1725	chr4	155061928	155065358	3430	1	chr4	155061897	155065425	3528	HiConf								
1726	chr4	155487085	155489845	2760	11	chr4	155486433	155489912	3479	HiConf								
1727	chr4	155641451	155645273	3822	4	chr4	155641420	155645340	3920	HiConf								
1728	chr4	155648262	155651839	3577	2	chr4	155648231	155651906	3675	HiConf								
1729	chr4	155971123	155976541	5418	1	chr4	155971092	155977413	6321	single								
1730	chr4	156201668	156202697	1029	2	chr4	156201637	156202715	1078	single								
1731	chr4	156442160	156442560	400	8	chr4	156442129	156442570	441	single								
1732	chr4	156468914	156468977	63	37	chr4	156468883	156468981	98	single								
1733	chr4	156550401	156565542	15141	4	chr4	156550370	156565854	15484	HiConf								
1734	chr4	156813678	156817549	3871	8	chr4	156813647	156817616	3969	HiConf	Variation_38530	chr4	156813718	156817445	3727	McCarroll et al. (2008)	Affymetrix Human SNP Array 6.0	
1735	chr4	156893254	156898595	5341	4	chr4	156893223	156898711	5488	HiConf								
1736	chr4	157099103	157099413	310	17	chr4	157099072	157099415	343	single	Variation_10753	chr4	157098929	157099379	450	Conrad et al. (2005)	Mendelian inconsistencies	
1737	chr4	157626833	157629200	2367	6	chr4	157626802	157629203	2401	HiConf	Variation_44137	chr4	157626740	157631878	5138	Bentley et al. (2008)	Illumina DNA sequencing	
100287	chr4	158458780	158554183	95403	2	chr4	158458749	158556112	97363	single	Variation_2551	chr4	158403546	158564742	161196	Redon et al. (2006)	Affymetrix 500K EA SNP Mapping Array	
1738	chr4	158947824	158951058	3234	10	chr4	158947793	158951125	3332	HiConf	Variation_38531	chr4	158949012	158950828	1816	McCarroll et al. (2008)	Affymetrix Human SNP Array 6.0	
100288	chr4	159889328	159914753	25425	1	chr4	159867499	159928063	60564	single								
1740	chr4	160019601	160020091	490	40	chr4	160019570	160020109	539	HiConf	Variation_4420	chr4	159993061	160190193	197132	Wong et al. (2007)	BAC Array CGH	
1741						chr4	161692773	161702818	10045	single								
1742	chr4	162098475	162105335	6860	23	chr4	162098444	162105451	7007	HiConf	Variation_23119	chr4	162097883	162104865	6982	Korbel et al. (2007)	Paired End Mapping	
1743	chr4	162272719	162273029	310	87	chr4	162272688	162273031	343	HiConf	Variation_34590	chr4	162231787	162329137	97350	Zogopoulos et al. (2007)	Affymetrix 500K and 100K SNP Mapping Arrays	
1744	chr4	162411655	162428564	16909	1	chr4	162411603	162428900	17297	HiConf	Variation_38533	chr4	162413794	162424561	10767	McCarroll et al. (2008)	Affymetrix Human SNP Array 6.0	
1745	chr4	162669080	162670893	1813	14	chr4	162669049	162670911	1862	HiConf	Variation_31202	chr4	162669049	162670579	1530	Perry et al. (2008)	Agilent Custom CGH Arrays	
1746	chr4	163194752	163196957	2205	4	chr4	163194721	163196975	2254	single								
1747	chr4	163497282	163500813	3531	5	chr4	163497247	163500873	3626	HiConf								
1748	chr4	163589888	163591995	2107	2	chr4	163589857	163592013	2156	HiConf								
1749	chr4	163613947	163615858	1911	26	chr4	163613916	163615876	1960	HiConf	Variation_6343	chr4	163613456	163615872	2416	Mills et al. (2006)	Sequence trace read mapping	
1750	chr4	164153339	164154270	931	7	chr4	164153308	164154288	980	single								
1751	chr4	164174213	164177104	2891	4	chr4	164174182	164177171	2989	HiConf	Variation_32664	chr4	164174233	164176945	2712	Perry et al. (2008)	Agilent Custom CGH Arrays	
100289	chr4	164268563	164348972	80409	6	chr4	164268532	164350607	82075	HiConf								
1752	chr4	164582432	164584147	1715	6	chr4	164582401	164584165	1764	HiConf								
1753	chr4	164833263	164834341	1078	6	chr4	164833232	164834359	1127	HiConf								
1754	chr4	165024559	165038034	13475	2	chr4	165024528	165038297	13769	HiConf	Variation_22875	chr4	165023789	165040669	16880	Korbel et al. (2007)	Paired End Mapping	
1755	chr4	165422684	165425526	2842	1	chr4	165422653	165425593	2940	single								

locus_id	chrom	start	end	length	Yoruba w/ event	putative chr	putative start	putative end	putative len	putative type	variation_id	DGV_chr	DGV_start	DGV_end	DGV_len	Reference	Method/platform	complex
1756	chr4	166079186	166102792	23606	14	chr4	166079155	166103263	24108	HiConf	Variation_31203	chr4	166081633	166105504	23871	Perry et al. (2008)	Agilent Custom CGH Arrays	
1757	chr4	166220551	166224275	3724	42	chr4	166220520	166224342	3822	HiConf								
100291						chr4	166383690	166431049	47359	single								
1758						chr4	167186506	167189887	3381	single								
1759	chr4	167519639	167520276	637	28	chr4	167519608	167520294	686	single								
1760	chr4	168112442	168127111	14669	3	chr4	168112410	168129070	16660	single								
100292	chr4	168258927	168317188	58261	5	chr4	168258896	168318357	59461	single	Variation_22948	chr4	168128702	168260370	131668	Korbel et al. (2007)	Paired End Mapping	
1762	chr4	168343672	168351610	7938	36	chr4	168343641	168351775	8134	HiConf	Variation_22712	chr4	168345443	168352348	6905	Korbel et al. (2007)	Paired End Mapping	
1763	chr4	168557116	168558586	1470	3	chr4	168557085	168558604	1519	single	Variation_5380	chr4	168475112	168730333	255221	Simon-Sanchez et al. (2007)	Illumina HumanHap300 BeadChip	
1764	chr4	168712348	168722295	9947	2	chr4	168712317	168722509	10192	HiConf	Variation_4421	chr4	168625507	168799583	174076	Wong et al. (2007)	BAC Array CGH	
1765	chr4	169025164	169033739	8575	4	chr4	169025133	169033904	8771	HiConf	Variation_2556	chr4	169031756	169334003	302247	Redon et al. (2006)	Affymetrix 500K EA SNP Mapping Array	
100293	chr4	169049803	169081860	32057	4	chr4	169049756	169082463	32707	HiConf	Variation_38903	chr4	169055017	169080681	25664	McCarroll et al. (2008)	Affymetrix Human SNP Array 6.0	
1767	chr4	169353611	169354836	1225	44	chr4	169353580	169354854	1274	HiConf	Variation_44140	chr4	169353392	169354991	1599	Bentley et al. (2008)	Illumina DNA sequencing	
1768	chr4	170523682	170524319	637	28	chr4	170523651	170524337	686	single	Variation_44141	chr4	170523423	170524978	1555	Bentley et al. (2008)	Illumina DNA sequencing	
1769	chr4	170578073	170581943	3870	1	chr4	170578041	170582010	3969	single								
1770	chr4	171504123	171510885	6762	8	chr4	171504092	171511001	6909	HiConf	Variation_32665	chr4	171504269	171510055	5786	Perry et al. (2008)	Agilent Custom CGH Arrays	
1771	chr4	172610912	172615721	4809	63	chr4	172610071	172615804	5733	HiConf	Variation_39405	chr4	172610986	172616000	5014	Wheeler et al. (2008)	Sequencing	
1772	chr4	172883914	172888179	4265	8	chr4	172883883	172888244	4361	HiConf								
1773	chr4	173225079	173229609	4530	50	chr4	173222179	173229774	7595	HiConf	Variation_37434	chr4	173225215	173229508	4293	Cooper et al. (2008)	Illumina Human 1M BeadChip	
1774	chr4	173661446	173667865	6419	46	chr4	173661415	173667981	6566	HiConf	Variation_31206	chr4	173662046	173666100	4054	Perry et al. (2008)	Agilent Custom CGH Arrays	y
100294	chr4	174322306	174355311	33005	7	chr4	174315835	174356088	40253	single								
1775	chr4	175862112	175863408	1296	21	chr4	175862103	175863426	1323	HiConf	Variation_39406	chr4	175861741	175863545	1804	Wheeler et al. (2008)	Sequencing	
1776	chr4	176357230	176357338	108	83	chr4	176357199	176357346	147	HiConf	Variation_12588	chr4	176357120	176357508	388	Mills et al. (2006)	Sequence trace read mapping	
1777	chr4	176499820	176501535	1715	28	chr4	176499789	176501553	1764	HiConf								
1778	chr4	176666371	176670193	3822	5	chr4	176666340	176670260	3920	HiConf	Variation_44145	chr4	176666305	176670144	3839	Bentley et al. (2008)	Illumina DNA sequencing	
1779	chr4	178039956	178040723	767	2	chr4	178039908	178040741	833	single								y
1780	chr4	178088547	178089380	833	68	chr4	178088516	178089398	882	HiConf	Variation_44146	chr4	178088090	178089243	1153	Bentley et al. (2008)	Illumina DNA sequencing	
1781	chr4	178289251	178294886	5635	7	chr4	178289220	178295002	5782	HiConf								
1782	chr4	178612308	178623431	11123	7	chr4	178612277	178623645	11368	HiConf	Variation_38538	chr4	178612788	178623504	10716	McCarroll et al. (2008)	Affymetrix Human SNP Array 6.0	
1783	chr4	178704379	178724910	20531	3	chr4	178704348	178725320	20972	HiConf	Variation_22894	chr4	178703409	178725756	22347	Korbel et al. (2007)	Paired End Mapping	
100295	chr4	178823572	178848512	24940	5	chr4	178823541	178848898	25357	HiConf	Variation_37435	chr4	178831040	178848075	17035	Cooper et al. (2008)	Illumina Human 1M BeadChip	
1785	chr4	179120977	179129748	8771	8	chr4	179120946	179129913	8967	HiConf	Variation_10080	chr4	179120182	179128405	8223	Wang et al. (2007)	Illumina HumanHap550 BeadChip	
1786	chr4	179280570	179281020	450	13	chr4	179280539	179281029	490	single								
1787	chr4	179365236	179372018	6782	5	chr4	179351246	179372414	21168	single								
1788	chr4	179476815	179483773	6958	46	chr4	179476784	179483889	7105	HiConf	Variation_6359	chr4	179477633	179483425	5792	Mills et al. (2006)	Sequence trace read mapping	
1789	chr4	179582900	179583880	980	4	chr4	179582869	179583898	1029	single								
1790	chr4	179770276	179771844	1568	4	chr4	179770245	179771862	1617	HiConf								
1791	chr4	180009886	180010572	686	2	chr4	180009855	180010590	735	single								y
100296	chr4	180095701	180123314	27613	12	chr4	180061893	180124491	62598	HiConf								
1793	chr4	180821865	180822265	400	9	chr4	180821834	180822275	441	single								
1794	chr4	181372135	181375565	3430	21	chr4	181372104	181375632	3528	HiConf	Variation_6360	chr4	181372110	181375637	3527	Mills et al. (2006)	Sequence trace read mapping	
1795	chr4	181507571	181511197	3626	8	chr4	181507540	181511264	3724	HiConf								
1796	chr4	181724004	181726013	2009	38	chr4	181723973	181726031	2058	single								
1797	chr4	181992965	181993455	490	17	chr4	181992934	181993473	539	single								
1798	chr4	182093415	182108703	15288	2	chr4	182093384	182109015	15631	single								
1799	chr4	182293754	182293839	85	34	chr4	182293745	182293843	98	single	Variation_40711	chr4	182293553	182294123	570	Wheeler et al. (2008)	Sequencing	y
1800	chr4	182381388	182411964	30576	9	chr4	182381357	182412570	31213	HiConf	Variation_36233	chr4	182382103	182405490	23387	Kidd et al. (2008)	Paired End Mapping	
1801	chr4	183209978	183217524	7546	1	chr4	183209947	183217689	7742	single								
1802	chr4	184548952	184551990	3038	19	chr4	184548921	184552057	3136	HiConf	Variation_36238	chr4	184534951	184553345	18394	Kidd et al. (2008)	Paired End Mapping	
1803	chr4	184911111	184912483	1372	11	chr4	184911080	184912501	1421	single	Variation_36239	chr4	184890647	184924535	33888	Kidd et al. (2008)	Paired End Mapping	
1804	chr4	185008033	185015530	7497	20	chr4	185008002	185015695	7693	single								
1805	chr4	185222261	185226437	4176	7	chr4	185222230	185226493	4263	HiConf	Variation_38542	chr4	185223006	185226185	3179	McCarroll et al. (2008)	Affymetrix Human SNP Array 6.0	
1806	chr4	185865141	185865249	108	24	chr4	185865110	185865257	147	single								
1807	chr4	186123224	186134146	10922	5	chr4	186123193	186135933	12740	HiConf								
1808	chr4	186678345	186681236	2891	16	chr4	186678314	186681303	2989	HiConf	Variation_43469	chr4	186678593	186681179	2586	Wang et al. (2008)	Illumina DNA sequencing	
1809	chr4	186706422	186707647	1225	7	chr4	186706391	186707665	1274	HiConf	Variation_3530	chr4	186550119	186711690	161571	Redon et al. (2006)	BAC Array CGH	
1810	chr4	187192404	187192992	588	39	chr4	187192373	187193010	637	HiConf								
1811	chr4	187330554	187338130	7576	24	chr4	187330553	187338295	7742	HiConf	Variation_7436	chr4	187330558	187335607	5049	de Smith et al. (2007)	Agilent 185k CGH Arrays/Agilent Custom CGH Arrays	y
100299	chr4	187569047	187627839	58792	48	chr4	187551641	187628620	76979	HiConf	Variation_23067	chr4	187588626	187594832	6206	Korbel et al. (2007)	Paired End Mapping	y
1813	chr4	187637128	187644735	7607	30	chr4	187637097	187644741	7644	HiConf	Variation_39409	chr4	187644140	187657248	13108	Wheeler et al. (2008)	Sequencing	y
1814	chr4	187759971	187768007	8036	25	chr4	187759940	187768172	8232	single								
1815	chr4	187979540	187979747	207	41	chr4	187979509	187979754	245	single								
1816	chr4	188080872	188087389	6517	8	chr4	188080841	188087505	6664	HiConf	Variation_10083	chr4	188080720	188085069	4349	Wang et al. (2007)	Illumina HumanHap550 BeadChip	
1817	chr4	188089202	188089265	63	3	chr4	188089171	188089269	98	single	Variation_36242	chr4	188057585	188092237	34652	Kidd et al. (2008)	Paired End Mapping	
1818	chr4	188108361	188117181	8820	13	chr4	188108330	188117346	9016	HiConf	Variation_8467	chr4	188102829	188115159	12330	Pinto et al. (2007)	Affymetrix 500K SNP Mapping Array	
1820	chr4	188752270	188769077	16807	7	chr4	188752239	188769389	17150	HiConf	Variation_8468	chr4	188277014	189258459	981445	Pinto et al. (2007)	Affymetrix 500K SNP Mapping Array	
100301	chr4	189042588	189102596	60008	4	chr4	189042540	189103839	61299	HiConf	Variation_6369	chr4	189050065	189054891	4826	Mills et al. (2006)	Sequence trace read mapping	y
1821	chr4	189317632	189318563	931	32	chr4	189317601	189318581	980	HiConf	Variation_36250	chr4	189290157	189334909	44752	Kidd et al. (2008)	Paired End Mapping	y
1822	chr4	189343602	189388385	44783	39	chr4	189343571	189389043	45472	HiConf	Variation_27567	chr4	189355247	189355367	120	Levy et al. (2007)	Sequencing	y
100303	chr4	189418141	189499132	80991	43	chr4	189417904	189500567	82663	single	Variation_2562	chr4	189408040	189499240	91200	Redon et al. (2006)	Affymetrix 500K EA SNP Mapping Array	
1823	chr4	189661220	189671755	10535	26	chr4	189661189	189671969	10780	HiConf	Variation_32681	chr4	189661575	189668909	7334	Perry et al. (2008)	Agilent Custom CGH Arrays	
100304	chr4	189797881	189975800	177919	1	chr4	189797850	189979420	181570	single	Variation_0703	chr4	189868649	190029092	160443	Sharp et al. (2005)	BAC Array CGH	
1825	chr4	189982001	189982383	382	35	chr4	189981992	189982384	392	HiConf	Variation_46589	chr4	189981955	189982103	148	Bentley et al. (2008)	Illumina DNA sequencing	
1826	chr4	189988197	189989422	1225	6	chr4	189988166	189989440	1274	HiConf	Variation_0703	chr4	189868649	190029092	160443	Sharp et al. (2005)	BAC Array CGH	
1827	chr4	190120350	190120660	310	31	chr4	190120319	190120662	343	single	Variation_34439	chr4	188622325	190339381	1717056	Zogopoulos et al. (2007)	Affymetrix 500K and 100K SNP Mapping Arrays	
1828	chr4	190194291	190194354	63	75	chr4	190194260	190194358	98	HiConf	Variation_34439	chr4	188622325	190339381	1717056	Zogopoulos et al. (2007)	Affymetrix 500K and 100K SNP Mapping Arrays	
1829	chr4	190342820	190343070	250	25	chr4	190342779	190343073	294	HiConf	Variation_27586	chr4	190342789	190343165	376	Levy et al. (2007)	Sequencing	y
1830	chr4	190380148	190413453	33305	41	chr4	190380117	190413878	33761	HiConf	Variation_32683	chr4	190388873	190393509	4636	Perry et al. (2008)	Agilent Custom CGH Arrays	
1831	chr4	190428609	190437151	8542	8	chr4	190428578	190437300	8722	HiConf	Variation_39410	chr4	190428594	190437456	8862	Wheeler et al. (2008)	Sequencing	
1832						chr4	190437790	190441024	3234	single								
1833	chr4	190697913	190706635	8722	6	chr4	190697882	190706800	8918	HiConf	Variation_36260	chr4	190696994	190721913	24919	Kidd et al. (2008)	Paired End Mapping	
1834	chr4	190707201	190717954	10753	19	chr4	190707192	190718168	10976	single	Variation_46605	chr4	190708712	190708849	137	Bentley et al. (2008)	Illumina DNA sequencing	
1835	chr4	190805174	190823696	18522	19	chr4	190805143	190824057	18914	HiConf	Variation_46610	chr4	190809656	190809834	178	Bentley et al. (2008)	Illumina DNA sequencing	
1836	chr4	190843541	190847118	3577	33	chr4	190843510	190847185	3675	HiConf	Variation_6373	chr4	190843982	190847294	3312	Mills et al. (2006)	Sequence trace read mapping	y
1837	chr4	190855987	190922008	66021	29	chr4	190855956	190922057	66101	HiConf	Variation_6374	chr4	190918122	190920127	2005	Mills et al. (2006)	Sequence trace read mapping	
1838	chr4	190978144	191003768	25624	40	chr4	190978113	191003936	25823	HiConf	Variation_42906	chr4	190978387	190978963	576	Wang et al. (2008)	Illumina DNA sequencing	y

locus_id	chrom	start	end	length	Yoruba w/ event	putative chr	putative start	putative end	putative len	putative type	variation_id	DGV_chr	DGV_start	DGV_end	DGV_len	Reference	Method/platform	complex
1839	chr4	191006760	191007495	735	55	chr4	191006729	191007513	784	HiConf	Variation_36263	chr4	190999435	191014952	15517	Kidd et al. (2008)	Paired End Mapping	
1840	chr4	191033073	191057279	24206	1	chr4	191033042	191057787	24745	HiConf	Variation_4426	chr4	191030568	191118518	87950	Wong et al. (2007)	BAC Array CGH	
1841	chr4	191057935	191061640	3705	2	chr4	191057934	191061707	3773	single	Variation_4426	chr4	191030568	191118518	87950	Wong et al. (2007)	BAC Array CGH	y
1842	chr4	191181906	191260972	79066	31	chr4	191181708	191262607	80899	HiConf	Variation_32686	chr4	191180398	191246555	66157	Perry et al. (2008)	Agilent Custom CGH Arrays	
1843	chr5	191989	200417	8428	25	chr5	191958	200582	8624	HiConf	Variation_8470	chr5	81949	399636	317687	Pinto et al. (2007)	Affymetrix 500K SNP Mapping Array	y
1844	chr5	299495	341037	41542	68	chr5	299464	341555	42091	HiConf	Variation_32689	chr5	309320	321893	12573	Perry et al. (2008)	Agilent Custom CGH Arrays	
1845	chr5	341996	351092	9096	8	chr5	341996	351257	9261	HiConf	Variation_4228	chr5	350725	536960	186235	Wong et al. (2007)	BAC Array CGH	y
1846	chr5	369418	372309	2891	32	chr5	369387	372376	2989	single	Variation_4228	chr5	350725	536960	186235	Wong et al. (2007)	BAC Array CGH	y
1847	chr5	702275	714646	12371	12	chr5	702244	714837	12593	HiConf	Variation_27991	chr5	703503	704199	696	Levy et al. (2007)	Sequencing	
1848	chr5	733096	735154	2058	3	chr5	733065	735172	2107	single	Variation_10086	chr5	723745	738748	15003	Wang et al. (2007)	Illumina HumanHap550 BeadChip	y
1849	chr5	737604	900204	162600	31	chr5	737573	901772	164199	HiConf	Variation_32692	chr5	732314	904042	171728	Perry et al. (2008)	Agilent Custom CGH Arrays	
1850	chr5	909229	911897	2668	46	chr5	907358	911964	4606	HiConf	Variation_7506	chr5	895229	916851	21622	de Smith et al. (2007)	Agilent 185k CGH Arrays/Agilent Custom CGH Arrays	
1851	chr5	931546	933604	2058	4	chr5	931515	933622	2107	single	Variation_4429	chr5	898120	1019970	121850	Wong et al. (2007)	BAC Array CGH	
1852	chr5	935515	936054	539	12	chr5	935484	936072	588	single	Variation_4429	chr5	898120	1019970	121850	Wong et al. (2007)	BAC Array CGH	
1853	chr5	1028860	1029260	400	45	chr5	1028829	1029270	441	single	Variation_2081	chr5	1024509	1248692	224183	Locke et al. (2006)	BAC Array CGH	
1854	chr5	1223470	1223848	378	49	chr5	1223457	1223849	392	HiConf	Variation_33405	chr5	1223439	1223854	415	Perry et al. (2008)	Agilent Custom CGH Arrays	
1855	chr5	1458345	1469860	11515	6	chr5	1458314	1470074	11760	single								
1856	chr5	1619996	1641311	21315	24	chr5	1619965	1641721	21756	HiConf	Variation_23280	chr5	1617675	1677351	59676	Levy et al. (2007)	Illumina HumanHap650Y BeadChip	y
1857	chr5	1664537	1694581	30044	26	chr5	1664506	1694935	30429	HiConf	Variation_0967	chr5	1680164	1681819	1655	Hinds et al. (2005)	Oligo arrays	
1858	chr5	1828785	1829765	980	27	chr5	1828754	1829783	1029	single								
1859	chr5	1919533	1922458	2925	2	chr5	1919502	1929253	9751	HiConf								
1860	chr5	2543450	2544234	784	48	chr5	2543419	2544252	833	HiConf								
1861	chr5	2766155	2768213	2058	7	chr5	2766124	2768231	2107	single								
1862	chr5	2872681	2874102	1421	12	chr5	2872650	2874120	1470	single								
100311	chr5	3409329	3456393	47064	8	chr5	3409298	3472925	63627	single								
1863	chr5	3710238	3713521	3283	29	chr5	3710207	3713588	3381	HiConf	Variation_37436	chr5	3709532	3713453	3921	Cooper et al. (2008)	Illumina Human 1M BeadChip	
1864	chr5	4188674	4189997	1323	5	chr5	4188643	4190015	1372	single								
1865	chr5	4896185	4897067	882	10	chr5	4896154	4897085	931	single								
1866	chr5	4953025	4954201	1176	3	chr5	4952994	4954219	1225	single								
1867	chr5	5160785	5162892	2107	6	chr5	5160754	5162910	2156	HiConf	Variation_43420	chr5	5160806	5162956	2150	Wang et al. (2008)	Illumina DNA sequencing	
100312	chr5	5338423	5378135	39712	14	chr5	5328212	5379123	50911	HiConf								
1869	chr5	5545484	5554031	8547	4	chr5	5545453	5554175	8722	HiConf	Variation_3540	chr5	5500098	5817620	317522	Redon et al. (2006)	BAC Array CGH	
1870	chr5	5554402	5565623	11221	4	chr5	5554371	5565837	11466	HiConf	Variation_3540	chr5	5500098	5817620	317522	Redon et al. (2006)	BAC Array CGH	
1871	chr5	5732045	5734232	2187	5	chr5	5732045	5734250	2205	single	Variation_0968	chr5	5731704	5733983	2279	Hinds et al. (2005)	Oligo arrays	
1872	chr5	5920930	5921314	384	2	chr5	5920499	5921332	833	single								
1873	chr5	6002164	6010298	8134	19	chr5	6002133	6010463	8330	HiConf								
100313	chr5	6602561	6663845	61284	21	chr5	6602530	6664907	62377	HiConf								
1876	chr5	7334866	7347202	12336	4	chr5	7334835	7347722	12887	HiConf								
100314	chr5	7405627	7455957	50330	2	chr5	7405469	7481958	76489	single								
1878	chr5	8164142	8174383	10241	1	chr5	8164111	8174597	10486	single								
1879	chr5	10326644	10328374	1730	42	chr5	10326628	10328392	1764	HiConf	Variation_23743	chr5	10326613	10328457	1844	Levy et al. (2007)	Sequencing	
1880	chr5	10581361	10584350	2989	8	chr5	10581330	10584417	3087	single	Variation_38583	chr5	10580501	10584996	4495	McCarroll et al. (2008)	Affymetrix Human SNP Array 6.0	
1881	chr5	11048674	11050046	1372	40	chr5	11048643	11050064	1421	single								
1882	chr5	12480405	12481434	1029	8	chr5	12480374	12481452	1078	single								
1883	chr5	12864241	12873140	8899	4	chr5	12864240	12873305	9065	HiConf	Variation_32700	chr5	12864144	12873198	9054	Perry et al. (2008)	Agilent Custom CGH Arrays	
1884	chr5	13106821	13112358	5537	2	chr5	13106790	13112474	5684	single								
1885	chr5	14400225	14400432	207	46	chr5	14400194	14400439	245	HiConf								
1886	chr5	14581917	14583485	1568	7	chr5	14581886	14583503	1617	HiConf								
1887	chr5	14674282	14692412	18130	18	chr5	14674251	14692773	18522	HiConf								
1888	chr5	15772568	15773842	1274	9	chr5	15772537	15773860	1323	HiConf	Variation_0975	chr5	15772456	15773910	1454	Hinds et al. (2005)	Oligo arrays	
1889	chr5	15968617	15988952	20335	2	chr5	15968586	15989362	20776	single								
1890	chr5	16061531	16071174	9643	2	chr5	16061490	16071388	9898	single								
1891	chr5	16203033	16203719	686	42	chr5	16203002	16203737	735	HiConf	Variation_12657	chr5	16203013	16203779	766	Mills et al. (2006)	Sequence trace read mapping	
1892	chr5	16942884	16943284	400	7	chr5	16942853	16943294	441	single	Variation_10899	chr5	16942500	16943218	718	Hinds et al. (2005)	Oligo arrays	
1893	chr5	17028242	17031378	3136	17	chr5	17028211	17031445	3234	single								
1894	chr5	17516674	17523926	7252	8	chr5	17516643	17524091	7448	HiConf	Variation_32704	chr5	17518865	17523994	5129	Perry et al. (2008)	Agilent Custom CGH Arrays	y
1895	chr5	17534971	17623420	88449	28	chr5	17534822	17625227	90405	HiConf	Variation_31221	chr5	17570684	17642455	71771	Perry et al. (2008)	Agilent Custom CGH Arrays	
1896	chr5	17643290	17708656	65366	9	chr5	17643259	17709997	66738	HiConf	Variation_37893	chr5	17644656	17698273	53617	McCarroll et al. (2008)	Affymetrix Human SNP Array 6.0	
1897	chr5	17744230	17755082	10852	13	chr5	17744199	17755469	11270	HiConf	Variation_31223	chr5	17745780	17748817	3037	Perry et al. (2008)	Agilent Custom CGH Arrays	
1898	chr5	18909128	18910332	1204	24	chr5	18909125	18910350	1225	HiConf	Variation_6382	chr5	18909070	18910604	1534	Mills et al. (2006)	Sequence trace read mapping	
1899	chr5	19258869	19267983	9114	7	chr5	19258838	19268148	9310	HiConf	Variation_44198	chr5	19258777	19267925	9148	Bentley et al. (2008)	Illumina DNA sequencing	
1900	chr5	19313014	19323157	10143	8	chr5	19312983	19323371	10388	HiConf	Variation_38604	chr5	19315328	19323214	7886	McCarroll et al. (2008)	Affymetrix Human SNP Array 6.0	y
1901	chr5	19411063	19412141	1078	58	chr5	19411032	19412159	1127	HiConf	Variation_0976	chr5	19411052	19412164	1112	Hinds et al. (2005)	Oligo arrays	
1902	chr5	20162723	20166741	4018	4	chr5	20162692	20166808	4116	HiConf								
1903	chr5	20454889	20464129	9240	30	chr5	20454438	20464973	10535	HiConf	Variation_32706	chr5	20457881	20462468	4587	Perry et al. (2008)	Agilent Custom CGH Arrays	
1904	chr5	20472960	20479949	6989	5	chr5	20472960	20480065	7105	HiConf	Variation_38605	chr5	20472958	20480059	7101	McCarroll et al. (2008)	Affymetrix Human SNP Array 6.0	
1905	chr5	20810219	20822998	12779	5	chr5	20810178	20823261	13083	HiConf								
1906	chr5	20868421	20870528	2107	21	chr5	20868390	20870546	2156	HiConf								
100316	chr5	21303174	21437532	134358	6	chr5	21303143	21440269	137126	HiConf	Variation_31224	chr5	21307127	21426744	119617	Perry et al. (2008)	Agilent Custom CGH Arrays	
1908	chr5	21486017	21488124	2107	60	chr5	21485986	21488142	2156	HiConf	Variation_7492	chr5	21485973	21487886	1913	de Smith et al. (2007)	Agilent 185k CGH Arrays/Agilent Custom CGH Arrays	
1909	chr5	21510223	21557275	47052	17	chr5	21510192	21558065	47873	HiConf	Variation_32710	chr5	21513711	21533107	19396	Perry et al. (2008)	Agilent Custom CGH Arrays	
1910	chr5	21646982	21650412	3430	22	chr5	21646951	21650479	3528	HiConf	Variation_32712	chr5	21647099	21650550	3451	Perry et al. (2008)	Agilent Custom CGH Arrays	
1911	chr5	21870226	21872921	2695	1	chr5	21870195	21872988	2793	single	Variation_32713	chr5	21870766	21872820	2054	Perry et al. (2008)	Agilent Custom CGH Arrays	
100317	chr5	21968030	22049762	81732	8	chr5	21967999	22051446	83447	HiConf	Variation_31226	chr5	21971258	22050370	79112	Perry et al. (2008)	Agilent Custom CGH Arrays	
1913	chr5	22160698	22166774	6076	13	chr5	22160667	22166890	6223	HiConf	Variation_31227	chr5	22161504	22167345	5841	Perry et al. (2008)	Agilent Custom CGH Arrays	
1914	chr5	22188677	22197007	8330	1	chr5	22188646	22197172	8526	single	Variation_3548	chr5	21814498	22199675	385177	Redon et al. (2006)	BAC Array CGH	
1915	chr5	22997961	23003004	5043	4	chr5	22997930	23003418	5488	HiConf								
1916	chr5	23084838	23086504	1666	1	chr5	23084807	23086522	1715	single								
1917	chr5	23542204	23554209	12005	3	chr5	23542173	23554472	12299	HiConf								
1918	chr5	24186260	24213504	27244	2	chr5	24186229	24214061	27832	single								
1919	chr5	24605357	24613344	7987	1	chr5	24605326	24613509	8183	single	Variation_2574	chr5	24564081	24613895	49814	Redon et al. (2006)	Affymetrix 500K EA SNP Mapping Array	
1920	chr5	24919153	24921456	2303	3	chr5	24919122	24921474	2352	HiConf								
1921	chr5	25107607	25109273	1666	2	chr5	25107576	25109291	1715	HiConf								
1922						chr5	25226205	25226254	49	single								y
1923	chr5	25857650	25858434	784	15	chr5	25857619	25858452	833	single								
1924	chr5	25925711	25928504	2793	35	chr5	25925680	25928571	2891	HiConf								

locus_id	chrom	start	end	length	Yoruba w/ event	putative chr	putative start	putative end	putative len	putative type	variation_id	DGV_chr	DGV_start	DGV_end	DGV_len	Reference	Method/platform	complex
1925	chr5	25945997	25948398	2401	2	chr5	25945966	25948416	2450	single								
1926	chr5	25972310	25972620	310	3	chr5	25972279	25972622	343	single								
1927	chr5	26027043	26029787	2744	3	chr5	26027012	26029854	2842	HiConf	Variation_38606	chr5	26027946	26034694	6748	McCarroll et al. (2008)	Affymetrix Human SNP Array 6.0	
1928	chr5	26033239	26038509	5270	3	chr5	26033235	26038625	5390	HiConf	Variation_6389	chr5	26031983	26035390	3407	Mills et al. (2006)	Sequence trace read mapping	
1929	chr5	26045614	26047329	1715	4	chr5	26045583	26047347	1764	HiConf								
1930	chr5	26062519	26064332	1813	3	chr5	26062488	26064350	1862	HiConf	Variation_9504	chr5	26049058	26064146	15088	Wang et al. (2007)	Illumina HumanHap550 BeadChip	
1931	chr5	26255775	26260283	4508	7	chr5	26255744	26260350	4606	single								
1932	chr5	26829320	26838042	8722	67	chr5	26829289	26838207	8918	HiConf	Variation_31228	chr5	26829858	26837469	7611	Perry et al. (2008)	Agilent Custom CGH Arrays	
1933	chr5	27212404	27214264	1860	2	chr5	27211293	27214331	3038	HiConf								
1934	chr5	28251888	28261002	9114	42	chr5	28251857	28261167	9310	HiConf	Variation_44203	chr5	28252416	28261058	8642	Bentley et al. (2008)	Illumina DNA sequencing	
1935	chr5	28281239	28289198	7959	15	chr5	28281208	28289293	8085	HiConf	Variation_36395	chr5	28280318	28313407	33089	Kidd et al. (2008)	Paired End Mapping	
1936	chr5	29470420	29491588	21168	3	chr5	29470389	29491998	21609	HiConf	Variation_10106	chr5	29462349	29497730	35381	Wang et al. (2007)	Illumina HumanHap550 BeadChip	
1937	chr5	31427137	31427627	490	17	chr5	31427106	31427645	539	HiConf	Variation_3550	chr5	31332034	31505885	173851	Redon et al. (2006)	BAC Array CGH	
1938	chr5	32563643	32565407	1764	3	chr5	32563612	32565425	1813	single	Variation_3552	chr5	32281118	32774401	493283	Redon et al. (2006)	BAC Array CGH	
1939	chr5	33147723	33147930	207	42	chr5	33147692	33147937	245	HiConf	Variation_46870	chr5	33147623	33148419	796	Bentley et al. (2008)	Illumina DNA sequencing	
1940	chr5	34110132	34120226	10094	5	chr5	34110101	34120440	10339	HiConf	Variation_4437	chr5	34081988	34278541	196553	Wong et al. (2007)	BAC Array CGH	
1941	chr5	34222195	34270950	48755	15	chr5	34222164	34271948	49784	HiConf	Variation_31231	chr5	34226739	34291585	64846	Perry et al. (2008)	Agilent Custom CGH Arrays	
1942	chr5	38008474	38010140	1666	13	chr5	38008443	38010158	1715	HiConf								
1943	chr5	39104310	39106662	2352	15	chr5	39104279	39106680	2401	single								
1944	chr5	39831029	39831429	400	17	chr5	39830998	39831439	441	single								
1945	chr5	40004809	40010253	5444	11	chr5	40004801	40010338	5537	HiConf	Variation_44207	chr5	40004655	40012423	7768	Bentley et al. (2008)	Illumina DNA sequencing	
1946	chr5	40377916	40387375	9459	10	chr5	40370782	40387687	16905	HiConf	Variation_38608	chr5	40371232	40387303	16071	McCarroll et al. (2008)	Affymetrix Human SNP Array 6.0	
1947	chr5	40847289	40847339	50	1	chr5	40847258	40847552	294	single								
1948	chr5	41936739	41940572	3833	17	chr5	41934421	41942947	8526	HiConf	Variation_6396	chr5	41937140	41945587	8447	Mills et al. (2006)	Sequence trace read mapping	
1949	chr5	42128002	42130746	2744	14	chr5	42127971	42130813	2842	HiConf								
1950	chr5	42664111	42666904	2793	45	chr5	42664080	42666971	2891	HiConf	Variation_43513	chr5	42664021	42667068	3047	Wang et al. (2008)	Illumina DNA sequencing	
1951	chr5	42986090	42989324	3234	8	chr5	42986059	42989391	3332	single								
1952	chr5	43274455	43277885	3430	2	chr5	43274424	43277952	3528	single								
100318	chr5	43522570	43590778	68208	1	chr5	43493307	43655718	162411	HiConf								
1954	chr5	44380238	44381169	931	6	chr5	44380207	44381187	980	HiConf								
1955	chr5	45040486	45049911	9425	11	chr5	45040335	45051213	10878	HiConf	Variation_39418	chr5	45040577	45050285	9708	Wheeler et al. (2008)	Sequencing	
1956	chr5	45163062	45163269	207	55	chr5	45163031	45163276	245	HiConf	Variation_46889	chr5	45162999	45163425	426	Bentley et al. (2008)	Illumina DNA sequencing	y
1957	chr5	45562853	45563539	686	43	chr5	45562822	45563557	735	HiConf	Variation_12681	chr5	45562930	45563429	499	Mills et al. (2006)	Sequence trace read mapping	
1958	chr5	45987046	46008312	21266	22	chr5	45987015	46008722	21707	HiConf	Variation_10114	chr5	45990384	46004619	14235	Wang et al. (2007)	Illumina HumanHap550 BeadChip	
1959	chr5	46030754	46044572	13818	24	chr5	46030723	46044835	14112	single	Variation_10113	chr5	45990384	46032659	42275	Wang et al. (2007)	Illumina HumanHap550 BeadChip	
1960	chr5	46136839	46153744	16905	8	chr5	46136808	46154105	17297	single								
1961	chr5	46201421	46220384	18963	16	chr5	46201390	46220745	19355	single								
1962	chr5	46237103	46303880	66777	24	chr5	46237062	46305221	68159	single	Variation_22611	chr5	46261806	46267788	5982	Korbel et al. (2007)	Paired End Mapping	
1963	chr5	46309123	46310348	1225	39	chr5	46309092	46310366	1274	single	Variation_38787	chr5	46306765	46309259	2494	McCarroll et al. (2008)	Affymetrix Human SNP Array 6.0	
1964	chr5	49976962	49985012	8050	2	chr5	49923822	49986297	62475	single	Variation_27948	chr5	49976771	49976983	212	Levy et al. (2007)	Sequencing	
1965	chr5	49997108	50019477	22369	6	chr5	49997077	50020009	22932	single	Variation_10115	chr5	49993978	50053481	59503	Wang et al. (2007)	Illumina HumanHap550 BeadChip	
100321	chr5	50086754	50132689	45935	1	chr5	50086723	50133640	46917	single	Variation_3554	chr5	49784109	50093939	309830	Redon et al. (2006)	BAC Array CGH	
1966	chr5	50253476	50269793	16317	22	chr5	50253445	50270105	16660	single								
1967	chr5	50270185	50271900	1715	21	chr5	50270154	50271918	1764	HiConf	Variation_44209	chr5	50270139	50275241	5102	Bentley et al. (2008)	Illumina DNA sequencing	
1968	chr5	51613226	51616656	3430	21	chr5	51613195	51616723	3528	HiConf								
1969	chr5	53089547	53093810	4263	4	chr5	53089516	53093877	4361	HiConf								
1970	chr5	54268683	54270643	1960	1	chr5	54268652	54270661	2009	single								
1971	chr5	54448562	54452629	4067	4	chr5	54448531	54452696	4165	HiConf	Variation_36418	chr5	54413754	54457644	43890	Kidd et al. (2008)	Paired End Mapping	
1972	chr5	55454777	55455757	980	15	chr5	55454746	55455775	1029	HiConf								y
1973	chr5	57359113	57369213	10100	25	chr5	57359082	57369421	10339	HiConf	Variation_33980	chr5	57359235	57369537	10302	Levy et al. (2007)	Sequencing	
1974	chr5	57991262	57995427	4165	24	chr5	57991231	57995494	4263	HiConf	Variation_6408	chr5	57991615	57995265	3650	Mills et al. (2006)	Sequence trace read mapping	
100322	chr5	59516595	59552084	35489	1	chr5	59476446	59553621	77175	single	Variation_7499	chr5	59525425	59531934	6509	de Smith et al. (2007)	Agilent 185k CGH Arrays/Agilent Custom CGH Arrays	
100323	chr5	59768346	59775056	6710	1	chr5	59733916	59775885	41969	single								y
1976	chr5	60037577	60039588	2011	41	chr5	60037569	60039970	2401	HiConf	Variation_39419	chr5	60037466	60039427	1961	Wheeler et al. (2008)	Sequencing	
1977	chr5	60164706	60168185	3479	4	chr5	60164675	60168252	3577	single	Variation_9019	chr5	60070232	60172383	102151	Pinto et al. (2007)	Affymetrix 500K SNP Mapping Array	
1978	chr5	60436705	60437930	1225	11	chr5	60436674	60437948	1274	HiConf	Variation_6409	chr5	60436674	60440257	3583	Mills et al. (2006)	Sequence trace read mapping	
1979	chr5	61142256	61143971	1715	4	chr5	61142225	61143989	1764	single								
1980	chr5	61187483	61197773	10290	1	chr5	61187452	61197987	10535	single								
100324	chr5	61545592	61587127	41535	5	chr5	61545397	61587954	42557	HiConf	Variation_31235	chr5	61547816	61586109	38293	Perry et al. (2008)	Agilent Custom CGH Arrays	
1982	chr5	61784058	61785038	980	27	chr5	61784027	61785056	1029	HiConf	Variation_33790	chr5	61784192	61784900	708	Perry et al. (2008)	Agilent Custom CGH Arrays	
1983	chr5	62042050	62050471	8421	1	chr5	62040836	62050636	9800	single								
1984	chr5	62506416	62508964	2548	7	chr5	62506385	62509031	2646	single								
1985	chr5	63734405	63736904	2499	7	chr5	63734374	63736971	2597	HiConf								
1986	chr5	65303777	65314900	11123	8	chr5	65303746	65315114	11368	HiConf	Variation_4446	chr5	65242231	65364579	122348	Wong et al. (2007)	BAC Array CGH	
1987	chr5	66249869	66250849	980	1	chr5	66249838	66250867	1029	single	Variation_47881	chr5	65828585	66250032	421447	Gusev et al. (2009)	SNP genotyping analysis	
1988	chr5	68865734	70169673	1303939	8	chr5	68865703	70196298	1330595	HiConf	Variation_37550	chr5	68899306	70033323	1134017	Cooper et al. (2008)	Illumina Human 1M BeadChip	y
1989	chr5	70196649	70687211	490562	29	chr5	70196641	70697225	500584	HiConf	Variation_37551	chr5	70327006	70685177	358171	Cooper et al. (2008)	Illumina Human 1M BeadChip	
1990	chr5	71208914	71209314	400	16	chr5	71208883	71209324	441	single								
1991	chr5	71634577	71642809	8232	1	chr5	71634546	71642974	8428	single								
1992	chr5	71644034	71662654	18620	1	chr5	71644003	71663015	19012	single								
100327	chr5	72759037	72785122	26085	1	chr5	72699880	72786879	86999	single								
1993	chr5	74294199	74297796	3597	7	chr5	74294168	74297843	3675	single	Variation_4455	chr5	74225251	74373700	148449	Wong et al. (2007)	BAC Array CGH	
1994	chr5	78145599	78147365	1766	12	chr5	78145568	78147381	1813	HiConf	Variation_7504	chr5	78145665	78147458	1793	de Smith et al. (2007)	Agilent 185k CGH Arrays/Agilent Custom CGH Arrays	
100328	chr5	78434479	78487546	53067	4	chr5	78434448	78488617	54169	single	Variation_36431	chr5	78461657	78482471	20814	Kidd et al. (2008)	Paired End Mapping	
1996	chr5	78500555	78522679	22124	1	chr5	78500524	78523064	22540	single								
1997	chr5	79673272	79694378	21106	3	chr5	79673241	79694703	21462	HiConf								
1998	chr5	79938607	79940322	1715	4	chr5	79938576	79940340	1764	HiConf	Variation_0065	chr5	79882591	80063088	180497	Iafrate et al. (2004)	BAC Array CGH	
1999	chr5	79981776	79983638	1862	48	chr5	79981745	79983656	1911	HiConf	Variation_32727	chr5	79982572	79983998	1426	Perry et al. (2008)	Agilent Custom CGH Arrays	
100329	chr5	80114857	80171974	57117	8	chr5	80106891	80186247	79356	HiConf								
100330	chr5	80708887	80800649	91762	13	chr5	80708856	80800903	92047	HiConf								
2001	chr5	82987044	82992826	5782	2	chr5	82987013	82992942	5929	HiConf								
100331	chr5	83792617	83823220	30603	4	chr5	83786620	83823933	37313	HiConf								
2003	chr5	83834793	83840610	5817	1	chr5	83834762	83840740	5978	single								
2004	chr5	83983633	83992226	8593	61	chr5	83983624	83992248	8624	HiConf	Variation_22791	chr5	83983452	83991751	8299	Korbel et al. (2007)	Paired End Mapping	
2005	chr5	84396676	84397705	1029	4	chr5	84396645	84397723	1078	single								
2006	chr5	85188810	85198267	9457	2	chr5	85188779	85198432	9653	single	Variation_5219	chr5	85034919	85205077	170158	Simon-Sanchez et al. (2007)	Illumina HumanHap300 BeadChip	

locus_id	chrom	start	end	length	Yoruba w/ event	putative chr	putative start	putative end	putative len	putative type	variation_id	DGV_chr	DGV_start	DGV_end	DGV_len	Reference	Method/platform	complex
2007	chr5	86151170	86153571	2401	20	chr5	86151139	86153589	2450	HiConf	Variation_38081	chr5	86151134	86154902	3768	McCarroll et al. (2008)	Affymetrix Human SNP Array 6.0	
2008	chr5	86281804	86282735	931	26	chr5	86281773	86282753	980	HiConf								
2009	chr5	87413704	87417722	4018	33	chr5	87413673	87417789	4116	HiConf	Variation_44215	chr5	87414638	87417819	3181	Bentley et al. (2008)	Illumina DNA sequencing	
2010	chr5	87764397	87775324	10927	3	chr5	87764366	87775538	11172	single								
100332	chr5	88200277	88240261	39984	1	chr5	88200246	88241063	40817	single								
2012	chr5	90535616	90537748	2132	44	chr5	90535610	90537766	2156	HiConf	Variation_44216	chr5	90535530	90537852	2322	Bentley et al. (2008)	Illumina DNA sequencing	
2013	chr5	90653535	90659513	5978	5	chr5	90653504	90659629	6125	HiConf	Variation_38616	chr5	90650176	90656821	6645	McCarroll et al. (2008)	Affymetrix Human SNP Array 6.0	
2014	chr5	90660248	90661032	784	6	chr5	90660217	90661050	833	HiConf								
2015	chr5	93929234	93932174	2940	18	chr5	93929203	93932241	3038	HiConf	Variation_32729	chr5	93929215	93931984	2769	Perry et al. (2008)	Agilent Custom CGH Arrays	
2016	chr5	95730964	95732189	1225	3	chr5	95730933	95732207	1274	single								
2017	chr5	97427197	97428477	1280	54	chr5	97421825	97428489	6664	HiConf	Variation_44217	chr5	97427322	97428513	1191	Bentley et al. (2008)	Illumina DNA sequencing	
100333	chr5	97462110	97574614	112504	3	chr5	97462079	97576910	114831	HiConf	Variation_9514	chr5	97473351	97583573	110222	Wang et al. (2007)	Illumina HumanHap550 BeadChip	
2021	chr5	98199927	98205856	5929	12	chr5	98199896	98205972	6076	single								
2022	chr5	98452326	98457471	5145	1	chr5	98452295	98457587	5292	single								
2023	chr5	98557333	98592615	35282	25	chr5	98557302	98593366	36064	HiConf								
2024	chr5	98742259	98743190	931	4	chr5	98742228	98743208	980	HiConf	Variation_0712	chr5	98625474	98814385	188911	Sharp et al. (2005)	BAC Array CGH	y
2025	chr5	98864199	98870759	6560	24	chr5	98864140	98870804	6664	HiConf	Variation_39427	chr5	98867788	98870805	3017	Wheeler et al. (2008)	Sequencing	
2026	chr5	99409884	99416548	6664	25	chr5	99409853	99416664	6811	HiConf	Variation_32735	chr5	99408997	99419746	10749	Perry et al. (2008)	Agilent Custom CGH Arrays	
2027	chr5	99542380	99548162	5782	10	chr5	99542349	99548278	5929	HiConf	Variation_22752	chr5	99541673	99549089	7416	Korbel et al. (2007)	Paired End Mapping	
2028	chr5	99754109	99755579	1470	24	chr5	99754078	99755597	1519	single	Variation_32737	chr5	99738184	99762102	23918	Perry et al. (2008)	Agilent Custom CGH Arrays	
100334	chr5	101183390	101246038	62648	8	chr5	101183359	101247329	63970	HiConf	Variation_10120	chr5	101197157	101234268	37111	Wang et al. (2007)	Illumina HumanHap550 BeadChip	
2030	chr5	101533001	101534701	1700	1	chr5	101521410	101538413	17003	single								
2031	chr5	103984671	103987366	2695	2	chr5	103984640	103987433	2793	HiConf	Variation_2590	chr5	103967573	104010013	42440	Redon et al. (2006)	Affymetrix 500K EA SNP Mapping Array	
2032	chr5	103987635	104003046	15411	2	chr5	103987629	104003358	15729	HiConf	Variation_2590	chr5	103967573	104010013	42440	Redon et al. (2006)	Affymetrix 500K EA SNP Mapping Array	
2033	chr5	105032536	105035427	2891	6	chr5	105032505	105035494	2989	single	Variation_30220	chr5	104978554	105035114	56560	Jakobsson et al. (2008)	Illumina HumanHap550 BeadChip	
2034	chr5	105922572	105923307	735	78	chr5	105922541	105923325	784	HiConf								
2035	chr5	106032721	106032945	224	1	chr5	106031762	106033379	1617	single								
2036	chr5	106352694	106357117	4423	73	chr5	106352663	106357171	4508	HiConf	Variation_43390	chr5	106352593	106354471	1878	Wang et al. (2008)	Illumina DNA sequencing	
2037	chr5	106527918	106532426	4508	2	chr5	106527887	106532493	4606	HiConf	Variation_3570	chr5	106519625	106579710	60085	Redon et al. (2006)	BAC Array CGH	
2038						chr5	108333488	108333537	49	single								
2039	chr5	108697099	108703812	6713	12	chr5	108697068	108703928	6860	single								
2040	chr5	108896333	108898097	1764	3	chr5	108896302	108898115	1813	single								
2041	chr5	109499082	109502855	3773	17	chr5	109499051	109502922	3871	HiConf								
100335	chr5	109967118	109984430	17312	8	chr5	109949729	109989198	39469	HiConf	Variation_9023	chr5	109793062	110093410	300348	Pinto et al. (2007)	Affymetrix 500K SNP Mapping Array	
2043	chr5	110933985	110938702	4717	11	chr5	110929018	110938916	9898	HiConf	Variation_10128	chr5	110933970	110938684	4714	Wang et al. (2007)	Illumina HumanHap550 BeadChip	
100336	chr5	112075058	112119088	44030	1	chr5	112060747	112120282	59535	single								
2044	chr5	112694764	112700056	5292	12	chr5	112694733	112700172	5439	HiConf								
100337	chr5	112858351	112873666	15315	1	chr5	112787760	112898059	110299	single								
2045	chr5	113595144	113596364	1220	22	chr5	113593344	113596431	3087	HiConf	Variation_6438	chr5	113594216	113596726	2510	Mills et al. (2006)	Sequence trace read mapping	
2046	chr5	114236353	114241228	4875	4	chr5	114236322	114246073	9751	single	Variation_38587	chr5	114235601	114238777	3176	McCarroll et al. (2008)	Affymetrix Human SNP Array 6.0	
2047	chr5	114281655	114287607	5952	29	chr5	114278168	114287772	9604	HiConf	Variation_22777	chr5	114282597	114290600	8003	Korbel et al. (2007)	Paired End Mapping	
2048	chr5	114959544	114993787	34243	7	chr5	114959513	114994499	34986	single								
100338	chr5	115162282	115213193	50911	3	chr5	115162251	115214240	51989	single								
100339	chr5	115619942	115653043	33101	7	chr5	115603863	115654015	50152	HiConf	Variation_38589	chr5	115624233	115653127	28894	McCarroll et al. (2008)	Affymetrix Human SNP Array 6.0	
2050	chr5	116168325	116176802	8477	4	chr5	116168294	116176967	8673	HiConf								
2051	chr5	116182437	116184054	1617	2	chr5	116182406	116184072	1666	single								
2052	chr5	116207476	116211641	4165	3	chr5	116207445	116211708	4263	HiConf								
2053	chr5	116213993	116216100	2107	7	chr5	116213962	116216118	2156	HiConf	Variation_6442	chr5	116213646	116216241	2595	Mills et al. (2006)	Sequence trace read mapping	
2054	chr5	117415081	117422186	7105	34	chr5	117415050	117422302	7252	HiConf	Variation_44181	chr5	117414987	117421796	6809	Bentley et al. (2008)	Illumina DNA sequencing	
2055	chr5	117891761	117912039	20278	6	chr5	117891673	117912449	20776	single	Variation_2598	chr5	117813488	117892105	78617	Redon et al. (2006)	Affymetrix 500K EA SNP Mapping Array	
2056	chr5	119042602	119047719	5117	5	chr5	119042389	119058804	16415	single	Variation_42252	chr5	119042966	119043427	461	Wang et al. (2008)	Illumina DNA sequencing	y
2057	chr5	119408058	119412374	4316	72	chr5	119408027	119412388	4361	HiConf	Variation_44183	chr5	119408044	119410589	2545	Bentley et al. (2008)	Illumina DNA sequencing	
2058	chr5	119738955	119740376	1421	3	chr5	119738924	119740394	1470	HiConf								
2059	chr5	119938385	119938448	63	68	chr5	119938354	119938452	98	single								
2060	chr5	120677403	120680000	2597	19	chr5	120677372	120680067	2695	HiConf	Variation_7470	chr5	120408424	120876659	468235	de Smith et al. (2007)	Agilent 185k CGH Arrays/Agilent Custom CGH Arrays	
2061	chr5	122767694	122778033	10339	3	chr5	122767663	122778247	10584	single	Variation_4477	chr5	122604462	122780319	175857	Wong et al. (2007)	BAC Array CGH	
2062	chr5	122836392	122857707	21315	3	chr5	122836361	122858117	21756	HiConf								
2063	chr5	123910864	123914343	3479	15	chr5	123910833	123914410	3577	HiConf								
100340	chr5	124077071	124106529	29458	1	chr5	124068760	124114771	46011	single								
2064	chr5	127364041	127364678	637	52	chr5	127364010	127364696	686	HiConf	Variation_12756	chr5	127363901	127364828	927	Mills et al. (2006)	Sequence trace read mapping	
2065	chr5	127434748	127439648	4900	56	chr5	127434717	127439764	5047	HiConf	Variation_23030	chr5	127434772	127440351	5579	Korbel et al. (2007)	Paired End Mapping	
2066	chr5	127804796	127805874	1078	4	chr5	127804765	127805892	1127	single								
2067	chr5	128326663	128331053	4390	1	chr5	128314022	128338277	24255	single								
2068	chr5	128910089	128910873	784	25	chr5	128910058	128910891	833	HiConf	Variation_4478	chr5	128861056	129039905	178849	Wong et al. (2007)	BAC Array CGH	
2069	chr5	129642198	129645922	3724	4	chr5	129642167	129645989	3822	HiConf	Variation_22593	chr5	129641581	129647384	5803	Korbel et al. (2007)	Paired End Mapping	
2070	chr5	133063868	133063976	108	30	chr5	133063837	133063984	147	single								y
2071	chr5	133162897	133164269	1372	49	chr5	133162866	133164287	1421	HiConf								y
2072	chr5	134287006	134292082	5076	47	chr5	134286975	134292414	5439	HiConf	Variation_4479	chr5	134188786	134347887	159101	Wong et al. (2007)	BAC Array CGH	
2073	chr5	135128924	135164694	35770	21	chr5	135128893	135165398	36505	HiConf								
2074	chr5	136159100	136160913	1813	2	chr5	136159069	136160931	1862	single								
2075	chr5	136349122	136351327	2205	3	chr5	136349091	136351345	2254	HiConf								
2076	chr5	136479805	136489507	9702	1	chr5	136479774	136489721	9947	single								
2077	chr5	136883173	136884006	833	38	chr5	136883142	136884024	882	HiConf	Variation_23750	chr5	136882557	136884273	1716	Levy et al. (2007)	Sequencing	
2078						chr5	136885053	136919843	34790	single								
2079	chr5	137041590	137042472	882	6	chr5	137041559	137042490	931	single								
2080	chr5	139248452	139251098	2646	5	chr5	139248421	139251165	2744	single								
100341	chr5	140140196	140223453	83257	9	chr5	140139805	140223815	84010	single	Variation_7473	chr5	140197260	140219007	21747	de Smith et al. (2007)	Agilent 185k CGH Arrays/Agilent Custom CGH Arrays	
2082	chr5	140512358	140516915	4557	19	chr5	140512327	140516982	4655	HiConf	Variation_32749	chr5	140512422	140516955	4533	Perry et al. (2008)	Agilent Custom CGH Arrays	
2083						chr5	140517031	140517080	49	single								
2084	chr5	140534898	140539945	5047	18	chr5	140534867	140540061	5194	HiConf	Variation_31251	chr5	140534999	140541206	6207	Perry et al. (2008)	Agilent Custom CGH Arrays	
2085	chr5	141418613	141420132	1519	20	chr5	141418582	141420150	1568	HiConf								
2086	chr5	141996421	142004212	7791	11	chr5	141996390	142004377	7987	HiConf	Variation_38592	chr5	141998202	142000197	1995	McCarroll et al. (2008)	Affymetrix Human SNP Array 6.0	
2087	chr5	142250829	142253773	2944	10	chr5	142250798	142253836	3038	HiConf								
2088	chr5	142910859	142915073	4214	1	chr5	142910828	142915140	4312	single								
2089	chr5	143386453	143389442	2989	5	chr5	143386422	143389509	3087	HiConf	Variation_38593	chr5	143386880	143390529	3649	McCarroll et al. (2008)	Affymetrix Human SNP Array 6.0	
2090	chr5	145313357	145315058	1701	1	chr5	145312759	145322216	9457	single								

locus_id	chrom	start	end	length	Yoruba w/ event	putative chr	putative start	putative end	putative len	putative type	variation_id	DGV_chr	DGV_start	DGV_end	DGV_len	Reference	Method/platform	complex
2091	chr5	145772851	145772959	108	45	chr5	145772820	145772967	147	HiConf								
2092	chr5	145773047	145773110	63	50	chr5	145773016	145773114	98	HiConf								
2093	chr5	145812002	145825909	13907	2	chr5	145811971	145826181	14210	single								
2094	chr5	146465613	146466103	490	11	chr5	146465582	146466121	539	HiConf	Variation_2605	chr5	146410036	146519693	109657	Redon et al. (2006)	Affymetrix 500K EA SNP Mapping Array	
2095	chr5	146603009	146603171	162	44	chr5	146602978	146603174	196	HiConf								
2096	chr5	146821059	146823705	2646	7	chr5	146821028	146823772	2744	HiConf								
2097	chr5	147307237	147317968	10731	6	chr5	147307206	147318182	10976	HiConf	Variation_37740	chr5	147307539	147318106	10567	McCarroll et al. (2008)	Affymetrix Human SNP Array 6.0	y
2098	chr5	147533274	147534842	1568	79	chr5	147533243	147534860	1617	HiConf								
2099	chr5	147722855	147729676	6821	1	chr5	147722824	147729831	7007	single								
2100	chr5	148991367	148992886	1519	8	chr5	148991336	148992904	1568	single								
2101	chr5	149291982	149292189	207	14	chr5	149291951	149292196	245	single								
2102	chr5	150156881	150161683	4802	25	chr5	150156850	150161799	4949	HiConf	Variation_32750	chr5	150157286	150161621	4335	Perry et al. (2008)	Agilent Custom CGH Arrays	
2103	chr5	150185203	150203774	18571	46	chr5	150185172	150204135	18963	HiConf	Variation_32751	chr5	150185218	150204151	18933	Perry et al. (2008)	Agilent Custom CGH Arrays	
2104	chr5	150770018	150772713	2695	12	chr5	150769987	150772780	2793	HiConf								
2105	chr5	150861158	150863363	2205	32	chr5	150861127	150863381	2254	HiConf								
2106	chr5	151229529	151234048	4519	70	chr5	151228823	151234164	5341	HiConf								
2107	chr5	151494924	151498844	3920	5	chr5	151494893	151498911	4018	HiConf	Variation_38594	chr5	151495579	151498544	2965	McCarroll et al. (2008)	Affymetrix Human SNP Array 6.0	
2108	chr5	151937138	151940726	3588	5	chr5	151936873	151940793	3920	single								
2109	chr5	152223554	152231933	8379	2	chr5	152223523	152232098	8575	single								
2110	chr5	152400542	152400992	450	25	chr5	152400511	152401001	490	HiConf	Variation_46792	chr5	152400334	152401293	959	Bentley et al. (2008)	Illumina DNA sequencing	
2111	chr5	152815502	152828900	13398	2	chr5	152808975	152829310	20335	HiConf								
2112	chr5	153282689	153283375	686	4	chr5	153282658	153283393	735	single	Variation_6458	chr5	153282632	153283695	1063	Mills et al. (2006)	Sequence trace read mapping	
2113	chr5	155058792	155063055	4263	2	chr5	155058761	155063122	4361	single								y
2114	chr5	155244502	155245090	588	83	chr5	155244471	155245108	637	HiConf	Variation_46795	chr5	155244364	155245152	788	Bentley et al. (2008)	Illumina DNA sequencing	
2115	chr5	155409779	155421254	11475	11	chr5	155409748	155432729	22981	HiConf	Variation_32754	chr5	155410984	155420819	9835	Perry et al. (2008)	Agilent Custom CGH Arrays	
2116	chr5	155509739	155523802	14063	14	chr5	155509708	155524065	14357	HiConf	Variation_32755	chr5	155510304	155524224	13920	Perry et al. (2008)	Agilent Custom CGH Arrays	
100344	chr5	157633669	157700701	67032	50	chr5	157633638	157702066	68428	HiConf								
2118	chr5	158828411	158829881	1470	3	chr5	158828380	158829899	1519	single								
2119	chr5	159854716	159856088	1372	5	chr5	159854685	159856106	1421	HiConf								y
2120	chr5	159994072	159998923	4851	17	chr5	159994041	159999039	4998	HiConf								
2121	chr5	160521618	160522439	821	50	chr5	160520448	160522457	2009	HiConf	Variation_3584	chr5	160477321	160626797	149476	Redon et al. (2006)	BAC Array CGH	
2122	chr5	161304479	161306880	2401	4	chr5	161304448	161306947	2499	HiConf								
100345	chr5	162771663	162889578	117915	2	chr5	162771557	162890774	119217	single								
2123	chr5	163103857	163116185	12328	5	chr5	163103826	163116566	12740	HiConf	Variation_38598	chr5	163106031	163113414	7383	McCarroll et al. (2008)	Affymetrix Human SNP Array 6.0	
2124	chr5	163598169	163598855	686	15	chr5	163598138	163598873	735	HiConf	Variation_44191	chr5	163597606	163599092	1486	Bentley et al. (2008)	Illumina DNA sequencing	
2125	chr5	163798544	163799216	672	2	chr5	163798499	163799234	735	single								
100346	chr5	163925318	163970202	44884	2	chr5	163925287	163971102	45815	single								
2126	chr5	164655001	164658284	3283	6	chr5	164654970	164658351	3381	HiConf								
2127	chr5	165770976	165782687	11711	13	chr5	165770945	165782901	11956	HiConf	Variation_37441	chr5	165770334	165782065	11731	Cooper et al. (2008)	Illumina Human 1M BeadChip	
2128	chr5	166791695	166792185	490	5	chr5	166791664	166792203	539	HiConf								
2129	chr5	167051052	167058696	7644	1	chr5	167051021	167058861	7840	single								
2130	chr5	168070350	168095144	24794	23	chr5	168070319	168095652	25333	HiConf								
2131	chr5	169087051	169092931	5880	3	chr5	169087020	169093047	6027	single								
2132	chr5	169332737	169332845	108	3	chr5	169332706	169332853	147	single								
2133	chr5	169400357	169400465	108	68	chr5	169400326	169400473	147	HiConf	Variation_12777	chr5	169400269	169400527	258	Mills et al. (2006)	Sequence trace read mapping	y
2134	chr5	170062592	170064601	2009	57	chr5	170062561	170064619	2058	HiConf	Variation_43386	chr5	170062304	170064159	1855	Wang et al. (2008)	Illumina DNA sequencing	
2135	chr5	172015527	172060066	44539	3	chr5	172015407	172060977	45570	HiConf	Variation_9524	chr5	172023755	172088612	64857	Wang et al. (2007)	Illumina HumanHap550 BeadChip	
2136	chr5	172061125	172094644	33519	5	chr5	172061124	172095424	34300	HiConf	Variation_9524	chr5	172023755	172088612	64857	Wang et al. (2007)	Illumina HumanHap550 BeadChip	
2137						chr5	172537355	172551222	13867	single								y
2138	chr5	172905942	172907287	1345	45	chr5	172905933	172907305	1372	HiConf	Variation_6468	chr5	172905621	172907360	1739	Mills et al. (2006)	Sequence trace read mapping	
2139	chr5	174062952	174064226	1274	43	chr5	174062921	174064244	1323	HiConf								
2140	chr5	174690936	174691867	931	19	chr5	174690905	174691885	980	single								
100349	chr5	174741558	174756635	15077	3	chr5	174714401	174789763	75362	HiConf								y
100350	chr5	175488464	175575435	86971	5	chr5	175488429	175577193	88764	HiConf	Variation_8502	chr5	175503757	175571000	67243	Pinto et al. (2007)	Affymetrix 500K SNP Mapping Array	
2144	chr5	176427300	176428721	1421	43	chr5	176427269	176428739	1470	HiConf	Variation_44194	chr5	176427644	176428913	1269	Bentley et al. (2008)	Illumina DNA sequencing	
100351	chr5	177014516	177074443	59927	6	chr5	177014485	177075637	61152	single	Variation_7487	chr5	177068866	177101111	32245	de Smith et al. (2007)	Agilent 185k CGH Arrays/Agilent Custom CGH Arrays	
2145	chr5	177340464	177358545	18081	16	chr5	177340433	177358906	18473	single								
2146	chr5	177443560	177456594	13034	36	chr5	177443529	177456857	13328	HiConf								
2147	chr5	178041846	178045721	3875	8	chr5	178038095	178045886	7791	HiConf	Variation_38090	chr5	178042581	178045769	3188	McCarroll et al. (2008)	Affymetrix Human SNP Array 6.0	
2148	chr5	178280823	178285377	4554	9	chr5	178280792	178285790	4998	HiConf								
2149	chr5	178573603	178575705	2102	24	chr5	178573567	178575723	2156	HiConf	Variation_46833	chr5	178573698	178573890	192	Bentley et al. (2008)	Illumina DNA sequencing	
2150	chr5	178664003	178665914	1911	17	chr5	178663972	178665932	1960	single	Variation_37744	chr5	178662194	178863912	201718	McCarroll et al. (2008)	Affymetrix Human SNP Array 6.0	
2151	chr5	178764747	178776556	11809	21	chr5	178764716	178776770	12054	HiConf	Variation_37744	chr5	178662194	178863912	201718	McCarroll et al. (2008)	Affymetrix Human SNP Array 6.0	
2152	chr5	178974173	178979955	5782	1	chr5	178974142	178980071	5929	single	Variation_4481	chr5	178971818	179108364	136546	Wong et al. (2007)	BAC Array CGH	y
2153	chr5	179963889	179976027	12138	4	chr5	179961835	179976290	14455	HiConf								y
100353	chr5	180273212	180472252	199040	48	chr5	180273181	180472709	199528	HiConf	Variation_2613	chr5	180311387	180421549	110162	Redon et al. (2006)	Affymetrix 500K EA SNP Mapping Array	
2155	chr5	180493271	180496260	2989	24	chr5	180493240	180496327	3087	HiConf	Variation_44196	chr5	180493081	180495752	2671	Bentley et al. (2008)	Illumina DNA sequencing	
2156	chr5	180527130	180528943	1813	5	chr5	180527099	180528961	1862	single	Variation_6473	chr5	180523346	180532903	9557	Mills et al. (2006)	Sequence trace read mapping	
100354	chr5	180631598	180821914	190316	11	chr5	180631567	180825803	194236	HiConf	Variation_32766	chr5	180715433	180820892	105459	Perry et al. (2008)	Agilent Custom CGH Arrays	
2158	chr6	23162	101682	78520	4	chr6	5464	103660	98196	HiConf	Variation_32767	chr6	21709	63238	41529	Perry et al. (2008)	Agilent Custom CGH Arrays	
100356	chr6	202206	324555	122349	5	chr6	202175	326929	124754	HiConf	Variation_37847	chr6	202353	326149	123796	McCarroll et al. (2008)	Affymetrix Human SNP Array 6.0	y
100357	chr6	409255	535164	125909	41	chr6	409224	536428	127204	HiConf	Variation_28313	chr6	464327	465280	953	Levy et al. (2007)	Sequencing	
2160	chr6	611447	612507	1060	62	chr6	611447	612525	1078	HiConf	Variation_44263	chr6	611378	612821	1443	Bentley et al. (2008)	Illumina DNA sequencing	y
2161	chr6	719180	719630	450	48	chr6	719149	719639	490	HiConf	Variation_3593	chr6	5001	720274	715273	Redon et al. (2006)	BAC Array CGH	
2162	chr6	773227	784252	11025	24	chr6	773196	784466	11270	HiConf								
2163	chr6	1189041	1189401	360	41	chr6	1189010	1189402	392	single	Variation_4484	chr6	1014903	1194050	179147	Wong et al. (2007)	BAC Array CGH	
100358						chr6	1697924	1762825	64901	single								
2164	chr6	1850590	1850850	260	12	chr6	1850559	1850853	294	single								
2165	chr6	2152430	2153214	784	10	chr6	2152399	2153232	833	single								y
2166	chr6	2447655	2451918	4263	14	chr6	2447624	2451985	4361	single								
2167	chr6	2513217	2516549	3332	22	chr6	2513186	2516616	3430	HiConf	Variation_7554	chr6	2472402	2569090	96688	de Smith et al. (2007)	Agilent 185k CGH Arrays/Agilent Custom CGH Arrays	
2168	chr6	2630918	2654131	23213	25	chr6	2630884	2654600	23716	HiConf								y
2169	chr6	3104747	3123574	18827	22	chr6	3104322	3124069	19747	single	Variation_43681	chr6	3101678	3125395	23717	Wang et al. (2008)	Illumina DNA sequencing	
2170	chr6	3126599	3127579	980	41	chr6	3126568	3127597	1029	HiConf								
2171	chr6	3140564	3140771	207	47	chr6	3140533	3140778	245	HiConf								
2172	chr6	3163251	3169033	5782	3	chr6	3163220	3169149	5929	single								

locus_id	chrom	start	end	length	Yoruba w/ event	putative chr	putative start	putative end	putative len	putative type	variation_id	DGV_chr	DGV_start	DGV_end	DGV_len	Reference	Method/platform	complex
2173	chr6	3821522	3822595	1073	2	chr6	3821143	3822613	1470	HiConf								
2174	chr6	3830183	3832199	2016	2	chr6	3830110	3832217	2107	single								
2175	chr6	4115592	4119535	3943	3	chr6	4115584	4119602	4018	HiConf	Variation_38641	chr6	4115525	4119295	3770	McCarroll et al. (2008)	Affymetrix Human SNP Array 6.0	
2176	chr6	4199650	4200140	490	26	chr6	4199619	4200158	539	single	Variation_5390	chr6	4045332	4322395	277063	Simon-Sanchez et al. (2007)	Illumina HumanHap300 BeadChip	
100359	chr6	4471896	4521753	49857	13	chr6	4464905	4522946	58041	HiConf								
2178						chr6	5732290	5734446	2156	single								y
2179	chr6	5843551	5843713	162	28	chr6	5843520	5843716	196	single	Variation_43166	chr6	5843414	5844235	821	Wang et al. (2008)	Illumina DNA sequencing	
2180	chr6	6199732	6202035	2303	3	chr6	6199701	6202053	2352	single								
2181	chr6	6471290	6489234	17944	24	chr6	6471259	6489683	18424	HiConf								
2182	chr6	8109262	8115534	6272	4	chr6	8109231	8115650	6419	HiConf								
2183	chr6	8282428	8286446	4018	18	chr6	8282397	8286513	4116	HiConf	Variation_36570	chr6	8274177	8318881	44704	Kidd et al. (2008)	Paired End Mapping	
2184	chr6	9381841	9382772	931	59	chr6	9381810	9382790	980	HiConf	Variation_8507	chr6	9372877	9436505	63628	Pinto et al. (2007)	Affymetrix 500K SNP Mapping Array	
2185	chr6	9870175	9873948	3773	1	chr6	9870144	9874015	3871	single								
2186	chr6	10171770	10177356	5586	4	chr6	10171739	10177472	5733	HiConf	Variation_38621	chr6	10175992	10177866	1874	McCarroll et al. (2008)	Affymetrix Human SNP Array 6.0	
2187	chr6	10204603	10212979	8376	2	chr6	10204569	10213144	8575	HiConf	Variation_10140	chr6	10205002	10212993	7991	Wang et al. (2007)	Illumina HumanHap550 BeadChip	
2188	chr6	10848411	10853033	4622	12	chr6	10848380	10853084	4704	HiConf								y
2189	chr6	11588409	11588859	450	32	chr6	11588378	11588868	490	single								
2190	chr6	11589536	11590810	1274	16	chr6	11589505	11590828	1323	single								
2191	chr6	12797337	12800375	3038	7	chr6	12797306	12800442	3136	single								
2192	chr6	12800620	12805961	5341	7	chr6	12800589	12806077	5488	single								
2193	chr6	12806647	12810567	3920	4	chr6	12806616	12810634	4018	single								
100361	chr6	13612886	13621454	8568	1	chr6	13596790	13734039	137249	single	Variation_36461	chr6	13606865	13613457	6592	Kidd et al. (2008)	Paired End Mapping	
2194	chr6	14847595	14848869	1274	9	chr6	14847564	14848887	1323	HiConf								
2195	chr6	14853426	14853588	162	46	chr6	14853395	14853591	196	HiConf	Variation_47014	chr6	14853263	14853677	414	Bentley et al. (2008)	Illumina DNA sequencing	
100362	chr6	15767129	15826762	59633	1	chr6	15767098	15827981	60883	single	Variation_2620	chr6	15776846	15833457	56611	Redon et al. (2006)	Affymetrix 500K EA SNP Mapping Array	
2197	chr6	17486147	17488762	2615	18	chr6	17486116	17489154	3038	single								
2198	chr6	17547544	17549455	1911	12	chr6	17547513	17549473	1960	HiConf	Variation_44243	chr6	17546270	17549948	3678	Bentley et al. (2008)	Illumina DNA sequencing	
2199	chr6	18214826	18221049	6223	7	chr6	18214795	18221165	6370	HiConf	Variation_44244	chr6	18214911	18220875	5964	Bentley et al. (2008)	Illumina DNA sequencing	
2200	chr6	18510217	18510737	520	57	chr6	18510216	18510755	539	HiConf	Variation_47077	chr6	18510071	18510847	776	Bentley et al. (2008)	Illumina DNA sequencing	
2201	chr6	18917829	18918417	588	10	chr6	18917798	18918435	637	single								
2202	chr6	19148717	19157047	8330	18	chr6	19148686	19157212	8526	HiConf	Variation_37442	chr6	19149533	19157202	7669	Cooper et al. (2008)	Illumina Human 1M BeadChip	
2203	chr6	19159252	19159742	490	21	chr6	19159221	19159760	539	single	Variation_36496	chr6	19144515	19161962	17447	Kidd et al. (2008)	Paired End Mapping	
2204	chr6	19793677	19794292	615	51	chr6	19793673	19794310	637	HiConf	Variation_28232	chr6	19793556	19794485	929	Levy et al. (2007)	Sequencing	
100363	chr6	19903097	19970325	67228	1	chr6	19903066	19971715	68649	single	Variation_38636	chr6	19924839	19927883	3044	McCarroll et al. (2008)	Affymetrix Human SNP Array 6.0	
2205	chr6	20281548	20286889	5341	2	chr6	20281517	20287005	5488	HiConf								
2206	chr6	21607322	21612437	5115	3	chr6	21607261	21612553	5292	HiConf	Variation_38637	chr6	21609666	21611713	2047	McCarroll et al. (2008)	Affymetrix Human SNP Array 6.0	y
2207	chr6	22159326	22161972	2646	61	chr6	22159295	22162039	2744	HiConf	Variation_12814	chr6	22160749	22161706	957	Mills et al. (2006)	Sequence trace read mapping	
100364	chr6	22398582	22422325	23743	2	chr6	22374846	22473728	98882	single								
2208	chr6	23568223	23573515	5292	2	chr6	23568192	23573631	5439	HiConf								
2209	chr6	23833999	23862285	28286	20	chr6	23833968	23862437	28469	HiConf	Variation_43492	chr6	23851202	23853998	2796	Wang et al. (2008)	Illumina DNA sequencing	y
100366	chr6	24325273	24360896	35623	11	chr6	24325242	24361600	36358	single								
100367						chr6	24451736	24563015	111279	single								
2212	chr6	24608922	24609959	1037	3	chr6	24607580	24611059	3479	single	Variation_47969	chr6	24589278	24714015	124737	Gusev et al. (2009)	SNP genotyping analysis	y
100368	chr6	25150041	25203500	53459	35	chr6	25150010	25204596	54586	HiConf	Variation_4488	chr6	25147353	25307091	159738	Wong et al. (2007)	BAC Array CGH	
2214	chr6	26093928	26100494	6566	3	chr6	26093897	26100610	6713	single								
2215	chr6	26171544	26174533	2989	1	chr6	26171513	26174600	3087	single								
2216	chr6	26451579	26459367	7788	18	chr6	26451548	26459535	7987	HiConf	Variation_44245	chr6	26451807	26459548	7741	Bentley et al. (2008)	Illumina DNA sequencing	
2217	chr6	26773362	26786788	13426	2	chr6	26773331	26787051	13720	single	Variation_6484	chr6	26785006	26794104	9098	Mills et al. (2006)	Sequence trace read mapping	
2218	chr6	26787199	26789679	2480	6	chr6	26787198	26789746	2548	single	Variation_6484	chr6	26785006	26794104	9098	Mills et al. (2006)	Sequence trace read mapping	
2219	chr6	26827899	26872293	44394	15	chr6	26827868	26872654	44786	HiConf	Variation_32773	chr6	26827989	26890093	62104	Perry et al. (2008)	Agilent Custom CGH Arrays	
2220	chr6	26872901	27074173	201272	12	chr6	26872899	27078258	205359	HiConf	Variation_7555	chr6	26811045	27054910	243865	de Smith et al. (2007)	Agilent 185k CGH Arrays/Agilent Custom CGH Arrays	y
2221	chr6	27781096	27787270	6174	11	chr6	27781065	27787386	6321	HiConf	Variation_1123	chr6	27783269	27784773	1504	Conrad et al. (2005)	Mendelian inconsistencies	
2222	chr6	28128261	28130123	1862	2	chr6	28128230	28130141	1911	single								
100370	chr6	28532144	28584217	52073	2	chr6	28532113	28592701	60588	single								
2223	chr6	28786331	28790104	3773	6	chr6	28786300	28790171	3871	single								
100371	chr6	29031014	29043310	12296	1	chr6	28882977	29137067	254090	single								
2224	chr6	29653092	29660638	7546	2	chr6	29653061	29660803	7742	HiConf	Variation_44248	chr6	29653013	29660735	7722	Bentley et al. (2008)	Illumina DNA sequencing	
2225	chr6	29927492	29927656	164	11	chr6	29927461	29927657	196	single	Variation_3599	chr6	29900413	30083123	182710	Redon et al. (2006)	BAC Array CGH	
100372	chr6	29947810	30013919	66109	25	chr6	29947772	30022374	74602	HiConf	Variation_9032	chr6	29944442	30024232	79790	Pinto et al. (2007)	Affymetrix 500K SNP Mapping Array	
2227	chr6	30024757	30046121	21364	7	chr6	30024726	30046531	21805	HiConf	Variation_32778	chr6	30025409	30045561	20152	Perry et al. (2008)	Agilent Custom CGH Arrays	
2228	chr6	31098981	31105795	6814	7	chr6	31098561	31105911	7350	HiConf								
2229	chr6	31131391	31131584	193	54	chr6	31131391	31131587	196	single	Variation_40849	chr6	31131240	31131636	396	Wheeler et al. (2008)	Sequencing	y
2230	chr6	31301599	31302049	450	43	chr6	31301568	31302058	490	single	Variation_7561	chr6	31249483	31358597	109114	de Smith et al. (2007)	Agilent 185k CGH Arrays/Agilent Custom CGH Arrays	y
100374	chr6	31379999	31429484	49485	20	chr6	31379968	31430046	50078	HiConf	Variation_32780	chr6	31383949	31418034	34085	Perry et al. (2008)	Agilent Custom CGH Arrays	y
100375	chr6	31460776	31565117	104341	60	chr6	31460745	31577879	117134	HiConf	Variation_8509	chr6	31461308	31565557	104249	Pinto et al. (2007)	Affymetrix 500K SNP Mapping Array	
2235	chr6	31892833	31902927	10094	3	chr6	31892802	31903141	10339	HiConf								
100376						chr6	32055433	32176243	120810	HiConf								
2236	chr6	32245633	32250974	5341	3	chr6	32245602	32251090	5488	HiConf	Variation_3602	chr6	31979491	32317091	337600	Redon et al. (2006)	BAC Array CGH	
2237	chr6	32460363	32463191	2828	1	chr6	32459928	32465024	5096	single								
2238	chr6	32550756	32557959	7203	10	chr6	32550725	32558075	7350	single	Variation_33420	chr6	32555421	32556081	660	Perry et al. (2008)	Agilent Custom CGH Arrays	y
2239	chr6	32561977	32763816	201839	47	chr6	32561946	32764414	202468	HiConf	Variation_31280	chr6	32557305	32763101	205796	Perry et al. (2008)	Agilent Custom CGH Arrays	
2240	chr6	32793287	32810579	17292	41	chr6	32793128	32810915	17787	HiConf	Variation_31282	chr6	32799289	32808807	9518	Perry et al. (2008)	Agilent Custom CGH Arrays	
2241	chr6	32886014	32887645	1631	53	chr6	32885983	32887649	1666	HiConf	Variation_23803	chr6	32886732	32887798	1066	Levy et al. (2007)	Sequencing	
2242	chr6	33159682	33163207	3525	19	chr6	33159648	33163274	3626	HiConf								
2243						chr6	33255100	33268722	13622	HiConf								
2244	chr6	33691966	33693044	1078	38	chr6	33691935	33693062	1127	HiConf	Variation_44253	chr6	33691176	33693821	2645	Bentley et al. (2008)	Illumina DNA sequencing	y
2245	chr6	34046383	34050254	3871	41	chr6	34046352	34050321	3969	HiConf	Variation_1127	chr6	34046274	34050206	3932	Conrad et al. (2005)	Mendelian inconsistencies	
100378	chr6	34163243	34207863	44620	1	chr6	34092143	34210208	118065	HiConf								
2247	chr6	34425129	34426574	1445	19	chr6	34425122	34426592	1470	HiConf	Variation_7572	chr6	34425516	34426525	1009	de Smith et al. (2007)	Agilent 185k CGH Arrays/Agilent Custom CGH Arrays	
2248	chr6	34808039	34808872	833	74	chr6	34808008	34808890	882	single	Variation_7573	chr6	34802784	34808099	5315	de Smith et al. (2007)	Agilent 185k CGH Arrays/Agilent Custom CGH Arrays	
2249	chr6	34820893	34824258	3365	5	chr6	34820846	34824325	3479	HiConf	Variation_3605	chr6	34490470	34822655	332185	Redon et al. (2006)	BAC Array CGH	
2250	chr6	35239925	35245021	5096	2	chr6	35239894	35245137	5243	single								
100379	chr6	35593301	35675903	82602	4	chr6	35592915	35677611	84696	HiConf	Variation_31283	chr6	35605248	35673825	68577	Perry et al. (2008)	Agilent Custom CGH Arrays	
2252	chr6	35734895	35737202	2307	46	chr6	35734892	35737587	2695	HiConf	Variation_23804	chr6	35734412	35737724	3312	Levy et al. (2007)	Sequencing	
2253	chr6	35855463	35855723	260	44	chr6	35855432	35855726	294	single	Variation_7575	chr6	35855681	35879293	23612	de Smith et al. (2007)	Agilent 185k CGH Arrays/Agilent Custom CGH Arrays	
2254	chr6	35858795	35876825	18030	13	chr6	35858764	35877139	18375	HiConf	Variation_7575	chr6	35855681	35879293	23612	de Smith et al. (2007)	Agilent 185k CGH Arrays/Agilent Custom CGH Arrays	

locus_id	chrom	start	end	length	Yoruba w/ event	putative chr	putative start	putative end	putative len	putative type	variation_id	DGV_chr	DGV_start	DGV_end	DGV_len	Reference	Method/platform	complex
2255	chr6	36737058	36737139	81	3	chr6	36736991	36737187	196	single								
2256	chr6	37823254	37824283	1029	3	chr6	37823223	37824301	1078	single								y
2257	chr6	37958592	37960797	2205	51	chr6	37958561	37960815	2254	HiConf								
2258	chr6	38042607	38065559	22952	2	chr6	38042547	38066018	23471	HiConf								
2259	chr6	38497494	38511754	14260	3	chr6	38497463	38512016	14553	HiConf	Variation_38639	chr6	38499784	38510599	10815	McCarroll et al. (2008)	Affymetrix Human SNP Array 6.0	
2260	chr6	39176928	39180799	3871	3	chr6	39176897	39180866	3969	HiConf	Variation_38640	chr6	39176890	39180010	3120	McCarroll et al. (2008)	Affymetrix Human SNP Array 6.0	
100381	chr6	39974705	40021149	46444	3	chr6	39974666	40022074	47408	single								
2261	chr6	40542264	40543783	1519	3	chr6	40542233	40543801	1568	single								
2262	chr6	40944064	40950483	6419	5	chr6	40944033	40950599	6566	HiConf								
2263	chr6	41685532	41694499	8967	1	chr6	41685501	41694664	9163	single								
2264	chr6	44248673	44262246	13573	5	chr6	44248642	44262509	13867	single								
2265	chr6	44618918	44623033	4115	6	chr6	44618886	44623100	4214	HiConf	Variation_37852	chr6	44621323	44622929	1606	McCarroll et al. (2008)	Affymetrix Human SNP Array 6.0	
100382						chr6	46706801	46865855	159054	single								
2267	chr6	47409859	47412260	2401	2	chr6	47409828	47412327	2499	single								
2268	chr6	48090861	48092380	1519	4	chr6	48090830	48092398	1568	HiConf	Variation_4494	chr6	47988109	48136638	148529	Wong et al. (2007)	BAC Array CGH	
2269	chr6	48204933	48208764	3831	3	chr6	48204902	48208822	3920	single								
2270	chr6	49039128	49046410	7282	4	chr6	49039127	49046575	7448	HiConf	Variation_22822	chr6	49038166	49047694	9528	Korbel et al. (2007)	Paired End Mapping	
2271	chr6	49099330	49103033	3703	3	chr6	49099299	49103121	3822	single								
2272	chr6	49123340	49123650	310	3	chr6	49123309	49123652	343	single								
2273	chr6	49389949	49402787	12838	9	chr6	49389918	49403050	13132	HiConf								
100383	chr6	50852403	50932747	80344	4	chr6	50852372	50934398	82026	single								
2274	chr6	51307637	51307922	285	42	chr6	51307631	51307925	294	HiConf	Variation_47129	chr6	51307439	51308080	641	Bentley et al. (2008)	Illumina DNA sequencing	
2275	chr6	51382779	51385866	3087	2	chr6	51382748	51385933	3185	HiConf	Variation_3613	chr6	51366288	51548810	182522	Redon et al. (2006)	BAC Array CGH	
2276	chr6	51774975	51777131	2156	17	chr6	51774944	51777149	2205	HiConf	Variation_11280	chr6	51776784	51777084	300	de Smith et al. (2007)	Agilent 185k CGH Arrays/Agilent Custom CGH Arrays	
2277	chr6	52087840	52094534	6694	7	chr6	52087809	52094963	7154	HiConf								
2278	chr6	52274285	52278940	4655	1	chr6	52274254	52279007	4753	single								
2279	chr6	52901240	52904474	3234	23	chr6	52901209	52904541	3332	HiConf	Variation_32802	chr6	52901202	52904081	2879	Perry et al. (2008)	Agilent Custom CGH Arrays	
2280	chr6	52905405	52912363	6958	5	chr6	52905374	52912479	7105	HiConf	Variation_32803	chr6	52905511	52911594	6083	Perry et al. (2008)	Agilent Custom CGH Arrays	
2281	chr6	53144280	53145897	1617	8	chr6	53144249	53145915	1666	single								
2282	chr6	54036815	54042597	5782	11	chr6	54036784	54042713	5929	HiConf	Variation_43642	chr6	54035666	54042896	7230	Wang et al. (2008)	Illumina DNA sequencing	
2283	chr6	54662055	54665053	2998	7	chr6	54662024	54665062	3038	HiConf	Variation_42895	chr6	54664340	54664913	573	Wang et al. (2008)	Illumina DNA sequencing	
2284	chr6	54769028	54773628	4600	11	chr6	54767962	54773744	5782	HiConf								
2285	chr6	54954536	54958848	4312	10	chr6	54954505	54958915	4410	HiConf	Variation_37944	chr6	54956414	54958101	1687	McCarroll et al. (2008)	Affymetrix Human SNP Array 6.0	
2286	chr6	55449632	55452621	2989	17	chr6	55449601	55452688	3087	HiConf	Variation_43068	chr6	55449667	55450355	688	Wang et al. (2008)	Illumina DNA sequencing	
2287	chr6	55521760	55525484	3724	8	chr6	55521729	55525551	3822	HiConf								
100384	chr6	55864638	55940343	75705	3	chr6	55864607	55941880	77273	HiConf	Variation_38643	chr6	55934114	55951619	17505	McCarroll et al. (2008)	Affymetrix Human SNP Array 6.0	
2290	chr6	56012103	56021119	9016	20	chr6	56012072	56021284	9212	HiConf								
2291	chr6	56749994	56751023	1029	2	chr6	56749963	56751041	1078	single								
100385	chr6	56778970	56826018	47048	5	chr6	56778898	56826967	48069	single								
100386	chr6	57586828	57607189	20361	2	chr6	57308661	57646173	337512	HiConf	Variation_40868	chr6	57588703	57588806	103	Wheeler et al. (2008)	Sequencing	
2292	chr6	57731856	57735531	3675	24	chr6	57731825	57735598	3773	HiConf	Variation_1422	chr6	57731819	57735109	3290	Conrad et al. (2005)	Mendelian inconsistencies	
100387	chr6	58194906	58372441	177535	12	chr6	58193552	58376028	182476	HiConf	Variation_32807	chr6	58352182	58377554	25372	Perry et al. (2008)	Agilent Custom CGH Arrays	
2294	chr6	58681917	58689169	7252	11	chr6	58681886	58689334	7448	HiConf	Variation_0287	chr6	58175273	58786187	610914	Sebat et al. (2004)	ROMA	
2295	chr6	58714313	58717589	3276	23	chr6	58709914	58717754	7840	HiConf	Variation_0287	chr6	58175273	58786187	610914	Sebat et al. (2004)	ROMA	
2296	chr6	62284642	62296353	11711	19	chr6	62284611	62296567	11956	single	Variation_0080	chr6	62236549	62301785	65236	Iafrate et al. (2004)	BAC Array CGH	
2297	chr6	63592991	63650959	57968	13	chr6	63592960	63652201	59241	HiConf	Variation_0289	chr6	63569838	63946210	376372	Sebat et al. (2004)	ROMA	
2298	chr6	63655368	63656838	1470	10	chr6	63655337	63656856	1519	HiConf	Variation_32808	chr6	63656644	63658061	1417	Perry et al. (2008)	Agilent Custom CGH Arrays	
2299	chr6	64163106	64164331	1225	27	chr6	64163075	64164349	1274	single								
2300	chr6	64264781	64267623	2842	2	chr6	64264750	64267690	2940	single								
2301	chr6	64552754	64554959	2205	2	chr6	64552723	64554977	2254	single								
2302	chr6	65055543	65075878	20335	36	chr6	65055512	65076288	20776	HiConf	Variation_41599	chr6	65072306	65072612	306	Wang et al. (2008)	Illumina DNA sequencing	
2303	chr6	65404276	65406089	1813	55	chr6	65404245	65406107	1862	HiConf	Variation_22573	chr6	65401929	65407393	5464	Korbel et al. (2007)	Paired End Mapping	
2304	chr6	65966956	65967303	347	21	chr6	65966912	65967304	392	single	Variation_6498	chr6	65958943	65968428	9485	Mills et al. (2006)	Sequence trace read mapping	
2305	chr6	66458658	66461206	2548	3	chr6	66458627	66461273	2646	HiConf	Variation_10786	chr6	66460447	66460558	111	Conrad et al. (2005)	Mendelian inconsistencies	
2306	chr6	66467380	66478405	11025	22	chr6	66467349	66478619	11270	HiConf	Variation_36545	chr6	66450698	66471698	21000	Kidd et al. (2008)	Paired End Mapping	
2307	chr6	67081840	67083947	2107	6	chr6	67081809	67083965	2156	HiConf	Variation_10154	chr6	67075448	67105019	29571	Wang et al. (2007)	Illumina HumanHap550 BeadChip	
2308	chr6	67098451	67099823	1372	31	chr6	67098420	67099841	1421	HiConf	Variation_38052	chr6	67097634	67099913	2279	McCarroll et al. (2008)	Affymetrix Human SNP Array 6.0	
2309	chr6	67263042	67263352	310	2	chr6	67263011	67263354	343	single								
2310	chr6	67267599	67271225	3626	3	chr6	67267568	67271292	3724	HiConf								
2311	chr6	67381867	67382700	833	15	chr6	67381836	67382718	882	HiConf								
2312	chr6	67487560	67489863	2303	7	chr6	67487529	67489881	2352	HiConf								
2313	chr6	67645977	67651566	5589	2	chr6	67645946	67651973	6027	HiConf	Variation_30235	chr6	67613966	67646473	32507	Jakobsson et al. (2008)	Illumina HumanHap550 BeadChip	
2314	chr6	68000198	68003383	3185	3	chr6	68000167	68003450	3283	single								
2315	chr6	68288563	68381026	92463	3	chr6	68288532	68382906	94374	HiConf	Variation_32810	chr6	68343271	68362554	19283	Perry et al. (2008)	Agilent Custom CGH Arrays	
2316	chr6	68383054	68395824	12770	1	chr6	68383053	68396087	13034	single	Variation_9034	chr6	68272068	68474413	202345	Pinto et al. (2007)	Affymetrix 500K SNP Mapping Array	
2317	chr6	68396235	68493414	97179	1	chr6	68396234	68495116	98882	single	Variation_33422	chr6	68407903	68408581	678	Perry et al. (2008)	Agilent Custom CGH Arrays	
2318	chr6	69528067	69528327	260	22	chr6	69528036	69528330	294	single	Variation_47152	chr6	69527914	69528438	524	Bentley et al. (2008)	Illumina DNA sequencing	
2319	chr6	70070791	70075054	4263	13	chr6	70070760	70075121	4361	HiConf								
100390	chr6	72342153	72378936	36783	14	chr6	72342106	72379689	37583	HiConf	Variation_4497	chr6	72300729	72467478	166749	Wong et al. (2007)	BAC Array CGH	
2321	chr6	72920386	72924698	4312	5	chr6	72920355	72924765	4410	HiConf	Variation_44266	chr6	72920515	72930273	9758	Bentley et al. (2008)	Illumina DNA sequencing	
2322	chr6	73308466	73309838	1372	11	chr6	73308435	73309856	1421	single								
2323	chr6	74621568	74673429	51861	20	chr6	74621537	74674457	52920	HiConf	Variation_2640	chr6	74624342	74681784	57442	Redon et al. (2006)	Affymetrix 500K EA SNP Mapping Array	
2324	chr6	74766167	74773342	7175	26	chr6	74766136	74780493	14357	HiConf	Variation_37810	chr6	74766429	74773741	7312	McCarroll et al. (2008)	Affymetrix Human SNP Array 6.0	y
2325	chr6	74875486	74876466	980	13	chr6	74875455	74876484	1029	HiConf								
2326	chr6	75361419	75365829	4410	2	chr6	75361388	75365896	4508	HiConf								
2327	chr6	75777772	75790341	12569	15	chr6	75777741	75790579	12838	HiConf	Variation_22836	chr6	75772358	75782735	10377	Korbel et al. (2007)	Paired End Mapping	
2328	chr6	75847058	75853673	6615	2	chr6	75847027	75853789	6762	single								
2329						chr6	75967763	75975456	7693	single								
2330	chr6	77072940	77085680	12740	2	chr6	77072909	77085943	13034	HiConf	Variation_32814	chr6	77073927	77084753	10826	Perry et al. (2008)	Agilent Custom CGH Arrays	
2331	chr6	77154574	77159486	4912	21	chr6	77154543	77159492	4949	HiConf	Variation_23811	chr6	77154216	77159365	5149	Levy et al. (2007)	Sequencing	
2332	chr6	77277515	77278103	588	3	chr6	77277484	77278121	637	single	Variation_4498	chr6	77168577	77347942	179365	Wong et al. (2007)	BAC Array CGH	
2333	chr6	77496006	77517174	21168	13	chr6	77495975	77517584	21609	HiConf	Variation_31293	chr6	77496500	77510011	13511	Perry et al. (2008)	Agilent Custom CGH Arrays	
2334	chr6	77922551	77934262	11711	14	chr6	77922520	77934476	11956	HiConf	Variation_22854	chr6	77920602	77933011	12409	Korbel et al. (2007)	Paired End Mapping	
2335	chr6	78065582	78068865	3283	14	chr6	78065551	78068932	3381	HiConf								
2336	chr6	78228507	78231986	3479	23	chr6	78228476	78232053	3577	single	Variation_3621	chr6	78156739	78339761	183022	Redon et al. (2006)	BAC Array CGH	
2337	chr6	78493156	78496926	3770	6	chr6	78493125	78507629	14504	HiConf	Variation_36558	chr6	78475161	78514359	39198	Kidd et al. (2008)	Paired End Mapping	

locus_id	chrom	start	end	length	Yoruba w/ event	putative chr	putative start	putative end	putative len	putative type	variation_id	DGV_chr	DGV_start	DGV_end	DGV_len	Reference	Method/platform	complex
2338	chr6	78934979	78961891	26912	29	chr6	78934909	78962447	27538	HiConf	Variation_37918	chr6	78938170	78955234	17064	McCarroll et al. (2008)	Affymetrix Human SNP Array 6.0	
100392	chr6	79014590	79094117	79527	13	chr6	79014559	79095727	81168	HiConf	Variation_29770	chr6	79016607	79088460	71853	Jakobsson et al. (2008)	Illumina HumanHap550 BeadChip	
2340	chr6	79574777	79585817	11040	22	chr6	79574702	79588569	13867	HiConf	Variation_38048	chr6	79577781	79585293	7512	McCarroll et al. (2008)	Affymetrix Human SNP Array 6.0	
2341	chr6	80434242	80435271	1029	7	chr6	80434211	80435289	1078	single								
2342	chr6	81564289	81566033	1744	13	chr6	81560770	81571697	10927	HiConf								y
2343	chr6	82174526	82175114	588	35	chr6	82174495	82175132	637	single	Variation_2646	chr6	82154779	82217840	63061	Redon et al. (2006)	Affymetrix 500K EA SNP Mapping Array	
2344	chr6	82185551	82192754	7203	3	chr6	82185520	82192870	7350	single	Variation_10160	chr6	82177366	82190504	13138	Wang et al. (2007)	Illumina HumanHap550 BeadChip	y
2345	chr6	82233081	82235237	2156	47	chr6	82233050	82240694	7644	HiConf	Variation_28377	chr6	82234968	82235095	127	Levy et al. (2007)	Sequencing	
2346	chr6	83392029	83392236	207	30	chr6	83391998	83392243	245	single								
2347	chr6	83792457	83793094	637	2	chr6	83792426	83793112	686	single								
2348	chr6	84010458	84017024	6566	1	chr6	84010427	84017140	6713	single								
2349	chr6	84791469	84795879	4410	3	chr6	84791438	84795946	4508	HiConf	Variation_36578	chr6	84762867	84798376	35509	Kidd et al. (2008)	Paired End Mapping	
2350	chr6	86618468	86623436	4968	4	chr6	86602429	86630065	27636	HiConf	Variation_10161	chr6	86602842	86622662	19820	Wang et al. (2007)	Illumina HumanHap550 BeadChip	
2351	chr6	86680468	86682918	2450	55	chr6	86680437	86682985	2548	HiConf								
2352	chr6	87114979	87116911	1932	8	chr6	87114626	87116929	2303	HiConf								
2353	chr6	87462998	87474268	11270	12	chr6	87462967	87474482	11515	HiConf	Variation_32824	chr6	87463069	87474530	11461	Perry et al. (2008)	Agilent Custom CGH Arrays	
100393	chr6	88173855	88177083	3228	50	chr6	88164329	88195125	30796	single	Variation_28381	chr6	88174914	88175047	133	Levy et al. (2007)	Sequencing	
100394	chr6	88451647	88522473	70826	1	chr6	88451616	88523866	72250	single	Variation_3627	chr6	88415707	88498192	82485	Redon et al. (2006)	BAC Array CGH	
2354	chr6	88857930	88859400	1470	61	chr6	88857899	88859418	1519	HiConf								
2355	chr6	89548046	89548732	686	15	chr6	89548015	89548750	735	single								
2356	chr6	89898450	89903835	5385	32	chr6	89898414	89903951	5537	HiConf								
2357	chr6	89978462	89978863	401	44	chr6	89978431	89978872	441	HiConf	Variation_40886	chr6	89978472	89978895	423	Wheeler et al. (2008)	Sequencing	
100395	chr6	93120540	93210135	89595	1	chr6	93112545	93212113	99568	single	Variation_9542	chr6	93122057	93207521	85464	Wang et al. (2007)	Illumina HumanHap550 BeadChip	
2359	chr6	93351965	93375975	24010	13	chr6	93351934	93376434	24500	HiConf								
100397	chr6	93469804	93495244	25440	1	chr6	93460445	93502879	42434	single								
2360	chr6	93631762	93635650	3888	33	chr6	93630597	93638731	8134	HiConf	Variation_32826	chr6	93631964	93635493	3529	Perry et al. (2008)	Agilent Custom CGH Arrays	
2361	chr6	93653198	93655520	2322	1	chr6	93652843	93655587	2744	single	Variation_30242	chr6	93587478	93672171	84693	Jakobsson et al. (2008)	Illumina HumanHap550 BeadChip	
100398	chr6	94035466	94080938	45472	1	chr6	94035435	94081863	46428	single								
100399	chr6	94503710	94645077	141367	1	chr6	94503679	94647862	144183	single								
2362	chr6	94792075	94792435	360	7	chr6	94792044	94792436	392	single								
2363	chr6	95020219	95025707	5488	2	chr6	95020188	95025823	5635	HiConf	Variation_10163	chr6	95020388	95023735	3347	Wang et al. (2007)	Illumina HumanHap550 BeadChip	
2364	chr6	96494403	96495903	1500	23	chr6	96494402	96495921	1519	HiConf	Variation_39466	chr6	96494322	96496142	1820	Wheeler et al. (2008)	Sequencing	
2365	chr6	96652213	96653732	1519	1	chr6	96652182	96653750	1568	single								
2366	chr6	97572580	97577774	5194	34	chr6	97572549	97577890	5341	HiConf	Variation_22653	chr6	97573523	97579899	6376	Korbel et al. (2007)	Paired End Mapping	
2367	chr6	97692483	97706987	14504	5	chr6	97692452	97707299	14847	HiConf								
2368	chr6	98703255	98704627	1372	2	chr6	98703224	98704645	1421	HiConf								
2369	chr6	99347096	99348536	1440	3	chr6	99346741	99348554	1813	single								
2370	chr6	99386070	99390578	4508	21	chr6	99386039	99390645	4606	single								
2371	chr6	100075990	100078538	2548	5	chr6	100075959	100078605	2646	single								
100400						chr6	100125866	100218647	92781	single								
2372	chr6	100372734	100377970	5236	9	chr6	100372703	100384610	11907	HiConf	Variation_8525	chr6	100364000	100781771	417771	Pinto et al. (2007)	Affymetrix 500K SNP Mapping Array	
2373	chr6	101301970	101303342	1372	7	chr6	101301939	101303360	1421	single								
2374	chr6	101486553	101491551	4998	12	chr6	101486522	101491667	5145	HiConf								
2375	chr6	101589257	101593569	4312	19	chr6	101589226	101593636	4410	HiConf								
2376	chr6	102041086	102045643	4557	35	chr6	102041055	102045710	4655	HiConf	Variation_7511	chr6	102041328	102045443	4115	de Smith et al. (2007)	Agilent 185k CGH Arrays/Agilent Custom CGH Arrays	
2377	chr6	102987178	102989824	2646	2	chr6	102987147	102989891	2744	single								
2378	chr6	103564055	103566569	2514	1	chr6	103564024	103566817	2793	single	Variation_7512	chr6	103381419	103609341	227922	de Smith et al. (2007)	Agilent 185k CGH Arrays/Agilent Custom CGH Arrays	
100401	chr6	103840440	103881524	41084	45	chr6	103840409	103882402	41993	HiConf	Variation_23220	chr6	103842682	103870484	27802	Korbel et al. (2007)	Paired End Mapping	
2380	chr6	104095215	104100654	5439	5	chr6	104095184	104100770	5586	HiConf	Variation_38622	chr6	104096781	104100508	3727	McCarroll et al. (2008)	Affymetrix Human SNP Array 6.0	
100402						chr6	104208007	104279718	71711	single								
2381	chr6	104320615	104324241	3626	4	chr6	104320584	104324308	3724	HiConf								
2382	chr6	105367552	105369561	2009	52	chr6	105367518	105369576	2058	HiConf	Variation_39467	chr6	105367521	105370452	2931	Wheeler et al. (2008)	Sequencing	
2383	chr6	106190112	106197266	7154	2	chr6	106190081	106197382	7301	single								
2384	chr6	106261407	106263906	2499	3	chr6	106261376	106263973	2597	HiConf								
2385	chr6	106511160	106512826	1666	15	chr6	106511129	106512844	1715	HiConf								y
2386	chr6	106560919	106562071	1152	3	chr6	106560472	106562089	1617	single								
2387	chr6	106621704	106632337	10633	4	chr6	106621673	106632551	10878	single								
2388	chr6	107136302	107153403	17101	17	chr6	107136271	107153764	17493	HiConf								
2389	chr6	107651733	107653497	1764	18	chr6	107651702	107653515	1813	HiConf								
2390	chr6	108138107	108139283	1176	31	chr6	108138076	108139301	1225	single	Variation_44219	chr6	108138029	108139092	1063	Bentley et al. (2008)	Illumina DNA sequencing	
2391	chr6	108361400	108364585	3185	3	chr6	108361369	108364652	3283	single								y
2392	chr6	108716258	108717385	1127	13	chr6	108716227	108717403	1176	single	Variation_4505	chr6	108594507	108770399	175892	Wong et al. (2007)	BAC Array CGH	
2393	chr6	109185237	109189598	4361	2	chr6	109185206	109189665	4459	single								
2394	chr6	109648483	109652795	4312	4	chr6	109648452	109652862	4410	single								y
2395	chr6	110280730	110280937	207	30	chr6	110280699	110280944	245	single	Variation_44220	chr6	110279402	110281115	1713	Bentley et al. (2008)	Illumina DNA sequencing	
2396	chr6	110799836	110823062	23226	12	chr6	110799756	110823521	23765	single								
2397	chr6	112294826	112295953	1127	3	chr6	112294795	112295971	1176	single								
100403	chr6	112917262	112956059	38797	1	chr6	112906879	112971583	64704	HiConf								
2400	chr6	112981904	112986216	4312	9	chr6	112981873	112986283	4410	HiConf	Variation_22525	chr6	112982011	112986370	4359	Korbel et al. (2007)	Paired End Mapping	
2401	chr6	114330825	114331658	833	87	chr6	114330794	114331676	882	HiConf								
100404	chr6	114331909	114395873	63964	4	chr6	114331750	114397165	65415	HiConf								
2402	chr6	114444979	114447932	2953	3	chr6	114444278	114450991	6713	single								
2404	chr6	115466841	115473162	6321	15	chr6	115466810	115473278	6468	HiConf	Variation_9545	chr6	114704350	115577822	873472	Wang et al. (2007)	Illumina HumanHap550 BeadChip	
2405	chr6	115808616	115810527	1911	1	chr6	115808585	115810545	1960	single								
2406	chr6	115810674	115824690	14016	16	chr6	115810643	115824951	14308	HiConf								
100406	chr6	116253211	116377531	124320	2	chr6	116236380	116404425	168045	HiConf								
2408	chr6	117200216	117202372	2156	5	chr6	117200185	117202390	2205	HiConf								
2409	chr6	117380879	117380999	120	8	chr6	117380848	117381191	343	single								
100407	chr6	117616352	117699232	82880	4	chr6	117597673	117701308	103635	HiConf	Variation_2653	chr6	117570741	117716222	145481	Redon et al. (2006)	Affymetrix 500K EA SNP Mapping Array	
2411	chr6	118135479	118138027	2548	15	chr6	118135448	118138094	2646	single								
2412	chr6	119146300	119146937	637	15	chr6	119146269	119146955	686	HiConf								
2413	chr6	120970619	120971158	539	12	chr6	120970588	120971176	588	HiConf								
2414	chr6	121220862	121221062	200	57	chr6	121220831	121221174	343	single	Variation_46983	chr6	121220809	121220941	132	Bentley et al. (2008)	Illumina DNA sequencing	
2415	chr6	121561265	121567684	6419	8	chr6	121561234	121567800	6566	HiConf	Variation_38624	chr6	121563601	121565754	2153	McCarroll et al. (2008)	Affymetrix Human SNP Array 6.0	
2416	chr6	121837674	121841184	3510	25	chr6	121837643	121841563	3920	HiConf	Variation_36451	chr6	121829220	121864753	35533	Kidd et al. (2008)	Paired End Mapping	
2417	chr6	121891721	121893534	1813	11	chr6	121891690	121893552	1862	HiConf								

locus_id	chrom	start	end	length	Yoruba w/ event	putative chr	putative start	putative end	putative len	putative type	variation_id	DGV_chr	DGV_start	DGV_end	DGV_len	Reference	Method/platform	complex
2418	chr6	122122168	122122854	686	24	chr6	122122137	122122872	735	HiConf								
2419	chr6	122132703	122134271	1568	6	chr6	122132672	122134289	1617	HiConf	Variation_44222	chr6	122132453	122134724	2271	Bentley et al. (2008)	Illumina DNA sequencing	
2420	chr6	122190278	122195962	5684	4	chr6	122190247	122196078	5831	single								
2421	chr6	122906707	122910127	3420	8	chr6	122906676	122910498	3822	single	Variation_3636	chr6	122765634	122925836	160202	Redon et al. (2006)	BAC Array CGH	
2422	chr6	123415670	123415778	108	33	chr6	123415639	123415786	147	HiConf								
2423	chr6	123603536	123603698	162	2	chr6	123603505	123603701	196	single								
2424	chr6	125859594	125864494	4900	8	chr6	125859563	125864610	5047	single								
100408	chr6	126094217	126137682	43465	1	chr6	126094151	126166671	72520	single								
2425	chr6	126224987	126228370	3383	43	chr6	126224956	126228435	3479	HiConf	Variation_38103	chr6	126225385	126228469	3084	McCarroll et al. (2008)	Affymetrix Human SNP Array 6.0	
2426	chr6	126276486	126281288	4802	2	chr6	126276455	126281404	4949	single								
2427	chr6	126281631	126286335	4704	3	chr6	126281600	126286402	4802	single								
2428	chr6	126509753	126509883	130	39	chr6	126509744	126509891	147	HiConf	Variation_40913	chr6	126509700	126509937	237	Wheeler et al. (2008)	Sequencing	
2429	chr6	127124970	127135701	10731	8	chr6	127124939	127135915	10976	single								
2430	chr6	127136191	127141042	4851	4	chr6	127136160	127141158	4998	single								
100409	chr6	127834791	127889223	54432	4	chr6	127825688	127890491	64803	single								
2431	chr6	128184548	128188564	4016	2	chr6	128184515	128188631	4116	HiConf								
2432	chr6	129342465	129345398	2933	3	chr6	129342434	129346550	4116	single	Variation_3638	chr6	129315361	129484648	169287	Redon et al. (2006)	BAC Array CGH	
2433	chr6	129745490	129760833	15343	7	chr6	129745459	129760992	15533	single								
2434	chr6	130795511	130801636	6125	3	chr6	130795480	130801752	6272	HiConf								
2435	chr6	131094411	131096616	2205	2	chr6	131094380	131096634	2254	single								
2436	chr6	132061052	132077743	16691	30	chr6	132061003	132078055	17052	HiConf	Variation_32838	chr6	132048222	132253866	205644	Perry et al. (2008)	Agilent Custom CGH Arrays	
2437	chr6	132148499	132150214	1715	4	chr6	132148468	132150232	1764	HiConf	Variation_44224	chr6	132148299	132150481	2182	Bentley et al. (2008)	Illumina DNA sequencing	
2438	chr6	132233465	132248753	15288	6	chr6	132233434	132249065	15631	HiConf	Variation_32838	chr6	132048222	132253866	205644	Perry et al. (2008)	Agilent Custom CGH Arrays	
100411	chr6	132919686	132998248	78562	41	chr6	132919655	132999868	80213	HiConf								
2440	chr6	133107968	133112819	4851	3	chr6	133107937	133112935	4998	HiConf								
2441	chr6	133557151	133562479	5328	2	chr6	133557120	133562706	5586	HiConf	Variation_32839	chr6	133559064	133562279	3215	Perry et al. (2008)	Agilent Custom CGH Arrays	
2442	chr6	133786597	133789117	2520	26	chr6	133786342	133789184	2842	HiConf								
100412						chr6	134184614	134266101	81487	HiConf								
2444	chr6	134311084	134311276	192	37	chr6	134311083	134311279	196	single	Variation_40917	chr6	134310673	134311421	748	Wheeler et al. (2008)	Sequencing	
2445	chr6	134631231	134636033	4802	9	chr6	134631200	134636100	4900	HiConf								
2446	chr6	135202375	135205462	3087	1	chr6	135202344	135205529	3185	single								
2447	chr6	135329579	135329642	63	27	chr6	135329548	135329646	98	single								
2448	chr6	135651019	135652489	1470	5	chr6	135650988	135652507	1519	HiConf								
2449	chr6	135824038	135825312	1274	10	chr6	135824007	135825330	1323	HiConf								
2450	chr6	136314283	136327268	12985	1	chr6	136314252	136327531	13279	single								
2451	chr6	136859359	136863475	4116	2	chr6	136859328	136863542	4214	single								
2452	chr6	136868622	136869355	733	2	chr6	136868295	136869373	1078	single								y
2453	chr6	136893071	136894590	1519	62	chr6	136893040	136894608	1568	HiConf	Variation_44230	chr6	136893367	136894613	1246	Bentley et al. (2008)	Illumina DNA sequencing	
2454	chr6	137355552	137355690	138	54	chr6	137355551	137355698	147	HiConf	Variation_12863	chr6	137355387	137355879	492	Mills et al. (2006)	Sequence trace read mapping	
100413	chr6	137429133	137459732	30599	2	chr6	137419178	137460534	41356	single	Variation_37750	chr6	137430261	137460049	29788	McCarroll et al. (2008)	Affymetrix Human SNP Array 6.0	
2455	chr6	138802650	138803483	833	11	chr6	138802619	138803501	882	single	Variation_8529	chr6	138757930	139068470	310540	Pinto et al. (2007)	Affymetrix 500K SNP Mapping Array	y
2456	chr6	138978952	138979834	882	26	chr6	138978921	138979852	931	single	Variation_8529	chr6	138757930	139068470	310540	Pinto et al. (2007)	Affymetrix 500K SNP Mapping Array	
2457	chr6	138980030	139001247	21217	8	chr6	138979999	139001657	21658	single	Variation_47005	chr6	138989439	138989759	320	Bentley et al. (2008)	Illumina DNA sequencing	
2458	chr6	139644301	139649811	5510	27	chr6	139644292	139649927	5635	HiConf	Variation_7526	chr6	139644130	139648760	4630	de Smith et al. (2007)	Agilent 185k CGH Arrays/Agilent Custom CGH Arrays	
100414	chr6	140044033	140094829	50796	2	chr6	140025659	140096219	70560	HiConf								
2459	chr6	141097124	141097331	207	31	chr6	141097093	141097338	245	HiConf	Variation_7527	chr6	140999039	141099048	100009	de Smith et al. (2007)	Agilent 185k CGH Arrays/Agilent Custom CGH Arrays	
2460	chr6	141419348	141421945	2597	26	chr6	141419317	141422012	2695	HiConf								
2461	chr6	142085062	142092356	7294	10	chr6	142085031	142092724	7693	HiConf	Variation_37449	chr6	142085057	142092341	7284	Cooper et al. (2008)	Illumina Human 1M BeadChip	
2462	chr6	142152437	142155279	2842	13	chr6	142152406	142155346	2940	single								
2463	chr6	143648162	143652768	4606	4	chr6	143648131	143652835	4704	HiConf								
2464	chr6	144239984	144241118	1134	10	chr6	144239953	144241325	1372	HiConf								
100415	chr6	144783492	144870320	86828	2	chr6	144783461	144872078	88617	single								
2466	chr6	145747195	145748969	1774	55	chr6	145747193	145749153	1960	HiConf	Variation_23782	chr6	145747164	145748218	1054	Levy et al. (2007)	Sequencing	
2467	chr6	146805134	146812680	7546	8	chr6	146805103	146812845	7742	single	Variation_0092	chr6	146665208	146865524	200316	Iafrate et al. (2004)	BAC Array CGH	
2468	chr6	146858985	146862807	3822	4	chr6	146858954	146862874	3920	single	Variation_0092	chr6	146665208	146865524	200316	Iafrate et al. (2004)	BAC Array CGH	
2469	chr6	147480011	147486038	6027	10	chr6	147479980	147486154	6174	HiConf	Variation_44232	chr6	147480085	147486123	6038	Bentley et al. (2008)	Illumina DNA sequencing	
2470	chr6	147976185	147977509	1324	12	chr6	147976154	147977526	1372	single	Variation_32841	chr6	147976145	147978550	2405	Perry et al. (2008)	Agilent Custom CGH Arrays	
2471	chr6	149239454	149242394	2940	44	chr6	149239423	149242461	3038	HiConf								
2472	chr6	149718674	149719422	748	5	chr6	149718643	149719525	882	single								
100416	chr6	150503162	150541119	37957	17	chr6	150503084	150541843	38759	HiConf								
2474	chr6	150685199	150689560	4361	20	chr6	150685168	150689627	4459	HiConf	Variation_36468	chr6	150682063	150694444	12381	Kidd et al. (2008)	Paired End Mapping	y
2475	chr6	150724840	150743792	18952	27	chr6	150724809	150743968	19159	HiConf	Variation_28121	chr6	150735256	150735382	126	Levy et al. (2007)	Sequencing	
2476	chr6	151397218	151397425	207	19	chr6	151397187	151397432	245	single	Variation_3644	chr6	151329901	151496532	166631	Redon et al. (2006)	BAC Array CGH	
2477	chr6	151548432	151553920	5488	14	chr6	151548401	151554036	5635	HiConf	Variation_6517	chr6	151548358	151554080	5722	Mills et al. (2006)	Sequence trace read mapping	
2478	chr6	152431461	152436361	4900	39	chr6	152431430	152436477	5047	HiConf	Variation_43439	chr6	152431648	152434006	2358	Wang et al. (2008)	Illumina DNA sequencing	
2479	chr6	153268577	153268887	310	3	chr6	153268546	153268889	343	single	Variation_3645	chr6	153104985	153276423	171438	Redon et al. (2006)	BAC Array CGH	
100417	chr6	153325605	153385170	59565	4	chr6	153299294	153386930	87636	single								
2480	chr6	154661941	154662970	1029	18	chr6	154661910	154662988	1078	single								
2481	chr6	154707315	154723534	16219	11	chr6	154707284	154723846	16562	HiConf								
2482	chr6	154749470	154766360	16890	2	chr6	154749081	154766721	17640	HiConf	Variation_32842	chr6	154750205	154766312	16107	Perry et al. (2008)	Agilent Custom CGH Arrays	
2483	chr6	155959804	155961323	1519	14	chr6	155959773	155961341	1568	HiConf								
2484	chr6	156095877	156096759	882	8	chr6	156095846	156096777	931	single								
2485	chr6	156202354	156203726	1372	2	chr6	156202323	156203744	1421	HiConf								y
2486	chr6	157502912	157512026	9114	22	chr6	157502881	157512191	9310	single	Variation_7533	chr6	157172446	157878320	705874	de Smith et al. (2007)	Agilent 185k CGH Arrays/Agilent Custom CGH Arrays	y
2487	chr6	158113109	158113469	360	43	chr6	158113078	158113470	392	single								
2488	chr6	158113795	158116539	2744	34	chr6	158113764	158116606	2842	single								
2489	chr6	158558568	158560577	2009	2	chr6	158558537	158560595	2058	single	Variation_2659	chr6	158548193	158569341	21148	Redon et al. (2006)	Affymetrix 500K EA SNP Mapping Array	
2490	chr6	159881764	159886027	4263	1	chr6	159881733	159886094	4361	single								
2491	chr6	160006567	160008576	2009	28	chr6	160006536	160008594	2058	HiConf	Variation_7540	chr6	160007135	160008150	1015	de Smith et al. (2007)	Agilent 185k CGH Arrays/Agilent Custom CGH Arrays	
100418	chr6	160944721	160988037	43316	14	chr6	160944690	160988913	44223	HiConf	Variation_31306	chr6	160947482	161001033	53551	Perry et al. (2008)	Agilent Custom CGH Arrays	
2493	chr6	161136654	161137242	588	24	chr6	161136623	161137260	637	single	Variation_5227	chr6	161131161	161362367	231206	Simon-Sanchez et al. (2007)	Illumina HumanHap300 BeadChip	
2494	chr6	161181685	161198737	17052	31	chr6	161181654	161199098	17444	HiConf	Variation_43028	chr6	161195263	161195905	642	Wang et al. (2008)	Illumina DNA sequencing	
2495	chr6	161486416	161486906	490	18	chr6	161486385	161486924	539	single								
2496	chr6	161646989	161647773	784	21	chr6	161646958	161647791	833	HiConf	Variation_8531	chr6	161512090	161775775	263685	Pinto et al. (2007)	Affymetrix 500K SNP Mapping Array	
2497	chr6	161732004	161734601	2597	3	chr6	161731973	161734668	2695	HiConf	Variation_8531	chr6	161512090	161775775	263685	Pinto et al. (2007)	Affymetrix 500K SNP Mapping Array	
2498						chr6	161893379	161893428	49	single								

locus_id	chrom	start	end	length	Yoruba w/ event	putative chr	putative start	putative end	putative len	putative type	variation_id	DGV_chr	DGV_start	DGV_end	DGV_len	Reference	Method/platform	complex
2499	chr6	162305206	162307019	1813	23	chr6	162305175	162307037	1862	single	Variation_3649	chr6	162110746	162331240	220494	Redon et al. (2006)	BAC Array CGH	
2500	chr6	162350325	162351119	794	3	chr6	162349961	162351137	1176	single	Variation_8532	chr6	162241367	162629938	388571	Pinto et al. (2007)	Affymetrix 500K SNP Mapping Array	
2501	chr6	162413643	162418151	4508	10	chr6	162413612	162418218	4606	HiConf	Variation_38631	chr6	162416281	162423724	7443	McCarroll et al. (2008)	Affymetrix Human SNP Array 6.0	
2502	chr6	162418321	162426775	8454	15	chr6	162418316	162426940	8624	HiConf	Variation_10169	chr6	162419167	162426558	7391	Wang et al. (2007)	Illumina HumanHap550 BeadChip	
2503	chr6	162498560	162500128	1568	6	chr6	162498529	162500146	1617	single	Variation_8534	chr6	162498782	162629938	131156	Pinto et al. (2007)	Affymetrix 500K SNP Mapping Array	
2504	chr6	162502823	162506645	3822	30	chr6	162502792	162506712	3920	HiConf	Variation_8534	chr6	162498782	162629938	131156	Pinto et al. (2007)	Affymetrix 500K SNP Mapping Array	y
2505	chr6	162643406	162643894	488	9	chr6	162643373	162643912	539	single	Variation_9042	chr6	162629579	162747454	117875	Pinto et al. (2007)	Affymetrix 500K SNP Mapping Array	
2506	chr6	162645168	162645478	310	15	chr6	162645137	162645480	343	single	Variation_9042	chr6	162629579	162747454	117875	Pinto et al. (2007)	Affymetrix 500K SNP Mapping Array	y
100419	chr6	162896493	162935452	38959	10	chr6	162896434	162943866	47432	single	Variation_28150	chr6	162933448	162933813	365	Levy et al. (2007)	Sequencing	y
2508	chr6	164462774	164468262	5488	47	chr6	164462743	164468378	5635	HiConf	Variation_23121	chr6	164462814	164469843	7029	Korbel et al. (2007)	Paired End Mapping	
2509	chr6	164966053	164972570	6517	2	chr6	164966022	164972686	6664	single	Variation_4517	chr6	164934466	165151194	216728	Wong et al. (2007)	BAC Array CGH	
2510	chr6	165643135	165652695	9560	55	chr6	165643104	165652708	9604	HiConf	Variation_22786	chr6	165644517	165652697	8180	Korbel et al. (2007)	Paired End Mapping	
2511	chr6	165796652	165813361	16709	3	chr6	165796621	165813673	17052	single	Variation_9553	chr6	165797421	165813337	15916	Wang et al. (2007)	Illumina HumanHap550 BeadChip	
100420	chr6	166092377	166132029	39652	1	chr6	166010506	166134525	124019	single	Variation_3652	chr6	166061631	166218925	157294	Redon et al. (2006)	BAC Array CGH	
2512	chr6	166235496	166246962	11466	11	chr6	166235465	166247176	11711	single								
2513	chr6	166386514	166389209	2695	2	chr6	166386483	166389276	2793	HiConf								y
2514	chr6	166417776	166430222	12446	47	chr6	166417745	166430485	12740	HiConf	Variation_43579	chr6	166417566	166422646	5080	Wang et al. (2008)	Illumina DNA sequencing	
2515	chr6	166662139	166664050	1911	10	chr6	166662108	166664068	1960	single								y
2516	chr6	167408066	167409830	1764	60	chr6	167408035	167409848	1813	HiConf	Variation_44239	chr6	167408049	167409568	1519	Bentley et al. (2008)	Illumina DNA sequencing	
2517	chr6	167463191	167464220	1029	14	chr6	167463160	167464238	1078	HiConf	Variation_33826	chr6	167463429	167463958	529	Perry et al. (2008)	Agilent Custom CGH Arrays	
2518	chr6	167671637	167671844	207	16	chr6	167671606	167671851	245	single	Variation_8539	chr6	167541579	167732488	190909	Pinto et al. (2007)	Affymetrix 500K SNP Mapping Array	
2519	chr6	167864942	167877241	12299	7	chr6	167864911	167877504	12593	single	Variation_2666	chr6	167520583	167919582	398999	Redon et al. (2006)	Affymetrix 500K EA SNP Mapping Array	
2520	chr6	168127925	168143017	15092	9	chr6	168127894	168143329	15435	HiConf	Variation_31312	chr6	168076956	168268834	191878	Perry et al. (2008)	Agilent Custom CGH Arrays	y
2521	chr6	168383950	168393261	9311	54	chr6	168383919	168393327	9408	single	Variation_28181	chr6	168389653	168389800	147	Levy et al. (2007)	Sequencing	
2522	chr6	168393532	168397180	3648	9	chr6	168393474	168397247	3773	single	Variation_36489	chr6	168360268	168395771	35503	Kidd et al. (2008)	Paired End Mapping	
2523	chr6	168439565	168440015	450	28	chr6	168439534	168440024	490	single	Variation_3654	chr6	167979717	168458233	478516	Redon et al. (2006)	BAC Array CGH	
2524	chr6	168738906	168740670	1764	11	chr6	168738875	168740688	1813	single	Variation_3655	chr6	168626236	168825483	199247	Redon et al. (2006)	BAC Array CGH	y
2525	chr6	168978516	169000981	22465	50	chr6	168978485	169001025	22540	HiConf	Variation_44242	chr6	168988763	168989764	1001	Bentley et al. (2008)	Illumina DNA sequencing	
2526	chr6	169375416	169378454	3038	18	chr6	169375385	169378521	3136	HiConf								
2527	chr6	169787163	169787563	400	39	chr6	169787132	169787573	441	single	Variation_47065	chr6	169787251	169788175	924	Bentley et al. (2008)	Illumina DNA sequencing	
100422	chr6	169825971	169902705	76734	2	chr6	169825940	169904267	78327	HiConf								
2528	chr6	169945629	169950725	5096	9	chr6	169945598	169950841	5243	HiConf	Variation_42458	chr6	169948141	169948628	487	Wang et al. (2008)	Illumina DNA sequencing	
2529	chr6	170226056	170226896	840	3	chr6	170226025	170227446	1421	single	Variation_4518	chr6	170226739	170318656	91917	Wong et al. (2007)	BAC Array CGH	
2530	chr6	170235709	170236836	1127	39	chr6	170235678	170236854	1176	HiConf	Variation_47068	chr6	170235693	170236123	430	Bentley et al. (2008)	Illumina DNA sequencing	
2531	chr6	170248841	170249216	375	3	chr6	170248810	170250084	1274	single	Variation_36492	chr6	170243194	170273085	29891	Kidd et al. (2008)	Paired End Mapping	
2532	chr6	170296028	170296518	490	11	chr6	170295997	170296536	539	single	Variation_12894	chr6	170296411	170296653	242	Mills et al. (2006)	Sequence trace read mapping	
2533	chr6	170299752	170302447	2695	13	chr6	170299721	170302514	2793	HiConf	Variation_36493	chr6	170291069	170325133	34064	Kidd et al. (2008)	Paired End Mapping	
2534	chr6	170325692	170329646	3954	8	chr6	170325691	170329660	3969	HiConf	Variation_6528	chr6	170326078	170328506	2428	Mills et al. (2006)	Sequence trace read mapping	
2535	chr6	170370802	170372125	1323	33	chr6	170370771	170372143	1372	single	Variation_36494	chr6	170368138	170389800	21662	Kidd et al. (2008)	Paired End Mapping	
2536	chr6	170494380	170495409	1029	8	chr6	170494349	170495427	1078	single	Variation_33828	chr6	170494889	170495544	655	Perry et al. (2008)	Agilent Custom CGH Arrays	
2537	chr6	170541420	170561118	19698	28	chr6	170541389	170561528	20139	HiConf	Variation_33433	chr6	170550290	170550872	582	Perry et al. (2008)	Agilent Custom CGH Arrays	y
2538	chr6	170611442	170626590	15148	41	chr6	170611410	170626698	15288	single	Variation_31319	chr6	170614231	170619361	5130	Perry et al. (2008)	Agilent Custom CGH Arrays	
2539	chr6	170641919	170652074	10155	38	chr6	170641888	170652178	10290	single	Variation_32855	chr6	170645511	170648343	2832	Perry et al. (2008)	Agilent Custom CGH Arrays	
100424	chr6	170753027	170885008	131981	12	chr6	170752996	170887721	134725	HiConf	Variation_32856	chr6	170779156	170885839	106683	Perry et al. (2008)	Agilent Custom CGH Arrays	
2541	chr7	48321	133091	84770	8	chr7	48290	134824	86534	HiConf	Variation_32857	chr7	113279	116290	3011	Perry et al. (2008)	Agilent Custom CGH Arrays	
2542	chr7	136717	147938	11221	6	chr7	136686	148152	11466	single	Variation_8543	chr7	141322	160848	19526	Pinto et al. (2007)	Affymetrix 500K SNP Mapping Array	
2543	chr7	149457	151907	2450	3	chr7	149426	151974	2548	single	Variation_8543	chr7	141322	160848	19526	Pinto et al. (2007)	Affymetrix 500K SNP Mapping Array	
2544	chr7	164157	174398	10241	14	chr7	164126	174612	10486	HiConf	Variation_8542	chr7	141322	166807	25485	Pinto et al. (2007)	Affymetrix 500K SNP Mapping Array	
2545	chr7	649012	650433	1421	16	chr7	648981	650451	1470	single								
2546	chr7	706538	716136	9598	11	chr7	706507	716209	9702	HiConf								
2547	chr7	1017492	1017699	207	29	chr7	1017461	1017706	245	single								
2548	chr7	1121764	1123626	1862	48	chr7	1121733	1123644	1911	HiConf	Variation_31322	chr7	1121911	1123667	1756	Perry et al. (2008)	Agilent Custom CGH Arrays	y
2549	chr7	1271704	1275428	3724	25	chr7	1271673	1275495	3822	HiConf	Variation_36603	chr7	1266569	1286569	20000	Kidd et al. (2008)	Paired End Mapping	
2550	chr7	1362599	1363775	1176	34	chr7	1362568	1363793	1225	single	Variation_4520	chr7	1322210	1518105	195895	Wong et al. (2007)	BAC Array CGH	
2551	chr7	1836233	1846327	10094	12	chr7	1836202	1846541	10339	single	Variation_36655	chr7	1817359	1859402	42043	Kidd et al. (2008)	Paired End Mapping	
2552	chr7	1908949	1911875	2926	30	chr7	1908918	1911907	2989	HiConf	Variation_47316	chr7	1909158	1910050	892	Bentley et al. (2008)	Illumina DNA sequencing	
100426	chr7	2059251	2143138	83887	1	chr7	2059128	2144878	85750	single								
2553	chr7	2383710	2384307	597	15	chr7	2383679	2384316	637	HiConf	Variation_12900	chr7	2383627	2384199	572	Mills et al. (2006)	Sequence trace read mapping	
2554	chr7	3102344	3104500	2156	21	chr7	3102313	3104518	2205	HiConf	Variation_44305	chr7	3101766	3104663	2897	Bentley et al. (2008)	Illumina DNA sequencing	
100427	chr7	3355520	3418657	63137	3	chr7	3349371	3426326	76955	HiConf	Variation_32862	chr7	3359748	3417864	58116	Perry et al. (2008)	Agilent Custom CGH Arrays	
2556	chr7	3892126	3893111	985	5	chr7	3892095	3893124	1029	single								
2557	chr7	4047769	4048681	912	47	chr7	4047719	4048699	980	HiConf	Variation_44310	chr7	4047787	4048820	1033	Bentley et al. (2008)	Illumina DNA sequencing	
2558	chr7	4079698	4094692	14994	5	chr7	4079667	4095004	15337	HiConf								
2559	chr7	4125709	4126444	735	14	chr7	4125678	4126462	784	single								
2560	chr7	4152365	4152625	260	34	chr7	4152334	4152628	294	HiConf								
2561	chr7	4383155	4386732	3577	25	chr7	4383124	4386799	3675	HiConf	Variation_37869	chr7	4383474	4385353	1879	McCarroll et al. (2008)	Affymetrix Human SNP Array 6.0	
2562	chr7	5324984	5326405	1421	27	chr7	5324953	5326423	1470	single	Variation_31328	chr7	5254913	5327343	72430	Perry et al. (2008)	Agilent Custom CGH Arrays	
2563	chr7	6008681	6010298	1617	17	chr7	6008650	6010316	1666	single								
2564	chr7	6088061	6111924	23863	9	chr7	6088030	6112383	24353	HiConf	Variation_36692	chr7	6071988	6113674	41686	Kidd et al. (2008)	Paired End Mapping	
100429	chr7	6782722	6819198	36476	1	chr7	6715892	7019667	303775	HiConf	Variation_7643	chr7	6751282	6806862	55580	de Smith et al. (2007)	Agilent 185k CGH Arrays/Agilent Custom CGH Arrays	
100430	chr7	7295328	7395714	100386	2	chr7	7287575	7416322	128747	HiConf	Variation_9044	chr7	7310505	7393969	83464	Pinto et al. (2007)	Affymetrix 500K SNP Mapping Array	
2566	chr7	7890477	7892633	2156	2	chr7	7890446	7892651	2205	HiConf	Variation_8547	chr7	7695772	8144214	448442	Pinto et al. (2007)	Affymetrix 500K SNP Mapping Array	
2567	chr7	8085546	8086771	1225	17	chr7	8085515	8086789	1274	HiConf	Variation_8547	chr7	7695772	8144214	448442	Pinto et al. (2007)	Affymetrix 500K SNP Mapping Array	
2568	chr7	8147090	8158879	11789	11	chr7	8147059	8159064	12005	single								
100431						chr7	8420749	8482195	61446	single								
2569	chr7	8513463	8513571	108	19	chr7	8513432	8513579	147	single								
2570	chr7	8987685	8987995	310	4	chr7	8987654	8987997	343	single								
2571	chr7	9048543	9056922	8379	4	chr7	9048512	9057087	8575	single	Variation_36718	chr7	9041789	9062944	21155	Kidd et al. (2008)	Paired End Mapping	
2572	chr7	10177748	10178287	539	1	chr7	10177717	10178305	588	single								
2573	chr7	10461458	10485223	23765	7	chr7	10461427	10485682	24255	HiConf	Variation_44279	chr7	10470842	10472676	1834	Bentley et al. (2008)	Illumina DNA sequencing	
100432	chr7	10977332	11005530	28198	2	chr7	10945425	11006773	61348	single	Variation_8549	chr7	10783585	10996273	212688	Pinto et al. (2007)	Affymetrix 500K SNP Mapping Array	
2575	chr7	11298329	11305385	7056	3	chr7	11298298	11305501	7203	single	Variation_2678	chr7	11274532	11318799	44267	Redon et al. (2006)	Affymetrix 500K EA SNP Mapping Array	
2576	chr7	11969829	11980115	10286	9	chr7	11969794	11980329	10535	HiConf	Variation_44285	chr7	11969835	11977099	7264	Bentley et al. (2008)	Illumina DNA sequencing	
100433	chr7	12478053	12531423	53370	23	chr7	12478022	12532216	54194	HiConf	Variation_31334	chr7	12482087	12530666	48579	Perry et al. (2008)	Agilent Custom CGH Arrays	
2578	chr7	12693933	12725356	31423	2	chr7	12693867	12726011	32144	HiConf	Variation_5397	chr7	12705816	12850075	144259	Simon-Sanchez et al. (2007)	Illumina HumanHap300 BeadChip	
2579	chr7	13071198	13090549	19351	5	chr7	13071167	13097333	26166	HiConf	Variation_32867	chr7	13071734	13089372	17638	Perry et al. (2008)	Agilent Custom CGH Arrays	

locus_id	chrom	start	end	length	Yoruba w/ event	putative chr	putative start	putative end	putative len	putative type	variation_id	DGV_chr	DGV_start	DGV_end	DGV_len	Reference	Method/platform	complex
2580	chr7	13244658	13247157	2499	84	chr7	13244627	13247224	2597	HiConf								
2581	chr7	13989605	13994456	4851	9	chr7	13989574	13994572	4998	single	Variation_0292	chr7	13405467	14521413	1115946	Sebat et al. (2004)	ROMA	
2582	chr7	15055992	15058589	2597	14	chr7	15055961	15058656	2695	HiConf	Variation_37898	chr7	15055803	15058658	2855	McCarroll et al. (2008)	Affymetrix Human SNP Array 6.0	
2583	chr7	15380323	15386742	6419	10	chr7	15380292	15386858	6566	HiConf								y
2584	chr7	16138500	16138990	490	19	chr7	16138469	16139008	539	HiConf	Variation_6533	chr7	16138299	16140597	2298	Mills et al. (2006)	Sequence trace read mapping	
100435	chr7	16591222	16605143	13921	1	chr7	16591180	16661422	70242	single								
2585	chr7	17414950	17418429	3479	3	chr7	17414919	17418496	3577	single	Variation_5236	chr7	17207480	17458368	250888	Simon-Sanchez et al. (2007)	Illumina HumanHap300 BeadChip	
2586	chr7	18786145	18796162	10017	23	chr7	18786086	18796376	10290	HiConf	Variation_0094	chr7	18697292	18872981	175689	Iafrate et al. (2004)	BAC Array CGH	
2587	chr7	18796799	18802630	5831	10	chr7	18796768	18802746	5978	HiConf	Variation_0094	chr7	18697292	18872981	175689	Iafrate et al. (2004)	BAC Array CGH	
2588	chr7	19085997	19086060	63	1	chr7	19085966	19086064	98	single	Variation_0099	chr7	19073773	19109124	35351	Iafrate et al. (2004)	BAC Array CGH	
100437						chr7	20310795	20402988	92193	single								
2589	chr7	20715149	20719951	4802	16	chr7	20715118	20720067	4949	HiConf	Variation_44300	chr7	20715310	20720100	4790	Bentley et al. (2008)	Illumina DNA sequencing	
2590	chr7	22401410	22403248	1838	47	chr7	22401404	22403266	1862	HiConf	Variation_39491	chr7	22401325	22403285	1960	Wheeler et al. (2008)	Sequencing	
2591	chr7	22630069	22636194	6125	3	chr7	22630038	22636310	6272	HiConf								
2592	chr7	23089591	23089951	360	12	chr7	23089560	23089952	392	single								
2593	chr7	23106447	23110706	4259	23	chr7	23106416	23110777	4361	HiConf	Variation_10181	chr7	23106465	23110722	4257	Wang et al. (2007)	Illumina HumanHap550 BeadChip	
2594	chr7	23184210	23187199	2989	6	chr7	23184179	23187266	3087	single								
100438						chr7	23637037	23744445	107408	single								
2595	chr7	24004791	24006430	1639	55	chr7	24004782	24006448	1666	HiConf	Variation_39492	chr7	24004690	24006599	1909	Wheeler et al. (2008)	Sequencing	
2596	chr7	24119130	24120453	1323	8	chr7	24119099	24120471	1372	single	Variation_4528	chr7	23981243	24156646	175403	Wong et al. (2007)	BAC Array CGH	
2597	chr7	24266583	24278891	12308	4	chr7	24266540	24284670	18130	HiConf	Variation_32878	chr7	24267982	24278919	10937	Perry et al. (2008)	Agilent Custom CGH Arrays	
2598	chr7	24326155	24329830	3675	6	chr7	24326124	24329897	3773	HiConf	Variation_9053	chr7	24302484	24400835	98351	Pinto et al. (2007)	Affymetrix 500K SNP Mapping Array	
2599	chr7	25305910	25308360	2450	1	chr7	25305879	25308427	2548	single								
2600	chr7	26103875	26111813	7938	32	chr7	26103844	26111978	8134	HiConf	Variation_36665	chr7	26101766	26113117	11351	Kidd et al. (2008)	Paired End Mapping	
2601	chr7	26186244	26187714	1470	10	chr7	26186213	26187732	1519	single								
2602	chr7	28990808	28999912	9104	1	chr7	28990777	29000332	9555	single								
100440	chr7	29655346	29704252	48906	8	chr7	29655315	29749371	94056	HiConf	Variation_7625	chr7	29641913	29681311	39398	de Smith et al. (2007)	Agilent 185k CGH Arrays/Agilent Custom CGH Arrays	
2603	chr7	30107975	30113901	5926	3	chr7	30107928	30114298	6370	HiConf								
100441	chr7	30581961	30656490	74529	2	chr7	30581930	30658002	76072	single								
2604	chr7	30835364	30839186	3822	3	chr7	30835333	30839253	3920	single								
2605	chr7	31282391	31285793	3402	41	chr7	31282360	31290494	8134	HiConf	Variation_44306	chr7	31282067	31285600	3533	Bentley et al. (2008)	Illumina DNA sequencing	
2606	chr7	31556889	31558506	1617	6	chr7	31556858	31558524	1666	HiConf								
2607	chr7	31679487	31682427	2940	4	chr7	31679456	31682494	3038	HiConf	Variation_8553	chr7	31590271	31706156	115885	Pinto et al. (2007)	Affymetrix 500K SNP Mapping Array	
2608	chr7	32253522	32253585	63	22	chr7	32253491	32253589	98	single	Variation_47342	chr7	32253376	32253647	271	Bentley et al. (2008)	Illumina DNA sequencing	
2609	chr7	32969118	32971218	2100	4	chr7	32969087	32971635	2548	single								
2610	chr7	34862753	34880109	17356	13	chr7	34862643	34880430	17787	HiConf	Variation_38676	chr7	34866102	34879011	12909	McCarroll et al. (2008)	Affymetrix Human SNP Array 6.0	
100443	chr7	35015040	35058972	43932	12	chr7	35015009	35198097	183088	HiConf								
100444	chr7	35885966	35984481	98515	3	chr7	35885935	35986507	100572	single	Variation_3674	chr7	35790751	36086963	296212	Redon et al. (2006)	BAC Array CGH	
2613	chr7	36318709	36319444	735	34	chr7	36318678	36319462	784	single								
100445	chr7	37684078	37743944	59866	1	chr7	37671103	37752026	80923	single	Variation_2690	chr7	37678050	37735131	57081	Redon et al. (2006)	Affymetrix 500K EA SNP Mapping Array	
2615	chr7	37753233	37755781	2548	2	chr7	37753202	37755848	2646	single	Variation_33837	chr7	37754168	37754989	821	Perry et al. (2008)	Agilent Custom CGH Arrays	y
2616	chr7	37849616	37856329	6713	30	chr7	37849585	37856445	6860	single	Variation_32880	chr7	37833985	38117000	283015	Perry et al. (2008)	Agilent Custom CGH Arrays	
2617	chr7	37897979	37903027	5048	4	chr7	37897948	37903142	5194	HiConf	Variation_32880	chr7	37833985	38117000	283015	Perry et al. (2008)	Agilent Custom CGH Arrays	
100446	chr7	38337852	38373818	35966	6	chr7	38337821	38374522	36701	HiConf	Variation_36670	chr7	38347509	38373707	26198	Kidd et al. (2008)	Paired End Mapping	
2620	chr7	39513901	39518115	4214	4	chr7	39513870	39518182	4312	single								
100447						chr7	39546504	39640486	93982	single								
2621	chr7	39771264	39784341	13077	2	chr7	39771218	39785281	14063	single								
2622	chr7	39824338	39864278	39940	5	chr7	39824285	39865004	40719	HiConf								
2623	chr7	41387024	41389670	2646	2	chr7	41386993	41389737	2744	HiConf								
2624	chr7	42510349	42514955	4606	1	chr7	42510318	42515022	4704	single								
2625	chr7	42524167	42532007	7840	1	chr7	42524136	42532172	8036	single								
2626	chr7	43347428	43349378	1950	13	chr7	43346797	43350570	3773	HiConf								
100448	chr7	43953032	44049662	96630	14	chr7	43953001	44051711	98710	HiConf	Variation_7628	chr7	43983706	44020882	37176	de Smith et al. (2007)	Agilent 185k CGH Arrays/Agilent Custom CGH Arrays	
2628	chr7	44384648	44386265	1617	10	chr7	44384617	44386283	1666	HiConf								
100449	chr7	45766028	45851681	85653	1	chr7	45728295	45862947	134652	HiConf								y
2630	chr7	46080440	46081616	1176	24	chr7	46080409	46081634	1225	HiConf								
2631	chr7	46796526	46807159	10633	12	chr7	46796495	46807373	10878	HiConf								
2632	chr7	46843223	46852141	8918	3	chr7	46843192	46852306	9114	HiConf	Variation_9055	chr7	46846075	46851185	5110	Pinto et al. (2007)	Affymetrix 500K SNP Mapping Array	
2633	chr7	47250217	47255558	5341	6	chr7	47250186	47255674	5488	HiConf	Variation_38678	chr7	47251811	47255449	3638	McCarroll et al. (2008)	Affymetrix Human SNP Array 6.0	
2634	chr7	48362370	48373983	11613	5	chr7	48362339	48374197	11858	HiConf	Variation_44311	chr7	48362276	48372418	10142	Bentley et al. (2008)	Illumina DNA sequencing	
2635	chr7	49143969	49150437	6468	3	chr7	49143938	49150553	6615	HiConf	Variation_38681	chr7	49144690	49148378	3688	McCarroll et al. (2008)	Affymetrix Human SNP Array 6.0	
2636	chr7	49557480	49559734	2254	2	chr7	49557449	49559752	2303	HiConf								
2637	chr7	50014846	50019424	4578	2	chr7	50014815	50019470	4655	HiConf								y
2638	chr7	50075459	50075909	450	19	chr7	50075428	50075918	490	single	Variation_44312	chr7	50075319	50076660	1341	Bentley et al. (2008)	Illumina DNA sequencing	
2639	chr7	50960889	50967700	6811	2	chr7	50960858	50967816	6958	HiConf	Variation_38682	chr7	50962568	50967266	4698	McCarroll et al. (2008)	Affymetrix Human SNP Array 6.0	
2640	chr7	51560845	51565633	4788	50	chr7	51560814	51569389	8575	HiConf	Variation_37452	chr7	51561819	51565696	3877	Cooper et al. (2008)	Illumina Human 1M BeadChip	
2641	chr7	51713242	51716906	3664	3	chr7	51703943	51717173	13230	HiConf	Variation_36679	chr7	51715999	51745948	29949	Kidd et al. (2008)	Paired End Mapping	
2642	chr7	51818389	51821182	2793	7	chr7	51818358	51821249	2891	HiConf								
100450	chr7	52069656	52110492	40836	4	chr7	52060957	52127205	66248	single								
2644	chr7	52338867	52340141	1274	11	chr7	52338836	52340159	1323	single	Variation_32885	chr7	52149672	52873819	724147	Perry et al. (2008)	Agilent Custom CGH Arrays	
2645	chr7	52931473	52932208	735	10	chr7	52931442	52932226	784	single								
2646	chr7	53181030	53185293	4263	8	chr7	53180999	53185360	4361	HiConf	Variation_38683	chr7	53181505	53186117	4612	McCarroll et al. (2008)	Affymetrix Human SNP Array 6.0	
2647	chr7	53187662	53205040	17378	6	chr7	53187565	53205401	17836	single								
2648	chr7	53290496	53295592	5096	15	chr7	53290465	53295708	5243	HiConf	Variation_32886	chr7	53290720	53293800	3080	Perry et al. (2008)	Agilent Custom CGH Arrays	
2649	chr7	53422747	53434752	12005	3	chr7	53422716	53434966	12250	HiConf	Variation_38684	chr7	53423096	53433669	10573	McCarroll et al. (2008)	Affymetrix Human SNP Array 6.0	
2650	chr7	54152945	54158041	5096	5	chr7	54152914	54158157	5243	HiConf	Variation_38685	chr7	54152898	54158714	5816	McCarroll et al. (2008)	Affymetrix Human SNP Array 6.0	
2651	chr7	54260892	54263904	3012	1	chr7	54260861	54273454	12593	single								
2652	chr7	54349288	54351493	2205	3	chr7	54349257	54351511	2254	single								
2653	chr7	54564349	54564839	490	57	chr7	54564318	54564857	539	HiConf	Variation_47372	chr7	54563984	54564943	959	Bentley et al. (2008)	Illumina DNA sequencing	
2654	chr7	54636575	54636975	400	7	chr7	54636544	54636985	441	single								
2655	chr7	55758038	55760243	2205	7	chr7	55758007	55760261	2254	single								
100451	chr7	56393411	56409170	15759	1	chr7	56346130	56458658	112528	single	Variation_36683	chr7	56406806	56409909	3103	Kidd et al. (2008)	Paired End Mapping	
2656	chr7	56527093	56528465	1372	18	chr7	56527062	56528483	1421	single	Variation_8558	chr7	56292285	57331131	1038846	Pinto et al. (2007)	Affymetrix 500K SNP Mapping Array	
2657	chr7	56729561	56739018	9457	36	chr7	56729530	56739183	9653	HiConf	Variation_38062	chr7	56729607	56736308	6701	McCarroll et al. (2008)	Affymetrix Human SNP Array 6.0	
2658	chr7	56864115	56885021	20906	13	chr7	56864084	56885546	21462	HiConf	Variation_31343	chr7	56846336	56864476	18140	Perry et al. (2008)	Agilent Custom CGH Arrays	
2659	chr7	56912919	56916545	3626	12	chr7	56912888	56917837	4949	single	Variation_0720	chr7	56749798	56932979	183181	Sharp et al. (2005)	BAC Array CGH	

locus_id	chrom	start	end	length	Yoruba w/ event	putative chr	putative start	putative end	putative len	putative type	variation_id	DGV_chr	DGV_start	DGV_end	DGV_len	Reference	Method/platform	complex
2660	chr7	56951776	56954863	3087	3	chr7	56951745	56954930	3185	HiConf	Variation_8558	chr7	56292285	57331131	1038846	Pinto et al. (2007)	Affymetrix 500K SNP Mapping Array	
2661	chr7	57068053	57084664	16611	6	chr7	57068022	57084976	16954	HiConf	Variation_8558	chr7	56292285	57331131	1038846	Pinto et al. (2007)	Affymetrix 500K SNP Mapping Array	
2662	chr7	57292963	57307646	14683	3	chr7	57292932	57308024	15092	HiConf	Variation_8559	chr7	57279775	57923922	644147	Pinto et al. (2007)	Affymetrix 500K SNP Mapping Array	
100452	chr7	57881060	58047874	166814	18	chr7	57586981	59540317	1953336	HiConf	Variation_31345	chr7	57881466	57917096	35630	Perry et al. (2008)	Agilent Custom CGH Arrays	
2667	chr7	61060034	61075469	15435	2	chr7	61060003	61075781	15778	single								
2668	chr7	61092178	61300232	208054	20	chr7	61092147	61304464	212317	HiConf	Variation_4538	chr7	61094178	61279220	185042	Wong et al. (2007)	BAC Array CGH	
2669	chr7	61378093	61396664	18571	30	chr7	61378062	61397025	18963	HiConf	Variation_34445	chr7	61269841	62474368	1204527	Zogopoulos et al. (2007)	Affymetrix 500K and 100K SNP Mapping Arrays	
2670	chr7	61418667	61439637	20970	35	chr7	61418585	61440047	21462	HiConf	Variation_1150	chr7	61416760	61438239	21479	Conrad et al. (2005)	Mendelian inconsistencies	
2671	chr7	61446595	61484080	37485	1	chr7	61446564	61484833	38269	HiConf	Variation_37907	chr7	61477137	61488622	11485	McCarroll et al. (2008)	Affymetrix Human SNP Array 6.0	
2672	chr7	61607560	61611480	3920	18	chr7	61607529	61611547	4018	single	Variation_5237	chr7	61534850	62234750	699900	Simon-Sanchez et al. (2007)	Illumina HumanHap300 BeadChip	y
2673	chr7	61612607	62068154	455547	20	chr7	61612576	62075185	462609	single	Variation_5237	chr7	61534850	62234750	699900	Simon-Sanchez et al. (2007)	Illumina HumanHap300 BeadChip	
2674	chr7	62458837	62471087	12250	3	chr7	62458806	62471350	12544	single	Variation_32901	chr7	62467771	62473004	5233	Perry et al. (2008)	Agilent Custom CGH Arrays	
2675	chr7	62663020	62681836	18816	4	chr7	62662989	62682197	19208	HiConf	Variation_7635	chr7	62622908	62887314	264406	de Smith et al. (2007)	Agilent 185k CGH Arrays/Agilent Custom CGH Arrays	
2676	chr7	62755140	62758227	3087	3	chr7	62755109	62758294	3185	HiConf	Variation_7635	chr7	62622908	62887314	264406	de Smith et al. (2007)	Agilent 185k CGH Arrays/Agilent Custom CGH Arrays	
2677	chr7	62786206	62787578	1372	16	chr7	62786175	62787596	1421	single	Variation_6549	chr7	62785788	62787052	1264	Mills et al. (2006)	Sequence trace read mapping	
2678	chr7	62825926	62869702	43776	40	chr7	62824983	62870602	45619	HiConf	Variation_36693	chr7	62841249	62880377	39128	Kidd et al. (2008)	Paired End Mapping	
2679	chr7	62958441	62965448	7007	6	chr7	62958410	62965564	7154	HiConf								
2680	chr7	63573685	63573793	108	18	chr7	63573654	63573801	147	single	Variation_5239	chr7	63347174	64269776	922602	Simon-Sanchez et al. (2007)	Illumina HumanHap300 BeadChip	
2681	chr7	64357292	64359743	2451	23	chr7	64356919	64359810	2891	HiConf	Variation_31352	chr7	64357774	64358939	1165	Perry et al. (2008)	Agilent Custom CGH Arrays	y
2682	chr7	64363810	64364210	400	26	chr7	64363779	64364220	441	single	Variation_33844	chr7	64363668	64363934	266	Perry et al. (2008)	Agilent Custom CGH Arrays	
2683	chr7	64454656	64461189	6533	16	chr7	64454625	64461583	6958	HiConf	Variation_34594	chr7	64365513	64555413	189900	Zogopoulos et al. (2007)	Affymetrix 500K and 100K SNP Mapping Arrays	
2684	chr7	64593228	64656357	63129	6	chr7	64593197	64657681	64484	HiConf	Variation_32910	chr7	64623990	64648811	24821	Perry et al. (2008)	Agilent Custom CGH Arrays	
2685	chr7	64758456	64776595	18139	10	chr7	64758425	64776800	18375	single	Variation_32911	chr7	64656954	64783369	126415	Perry et al. (2008)	Agilent Custom CGH Arrays	
2686	chr7	66335080	66336158	1078	25	chr7	66335049	66336176	1127	single	Variation_44317	chr7	66334933	66336829	1896	Bentley et al. (2008)	Illumina DNA sequencing	
2687	chr7	66379131	66402915	23784	9	chr7	66379100	66403453	24353	HiConf	Variation_31354	chr7	66373260	66400012	26752	Perry et al. (2008)	Agilent Custom CGH Arrays	
100454	chr7	66503058	66511386	8328	2	chr7	66480555	66515565	35010	single	Variation_7642	chr7	66492870	66596333	103463	de Smith et al. (2007)	Agilent 185k CGH Arrays/Agilent Custom CGH Arrays	
2689	chr7	66983203	66989354	6151	4	chr7	66983172	66989738	6566	HiConf								
2690	chr7	67840458	67841438	980	8	chr7	67840427	67841456	1029	single								
2691	chr7	68436249	68437817	1568	3	chr7	68436218	68437835	1617	single								
100455	chr7	69951158	70016296	65138	9	chr7	69951127	70017742	66615	HiConf	Variation_29784	chr7	69959579	70013220	53641	Jakobsson et al. (2008)	Illumina HumanHap550 BeadChip	
2693	chr7	70059001	70063686	4685	37	chr7	70059000	70063753	4753	HiConf	Variation_38800	chr7	70058925	70064077	5152	McCarroll et al. (2008)	Affymetrix Human SNP Array 6.0	
2694	chr7	70257902	70262479	4577	4	chr7	70257842	70262546	4704	HiConf								y
2695	chr7	70772813	70793345	20532	55	chr7	70772097	70793559	21462	HiConf								y
2696	chr7	71207052	71213079	6027	25	chr7	71207021	71213195	6174	single								
2697	chr7	71215774	71219057	3283	16	chr7	71215743	71219124	3381	HiConf								
2698	chr7	71294125	71319990	25865	22	chr7	71294094	71320456	26362	HiConf								
2699	chr7	71459157	71461411	2254	8	chr7	71459126	71461429	2303	HiConf								
2700	chr7	71711164	71713663	2499	1	chr7	71711133	71713730	2597	single	Variation_8566	chr7	71645034	71906230	261196	Pinto et al. (2007)	Affymetrix 500K SNP Mapping Array	
2701	chr7	71713878	71725864	11986	3	chr7	71713877	71726078	12201	HiConf	Variation_8566	chr7	71645034	71906230	261196	Pinto et al. (2007)	Affymetrix 500K SNP Mapping Array	
2702	chr7	71908193	71919169	10976	51	chr7	71908162	71919383	11221	HiConf	Variation_36707	chr7	71907038	71919062	12024	Kidd et al. (2008)	Paired End Mapping	
2703	chr7	72115316	72164494	49178	18	chr7	72115285	72165314	50029	HiConf	Variation_32915	chr7	72115995	72156215	40220	Perry et al. (2008)	Agilent Custom CGH Arrays	
2704	chr7	73467099	73469149	2050	61	chr7	73467097	73469645	2548	HiConf	Variation_23850	chr7	73466431	73469249	2818	Levy et al. (2007)	Sequencing	
2705	chr7	73941791	73971246	29455	14	chr7	73941760	73972385	30625	HiConf	Variation_32918	chr7	73946263	73972194	25931	Perry et al. (2008)	Agilent Custom CGH Arrays	
2706	chr7	74240152	74242896	2744	11	chr7	74240121	74242963	2842	single	Variation_4544	chr7	74151477	74293398	141921	Wong et al. (2007)	BAC Array CGH	
100457	chr7	74741422	74988774	247352	5	chr7	74741342	74993839	252497	HiConf	Variation_4545	chr7	74789544	74903501	113957	Wong et al. (2007)	BAC Array CGH	
2708	chr7	75966422	75971910	5488	4	chr7	75966391	75972026	5635	single	Variation_36708	chr7	75971699	76021246	49547	Kidd et al. (2008)	Paired End Mapping	
2709	chr7	75989403	76015107	25704	27	chr7	75989372	76015734	26362	HiConf	Variation_31371	chr7	75991887	76010473	18586	Perry et al. (2008)	Agilent Custom CGH Arrays	
2710	chr7	76016451	76022919	6468	12	chr7	76016420	76023035	6615	HiConf	Variation_0611	chr7	75980534	76020295	39761	Tuzun et al. (2005)	Paired End Mapping	
2711	chr7	76023605	76027721	4116	14	chr7	76023574	76027788	4214	single	Variation_36710	chr7	75981671	76028269	46598	Kidd et al. (2008)	Paired End Mapping	
2712	chr7	76036443	76042519	6076	8	chr7	76036412	76042635	6223	HiConf	Variation_29785	chr7	76023564	76395148	371584	Jakobsson et al. (2008)	Illumina HumanHap550 BeadChip	
2713	chr7	76042837	76068293	25456	9	chr7	76042831	76068801	25970	HiConf	Variation_32926	chr7	76046675	76052686	6011	Perry et al. (2008)	Agilent Custom CGH Arrays	
2714	chr7	76476925	76538226	61301	21	chr7	76476873	76539495	62622	HiConf	Variation_31372	chr7	76481460	76539982	58522	Perry et al. (2008)	Agilent Custom CGH Arrays	
2715	chr7	76617191	76626697	9506	8	chr7	76617160	76626862	9702	single	Variation_32930	chr7	76591834	76630197	38363	Perry et al. (2008)	Agilent Custom CGH Arrays	
2716	chr7	76642377	76643700	1323	10	chr7	76642346	76643718	1372	HiConf	Variation_33849	chr7	76642409	76643205	796	Perry et al. (2008)	Agilent Custom CGH Arrays	
100459	chr7	78637976	78691386	53410	10	chr7	78637945	78692457	54512	HiConf	Variation_32931	chr7	78642750	78691419	48669	Perry et al. (2008)	Agilent Custom CGH Arrays	
100460	chr7	79207269	79218221	10952	1	chr7	79190175	79251033	60858	single	Variation_3687	chr7	79099400	79265582	166182	Redon et al. (2006)	BAC Array CGH	y
2718	chr7	79307291	79319345	12054	55	chr7	79307260	79319608	12348	HiConf	Variation_44319	chr7	79309556	79319607	10051	Bentley et al. (2008)	Illumina DNA sequencing	
2719	chr7	80440318	80440678	360	12	chr7	80440287	80440679	392	HiConf								
2720	chr7	80526509	80533565	7056	3	chr7	80526478	80533681	7203	single								
2721	chr7	81279640	81280424	784	65	chr7	81279608	81280441	833	HiConf	Variation_28693	chr7	81279609	81280516	907	Levy et al. (2007)	Sequencing	
2722	chr7	81523414	81526697	3283	1	chr7	81523383	81526764	3381	single								
2723	chr7	81884593	81886945	2352	4	chr7	81884562	81886963	2401	HiConf	Variation_8571	chr7	81795573	82298885	503312	Pinto et al. (2007)	Affymetrix 500K SNP Mapping Array	
2724	chr7	81965051	81966689	1638	3	chr7	81965020	81968205	3185	single	Variation_8571	chr7	81795573	82298885	503312	Pinto et al. (2007)	Affymetrix 500K SNP Mapping Array	
2725	chr7	82300211	82302073	1862	6	chr7	82300180	82302091	1911	HiConf								
100461	chr7	82572259	82644289	72030	1	chr7	82572228	82645753	73525	single								
2726	chr7	82888652	82889052	400	17	chr7	82888621	82889062	441	HiConf	Variation_11305	chr7	82888647	82888951	304	de Smith et al. (2007)	Agilent 185k CGH Arrays/Agilent Custom CGH Arrays	
2727	chr7	83620124	83624643	4519	2	chr7	83620093	83624699	4606	HiConf	Variation_38690	chr7	83620369	83624624	4255	McCarroll et al. (2008)	Affymetrix Human SNP Array 6.0	
2728	chr7	84712579	84716842	4263	5	chr7	84712548	84716909	4361	single								y
2729	chr7	87066637	87100202	33565	2	chr7	87066606	87100857	34251	single								
2730	chr7	87702314	87707655	5341	14	chr7	87702283	87707771	5488	HiConf								
100462	chr7	87971120	88092305	121185	1	chr7	87971048	88094773	123725	single	Variation_10200	chr7	87974520	88092199	117679	Wang et al. (2007)	Illumina HumanHap550 BeadChip	y
2732	chr7	89377232	89410886	33654	49	chr7	89377201	89411256	34055	HiConf	Variation_43075	chr7	89391993	89392684	691	Wang et al. (2008)	Illumina DNA sequencing	
2733	chr7	89455387	89457250	1863	5	chr7	89455356	89457267	1911	HiConf	Variation_5399	chr7	89237888	89522125	284237	Simon-Sanchez et al. (2007)	Illumina HumanHap300 BeadChip	
2734	chr7	89648212	89650901	2689	59	chr7	89648171	89650915	2744	HiConf	Variation_39498	chr7	89648345	89650537	2192	Wheeler et al. (2008)	Sequencing	
2735	chr7	90263985	90265553	1568	11	chr7	90263954	90265571	1617	HiConf								
2736	chr7	90480369	90504575	24206	14	chr7	90480338	90505083	24745	HiConf								
2737	chr7	91093212	91098014	4802	3	chr7	91093181	91098130	4949	single								
2738	chr7	91196504	91198464	1960	18	chr7	91196473	91198482	2009	HiConf	Variation_10799	chr7	91196798	91196930	132	Conrad et al. (2005)	Mendelian inconsistencies	
2739	chr7	91400295	91402598	2303	11	chr7	91400264	91402616	2352	HiConf								
100465	chr7	91979230	92074241	95011	2	chr7	91979199	92076170	96971	single								
2740	chr7	92842806	92845158	2352	1	chr7	92842775	92845176	2401	single								
2741	chr7	93011170	93013571	2401	2	chr7	93011139	93013638	2499	HiConf								
2742	chr7	93164027	93170752	6725	27	chr7	93164019	93170830	6811	HiConf	Variation_37453	chr7	93165082	93170173	5091	Cooper et al. (2008)	Illumina Human 1M BeadChip	y
2743	chr7	93340303	93398729	58426	73	chr7	93340272	93399121	58849	HiConf								
2744	chr7	94550897	94558341	7444	3	chr7	94550866	94558510	7644	HiConf								
100467	chr7	95051614	95067224	15610	1	chr7	95004753	95082810	78057	single								

locus_id	chrom	start	end	length	Yoruba w/ event	putative chr	putative start	putative end	putative len	putative type	variation_id	DGV_chr	DGV_start	DGV_end	DGV_len	Reference	Method/platform	complex
2745	chr7	95954257	95955286	1029	41	chr7	95954226	95955304	1078	HiConf								
2746	chr7	96380900	96384232	3332	73	chr7	96380869	96384299	3430	HiConf	Variation_39502	chr7	96364773	96390745	25972	Wheeler et al. (2008)	Sequencing	
2747	chr7	96426764	96429214	2450	7	chr7	96426733	96429281	2548	single								
2748	chr7	96604634	96637562	32928	1	chr7	96604603	96638217	33614	single								
2749	chr7	96638444	96651743	13299	1	chr7	96638413	96659875	21462	single								
2750	chr7	96691511	96695186	3675	5	chr7	96691480	96695253	3773	HiConf								
2751	chr7	97234529	97240333	5804	9	chr7	97234498	97240427	5929	HiConf	Variation_1733	chr7	97234645	97238934	4289	McCarroll et al. (2005)	Mendelian inconsistencies/Null genotypes	
2752	chr7	97342868	97386884	44016	8	chr7	97342837	97387819	44982	HiConf	Variation_3691	chr7	97314220	97485965	171745	Redon et al. (2006)	BAC Array CGH	
2753	chr7	97388438	97392995	4557	9	chr7	97388407	97393062	4655	single	Variation_32935	chr7	97392003	97398068	6065	Perry et al. (2008)	Agilent Custom CGH Arrays	
2754	chr7	98066451	98067774	1323	23	chr7	98066420	98067792	1372	single	Variation_44321	chr7	98066484	98068687	2203	Bentley et al. (2008)	Illumina DNA sequencing	y
2755	chr7	98241920	98242358	438	15	chr7	98241889	98242379	490	single								
2756	chr7	98258188	98272692	14504	2	chr7	98258157	98273004	14847	single								
2757	chr7	99299291	99301447	2156	53	chr7	99299260	99301465	2205	HiConf								
2758	chr7	99735979	99774496	38517	24	chr7	99735948	99775050	39102	HiConf	Variation_31379	chr7	99737488	99773734	36246	Perry et al. (2008)	Agilent Custom CGH Arrays	
2759	chr7	100167179	100175656	8477	10	chr7	100167148	100175821	8673	HiConf	Variation_38035	chr7	100167180	100170778	3598	McCarroll et al. (2008)	Affymetrix Human SNP Array 6.0	
2760	chr7	100187465	100188543	1078	33	chr7	100187434	100188561	1127	HiConf	Variation_4550	chr7	100000439	100200771	200332	Wong et al. (2007)	BAC Array CGH	
2761	chr7	100311239	100318001	6762	14	chr7	100311208	100318117	6909	single	Variation_3692	chr7	100193426	100535966	342540	Redon et al. (2006)	BAC Array CGH	y
100469	chr7	100330846	100452866	122020	19	chr7	100330392	100453063	122671	HiConf	Variation_0617	chr7	100430303	100448907	18604	Tuzun et al. (2005)	Paired End Mapping	
2763	chr7	100461865	100472694	10829	13	chr7	100461834	100472908	11074	HiConf	Variation_31382	chr7	100462678	100471602	8924	Perry et al. (2008)	Agilent Custom CGH Arrays	
2764	chr7	101468178	101470677	2499	4	chr7	101468147	101470744	2597	HiConf	Variation_31383	chr7	101469341	101470578	1237	Perry et al. (2008)	Agilent Custom CGH Arrays	
2765	chr7	101761247	101796870	35623	8	chr7	101761216	101797574	36358	single	Variation_31384	chr7	101763307	101786113	22806	Perry et al. (2008)	Agilent Custom CGH Arrays	
2766	chr7	101901142	102121334	220192	31	chr7	101901111	102123326	222215	HiConf	Variation_31385	chr7	101884173	102119482	235309	Perry et al. (2008)	Agilent Custom CGH Arrays	
2767	chr7	102139233	102148739	9506	19	chr7	102139202	102148904	9702	HiConf	Variation_37916	chr7	102139082	102145851	6769	McCarroll et al. (2008)	Affymetrix Human SNP Array 6.0	
2768	chr7	102407263	102411615	4352	2	chr7	102407232	102411691	4459	single	Variation_9065	chr7	102145556	102574371	428815	Pinto et al. (2007)	Affymetrix 500K SNP Mapping Array	
2769	chr7	102562495	102563622	1127	6	chr7	102562464	102563640	1176	HiConf	Variation_9065	chr7	102145556	102574371	428815	Pinto et al. (2007)	Affymetrix 500K SNP Mapping Array	y
2770	chr7	102569943	102597341	27398	60	chr7	102569912	102597499	27587	HiConf	Variation_36598	chr7	102576452	102594677	18225	Kidd et al. (2008)	Paired End Mapping	
2771						chr7	102634984	102712943	77959	single								
2772	chr7	103559841	103562683	2842	3	chr7	103559810	103562750	2940	single								
2773	chr7	104252769	104263530	10761	6	chr7	104252768	104263744	10976	HiConf	Variation_32943	chr7	104252732	104263691	10959	Perry et al. (2008)	Agilent Custom CGH Arrays	
100472	chr7	104641418	104754363	112945	1	chr7	104641387	104756684	115297	single	Variation_3694	chr7	104553943	104897817	343874	Redon et al. (2006)	BAC Array CGH	
2775	chr7	106029882	106034935	5053	10	chr7	106029851	106035045	5194	single								
100473	chr7	106981576	107013316	31740	2	chr7	106961929	107023277	61348	single								
2776	chr7	108092047	108093125	1078	11	chr7	108092016	108093143	1127	single								
2777	chr7	109216597	109249917	33320	12	chr7	109216566	109250572	34006	HiConf	Variation_0619	chr7	109214180	109249168	34988	Tuzun et al. (2005)	Paired End Mapping	
2778	chr7	109555579	109559009	3430	3	chr7	109555548	109559076	3528	HiConf	Variation_3696	chr7	109550832	109699468	148636	Redon et al. (2006)	BAC Array CGH	
100474	chr7	109968242	109997115	28873	66	chr7	109968104	109997528	29424	HiConf	Variation_36607	chr7	109966683	109995470	28787	Kidd et al. (2008)	Paired End Mapping	
2780	chr7	110215413	110219627	4214	6	chr7	110215382	110219694	4312	HiConf	Variation_38651	chr7	110216246	110219730	3484	McCarroll et al. (2008)	Affymetrix Human SNP Array 6.0	
2781	chr7	110770093	110770926	833	4	chr7	110770062	110770944	882	HiConf	Variation_9067	chr7	110768285	110901285	133000	Pinto et al. (2007)	Affymetrix 500K SNP Mapping Array	
2782	chr7	110808215	110809832	1617	3	chr7	110808184	110809850	1666	HiConf	Variation_33854	chr7	110808261	110808902	641	Perry et al. (2008)	Agilent Custom CGH Arrays	
100475	chr7	110834161	110878996	44835	6	chr7	110834130	110879896	45766	HiConf	Variation_1163	chr7	110835815	110867643	31828	Conrad et al. (2005)	Mendelian inconsistencies	
2784	chr7	110882156	110884802	2646	5	chr7	110882125	110884869	2744	single	Variation_31390	chr7	110882245	110885339	3094	Perry et al. (2008)	Agilent Custom CGH Arrays	
2786	chr7	111019650	111020050	400	37	chr7	111019619	111020060	441	HiConf	Variation_9069	chr7	111016229	111123819	107590	Pinto et al. (2007)	Affymetrix 500K SNP Mapping Array	
2787	chr7	111035085	111044325	9240	11	chr7	111035054	111044413	9359	HiConf	Variation_32946	chr7	111038720	111042719	3999	Perry et al. (2008)	Agilent Custom CGH Arrays	
2788	chr7	111800024	111802425	2401	3	chr7	111799993	111802492	2499	single	Variation_5246	chr7	111360255	112415163	1054908	Simon-Sanchez et al. (2007)	Illumina HumanHap300 BeadChip	
100477	chr7	112270718	112404537	133819	2	chr7	112270687	112407250	136563	HiConf	Variation_0108	chr7	112297910	112436546	138636	Iafrate et al. (2004)	BAC Array CGH	
2790	chr7	112667576	112675948	8372	6	chr7	112658081	112676309	18228	single	Variation_23248	chr7	112405812	112921258	515446	Korbel et al. (2007)	Paired End Mapping	
2791	chr7	113440299	113446326	6027	7	chr7	113440268	113446442	6174	HiConf	Variation_22774	chr7	113439761	113447716	7955	Korbel et al. (2007)	Paired End Mapping	
2792	chr7	114770845	114771155	310	4	chr7	114770814	114771157	343	single								
2793	chr7	115117961	115120901	2940	6	chr7	115117930	115120968	3038	HiConf								
100478	chr7	115597132	115647349	50217	3	chr7	115597101	115648355	51254	single								
100479	chr7	115912300	115923560	11260	1	chr7	115912269	115968595	56326	single								
100480	chr7	117920178	117950179	30001	5	chr7	117917864	117975561	57697	HiConf	Variation_37458	chr7	117919581	117950473	30892	Cooper et al. (2008)	Illumina Human 1M BeadChip	
2795	chr7	118170220	118178207	7987	3	chr7	118170189	118178372	8183	HiConf	Variation_22818	chr7	118169275	118178489	9214	Korbel et al. (2007)	Paired End Mapping	
2796	chr7	118618542	118621853	3311	46	chr7	118618539	118621920	3381	HiConf	Variation_6567	chr7	118618496	118621990	3494	Mills et al. (2006)	Sequence trace read mapping	
2797	chr7	118941235	118949761	8526	15	chr7	118941204	118949926	8722	HiConf	Variation_32947	chr7	118941522	118949587	8065	Perry et al. (2008)	Agilent Custom CGH Arrays	
2798	chr7	119020174	119023310	3136	8	chr7	119020143	119023377	3234	HiConf								
2799	chr7	119023555	119024675	1120	3	chr7	119023524	119025288	1764	single								
2800	chr7	119161833	119173348	11515	2	chr7	119161802	119173562	11760	HiConf	Variation_36614	chr7	119137276	119176877	39601	Kidd et al. (2008)	Paired End Mapping	
100481	chr7	119360626	119430717	70091	3	chr7	119360595	119432111	71516	single	Variation_4555	chr7	119372405	119563855	191450	Wong et al. (2007)	BAC Array CGH	y
2802	chr7	119435637	119449267	13630	15	chr7	119435026	119449530	14504	HiConf	Variation_4555	chr7	119372405	119563855	191450	Wong et al. (2007)	BAC Array CGH	
2803	chr7	119513555	119518161	4606	3	chr7	119513524	119518228	4704	single	Variation_4555	chr7	119372405	119563855	191450	Wong et al. (2007)	BAC Array CGH	
2804	chr7	120212393	120213471	1078	1	chr7	120212362	120213489	1127	single								
2805	chr7	120435147	120436029	882	1	chr7	120435116	120436047	931	single								
2806	chr7	121128252	121130016	1764	5	chr7	121128221	121130034	1813	HiConf								
2807	chr7	122219188	122237563	18375	6	chr7	122219157	122237924	18767	single								
100482	chr7	122576447	122675946	99499	48	chr7	122576416	122676646	100230	single	Variation_2712	chr7	122585872	122676571	90699	Redon et al. (2006)	Affymetrix 500K EA SNP Mapping Array	
2808	chr7	124424286	124424953	667	6	chr7	124424255	124425725	1470	single								
2809	chr7	125257727	125264440	6713	4	chr7	125257696	125264556	6860	HiConf	Variation_38660	chr7	125259938	125263279	3341	McCarroll et al. (2008)	Affymetrix Human SNP Array 6.0	
2810	chr7	125833156	125838777	5621	55	chr7	125833152	125838787	5635	HiConf	Variation_43591	chr7	125833088	125838731	5643	Wang et al. (2008)	Illumina DNA sequencing	
2811	chr7	125879929	125882036	2107	1	chr7	125879898	125882054	2156	single								
2812	chr7	126058485	126065198	6713	7	chr7	126058454	126065314	6860	HiConf	Variation_36618	chr7	126057557	126060959	3402	Kidd et al. (2008)	Paired End Mapping	
2813	chr7	126563381	126568477	5096	4	chr7	126563350	126568593	5243	HiConf	Variation_32950	chr7	126563587	126568056	4469	Perry et al. (2008)	Agilent Custom CGH Arrays	
2814	chr7	127002236	127005378	3142	8	chr7	127002194	127005379	3185	HiConf	Variation_43521	chr7	127002157	127005306	3149	Wang et al. (2008)	Illumina DNA sequencing	
100483	chr7	128232251	128274878	42627	5	chr7	128227807	128301307	73500	HiConf	Variation_9584	chr7	128245885	128294533	48648	Wang et al. (2007)	Illumina HumanHap550 BeadChip	
2815	chr7	131045117	131046538	1421	25	chr7	131045086	131046556	1470	HiConf	Variation_44289	chr7	131044880	131047078	2198	Bentley et al. (2008)	Illumina DNA sequencing	
2816	chr7	131210884	131212942	2058	12	chr7	131210853	131212960	2107	HiConf								
2817	chr7	131307218	131311481	4263	3	chr7	131307187	131311548	4361	single								
2818	chr7	131652325	131652685	360	20	chr7	131652294	131652686	392	single								y
2819	chr7	131889044	131903178	14134	15	chr7	131889013	131903419	14406	single								
2820	chr7	132141100	132145412	4312	3	chr7	132141069	132145479	4410	single	Variation_3703	chr7	131972230	132195023	222793	Redon et al. (2006)	BAC Array CGH	
2821	chr7	132503406	132505758	2352	8	chr7	132503375	132505776	2401	single								
2822	chr7	133435406	133449694	14288	18	chr7	133434767	133450006	15239	HiConf	Variation_38107	chr7	133435735	133449694	13959	McCarroll et al. (2008)	Affymetrix Human SNP Array 6.0	
2823	chr7	133882217	133885108	2891	6	chr7	133882186	133885175	2989	HiConf								
2824	chr7	133961205	133962626	1421	12	chr7	133961174	133962644	1470	HiConf								
2825	chr7	135805761	135808296	2535	2	chr7	135805730	135809013	3283	single								
100484	chr7	136476496	136517365	40869	4	chr7	136472449	136535316	62867	single								

locus_id	chrom	start	end	length	Yoruba w/ event	putative chr	putative start	putative end	putative len	putative type	variation_id	DGV_chr	DGV_start	DGV_end	DGV_len	Reference	Method/platform	complex
2827	chr7	136586968	136588193	1225	7	chr7	136586937	136588211	1274	HiConf	Variation_7605	chr7	136570553	137618389	1047836	de Smith et al. (2007)	Agilent 185k CGH Arrays/Agilent Custom CGH Arrays	
2828	chr7	137522623	137523750	1127	3	chr7	137522592	137523768	1176	single	Variation_47950	chr7	137374632	137863659	489027	Gusev et al. (2009)	SNP genotyping analysis	
2829	chr7	138520606	138521341	735	13	chr7	138520575	138521359	784	HiConf	Variation_3705	chr7	138384237	138577879	193642	Redon et al. (2006)	BAC Array CGH	
2830	chr7	139841303	139848457	7154	6	chr7	139841272	139848573	7301	HiConf	Variation_32951	chr7	139839959	139848533	8574	Perry et al. (2008)	Agilent Custom CGH Arrays	
2831	chr7	140681510	140682780	1270	24	chr7	140681475	140682798	1323	single								
2832	chr7	140919254	140921116	1862	5	chr7	140919223	140921134	1911	single	Variation_47923	chr7	140693923	141139367	445444	Gusev et al. (2009)	SNP genotyping analysis	y
100485	chr7	141685060	141736269	51209	43	chr7	141684995	141736470	51475	HiConf	Variation_36631	chr7	141689906	141732089	42183	Kidd et al. (2008)	Paired End Mapping	
2834	chr7	141904791	141908221	3430	4	chr7	141904760	141908288	3528	HiConf	Variation_1746	chr7	141896503	141908580	12077	McCarroll et al. (2005)	Mendelian inconsistencies	
100486	chr7	142128060	142190403	62343	9	chr7	142128029	142190896	62867	HiConf	Variation_10216	chr7	142114524	142192134	77610	Wang et al. (2007)	Illumina HumanHap550 BeadChip	
2836	chr7	142582363	142593241	10878	4	chr7	142582332	142593455	11123	HiConf								
2837	chr7	142930998	143028376	97378	20	chr7	142930967	143030192	99225	HiConf	Variation_2107	chr7	142959070	143028681	69611	Locke et al. (2006)	BAC Array CGH	
2838	chr7	143030394	143053612	23218	35	chr7	143030388	143054055	23667	HiConf	Variation_31396	chr7	142927418	143056093	128675	Perry et al. (2008)	Agilent Custom CGH Arrays	
2839	chr7	143054546	143064278	9732	23	chr7	143054545	143064492	9947	single	Variation_9587	chr7	143056311	143063852	7541	Wang et al. (2007)	Illumina HumanHap550 BeadChip	
2840	chr7	143066777	143100293	33516	23	chr7	143066746	143100948	34202	HiConf	Variation_2108	chr7	142956491	143108279	151788	Locke et al. (2006)	BAC Array CGH	
2841	chr7	143101150	143134936	33786	32	chr7	143101144	143135640	34496	single	Variation_31397	chr7	143085896	143202384	116488	Perry et al. (2008)	Agilent Custom CGH Arrays	
2842	chr7	143136081	143137793	1712	39	chr7	143136081	143137796	1715	single	Variation_31397	chr7	143085896	143202384	116488	Perry et al. (2008)	Agilent Custom CGH Arrays	
2843	chr7	143137998	143144785	6787	23	chr7	143137992	143144901	6909	single	Variation_31397	chr7	143085896	143202384	116488	Perry et al. (2008)	Agilent Custom CGH Arrays	
2844	chr7	143145049	143163405	18356	34	chr7	143145048	143163766	18718	single	Variation_7611	chr7	143146453	143178809	32356	de Smith et al. (2007)	Agilent 185k CGH Arrays/Agilent Custom CGH Arrays	
2845	chr7	143166737	143201998	35261	36	chr7	143166706	143202672	35966	HiConf	Variation_7611	chr7	143146453	143178809	32356	de Smith et al. (2007)	Agilent 185k CGH Arrays/Agilent Custom CGH Arrays	
2846	chr7	143315746	143321185	5439	6	chr7	143315715	143321301	5586	HiConf	Variation_1171	chr7	143315921	143319933	4012	Conrad et al. (2005)	Mendelian inconsistencies	y
2847	chr7	143506356	143592809	86453	60	chr7	143506325	143593839	87514	HiConf	Variation_7612	chr7	143514986	143584377	69391	de Smith et al. (2007)	Agilent 185k CGH Arrays/Agilent Custom CGH Arrays	
2848	chr7	143594086	143704218	110132	31	chr7	143594084	143706441	112357	HiConf	Variation_0726	chr7	143536879	143690749	153870	Sharp et al. (2005)	BAC Array CGH	y
2849	chr7	143714606	143715194	588	18	chr7	143714575	143715212	637	single	Variation_4573	chr7	143665199	143839638	174439	Wong et al. (2007)	BAC Array CGH	
2850	chr7	144401341	144408054	6713	20	chr7	144401310	144408170	6860	HiConf								
2851	chr7	145424706	145435986	11280	3	chr7	145424675	145436190	11515	single								
2852	chr7	145764570	145770303	5733	13	chr7	145764539	145770419	5880	HiConf	Variation_44294	chr7	145764702	145770461	5759	Bentley et al. (2008)	Illumina DNA sequencing	
2853	chr7	147640143	147683557	43414	15	chr7	147640112	147684457	44345	HiConf	Variation_36644	chr7	147634092	147666381	32289	Kidd et al. (2008)	Paired End Mapping	
2854	chr7	147704088	147708319	4231	44	chr7	147704057	147708320	4263	HiConf	Variation_7614	chr7	147704229	147707190	2961	de Smith et al. (2007)	Agilent 185k CGH Arrays/Agilent Custom CGH Arrays	
2855	chr7	149345490	149347205	1715	22	chr7	149345459	149347223	1764	HiConf	Variation_7615	chr7	149309917	149383517	73600	de Smith et al. (2007)	Agilent 185k CGH Arrays/Agilent Custom CGH Arrays	
2856	chr7	149493813	149512482	18669	16	chr7	149493782	149512843	19061	HiConf	Variation_32964	chr7	149493995	149512717	18722	Perry et al. (2008)	Agilent Custom CGH Arrays	
2857	chr7	149625525	149635129	9604	3	chr7	149625494	149635294	9800	single								
2858	chr7	150442033	150442303	270	13	chr7	150441932	150442373	441	single	Variation_4574	chr7	150366138	150543808	177670	Wong et al. (2007)	BAC Array CGH	
2859						chr7	150443892	150445607	1715	single								
2860	chr7	151540298	151577739	37441	5	chr7	151540267	151578487	38220	HiConf	Variation_31401	chr7	151552498	151587195	34697	Perry et al. (2008)	Agilent Custom CGH Arrays	
2861	chr7	151583566	151619678	36112	1	chr7	151583534	151620431	36897	HiConf	Variation_31401	chr7	151552498	151587195	34697	Perry et al. (2008)	Agilent Custom CGH Arrays	
2862	chr7	151709005	151723179	14174	10	chr7	151708974	151723870	14896	HiConf								
2863	chr7	151729781	151742608	12827	7	chr7	151729750	151743274	13524	single								
2864	chr7	151922351	151927937	5586	2	chr7	151922320	151928053	5733	single								
2865	chr7	152205669	152210961	5292	37	chr7	152205638	152211077	5439	HiConf	Variation_36649	chr7	152202069	152216751	14682	Kidd et al. (2008)	Paired End Mapping	
100492	chr7	152773020	152828212	55192	3	chr7	152769383	152829384	60001	HiConf	Variation_38908	chr7	152780054	152825299	45245	McCarroll et al. (2008)	Affymetrix Human SNP Array 6.0	
2867	chr7	153110580	153129025	18445	7	chr7	153110472	153129386	18914	HiConf	Variation_32966	chr7	153109901	153130733	20832	Perry et al. (2008)	Agilent Custom CGH Arrays	
100493	chr7	153324809	153340165	15356	1	chr7	153302626	153387837	85211	single	Variation_0730	chr7	153229742	153389164	159422	Sharp et al. (2005)	BAC Array CGH	
2868	chr7	153406512	153407443	931	30	chr7	153406481	153407461	980	single	Variation_31403	chr7	153114837	153439272	324435	Perry et al. (2008)	Agilent Custom CGH Arrays	
2869	chr7	153550768	153554247	3479	23	chr7	153550737	153554314	3577	HiConf								
2870	chr7	153897247	153898521	1274	5	chr7	153897216	153898539	1323	HiConf								
2871	chr7	154022589	154029774	7185	34	chr7	154022558	154029908	7350	HiConf	Variation_23831	chr7	154022834	154030686	7852	Levy et al. (2007)	Sequencing	y
2872	chr7	154073892	154089830	15938	45	chr7	154073861	154089884	16023	HiConf	Variation_43396	chr7	154085026	154086951	1925	Wang et al. (2008)	Illumina DNA sequencing	
2873	chr7	154341138	154345597	4459	3	chr7	154341107	154345664	4557	single	Variation_0111	chr7	154256381	154418755	162374	Iafrate et al. (2004)	BAC Array CGH	
2874	chr7	154516283	154518887	2604	7	chr7	154516282	154520202	3920	HiConf	Variation_38667	chr7	154515932	154520474	4542	McCarroll et al. (2008)	Affymetrix Human SNP Array 6.0	
2875	chr7	154593733	154595880	2147	7	chr7	154593702	154596250	2548	single	Variation_1173	chr7	154593778	154596312	2534	Conrad et al. (2005)	Mendelian inconsistencies	y
2876	chr7	154831040	154833974	2934	42	chr7	154831009	154833998	2989	HiConf	Variation_39514	chr7	154833257	154834302	1045	Wheeler et al. (2008)	Sequencing	
2877	chr7	154880334	154908166	27832	12	chr7	154880303	154908723	28420	HiConf	Variation_44298	chr7	154892352	154894096	1744	Bentley et al. (2008)	Illumina DNA sequencing	
2878	chr7	155541932	155543990	2058	7	chr7	155541901	155544008	2107	single								
2879	chr7	156096759	156098278	1519	6	chr7	156096728	156098296	1568	single	Variation_3714	chr7	155852149	156118628	266479	Redon et al. (2006)	BAC Array CGH	
2880	chr7	156275511	156276148	637	3	chr7	156275480	156276166	686	HiConf								
2881	chr7	156854201	156855867	1666	40	chr7	156854170	156855885	1715	HiConf	Variation_47290	chr7	156855111	156855336	225	Bentley et al. (2008)	Illumina DNA sequencing	
2882	chr7	157479980	157480780	800	54	chr7	157479949	157480782	833	HiConf	Variation_28535	chr7	157480197	157480439	242	Levy et al. (2007)	Sequencing	
2883	chr7	157630508	157630768	260	49	chr7	157630477	157630771	294	HiConf								
2884	chr7	157644130	157650990	6860	11	chr7	157644099	157651106	7007	HiConf								y
2885	chr7	157939600	157942932	3332	16	chr7	157939569	157942999	3430	single	Variation_42905	chr7	157942691	157943266	575	Wang et al. (2008)	Illumina DNA sequencing	
2886	chr7	158193714	158198598	4884	30	chr7	158193683	158199269	5586	HiConf	Variation_43560	chr7	158193482	158197608	4126	Wang et al. (2008)	Illumina DNA sequencing	y
2887	chr7	158394173	158409721	15548	60	chr7	158394142	158409871	15729	single	Variation_0626	chr7	158386631	158414514	27883	Tuzun et al. (2005)	Paired End Mapping	
2888	chr7	158435970	158436280	310	41	chr7	158435939	158436282	343	single	Variation_34452	chr7	158284285	158819535	535250	Zogopoulos et al. (2007)	Affymetrix 500K and 100K SNP Mapping Arrays	
2889	chr7	158457530	158472426	14896	4	chr7	158457499	158472738	15239	single	Variation_34452	chr7	158284285	158819535	535250	Zogopoulos et al. (2007)	Affymetrix 500K and 100K SNP Mapping Arrays	y
2890	chr7	158545436	158548131	2695	30	chr7	158545405	158548198	2793	single	Variation_34452	chr7	158284285	158819535	535250	Zogopoulos et al. (2007)	Affymetrix 500K and 100K SNP Mapping Arrays	
2891	chr7	158805969	158820816	14847	5	chr7	158805938	158821128	15190	single	Variation_7623	chr7	158810518	158821290	10772	de Smith et al. (2007)	Agilent 185k CGH Arrays/Agilent Custom CGH Arrays	y
2892	chr8	2069	78892	76823	47	chr8	1985	145751	143766	HiConf	Variation_32973	chr8	12850	118483	105633	Perry et al. (2008)	Agilent Custom CGH Arrays	y
2893	chr8	230895	233786	2891	2	chr8	230864	233853	2989	single	Variation_8587	chr8	180568	400640	220072	Pinto et al. (2007)	Affymetrix 500K SNP Mapping Array	
2894	chr8	301357	301519	162	26	chr8	301326	301522	196	single	Variation_8587	chr8	180568	400640	220072	Pinto et al. (2007)	Affymetrix 500K SNP Mapping Array	y
2895	chr8	584332	590358	6026	35	chr8	584301	590377	6076	HiConf	Variation_7695	chr8	584720	590672	5952	de Smith et al. (2007)	Agilent 185k CGH Arrays/Agilent Custom CGH Arrays	
2896	chr8	917140	938259	21119	27	chr8	917109	938669	21560	HiConf	Variation_36890	chr8	910422	939093	28671	Kidd et al. (2008)	Paired End Mapping	
2897	chr8	1011416	1011676	260	43	chr8	1011385	1011679	294	HiConf								
2898	chr8	1056643	1059044	2401	27	chr8	1056612	1059111	2499	single								
2899	chr8	1101625	1101733	108	39	chr8	1101594	1101741	147	single	Variation_41440	chr8	1101422	1101674	252	Wang et al. (2008)	Illumina DNA sequencing	
2900	chr8	1172283	1178359	6076	26	chr8	1172252	1178475	6223	HiConf	Variation_47442	chr8	1172925	1173337	412	Bentley et al. (2008)	Illumina DNA sequencing	
2901	chr8	1184337	1185966	1629	25	chr8	1184306	1185972	1666	single	Variation_43342	chr8	1184467	1186030	1563	Wang et al. (2008)	Illumina DNA sequencing	
2902	chr8	1281749	1284591	2842	31	chr8	1281718	1284658	2940	HiConf								
2903	chr8	1323203	1333346	10143	49	chr8	1323172	1333560	10388	HiConf	Variation_32974	chr8	1327116	1342795	15679	Perry et al. (2008)	Agilent Custom CGH Arrays	
2904	chr8	1388226	1405817	17591	11	chr8	1388195	1406178	17983	single	Variation_32975	chr8	1395072	1412598	17526	Perry et al. (2008)	Agilent Custom CGH Arrays	
2905	chr8	1446781	1447320	539	37	chr8	1446750	1447338	588	HiConf	Variation_2112	chr8	1362046	1549356	187310	Locke et al. (2006)	BAC Array CGH	
2906	chr8	1509109	1513948	4839	8	chr8	1509078	1514076	4998	single	Variation_2112	chr8	1362046	1549356	187310	Locke et al. (2006)	BAC Array CGH	
2907	chr8	1734282	1740060	5778	23	chr8	1734282	1740162	5880	single								
2908	chr8	1794485	1795024	539	17	chr8	1794454	1795042	588	single								
2909	chr8	1823052	1823259	207	42	chr8	1823021	1823266	245	single								y
2910	chr8	1823444	1826692	3248	50	chr8	1823413	1826745	3332	HiConf	Variation_43362	chr8	1824042	1825719	1677	Wang et al. (2008)	Illumina DNA sequencing	
2911	chr8	1890476	1891064	588	26	chr8	1890445	1891082	637	HiConf								

locus_id	chrom	start	end	length	Yoruba w/ event	putative chr	putative start	putative end	putative len	putative type	variation_id	DGV_chr	DGV_start	DGV_end	DGV_len	Reference	Method/platform	complex
2912	chr8	2117248	2120335	3087	25	chr8	2117217	2120402	3185	single	Variation_37894	chr8	2116952	2120782	3830	McCarroll et al. (2008)	Affymetrix Human SNP Array 6.0	
2913	chr8	2174676	2197487	22811	21	chr8	2174645	2198018	23373	single	Variation_36800	chr8	2171813	2192861	21048	Kidd et al. (2008)	Paired End Mapping	
2914	chr8	2202900	2203537	637	35	chr8	2202869	2203555	686	HiConf	Variation_33450	chr8	2203034	2203659	625	Perry et al. (2008)	Agilent Custom CGH Arrays	
2915	chr8	2241317	2250969	9652	65	chr8	2234033	2258190	24157	HiConf	Variation_31417	chr8	2241488	2251039	9551	Perry et al. (2008)	Agilent Custom CGH Arrays	
2916	chr8	2317315	2324861	7546	18	chr8	2317284	2325026	7742	single	Variation_0732	chr8	2215497	2435332	219835	Sharp et al. (2005)	BAC Array CGH	
100498						chr8	2344773	2418102	73329	single								
2917	chr8	2685011	2696918	11907	1	chr8	2684980	2697132	12152	single	Variation_2725	chr8	2684771	2697799	13028	Redon et al. (2006)	Affymetrix 500K EA SNP Mapping Array	
2918	chr8	3288593	3305841	17248	24	chr8	3288562	3306202	17640	HiConf								
2919	chr8	3464258	3464658	400	29	chr8	3464227	3464668	441	single	Variation_10224	chr8	3448656	3465484	16828	Wang et al. (2007)	Illumina HumanHap550 BeadChip	
2920	chr8	3773693	3777659	3966	16	chr8	3773662	3777729	4067	single	Variation_10225	chr8	3774231	3777675	3444	Wang et al. (2007)	Illumina HumanHap550 BeadChip	
2921	chr8	3982335	3991873	9538	11	chr8	3982304	3991957	9653	HiConf	Variation_1753	chr8	3987468	3992429	4961	McCarroll et al. (2005)	Null genotypes	y
2922	chr8	4012568	4021290	8722	29	chr8	4012537	4021455	8918	HiConf	Variation_10227	chr8	4013913	4020594	6681	Wang et al. (2007)	Illumina HumanHap550 BeadChip	
2923	chr8	4100082	4108216	8134	6	chr8	4100051	4108381	8330	single	Variation_5403	chr8	4040046	4114650	74604	Simon-Sanchez et al. (2007)	Illumina HumanHap300 BeadChip	
2924	chr8	4110395	4112591	2196	20	chr8	4110390	4112742	2352	HiConf	Variation_39518	chr8	4110292	4112380	2088	Wheeler et al. (2008)	Sequencing	
100499	chr8	4288630	4339202	50572	1	chr8	4286447	4340274	53827	single	Variation_9076	chr8	4290322	4338060	47738	Pinto et al. (2007)	Affymetrix 500K SNP Mapping Array	
2926	chr8	4497873	4501499	3626	2	chr8	4497833	4501557	3724	HiConf	Variation_5404	chr8	4446054	4629092	183038	Simon-Sanchez et al. (2007)	Illumina HumanHap300 BeadChip	
100500	chr8	4597040	4740316	143276	6	chr8	4597009	4743250	146241	HiConf	Variation_9077	chr8	4605186	4706329	101143	Pinto et al. (2007)	Affymetrix 500K SNP Mapping Array	
2928	chr8	6050086	6056799	6713	5	chr8	6050055	6056915	6860	HiConf	Variation_32992	chr8	6050630	6056619	5989	Perry et al. (2008)	Agilent Custom CGH Arrays	
2929	chr8	6165109	6189599	24490	3	chr8	6165107	6190293	25186	HiConf	Variation_38722	chr8	6165713	6187827	22114	McCarroll et al. (2008)	Affymetrix Human SNP Array 6.0	
2930	chr8	6217568	6218989	1421	16	chr8	6217537	6219007	1470	HiConf	Variation_2730	chr8	6160762	6236366	75604	Redon et al. (2006)	Affymetrix 500K EA SNP Mapping Array	
100501	chr8	6498877	6556753	57876	24	chr8	6498846	6557867	59021	HiConf	Variation_5250	chr8	6349797	6769003	419206	Simon-Sanchez et al. (2007)	Illumina HumanHap300 BeadChip	
100502	chr8	6810246	6868313	58067	34	chr8	6810119	6869482	59363	HiConf	Variation_36853	chr8	6813245	6869017	55772	Kidd et al. (2008)	Paired End Mapping	
2933	chr8	6951490	7043784	92294	3	chr8	6951459	7045686	94227	HiConf	Variation_37613	chr8	6928066	7041357	113291	Cooper et al. (2008)	Illumina Human 1M BeadChip	
2934	chr8	7141855	7173411	31556	12	chr8	7141824	7174066	32242	single	Variation_36861	chr8	7090203	7148441	58238	Kidd et al. (2008)	Paired End Mapping	
2935	chr8	7174214	7258230	84016	27	chr8	7174213	7259914	85701	HiConf	Variation_4580	chr8	7138613	7258467	119854	Wong et al. (2007)	BAC Array CGH	y
2936	chr8	7353192	7703640	350448	37	chr8	7353161	7710763	357602	HiConf	Variation_38958	chr8	7237790	7836261	598471	McCarroll et al. (2008)	Affymetrix Human SNP Array 6.0	
2937	chr8	7812567	7812774	207	26	chr8	7812536	7812781	245	single	Variation_37568	chr8	7683445	7869061	185616	Cooper et al. (2008)	Illumina Human 1M BeadChip	y
2938	chr8	7813351	7879893	66542	39	chr8	7813320	7881234	67914	HiConf	Variation_1766	chr8	7814659	7824363	9704	McCarroll et al. (2005)	Mendelian inconsistencies	y
2939	chr8	7881585	7972498	90913	39	chr8	7881577	7974285	92708	HiConf	Variation_0738	chr8	7873159	8024984	151825	Sharp et al. (2005)	BAC Array CGH	
2940	chr8	7979902	8134185	154283	9	chr8	7979871	8137161	157290	HiConf	Variation_0739	chr8	7917018	8067760	150742	Sharp et al. (2005)	BAC Array CGH	
2941	chr8	8283457	8298694	15237	3	chr8	8283426	8299008	15582	HiConf	Variation_7710	chr8	8284466	8286644	2178	de Smith et al. (2007)	Agilent 185k CGH Arrays/Agilent Custom CGH Arrays	
2942	chr8	8423597	8423759	162	58	chr8	8423566	8423762	196	HiConf	Variation_10916	chr8	8423147	8423904	757	Hinds et al. (2005)	Oligo arrays	
2943	chr8	8888166	8889440	1274	10	chr8	8888135	8889458	1323	single								
2945	chr8	9534868	9539229	4361	26	chr8	9534837	9539296	4459	HiConf	Variation_23010	chr8	9534942	9539723	4781	Korbel et al. (2007)	Paired End Mapping	
2946	chr8	10498747	10501246	2499	19	chr8	10498716	10501313	2597	single								
2947	chr8	10568915	10569454	539	14	chr8	10568884	10569472	588	HiConf								
2948	chr8	11282894	11284707	1813	51	chr8	11282863	11284725	1862	HiConf	Variation_44324	chr8	11282970	11284618	1648	Bentley et al. (2008)	Illumina DNA sequencing	y
2949	chr8	11530197	11535664	5467	43	chr8	11530166	11535703	5537	HiConf	Variation_44325	chr8	11529692	11532309	2617	Bentley et al. (2008)	Illumina DNA sequencing	
2950	chr8	11774707	11775981	1274	2	chr8	11774676	11775999	1323	single								
2951	chr8	11986485	12097271	110786	15	chr8	11986454	12199506	213052	HiConf	Variation_33002	chr8	12034973	12072625	37652	Perry et al. (2008)	Agilent Custom CGH Arrays	
2952	chr8	12241122	12261767	20645	18	chr8	12241058	12262177	21119	HiConf	Variation_38849	chr8	12260380	12286526	26146	McCarroll et al. (2008)	Affymetrix Human SNP Array 6.0	
2953	chr8	12276222	12612348	336126	15	chr8	12276191	12613213	337022	HiConf	Variation_8597	chr8	12264620	12630632	366012	Pinto et al. (2007)	Affymetrix 500K SNP Mapping Array	
100506	chr8	12826805	12857361	30556	1	chr8	12816955	12871517	54562	single								
2954	chr8	13624261	13694761	70500	40	chr8	13624230	13695868	71638	HiConf	Variation_9084	chr8	13631654	13678755	47101	Pinto et al. (2007)	Affymetrix 500K SNP Mapping Array	
2955	chr8	13702073	13740097	38024	24	chr8	13702042	13740850	38808	HiConf	Variation_38704	chr8	13720460	13740957	20497	McCarroll et al. (2008)	Affymetrix Human SNP Array 6.0	
2956	chr8	13947661	13950405	2744	4	chr8	13947630	13950472	2842	HiConf	Variation_29789	chr8	13939561	14017836	78275	Jakobsson et al. (2008)	Illumina HumanHap550 BeadChip	
2957	chr8	14305626	14318787	13161	2	chr8	14303027	14319099	16072	HiConf	Variation_33011	chr8	14305804	14318121	12317	Perry et al. (2008)	Agilent Custom CGH Arrays	
2958	chr8	14523594	14535171	11577	1	chr8	14519852	14535483	15631	single	Variation_36792	chr8	14525015	14565465	40450	Kidd et al. (2008)	Paired End Mapping	
2959	chr8	14551390	14559328	7938	5	chr8	14551359	14559493	8134	HiConf	Variation_2740	chr8	14549778	14559616	9838	Redon et al. (2006)	Affymetrix 500K EA SNP Mapping Array	
2960	chr8	14832985	14837305	4320	2	chr8	14831394	14837421	6027	HiConf	Variation_38708	chr8	14832796	14840174	7378	McCarroll et al. (2008)	Affymetrix Human SNP Array 6.0	
2961	chr8	14963529	14965881	2352	4	chr8	14963498	14965899	2401	single	Variation_1450	chr8	14900309	15109533	209224	Conrad et al. (2005)	Mendelian inconsistencies	
2962	chr8	15092546	15095714	3168	7	chr8	15092515	15096141	3626	HiConf	Variation_10245	chr8	15092801	15095529	2728	Wang et al. (2007)	Illumina HumanHap550 BeadChip	
2963	chr8	15186969	15188194	1225	13	chr8	15186938	15188212	1274	HiConf	Variation_1451	chr8	15117474	15206966	89492	Conrad et al. (2005)	Mendelian inconsistencies	
2964	chr8	15858318	15862042	3724	5	chr8	15858287	15862109	3822	HiConf	Variation_33014	chr8	15817728	15886737	69009	Perry et al. (2008)	Agilent Custom CGH Arrays	
2965	chr8	16245239	16252376	7137	45	chr8	16245142	16252541	7399	HiConf	Variation_22699	chr8	16245666	16252485	6819	Korbel et al. (2007)	Paired End Mapping	
2966	chr8	16306472	16322985	16513	17	chr8	16306441	16323297	16856	HiConf	Variation_33016	chr8	16306994	16323221	16227	Perry et al. (2008)	Agilent Custom CGH Arrays	
2967	chr8	16365321	16370809	5488	8	chr8	16365290	16370925	5635	HiConf	Variation_33018	chr8	16365707	16370012	4305	Perry et al. (2008)	Agilent Custom CGH Arrays	
2968	chr8	16866346	16866553	207	42	chr8	16866315	16866560	245	HiConf	Variation_13031	chr8	16866251	16866620	369	Mills et al. (2006)	Sequence trace read mapping	
2969	chr8	17354435	17359776	5341	1	chr8	17354404	17359892	5488	single	Variation_3724	chr8	17335551	17685810	350259	Redon et al. (2006)	BAC Array CGH	
2970	chr8	17481737	17490655	8918	1	chr8	17481706	17490820	9114	single	Variation_10248	chr8	17482709	17490705	7996	Wang et al. (2007)	Illumina HumanHap550 BeadChip	
2971	chr8	17795288	17796562	1274	53	chr8	17795257	17796580	1323	HiConf								
2972	chr8	17831156	17831269	113	1	chr8	17831125	17831272	147	single								
2973	chr8	18897053	18903521	6468	6	chr8	18897022	18903637	6615	HiConf	Variation_38711	chr8	18895819	18903101	7282	McCarroll et al. (2008)	Affymetrix Human SNP Array 6.0	y
2974	chr8	19059831	19082175	22344	19	chr8	19059800	19082634	22834	HiConf								
2975	chr8	19091436	19121841	30405	6	chr8	19091405	19122422	31017	HiConf								
2976	chr8	20827996	20828976	980	14	chr8	20827965	20828994	1029	single	Variation_44340	chr8	20827869	20829022	1153	Bentley et al. (2008)	Illumina DNA sequencing	y
100508	chr8	20942066	21041380	99314	24	chr8	20942013	21042438	100425	single	Variation_13036	chr8	20944957	20945760	803	Mills et al. (2006)	Sequence trace read mapping	
2977	chr8	21212499	21215635	3136	9	chr8	21212468	21215702	3234	single	Variation_31434	chr8	21212588	21215588	3000	Perry et al. (2008)	Agilent Custom CGH Arrays	
2978	chr8	21374640	21380471	5831	5	chr8	21374609	21380587	5978	HiConf	Variation_33025	chr8	21374667	21380535	5868	Perry et al. (2008)	Agilent Custom CGH Arrays	y
2979	chr8	21974008	21974939	931	17	chr8	21973977	21974957	980	single	Variation_4586	chr8	21944545	22097871	153326	Wong et al. (2007)	BAC Array CGH	
2980						chr8	22611222	22619405	8183	HiConf								
2981	chr8	22735601	22740221	4620	5	chr8	22734800	22740337	5537	single	Variation_2746	chr8	21729708	22858325	1128617	Redon et al. (2006)	Affymetrix 500K EA SNP Mapping Array	
2982	chr8	22822884	22828372	5488	3	chr8	22822853	22828488	5635	single	Variation_2746	chr8	21729708	22858325	1128617	Redon et al. (2006)	Affymetrix 500K EA SNP Mapping Array	
2983	chr8	23532355	23542596	10241	4	chr8	23532324	23542810	10486	single								
2984	chr8	24145492	24154753	9261	7	chr8	24145461	24154918	9457	single	Variation_4588	chr8	24116701	24305391	188690	Wong et al. (2007)	BAC Array CGH	
2985	chr8	24200764	24206987	6223	32	chr8	24200733	24207103	6370	HiConf	Variation_6597	chr8	24201255	24207030	5775	Mills et al. (2006)	Sequence trace read mapping	
100509						chr8	24347537	24372405	24868	single								
2986	chr8	24498243	24502653	4410	15	chr8	24498212	24502720	4508	HiConf								
2987	chr8	24649800	24651368	1568	11	chr8	24649769	24651386	1617	HiConf	Variation_44341	chr8	24649436	24651485	2049	Bentley et al. (2008)	Illumina DNA sequencing	
2988	chr8	24998925	25001130	2205	3	chr8	24998894	25001148	2254	single								
2989	chr8	25028325	25047288	18963	27	chr8	25028294	25047649	19355	HiConf	Variation_44342	chr8	25028349	25046860	18511	Bentley et al. (2008)	Illumina DNA sequencing	y
2990	chr8	25122633	25126832	4199	46	chr8	25122619	25126833	4214	HiConf	Variation_43561	chr8	25122559	25126702	4143	Wang et al. (2008)	Illumina DNA sequencing	
2991	chr8	25175423	25180176	4753	9	chr8	25175392	25180243	4851	HiConf								
2992	chr8	25466679	25472510	5831	5	chr8	25466648	25472626	5978	HiConf	Variation_43594	chr8	25466564	25472644	6080	Wang et al. (2008)	Illumina DNA sequencing	
2993	chr8	26977545	26978770	1225	7	chr8	26977514	26978788	1274	single								
2994	chr8	27548591	27551335	2744	15	chr8	27548560	27551402	2842	single								

locus_id	chrom	start	end	length	Yoruba w/ event	putative chr	putative start	putative end	putative len	putative type	variation_id	DGV_chr	DGV_start	DGV_end	DGV_len	Reference	Method/platform	complex
100510	chr8	27677209	27757037	79828	2	chr8	27676940	27758648	81708	single								
2995	chr8	27828088	27832938	4850	17	chr8	27828056	27833054	4998	single								
2996	chr8	27927655	27930840	3185	4	chr8	27927624	27930907	3283	HiConf								
2997	chr8	28366891	28367773	882	8	chr8	28366860	28367791	931	single								
2998	chr8	28507668	28508844	1176	3	chr8	28507637	28508862	1225	single								
2999	chr8	28521731	28522809	1078	4	chr8	28521700	28522827	1127	single								y
3000	chr8	29957235	29957635	400	55	chr8	29957204	29957645	441	HiConf	Variation_13045	chr8	29957081	29957827	746	Mills et al. (2006)	Sequence trace read mapping	
3001	chr8	30596832	30602369	5537	6	chr8	30596801	30602485	5684	HiConf								
3002	chr8	30724330	30725016	686	46	chr8	30724299	30725034	735	HiConf								
3003	chr8	31197180	31197866	686	4	chr8	31197149	31197884	735	single								y
3004	chr8	32799432	32811410	11978	75	chr8	32799302	32811503	12201	HiConf	Variation_22849	chr8	32799340	32811101	11761	Korbel et al. (2007)	Paired End Mapping	
3005	chr8	33208385	33217771	9386	1	chr8	33208354	33220751	12397	single								
3006	chr8	34142178	34142286	108	73	chr8	34142147	34142294	147	HiConf								
3007	chr8	35175735	35178724	2989	3	chr8	35175704	35178791	3087	HiConf								
3008	chr8	35479339	35492434	13095	18	chr8	35479308	35492783	13475	HiConf								y
3009	chr8	36210811	36211211	400	36	chr8	36210780	36211221	441	HiConf								
3010	chr8	36489082	36490258	1176	66	chr8	36489051	36490276	1225	HiConf								
3011	chr8	37473884	37491833	17949	11	chr8	37473853	37492130	18277	single	Variation_36812	chr8	37483934	37518440	34506	Kidd et al. (2008)	Paired End Mapping	
3012	chr8	37508821	37515001	6180	9	chr8	37508790	37515062	6272	HiConf	Variation_22601	chr8	37509098	37514976	5878	Korbel et al. (2007)	Paired End Mapping	
3013						chr8	37792696	37799164	6468	single								
3014	chr8	38845606	38848726	3120	2	chr8	38842815	38848842	6027	single								
3015	chr8	39351466	39463137	111671	13	chr8	39351435	39465409	113974	HiConf	Variation_1462	chr8	39360327	39492651	132324	Conrad et al. (2005)	Mendelian inconsistencies	
3016	chr8	39469654	39505443	35789	12	chr8	39469623	39506324	36701	HiConf	Variation_1464	chr8	39488280	39492651	4371	Conrad et al. (2005)	Mendelian inconsistencies	
3017	chr8	39506427	39513901	7474	14	chr8	39506422	39514066	7644	single	Variation_7684	chr8	39507846	39563631	55785	de Smith et al. (2007)	Agilent 185k CGH Arrays/Agilent Custom CGH Arrays	
3018	chr8	39535314	39537568	2254	16	chr8	39535283	39537586	2303	HiConf	Variation_7684	chr8	39507846	39563631	55785	de Smith et al. (2007)	Agilent 185k CGH Arrays/Agilent Custom CGH Arrays	
3019	chr8	40302182	40309024	6842	8	chr8	40302182	40309140	6958	HiConf	Variation_33032	chr8	40302061	40308873	6812	Perry et al. (2008)	Agilent Custom CGH Arrays	
3020	chr8	40415550	40429466	13916	4	chr8	40415519	40429729	14210	HiConf								
3021	chr8	40588961	40589411	450	16	chr8	40588930	40589420	490	HiConf	Variation_7686	chr8	40588917	40590960	2043	de Smith et al. (2007)	Agilent 185k CGH Arrays/Agilent Custom CGH Arrays	
3022	chr8	40835431	40845427	9996	4	chr8	40835400	40845641	10241	single								
3023	chr8	40893986	40899327	5341	48	chr8	40893955	40899443	5488	HiConf	Variation_23044	chr8	40893619	40899501	5882	Korbel et al. (2007)	Paired End Mapping	
3024	chr8	41723066	41727770	4704	6	chr8	41723035	41727837	4802	single								
3025	chr8	42309302	42313720	4418	29	chr8	42309271	42313730	4459	HiConf	Variation_23873	chr8	42309596	42313559	3963	Levy et al. (2007)	Sequencing	
3026	chr8	42872606	42907151	34545	7	chr8	42872575	42907855	35280	single								
3027	chr8	43287832	43294351	6519	5	chr8	43287801	43294465	6664	HiConf	Variation_29791	chr8	43206085	43405535	199450	Jakobsson et al. (2008)	Illumina HumanHap550 BeadChip	
3028						chr8	43504479	43518836	14357	single								
3029	chr8	43727166	43781556	54390	17	chr8	43727135	43782652	55517	HiConf	Variation_43694	chr8	43678430	43739342	60912	Wang et al. (2008)	Illumina DNA sequencing	
3030	chr8	47044074	47051571	7497	2	chr8	47044043	47051736	7693	single								
3031	chr8	47149847	47162996	13149	3	chr8	47119944	47163848	43904	HiConf								
3032	chr8	47242818	47249874	7056	2	chr8	47242787	47249990	7203	single	Variation_29793	chr8	47224323	47287319	62996	Jakobsson et al. (2008)	Illumina HumanHap550 BeadChip	
3033	chr8	47402607	47417797	15190	23	chr8	47402576	47418109	15533	single								
3034	chr8	47449483	47477431	27948	15	chr8	47449420	47478036	28616	HiConf								
3035						chr8	48729692	48775556	45864	single								
3036	chr8	49064540	49078946	14406	5	chr8	49064509	49079258	14749	HiConf	Variation_3730	chr8	48950846	49170567	219721	Redon et al. (2006)	BAC Array CGH	
3037	chr8	49554051	49556010	1959	15	chr8	49554019	49556028	2009	single								
3038	chr8	50691570	50696142	4572	10	chr8	50689888	50696258	6370	HiConf	Variation_44348	chr8	50691565	50697122	5557	Bentley et al. (2008)	Illumina DNA sequencing	
100513	chr8	51034193	51125823	91630	1	chr8	51034162	51127679	93517	single	Variation_9093	chr8	51035800	51127991	92191	Pinto et al. (2007)	Affymetrix 500K SNP Mapping Array	
3040	chr8	51193389	51200731	7342	13	chr8	51193118	51200909	7791	HiConf	Variation_37463	chr8	51193494	51200884	7390	Cooper et al. (2008)	Illumina Human 1M BeadChip	
3041	chr8	51387499	51390864	3365	15	chr8	51387452	51390931	3479	HiConf	Variation_44350	chr8	51387525	51390863	3338	Bentley et al. (2008)	Illumina DNA sequencing	
3042	chr8	52186397	52186731	334	45	chr8	52186397	52186740	343	HiConf	Variation_13055	chr8	52186317	52186590	273	Mills et al. (2006)	Sequence trace read mapping	
3043	chr8	52472588	52475724	3136	10	chr8	52472557	52475791	3234	single								
3044	chr8	53863649	53864099	450	15	chr8	53863618	53864108	490	HiConf	Variation_47552	chr8	53863514	53864186	672	Bentley et al. (2008)	Illumina DNA sequencing	
3045	chr8	53902212	53903339	1127	6	chr8	53902181	53903357	1176	single	Variation_2754	chr8	53897210	54039563	142353	Redon et al. (2006)	Affymetrix 500K EA SNP Mapping Array	
3046	chr8	54118204	54153876	35672	5	chr8	54118173	54154580	36407	HiConf	Variation_38718	chr8	54122300	54154739	32439	McCarroll et al. (2008)	Affymetrix Human SNP Array 6.0	
3047	chr8	54285294	54286029	735	3	chr8	54285263	54286047	784	single								
3048						chr8	55033346	55033444	98	single								
3049	chr8	55167735	55184052	16317	6	chr8	55167704	55184364	16660	single	Variation_3735	chr8	54995543	55179518	183975	Redon et al. (2006)	BAC Array CGH	
3050	chr8	55371790	55374466	2676	38	chr8	55371789	55374533	2744	HiConf	Variation_44351	chr8	55371519	55373671	2152	Bentley et al. (2008)	Illumina DNA sequencing	
3051	chr8	55451788	55454679	2891	2	chr8	55451757	55454746	2989	HiConf								
3052	chr8	56541891	56551250	9359	1	chr8	56541860	56551415	9555	single								
3053	chr8	57151549	57161251	9702	2	chr8	57151518	57161465	9947	HiConf								
3054	chr8	57323735	57330497	6762	2	chr8	57323704	57330613	6909	HiConf								
100515	chr8	57512693	57526079	13386	3	chr8	57504882	57560619	55737	single								
3055	chr8	58126306	58132480	6174	3	chr8	58126275	58132596	6321	HiConf	Variation_6620	chr8	58126642	58131991	5349	Mills et al. (2006)	Sequence trace read mapping	
100516	chr8	58277471	58352637	75166	23	chr8	58277440	58354150	76710	HiConf	Variation_7694	chr8	58280866	58306288	25422	de Smith et al. (2007)	Agilent 185k CGH Arrays/Agilent Custom CGH Arrays	
3057	chr8	58519188	58531819	12631	10	chr8	58519157	58532044	12887	HiConf	Variation_44355	chr8	58519650	58531061	11411	Bentley et al. (2008)	Illumina DNA sequencing	
3058	chr8	59476942	59503255	26313	2	chr8	59476911	59503763	26852	single	Variation_2756	chr8	59467844	59504885	37041	Redon et al. (2006)	Affymetrix 500K EA SNP Mapping Array	
3059	chr8	59813866	59814944	1078	3	chr8	59813835	59814962	1127	single								y
3060	chr8	60298231	60298966	735	13	chr8	60298200	60298984	784	single	Variation_4595	chr8	60225525	60450830	225305	Wong et al. (2007)	BAC Array CGH	
3061	chr8	60614232	60617123	2891	7	chr8	60614201	60617190	2989	HiConf	Variation_36845	chr8	60602744	60646693	43949	Kidd et al. (2008)	Paired End Mapping	
3062	chr8	61159357	61170821	11464	5	chr8	61159326	61171037	11711	HiConf	Variation_37465	chr8	61159097	61170274	11177	Cooper et al. (2008)	Illumina Human 1M BeadChip	y
100517	chr8	62102338	62108757	6419	61	chr8	62070261	62179482	109221	single								
3063	chr8	62676103	62677867	1764	17	chr8	62676072	62677885	1813	HiConf								
3064	chr8	63197610	63203000	5390	4	chr8	63197579	63203116	5537	HiConf								
3065	chr8	63377832	63388171	10339	36	chr8	63377801	63388385	10584	HiConf	Variation_44356	chr8	63377896	63387831	9935	Bentley et al. (2008)	Illumina DNA sequencing	
3066	chr8	64122485	64128169	5684	6	chr8	64122454	64128285	5831	HiConf								
3067	chr8	64998370	65013844	15474	13	chr8	64998329	65014156	15827	HiConf	Variation_33044	chr8	65009303	65013074	3771	Perry et al. (2008)	Agilent Custom CGH Arrays	
3068	chr8	65388645	65390311	1666	14	chr8	65388614	65390329	1715	HiConf								
3069	chr8	65406383	65419007	12624	4	chr8	65406352	65419533	13181	HiConf	Variation_37467	chr8	65407279	65419521	12242	Cooper et al. (2008)	Illumina Human 1M BeadChip	
3070	chr8	65436518	65456216	19698	2	chr8	65436487	65456626	20139	single								
3071	chr8	65497327	65497687	360	36	chr8	65497296	65497688	392	HiConf								
3072	chr8	66254573	66257245	2672	67	chr8	66254542	66257286	2744	HiConf	Variation_4598	chr8	66151384	66325607	174223	Wong et al. (2007)	BAC Array CGH	
3073	chr8	67289747	67291756	2009	2	chr8	67289716	67291774	2058	single								
3074	chr8	68319727	68324235	4508	18	chr8	68319696	68324302	4606	HiConf								
3075	chr8	68708934	68716088	7154	19	chr8	68708903	68716204	7301	HiConf	Variation_33045	chr8	68709340	68716127	6787	Perry et al. (2008)	Agilent Custom CGH Arrays	
3076	chr8	69260625	69260832	207	24	chr8	69260594	69260839	245	single	Variation_47576	chr8	69260463	69260886	423	Bentley et al. (2008)	Illumina DNA sequencing	
3077	chr8	69347894	69350981	3087	17	chr8	69347863	69351048	3185	HiConf	Variation_36860	chr8	69332480	69362988	30508	Kidd et al. (2008)	Paired End Mapping	

locus_id	chrom	start	end	length	Yoruba w/ event	putative chr	putative start	putative end	putative len	putative type	variation_id	DGV_chr	DGV_start	DGV_end	DGV_len	Reference	Method/platform	complex
3078	chr8	70362390	70370622	8232	6	chr8	70362359	70370787	8428	HiConf								
3079	chr8	70496846	70497008	162	24	chr8	70496815	70497011	196	single								
3080	chr8	72377417	72380651	3234	11	chr8	72377386	72380718	3332	HiConf	Variation_7704	chr8	72377373	72380559	3186	de Smith et al. (2007)	Agilent 185k CGH Arrays/Agilent Custom CGH Arrays	
3081	chr8	73488296	73492902	4606	4	chr8	73488265	73492969	4704	single								
3082	chr8	74508917	74513477	4560	11	chr8	74508886	74513541	4655	HiConf								
3083	chr8	75067566	75070506	2940	5	chr8	75067535	75070573	3038	single								
3084	chr8	75470003	75492837	22834	29	chr8	75469972	75493296	23324	HiConf	Variation_37468	chr8	75477774	75492574	14800	Cooper et al. (2008)	Illumina Human 1M BeadChip	
3085	chr8	75523903	75529587	5684	62	chr8	75523872	75529703	5831	HiConf	Variation_44360	chr8	75525430	75529542	4112	Bentley et al. (2008)	Illumina DNA sequencing	
3086	chr8	76441526	76450589	9063	1	chr8	76441495	76451001	9506	single								
100519	chr8	77730986	77784151	53165	1	chr8	77730955	77785247	54292	single								
3088	chr8	77996002	77998207	2205	2	chr8	77995971	77998225	2254	HiConf								
3089	chr8	78287552	78288826	1274	8	chr8	78287521	78288844	1323	HiConf								
3090	chr8	78415883	78422302	6419	5	chr8	78415852	78422418	6566	HiConf								y
3091	chr8	78994230	79002269	8039	17	chr8	78994199	79002431	8232	HiConf	Variation_28992	chr8	78994491	78994594	103	Levy et al. (2007)	Sequencing	
3092	chr8	79235555	79236005	450	14	chr8	79235524	79236014	490	single								
3093	chr8	79708013	79710806	2793	6	chr8	79707982	79710873	2891	HiConf								
3094	chr8	79834776	79836981	2205	5	chr8	79834745	79836999	2254	single	Variation_3737	chr8	79784789	79950902	166113	Redon et al. (2006)	BAC Array CGH	
3095	chr8	80193554	80197964	4410	4	chr8	80193523	80198031	4508	single								
3096	chr8	80597314	80605874	8560	2	chr8	80597283	80606250	8967	HiConf								
3097	chr8	80805189	80805572	383	16	chr8	80805141	80805582	441	single								
3098	chr8	81414634	81414944	310	13	chr8	81414603	81414946	343	HiConf								
3099	chr8	82207650	82208630	980	21	chr8	82207619	82208648	1029	HiConf								
3100	chr8	82541095	82545162	4067	2	chr8	82541064	82545229	4165	HiConf	Variation_1793	chr8	82542508	82543678	1170	McCarroll et al. (2005)	Null genotypes	
3101	chr8	83136494	83143215	6721	37	chr8	83136463	83143617	7154	HiConf	Variation_22775	chr8	83136165	83144126	7961	Korbel et al. (2007)	Paired End Mapping	
3102	chr8	83165404	83167854	2450	6	chr8	83165373	83167921	2548	single								
3103	chr8	83359150	83360187	1037	24	chr8	83359119	83360197	1078	single	Variation_28994	chr8	83359696	83360306	610	Levy et al. (2007)	Sequencing	
3104	chr8	83940592	83951119	10527	2	chr8	83940504	83951333	10829	HiConf								
3105	chr8	85253147	85256332	3185	4	chr8	85253116	85256399	3283	HiConf	Variation_8608	chr8	84967900	86064700	1096800	Pinto et al. (2007)	Affymetrix 500K SNP Mapping Array	
3106	chr8	85423030	85431654	8624	14	chr8	85422999	85431819	8820	HiConf	Variation_37469	chr8	85423547	85431554	8007	Cooper et al. (2008)	Illumina Human 1M BeadChip	
3107	chr8	85687434	85690130	2696	9	chr8	85687403	85690196	2793	HiConf	Variation_8608	chr8	84967900	86064700	1096800	Pinto et al. (2007)	Affymetrix 500K SNP Mapping Array	
100520	chr8	86737798	86917626	179828	9	chr8	86737767	86921346	183579	HiConf	Variation_31448	chr8	86727125	86910509	183384	Perry et al. (2008)	Agilent Custom CGH Arrays	
3109	chr8	86927918	86942392	14474	4	chr8	86927887	86942685	14798	single	Variation_0121	chr8	86898422	86955420	56998	Iafrate et al. (2004)	BAC Array CGH	y
3110	chr8	87168998	87169831	833	61	chr8	87168967	87169849	882	HiConf	Variation_44361	chr8	87168832	87169936	1104	Bentley et al. (2008)	Illumina DNA sequencing	
3111	chr8	87236373	87249611	13238	10	chr8	87236342	87249719	13377	HiConf	Variation_28999	chr8	87243434	87243700	266	Levy et al. (2007)	Sequencing	
3112	chr8	87257443	87263029	5586	15	chr8	87257412	87263145	5733	HiConf	Variation_36889	chr8	87244856	87290168	45312	Kidd et al. (2008)	Paired End Mapping	
3113	chr8	88581496	88582109	613	11	chr8	88581490	88582127	637	HiConf	Variation_10806	chr8	88581402	88581768	366	Conrad et al. (2005)	Mendelian inconsistencies	
3114	chr8	88591958	88597397	5439	8	chr8	88591927	88597513	5586	HiConf	Variation_22797	chr8	88590541	88599067	8526	Korbel et al. (2007)	Paired End Mapping	
3115	chr8	89167512	89168884	1372	5	chr8	89167481	89168902	1421	single	Variation_3739	chr8	89032945	89202820	169875	Redon et al. (2006)	BAC Array CGH	
3116	chr8	89898739	89901336	2597	4	chr8	89898708	89901403	2695	single								
3117	chr8	89995955	89999008	3053	10	chr8	89995924	89999501	3577	HiConf	Variation_44362	chr8	89995882	89999033	3151	Bentley et al. (2008)	Illumina DNA sequencing	
3118	chr8	90399225	90406379	7154	2	chr8	90399194	90406495	7301	HiConf	Variation_33048	chr8	90399355	90406183	6828	Perry et al. (2008)	Agilent Custom CGH Arrays	
3119	chr8	90806464	90809894	3430	7	chr8	90806433	90809961	3528	HiConf								
3120	chr8	91270739	91271719	980	19	chr8	91270708	91271737	1029	HiConf	Variation_10263	chr8	91222484	91281324	58840	Wang et al. (2007)	Illumina HumanHap550 BeadChip	
3121	chr8	93450243	93462460	12217	2	chr8	93445279	93462821	17542	single								
3122	chr8	94141649	94146731	5082	21	chr8	94141618	94146861	5243	HiConf	Variation_43578	chr8	94141461	94146423	4962	Wang et al. (2008)	Illumina DNA sequencing	
3123	chr8	94306583	94312659	6076	8	chr8	94306552	94312775	6223	HiConf	Variation_36893	chr8	94299224	94315571	16347	Kidd et al. (2008)	Paired End Mapping	
3124	chr8	95542265	95544269	2004	2	chr8	95542234	95544733	2499	single								
3125	chr8	95627525	95630465	2940	14	chr8	95627494	95630532	3038	HiConf	Variation_1798	chr8	95627870	95640619	12749	McCarroll et al. (2005)	Null genotypes	
3126	chr8	95707738	95708571	833	8	chr8	95707707	95708589	882	HiConf								
3127	chr8	95716656	95716916	260	19	chr8	95716625	95716919	294	single	Variation_44364	chr8	95716062	95718223	2161	Bentley et al. (2008)	Illumina DNA sequencing	
3128	chr8	95800985	95801818	833	35	chr8	95800954	95801836	882	single								
3129	chr8	96032559	96041428	8869	1	chr8	96032528	96041593	9065	single	Variation_10264	chr8	96037014	96043549	6535	Wang et al. (2007)	Illumina HumanHap550 BeadChip	
3130	chr8	96041771	96043192	1421	3	chr8	96041740	96043210	1470	single	Variation_10264	chr8	96037014	96043549	6535	Wang et al. (2007)	Illumina HumanHap550 BeadChip	
3131	chr8	96043564	96068770	25206	1	chr8	96043504	96069278	25774	single								
3132	chr8	96072393	96074796	2403	1	chr8	96069425	96082802	13377	single								
3133	chr8	96118015	96123601	5586	1	chr8	96117984	96123717	5733	single								
3134	chr8	96148689	96151335	2646	11	chr8	96148658	96151402	2744	HiConf								
3135	chr8	96923918	96926564	2646	5	chr8	96923887	96926631	2744	HiConf	Variation_38729	chr8	96923541	96926233	2692	McCarroll et al. (2008)	Affymetrix Human SNP Array 6.0	
3136	chr8	96944523	96949790	5267	46	chr8	96944516	96949906	5390	HiConf	Variation_22610	chr8	96944202	96950175	5973	Korbel et al. (2007)	Paired End Mapping	
3137	chr8	97308715	97311361	2646	7	chr8	97308684	97311428	2744	single								
3138	chr8	97590073	97591200	1127	16	chr8	97590042	97591218	1176	HiConf								
3139	chr8	98431550	98432187	637	3	chr8	98431519	98432205	686	single								
3140	chr8	99426397	99428590	2193	12	chr8	99426366	99428963	2597	HiConf								
100522	chr8	100012903	100120897	107994	17	chr8	100012872	100121088	108216	single	Variation_28725	chr8	100110126	100110282	156	Levy et al. (2007)	Sequencing	
3141	chr8	100675652	100676926	1274	2	chr8	100675621	100676944	1323	HiConf	Variation_3744	chr8	100473327	100907800	434473	Redon et al. (2006)	BAC Array CGH	
100523	chr8	101481604	101531094	49490	11	chr8	101481573	101532117	50544	single								
3143	chr8	102051337	102066370	15033	2	chr8	102051296	102066682	15386	single	Variation_38696	chr8	102057367	102061693	4326	McCarroll et al. (2008)	Affymetrix Human SNP Array 6.0	
3144	chr8	102456949	102472580	15631	6	chr8	102456918	102472892	15974	single	Variation_2765	chr8	102462222	102471681	9459	Redon et al. (2006)	Affymetrix 500K EA SNP Mapping Array	
3145	chr8	102548628	102550637	2009	2	chr8	102548597	102550655	2058	single								
3146	chr8	102690238	102696412	6174	25	chr8	102690207	102696528	6321	HiConf	Variation_33049	chr8	102690530	102694979	4449	Perry et al. (2008)	Agilent Custom CGH Arrays	
3147	chr8	103123202	103130111	6909	9	chr8	103123171	103130227	7056	HiConf	Variation_10265	chr8	103123275	103125693	2418	Wang et al. (2007)	Illumina HumanHap550 BeadChip	
3148	chr8	103640152	103641720	1568	8	chr8	103640121	103641738	1617	single	Variation_7659	chr8	103427743	104137348	709605	de Smith et al. (2007)	Agilent 185k CGH Arrays/Agilent Custom CGH Arrays	
3149	chr8	104064100	104065668	1568	10	chr8	104064069	104065686	1617	HiConf	Variation_7659	chr8	103427743	104137348	709605	de Smith et al. (2007)	Agilent 185k CGH Arrays/Agilent Custom CGH Arrays	
3150	chr8	104828745	104831734	2989	2	chr8	104828714	104831801	3087	HiConf								
3151	chr8	104975745	104980050	4305	9	chr8	104975714	104981888	6174	HiConf								
3152	chr8	106600144	106618078	17934	11	chr8	106600113	106618439	18326	HiConf								
3153	chr8	106865773	106866410	637	52	chr8	106865742	106866428	686	HiConf								
3154	chr8	107303490	107304617	1127	14	chr8	107303459	107304635	1176	HiConf	Variation_44323	chr8	107303391	107305165	1774	Bentley et al. (2008)	Illumina DNA sequencing	
3155	chr8	107925496	107931180	5684	9	chr8	107925465	107931296	5831	HiConf	Variation_38697	chr8	107927695	107931446	3751	McCarroll et al. (2008)	Affymetrix Human SNP Array 6.0	
3156	chr8	108204502	108205629	1127	15	chr8	108204471	108205647	1176	single								
3157	chr8	108573668	108575677	2009	13	chr8	108573637	108575695	2058	HiConf	Variation_6653	chr8	108573995	108575539	1544	Mills et al. (2006)	Sequence trace read mapping	
3158	chr8	108648683	108679410	30727	20	chr8	108648558	108680016	31458	HiConf								
3159	chr8	109305287	109307394	2107	11	chr8	109305256	109307412	2156	HiConf								
3160	chr8	112009205	112012978	3773	7	chr8	112009174	112013045	3871	HiConf								
3161	chr8	112236810	112242502	5692	11	chr8	112236779	112242610	5831	HiConf	Variation_42495	chr8	112237350	112237843	493	Wang et al. (2008)	Illumina DNA sequencing	
3162	chr8	112363132	112368110	4978	58	chr8	112363101	112368393	5292	HiConf	Variation_22987	chr8	112363190	112366578	3388	Korbel et al. (2007)	Paired End Mapping	

locus_id	chrom	start	end	length	Yoruba w/ event	putative chr	putative start	putative end	putative len	putative type	variation_id	DGV_chr	DGV_start	DGV_end	DGV_len	Reference	Method/platform	complex
3163	chr8	113145174	113159285	14111	33	chr8	113145141	113159351	14210	HiConf	Variation_22846	chr8	113147243	113158579	11336	Korbel et al. (2007)	Paired End Mapping	
3164	chr8	114982152	114982389	237	14	chr8	114982151	114982396	245	single	Variation_11309	chr8	114982031	114982439	408	de Smith et al. (2007)	Agilent 185k CGH Arrays/Agilent Custom CGH Arrays	
3165	chr8	115003712	115003805	93	72	chr8	115003711	115003809	98	HiConf	Variation_13107	chr8	115003569	115003858	289	Mills et al. (2006)	Sequence trace read mapping	
3166	chr8	115148612	115149762	1150	18	chr8	115148604	115149780	1176	HiConf	Variation_39534	chr8	115148491	115149848	1357	Wheeler et al. (2008)	Sequencing	
100525	chr8	115220151	115279049	58898	4	chr8	115220120	115280267	60147	HiConf	Variation_38699	chr8	115222742	115279466	56724	McCarroll et al. (2008)	Affymetrix Human SNP Array 6.0	
3168	chr8	115703096	115712539	9443	23	chr8	115703088	115718817	15729	HiConf	Variation_7663	chr8	115703066	115712529	9463	de Smith et al. (2007)	Agilent 185k CGH Arrays/Agilent Custom CGH Arrays	
3169	chr8	115934644	115936163	1519	6	chr8	115934613	115936181	1568	HiConf	Variation_9098	chr8	115905670	116015087	109417	Pinto et al. (2007)	Affymetrix 500K SNP Mapping Array	
100526	chr8	116702989	116763749	60760	6	chr8	116702958	116764967	62009	HiConf								
3171	chr8	117311348	117312230	882	3	chr8	117311317	117312248	931	single								
3172	chr8	119315840	119318290	2450	3	chr8	119315809	119318357	2548	HiConf	Variation_33880	chr8	119316376	119317000	624	Perry et al. (2008)	Agilent Custom CGH Arrays	
3173	chr8	119757330	119764239	6909	2	chr8	119757299	119764355	7056	HiConf								
3174	chr8	120002428	120002590	162	17	chr8	120002397	120002593	196	HiConf	Variation_33881	chr8	120002481	120002653	172	Perry et al. (2008)	Agilent Custom CGH Arrays	
3175	chr8	120088570	120096662	8092	32	chr8	120088539	120096820	8281	HiConf	Variation_10270	chr8	120088774	120091667	2893	Wang et al. (2007)	Illumina HumanHap550 BeadChip	y
3176	chr8	120223235	120230376	7141	58	chr8	120220888	120230541	9653	HiConf	Variation_22724	chr8	120223528	120230562	7034	Korbel et al. (2007)	Paired End Mapping	
3177	chr8	120385608	120397025	11417	14	chr8	120385577	120397239	11662	HiConf								
3178	chr8	121722524	121725415	2891	6	chr8	121722493	121725482	2989	single								
3179	chr8	121941848	121943416	1568	6	chr8	121941817	121943434	1617	single	Variation_10800	chr8	121941910	121942526	616	Conrad et al. (2005)	Mendelian inconsistencies	
3180	chr8	121994180	122006528	12348	4	chr8	121994149	122006791	12642	HiConf								
3181	chr8	122388532	122410827	22295	3	chr8	122388501	122411286	22785	HiConf	Variation_37762	chr8	122390260	122408901	18641	McCarroll et al. (2008)	Affymetrix Human SNP Array 6.0	
3182	chr8	122469235	122498488	29253	3	chr8	122469204	122499094	29890	HiConf	Variation_37763	chr8	122474363	122496536	22173	McCarroll et al. (2008)	Affymetrix Human SNP Array 6.0	
3183	chr8	122727318	122733590	6272	11	chr8	122727287	122733706	6419	single								
3184	chr8	123128383	123130196	1813	19	chr8	123128352	123130214	1862	single	Variation_0980	chr8	123127800	123130521	2721	Hinds et al. (2005)	Oligo arrays	
3185	chr8	123130855	123131395	540	4	chr8	123130704	123131733	1029	single	Variation_10271	chr8	123129597	123131375	1778	Wang et al. (2007)	Illumina HumanHap550 BeadChip	
3186	chr8	123182724	123184205	1481	9	chr8	123182693	123184212	1519	HiConf	Variation_43364	chr8	123182622	123184310	1688	Wang et al. (2008)	Illumina DNA sequencing	
100528	chr8	123917359	123923814	6455	1	chr8	123909216	123934598	25382	single								
3187	chr8	126178584	126181769	3185	2	chr8	126178553	126181836	3283	HiConf								
3188	chr8	127261435	127263738	2303	66	chr8	127261404	127263756	2352	HiConf	Variation_44329	chr8	127261391	127263753	2362	Bentley et al. (2008)	Illumina DNA sequencing	
3189	chr8	127470420	127474242	3822	13	chr8	127470389	127474309	3920	HiConf								
3190	chr8	128081852	128081998	146	8	chr8	128081517	128082007	490	single								
3191	chr8	128316699	128318806	2107	21	chr8	128316668	128318824	2156	HiConf								
3192	chr8	128778671	128779071	400	12	chr8	128778640	128779081	441	single	Variation_8615	chr8	128714000	128977143	263143	Pinto et al. (2007)	Affymetrix 500K SNP Mapping Array	
3193	chr8	129831946	129835307	3361	52	chr8	129826015	129835472	9457	HiConf	Variation_36747	chr8	129834071	129841760	7689	Kidd et al. (2008)	Paired End Mapping	
3194	chr8	130209520	130214322	4802	34	chr8	130209489	130214438	4949	HiConf	Variation_37923	chr8	130211860	130213708	1848	McCarroll et al. (2008)	Affymetrix Human SNP Array 6.0	
3195	chr8	130504598	130512389	7791	6	chr8	130504567	130512554	7987	HiConf	Variation_36748	chr8	130490684	130515217	24533	Kidd et al. (2008)	Paired End Mapping	
100529	chr8	131935130	131941916	6786	2	chr8	131911725	131949430	37705	single								
3196	chr8	132160798	132162072	1274	8	chr8	132160767	132162090	1323	HiConf								y
3197	chr8	133129969	133130655	686	29	chr8	133129938	133130673	735	HiConf								
3198	chr8	133152166	133152426	260	3	chr8	133152135	133152429	294	single								
100530	chr8	134237080	134254617	17537	3	chr8	134237044	134254978	17934	single	Variation_2771	chr8	134236863	134249655	12792	Redon et al. (2006)	Affymetrix 500K EA SNP Mapping Array	
3200	chr8	134836394	134837767	1373	10	chr8	134836363	134837784	1421	HiConf	Variation_4610	chr8	134707520	134896260	188740	Wong et al. (2007)	BAC Array CGH	
3201	chr8	135545228	135548151	2923	21	chr8	135545197	135548186	2989	HiConf	Variation_44333	chr8	135546187	135547250	1063	Bentley et al. (2008)	Illumina DNA sequencing	
3202	chr8	136124114	136124374	260	4	chr8	136124083	136124377	294	single	Variation_0124	chr8	135975722	136154996	179274	Iafrate et al. (2004)	BAC Array CGH	
3203	chr8	136288145	136288656	511	16	chr8	136285048	136288723	3675	single								
3204	chr8	136691240	136696581	5341	4	chr8	136691209	136696697	5488	HiConf	Variation_38703	chr8	136692574	136694147	1573	McCarroll et al. (2008)	Affymetrix Human SNP Array 6.0	
3205	chr8	136902871	136904145	1274	2	chr8	136902840	136904163	1323	single								
3206	chr8	137030075	137030183	108	45	chr8	137030044	137030191	147	HiConf								y
3207	chr8	137229113	137233140	4027	73	chr8	137229082	137233149	4067	HiConf	Variation_39540	chr8	137229379	137233082	3703	Wheeler et al. (2008)	Sequencing	
3208	chr8	137763458	137774434	10976	13	chr8	137763427	137774648	11221	HiConf	Variation_33059	chr8	137765474	137774517	9043	Perry et al. (2008)	Agilent Custom CGH Arrays	
100531	chr8	137974942	138074363	99421	26	chr8	137974911	138076390	101479	HiConf	Variation_41553	chr8	138034881	138035171	290	Wang et al. (2008)	Illumina DNA sequencing	
3210	chr8	138215532	138218619	3087	3	chr8	138215501	138218686	3185	single								
3211	chr8	138812597	138812907	310	25	chr8	138812566	138812909	343	HiConf	Variation_13129	chr8	138812150	138813045	895	Mills et al. (2006)	Sequence trace read mapping	
3212	chr8	138870025	138871593	1568	16	chr8	138869994	138871611	1617	HiConf	Variation_44335	chr8	138870085	138871644	1559	Bentley et al. (2008)	Illumina DNA sequencing	
3213	chr8	138886352	138891644	5292	13	chr8	138886311	138891701	5390	HiConf	Variation_6673	chr8	138886322	138888624	2302	Mills et al. (2006)	Sequence trace read mapping	
3214	chr8	139050492	139053481	2989	3	chr8	139050461	139053548	3087	single	Variation_31453	chr8	139050666	139053464	2798	Perry et al. (2008)	Agilent Custom CGH Arrays	
3215	chr8	139287750	139288240	490	29	chr8	139287719	139288258	539	single								
3216	chr8	140033922	140034182	260	33	chr8	140033891	140034185	294	single								
3217	chr8	140327285	140328167	882	13	chr8	140327254	140328185	931	HiConf	Variation_2776	chr8	140327110	140328379	1269	Redon et al. (2006)	Affymetrix 500K EA SNP Mapping Array	
3218	chr8	140672735	140676606	3871	16	chr8	140672704	140676673	3969	HiConf	Variation_37764	chr8	140673518	140675508	1990	McCarroll et al. (2008)	Affymetrix Human SNP Array 6.0	
3219	chr8	140909831	140910630	799	4	chr8	140908296	140910648	2352	single								
3220	chr8	141031562	141036462	4900	4	chr8	141031531	141036578	5047	single								
3221	chr8	141274259	141275925	1666	14	chr8	141274228	141275943	1715	single								
3222	chr8	141414154	141416800	2646	3	chr8	141414123	141416867	2744	single								
3223	chr8	142072200	142074075	1875	6	chr8	142072193	142074104	1911	single	Variation_1806	chr8	142072079	142073375	1296	McCarroll et al. (2005)	Null genotypes	
3224	chr8	142709959	142725633	15674	30	chr8	142709928	142725853	15925	HiConf	Variation_10273	chr8	142716870	142718373	1503	Wang et al. (2007)	Illumina HumanHap550 BeadChip	
3225	chr8	143061592	143064572	2980	35	chr8	143061552	143064639	3087	HiConf	Variation_47487	chr8	143063000	143063309	309	Bentley et al. (2008)	Illumina DNA sequencing	
3226	chr8	143631747	143640452	8705	14	chr8	143631716	143644750	13034	HiConf	Variation_34338	chr8	143638570	143638755	185	Levy et al. (2007)	Sequencing	
3227	chr8	143812557	143819466	6909	12	chr8	143812526	143819582	7056	HiConf	Variation_4791	chr8	143739731	143909192	169461	Wong et al. (2007)	BAC Array CGH	
3228	chr8	143935106	143939908	4802	23	chr8	143935075	143939975	4900	HiConf								
3229	chr8	144258310	144279850	21540	2	chr8	144258279	144280182	21903	HiConf								
3230	chr8	145475764	145476164	400	48	chr8	145475733	145476174	441	HiConf	Variation_23291	chr8	145348220	145484561	136341	Levy et al. (2007)	Illumina HumanHap650Y BeadChip	
3231	chr8	145858503	145859924	1421	16	chr8	145858472	145859942	1470	single	Variation_4614	chr8	145749309	145882680	133371	Wong et al. (2007)	BAC Array CGH	y
3232	chr8	145887217	145887805	588	37	chr8	145887186	145887823	637	single	Variation_36793	chr8	145866720	145892921	26201	Kidd et al. (2008)	Paired End Mapping	
3233	chr8	146163847	146163934	87	53	chr8	146163840	146163938	98	HiConf	Variation_41140	chr8	146163672	146163978	306	Wheeler et al. (2008)	Sequencing	
3234	chr8	146188028	146209536	21508	10	chr8	146187997	146216319	28322	single	Variation_31459	chr8	146196231	146201474	5243	Perry et al. (2008)	Agilent Custom CGH Arrays	
3235	chr8	146226256	146265135	38879	4	chr8	146226168	146272620	46452	single	Variation_33464	chr8	146248616	146249251	635	Perry et al. (2008)	Agilent Custom CGH Arrays	y
100533	chr9	693	213670	212977	39	chr9	662	217683	217021	HiConf	Variation_8616	chr9	30910	307370	276460	Pinto et al. (2007)	Affymetrix 500K SNP Mapping Array	y
3238	chr9	382452	392154	9702	16	chr9	382421	392368	9947	single	Variation_8618	chr9	372810	723212	350402	Pinto et al. (2007)	Affymetrix 500K SNP Mapping Array	
100534	chr9	466192	540652	74460	43	chr9	465917	542161	76244	HiConf	Variation_36955	chr9	501466	533550	32084	Kidd et al. (2008)	Paired End Mapping	
3240	chr9	568603	569877	1274	7	chr9	568572	569895	1323	single	Variation_2781	chr9	554836	728434	173598	Redon et al. (2006)	Affymetrix 500K EA SNP Mapping Array	
3241	chr9	577472	598352	20880	9	chr9	577441	598658	21217	HiConf	Variation_1808	chr9	581040	598622	17582	McCarroll et al. (2005)	Null genotypes	
3242	chr9	599767	661311	61544	8	chr9	599736	662554	62818	HiConf	Variation_36957	chr9	606860	639030	32170	Kidd et al. (2008)	Paired End Mapping	
3243	chr9	662657	694045	31388	2	chr9	662652	694649	31997	HiConf	Variation_1809	chr9	665600	685607	20007	McCarroll et al. (2005)	Mendelian inconsistencies	
3244	chr9	789642	815064	25422	8	chr9	789611	815630	26019	HiConf	Variation_0126	chr9	615148	812246	197098	Iafrate et al. (2004)	BAC Array CGH	
3246	chr9	2031645	2061682	30037	3	chr9	2031614	2062288	30674	HiConf								
3247	chr9	2276547	2277919	1372	13	chr9	2276516	2277937	1421	single								
3248	chr9	3999942	4011669	11727	1	chr9	3999895	4011900	12005	single	Variation_2784	chr9	3998954	4012297	13343	Redon et al. (2006)	Affymetrix 500K EA SNP Mapping Array	

locus_id	chrom	start	end	length	Yoruba w/ event	putative chr	putative start	putative end	putative len	putative type	variation_id	DGV_chr	DGV_start	DGV_end	DGV_len	Reference	Method/platform	complex
3249	chr9	4366544	4367034	490	43	chr9	4366513	4367052	539	HiConf	Variation_44390	chr9	4366424	4367644	1220	Bentley et al. (2008)	Illumina DNA sequencing	
3250	chr9	4444503	4447054	2551	1	chr9	4444472	4448049	3577	single								
3251	chr9	4491151	4493944	2793	11	chr9	4491120	4494011	2891	HiConf	Variation_10283	chr9	4489426	4492575	3149	Wang et al. (2007)	Illumina HumanHap550 BeadChip	y
3252	chr9	5097293	5104631	7338	23	chr9	5097250	5104796	7546	single	Variation_22831	chr9	5095830	5105811	9981	Korbel et al. (2007)	Paired End Mapping	
100537	chr9	5375480	5393180	17700	4	chr9	5373169	5395391	22222	HiConf	Variation_38745	chr9	5375779	5388011	12232	McCarroll et al. (2008)	Affymetrix Human SNP Array 6.0	
3254	chr9	5740308	5741729	1421	9	chr9	5740277	5741747	1470	HiConf								
3255						chr9	6045596	6054220	8624	single								
3256	chr9	6390587	6391175	588	3	chr9	6390556	6391193	637	single								
3257	chr9	6662395	6664705	2310	5	chr9	6662261	6664760	2499	single	Variation_38746	chr9	6662095	6663766	1671	McCarroll et al. (2008)	Affymetrix Human SNP Array 6.0	
3258	chr9	7406210	7407484	1274	14	chr9	7406179	7407502	1323	HiConf	Variation_3758	chr9	7165387	7472982	307595	Redon et al. (2006)	BAC Array CGH	
3260	chr9	8105587	8109066	3479	5	chr9	8105556	8109133	3577	single	Variation_33070	chr9	7877756	8408897	531141	Perry et al. (2008)	Agilent Custom CGH Arrays	
3261	chr9	8271011	8276107	5096	3	chr9	8270980	8276223	5243	HiConf	Variation_33070	chr9	7877756	8408897	531141	Perry et al. (2008)	Agilent Custom CGH Arrays	
3262	chr9	8630916	8631602	686	79	chr9	8630885	8631620	735	HiConf								
3263	chr9	10394592	10395022	430	70	chr9	10394591	10395032	441	HiConf	Variation_13151	chr9	10394562	10395091	529	Mills et al. (2006)	Sequence trace read mapping	
3264	chr9	10437203	10441656	4453	15	chr9	10437172	10441729	4557	HiConf								
3265	chr9	11200966	11203024	2058	6	chr9	11200935	11203042	2107	single								
3266	chr9	11620406	11623640	3234	6	chr9	11620375	11623707	3332	HiConf	Variation_9115	chr9	11590500	11691727	101227	Pinto et al. (2007)	Affymetrix 500K SNP Mapping Array	
3267	chr9	11635400	11707056	71656	2	chr9	11635369	11708477	73108	HiConf	Variation_30317	chr9	11635495	11706862	71367	Jakobsson et al. (2008)	Illumina HumanHap550 BeadChip	
100540	chr9	11856856	12167761	310905	5	chr9	11856825	12174124	317299	HiConf	Variation_33073	chr9	11860284	12175002	314718	Perry et al. (2008)	Agilent Custom CGH Arrays	
100541	chr9	12276369	12571104	294735	1	chr9	12276338	12577125	300787	single	Variation_2798	chr9	12273944	12579068	305124	Redon et al. (2006)	Affymetrix 500K EA SNP Mapping Array	
3274	chr9	12635882	12639263	3381	2	chr9	12635851	12639330	3479	HiConf	Variation_3762	chr9	12611942	12774092	162150	Redon et al. (2006)	BAC Array CGH	
100542	chr9	12714377	12738709	24332	4	chr9	12714227	12739217	24990	single	Variation_10294	chr9	12716962	12738962	22000	Wang et al. (2007)	Illumina HumanHap550 BeadChip	
3276	chr9	13055812	13059683	3871	8	chr9	13055781	13059750	3969	HiConf	Variation_5261	chr9	12438789	13117738	678949	Simon-Sanchez et al. (2007)	Illumina HumanHap300 BeadChip	
100543	chr9	13239097	13281090	41993	1	chr9	13239066	13281916	42850	single	Variation_9120	chr9	13274600	13395072	120472	Pinto et al. (2007)	Affymetrix 500K SNP Mapping Array	
3278	chr9	14024150	14026110	1960	3	chr9	14024119	14026128	2009	single								
100544	chr9	14278289	14337653	59364	3	chr9	14278258	14338895	60637	single								
3279	chr9	14677173	14678398	1225	24	chr9	14677142	14678416	1274	HiConf	Variation_4615	chr9	14586657	14729902	143245	Wong et al. (2007)	BAC Array CGH	
3280	chr9	15147769	15148847	1078	12	chr9	15147738	15148865	1127	HiConf								
3281	chr9	15805278	15807060	1782	56	chr9	15805269	15814089	8820	HiConf	Variation_44382	chr9	15805275	15811942	6667	Bentley et al. (2008)	Illumina DNA sequencing	
3282	chr9	16060982	16064657	3675	2	chr9	16060951	16064724	3773	HiConf	Variation_38921	chr9	16061336	16064727	3391	McCarroll et al. (2008)	Affymetrix Human SNP Array 6.0	
3283	chr9	16825823	16828242	2419	2	chr9	16825792	16828585	2793	single								
100545	chr9	16888300	16928457	40157	2	chr9	16888120	16929011	40891	single	Variation_2801	chr9	16893850	16908196	14346	Redon et al. (2006)	Affymetrix 500K EA SNP Mapping Array	
3285	chr9	17391234	17397065	5831	7	chr9	17391203	17397181	5978	HiConf	Variation_5260	chr9	17386711	17588187	201476	Simon-Sanchez et al. (2007)	Illumina HumanHap300 BeadChip	
3286	chr9	17447829	17447991	162	1	chr9	17447798	17447994	196	single	Variation_5260	chr9	17386711	17588187	201476	Simon-Sanchez et al. (2007)	Illumina HumanHap300 BeadChip	
3287	chr9	17900019	17901618	1599	54	chr9	17900019	17901636	1617	HiConf	Variation_23898	chr9	17899518	17902883	3365	Levy et al. (2007)	Sequencing	
3288	chr9	18718644	18733246	14602	8	chr9	18718613	18733558	14945	single								
3289	chr9	19263573	19263883	310	19	chr9	19263542	19263885	343	single								
100546	chr9	19355742	19461778	106036	2	chr9	19355711	19463928	108217	single								
3290	chr9	20948879	20962060	13181	2	chr9	20948848	20962323	13475	single								
3291	chr9	21273847	21295799	21952	2	chr9	21273816	21296258	22442	HiConf	Variation_1818	chr9	21275389	21296318	20929	McCarroll et al. (2005)	Mendelian inconsistencies/Null genotypes	
100547	chr9	21935078	22019062	83984	5	chr9	21935047	22020748	85701	HiConf	Variation_33079	chr9	21822756	22026474	203718	Perry et al. (2008)	Agilent Custom CGH Arrays	
100548	chr9	22109101	22159816	50715	2	chr9	22109070	22160839	51769	single								
3293	chr9	22486819	22492477	5658	4	chr9	22486811	22492593	5782	HiConf	Variation_7739	chr9	22486611	22492632	6021	de Smith et al. (2007)	Agilent 185k CGH Arrays/Agilent Custom CGH Arrays	
3294	chr9	22643446	22643756	310	54	chr9	22643415	22643758	343	HiConf	Variation_42935	chr9	22643356	22643942	586	Wang et al. (2008)	Illumina DNA sequencing	
3295	chr9	22656529	22656736	207	45	chr9	22656498	22656743	245	single	Variation_47698	chr9	22656438	22656928	490	Bentley et al. (2008)	Illumina DNA sequencing	
3296	chr9	23349536	23367323	17787	16	chr9	23349505	23367684	18179	HiConf	Variation_22872	chr9	23351883	23367695	15812	Korbel et al. (2007)	Paired End Mapping	
100549						chr9	23557633	23627850	70217	single								
3297	chr9	23803962	23836792	32830	5	chr9	23803931	23837447	33516	single								
3298	chr9	23837630	23838997	1367	7	chr9	23837594	23839015	1421	single								
3299	chr9	23839340	23845563	6223	8	chr9	23839309	23845679	6370	single								
3300	chr9	24486483	24498243	11760	4	chr9	24486452	24498457	12005	HiConf	Variation_38739	chr9	24487680	24498238	10558	McCarroll et al. (2008)	Affymetrix Human SNP Array 6.0	
3301	chr9	24518039	24519950	1911	2	chr9	24518008	24519968	1960	single	Variation_2809	chr9	24457996	24518103	60107	Redon et al. (2006)	Affymetrix 500K EA SNP Mapping Array	
3302	chr9	24546145	24551212	5067	10	chr9	24546085	24551328	5243	HiConf								
3303	chr9	25683406	25683806	400	10	chr9	25683375	25683816	441	HiConf								
3304	chr9	25713002	25714717	1715	6	chr9	25712971	25714735	1764	HiConf								
100550	chr9	26170845	26228213	57368	2	chr9	26170754	26229358	58604	single								
3305	chr9	26450011	26450321	310	9	chr9	26449980	26450323	343	single	Variation_4616	chr9	26324440	26465736	141296	Wong et al. (2007)	BAC Array CGH	
3306	chr9	28037464	28043019	5555	9	chr9	28037433	28043117	5684	HiConf	Variation_37909	chr9	28037588	28040691	3103	McCarroll et al. (2008)	Affymetrix Human SNP Array 6.0	y
3307	chr9	28047460	28049910	2450	6	chr9	28047429	28049977	2548	HiConf								
3308	chr9	28067697	28069265	1568	48	chr9	28067666	28069283	1617	HiConf	Variation_44386	chr9	28067562	28069208	1646	Bentley et al. (2008)	Illumina DNA sequencing	
3309	chr9	28173733	28196247	22514	19	chr9	28173702	28196585	22883	HiConf	Variation_9125	chr9	28156400	28370167	213767	Pinto et al. (2007)	Affymetrix 500K SNP Mapping Array	
3310	chr9	28652463	28654864	2401	3	chr9	28652432	28654931	2499	HiConf	Variation_9127	chr9	28642515	28705838	63323	Pinto et al. (2007)	Affymetrix 500K SNP Mapping Array	
100551	chr9	28727556	28758377	30821	2	chr9	28727525	28759007	31482	single	Variation_9128	chr9	28642515	28751593	109078	Pinto et al. (2007)	Affymetrix 500K SNP Mapping Array	y
3312	chr9	28830578	28838369	7791	21	chr9	28830547	28838534	7987	HiConf	Variation_33083	chr9	28830605	28837661	7056	Perry et al. (2008)	Agilent Custom CGH Arrays	
3313	chr9	29082438	29088416	5978	73	chr9	29082407	29088532	6125	HiConf	Variation_43589	chr9	29082535	29088153	5618	Wang et al. (2008)	Illumina DNA sequencing	
100552	chr9	29145893	29208393	62500	9	chr9	29145862	29209709	63847	single	Variation_3765	chr9	29125948	29299828	173880	Redon et al. (2006)	BAC Array CGH	
3315	chr9	29249675	29250606	931	8	chr9	29249644	29250624	980	HiConf	Variation_3765	chr9	29125948	29299828	173880	Redon et al. (2006)	BAC Array CGH	
100553	chr9	30238027	30390511	152484	2	chr9	30237950	30391614	153664	HiConf	Variation_2813	chr9	30245385	30379493	134108	Redon et al. (2006)	Affymetrix 500K EA SNP Mapping Array	
3317	chr9	30898378	30898486	108	28	chr9	30898347	30898494	147	single	Variation_3768	chr9	30854032	31028881	174849	Redon et al. (2006)	BAC Array CGH	
3318	chr9	31281437	31282489	1052	69	chr9	31281429	31282507	1078	HiConf	Variation_39548	chr9	31281356	31282673	1317	Wheeler et al. (2008)	Sequencing	
3319	chr9	31970400	31972818	2418	3	chr9	31970369	31974289	3920	single								
100554	chr9	32501217	32597796	96579	2	chr9	32501186	32599774	98588	single								
3320	chr9	32706775	32718728	11953	3	chr9	32706692	32718942	12250	HiConf	Variation_37772	chr9	32706572	32717150	10578	McCarroll et al. (2008)	Affymetrix Human SNP Array 6.0	
3321	chr9	33004447	33013659	9212	2	chr9	33004416	33013824	9408	HiConf	Variation_31467	chr9	33005780	33018086	12306	Perry et al. (2008)	Agilent Custom CGH Arrays	
3322	chr9	33375181	33392772	17591	10	chr9	33375150	33393133	17983	HiConf	Variation_33088	chr9	33376561	33392111	15550	Perry et al. (2008)	Agilent Custom CGH Arrays	
3323	chr9	33646886	33648993	2107	11	chr9	33646855	33649011	2156	single	Variation_33090	chr9	33646996	33648420	1424	Perry et al. (2008)	Agilent Custom CGH Arrays	
3324	chr9	33928484	33934124	5640	5	chr9	33927968	33934240	6272	single								
3325	chr9	34043884	34046481	2597	2	chr9	34043853	34046548	2695	single	Variation_36949	chr9	34037775	34080440	42665	Kidd et al. (2008)	Paired End Mapping	
3326	chr9	34875414	34876149	735	3	chr9	34875383	34876167	784	single								
3327	chr9	34925198	34958518	33320	9	chr9	34925167	34959173	34006	single								
3328	chr9	35154518	35165702	11184	14	chr9	35154487	35166149	11662	HiConf								
3329	chr9	35658140	35661668	3528	3	chr9	35658109	35661735	3626	single								
100555	chr9	35890012	35926542	36530	15	chr9	35858421	35945764	87343	HiConf								
3331	chr9	35970760	35971945	1185	50	chr9	35970729	35971954	1225	HiConf	Variation_39549	chr9	35970787	35971992	1205	Wheeler et al. (2008)	Sequencing	y
3332	chr9	36353058	36353508	450	69	chr9	36353027	36353517	490	HiConf	Variation_11342	chr9	36353014	36353550	536	de Smith et al. (2007)	Agilent 185k CGH Arrays/Agilent Custom CGH Arrays	
3333	chr9	37467808	37470823	3015	5	chr9	37467777	37471158	3381	single								

locus_id	chrom	start	end	length	Yoruba w/ event	putative chr	putative start	putative end	putative len	putative type	variation_id	DGV_chr	DGV_start	DGV_end	DGV_len	Reference	Method/platform	complex
3334	chr9	38478580	38488037	9457	9	chr9	38478549	38488202	9653	HiConf	Variation_44389	chr9	38479355	38488345	8990	Bentley et al. (2008)	Illumina DNA sequencing	
3335	chr9	38702265	38744944	42679	8	chr9	38702234	38745795	43561	HiConf	Variation_3769	chr9	38557168	46588540	8031372	Redon et al. (2006)	BAC Array CGH	
3336	chr9	38857203	38864405	7202	30	chr9	38857172	38864473	7301	HiConf	Variation_36952	chr9	38837386	38865364	27978	Kidd et al. (2008)	Paired End Mapping	
3337	chr9	38925558	38927959	2401	17	chr9	38925527	38927977	2450	single	Variation_33093	chr9	38759754	38954213	194459	Perry et al. (2008)	Agilent Custom CGH Arrays	
3338	chr9	38928125	38952211	24086	19	chr9	38928124	38952722	24598	HiConf	Variation_33093	chr9	38759754	38954213	194459	Perry et al. (2008)	Agilent Custom CGH Arrays	
3339	chr9	38959760	39016415	56655	5	chr9	38959729	39016667	56938	single	Variation_33094	chr9	38966973	38975294	8321	Perry et al. (2008)	Agilent Custom CGH Arrays	
3340	chr9	39016815	39047715	30900	8	chr9	39016814	39048321	31507	single	Variation_33095	chr9	39017681	39048196	30515	Perry et al. (2008)	Agilent Custom CGH Arrays	
3341	chr9	39145078	39401985	256907	12	chr9	39145047	39407246	262199	HiConf	Variation_33096	chr9	39053784	39407326	353542	Perry et al. (2008)	Agilent Custom CGH Arrays	
3342	chr9	39423006	39436285	13279	18	chr9	39422975	39436548	13573	HiConf	Variation_31473	chr9	39433688	39435084	1396	Perry et al. (2008)	Agilent Custom CGH Arrays	y
3343	chr9	39577699	39578679	980	44	chr9	39577668	39578697	1029	single	Variation_38922	chr9	38906782	39964796	1058014	McCarroll et al. (2008)	Affymetrix Human SNP Array 6.0	y
3344	chr9	39770857	39790258	19401	27	chr9	39770826	39790622	19796	single	Variation_41166	chr9	39772180	39772364	184	Wheeler et al. (2008)	Sequencing	
3345	chr9	39802903	39831127	28224	13	chr9	39802872	39831684	28812	single	Variation_33097	chr9	39704132	39936565	232433	Perry et al. (2008)	Agilent Custom CGH Arrays	y
3346	chr9	39935987	39963231	27244	14	chr9	39935956	39963788	27832	HiConf	Variation_33097	chr9	39704132	39936565	232433	Perry et al. (2008)	Agilent Custom CGH Arrays	
3347	chr9	39964603	40126401	161798	9	chr9	39964572	40129702	165130	HiConf	Variation_4619	chr9	40014910	40222914	208004	Wong et al. (2007)	BAC Array CGH	
3348	chr9	40131448	40222422	90974	10	chr9	40131417	40273664	142247	HiConf	Variation_4619	chr9	40014910	40222914	208004	Wong et al. (2007)	BAC Array CGH	y
3349	chr9	40315982	40640607	324625	17	chr9	40315951	40647240	331289	HiConf	Variation_33098	chr9	40465835	40752041	286206	Perry et al. (2008)	Agilent Custom CGH Arrays	
3350	chr9	41041770	41044122	2352	16	chr9	41041739	41044140	2401	single	Variation_33100	chr9	40822700	41355793	533093	Perry et al. (2008)	Agilent Custom CGH Arrays	
3351	chr9	41088810	41093563	4753	16	chr9	41088779	41093630	4851	single	Variation_33100	chr9	40822700	41355793	533093	Perry et al. (2008)	Agilent Custom CGH Arrays	
3352	chr9	41569157	41578516	9359	18	chr9	41569126	41578681	9555	HiConf	Variation_2129	chr9	41414861	41579649	164788	Locke et al. (2006)	BAC Array CGH	
3353	chr9	41895742	41940332	44590	4	chr9	41895711	41941232	45521	single	Variation_31486	chr9	41779777	41897254	117477	Perry et al. (2008)	Agilent Custom CGH Arrays	
3354	chr9	42297890	42330225	32335	12	chr9	42297854	42330880	33026	single	Variation_33103	chr9	41990617	44671338	2680721	Perry et al. (2008)	Agilent Custom CGH Arrays	
3355	chr9	42692825	42739326	46501	7	chr9	42692794	42740275	47481	single	Variation_0302	chr9	42671740	43945910	1274170	Sebat et al. (2004)	ROMA	
3356	chr9	43029603	43044596	14993	1	chr9	43029522	43044908	15386	single	Variation_36953	chr9	43035536	43040615	5079	Kidd et al. (2008)	Paired End Mapping	
3357	chr9	43293075	43294349	1274	42	chr9	43293044	43294367	1323	HiConf	Variation_38097	chr9	43255666	43735571	479905	McCarroll et al. (2008)	Affymetrix Human SNP Array 6.0	
3358	chr9	43362704	43365987	3283	14	chr9	43362673	43366054	3381	HiConf	Variation_38097	chr9	43255666	43735571	479905	McCarroll et al. (2008)	Affymetrix Human SNP Array 6.0	
3359	chr9	43366495	43368731	2236	15	chr9	43366495	43368749	2254	single	Variation_38097	chr9	43255666	43735571	479905	McCarroll et al. (2008)	Affymetrix Human SNP Array 6.0	
3360	chr9	43369100	43512443	143343	8	chr9	43369092	43515357	146265	HiConf	Variation_4627	chr9	43400001	43485531	85530	Wong et al. (2007)	BAC Array CGH	
3361	chr9	43592759	43818728	225969	18	chr9	43592728	43823371	230643	HiConf	Variation_4622	chr9	43583844	43746434	162590	Wong et al. (2007)	BAC Array CGH	
3362	chr9	43828645	43959622	130977	8	chr9	43828614	43962286	133672	HiConf	Variation_4621	chr9	43662586	43856412	193826	Wong et al. (2007)	BAC Array CGH	
3363	chr9	44296647	44331728	35081	5	chr9	44294359	44332481	38122	single	Variation_33103	chr9	41990617	44671338	2680721	Perry et al. (2008)	Agilent Custom CGH Arrays	
3364	chr9	44395526	44403562	8036	8	chr9	44395495	44403727	8232	single	Variation_33103	chr9	41990617	44671338	2680721	Perry et al. (2008)	Agilent Custom CGH Arrays	
3365	chr9	44452023	44453199	1176	45	chr9	44451992	44453217	1225	HiConf	Variation_33103	chr9	41990617	44671338	2680721	Perry et al. (2008)	Agilent Custom CGH Arrays	
3366	chr9	44666741	44782047	115306	28	chr9	44666710	44782791	116081	HiConf	Variation_38121	chr9	44667855	44795733	127878	McCarroll et al. (2008)	Affymetrix Human SNP Array 6.0	
3367	chr9	44783018	44787526	4508	12	chr9	44782987	44787593	4606	single	Variation_5000	chr9	44772753	44801079	28326	Wong et al. (2007)	BAC Array CGH	
3368	chr9	44787894	44809868	21974	13	chr9	44787887	44810280	22393	HiConf	Variation_33106	chr9	44798753	44800062	1309	Perry et al. (2008)	Agilent Custom CGH Arrays	
3369	chr9	44935065	44976922	41857	15	chr9	44935034	44977762	42728	HiConf	Variation_4629	chr9	44967017	45024324	57307	Wong et al. (2007)	BAC Array CGH	y
3370	chr9	45042277	45186757	144480	23	chr9	45042246	45189736	147490	HiConf	Variation_33889	chr9	45052652	45053499	847	Perry et al. (2008)	Agilent Custom CGH Arrays	
3371	chr9	45242050	45313491	71441	6	chr9	45242019	45314931	72912	HiConf	Variation_4631	chr9	45280769	45456039	175270	Wong et al. (2007)	BAC Array CGH	
3372	chr9	45315795	45317265	1470	17	chr9	45315764	45317283	1519	single	Variation_33109	chr9	45314257	45333547	19290	Perry et al. (2008)	Agilent Custom CGH Arrays	
3373	chr9	45317902	45323488	5586	18	chr9	45317871	45323604	5733	single	Variation_33109	chr9	45314257	45333547	19290	Perry et al. (2008)	Agilent Custom CGH Arrays	
3374	chr9	45610144	45667223	57079	9	chr9	45610058	45668368	58310	HiConf	Variation_4638	chr9	45551008	45626173	75165	Wong et al. (2007)	BAC Array CGH	
3375	chr9	45984596	46044474	59878	15	chr9	45984565	46045668	61103	HiConf	Variation_31492	chr9	46003954	46023335	19381	Perry et al. (2008)	Agilent Custom CGH Arrays	y
3376	chr9	46157370	46170943	13573	13	chr9	46157339	46171206	13867	single	Variation_4636	chr9	46162950	46239508	76558	Wong et al. (2007)	BAC Array CGH	
3377	chr9	46241893	46275417	33524	13	chr9	46241815	46276066	34251	HiConf	Variation_31495	chr9	46260938	46280352	19414	Perry et al. (2008)	Agilent Custom CGH Arrays	
3378	chr9	46577300	46594401	17101	10	chr9	46577269	46594762	17493	single	Variation_31498	chr9	46575342	46577652	2310	Perry et al. (2008)	Agilent Custom CGH Arrays	
3379	chr9	65510358	65533078	22720	20	chr9	65510281	65533556	23275	single	Variation_3770	chr9	65207500	70343347	5135847	Redon et al. (2006)	BAC Array CGH	
3380	chr9	65597826	65604980	7154	30	chr9	65597795	65605096	7301	HiConf	Variation_3770	chr9	65207500	70343347	5135847	Redon et al. (2006)	BAC Array CGH	
3381	chr9	66017364	66028340	10976	3	chr9	66017333	66028554	11221	single	Variation_31500	chr9	66023265	66603163	579898	Perry et al. (2008)	Agilent Custom CGH Arrays	
3382	chr9	66256581	66273833	17252	8	chr9	66256502	66274191	17689	single	Variation_31500	chr9	66023265	66603163	579898	Perry et al. (2008)	Agilent Custom CGH Arrays	
3383	chr9	66542399	66600268	57869	9	chr9	66542368	66601462	59094	single	Variation_31500	chr9	66023265	66603163	579898	Perry et al. (2008)	Agilent Custom CGH Arrays	
3384	chr9	67055380	67274401	219021	26	chr9	67055349	67278985	223636	HiConf	Variation_33120	chr9	67106117	67249008	142891	Perry et al. (2008)	Agilent Custom CGH Arrays	
3385	chr9	67331005	67391295	60290	14	chr9	67330974	67392518	61544	single	Variation_31506	chr9	67362682	67383257	20575	Perry et al. (2008)	Agilent Custom CGH Arrays	
3386	chr9	68179146	68196532	17386	10	chr9	68179115	68197049	17934	single	Variation_38945	chr9	68143441	68227664	84223	McCarroll et al. (2008)	Affymetrix Human SNP Array 6.0	
3387	chr9	68197197	68198844	1647	9	chr9	68197196	68198862	1666	single	Variation_38945	chr9	68143441	68227664	84223	McCarroll et al. (2008)	Affymetrix Human SNP Array 6.0	
3388	chr9	68200216	68224961	24745	7	chr9	68200185	68225469	25284	single	Variation_38945	chr9	68143441	68227664	84223	McCarroll et al. (2008)	Affymetrix Human SNP Array 6.0	
3389	chr9	68505192	68548263	43071	6	chr9	68505161	68549114	43953	single	Variation_2144	chr9	67627819	68779114	1151295	Locke et al. (2006)	BAC Array CGH	
3390	chr9	68549361	68714226	164865	5	chr9	68549359	68717576	168217	HiConf	Variation_2144	chr9	67627819	68779114	1151295	Locke et al. (2006)	BAC Array CGH	
3391						chr9	68737911	68749279	11368	single								
3392	chr9	69466327	69473187	6860	15	chr9	69466296	69473303	7007	single	Variation_7748	chr9	68173261	70091682	1918421	de Smith et al. (2007)	Agilent 185k CGH Arrays/Agilent Custom CGH Arrays	
3393	chr9	70030562	70060170	29608	7	chr9	70030531	70060764	30233	single	Variation_31515	chr9	69667115	70105249	438134	Perry et al. (2008)	Agilent Custom CGH Arrays	
3394	chr9	70060912	70076573	15661	8	chr9	70060911	70076885	15974	single	Variation_31515	chr9	69667115	70105249	438134	Perry et al. (2008)	Agilent Custom CGH Arrays	
3395	chr9	70077553	70174083	96530	5	chr9	70077522	70176061	98539	HiConf	Variation_33132	chr9	70076830	70105249	28419	Perry et al. (2008)	Agilent Custom CGH Arrays	
3396	chr9	70927654	70933647	5993	49	chr9	70927623	70933650	6027	HiConf	Variation_22576	chr9	70927642	70933118	5476	Korbel et al. (2007)	Paired End Mapping	
3397	chr9	71224676	71238516	13840	9	chr9	71216527	71239116	22589	HiConf	Variation_37778	chr9	71225013	71238848	13835	McCarroll et al. (2008)	Affymetrix Human SNP Array 6.0	
100559	chr9	71288006	71325839	37833	4	chr9	71280448	71343486	63038	HiConf	Variation_8629	chr9	71289871	71324196	34325	Pinto et al. (2007)	Affymetrix 500K SNP Mapping Array	
3399	chr9	71952146	71954057	1911	15	chr9	71952115	71954075	1960	HiConf								
3400	chr9	72690919	72703855	12936	3	chr9	72690888	72704118	13230	HiConf	Variation_10301	chr9	72690677	72700700	10023	Wang et al. (2007)	Illumina HumanHap550 BeadChip	
3401	chr9	73095855	73100020	4165	2	chr9	73095824	73100087	4263	single								
3402	chr9	73134864	73139171	4307	5	chr9	73134828	73139238	4410	single								
3403	chr9	73445617	73450713	5096	14	chr9	73445586	73450829	5243	HiConf	Variation_22580	chr9	73445407	73451023	5616	Korbel et al. (2007)	Paired End Mapping	
3404	chr9	73871770	73874416	2646	6	chr9	73871739	73874483	2744	HiConf								
3405	chr9	74365560	74368140	2580	3	chr9	74365169	74368207	3038	single								y
100560	chr9	74594349	74663639	69290	20	chr9	74594318	74663800	69482	single								
3406	chr9	75251120	75252296	1176	5	chr9	75251089	75252314	1225	single								
3407	chr9	75257000	75268956	11956	3	chr9	75256969	75269170	12201	HiConf	Variation_38750	chr9	75257832	75268983	11151	McCarroll et al. (2008)	Affymetrix Human SNP Array 6.0	
3408	chr9	75934761	75936777	2016	10	chr9	75934688	75936795	2107	HiConf								
3409	chr9	75979603	75980436	833	18	chr9	75979572	75980454	882	HiConf								
3410	chr9	76709556	76737761	28205	20	chr9	76709525	76738729	29204	HiConf								
3411	chr9	78056517	78056625	108	7	chr9	78056486	78056633	147	single								
3412	chr9	78672790	78673329	539	7	chr9	78672759	78673347	588	single	Variation_33894	chr9	78672807	78673436	629	Perry et al. (2008)	Agilent Custom CGH Arrays	y
3413	chr9	78744918	78747864	2946	30	chr9	78744887	78747925	3038	HiConf	Variation_39555	chr9	78745579	78747921	2342	Wheeler et al. (2008)	Sequencing	
100561	chr9	80624632	80663128	38496	7	chr9	80624601	80684773	60172	HiConf	Variation_22911	chr9	80627200	80661390	34190	Korbel et al. (2007)	Paired End Mapping	
3415	chr9	82097498	82098919	1421	12	chr9	82097467	82098937	1470	HiConf								
3416	chr9	82171978	82174820	2842	14	chr9	82171947	82174887	2940	HiConf	Variation_37889	chr9	82172211	82174215	2004	McCarroll et al. (2008)	Affymetrix Human SNP Array 6.0	
3417	chr9	82261550	82261810	260	14	chr9	82261519	82261813	294	HiConf	Variation_42907	chr9	82261411	82261987	576	Wang et al. (2008)	Illumina DNA sequencing	
100562	chr9	83717291	83758843	41552	8	chr9	83717260	83759670	42410	HiConf	Variation_33138	chr9	83721636	83753825	32189	Perry et al. (2008)	Agilent Custom CGH Arrays	

locus_id	chrom	start	end	length	Yoruba w/ event	putative chr	putative start	putative end	putative len	putative type	variation_id	DGV_chr	DGV_start	DGV_end	DGV_len	Reference	Method/platform	complex
3419	chr9	83793241	83796622	3381	3	chr9	83793210	83796689	3479	single	Variation_33139	chr9	83793303	83800378	7075	Perry et al. (2008)	Agilent Custom CGH Arrays	
3420	chr9	84076492	84077000	508	6	chr9	84076479	84077018	539	single	Variation_33897	chr9	84076461	84077006	545	Perry et al. (2008)	Agilent Custom CGH Arrays	
3421	chr9	84562783	84573961	11178	4	chr9	84556434	84580738	24304	HiConf	Variation_38927	chr9	84566389	84574064	7675	McCarroll et al. (2008)	Affymetrix Human SNP Array 6.0	
100563	chr9	84851519	84914531	63012	1	chr9	84851439	84915800	64361	single	Variation_22674	chr9	84854348	84860978	6630	Korbel et al. (2007)	Paired End Mapping	
3422	chr9	86167781	86171653	3872	5	chr9	86167750	86172160	4410	single	Variation_33140	chr9	86015443	86208957	193514	Perry et al. (2008)	Agilent Custom CGH Arrays	
3423	chr9	86542582	86543072	490	22	chr9	86542551	86543090	539	HiConf	Variation_47743	chr9	86542431	86543149	718	Bentley et al. (2008)	Illumina DNA sequencing	y
3424	chr9	86965158	86965991	833	47	chr9	86965127	86966009	882	HiConf	Variation_29198	chr9	86965117	86966088	971	Levy et al. (2007)	Sequencing	
3425	chr9	87093538	87096674	3136	17	chr9	87093507	87096741	3234	single								
3426	chr9	87663261	87665949	2688	21	chr9	87663230	87667444	4214	HiConf								
100564	chr9	88079320	88161346	82026	1	chr9	88079289	88163006	83717	single								
3427	chr9	88344998	88345733	735	63	chr9	88344967	88345751	784	HiConf	Variation_44400	chr9	88344353	88345766	1413	Bentley et al. (2008)	Illumina DNA sequencing	
3428	chr9	88865819	88872679	6860	1	chr9	88865788	88872795	7007	single								
3429	chr9	89255761	89256121	360	68	chr9	89255730	89256122	392	HiConf	Variation_33899	chr9	89255908	89256805	897	Perry et al. (2008)	Agilent Custom CGH Arrays	
3430						chr9	89569869	89573054	3185	single								
3431	chr9	89703768	89725426	21658	13	chr9	89703737	89725885	22148	HiConf	Variation_36971	chr9	89700620	89720532	19912	Kidd et al. (2008)	Paired End Mapping	
3432	chr9	89934656	89945998	11342	14	chr9	89934625	89946042	11417	HiConf	Variation_23907	chr9	89939832	89943321	3489	Levy et al. (2007)	Sequencing	
3433	chr9	90098120	90099100	980	20	chr9	90098089	90099118	1029	HiConf								
3434	chr9	90728554	90728814	260	35	chr9	90728523	90728817	294	single								
3435	chr9	90863010	90865313	2303	46	chr9	90862979	90865331	2352	HiConf								
3436	chr9	91365818	91367761	1943	11	chr9	91365817	91367777	1960	HiConf	Variation_7752	chr9	91365597	91367649	2052	de Smith et al. (2007)	Agilent 185k CGH Arrays/Agilent Custom CGH Arrays	
3437	chr9	95007920	95012510	4590	3	chr9	95007889	95013034	5145	HiConf								y
3438	chr9	95510287	95511640	1353	28	chr9	95510286	95511658	1372	HiConf	Variation_43273	chr9	95510147	95511370	1223	Wang et al. (2008)	Illumina DNA sequencing	
3439	chr9	95540256	95541432	1176	58	chr9	95540225	95541450	1225	HiConf	Variation_43510	chr9	95539946	95542938	2992	Wang et al. (2008)	Illumina DNA sequencing	
100565	chr9	97075647	97153471	77824	7	chr9	97075616	97155045	79429	HiConf								
3440	chr9	97359283	97359490	207	25	chr9	97359252	97359497	245	single								
3441	chr9	98881027	98889014	7987	10	chr9	98880996	98889179	8183	single								
3442	chr9	100349067	100350880	1813	20	chr9	100349036	100350898	1862	HiConf	Variation_43499	chr9	100348790	100351626	2836	Wang et al. (2008)	Illumina DNA sequencing	
3443	chr9	101227000	101230773	3773	3	chr9	101226969	101230840	3871	HiConf								
100566	chr9	102852404	102896936	44532	7	chr9	102852373	102906763	54390	HiConf								
3445	chr9	103259691	103259923	232	60	chr9	103259685	103259930	245	HiConf	Variation_13190	chr9	103259521	103260203	682	Mills et al. (2006)	Sequence trace read mapping	
3446	chr9	103262852	103263489	637	8	chr9	103262821	103263507	686	single								
3447	chr9	103528628	103530735	2107	10	chr9	103528597	103530753	2156	single								
3448	chr9	105009114	105023961	14847	6	chr9	105009083	105024273	15190	HiConf	Variation_37769	chr9	105010731	105023627	12896	McCarroll et al. (2008)	Affymetrix Human SNP Array 6.0	
3449	chr9	105608237	105609609	1372	41	chr9	105608206	105609627	1421	HiConf	Variation_44367	chr9	105608070	105609570	1500	Bentley et al. (2008)	Illumina DNA sequencing	
3450	chr9	105757295	105760480	3185	10	chr9	105757264	105760547	3283	HiConf								
100567	chr9	105862393	105890448	28055	10	chr9	105844019	105898017	53998	single	Variation_10305	chr9	105853286	105890672	37386	Wang et al. (2007)	Illumina HumanHap550 BeadChip	
3452	chr9	106401057	106419530	18473	21	chr9	106401026	106419891	18865	HiConf	Variation_2829	chr9	106399623	106424215	24592	Redon et al. (2006)	Affymetrix 500K EA SNP Mapping Array	
100568	chr9	106526252	106580201	53949	7	chr9	106526221	106581273	55052	single								
3454	chr9	107412270	107413103	833	9	chr9	107412239	107413121	882	single								
3455	chr9	108000319	108011282	10963	5	chr9	108000288	108011411	11123	HiConf								
3456	chr9	108316418	108319554	3136	3	chr9	108316387	108319621	3234	single	Variation_10307	chr9	108224416	108331018	106602	Wang et al. (2007)	Illumina HumanHap550 BeadChip	
3457	chr9	108779370	108781820	2450	11	chr9	108779339	108781887	2548	HiConf								
3458	chr9	109467085	109471887	4802	20	chr9	109467054	109471954	4900	HiConf	Variation_44369	chr9	109467496	109471169	3673	Bentley et al. (2008)	Illumina DNA sequencing	
3459	chr9	109698169	109702383	4214	3	chr9	109698138	109702450	4312	single								
3460	chr9	110041610	110050332	8722	4	chr9	110041579	110050497	8918	single								
3461	chr9	111870143	111870453	310	20	chr9	111870112	111870455	343	HiConf	Variation_47623	chr9	111869976	111870748	772	Bentley et al. (2008)	Illumina DNA sequencing	
3462	chr9	112215740	112226422	10682	10	chr9	112215709	112226636	10927	HiConf	Variation_8630	chr9	111973092	112240266	267174	Pinto et al. (2007)	Affymetrix 500K SNP Mapping Array	y
3463	chr9	112516747	112519834	3087	64	chr9	112516716	112519901	3185	HiConf	Variation_22577	chr9	112516446	112521930	5484	Korbel et al. (2007)	Paired End Mapping	
3464	chr9	113064046	113075281	11235	5	chr9	113063997	113075512	11515	HiConf	Variation_38732	chr9	113064716	113071390	6674	McCarroll et al. (2008)	Affymetrix Human SNP Array 6.0	
3465	chr9	113207941	113209166	1225	41	chr9	113207910	113209184	1274	HiConf	Variation_44371	chr9	113205470	113209565	4095	Bentley et al. (2008)	Illumina DNA sequencing	
3466	chr9	114237039	114239734	2695	8	chr9	114237008	114239801	2793	HiConf								
3467	chr9	114314802	114327402	12600	15	chr9	114314771	114327609	12838	HiConf	Variation_29038	chr9	114316381	114316934	553	Levy et al. (2007)	Sequencing	
100569	chr9	114417972	114629228	211256	5	chr9	114417941	114633516	215575	HiConf	Variation_37770	chr9	114423076	114630399	207323	McCarroll et al. (2008)	Affymetrix Human SNP Array 6.0	y
100570	chr9	114845227	114902900	57673	25	chr9	114845196	114904094	58898	HiConf	Variation_36908	chr9	114850851	114896276	45425	Kidd et al. (2008)	Paired End Mapping	
3471	chr9	115191804	115194058	2254	20	chr9	115191773	115194076	2303	single								y
3472	chr9	115437637	115438225	588	35	chr9	115437606	115438243	637	HiConf	Variation_47633	chr9	115437499	115438339	840	Bentley et al. (2008)	Illumina DNA sequencing	
3473	chr9	115462627	115466743	4116	4	chr9	115462596	115466810	4214	single								
3474	chr9	116125352	116130644	5292	2	chr9	116125321	116130760	5439	single	Variation_7718	chr9	116118480	116130096	11616	de Smith et al. (2007)	Agilent 185k CGH Arrays/Agilent Custom CGH Arrays	
3475	chr9	117621567	117630877	9310	12	chr9	117621536	117631042	9506	HiConf								
3476	chr9	118553466	118555654	2188	35	chr9	118553418	118555672	2254	HiConf								
3477	chr9	118563298	118576430	13132	4	chr9	118563267	118576693	13426	HiConf	Variation_31526	chr9	118563219	118576012	12793	Perry et al. (2008)	Agilent Custom CGH Arrays	
100571	chr9	119196109	119219090	22981	4	chr9	119196078	119219573	23495	single								
3478	chr9	120574160	120577737	3577	8	chr9	120574129	120577804	3675	HiConf								
3479	chr9	120649228	120656382	7154	3	chr9	120649197	120656498	7301	single								
100572	chr9	121331254	121344826	13572	1	chr9	121330469	121349358	18889	single	Variation_44373	chr9	121331224	121344458	13234	Bentley et al. (2008)	Illumina DNA sequencing	
3481	chr9	121520840	121526034	5194	8	chr9	121520809	121526150	5341	single								
3482	chr9	121714733	121717526	2793	9	chr9	121714702	121717593	2891	single								
3483	chr9	122210711	122212139	1428	2	chr9	122210680	122212395	1715	HiConf								
3484	chr9	122300479	122304056	3577	10	chr9	122300448	122304123	3675	single	Variation_7723	chr9	122293522	122510910	217388	de Smith et al. (2007)	Agilent 185k CGH Arrays/Agilent Custom CGH Arrays	
3485	chr9	123262594	123270777	8183	2	chr9	123262563	123270942	8379	HiConf								
3486	chr9	125067656	125068685	1029	10	chr9	125067625	125068703	1078	single								
3487	chr9	126697788	126698048	260	20	chr9	126697757	126698051	294	single								
3488	chr9	126998452	127001686	3234	3	chr9	126998421	127001753	3332	single								
3489	chr9	127673623	127674505	882	3	chr9	127673592	127674523	931	HiConf								
3490	chr9	127819839	127822436	2597	6	chr9	127819808	127822503	2695	single								
3491	chr9	127937635	127947043	9408	2	chr9	127937604	127947208	9604	single	Variation_3781	chr9	127915778	128076779	161001	Redon et al. (2006)	BAC Array CGH	
3492	chr9	128011037	128018142	7105	21	chr9	128011006	128018258	7252	HiConf	Variation_33152	chr9	128011207	128012840	1633	Perry et al. (2008)	Agilent Custom CGH Arrays	
3493	chr9	128385348	128388092	2744	25	chr9	128385317	128388159	2842	single								
100573	chr9	128423593	128546058	122465	2	chr9	128423562	128548512	124950	HiConf	Variation_4654	chr9	128395518	128572086	176568	Wong et al. (2007)	BAC Array CGH	
3496						chr9	128552456	128563285	10829	single								
3497	chr9	128671949	128679005	7056	11	chr9	128671918	128679121	7203	single	Variation_4655	chr9	128518738	128682074	163336	Wong et al. (2007)	BAC Array CGH	
3498	chr9	129072524	129074883	2359	19	chr9	129072493	129074894	2401	HiConf								
3499	chr9	130450548	130454092	3544	37	chr9	130450177	130454097	3920	HiConf	Variation_23886	chr9	130451889	130453809	1920	Levy et al. (2007)	Sequencing	
3500	chr9	130493769	130498375	4606	15	chr9	130493738	130498442	4704	single								
3501						chr9	130955808	130970067	14259	HiConf								
3502	chr9	131566526	131569270	2744	10	chr9	131566495	131569337	2842	single	Variation_22622	chr9	131564643	131570728	6085	Korbel et al. (2007)	Paired End Mapping	

locus_id	chrom	start	end	length	Yoruba w/ event	putative chr	putative start	putative end	putative len	putative type	variation_id	DGV_chr	DGV_start	DGV_end	DGV_len	Reference	Method/platform	complex
3503	chr9	132678042	132683775	5733	6	chr9	132678011	132683891	5880	single								
3504	chr9	132740223	132742673	2450	17	chr9	132740192	132742740	2548	single	Variation_3782	chr9	132727387	133018382	290995	Redon et al. (2006)	BAC Array CGH	
3505	chr9	133380898	133381584	686	30	chr9	133380867	133381602	735	HiConf	Variation_1833	chr9	133372494	133438117	65623	McCarroll et al. (2005)	Null genotypes	y
3506	chr9	134071994	134072684	690	17	chr9	134071963	134072747	784	single								
3507	chr9	134897889	134904112	6223	2	chr9	134897858	134904228	6370	single								
3508	chr9	134934051	134951740	17689	4	chr9	134934020	134952101	18081	HiConf	Variation_37814	chr9	134933035	134947261	14226	McCarroll et al. (2008)	Affymetrix Human SNP Array 6.0	
3509	chr9	136635919	136636703	784	5	chr9	136635888	136636721	833	single								
3510	chr9	136688643	136688903	260	43	chr9	136688612	136688906	294	single								
3511	chr9	137064767	137068687	3920	23	chr9	137064736	137068754	4018	single								
3512	chr9	137150076	137151056	980	16	chr9	137150045	137151074	1029	single								
3513	chr9	137353987	137357746	3759	41	chr9	137353983	137357756	3773	HiConf	Variation_23893	chr9	137353887	137357427	3540	Levy et al. (2007)	Sequencing	
3514	chr9	137484158	137488225	4067	14	chr9	137484127	137488292	4165	single	Variation_34501	chr9	137256876	137520876	264000	Zogopoulos et al. (2007)	Affymetrix 500K and 100K SNP Mapping Arrays	
3515	chr9	137867828	137869445	1617	10	chr9	137867797	137869463	1666	HiConf								
3516	chr9	137869739	137870719	980	16	chr9	137869708	137870737	1029	single								y
3517	chr9	137879364	137884684	5320	4	chr9	137871619	137884947	13328	single								
3518						chr9	138105349	138110886	5537	single								
3519	chr9	138646800	138647418	618	46	chr9	138646799	138647436	637	HiConf	Variation_10315	chr9	138620707	138655535	34828	Wang et al. (2007)	Illumina HumanHap550 BeadChip	
3520	chr9	139116495	139118161	1666	50	chr9	139116464	139118179	1715	HiConf	Variation_13217	chr9	139116332	139117034	702	Mills et al. (2006)	Sequence trace read mapping	
3521	chr9	140040635	140050385	9750	3	chr9	140040604	140050845	10241	single	Variation_3787	chr9	140005384	140273252	267868	Redon et al. (2006)	BAC Array CGH	
3522	chr9	140095025	140110901	15876	11	chr9	140094994	140111213	16219	HiConf	Variation_36938	chr9	140091311	140113709	22398	Kidd et al. (2008)	Paired End Mapping	
100574	chr9	140135679	140242540	106861	3	chr9	140135591	140244738	109147	HiConf	Variation_31541	chr9	140155756	140250494	94738	Perry et al. (2008)	Agilent Custom CGH Arrays	y
5954	chrX	10199	108787	98588	47	chrX	10168	110814	100646	HiConf								
5955	chrX	116480	124148	7668	7	chrX	116449	124534	8085	single								
5956	chrX	317126	321349	4223	11	chrX	316271	321465	5194	HiConf	Variation_47804	chrX	321315	321468	153	Bentley et al. (2008)	Illumina DNA sequencing	
5957	chrX	364028	365302	1274	37	chrX	363997	365320	1323	single	Variation_4141	chrX	263529	482251	218722	Redon et al. (2006)	BAC Array CGH	
5958	chrX	382599	383285	686	33	chrX	382568	383303	735	single	Variation_4141	chrX	263529	482251	218722	Redon et al. (2006)	BAC Array CGH	y
5959	chrX	475258	481873	6615	26	chrX	475227	481989	6762	single	Variation_47815	chrX	480078	480310	232	Bentley et al. (2008)	Illumina DNA sequencing	
5960						chrX	530156	530205	49	single								
5961	chrX	586635	613879	27244	3	chrX	586604	614387	27783	HiConf	Variation_33901	chrX	605586	606091	505	Perry et al. (2008)	Agilent Custom CGH Arrays	
5962	chrX	742161	744906	2745	1	chrX	742130	745217	3087	single	Variation_34411	chrX	563862	966564	402702	Zogopoulos et al. (2007)	Affymetrix 500K and 100K SNP Mapping Arrays	
5963	chrX	830518	830664	146	39	chrX	830183	830673	490	HiConf	Variation_47833	chrX	830116	830862	746	Bentley et al. (2008)	Illumina DNA sequencing	
5964	chrX	1143569	1158906	15337	13	chrX	1143538	1159218	15680	HiConf								
5965	chrX	1670368	1680070	9702	2	chrX	1670337	1680284	9947	HiConf								
5966	chrX	1689919	1714814	24895	7	chrX	1689888	1715221	25333	HiConf								
5967	chrX	1753717	1754942	1225	43	chrX	1753686	1754960	1274	single								
5968	chrX	1870925	1874453	3528	28	chrX	1870894	1874520	3626	HiConf	Variation_33162	chrX	1870920	1874408	3488	Perry et al. (2008)	Agilent Custom CGH Arrays	
5969	chrX	2022542	2027970	5428	8	chrX	2022157	2028086	5929	single	Variation_4142	chrX	1781901	3204365	1422464	Redon et al. (2006)	BAC Array CGH	
5970	chrX	2377340	2410637	33297	13	chrX	2377309	2411021	33712	HiConf	Variation_4142	chrX	1781901	3204365	1422464	Redon et al. (2006)	BAC Array CGH	
5971	chrX	2526006	2534434	8428	7	chrX	2525975	2534599	8624	single	Variation_8292	chrX	2432308	2704240	271932	Pinto et al. (2007)	Affymetrix 500K SNP Mapping Array	
5972	chrX	2568881	2591080	22199	16	chrX	2568850	2591635	22785	HiConf	Variation_23329	chrX	2542769	2704239	161470	Levy et al. (2007)	Affymetrix 500K SNP Mapping Array	
5973	chrX	2756747	2758609	1862	14	chrX	2756716	2758627	1911	HiConf	Variation_8291	chrX	2713391	2813276	99885	Pinto et al. (2007)	Affymetrix 500K SNP Mapping Array	y
5974	chrX	2855090	2857442	2352	26	chrX	2855059	2857460	2401	HiConf	Variation_3260	chrX	2713391	3186492	473101	Redon et al. (2006)	Affymetrix 500K EA SNP Mapping Array	
5975	chrX	3273795	3274195	400	24	chrX	3273764	3274205	441	single								
5976	chrX	3714305	3727045	12740	1	chrX	3714274	3727308	13034	single	Variation_0820	chrX	3688954	3863129	174175	Sharp et al. (2005)	BAC Array CGH	
100980	chrX	3729798	3868350	138552	17	chrX	3729734	3871148	141414	HiConf	Variation_0820	chrX	3688954	3863129	174175	Sharp et al. (2005)	BAC Array CGH	
5978	chrX	3993066	4000710	7644	3	chrX	3993035	4000875	7840	HiConf								y
5979	chrX	4244044	4244828	784	51	chrX	4244013	4244846	833	HiConf	Variation_7786	chrX	4033374	4800687	767313	de Smith et al. (2007)	Agilent 185k CGH Arrays/Agilent Custom CGH Arrays	
5980	chrX	4246788	4261067	14279	3	chrX	4246757	4261359	14602	HiConf	Variation_1837	chrX	4248504	4256678	8174	McCarroll et al. (2005)	Null genotypes	
5981	chrX	4392318	4401786	9468	2	chrX	4392287	4402283	9996	single	Variation_7786	chrX	4033374	4800687	767313	de Smith et al. (2007)	Agilent 185k CGH Arrays/Agilent Custom CGH Arrays	
5982	chrX	4826213	4830182	3969	2	chrX	4826182	4830249	4067	single								y
5983	chrX	5065548	5067650	2102	62	chrX	5065547	5067654	2107	HiConf	Variation_23920	chrX	5065347	5067503	2156	Levy et al. (2007)	Sequencing	
5984	chrX	5372537	5372824	287	3	chrX	5372483	5372826	343	single	Variation_8907	chrX	5068980	5597394	528414	Pinto et al. (2007)	Affymetrix 500K SNP Mapping Array	
5985	chrX	6148429	6152643	4214	26	chrX	6148398	6152710	4312	HiConf	Variation_7794	chrX	6148253	6157490	9237	de Smith et al. (2007)	Agilent 185k CGH Arrays/Agilent Custom CGH Arrays	
5986	chrX	6347957	6359573	11616	2	chrX	6347926	6360029	12103	HiConf	Variation_5359	chrX	6330011	6624726	294715	Simon-Sanchez et al. (2007)	Illumina HumanHap300 BeadChip	
100982	chrX	6427117	6487583	60466	22	chrX	6427086	6488826	61740	HiConf	Variation_0655	chrX	6460342	6475413	15071	Tuzun et al. (2005)	Paired End Mapping	
5988	chrX	6668025	6672582	4557	1	chrX	6667994	6672649	4655	single	Variation_9336	chrX	6468166	6867230	399064	Wang et al. (2007)	Illumina HumanHap550 BeadChip	
100983	chrX	6929734	6976088	46354	3	chrX	6929703	6977013	47310	HiConf								
5989	chrX	7075852	7076538	686	11	chrX	7075821	7076556	735	single								
5990	chrX	7760725	7782089	21364	22	chrX	7760694	7782499	21805	HiConf	Variation_7798	chrX	7768949	7774040	5091	de Smith et al. (2007)	Agilent 185k CGH Arrays/Agilent Custom CGH Arrays	y
5991	chrX	8093435	8100295	6860	21	chrX	8093404	8100411	7007	HiConf	Variation_9337	chrX	7970400	8095053	124653	Wang et al. (2007)	Illumina HumanHap550 BeadChip	
5992	chrX	8131459	8139397	7938	1	chrX	8131428	8139562	8134	single	Variation_1840	chrX	8132307	8138688	6381	McCarroll et al. (2005)	Null genotypes	
100985	chrX	8368570	8423646	55076	8	chrX	8368539	8424767	56228	HiConf	Variation_8300	chrX	8326726	8838134	511408	Pinto et al. (2007)	Affymetrix 500K SNP Mapping Array	y
5994	chrX	8659483	8660267	784	8	chrX	8659452	8660285	833	single	Variation_8300	chrX	8326726	8838134	511408	Pinto et al. (2007)	Affymetrix 500K SNP Mapping Array	
5995	chrX	9332106	9343300	11194	19	chrX	9332075	9343492	11417	HiConf	Variation_31545	chrX	9329800	9341791	11991	Perry et al. (2008)	Agilent Custom CGH Arrays	
5996	chrX	9392670	9394336	1666	27	chrX	9392639	9394354	1715	HiConf	Variation_2275	chrX	9318753	9460420	141667	Locke et al. (2006)	BAC Array CGH	
100986						chrX	9822737	9877004	54267	single								
5997	chrX	9918587	9921380	2793	9	chrX	9918556	9921447	2891	HiConf								
5998	chrX	9942744	9943871	1127	30	chrX	9942713	9943889	1176	single								
5999	chrX	10490515	10491642	1127	46	chrX	10490484	10491660	1176	HiConf								
6000	chrX	11036669	11038482	1813	24	chrX	11036638	11038500	1862	HiConf								
6001	chrX	11592378	11593995	1617	12	chrX	11592347	11594013	1666	HiConf								
6002	chrX	11861731	11862956	1225	2	chrX	11861700	11862974	1274	single	Variation_36994	chrX	11855085	11874191	19106	Kidd et al. (2008)	Paired End Mapping	
6003	chrX	13386513	13386675	162	7	chrX	13386482	13386678	196	single								
6004	chrX	13767733	13772682	4949	2	chrX	13767702	13772798	5096	single								
6005	chrX	14555261	14555751	490	6	chrX	14555230	14555769	539	single								
6006	chrX	14707798	14709023	1225	27	chrX	14707767	14709041	1274	HiConf								
100987	chrX	15243968	15287052	43084	2	chrX	15243901	15287903	44002	single								
6007	chrX	15602685	15604302	1617	22	chrX	15602654	15604320	1666	HiConf	Variation_3262	chrX	15568691	15633925	65234	Redon et al. (2006)	Affymetrix 500K EA SNP Mapping Array	
6008	chrX	16094792	16097585	2793	12	chrX	16094761	16097652	2891	HiConf								
6009	chrX	16097879	16098516	637	11	chrX	16097848	16098534	686	single								
6010	chrX	16382177	16388792	6615	5	chrX	16382146	16388908	6762	HiConf	Variation_7772	chrX	16382326	16388348	6022	de Smith et al. (2007)	Agilent 185k CGH Arrays/Agilent Custom CGH Arrays	
6011	chrX	16639918	16641877	1959	16	chrX	16639886	16641895	2009	single								
6012	chrX	16714544	16715181	637	21	chrX	16714513	16715199	686	single								
6013	chrX	17303132	17304651	1519	19	chrX	17303101	17304669	1568	HiConf								
6014	chrX	17583951	17584311	360	17	chrX	17583920	17584312	392	single								
6015	chrX	17788575	17789653	1078	24	chrX	17788544	17789671	1127	HiConf								

locus_id	chrom	start	end	length	Yoruba w/ event	putative chr	putative start	putative end	putative len	putative type	variation_id	DGV_chr	DGV_start	DGV_end	DGV_len	Reference	Method/platform	complex
6016	chrX	18041807	18042117	310	7	chrX	18041776	18042119	343	single								
6017	chrX	18188625	18190326	1701	33	chrX	18188580	18190344	1764	HiConf								
6018	chrX	18353300	18354231	931	26	chrX	18353269	18354249	980	single								
6019	chrX	18815125	18815455	330	6	chrX	18815094	18815584	490	single	Variation_7775	chrX	18589089	19056108	467019	de Smith et al. (2007)	Agilent 185k CGH Arrays/Agilent Custom CGH Arrays	
6020	chrX	18911851	18913076	1225	13	chrX	18911820	18913094	1274	HiConf	Variation_7775	chrX	18589089	19056108	467019	de Smith et al. (2007)	Agilent 185k CGH Arrays/Agilent Custom CGH Arrays	
6021	chrX	18993380	18994416	1036	2	chrX	18993013	18994434	1421	single	Variation_7775	chrX	18589089	19056108	467019	de Smith et al. (2007)	Agilent 185k CGH Arrays/Agilent Custom CGH Arrays	y
6022	chrX	18997013	19002109	5096	21	chrX	18996982	19002225	5243	HiConf	Variation_7775	chrX	18589089	19056108	467019	de Smith et al. (2007)	Agilent 185k CGH Arrays/Agilent Custom CGH Arrays	
6023	chrX	19307231	19308095	864	1	chrX	19305976	19309602	3626	single								
6024	chrX	19374168	19387249	13081	1	chrX	19374135	19387512	13377	single								
6025	chrX	19815019	19816242	1223	18	chrX	19814988	19816262	1274	single								
6026	chrX	20053894	20056295	2401	48	chrX	20053863	20056362	2499	HiConf								y
6027	chrX	20069672	20070122	450	49	chrX	20069641	20070131	490	single								
6028	chrX	20174385	20176002	1617	7	chrX	20174354	20176020	1666	HiConf								
6029	chrX	20194132	20195553	1421	26	chrX	20194101	20195571	1470	single								
6030	chrX	20835836	20836196	360	2	chrX	20835805	20836197	392	single								
6031	chrX	20938393	20944077	5684	11	chrX	20938362	20944193	5831	HiConf								
6032	chrX	21028357	21035658	7301	14	chrX	21028326	21035823	7497	single								
6033	chrX	21301924	21303198	1274	23	chrX	21301893	21303216	1323	HiConf								
6034	chrX	21584017	21586614	2597	15	chrX	21583986	21586681	2695	HiConf								
6035	chrX	21869442	21869523	81	5	chrX	21869411	21869558	147	single								
6036	chrX	21946127	21950439	4312	7	chrX	21946096	21950506	4410	HiConf								
6037	chrX	22646141	22647464	1323	2	chrX	22646110	22647482	1372	single								
6038	chrX	23259964	23263737	3773	9	chrX	23259933	23263804	3871	HiConf								
6039	chrX	23952922	23953804	882	23	chrX	23952891	23953822	931	HiConf								
6040	chrX	24077335	24078558	1223	20	chrX	24077302	24078576	1274	HiConf	Variation_9338	chrX	24043387	24088963	45576	Wang et al. (2007)	Illumina HumanHap550 BeadChip	
100988	chrX	24926209	24968158	41949	6	chrX	24926178	24969151	42973	single								
6041	chrX	27624835	27625962	1127	6	chrX	27624804	27625980	1176	HiConf								
100989	chrX	27680510	27685464	4954	1	chrX	27675617	27716532	40915	single								
6042	chrX	29176714	29178233	1519	4	chrX	29176683	29178251	1568	HiConf	Variation_3265	chrX	27901452	31506888	3605436	Redon et al. (2006)	Affymetrix 500K EA SNP Mapping Array	
6043	chrX	30211177	30219973	8796	6	chrX	30211122	30220138	9016	HiConf	Variation_9339	chrX	30211162	30215789	4627	Wang et al. (2007)	Illumina HumanHap550 BeadChip	y
6044	chrX	30714922	30734130	19208	12	chrX	30714891	30734540	19649	HiConf	Variation_37021	chrX	30703732	30719401	15669	Kidd et al. (2008)	Paired End Mapping	
6045	chrX	31065860	31080533	14673	2	chrX	31065829	31081068	15239	HiConf	Variation_3265	chrX	27901452	31506888	3605436	Redon et al. (2006)	Affymetrix 500K EA SNP Mapping Array	
6046	chrX	31212517	31227952	15435	1	chrX	31212486	31228264	15778	single	Variation_3265	chrX	27901452	31506888	3605436	Redon et al. (2006)	Affymetrix 500K EA SNP Mapping Array	y
6047	chrX	32554578	32555509	931	53	chrX	32554547	32555527	980	HiConf								
6048	chrX	32829468	32830840	1372	7	chrX	32829437	32830858	1421	HiConf								y
6049	chrX	32897529	32898803	1274	55	chrX	32897498	32898821	1323	HiConf	Variation_44409	chrX	32897235	32899009	1774	Bentley et al. (2008)	Illumina DNA sequencing	
6050	chrX	33119891	33125820	5929	4	chrX	33119860	33125936	6076	HiConf	Variation_0242	chrX	33118826	33252714	133888	Iafrate et al. (2004)	BAC Array CGH	
6051	chrX	33749126	33751697	2571	6	chrX	33749118	33751764	2646	HiConf	Variation_7782	chrX	33749050	33751445	2395	de Smith et al. (2007)	Agilent 185k CGH Arrays/Agilent Custom CGH Arrays	
6052	chrX	34349742	34356014	6272	31	chrX	34349711	34356130	6419	HiConf	Variation_37022	chrX	34333744	34355002	21258	Kidd et al. (2008)	Paired End Mapping	
6053	chrX	34584844	34585244	400	24	chrX	34584813	34585254	441	single								
6054	chrX	34770701	34783791	13090	4	chrX	34770670	34783949	13279	HiConf								
6055	chrX	35009674	35013006	3332	1	chrX	35009643	35013073	3430	single								y
6056	chrX	35352233	35354855	2622	4	chrX	35352202	35355093	2891	single								
6057	chrX	35504672	35504735	63	5	chrX	35504641	35504739	98	single								
6058	chrX	35527996	35544019	16023	11	chrX	35527965	35544331	16366	HiConf	Variation_22764	chrX	35537309	35545019	7710	Korbel et al. (2007)	Paired End Mapping	
6059	chrX	35623889	35625849	1960	8	chrX	35623858	35625867	2009	HiConf								y
100990	chrX	36637160	36663080	25920	19	chrX	36637129	36675496	38367	HiConf								
6061	chrX	36891617	36892017	400	20	chrX	36891586	36892027	441	single								
6062	chrX	36941401	36944145	2744	22	chrX	36941370	36944212	2842	HiConf	Variation_44410	chrX	36941255	36944341	3086	Bentley et al. (2008)	Illumina DNA sequencing	
6063	chrX	36971438	36971798	360	25	chrX	36971407	36971799	392	single								y
6064	chrX	38271604	38272360	756	58	chrX	38271573	38273680	2107	HiConf	Variation_11360	chrX	38271511	38272233	722	de Smith et al. (2007)	Agilent 185k CGH Arrays/Agilent Custom CGH Arrays	
6065	chrX	38469466	38471377	1911	6	chrX	38469435	38471395	1960	single	Variation_33902	chrX	38469372	38470015	643	Perry et al. (2008)	Agilent Custom CGH Arrays	
6066	chrX	38548062	38550071	2009	15	chrX	38548031	38550089	2058	single								
100991	chrX	39893827	39914332	20505	1	chrX	39803877	39932281	128404	HiConf	Variation_7785	chrX	39861571	40436512	574941	de Smith et al. (2007)	Agilent 185k CGH Arrays/Agilent Custom CGH Arrays	
6067	chrX	41217827	41218660	833	12	chrX	41217796	41218678	882	HiConf	Variation_7787	chrX	40635684	42901711	2266027	de Smith et al. (2007)	Agilent 185k CGH Arrays/Agilent Custom CGH Arrays	y
6068	chrX	41667353	41668039	686	23	chrX	41667322	41668057	735	single	Variation_7787	chrX	40635684	42901711	2266027	de Smith et al. (2007)	Agilent 185k CGH Arrays/Agilent Custom CGH Arrays	
6069	chrX	42089243	42092673	3430	2	chrX	42089212	42092740	3528	HiConf	Variation_7787	chrX	40635684	42901711	2266027	de Smith et al. (2007)	Agilent 185k CGH Arrays/Agilent Custom CGH Arrays	
6070	chrX	42510398	42513632	3234	13	chrX	42510367	42513699	3332	single	Variation_7787	chrX	40635684	42901711	2266027	de Smith et al. (2007)	Agilent 185k CGH Arrays/Agilent Custom CGH Arrays	
6071	chrX	43333745	43340607	6862	4	chrX	43333714	43341015	7301	single								
6072	chrX	43458107	43464232	6125	36	chrX	43458076	43464348	6272	HiConf								
6073	chrX	43830115	43832663	2548	28	chrX	43830084	43832730	2646	HiConf								
6074	chrX	44185561	44190216	4655	2	chrX	44185530	44190283	4753	single								
6075	chrX	44616663	44618574	1911	11	chrX	44616632	44618592	1960	HiConf								
6076	chrX	46656337	46657709	1372	11	chrX	46656306	46657727	1421	HiConf	Variation_9342	chrX	46653913	46795054	141141	Wang et al. (2007)	Illumina HumanHap550 BeadChip	
6077	chrX	46889528	46889788	260	8	chrX	46889497	46889791	294	single								
6078	chrX	46963077	46963527	450	26	chrX	46963046	46963536	490	single								
6079						chrX	47108478	47111026	2548	single								
6080	chrX	47317837	47319125	1288	6	chrX	47317806	47319227	1421	HiConf								
100993	chrX	47743791	48189031	445240	11	chrX	47743396	48193338	449942	HiConf	Variation_8304	chrX	47738828	48056984	318156	Pinto et al. (2007)	Affymetrix 500K SNP Mapping Array	
6082	chrX	48219927	48220377	450	21	chrX	48219896	48220386	490	single	Variation_7789	chrX	47747717	49029052	1281335	de Smith et al. (2007)	Agilent 185k CGH Arrays/Agilent Custom CGH Arrays	y
6083	chrX	48276865	48277894	1029	17	chrX	48276834	48277912	1078	single	Variation_7789	chrX	47747717	49029052	1281335	de Smith et al. (2007)	Agilent 185k CGH Arrays/Agilent Custom CGH Arrays	
6084	chrX	48282990	48283529	539	21	chrX	48282959	48283547	588	single	Variation_7789	chrX	47747717	49029052	1281335	de Smith et al. (2007)	Agilent 185k CGH Arrays/Agilent Custom CGH Arrays	y
6085	chrX	48574785	48575520	735	9	chrX	48574754	48575538	784	single	Variation_7789	chrX	47747717	49029052	1281335	de Smith et al. (2007)	Agilent 185k CGH Arrays/Agilent Custom CGH Arrays	
6086	chrX	48792198	48797588	5390	3	chrX	48792167	48797704	5537	HiConf	Variation_7789	chrX	47747717	49029052	1281335	de Smith et al. (2007)	Agilent 185k CGH Arrays/Agilent Custom CGH Arrays	
6088	chrX	49048174	49253680	205506	29	chrX	49048143	49257863	209720	HiConf	Variation_31551	chrX	49044351	49253943	209592	Perry et al. (2008)	Agilent Custom CGH Arrays	
6089	chrX	49269117	49278474	9357	21	chrX	49269084	49278639	9555	HiConf								
6090	chrX	49343938	49344869	931	23	chrX	49343907	49344887	980	single								y
6091	chrX	49530628	49531167	539	31	chrX	49530597	49531185	588	single								
6092	chrX	50229956	50231181	1225	26	chrX	50229925	50231199	1274	HiConf								
100995	chrX	51422763	51499180	76417	2	chrX	51422732	51500691	77959	single								
6094	chrX	52248021	52261447	13426	27	chrX	52247990	52261710	13720	HiConf								
6095	chrX	52270512	52284085	13573	32	chrX	52270481	52284348	13867	HiConf								
6096	chrX	52520902	52535376	14474	27	chrX	52520871	52535571	14700	HiConf								
6097	chrX	52543491	52570049	26558	29	chrX	52543460	52570606	27146	HiConf								
100997	chrX	52642888	52841681	198793	5	chrX	52642857	52845717	202860	HiConf								
6098	chrX	52939558	52962090	22532	1	chrX	52939527	52962606	23079	single	Variation_23256	chrX	52907733	55696471	2788738	Korbel et al. (2007)	Paired End Mapping	

locus_id	chrom	start	end	length	Yoruba w/ event	putative chr	putative start	putative end	putative len	putative type	variation_id	DGV_chr	DGV_start	DGV_end	DGV_len	Reference	Method/platform	complex
6099	chrX	53122671	53125170	2499	9	chrX	53122640	53125237	2597	single	Variation_23256	chrX	52907733	55696471	2788738	Korbel et al. (2007)	Paired End Mapping	
6100	chrX	53470032	53482478	12446	1	chrX	53470001	53482741	12740	single	Variation_23256	chrX	52907733	55696471	2788738	Korbel et al. (2007)	Paired End Mapping	
6101	chrX	53490171	53493945	3774	2	chrX	53490140	53495726	5586	single	Variation_23256	chrX	52907733	55696471	2788738	Korbel et al. (2007)	Paired End Mapping	
6102	chrX	53495806	53507374	11568	2	chrX	53495775	53507829	12054	HiConf	Variation_22864	chrX	53494342	53508866	14524	Korbel et al. (2007)	Paired End Mapping	
6103	chrX	55116992	55139168	22176	8	chrX	55108953	55139774	30821	HiConf	Variation_23256	chrX	52907733	55696471	2788738	Korbel et al. (2007)	Paired End Mapping	
6104	chrX	55565027	55565615	588	26	chrX	55564996	55565633	637	single	Variation_23256	chrX	52907733	55696471	2788738	Korbel et al. (2007)	Paired End Mapping	
6105	chrX	56063553	56067179	3626	1	chrX	56063522	56067246	3724	single								
6106	chrX	56685454	56687959	2505	26	chrX	56679158	56688125	8967	HiConf	Variation_38768	chrX	56678637	56689430	10793	McCarroll et al. (2008)	Affymetrix Human SNP Array 6.0	
6107	chrX	56809970	56823927	13957	23	chrX	56809939	56824198	14259	HiConf	Variation_31552	chrX	56811524	56824015	12491	Perry et al. (2008)	Agilent Custom CGH Arrays	
6108	chrX	56964216	56972454	8238	8	chrX	56964142	56972619	8477	HiConf	Variation_1848	chrX	56968188	56972761	4573	McCarroll et al. (2005)	Null genotypes	
6109	chrX	57760570	57774731	14161	45	chrX	57760539	57774994	14455	HiConf	Variation_37054	chrX	57764583	57766613	2030	Kidd et al. (2008)	Paired End Mapping	
6110	chrX	57952307	57953581	1274	13	chrX	57952276	57953599	1323	HiConf								
6111	chrX	58145973	58163546	17573	4	chrX	58145924	58163907	17983	HiConf	Variation_9344	chrX	58138149	58363397	225248	Wang et al. (2007)	Illumina HumanHap550 BeadChip	y
100999	chrX	58284870	58597417	312547	27	chrX	58284839	60105753	1820914	HiConf	Variation_1850	chrX	58480199	58499972	19773	McCarroll et al. (2005)	Null genotypes	
6114	chrX	61952716	61953794	1078	5	chrX	61952685	61953812	1127	single								
6115	chrX	62965301	62965938	637	16	chrX	62965270	62965956	686	HiConf								
6116	chrX	63341532	63341792	260	1	chrX	63341345	63342668	1323	single								
6117	chrX	63495432	63661003	165571	1	chrX	63495401	63664353	168952	HiConf	Variation_9346	chrX	63553284	63704137	150853	Wang et al. (2007)	Illumina HumanHap550 BeadChip	
6119	chrX	66074302	66079000	4698	4	chrX	66074271	66087354	13083	single								
6120	chrX	66087434	66103922	16488	1	chrX	66087403	66104259	16856	single								
6121	chrX	66680432	66682686	2254	5	chrX	66680401	66682704	2303	HiConf	Variation_4154	chrX	66416394	66716658	300264	Redon et al. (2006)	BAC Array CGH	
6122	chrX	67040689	67046805	6116	41	chrX	67040600	67046921	6321	HiConf	Variation_22769	chrX	67039639	67047433	7794	Korbel et al. (2007)	Paired End Mapping	
6123	chrX	67965310	67966829	1519	3	chrX	67965279	67966847	1568	single								
6124	chrX	68640579	68642115	1536	1	chrX	68640548	68643782	3234	HiConf								
6125	chrX	68691735	68692813	1078	8	chrX	68691704	68692831	1127	single								
6126	chrX	68752593	68753720	1127	16	chrX	68752562	68753738	1176	single	Variation_11365	chrX	68752805	68753390	585	de Smith et al. (2007)	Agilent 185k CGH Arrays/Agilent Custom CGH Arrays	
6127	chrX	69202119	69204667	2548	18	chrX	69202088	69204734	2646	HiConf								
6128	chrX	69648509	69650812	2303	11	chrX	69648478	69650830	2352	HiConf	Variation_37057	chrX	69645783	69665110	19327	Kidd et al. (2008)	Paired End Mapping	
6129	chrX	70040019	70040329	310	31	chrX	70039988	70040331	343	HiConf	Variation_7797	chrX	69557260	71024758	1467498	de Smith et al. (2007)	Agilent 185k CGH Arrays/Agilent Custom CGH Arrays	y
6130	chrX	70714510	70716513	2003	29	chrX	70714473	70716531	2058	single	Variation_7797	chrX	69557260	71024758	1467498	de Smith et al. (2007)	Agilent 185k CGH Arrays/Agilent Custom CGH Arrays	
6131	chrX	70753361	70753811	450	14	chrX	70753330	70753820	490	single	Variation_7797	chrX	69557260	71024758	1467498	de Smith et al. (2007)	Agilent 185k CGH Arrays/Agilent Custom CGH Arrays	
101001	chrX	70765309	70786000	20691	1	chrX	70765090	70939187	174097	HiConf	Variation_7797	chrX	69557260	71024758	1467498	de Smith et al. (2007)	Agilent 185k CGH Arrays/Agilent Custom CGH Arrays	
6136	chrX	71325926	71332745	6819	2	chrX	71325895	71332804	6909	HiConf								
101002	chrX	71368375	71450803	82428	1	chrX	71368305	71452487	84182	HiConf								
101003	chrX	71907093	71945275	38182	4	chrX	71907011	71946162	39151	single								y
6138	chrX	72139571	72140021	450	32	chrX	72139540	72140030	490	single								
6139	chrX	72225321	72226154	833	5	chrX	72225290	72226172	882	single								
6140	chrX	72743349	72747367	4018	26	chrX	72743318	72747434	4116	HiConf	Variation_37058	chrX	72727568	72756079	28511	Kidd et al. (2008)	Paired End Mapping	
6141	chrX	73082429	73086839	4410	23	chrX	73082398	73086906	4508	HiConf								
6142	chrX	73556651	73560571	3920	3	chrX	73556620	73560638	4018	single								
6143	chrX	74331096	74332027	931	11	chrX	74331065	74332045	980	HiConf								
6144	chrX	75222732	75225100	2368	10	chrX	75220023	75225217	5194	single								
6145	chrX	77008456	77010318	1862	25	chrX	77008425	77010336	1911	single								
6146	chrX	77405013	77410942	5929	20	chrX	77404982	77411058	6076	HiConf								
6147	chrX	78809451	78811215	1764	15	chrX	78809420	78811233	1813	single	Variation_37059	chrX	78804588	78818243	13655	Kidd et al. (2008)	Paired End Mapping	
6148	chrX	79048228	79064937	16709	2	chrX	79048197	79065249	17052	single	Variation_8308	chrX	79016244	79125114	108870	Pinto et al. (2007)	Affymetrix 500K SNP Mapping Array	
6149	chrX	80111871	80118290	6419	4	chrX	80111840	80118406	6566	single	Variation_7799	chrX	79876274	80256797	380523	de Smith et al. (2007)	Agilent 185k CGH Arrays/Agilent Custom CGH Arrays	
6150	chrX	80303265	80304000	735	10	chrX	80303234	80304018	784	HiConf								
6151	chrX	80635436	80666208	30772	6	chrX	80635405	80666814	31409	HiConf								
6152	chrX	81060462	81062471	2009	5	chrX	81060431	81062489	2058	HiConf								
6153	chrX	81179091	81184579	5488	2	chrX	81179060	81184695	5635	HiConf								
101004	chrX	81250792	81302147	51355	7	chrX	81250600	81303349	52749	HiConf	Variation_1854	chrX	81282996	81298625	15629	McCarroll et al. (2005)	Null genotypes	
6155	chrX	81854948	81870873	15925	9	chrX	81854917	81871185	16268	HiConf	Variation_37062	chrX	81863827	81878282	14455	Kidd et al. (2008)	Paired End Mapping	
6156	chrX	84777210	84783286	6076	6	chrX	84777179	84783402	6223	HiConf								
6157	chrX	86422434	86425227	2793	2	chrX	86422403	86425294	2891	single								
6158	chrX	86915864	86917138	1274	4	chrX	86915833	86917499	1666	HiConf								
6159	chrX	87108875	87110835	1960	3	chrX	87108844	87110853	2009	single								
6160	chrX	87732988	87733348	360	22	chrX	87732957	87733349	392	HiConf								
6161	chrX	88346223	88348967	2744	24	chrX	88346192	88349034	2842	single	Variation_43728	chrX	88347646	88349076	1430	Wang et al. (2008)	Illumina DNA sequencing	
6162	chrX	88444272	88446379	2107	11	chrX	88444241	88446397	2156	HiConf								
6163	chrX	90353900	90357330	3430	2	chrX	90353869	90357397	3528	single								
6164	chrX	90856199	90865558	9359	11	chrX	90856168	90865723	9555	HiConf	Variation_1858	chrX	90858112	90864984	6872	McCarroll et al. (2005)	Mendelian inconsistencies/Null genotypes	y
6165	chrX	91253295	91255402	2107	22	chrX	91253264	91255420	2156	HiConf								
6166	chrX	91637749	91638386	637	25	chrX	91637718	91638404	686	HiConf								
101005	chrX	92193262	92268526	75264	2	chrX	92193231	92270063	76832	HiConf	Variation_1859	chrX	92200804	92224565	23761	McCarroll et al. (2005)	Null genotypes	
6168	chrX	92494563	92495837	1274	3	chrX	92494532	92495855	1323	single								
6169						chrX	92496247	92530645	34398	single								
6170	chrX	92608929	92610497	1568	8	chrX	92608898	92610515	1617	HiConf								
6171	chrX	92683043	92687721	4678	44	chrX	92683035	92687788	4753	HiConf	Variation_6742	chrX	92682956	92688163	5207	Mills et al. (2006)	Sequence trace read mapping	y
6172	chrX	93054535	93054895	360	21	chrX	93054504	93054896	392	HiConf								
6173	chrX	93073792	93074576	784	13	chrX	93073761	93074594	833	HiConf								
6174	chrX	93288216	93290519	2303	6	chrX	93288185	93290537	2352	HiConf	Variation_1860	chrX	93288229	93290555	2326	McCarroll et al. (2005)	Null genotypes	
6175	chrX	93335550	93337167	1617	2	chrX	93335519	93337185	1666	single								
6176	chrX	94585050	94587794	2744	5	chrX	94585019	94587861	2842	HiConf								
6177	chrX	94937574	94938055	481	60	chrX	94937574	94938064	490	HiConf	Variation_29370	chrX	94937503	94938136	633	Levy et al. (2007)	Sequencing	
6178	chrX	97054699	97054906	207	13	chrX	97054668	97054913	245	HiConf								
6179	chrX	97252904	97253214	310	1	chrX	97252873	97253216	343	single	Variation_9351	chrX	97236484	97320031	83547	Wang et al. (2007)	Illumina HumanHap550 BeadChip	
6180	chrX	97630743	97633928	3185	2	chrX	97630712	97633995	3283	single								
6181	chrX	97698412	97699147	735	7	chrX	97698381	97699165	784	single								
6182	chrX	97826694	97827233	539	8	chrX	97826663	97827251	588	single								
6183	chrX	99548358	99553797	5439	8	chrX	99548327	99553913	5586	HiConf	Variation_8309	chrX	98373457	99654701	1281244	Pinto et al. (2007)	Affymetrix 500K SNP Mapping Array	
101006	chrX	99763091	99783148	20057	1	chrX	99743788	99783968	40180	single								
6184	chrX	100110927	100116415	5488	21	chrX	100110896	100116531	5635	single								
6185	chrX	100627044	100627206	162	9	chrX	100627013	100627209	196	single								
6186	chrX	100627436	100635668	8232	21	chrX	100627405	100635833	8428	HiConf								
101007	chrX	100692165	100756003	63838	2	chrX	100692085	100757378	65293	single								

locus_id	chrom	start	end	length	Yoruba w/ event	putative chr	putative start	putative end	putative len	putative type	variation_id	DGV_chr	DGV_start	DGV_end	DGV_len	Reference	Method/platform	complex
101008	chrX	101263817	101292749	28932	3	chrX	101249754	101293634	43880	HiConf								
6188	chrX	102222386	102223954	1568	2	chrX	102222355	102223972	1617	single								
101009	chrX	102446561	102524335	77774	2	chrX	102446530	102525788	79258	single								
6189	chrX	102744187	102745363	1176	19	chrX	102744156	102745381	1225	single								
6190	chrX	103098898	103110168	11270	12	chrX	103098867	103110382	11515	HiConf	Variation_4160	chrX	102969057	103341717	372660	Redon et al. (2006)	BAC Array CGH	
101010	chrX	103867320	103883840	16520	1	chrX	103815664	103918931	103267	single								
101011	chrX	104130469	104137190	6721	1	chrX	104111697	104155601	43904	single								
6192	chrX	104276956	104280778	3822	1	chrX	104275945	104280845	4900	single	Variation_4161	chrX	104189662	104301693	112031	Redon et al. (2006)	BAC Array CGH	
6193						chrX	104408931	104408980	49	single								
6194	chrX	104537048	104539400	2352	6	chrX	104537017	104539418	2401	single								
6195	chrX	104952862	104953793	931	33	chrX	104952831	104953811	980	single	Variation_8310	chrX	104836584	106253758	1417174	Pinto et al. (2007)	Affymetrix 500K SNP Mapping Array	
6196	chrX	105693791	105694330	539	56	chrX	105693760	105694348	588	HiConf	Variation_8310	chrX	104836584	106253758	1417174	Pinto et al. (2007)	Affymetrix 500K SNP Mapping Array	
6197						chrX	106947229	106953844	6615	single								
6198	chrX	107865814	107867137	1323	27	chrX	107865783	107867155	1372	HiConf								y
6199	chrX	108184167	108184374	207	28	chrX	108184136	108184381	245	HiConf								
6200	chrX	108239978	108241112	1134	21	chrX	108239947	108243132	3185	HiConf								
6201	chrX	108784074	108788742	4668	9	chrX	108784043	108788796	4753	HiConf	Variation_1862	chrX	108781903	108860497	78594	McCarroll et al. (2005)	Mendelian inconsistencies/Null genotypes	
6202	chrX	109825324	109828852	3528	7	chrX	109825293	109828919	3626	HiConf	Variation_1864	chrX	109825861	109827815	1954	McCarroll et al. (2005)	Null genotypes	
6203	chrX	111731277	111734609	3332	13	chrX	111731246	111734676	3430	HiConf								
6204	chrX	111881119	111883422	2303	15	chrX	111881088	111883440	2352	HiConf								
6205	chrX	113205687	113208186	2499	9	chrX	113205656	113208253	2597	HiConf	Variation_7758	chrX	112815468	114902000	2086532	de Smith et al. (2007)	Agilent 185k CGH Arrays/Agilent Custom CGH Arrays	
6206	chrX	113722686	113725871	3185	12	chrX	113722655	113725938	3283	HiConf	Variation_7758	chrX	112815468	114902000	2086532	de Smith et al. (2007)	Agilent 185k CGH Arrays/Agilent Custom CGH Arrays	
6207	chrX	114330482	114333324	2842	14	chrX	114330451	114333391	2940	HiConf	Variation_7758	chrX	112815468	114902000	2086532	de Smith et al. (2007)	Agilent 185k CGH Arrays/Agilent Custom CGH Arrays	
6208	chrX	114671375	114671438	63	15	chrX	114671344	114671442	98	single	Variation_7758	chrX	112815468	114902000	2086532	de Smith et al. (2007)	Agilent 185k CGH Arrays/Agilent Custom CGH Arrays	
6209	chrX	114919854	114929997	10143	6	chrX	114919823	114930211	10388	HiConf	Variation_36992	chrX	114912288	114925414	13126	Kidd et al. (2008)	Paired End Mapping	
6211	chrX	115051713	115070382	18669	32	chrX	115051682	115070743	19061	HiConf	Variation_38782	chrX	115051983	115064666	12683	McCarroll et al. (2008)	Affymetrix Human SNP Array 6.0	
6212	chrX	116353447	116354525	1078	19	chrX	116353416	116354543	1127	HiConf								
6213	chrX	116786166	116789449	3283	4	chrX	116786135	116789516	3381	single								
6214	chrX	117338372	117338603	231	41	chrX	117338365	117338610	245	single	Variation_13241	chrX	117338173	117338998	825	Mills et al. (2006)	Sequence trace read mapping	
6215	chrX	117363778	117364856	1078	18	chrX	117363747	117364874	1127	single								
6216	chrX	117512983	117514796	1813	12	chrX	117512952	117514814	1862	HiConf								
6217	chrX	117745292	117746174	882	12	chrX	117745261	117746192	931	HiConf								
6218	chrX	117841920	117842557	637	25	chrX	117841889	117842575	686	single								
6219	chrX	117861226	117863284	2058	5	chrX	117861195	117863302	2107	HiConf								
6220	chrX	117991517	117994702	3185	11	chrX	117991486	117994769	3283	HiConf								
101013	chrX	118227918	118282455	54537	5	chrX	118227887	118283551	55664	single								
6221	chrX	118487005	118487315	310	28	chrX	118486974	118487317	343	single								
6222	chrX	118710396	118713532	3136	18	chrX	118710365	118713599	3234	HiConf								
6223						chrX	118775437	118777691	2254	HiConf								
6224	chrX	118917764	118943169	25405	12	chrX	118917733	118943262	25529	HiConf	Variation_31559	chrX	118918875	118942192	23317	Perry et al. (2008)	Agilent Custom CGH Arrays	
101014	chrX	118943870	119048839	104969	1	chrX	118943654	119050964	107310	single	Variation_6746	chrX	118945078	118952421	7343	Mills et al. (2006)	Sequence trace read mapping	
6225	chrX	119723569	119727048	3479	11	chrX	119723538	119727115	3577	HiConf								
6226	chrX	120804215	120804999	784	13	chrX	120804184	120805017	833	HiConf								
6227	chrX	121700474	121701797	1323	1	chrX	121700443	121701815	1372	single								
6228	chrX	121775689	121776669	980	4	chrX	121775658	121776687	1029	single								
6229	chrX	122067729	122083507	15778	1	chrX	122067698	122083819	16121	single								
6230	chrX	122559395	122561600	2205	3	chrX	122559364	122561618	2254	HiConf								y
6231	chrX	122779748	122780238	490	61	chrX	122779717	122780256	539	single	Variation_23912	chrX	122778924	122780533	1609	Levy et al. (2007)	Sequencing	
6232	chrX	122921113	122923808	2695	3	chrX	122921082	122923875	2793	HiConf	Variation_4169	chrX	122792497	122990246	197749	Redon et al. (2006)	BAC Array CGH	
6233	chrX	125126505	125128906	2401	18	chrX	125126474	125128973	2499	HiConf								
6234	chrX	125433539	125435499	1960	30	chrX	125433508	125435517	2009	HiConf	Variation_0253	chrX	125410171	125560547	150376	Iafrate et al. (2004)	BAC Array CGH	
6235	chrX	125512772	125514536	1764	21	chrX	125512741	125514554	1813	HiConf	Variation_0253	chrX	125410171	125560547	150376	Iafrate et al. (2004)	BAC Array CGH	
6236	chrX	125678000	125680499	2499	14	chrX	125677969	125680566	2597	HiConf	Variation_6748	chrX	125678032	125680572	2540	Mills et al. (2006)	Sequence trace read mapping	
6237	chrX	126427063	126430052	2989	23	chrX	126427032	126430119	3087	HiConf	Variation_23076	chrX	126425157	126431507	6350	Korbel et al. (2007)	Paired End Mapping	
6238	chrX	127259818	127260025	207	22	chrX	127259787	127260032	245	HiConf								
101015						chrX	127833332	127933194	99862	single								
6239	chrX	128328165	128330076	1911	43	chrX	128328134	128330094	1960	HiConf	Variation_44404	chrX	128328121	128330119	1998	Bentley et al. (2008)	Illumina DNA sequencing	
6240	chrX	129132892	129134019	1127	11	chrX	129132861	129134037	1176	single								
6241	chrX	129495256	129496374	1118	8	chrX	129492129	129496441	4312	HiConf								
101016	chrX	130135775	130198691	62916	7	chrX	130135744	130199983	64239	single								
6242	chrX	130640867	130641798	931	5	chrX	130640836	130641816	980	HiConf								
6243	chrX	130833339	130836181	2842	38	chrX	130833308	130836248	2940	HiConf								
6244	chrX	130984945	130985582	637	29	chrX	130984914	130985600	686	single								
6245	chrX	131450886	131451817	931	19	chrX	131450855	131451835	980	HiConf								
6246	chrX	131572847	131573386	539	7	chrX	131572816	131573404	588	single								
6247	chrX	131741309	131741848	539	9	chrX	131741278	131741866	588	single								y
6248	chrX	132376741	132377672	931	21	chrX	132376710	132377690	980	single								
6249	chrX	132613950	132615616	1666	9	chrX	132613919	132615634	1715	single								
6250	chrX	132615812	132624191	8379	4	chrX	132615781	132624356	8575	single								
6251	chrX	132946660	132948720	2060	16	chrX	132946629	132948736	2107	HiConf								
6252	chrX	133222187	133225149	2962	11	chrX	133222156	133225194	3038	HiConf								
6253	chrX	134057000	134060528	3528	5	chrX	134056969	134060595	3626	HiConf	Variation_4173	chrX	134028553	134325981	297428	Redon et al. (2006)	BAC Array CGH	
6254	chrX	134347423	134347733	310	16	chrX	134347392	134347735	343	HiConf	Variation_2280	chrX	134249130	134425978	176848	Locke et al. (2006)	BAC Array CGH	
6255	chrX	134394022	134400539	6517	1	chrX	134393991	134400655	6664	HiConf	Variation_33177	chrX	134394392	134399555	5163	Perry et al. (2008)	Agilent Custom CGH Arrays	
6256	chrX	134559201	134561455	2254	4	chrX	134559170	134561473	2303	single	Variation_4174	chrX	134430203	134982407	552204	Redon et al. (2006)	BAC Array CGH	
6257	chrX	134568217	134803155	234938	17	chrX	134568186	134803239	235053	HiConf	Variation_3256	chrX	134568194	134775700	207506	Redon et al. (2006)	Affymetrix 500K EA SNP Mapping Array	
6258	chrX	134852551	134857611	5060	5	chrX	134845183	134857874	12691	HiConf	Variation_31564	chrX	134852662	134857258	4596	Perry et al. (2008)	Agilent Custom CGH Arrays	
6259	chrX	136014158	136016706	2548	7	chrX	136014127	136016773	2646	HiConf								
101018	chrX	136161459	136181643	20184	1	chrX	136076651	136278531	201880	single								
6260	chrX	136335059	136340890	5831	4	chrX	136335028	136341006	5978	single								
6261	chrX	137951134	137958086	6952	1	chrX	137950313	137958251	7938	single								
101019						chrX	138007766	138060441	52675	single								
6262	chrX	138112387	138114935	2548	20	chrX	138112356	138115002	2646	single								
6263	chrX	138601162	138602436	1274	15	chrX	138601131	138602454	1323	HiConf								
6264	chrX	138841164	138843516	2352	8	chrX	138841133	138843534	2401	HiConf								y
6265	chrX	139317248	139330282	13034	24	chrX	139317217	139330545	13328	HiConf	Variation_38767	chrX	139324076	139328860	4784	McCarroll et al. (2008)	Affymetrix Human SNP Array 6.0	

locus_id	chrom	start	end	length	Yoruba w/ event	putative chr	putative start	putative end	putative len	putative type	variation_id	DGV_chr	DGV_start	DGV_end	DGV_len	Reference	Method/platform	complex
6266	chrX	139330870	139332095	1225	2	chrX	139330839	139332113	1274	single	Variation_37004	chrX	139320148	139336467	16319	Kidd et al. (2008)	Paired End Mapping	
6267	chrX	139413827	139414464	637	18	chrX	139413796	139414482	686	single								
6268	chrX	139624135	139628741	4606	7	chrX	139624104	139628808	4704	single								
6269	chrX	139693029	139694352	1323	11	chrX	139692998	139694370	1372	HiConf								
6270	chrX	139981846	140033579	51733	2	chrX	139906883	140036145	129262	HiConf	Variation_4175	chrX	139813929	140661678	847749	Redon et al. (2006)	BAC Array CGH	
6271	chrX	140159313	140164185	4872	15	chrX	140159282	140164329	5047	HiConf	Variation_31566	chrX	140145405	140164882	19477	Perry et al. (2008)	Agilent Custom CGH Arrays	
6272	chrX	140175073	140180481	5408	3	chrX	140168298	140180744	12446	single	Variation_3257	chrX	140138272	140571564	433292	Redon et al. (2006)	Affymetrix 500K EA SNP Mapping Array	
6273	chrX	140306068	140306852	784	52	chrX	140306037	140306870	833	HiConf	Variation_8311	chrX	140181127	140382870	201743	Pinto et al. (2007)	Affymetrix 500K SNP Mapping Array	
6274	chrX	140490160	140514808	24648	11	chrX	140421873	140516688	94815	HiConf	Variation_31567	chrX	140480543	140521007	40464	Perry et al. (2008)	Agilent Custom CGH Arrays	
6275	chrX	140593453	140616973	23520	10	chrX	140593422	140617432	24010	HiConf	Variation_31568	chrX	140606574	140614853	8279	Perry et al. (2008)	Agilent Custom CGH Arrays	
6276	chrX	142389793	142392782	2989	6	chrX	142389762	142392849	3087	HiConf	Variation_37008	chrX	142377045	142424551	47506	Kidd et al. (2008)	Paired End Mapping	
6277	chrX	143464853	143467107	2254	1	chrX	143464822	143467125	2303	single								
6278	chrX	144229939	144232732	2793	55	chrX	144229908	144232799	2891	HiConf	Variation_43465	chrX	144229728	144232265	2537	Wang et al. (2008)	Illumina DNA sequencing	y
6279	chrX	145697146	145703271	6125	9	chrX	145697115	145703387	6272	single								
6280	chrX	145846008	145849291	3283	23	chrX	145845977	145849358	3381	HiConf								
6281	chrX	146167463	146176219	8756	28	chrX	146167417	146176384	8967	HiConf	Variation_31570	chrX	146167253	146176681	9428	Perry et al. (2008)	Agilent Custom CGH Arrays	
6282	chrX	146651421	146656539	5118	7	chrX	146651390	146656682	5292	HiConf	Variation_38761	chrX	146651390	146655904	4514	McCarroll et al. (2008)	Affymetrix Human SNP Array 6.0	
6283	chrX	146866237	146867217	980	6	chrX	146866206	146867235	1029	single	Variation_3272	chrX	146820296	146959504	139208	Redon et al. (2006)	Affymetrix 500K EA SNP Mapping Array	y
6284	chrX	147317799	147319693	1894	67	chrX	147317790	147319701	1911	HiConf	Variation_39580	chrX	147317388	147318927	1539	Wheeler et al. (2008)	Sequencing	y
6285	chrX	147388430	147390929	2499	18	chrX	147388399	147390996	2597	HiConf								
101023	chrX	147450775	147459592	8817	1	chrX	147434949	147522880	87931	HiConf								
6286	chrX	148451632	148462363	10731	13	chrX	148451601	148462577	10976	HiConf	Variation_38001	chrX	148452463	148462404	9941	McCarroll et al. (2008)	Affymetrix Human SNP Array 6.0	
6287	chrX	148494802	148494957	155	58	chrX	148494770	148494966	196	HiConf	Variation_29284	chrX	148494771	148494899	128	Levy et al. (2007)	Sequencing	
6288	chrX	148638371	148643614	5243	26	chrX	148638340	148643730	5390	single	Variation_4177	chrX	148524364	148790049	265685	Redon et al. (2006)	BAC Array CGH	
101024	chrX	148677130	148847356	170226	5	chrX	148677099	148850804	173705	HiConf	Variation_3258	chrX	148693067	148880464	187397	Redon et al. (2006)	Affymetrix 500K EA SNP Mapping Array	
6290	chrX	148854167	148859851	5684	22	chrX	148854136	148859967	5831	HiConf	Variation_31572	chrX	148851822	148859977	8155	Perry et al. (2008)	Agilent Custom CGH Arrays	
6291	chrX	149279928	149288405	8477	6	chrX	149279897	149288570	8673	HiConf								
6293	chrX	149487884	149488046	162	32	chrX	149487853	149488049	196	single								
6294	chrX	149612589	149612989	400	20	chrX	149612558	149612999	441	single								
6295	chrX	149687657	149691871	4214	9	chrX	149687626	149691938	4312	single								
6296	chrX	149903061	149903796	735	16	chrX	149903030	149903814	784	single								
6297	chrX	150019191	150021543	2352	20	chrX	150019160	150021561	2401	HiConf	Variation_44406	chrX	150018808	150021467	2659	Bentley et al. (2008)	Illumina DNA sequencing	
6298	chrX	150039673	150046098	6425	10	chrX	150039642	150046110	6468	single	Variation_39581	chrX	150044597	150046373	1776	Wheeler et al. (2008)	Sequencing	
6299	chrX	150096268	150097346	1078	14	chrX	150096237	150097364	1127	single								
6300	chrX	150457594	150462984	5390	25	chrX	150457563	150463100	5537	HiConf								
6301	chrX	150627771	150629143	1372	5	chrX	150627740	150629161	1421	HiConf	Variation_6752	chrX	150627556	150629154	1598	Mills et al. (2006)	Sequence trace read mapping	
6302	chrX	150834649	150841901	7252	10	chrX	150834618	150842066	7448	HiConf								
101026	chrX	151745553	151762033	16480	3	chrX	151733719	151766451	32732	single	Variation_3273	chrX	151652710	151768408	115698	Redon et al. (2006)	Affymetrix 500K EA SNP Mapping Array	
101027						chrX	151781739	151868053	86314	HiConf								
6304	chrX	151943274	151953417	10143	6	chrX	151943243	151953631	10388	single								
6305	chrX	152138343	152139323	980	28	chrX	152138312	152139341	1029	single	Variation_8314	chrX	152089732	152202025	112293	Pinto et al. (2007)	Affymetrix 500K SNP Mapping Array	
6306	chrX	152611732	152613923	2191	1	chrX	152611701	152627381	15680	HiConf								
6307	chrX	152694983	152709291	14308	2	chrX	152694952	152709554	14602	HiConf								
6308	chrX	152930470	152937308	6838	1	chrX	152873214	152937551	64337	single								
6309	chrX	152937729	152938758	1029	16	chrX	152937698	152938776	1078	single								
6310	chrX	153061845	153174311	112466	23	chrX	153061766	153176671	114905	HiConf	Variation_37014	chrX	153098879	153206129	107250	Kidd et al. (2008)	Paired End Mapping	
6311	chrX	153219871	153222298	2427	1	chrX	153219840	153251151	31311	HiConf								
6312						chrX	153439458	153449797	10339	single								
6313	chrX	153514851	153527591	12740	2	chrX	153514820	153527854	13034	HiConf								
6314	chrX	153761566	153770729	9163	4	chrX	153761535	153770894	9359	single	Variation_23331	chrX	153662541	154582606	920065	Levy et al. (2007)	Illumina HumanHap650Y BeadChip	
6315	chrX	153965455	153967208	1753	3	chrX	153965424	153967237	1813	single	Variation_23331	chrX	153662541	154582606	920065	Levy et al. (2007)	Illumina HumanHap650Y BeadChip	
6316	chrX	154097608	154098294	686	20	chrX	154097577	154098312	735	single	Variation_23331	chrX	153662541	154582606	920065	Levy et al. (2007)	Illumina HumanHap650Y BeadChip	
6317	chrX	154139209	154140042	833	69	chrX	154139178	154140060	882	HiConf	Variation_44407	chrX	154139111	154140146	1035	Bentley et al. (2008)	Illumina DNA sequencing	
6318	chrX	154209034	154209818	784	2	chrX	154209003	154209836	833	single	Variation_23331	chrX	153662541	154582606	920065	Levy et al. (2007)	Illumina HumanHap650Y BeadChip	y
6319	chrX	154263669	154264355	686	35	chrX	154263638	154264373	735	single	Variation_23331	chrX	153662541	154582606	920065	Levy et al. (2007)	Illumina HumanHap650Y BeadChip	y
6320	chrX	154431788	154437640	5852	9	chrX	154431757	154438911	7154	HiConf	Variation_37015	chrX	154428697	154462271	33574	Kidd et al. (2008)	Paired End Mapping	
6321	chrX	154443508	154450652	7144	10	chrX	154443419	154457286	13867	HiConf	Variation_37019	chrX	154442775	154465568	22793	Kidd et al. (2008)	Paired End Mapping	
6322	chrX	154573741	154573903	162	44	chrX	154573710	154573906	196	HiConf	Variation_44408	chrX	154571658	154574948	3290	Bentley et al. (2008)	Illumina DNA sequencing	
6323	chrX	154678454	154682962	4508	8	chrX	154678423	154683029	4606	single	Variation_0828	chrX	154535313	154695188	159875	Sharp et al. (2005)	BAC Array CGH	y
6324	chrX	154744016	154745290	1274	50	chrX	154743985	154745308	1323	HiConf	Variation_33910	chrX	154744350	154744673	323	Perry et al. (2008)	Agilent Custom CGH Arrays	
101029	chrX	154863454	154909171	45717	20	chrX	154863423	154910095	46672	HiConf	Variation_31576	chrX	154903113	154910982	7869	Perry et al. (2008)	Agilent Custom CGH Arrays	
101030	chrY	546	455892	455346	11	chrY	515	555857	555342	HiConf								
6334	chrY	594671	611692	17021	2	chrY	594640	612378	17738	single								
6335	chrY	773521	800275	26754	4	chrY	773490	800832	27342	single								
6336	chrY	1332241	1373281	41040	4	chrY	1331355	1374132	42777	single								
6337						chrY	1543917	1551120	7203	HiConf								
6338						chrY	1597817	1597866	49	single								
6339	chrY	1688793	1711605	22812	7	chrY	1676658	1712281	35623	HiConf								
6340	chrY	1870925	1874306	3381	12	chrY	1870894	1874373	3479	HiConf	Variation_33188	chrY	1870920	1874408	3488	Perry et al. (2008)	Agilent Custom CGH Arrays	
6341	chrY	2022548	2028068	5520	9	chrY	2022157	2028184	6027	HiConf								y
6342	chrY	2200548	2221422	20874	17	chrY	2200517	2221832	21315	HiConf								
6343	chrY	2343432	2344118	686	19	chrY	2343401	2344136	735	HiConf								
6344	chrY	2575692	2593234	17542	4	chrY	2575661	2593595	17934	HiConf								
6345	chrY	3772125	3780847	8722	9	chrY	3772094	3781012	8918	HiConf	Variation_31579	chrY	3657424	4174365	516941	Perry et al. (2008)	Agilent Custom CGH Arrays	
6346						chrY	4603232	4607152	3920	single								
6347	chrY	5543573	5543933	360	11	chrY	5543542	5543934	392	single								
101032	chrY	6096073	6145122	49049	2	chrY	6096042	6146120	50078	single	Variation_2283	chrY	6092614	6258231	165617	Locke et al. (2006)	BAC Array CGH	
6349	chrY	6175134	6192431	17297	11	chrY	6175103	6192792	17689	HiConf	Variation_31581	chrY	6178668	6192691	14023	Perry et al. (2008)	Agilent Custom CGH Arrays	
6350	chrY	6564782	6566742	1960	4	chrY	6564751	6566760	2009	HiConf								
6351	chrY	7506660	7512736	6076	7	chrY	7506629	7512852	6223	HiConf								
6352	chrY	7536942	7566097	29155	5	chrY	7536911	7566703	29792	single								
101033	chrY	9216540	9985399	768859	16	chrY	9216509	10001097	784588	HiConf	Variation_33190	chrY	9230964	9988378	757414	Perry et al. (2008)	Agilent Custom CGH Arrays	
6354	chrY	10250366	10260019	9653	10	chrY	10250335	10260233	9898	HiConf								
6355	chrY	10542553	10549119	6566	3	chrY	10542522	10549235	6713	single	Variation_4182	chrY	10449174	10714553	265379	Redon et al. (2006)	BAC Array CGH	y
6356	chrY	10626784	10644718	17934	3	chrY	10626753	10645079	18326	HiConf	Variation_31585	chrY	10629001	10639996	10995	Perry et al. (2008)	Agilent Custom CGH Arrays	
101034	chrY	11697140	12091744	394604	21	chrY	11697109	12098517	401408	HiConf								

locus_id	chrom	start	end	length	Yoruba w/ event	putative chr	putative start	putative end	putative len	putative type	variation_id	DGV_chr	DGV_start	DGV_end	DGV_len	Reference	Method/platform	complex
101035	chrY	12102150	12378553	276403	22	chrY	12102119	12378822	276703	HiConf								
6361	chrY	12843495	12846680	3185	2	chrY	12843464	12846747	3283	single	Variation_0830	chrY	12828561	12995792	167231	Sharp et al. (2005)	BAC Array CGH	
6362						chrY	14458994	14464237	5243	single								
6363	chrY	18088063	18090660	2597	1	chrY	18088032	18090727	2695	single								
6364	chrY	18183711	18188954	5243	1	chrY	18183680	18189070	5390	single								
6365	chrY	18454877	18475457	20580	6	chrY	18454846	18475867	21021	HiConf	Variation_2286	chrY	18468367	18668341	199974	Locke et al. (2006)	BAC Array CGH	
6366	chrY	18685716	18687720	2004	8	chrY	18685685	18687743	2058	HiConf								
6367	chrY	19058655	19059341	686	9	chrY	19058624	19059359	735	HiConf								y
6368	chrY	19059586	19060223	637	7	chrY	19059555	19060241	686	HiConf								
6369	chrY	19155381	19157341	1960	2	chrY	19155350	19157359	2009	HiConf	Variation_31586	chrY	19154351	19167870	13519	Perry et al. (2008)	Agilent Custom CGH Arrays	
6370	chrY	19405330	19406653	1323	7	chrY	19405299	19406671	1372	HiConf	Variation_4183	chrY	19134214	19464452	330238	Redon et al. (2006)	BAC Array CGH	
6371	chrY	22204553	22206709	2156	1	chrY	22204522	22206727	2205	HiConf	Variation_31589	chrY	22063090	22481793	418703	Perry et al. (2008)	Agilent Custom CGH Arrays	
101037	chrY	22432360	22644573	212213	4	chrY	22432274	22648879	216605	HiConf	Variation_33912	chrY	22495335	22495714	379	Perry et al. (2008)	Agilent Custom CGH Arrays	
6373	chrY	22764708	22826059	61351	3	chrY	22764641	22827361	62720	HiConf	Variation_38809	chrY	22771258	22874762	103504	McCarroll et al. (2008)	Affymetrix Human SNP Array 6.0	
6374	chrY	22827509	22877813	50304	3	chrY	22827508	22878811	51303	HiConf	Variation_33495	chrY	22868976	22869223	247	Perry et al. (2008)	Agilent Custom CGH Arrays	
6375	chrY	22916327	22932674	16347	3	chrY	22916296	22933103	16807	HiConf	Variation_38810	chrY	22919906	22926380	6474	McCarroll et al. (2008)	Affymetrix Human SNP Array 6.0	
6376	chrY	23056271	23070040	13769	3	chrY	23056240	23070303	14063	single	Variation_4185	chrY	23005517	27194739	4189222	Redon et al. (2006)	BAC Array CGH	
6377	chrY	23302300	23364285	61985	7	chrY	23302269	23365528	63259	HiConf	Variation_4185	chrY	23005517	27194739	4189222	Redon et al. (2006)	BAC Array CGH	
6378	chrY	23618154	23627562	9408	11	chrY	23618123	23627727	9604	single	Variation_33192	chrY	23391116	24081466	690350	Perry et al. (2008)	Agilent Custom CGH Arrays	
6379	chrY	23628439	23667350	38911	16	chrY	23628364	23668152	39788	single	Variation_33192	chrY	23391116	24081466	690350	Perry et al. (2008)	Agilent Custom CGH Arrays	
6380	chrY	23668976	23723161	54185	12	chrY	23668936	23724257	55321	single	Variation_0834	chrY	23670847	23835302	164455	Sharp et al. (2005)	BAC Array CGH	
6381	chrY	23914408	24009746	95338	3	chrY	23914377	24011495	97118	HiConf	Variation_2293	chrY	23933595	24101017	167422	Locke et al. (2006)	BAC Array CGH	
6382	chrY	24258189	24301854	43665	7	chrY	24258112	24302702	44590	HiConf	Variation_2288	chrY	24149359	24341760	192401	Locke et al. (2006)	BAC Array CGH	
6383	chrY	24552290	24561934	9644	9	chrY	24552259	24562108	9849	single	Variation_38828	chrY	24377178	24639899	262721	McCarroll et al. (2008)	Affymetrix Human SNP Array 6.0	
6384	chrY	24726248	24770732	44484	15	chrY	24726209	24771632	45423	HiConf	Variation_38829	chrY	24720477	25412124	691647	McCarroll et al. (2008)	Affymetrix Human SNP Array 6.0	
6385	chrY	24808952	24864763	55811	4	chrY	24808921	24865908	56987	single	Variation_38829	chrY	24720477	25412124	691647	McCarroll et al. (2008)	Affymetrix Human SNP Array 6.0	
6386	chrY	24874044	24954512	80468	8	chrY	24873993	24956068	82075	HiConf	Variation_38829	chrY	24720477	25412124	691647	McCarroll et al. (2008)	Affymetrix Human SNP Array 6.0	
6387	chrY	25249560	25252157	2597	9	chrY	25249529	25252224	2695	single	Variation_38829	chrY	24720477	25412124	691647	McCarroll et al. (2008)	Affymetrix Human SNP Array 6.0	
6388	chrY	25279842	25371472	91630	11	chrY	25279811	25373352	93541	HiConf	Variation_38829	chrY	24720477	25412124	691647	McCarroll et al. (2008)	Affymetrix Human SNP Array 6.0	
6389	chrY	25387446	25406719	19273	11	chrY	25387415	25407113	19698	single	Variation_38829	chrY	24720477	25412124	691647	McCarroll et al. (2008)	Affymetrix Human SNP Array 6.0	
6390	chrY	25407315	25527390	120075	11	chrY	25407309	25529809	122500	single	Variation_38829	chrY	24720477	25412124	691647	McCarroll et al. (2008)	Affymetrix Human SNP Array 6.0	
6391	chrY	25529912	25620882	90970	9	chrY	25529907	25622713	92806	HiConf	Variation_38830	chrY	25450517	26584649	1134132	McCarroll et al. (2008)	Affymetrix Human SNP Array 6.0	
6392	chrY	25878707	25888765	10058	9	chrY	25878248	25888979	10731	single	Variation_38830	chrY	25450517	26584649	1134132	McCarroll et al. (2008)	Affymetrix Human SNP Array 6.0	
6393	chrY	25890882	26105002	214120	13	chrY	25890841	26109381	218540	HiConf	Variation_38830	chrY	25450517	26584649	1134132	McCarroll et al. (2008)	Affymetrix Human SNP Array 6.0	
6394	chrY	26338393	26410615	72222	8	chrY	26338309	26412103	73794	single	Variation_38830	chrY	25450517	26584649	1134132	McCarroll et al. (2008)	Affymetrix Human SNP Array 6.0	
6395	chrY	26412452	26414109	1657	11	chrY	26412446	26414112	1666	HiConf	Variation_38830	chrY	25450517	26584649	1134132	McCarroll et al. (2008)	Affymetrix Human SNP Array 6.0	
6396	chrY	26530224	26621903	91679	4	chrY	26530193	26623783	93590	single	Variation_33194	chrY	26548938	26554821	5883	Perry et al. (2008)	Agilent Custom CGH Arrays	
6397	chrY	26861660	26865531	3871	6	chrY	26861629	26865598	3969	HiConf	Variation_4185	chrY	23005517	27194739	4189222	Redon et al. (2006)	BAC Array CGH	
6398	chrY	57411053	57441673	30620	8	chrY	57411022	57442137	31115	HiConf								y
6399	chrY	57603476	57604505	1029	24	chrY	57603445	57604523	1078	single	Variation_33916	chrY	57603550	57603873	323	Perry et al. (2008)	Agilent Custom CGH Arrays	
6400	chrY	57691676	57695939	4263	1	chrY	57691645	57696006	4361	single								
6401	chrY	57747389	57771252	23863	4	chrY	57747358	57771711	24353	HiConf	Variation_31593	chrY	57762313	57770182	7869	Perry et al. (2008)	Agilent Custom CGH Arrays	
