# Supplementary material for: High resolution discovery and confirmation of copy number variants in 90 Yoruba Nigerians
Source: Genome Biol. 2009 Nov 9;10(11):R125. doi: 10.1186/gb-2009-10-11-r125 (PMC3091319; doi:10.1186/gb-2009-10-11-r125)
Supplement: Additional data file 3 — Gel images correspond to 4% agarose (E-gel), gradient polyacrlyamide (PA gel), and 1% agarose (1% gel) electrophoresis gels. DNAs were run in pairs with one having an observed event (Event DNA_ID), and the other without an observed event (non-event DNA_ID). Confirmation calls (Call Lane 1 or 2) were made based on amplicon length differences in each DNA pair, and marked the status of each pair: confirm, maybe (ambiguous), no (no evidence of event), or fail (PCR did not yield expected amplicons). At a subset of regions, amplicon bands were excised and sequenced (seq). The lengths of the putative CNVs are also listed. [file gb-2009-10-11-r125-S3.PDF]

LocusID	Event DNA_ID	Event Lane	Event	non-event DNA_ID	non-event Amp	Call Lane1	Call Lane2	**status**	**seq**	E-gel	PA gel	1% gel	notes	putative locus len
516	NA19192	2	Loss	NA18505	1822 bp	non	Loss	**confirm**		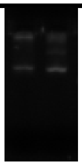		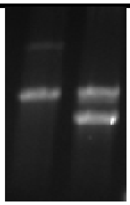	non (~1.8kb) in both. Lower Loss band in Lane2. (~1.6kb). Loss ~ 200 bp. Unexpected ~ 2.5kb in Lane1 only	980 bp
756	NA18515	1	Loss	NA18857	1989 bp	Loss	non	**confirm**	**read**	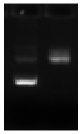				1519 bp
777	NA18505	1	Loss	NA19192	1382 bp	Loss2	Gain	**confirm**		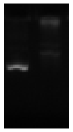		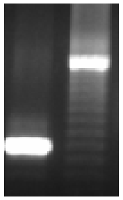	non (~1.3kb) NOT observed. Larger band in Lane2 (~ 1.9kb) suggests ~ 600 bp GAIN. Shorter band in Lane1 (~ 1kb) suggests ~300 bp LOSS	539 bp
954	NA19132	1	Loss	NA19101	2231 bp	Loss	non	**confirm**	**read**	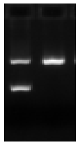				1813 bp
1017	NA18857	2	Loss	NA18515	2121 bp	non	Loss	**confirm**	**read**	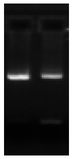			Faint Deletion band in Lane2	1715 bp
1105	NA18857	2	Loss	NA18515	1762 bp	non	Loss	**confirm**	**read**	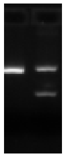				1078 bp

LocusID	Event DNA_ID	Event Lane	Event	non-event DNA_ID	non-event Amp	Call Lane1	Call Lane2	**status**	**seq**	E-gel	PA gel	1% gel	notes	putative locus len
1506	NA18871	2	Loss	NA18508	1909 bp	non	Loss2	**confirm**		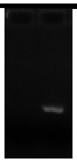	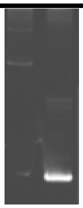		very faint non band (~2kb) in PA gel.	1421 bp
1565	NA18505	1	Gain	NA19192	1349 bp	Gain	Loss2	**confirm**		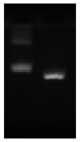		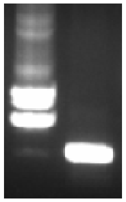	non (~1.3kb) in Lane1 Larger band in Lane1 (~ 1.4kb) suggests ~ 100 bp GAIN. Shorter band in Lane2 (~ 1kb) suggests ~300 bp LOSS	784 bp
1609	NA18505	1	Loss	NA19192	1395 bp	Loss2	non	**confirm**		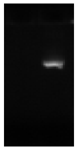	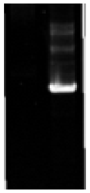		non (1.4kb) in Lane2. Unexpected higher bands in Lane2. Loss Band not observed in PA gel	1421 bp
2257	NA18505	1	Loss	NA19192	1622 bp	Loss2	Loss	**confirm**		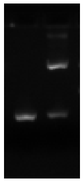			faint high band in Lane2	2254 bp
2466	NA18505	1	Loss	NA19192	1545 bp	Loss	non	**confirm**		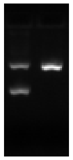				1960 bp

LocusID	Event DNA_ID	Event Lane	Event	non-event DNA_ID	non-event Amp	Call Lane1	Call Lane2	**status**	**seq**	E-gel	PA gel	1% gel	notes	putative locus len
2745	NA19132	1	Loss	NA19101	1403 bp	Loss2	non	**confirm**	**read**	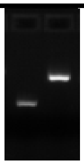				1078 bp
2786	NA19101	2	Gain	NA19132	1158 bp	non	Gain	**confirm**		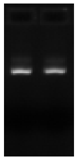		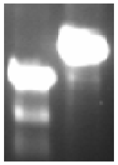	~ 1.2 kb non band in Lane1; ~ 1.4 kb in Lane2. Suggest ~ 200 bp GAIN	441 bp
2827	NA19132	1	Loss	NA19101	1772 bp	Loss	non	**confirm**	**read**	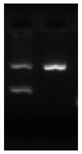				1274 bp
3157	NA18871	2	Loss	NA18508	2328 bp	non	Loss	**confirm**	**read**	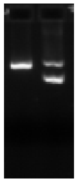				2058 bp
3186	NA19192	2	Loss	NA18505	1916 bp	non	Loss	**confirm**	**fail**	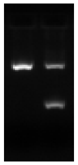				1519 bp
3262	NA19132	1	Loss	NA19101	1161 bp	Loss2	Loss	**confirm**	**read**	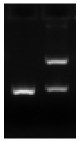				735 bp

LocusID	Event DNA_ID	Event Lane	Event	non-event DNA_ID	non-event Amp	Call Lane1	Call Lane2	**status**	**seq**	E-gel	PA gel	1% gel	notes	putative locus len
3689	NA18871	2	Loss	NA18508	2264 bp	Loss	Loss2	**confirm**	**read**	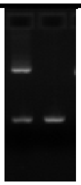				1813 bp
3860	NA19132	1	Loss	NA19101	1909 bp	Loss2	non	**confirm**	**read**	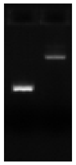				1127 bp
3907	NA18505	1	Loss	NA19192	2160 bp	Loss2	non	**confirm**	**read**	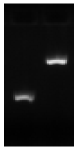				1470 bp
4235	NA19101	2	Loss	NA19132	1880 bp	Loss	Loss	**confirm**	**fail**	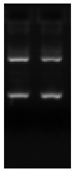			missed Loss in Lane1	1519 bp
4260	NA19192	2	Loss	NA18505	1446 bp	non	Loss2	**confirm**		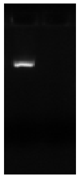	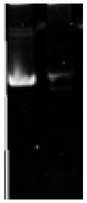		no band in Lane2.	1617 bp

LocusID	Event DNA_ID	Event Lane	Event	non-event DNA_ID	non-event Amp	Call Lane1	Call Lane2	**status**	**seq**	E-gel	PA gel	1% gel	notes	putative locus len
4292	NA18515	1	Loss	NA18857	2050 bp	Loss	non	**confirm**	**read**	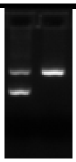				1470 bp
4582	NA18505	1	Loss	NA19192	1191 bp	Loss2	non	**confirm**	**read**	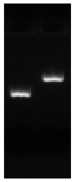				490 bp
4799	NA18505	1	Loss	NA19192	2474 bp	Loss2	non	**confirm**	**read**	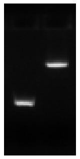				2842 bp
5388	NA19101	2	Loss	NA19132	1455 bp	non	Loss2	**confirm**		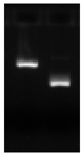		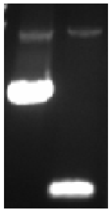	non band in Lane1 (~1.5kb). Faint unexpected ~3kb band in both Lanes.	931 bp
5439	NA18857	2	Loss	NA18515	1647 bp	Loss	Loss2	**confirm**	**read**	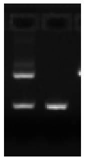				1225 bp

LocusID	Event DNA_ID	Event Lane	Event	non-event DNA_ID	non-event Amp	Call Lane1	Call Lane2	**status**	**seq**	E-gel	PA gel	1% gel	notes	putative locus len
6041	NA18508	1	Loss	NA18871	1667 bp	Loss	non	**confirm**	**read**	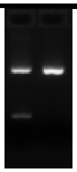				1176 bp
212	NA18505	1	Loss	NA19192	1798 bp	?	?	**fail**		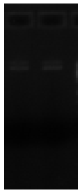			Expected ~ 1.8 kb NOT clearly observed.	1078 bp
5458	NA19132	1	Gain	NA19101	760 bp	?	?	**fail**		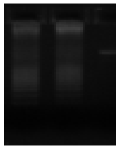		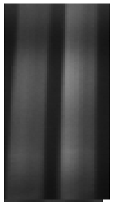	Expected ~ 0.7 kb NOT observed. Smears	686 bp
5682	NA18857	2	Loss	NA18515	1581 bp	?	?	**fail**		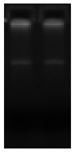		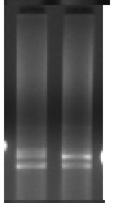	Expected ~ 1.6 kb NOT observed	1323 bp
6049	NA19132	1	Loss	NA19101	1715 bp	?	?	**fail**		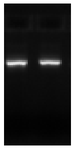		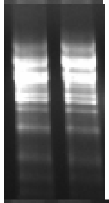	Expected ~ 1.7 kb NOT clearly observed. Many unexpected large bands	1323 bp
79	NA18871	2	Loss	NA18508	2279 bp	non	Loss	**maybe**		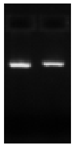		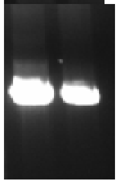	Lane2 may have less product. Possibly Loss	1372 bp

LocusID	Event DNA_ID	Event Lane	Event	non-event DNA_ID	non-event Amp	Call Lane1	Call Lane2	**status**	**seq**	E-gel	PA gel	1% gel	notes	putative locus len
932	NA18871	2	Loss	NA18508	2270 bp	non	Loss	**maybe**		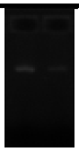		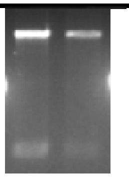	Lane2 may have less product. Possibly Loss	2205 bp
2364	NA19101	2	Loss	NA19132	2042 bp	non	Gain	**maybe**		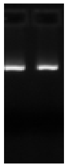		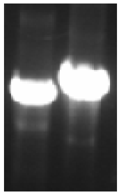	Band in Lane2 is larger than in Lane1; but expect Loss in Lane2	1519 bp
2453	NA18871	2	Loss	NA18508	1538 bp	Loss	Loss2	**maybe**		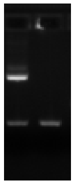		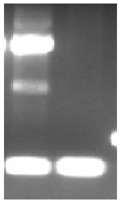	non band in Lane1 (~1.5kb). Faint unexpected bands in Lane1 only	1568 bp
3685	NA18871	2	Loss	NA18508	2679 bp	Loss2	non	**maybe**		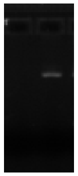	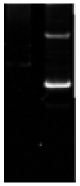		no band in Lane1. Expected non band (2.6 kb) NOT observed	882 bp
3734	NA19132	1	Gain	NA19101	1466 bp	non	Loss	**maybe**		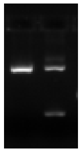			"Gain" may be non-event	1127 bp

LocusID	Event DNA_ID	Event Lane	Event	non-event DNA_ID	non-event Amp	Call Lane1	Call Lane2	**status**	**seq**	E-gel	PA gel	1% gel	notes	putative locus len
4505	NA18871	2	Loss	NA18508	1456 bp	non	Loss	**maybe**		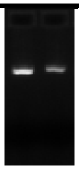		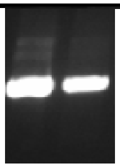	Lane2 may have less product. Possibly Loss	2842 bp
4617	NA19132	1	Loss	NA19101	1999 bp	Loss	non	**maybe**		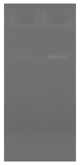	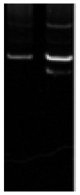		Faint non (~2kb) band in Lane1. Possibly Loss	2450 bp
5936	NA19192	2	Loss	NA18505	1569 bp	non	Loss	**maybe**		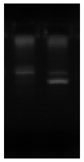		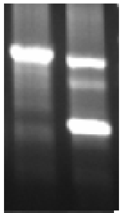	Faint unexpected bands	1078 bp
614	NA18871	2	Loss	NA18508	1432 bp	non	non	**no**		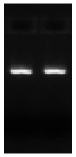		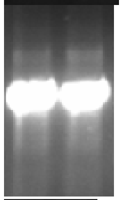	non-event bands (~1.4kb) in Both	1078 bp
4448	NA18515	1	Gain	NA18857	1671 bp	non	non	**no**		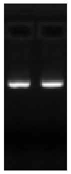		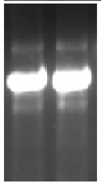	non-event bands (~1.6 kb) in Both	1029 bp

LocusID	Event DNA_ID	Event Lane	Event	non-event DNA_ID	non-event Amp	Call Lane1	Call Lane2	**status**	**seq**	E-gel	PA gel	1% gel	notes	putative locus len
5537	NA18857	2	Loss	NA18515	2352 bp	non	non	**no**		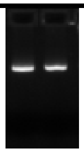		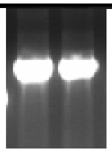	non-event bands (~2.3 kb) in Both.	2842 bp
