# Supplementary material for: High resolution discovery and confirmation of copy number variants in 90 Yoruba Nigerians
Source: Genome Biol. 2009 Nov 9;10(11):R125. doi: 10.1186/gb-2009-10-11-r125 (PMC3091319; doi:10.1186/gb-2009-10-11-r125)
Supplement: Additional data file 4 — (A) Event calls at confirmed CNVs are compared against consensus references from the Wang et al. [15] and McCarroll et al. [14] studies. Calls in red are in disagreement with the reference, and calls in blue are cases of possible false-positive calls not in the reference. Missed gain and loss events are shown as blue and red boxes, respectively. Consensus among the references and agreement with the references were determined by comparing loss versus gain events, and not integer copy numbers. Trio_ids are detailed in (D). (B) Calls reported in the McCarroll et al. [14] study are compared against consensus reference from our survey and the Wang et al. [15] study. (C) Calls reported in the Wang et al. [15] study are compared against consensus reference from our survey and the McCarroll et al. [14] study. (D) Yoruba trios were arbitrarily assigned trio_ids. The DNA_ids of the 90 Yoruba are listed with the corresponding trio_ids. [file gb-2009-10-11-r125-S4.PDF]

A

Trio_ID	Locus_ID	Father	Mother	Child	variation_id	Father	Mother	Child	variation_id	Father	Mother	Child
	Confirmed Loci				Wang et al (2007)				McCarroll et al (2008)			
5	1896	1	1	0	Variation_10096	1	1	1	Variation_37893	1	1	0
5	2542		3	3	Variation_9555		3	3	Variation_37811		3	3
5	2543		3	3	Variation_9555		3	3	Variation_37811		3	3
5	2593		1	1	Variation_10181		1	1	Variation_38909		1	1
5	3062	0		1	Variation_10255	0		1	Variation_37854	0		1
5	3804	1		1	Variation_9648	3		3	Variation_37784	3		3
5	3805			3	Variation_9648	3		3	Variation_37784	3		3
5	4138	1	1	1	Variation_10386		1	0	Variation_38837		1	0
5	4573	3		3	Variation_9724	3		3	Variation_37686	3		3
5	4956			1	Variation_10500		1	1	Variation_38340		1	1
5	5600		1	1	Variation_10566		1	1	Variation_38845		1	1
5	5782			1	Variation_10598	1		1	Variation_38452	1		1
5	100350			3	Variation_9525		3	3	Variation_37743		3	3
5	100646			3	Variation_9664	3		3	Variation_37788	3		3
5	100905			3	Variation_9788	3		3	Variation_37705	3		3
8	26	1		1	Variation_9870	1		1	Variation_37940	1		1
8	238		0	1	Variation_9367		0	1	Variation_38009		0	1
8	324	1		1	Variation_9899	1		1	Variation_38020	1		1
8	371		1	1	Variation_9905		1	1	Variation_38156		1	1
8	613	1		1	Variation_9929	1		1	Variation_37996	1		1
8	956		1	1	Variation_9975		1	1	Variation_37992		1	1
8	1061		1	1	Variation_9982		1	1	Variation_38498		1	1
8	2650		1	1	Variation_22462		1	1	Variation_38685		1	1
8	2954	1	0	1	Variation_10239		0	1	Variation_37887		0	1
8	3057		1	1	Variation_10254		1	1	Variation_38719		1	1
8	3698	1		1	Variation_10334	1		1	Variation_38207	1		1
8	3804	4		3	Variation_9648	3		3	Variation_37784	3		3
8	3805			3	Variation_9648	3		3	Variation_37784	3		3
8	3877		1	1	Variation_10350		1	1	Variation_38227		1	1
8	4573	3		3	Variation_9724	3		3	Variation_37686	3		3
8	5600		1	1	Variation_10566	3	1	1	Variation_38845	3	1	1
8	100214	1		1	Variation_10021	1		1	Variation_38478	1		1
8	100234	1		1	Variation_10037	1		1	Variation_38520	1		1
8	100615	1	0		Variation_10342	1	0		Variation_38189	1	1	
8	100632		1	1	Variation_10349		1	1	Variation_38226		1	1
9	2929		1	1	Variation_10234		1	1	Variation_38722		1	1
9	3065		1	1	Variation_10256		1	1	Variation_37922		1	1
9	3257	1		1	Variation_22490	1		1	Variation_38746	1		1
9	4904	3		3	Variation_9753	3		3	Variation_38878	3		3
9	4956	1		1	Variation_10500	1		1	Variation_38340	1		1
9	5600	4			Variation_10566	3		3	Variation_38845	4		3
9	5782	1	1	1	Variation_10598	1		1	Variation_38452	1		1
9	100214		1	1	Variation_10022		1	1	Variation_38478		1	1
9	100293	4		3	Variation_9501	3		3	Variation_38903	3		3
12	31			3	Variation_9358	3		3	Variation_38811	3		3
12	564		1	1	Variation_9923		1	1	Variation_38411		1	1
12	613		1	1	Variation_9929		1	1	Variation_37996		1	1
12	2043	1		1	Variation_10128	1		1	Variation_38585	1		1
12	2360		1	1	Variation_10162		1	1	Variation_37951		1	1
12	2954	1		1	Variation_10240	1		1	Variation_37924	1		1

A

Trio_ID	Locus_ID	Father	Mother	Child	variation_id	Father	Mother	Child	variation_id	Father	Mother	Child
	Confirmed Loci				Wang et al (2007)				McCarroll et al (2008)			
12	3674	1		1	Variation_10330	1		1	Variation_38202	1		1
12	3920		1	1	Variation_10360		1	1	Variation_38236		1	1
12	3921		0	1	Variation_10360		1	1	Variation_38236		1	1
12	3922		0	0	Variation_10360		1	1	Variation_38236		1	1
12	3923		1	1	Variation_10360		1	1	Variation_38236		1	1
12	3924		1	1	Variation_10360		1	1	Variation_38236		1	1
12	4791			1	Variation_9745		1	1	Variation_38819		1	1
12	4792		1	1	Variation_9745		1	1	Variation_38819		1	1
12	4793		1	1	Variation_9745		1	1	Variation_38819		1	1
12	4794		1	1	Variation_9745		1	1	Variation_38819		1	1
12	4795		1		Variation_9745		1	1	Variation_38819		1	1
12	4796		1		Variation_9745		1	1	Variation_38819		1	1
12	5600	1		1	Variation_10567	1		1	Variation_38845	1		1
12	5745		1	1	Variation_10587		1	1	Variation_38447		1	1
12	100214	1		1	Variation_10021	1		1	Variation_38478	1		1
12	100339	1	1	1	Variation_10129	1	1	1	Variation_38589	1	1	1
12	100459	1		1	Variation_10193	1		1	Variation_38688	1		1
12	100684	3		3	Variation_9681	3		3	Variation_37682	3		3
12	100803		3	3	Variation_9752		3	3	Variation_37691		3	3
12	100835		1	1	Variation_10505		1	1	Variation_38345		1	1
12	100864		1	1	Variation_10516		1	1	Variation_37949		1	1
12	100933	1		1	Variation_10574	1		1	Variation_38439	1		1
14	613		1	1	Variation_9929		1	1	Variation_37996		1	1
14	718		1		Variation_9954		0	1	Variation_37972		0	1
14	719		0	1	Variation_9954		0	1	Variation_37972		0	1
14	2380		1	1	Variation_10164		1	1	Variation_38622		1	1
14	2576	1	1		Variation_10177	1	1		Variation_38656	1	1	
14	3397	3		3	Variation_9617	3		3	Variation_37778	3		3
14	3448		3	4	Variation_9620		3	3	Variation_37769		3	3
14	3636	1		1	Variation_10325	1		1	Variation_38933	1		1
14	4576		1	1	Variation_10437		1	1	Variation_38870		1	1
14	5782	1		1	Variation_10598	1		1	Variation_38452	1		1
14	100334		1	1	Variation_10120		1	1	Variation_38581		1	1
14	100632		1	1	Variation_10349		1	1	Variation_38226		1	1
14	100905			4	Variation_9788		3	3	Variation_37705		3	3
14	100923		1		Variation_9816		0	1	Variation_38822		0	1
14	100949		1	1	Variation_10596		1	1	Variation_38451		1	1
16	324		1	1	Variation_9899		1	1	Variation_38020		1	1
16	582		1	1	Variation_9925		1	1	Variation_38895		1	1
16	613		1	1	Variation_9929		1	1	Variation_37996		1	1
16	2501	1		1	Variation_10169	1		1	Variation_38631	1		1
16	2502	1		1	Variation_10169	1		1	Variation_38631	1		1
16	2610	1		1	Variation_10183	1		1	Variation_38676	1		1
16	2954		1	1	Variation_10239		1	1	Variation_37887		1	1
16	3019	1		1	Variation_10250	1		1	Variation_38717	1		1
16	3065	1	1	0	Variation_10257	1	1	0	Variation_37922	1	1	0
16	3452	1		1	Variation_22486	1		1	Variation_38730	1		1
16	4053			3	Variation_9671		3	3	Variation_37661		3	3
16	4119		3	3	Variation_9679		3	3	Variation_37679		3	3
16	4754		0	1	Variation_10470		0	1	Variation_37848		0	1

A Trio_ID	Confirmed Loci Locus_ID	Father	Mother	Child	Wang et al (2007) variation_id	Father	Mother	Child	McCarroll et al (2008) variation_id	Father	Mother	Child
16	4792	1			Variation_9745	1		1	Variation_38819	1		1
16	4793	1		1	Variation_9745	1		1	Variation_38819	1		1
16	4794	1		1	Variation_9745	1		1	Variation_38819	1		1
16	4795	1			Variation_9745	1		1	Variation_38819	1		1
16	4863	1		1	Variation_10486	1		1	Variation_38332	1		1
16	5415		3	3	Variation_9783		3	3	Variation_37702		3	3
16	5600	3		4	Variation_10566	3		3	Variation_38845	4		4
16	100295	1		1	Variation_10079	1		1	Variation_38540	1		1
16	100379		3	3	Variation_9529		3	3	Variation_37752		3	3
16	100459		1	1	Variation_10193		1	1	Variation_38688		1	1
16	100905	4			Variation_9788	3		3	Variation_37706	3		3
16	100907	1			Variation_10546	0		1	Variation_37961	0		1
20	534	1		1	Variation_9920	1		1	Variation_38434	1		1
20	836	3			Variation_9432	3		3	Variation_37712	3		3
20	1475	1	1	0	Variation_10039	1	1	0	Variation_37871	1	1	0
20	1904		1	1	Variation_10099		1	1	Variation_38605		1	1
20	2610	1		1	Variation_10183	1		1	Variation_38676	1		1
20	3155		1	1	Variation_10267		1	1	Variation_38697		1	1
20	3400	1		1	Variation_10301	1		1	Variation_38747	1		1
20	3674		1	1	Variation_10330		1	1	Variation_38202		1	1
20	3698		1	1	Variation_10334		1	1	Variation_38207		1	1
20	3877		1	1	Variation_10350		1	1	Variation_38227		1	1
20	4200	1		1	Variation_10397	1		1	Variation_37935	1		1
20	4573		3	3	Variation_9725		3	3	Variation_37686		3	3
20	5782		1	1	Variation_10598		1	1	Variation_38452		1	1
20	100500	1		1	Variation_10230	1		1	Variation_38917	1		1
20	100540		1	1	Variation_10293		1	1	Variation_38733		1	1
22	238		1	1	Variation_9367		1	1	Variation_38009		1	1
22	1605	1		1	Variation_10050	1		1	Variation_38572	1		1
22	2360		1	1	Variation_10162		1	1	Variation_37951		1	1
22	2954	1		1	Variation_10240	1		1	Variation_37924	1		1
22	3674		1	1	Variation_10330		1	1	Variation_38202		1	1
22	4781			3	Variation_9743	3		3	Variation_38874	3		3
22	5434	3		3	Variation_9786	3		3	Variation_37703	3		3
22	5600	3		3	Variation_10566	3		3	Variation_38845	3		3
22	100561		1	1	Variation_10302		1	1	Variation_38752		1	1
22	100890	1	1	1	Variation_9785	1		1	Variation_38377	1		1
22	100915	1		1	Variation_9795	1		1	Variation_38821	1		1
23	238	1	1	0	Variation_9367	1	1	0	Variation_38009	1	1	0
23	606	1		1	Variation_9927	1		1	Variation_38428	1		1
23	613	1	1	1	Variation_9929	1	1	1	Variation_37996	1	1	1
23	822	1		1	Variation_9961	1		1	Variation_38402	1		1
23	2922		1	1	Variation_10227		1	1	Variation_38915		1	1
23	2966	1	1	1	Variation_10247	1	1	1	Variation_38710	1	1	1
23	3452		1	1	Variation_22486		1	1	Variation_38730		1	1
23	3636	3	3	3	Variation_9639	3	3	4	Variation_38933	3	3	4
23	4053			3	Variation_9671		3	3	Variation_37661		3	3
23	4060		1	1	Variation_10379		1	1	Variation_38219		1	1
23	4200	1	0	0	Variation_10397	1	0	0	Variation_37935	1	0	0
23	5600	1			Variation_9813		3	3	Variation_38845		3	3

A

Trio_ID	Confirmed Loci<br>Locus_ID	Father	Mother	Child	Wang et al (2007)<br>variation_id	Father	Mother	Child	McCarroll et al (2008)<br>variation_id	Father	Mother	Child
23	5663		1	1	Variation_10576		1	1	Variation_37880		1	1
23	100032	0		1	Variation_9887	0		1	Variation_37860	0		1
23	100060		1	1	Variation_9909		1	1	Variation_38160		1	1
25	238		1	1	Variation_9890		1	1	Variation_38009		1	1
25	425		1	1	Variation_9913		1	1	Variation_38166		1	1
25	1165	1		1	Variation_9999	1		1	Variation_38499	1		1
25	1455		**3**		Variation_9467	3		3	Variation_37807	3		3
25	2135		3		Variation_9524		3	3	Variation_37741		3	3
25	2136		3	3	Variation_9524		3	3	Variation_37741		3	3
25	2280	1		1	Variation_10152	1		1	Variation_38642	1		1
25	3683		1	1	Variation_10333		1	1	Variation_37988		1	1
25	4138	1	1		Variation_10386	1	1	1	Variation_38837	1	1	1
25	4169	1	1	1	Variation_10391	1	1	1	Variation_37946	1	1	1
25	4333	1		1	Variation_10404	1		1	Variation_38250	1		1
25	4580	3		4	Variation_9727	3		3	Variation_37792	4		4
25	4791	1			Variation_9745	0		1	Variation_38819	0		1
25	4792	1		1	Variation_9745	0		1	Variation_38819	0		1
25	4793	1		1	Variation_9745	0		1	Variation_38819	0		1
25	4794	1		1	Variation_9745	0		1	Variation_38819	0		1
25	4795	1		1	Variation_9745	0		1	Variation_38819	0		1
25	4796	1			Variation_9745	0		1	Variation_38819	0		1
25	5300		1	1	Variation_10523		1	1	Variation_38366		1	1
25	5600		3	3	Variation_9813	3	3	3	Variation_38845	3	3	3
25	100627	1		1	Variation_10346	1		1	Variation_38222	1		1
25	100646			3	Variation_9665	3		3	Variation_37788	3		3
25	100821		1	1	Variation_10502		1	1	Variation_38342		1	1
29	613	1	1		Variation_9929	1	1		Variation_37996	1	1	
29	1611	1		1	Variation_22434	1		1	Variation_38575	1		1
29	1718			1	Variation_10071		1	1	Variation_37986		1	1
29	1785		1	1	Variation_10080		1	1	Variation_38541		1	1
29	2097		3	3	Variation_9523		3	3	Variation_37740		3	3
29	2360	1	1		Variation_10162	1	1		Variation_37951	1	1	
29	2593	1		1	Variation_10181	1		1	Variation_38909	1		1
29	2954	1		1	Variation_10240	1		1	Variation_37924	1		1
29	3987	1		1	Variation_10372	1		1	Variation_38241	1		1
29	4052	1		1	Variation_10378	1		1	Variation_37906	1		1
29	4956	1			Variation_10500	1		1	Variation_38340	1		1
29	100339	1		1	Variation_10129	1		1	Variation_38589	1		1
29	100917		1	1	Variation_10557		1	1	Variation_38383		1	1
30	238	1		1	Variation_9367	1		1	Variation_38009	1		1
30	1223		1	1	Variation_10006		1	1	Variation_38509		1	1
30	2921	1	1		Variation_10226	1	1		Variation_38914	1	1	
30	3062		1	1	Variation_10255		1	1	Variation_37854		1	1
30	4580	3	3	4	Variation_9728	3	3	4	Variation_37792	4	4	4
30	4781	3			Variation_9743	3		3	Variation_38874	3		3
30	4791		1		Variation_9745	1	1		Variation_38819	1	1	
30	4792	1	1		Variation_9745	1	1		Variation_38819	1	1	
30	4793	1	1		Variation_9745	1	1		Variation_38819	1	1	
30	4794	1	1		Variation_9745	1	1		Variation_38819	1	1	
30	4795	1	1		Variation_9745	1	1		Variation_38819	1	1	

A

	Confirmed Loci				Wang et al (2007)				McCarroll et al (2008)			
**Trio_ID**	**Locus_ID**	**Father**	**Mother**	**Child**	variation_id	Father	Mother	Child	variation_id	Father	Mother	Child
30	4796	1	1		Variation_9745	1	1		Variation_38819	1	1	
30	5600		1		Variation_10566	3	1		Variation_38845	3	1	
30	100097	1		1	Variation_9926	1		1	Variation_38426	1		1
30	100534			**3**	Variation_10279		1	1	Variation_38743		1	1
30	100821	1		1	Variation_10502	1		1	Variation_38342	1		1
30	100890		1	1	Variation_10534		1	1	Variation_38377		1	1
30	100923			1	Variation_9816		1	1	Variation_38822		1	1

B	McCarroll et al (2008)				Wang et al (2007)				Confirmed Loci			
**Trio_ID**	**variation_id**	**Father**	**Mother**	**Child**	variation_id	Father	Mother	Child	Locus_ID	Father	Mother	Child
5	Variation_37686	3		3	Variation_9724	3		3	4573	3		3
5	Variation_37811		3	3	Variation_9555		3	3	2542		3	3
5	Variation_37854	0		1	Variation_10255	0		1	3062	0		1
5	Variation_37893	1	1	0	Variation_10096	1	1	1	1896	1	1	0
5	Variation_38189	1			Variation_10342	1		1	100615	1		1
5	Variation_38837		1	0	Variation_10386		1	0	4138		1	1
5	Variation_38845		1	1	Variation_10566		1	1	5600		1	1
5	Variation_38909		1	1	Variation_10181		1	1	2593		1	1
8	Variation_37686	3		3	Variation_9724	3		3	4573	3		3
8	Variation_37940	1		1	Variation_9870	1		1	26	1		1
8	Variation_37992		1	1	Variation_9975		1	1	956		1	1
8	Variation_37996	1		1	Variation_9929	1		1	613	1		1
8	Variation_38009		0	1	Variation_9367		0	1	238		0	1
8	Variation_38020	1		1	Variation_9899	1		1	324	1		1
8	Variation_38156		1	1	Variation_9905		1	1	371		1	1
8	Variation_38189	1	1		Variation_10342	1	0	1	100615	1	0	1
8	Variation_38207	1		1	Variation_10334	1		1	3698	1		1
8	Variation_38226		1	1	Variation_10349		1	1	100632		1	1
8	Variation_38227		1	1	Variation_10350		1	1	3877		1	1
8	Variation_38478	1		1	Variation_10021	1		1	100214	1		1
8	Variation_38498		1	1	Variation_9982		1	1	1061		1	1
8	Variation_38520	1		1	Variation_10037	1		1	100234	1		1
8	Variation_38685		1	1	Variation_22462		1	1	2650		1	1
8	Variation_38719		1	1	Variation_10254		1	1	3057		1	1
8	Variation_38845	3	1	1	Variation_10566		1	1	5600		1	1
9	Variation_37922		1	1	Variation_10256		1	1	3065		1	1
9	Variation_38340	1		1	Variation_10500	1		1	4956	1		1
9	Variation_38452	1		1	Variation_10598	1		1	5782	1		1
9	Variation_38478		1	1	Variation_10022		1	1	100214		1	1
9	Variation_38722		1	1	Variation_10234		1	1	2929		1	1
9	Variation_38746	1		1	Variation_22490	1		1	3257	1		1
9	Variation_38878	3		3	Variation_9753	3		3	4904	3		3
9	Variation_38903	3		3	Variation_9501	3		3	100293	4		3
12	Variation_37682	3		3	Variation_9681	3		3	100684	3		3
12	Variation_37691		3	3	Variation_9752		3	3	100803		3	3
12	Variation_37924	1		1	Variation_10240	1		1	2954	1		1
12	Variation_37949		1	1	Variation_10516		1	1	100864		1	1
12	Variation_37951		1	1	Variation_10162		1	1	2360		1	1
12	Variation_37996		1	1	Variation_9929		1	1	613		1	1
12	Variation_38202	1		1	Variation_10330	1		1	3674	1		1
12	Variation_38236		1	1	Variation_10360		1	1	3920		1	1
12	Variation_38345		1	1	Variation_10505		1	1	100835		1	1
12	Variation_38411		1	1	Variation_9923		1	1	564		1	1
12	Variation_38439	1		1	Variation_10574	1		1	100933	1		1
12	Variation_38447		1	1	Variation_10587		1	1	5745		1	1
12	Variation_38478	1		1	Variation_10021	1		1	100214	1		1
12	Variation_38585	1		1	Variation_10128	1		1	2043	1		1
12	Variation_38589	1	1	1	Variation_10129	1	1	1	100339	1	1	1
12	Variation_38688	1		1	Variation_10193	1		1	100459	1		1
12	Variation_38819		1	1	Variation_9745		1	1	4792		1	1

B

	McCarroll et al (2008)				Wang et al (2007)				Confirmed Loci			
Trio_ID	variation_id	Father	Mother	Child	variation_id	Father	Mother	Child	Locus_ID	Father	Mother	Child
12	Variation_38845	1		1	Variation_10567	1		1	5600	1		1
14	Variation_37769		3	3	Variation_9620		3	3	3448		3	4
14	Variation_37778	3		3	Variation_9617	3		3	3397	3		3
14	Variation_37972		0	1	Variation_9954		0	1	719		0	1
14	Variation_37996		1	1	Variation_9929		1	1	613		1	1
14	Variation_38226		1	1	Variation_10349		1	1	100632		1	1
14	Variation_38451		1	1	Variation_10596		1	1	100949		1	1
14	Variation_38452	1		1	Variation_10598	1		1	5782	1		1
14	Variation_38581		1	1	Variation_10120		1	1	100334		1	1
14	Variation_38622		1	1	Variation_10164		1	1	2380		1	1
14	Variation_38656	1	1		Variation_10177	1	1		2576	1	1	
14	Variation_38870		1	1	Variation_10437		1	1	4576		1	1
14	Variation_38933	1		1	Variation_10325	1		1	3636	1		1
16	Variation_37679		3	3	Variation_9679		3	3	4119		3	3
16	Variation_37702		3	3	Variation_9783		3	3	5415		3	3
16	Variation_37706	3		3	Variation_9788	3		3	100905	4		4
16	Variation_37752		3	3	Variation_9529		3	3	100379		3	3
16	Variation_37848		0	1	Variation_10470		0	1	4754		0	1
16	Variation_37887		1	1	Variation_10239		1	1	2954		1	1
16	Variation_37922	1	1	0	Variation_10257	1	1	0	3065	1	1	0
16	Variation_37996		1	1	Variation_9929		1	1	613		1	1
16	Variation_38020		1	1	Variation_9899		1	1	324		1	1
16	Variation_38189		1		Variation_10342	1	1	0	100615	1	1	0
16	Variation_38332	1		1	Variation_10486	1		1	4863	1		1
16	Variation_38540	1		1	Variation_10079	1		1	100295	1		1
16	Variation_38631	1		1	Variation_10169	1		1	2502	1		1
16	Variation_38676	1		1	Variation_10183	1		1	2610	1		1
16	Variation_38688		1	1	Variation_10193		1	1	100459		1	1
16	Variation_38717	1		1	Variation_10250	1		1	3019	1		1
16	Variation_38730	1		1	Variation_22486	1		1	3452	1		1
16	Variation_38819	1		1	Variation_9745	1		1	4793	1		1
16	Variation_38845	4		4	Variation_10566	3		3	5600	3		4
16	Variation_38895		1	1	Variation_9925		1	1	582		1	1
20	Variation_37686		3	3	Variation_9725		3	3	4573		3	3
20	Variation_37712	3		3	Variation_9432	3		3	836	3		3
20	Variation_37871	1	1	0	Variation_10039	1	1	0	1475	1	1	0
20	Variation_37935	1		1	Variation_10397	1		1	4200	1		1
20	Variation_38202		1	1	Variation_10330		1	1	3674		1	1
20	Variation_38207		1	1	Variation_10334		1	1	3698		1	1
20	Variation_38227		1	1	Variation_10350		1	1	3877		1	1
20	Variation_38434	1		1	Variation_9920	1		1	534	1		1
20	Variation_38452		1	1	Variation_10598		1	1	5782		1	1
20	Variation_38605		1	1	Variation_10099		1	1	1904		1	1
20	Variation_38676	1		1	Variation_10183	1		1	2610	1		1
20	Variation_38697		1	1	Variation_10267		1	1	3155		1	1
20	Variation_38733		1	1	Variation_10293		1	1	100540		1	1
20	Variation_38734		1	1	Variation_10293		1	1	100540		1	1
20	Variation_38747	1		1	Variation_10301	1		1	3400	1		1
20	Variation_38845		4		Variation_9813		3	3	5600		3	4
20	Variation_38909			1	Variation_22460	1		1	2593	1		1

B

Trio_ID	McCarroll et al (2008) variation_id	Father	Mother	Child	Wang et al (2007) variation_id	Father	Mother	Child	Confirmed Loci Locus_ID	Father	Mother	Child
20	Variation_38917	1		1	Variation_10230	1		1	100500	1		1
22	Variation_37703	3		3	Variation_9786	3		3	5434	3		3
22	Variation_37924	1		1	Variation_10240	1		1	2954	1		1
22	Variation_37951		1	1	Variation_10162		1	1	2360		1	1
22	Variation_38009		1	1	Variation_9367		1	1	238		1	1
22	Variation_38202		1	1	Variation_10330		1	1	3674		1	1
22	Variation_38377	1		1	Variation_9785	1		1	100890	1		1
22	Variation_38572	1		1	Variation_10050	1		1	1605	1		1
22	Variation_38752		1	1	Variation_10302		1	1	100561		1	1
22	Variation_38821	1		1	Variation_9795	1	3	1	100915	1	3	1
22	Variation_38845	3		3	Variation_10566	3		3	5600	3		3
23	Variation_37860	0		1	Variation_9887	0		1	100032	0		1
23	Variation_37880		1	1	Variation_10576		1	1	5663		1	1
23	Variation_37935	1	0	0	Variation_10397	1	0	0	4200	1	0	0
23	Variation_37996	1	1	1	Variation_9929	1	1	1	613	1	1	1
23	Variation_38009	1	1	0	Variation_9367	1	1	0	238	1	1	0
23	Variation_38160		1	1	Variation_9909		1	1	100060		1	1
23	Variation_38219		1	1	Variation_10379		1	1	4060		1	1
23	Variation_38402	1		1	Variation_9961	1		1	822	1		1
23	Variation_38428	1		1	Variation_9927	1		1	606	1		1
23	Variation_38710	1	1	1	Variation_10247	1	1	1	2966	1	1	1
23	Variation_38730		1	1	Variation_22486		1	1	3452		1	1
23	Variation_38915		1	1	Variation_10227		1	1	2922		1	1
23	Variation_38933	3	3	4	Variation_9639	3	3	4	3636	3	3	3
25	Variation_37741		3	3	Variation_9524		3	3	2136		3	3
25	Variation_37792	4		4	Variation_9727	3		3	4580	3		4
25	Variation_37946	1	1	1	Variation_10391	1	1	1	4169	1	1	1
25	Variation_37988		1	1	Variation_10333		1	1	3683		1	1
25	Variation_38009		1	1	Variation_9890		1	1	238		1	1
25	Variation_38079		1		Variation_10359		1	3	3920		1	3
25	Variation_38166		1	1	Variation_9913		1	1	425		1	1
25	Variation_38189		1		Variation_10342		1	1	100615		1	1
25	Variation_38222	1		1	Variation_10346	1		1	100627	1		1
25	Variation_38250	1		1	Variation_10404	1		1	4333	1		1
25	Variation_38342		1	1	Variation_10502		1	1	100821		1	1
25	Variation_38366		1	1	Variation_10523		1	1	5300		1	1
25	Variation_38499	1		1	Variation_9999	1		1	1165	1		1
25	Variation_38642	1		1	Variation_10152	1		1	2280	1		1
25	Variation_38819	0		1	Variation_9745	0		1	4792	1		1
25	Variation_38837	1	1	**1**	Variation_10386	1	1		4138	1	1	
25	Variation_38845	**3**	3	3	Variation_9813		3	3	5600		3	3
29	Variation_37740		3	3	Variation_9523		3	3	2097		3	3
29	Variation_37906	1		1	Variation_10378	1		1	4052	1		1
29	Variation_37924	1		1	Variation_10240	1		1	2954	1		1
29	Variation_37951	1	1		Variation_10162	1	1		2360	1	1	
29	Variation_37996	1	1		Variation_9929	1	1		613	1	1	
29	Variation_38189			1	Variation_10342		1	1	100615		1	1
29	Variation_38241	1		1	Variation_10372	1		1	3987	1		1
29	Variation_38383		1	1	Variation_10557		1	1	100917		1	1
29	Variation_38541		1	1	Variation_10080		1	1	1785		1	1

B	McCarroll et al (2008)				Wang et al (2007)				Confirmed Loci			
**Trio_ID**	**variation_id**	**Father**	**Mother**	**Child**	variation_id	Father	Mother	Child	Locus_ID	Father	Mother	Child
29	Variation_38575	1		1	Variation_22434	1		1	1611	1		1
29	Variation_38589	1		1	Variation_10129	1		1	100339	1		1
29	Variation_38909	1		1	Variation_10181	1		1	2593	1		1
30	Variation_37792	4	4	4	Variation_9728	3	3	4	4580	3	3	4
30	Variation_37854		1	1	Variation_10255		1	1	3062		1	1
30	Variation_38009	1		1	Variation_9367	1		1	238	1		1
30	Variation_38342	1		1	Variation_10502	1		1	100821	1		1
30	Variation_38377		1	1	Variation_10534		1	1	100890		1	1
30	Variation_38426	1		1	Variation_9926	1		1	100097	1		1
30	Variation_38509		1	1	Variation_10006		1	1	1223		1	1
30	Variation_38819	1	1		Variation_9745	1	1		4792	1	1	
30	Variation_38914	1	1		Variation_10226	1	1		2921	1	1	

C

Trio_ID	Wang et al (2007) variation_id	Father	Mother	Child	McCarroll et al (2008) variation_id	Father	Mother	Child	Confirmed Loci Locus_ID	Father	Mother	Child
5	Variation_10096	1	1	1	Variation_37893	1	1	0	1896	1	1	0
5	Variation_10181		1	1	Variation_38909		1	1	2593		1	1
5	Variation_10253		1		Variation_37943		1	1	3050		1	1
5	Variation_10255	0		1	Variation_37854	0		1	3062	0		1
5	Variation_10386		1	0	Variation_38837	1	1	0	4138	1	1	1
5	Variation_10387	1			Variation_38837	1	1	0	4138	1	1	1
5	Variation_10566		1	1	Variation_38845		1	1	5600		1	1
5	Variation_9555		3	3	Variation_37811		3	3	2542		3	3
5	Variation_9724	3		3	Variation_37686	3		3	4573	3		3
8	Variation_10021	1		1	Variation_38478	1		1	100214	1		1
8	Variation_10037	1		1	Variation_38520	1		1	100234	1		1
8	Variation_10254		1	1	Variation_38719		1	1	3057		1	1
8	Variation_10334	1		1	Variation_38207	1		1	3698	1		1
8	Variation_10342	1	0		Variation_38189	1	1		100615	1	0	
8	Variation_10349		1	1	Variation_38226		1	1	100632		1	1
8	Variation_10350		1	1	Variation_38227		1	1	3877		1	1
8	Variation_10566	3	1	1	Variation_38845		1	1	5600		1	1
8	Variation_22462		1	1	Variation_38685		1	1	2650		1	1
8	Variation_9367		0	1	Variation_38009		0	1	238		0	1
8	Variation_9648	3		3	Variation_37784	3		3	3804	4		3
8	Variation_9724	3		3	Variation_37686	3		3	4573	3		3
8	Variation_9870	1		1	Variation_37940	1		1	26	1		1
8	Variation_9899	1		1	Variation_38020	1		1	324	1		1
8	Variation_9905		1	1	Variation_38156		1	1	371		1	1
8	Variation_9929	1		1	Variation_37996	1		1	613	1		1
8	Variation_9975		1	1	Variation_37992		1	1	956		1	1
8	Variation_9982		1	1	Variation_38498		1	1	1061		1	1
9	Variation_10022		1	1	Variation_38478		1	1	100214		1	1
9	Variation_10234		1	1	Variation_38722		1	1	2929		1	1
9	Variation_10256		1	1	Variation_37922		1	1	3065		1	1
9	Variation_10321	1			Variation_38194	1		1	3593	1		1
9	Variation_10322			1	Variation_38194	1		1	3593	1		1
9	Variation_10359	1			Variation_38079	1		1	3920	1		1
9	Variation_10500	1		1	Variation_38340	1		1	4956	1		1
9	Variation_10598	1		1	Variation_38452	1	1	1	5782	1	1	1
9	Variation_10599		1		Variation_38452	1	1	1	5782	1	1	1
9	Variation_22490	1		1	Variation_38746	1		1	3257	1		1
9	Variation_9501	3		3	Variation_38903	3		3	100293	4		3
9	Variation_9753	3		3	Variation_38878	3		3	4904	3		3
9	Variation_9895			1	Variation_38813	1		1	280	1		1
12	Variation_10021	1		1	Variation_38478	1		1	100214	1		1
12	Variation_10128	1		1	Variation_38585	1		1	2043	1		1
12	Variation_10129	1	1	1	Variation_38589	1	1	1	100339	1	1	1
12	Variation_10162		1	1	Variation_37951		1	1	2360		1	1
12	Variation_10193	1		1	Variation_38688	1		1	100459	1		1
12	Variation_10240	1		1	Variation_37924	1		1	2954	1		1
12	Variation_10330	1		1	Variation_38202	1		1	3674	1		1
12	Variation_10360		1	1	Variation_38236		1	1	3920		1	1
12	Variation_10505		1	1	Variation_38345		1	1	100835		1	1
12	Variation_10516		1	1	Variation_37949		1	1	100864		1	1

C	Wang et al (2007)				McCarroll et al (2008)				Confirmed Loci			
**Trio_ID**	**variation_id**	**Father**	**Mother**	**Child**	variation_id	Father	Mother	Child	Locus_ID	Father	Mother	Child
12	Variation_10567	1		1	Variation_38845	1		1	5600	1		1
12	Variation_10574	1		1	Variation_38439	1		1	100933	1		1
12	Variation_10587		1	1	Variation_38447		1	1	5745		1	1
12	Variation_9681	3		3	Variation_37682	3		3	100684	3		3
12	Variation_9745		1	1	Variation_38819		1	1	4792		1	1
12	Variation_9752		3	3	Variation_37691		3	3	100803		3	3
12	Variation_9923		1	1	Variation_38411		1	1	564		1	1
12	Variation_9929		1	1	Variation_37996		1	1	613		1	1
14	Variation_10120		1	1	Variation_38581		1	1	100334		1	1
14	Variation_10164		1	1	Variation_38622		1	1	2380		1	1
14	Variation_10177	1	1		Variation_38656	1	1		2576	1	1	
14	Variation_10325	1		1	Variation_38933	1		1	3636	1		1
14	Variation_10349		1	1	Variation_38226		1	1	100632		1	1
14	Variation_10437		1	1	Variation_38870		1	1	4576		1	1
14	Variation_10514		1		Variation_38351		1	1	100852		1	1
14	Variation_10596		1	1	Variation_38451		1	1	100949		1	1
14	Variation_10598	1		1	Variation_38452	1		1	5782	1		1
14	Variation_9617	3		3	Variation_37778	3		3	3397	3		3
14	Variation_9620		3	3	Variation_37769		3	3	3448		3	4
14	Variation_9929		1	1	Variation_37996		1	1	613		1	1
14	Variation_9954		0	1	Variation_37972		0	1	719		0	1
16	Variation_10079	1		1	Variation_38540	1		1	100295	1		1
16	Variation_10169	1		1	Variation_38631	1		1	2501	1		1
16	Variation_10183	1		1	Variation_38676	1		1	2610	1		1
16	Variation_10193		1	1	Variation_38688		1	1	100459		1	1
16	Variation_10239		1	1	Variation_37887		1	1	2954		1	1
16	Variation_10250	1		1	Variation_38717	1		1	3019	1		1
16	Variation_10257	1	1	0	Variation_37922	1	1	0	3065	1	1	0
16	Variation_10470		0	1	Variation_37848		0	1	4754		0	1
16	Variation_10486	1		1	Variation_38332	1		1	4863	1		1
16	Variation_10566	3		3	Variation_38845	4		4	5600	3		4
16	Variation_22486	1		1	Variation_38730	1		1	3452	1		1
16	Variation_9529		3	3	Variation_37752		3	3	100379		3	3
16	Variation_9679		3	3	Variation_37679		3	3	4119		3	3
16	Variation_9745	1		1	Variation_38819	1		1	4793	1		1
16	Variation_9783		3	3	Variation_37702		3	3	5415		3	3
16	Variation_9813		3		Variation_38845	4		4	5600	3		4
16	Variation_9899		1	1	Variation_38020		1	1	324		1	1
16	Variation_9925		1	1	Variation_38895		1	1	582		1	1
16	Variation_9929		1	1	Variation_37996		1	1	613		1	1
20	Variation_10039	1	1	0	Variation_37871	1	1	0	1475	1	1	0
20	Variation_10099		1	1	Variation_38605		1	1	1904		1	1
20	Variation_10181		1		Variation_38909		1	1	2593		1	1
20	Variation_10183	1		1	Variation_38676	1		1	2610	1		1
20	Variation_10230	1		1	Variation_38917	1		1	100500	1		1
20	Variation_10267		1	1	Variation_38697		1	1	3155		1	1
20	Variation_10293		1	1	Variation_38734		1	1	100540		1	1
20	Variation_10301	1		1	Variation_38747	1		1	3400	1		1
20	Variation_10330		1	1	Variation_38202		1	1	3674		1	1
20	Variation_10334		1	1	Variation_38207		1	1	3698		1	1

C	Wang et al (2007)				McCarroll et al (2008)				Confirmed Loci			
**Trio_ID**	**variation_id**	**Father**	**Mother**	**Child**	variation_id	Father	Mother	Child	Locus_ID	Father	Mother	Child
20	Variation_10350		1	1	Variation_38227		1	1	3877		1	1
20	Variation_10397	1		1	Variation_37935	1		1	4200	1		1
20	Variation_10566	3			Variation_38845	4	4		5600	4	3	
20	Variation_10598		1	1	Variation_38452		1	1	5782		1	1
20	Variation_22460			1	Variation_38909		1	1	2593		1	1
20	Variation_9725		3	3	Variation_37686		3	3	4573		3	3
20	Variation_9813		3		Variation_38845	4	4		5600	4	3	
20	Variation_9920	1		1	Variation_38434	1		1	534	1		1
22	Variation_10050	1		1	Variation_38572	1		1	1605	1		1
22	Variation_10162		1	1	Variation_37951		1	1	2360		1	1
22	Variation_10240	1		1	Variation_37924	1		1	2954	1		1
22	Variation_10302		1	1	Variation_38752		1	1	100561		1	1
22	Variation_10330		1	1	Variation_38202		1	1	3674		1	1
22	Variation_10566	3		3	Variation_38845	3		3	5600	3		3
22	Variation_9367		1	1	Variation_38009		1	1	238		1	1
22	Variation_9785	1		1	Variation_38377	1	1	1	100890	1	1	1
22	Variation_9786	3		3	Variation_37703	3		3	5434	3		3
22	Variation_9795	1		1	Variation_38821	1		1	100915	1		1
23	Variation_10227		1	1	Variation_38915		1	1	2922		1	1
23	Variation_10247	1	1	1	Variation_38710	1	1	1	2966	1	1	1
23	Variation_10379		1	1	Variation_38219		1	1	4060		1	1
23	Variation_10397	1	0	0	Variation_37935	1	0	0	4200	1	0	0
23	Variation_10576		1	1	Variation_37880		1	1	5663		1	1
23	Variation_22486		1	1	Variation_38730		1	1	3452		1	1
23	Variation_9367	1	1	0	Variation_38009	1	1	0	238	1	1	0
23	Variation_9639	3	3	4	Variation_38933	3	3	4	3636	3	3	3
23	Variation_9887	0		1	Variation_37860	0		1	100032	0		1
23	Variation_9909		1	1	Variation_38160		1	1	100060		1	1
23	Variation_9927	1		1	Variation_38428	1		1	606	1		1
23	Variation_9929	1	1	1	Variation_37996	1	1	1	613	1	1	1
23	Variation_9961	1		1	Variation_38402	1		1	822	1		1
25	Variation_10152	1		1	Variation_38642	1		1	2280	1		1
25	Variation_10333		1	1	Variation_37988		1	1	3683		1	1
25	Variation_10346	1		1	Variation_38222	1		1	100627	1		1
25	Variation_10386	1	1	**1**	Variation_38837	1	1		4138	1	1	
25	Variation_10391	1	1	1	Variation_37946	1	1	1	4169	1	1	1
25	Variation_10404	1		1	Variation_38250	1		1	4333	1		1
25	Variation_10502		1	1	Variation_38342		1	1	100821		1	1
25	Variation_10523		1	1	Variation_38366		1	1	5300		1	1
25	Variation_9524		3	3	Variation_37741		3	3	2136		3	3
25	Variation_9727	3		3	Variation_37792	4		4	4580	3		4
25	Variation_9745	0		1	Variation_38819	0		1	4792	1		1
25	Variation_9813	**3**	3	3	Variation_38845		3	3	5600		3	3
25	Variation_9890		1	1	Variation_38009		1	1	238		1	1
25	Variation_9913		1	1	Variation_38166		1	1	425		1	1
25	Variation_9999	1		1	Variation_38499	1		1	1165	1		1
29	Variation_10080		1	1	Variation_38541		1	1	1785		1	1
29	Variation_10129	1		1	Variation_38589	1		1	100339	1		1
29	Variation_10162	1	1		Variation_37951	1	1		2360	1	1	
29	Variation_10181	1		1	Variation_38909	1		1	2593	1		1

C	Wang et al (2007)				McCarroll et al (2008)				Confirmed Loci			
**Trio_ID**	**variation_id**	**Father**	**Mother**	**Child**	variation_id	Father	Mother	Child	Locus_ID	Father	Mother	Child
29	Variation_10240	1		1	Variation_37924	1		1	2954	1		1
29	Variation_10372	1		1	Variation_38241	1		1	3987	1		1
29	Variation_10378	1		1	Variation_37906	1		1	4052	1		1
29	Variation_10557		1	1	Variation_38383		1	1	100917		1	1
29	Variation_22434	1		1	Variation_38575	1		1	1611	1		1
29	Variation_9523		3	3	Variation_37740		3	3	2097		3	3
29	Variation_9929	1	1		Variation_37996	1	1		613	1	1	
30	Variation_10006		1	1	Variation_38509		1	1	1223		1	1
30	Variation_10226	1	1		Variation_38914	1	1		2921	1	1	
30	Variation_10255		1	1	Variation_37854		1	1	3062		1	1
30	Variation_10502	1		1	Variation_38342	1		1	100821	1		1
30	Variation_10534		1	1	Variation_38377		1	1	100890		1	1
30	Variation_9367	1		1	Variation_38009	1		1	238	1		1
30	Variation_9728	3	3	4	Variation_37792	4	4	4	4580	3	3	4
30	Variation_9745	1	1		Variation_38819	1	1		4792	1	1	
30	Variation_9926	1		1	Variation_38426	1		1	100097	1		1

D

Trio_ID	DNA_ID	Father	Mother
1	NA18500	NA18501	NA18502
1	NA18501	0	0
1	NA18502	0	0
2	NA18503	NA18504	NA18505
2	NA18504	0	0
2	NA18505	0	0
3	NA18506	NA18507	NA18508
3	NA18507	0	0
3	NA18508	0	0
4	NA18860	NA18859	NA18858
4	NA18859	0	0
4	NA18858	0	0
5	NA18515	NA18516	NA18517
5	NA18516	0	0
5	NA18517	0	0
6	NA18521	NA18522	NA18523
6	NA18522	0	0
6	NA18523	0	0
7	NA18872	NA18871	NA18870
7	NA18871	0	0
7	NA18870	0	0
8	NA18854	NA18853	NA18852
8	NA18853	0	0
8	NA18852	0	0
9	NA18857	NA18856	NA18855
9	NA18856	0	0
9	NA18855	0	0
10	NA18863	NA18862	NA18861
10	NA18862	0	0
10	NA18861	0	0
11	NA18914	NA18913	NA18912
11	NA18913	0	0
11	NA18912	0	0
12	NA19094	NA19092	NA19093
12	NA19092	0	0
12	NA19093	0	0
13	NA19103	NA19101	NA19102
13	NA19101	0	0
13	NA19102	0	0
14	NA19139	NA19138	NA19137
14	NA19138	0	0
14	NA19137	0	0
15	NA19202	NA19200	NA19201
15	NA19200	0	0
15	NA19201	0	0
16	NA19173	NA19171	NA19172
16	NA19171	0	0
16	NA19172	0	0
17	NA19205	NA19203	NA19204
17	NA19203	0	0
17	NA19204	0	0
18	NA19211	NA19210	NA19209

D

Trio_ID	DNA_ID	Father	Mother
18	NA19210	0	0
18	NA19209	0	0
19	NA19208	NA19207	NA19206
19	NA19207	0	0
19	NA19206	0	0
20	NA19161	NA19160	NA19159
20	NA19160	0	0
20	NA19159	0	0
21	NA19221	NA19223	NA19222
21	NA19223	0	0
21	NA19222	0	0
22	NA19120	NA19119	NA19116
22	NA19119	0	0
22	NA19116	0	0
23	NA19142	NA19141	NA19140
23	NA19141	0	0
23	NA19140	0	0
24	NA19154	NA19153	NA19152
24	NA19153	0	0
24	NA19152	0	0
25	NA19145	NA19144	NA19143
25	NA19144	0	0
25	NA19143	0	0
26	NA19129	NA19128	NA19127
26	NA19128	0	0
26	NA19127	0	0
27	NA19132	NA19130	NA19131
27	NA19130	0	0
27	NA19131	0	0
28	NA19100	NA19098	NA19099
28	NA19098	0	0
28	NA19099	0	0
29	NA19194	NA19192	NA19193
29	NA19192	0	0
29	NA19193	0	0
30	NA19240	NA19239	NA19238
30	NA19239	0	0
30	NA19238	0	0
