# Supplementary material for: High resolution discovery and confirmation of copy number variants in 90 Yoruba Nigerians
Source: Genome Biol. 2009 Nov 9;10(11):R125. doi: 10.1186/gb-2009-10-11-r125 (PMC3091319; doi:10.1186/gb-2009-10-11-r125)
Supplement: Additional data file 5 — Primer sequences, along with sizes of the expected amplicons. [file gb-2009-10-11-r125-S5.PDF]

Type	LocusID	Left (5' to 3')	Right (5' to 3')	Amplicon
PCR	79	GTGCAGCTCAGATGCTATGAAT	CTTCCCTCCTTTCAAGTCTGG	2279 bp
PCR	212	CCAAAAAATTAATGAATCTTCCCA	ATGGAATGATGCTTGTGTGTGT	1798 bp
PCR	212	GACCTCAGTCAGAGCGAGAACT	AAATGGGCTCCTCTTAAATGGT	1822 bp
PCR	614	ATGCTAAATTCCATCTGGTGCT	GCCCACCCTCACAAATAAAATA	1432 bp
PCR	756	CATTAGTCAACACAGGGAGCAA	ACTACCCTAAGCTCACCCACAG	1989 bp
PCR	777	TCTCATCAGCAGTGGAAAAGAG	ATCCTCAGACAGTCCTTTCAGC	1382 bp
PCR	932	CACATACAGGTAATTGAAATTTGAGG	TGGCAAAATAAAACCATAAGCA	2270 bp
PCR	954	GCTAGATTGAAAGCCCAAGGTA	ATTCTTGGAAGACCTTGCTTTG	2231 bp
PCR	1017	CAGTTGTGAGAGCGAAAATGAA	CCATTTGCAGGAACATAAACAA	2121 bp
PCR	1105	TGGATAGGTTTGATGATTATTGG	CAATAAACATTTTTAAGTAATCGAACA	1762 bp
PCR	1506	TGTTGGGTGAATTCTTTTCCTT	GGATAAAGTAGCTTCTTCTTTTGCTG	1909 bp
PCR	1565	TTTAAAGCTTAGAGCTTCCCAAT	ATTTTTGTGCCCTTAATGGATG	1349 bp
PCR	1609	TGCTATCCAGCATTGATTTGAG	TTCAAATGGAATGAATTTTCCAC	1395 bp
PCR	2257	CATCCAGAGAGTAAGTTTAGCATCC	AATTTGAAGGTGGAGGGAAAAT	1622 bp
PCR	2364	TGGTAGAGAAACTCCACCCATT	TTTGAATCTTTTTCGGCATTAAA	2042 bp
PCR	2453	TCCAGAAGTGCCAAAATTCTTT	AACTACCTTTCTTTGCATTGCC	1538 bp
PCR	2466	CCATAGCATTGGTCCTTTCTGT	TTCCTCTGTGACTTTGGCAGTA	1545 bp
PCR	2745	GATGGGTGTGTAGTTGCAGAAA	TTTCTTCTTCTCTCATGCGTCA	1403 bp
PCR	2786	GCTTCTTGAGACAGCAATTCTC	TAGGTTTTGCCTGCTTGCTTAT	1158 bp
PCR	2827	TGGAGATGGTGACAGTCTTCTG	TTTGATTTTTCCTTTCCCCTTT	1772 bp
PCR	3157	AGCCCTAAAACCTGAGCTCTCT	CCCACATCCTGTACTTTGTGAA	2328 bp
PCR	3186	GGCACTAGCATTCTAGACCACA	AAACAGGAGATAGGGCATGAAA	1916 bp
PCR	3262	ATCATGACTTTCCTATGGGACC	CATCACCTTGTCTTCAGAGTGC	1161 bp
PCR	3685	TGAATTCCAGTGTGATTTCTGG	GCCTCTGAAAGTTGGAGTGTTT	2679 bp
PCR	3689	TAAGCATGCAAGCCTTGTTTTA	CATTAAAACCCAAAATTTGCTCA	2264 bp
PCR	3734	GGCTTGCTTTTCACTCTATTGC	AGTCCATGTGAAAGGCAATTCT	1466 bp
PCR	3860	CCATCTTTCCAAAGTTGCTACC	CCATCTCTTCCCATGATCTCTC	1909 bp
PCR	3907	GTTTCCATATGGGACCACATTC	GCACAGTCTTCCCACTTCTTTC	2160 bp
PCR	4235	CATTAGGGCTTAGCAGTCAACC	CCCTCTGTAGGGAAAGTGTGTC	1880 bp
PCR	4260	TCCATGAATAGCCCCTTAAAAA	GTACAACATGAGGGGGACAAAT	1446 bp
PCR	4292	CAAAGCATGCCAGTGAGATAGA	TGAAAAGAGCAGATGAGGGTTT	2050 bp
PCR	4448	TTTCATCTTAAATTTAGCTCCGTG	CCATCATCTTCTGCATTTCTCC	1671 bp
PCR	4505	AATCAAAATAATTCCAGGAAGCC	TTCTTGGAAGAGCTTTTGATTTT	1456 bp
PCR	4582	AAGACAAAAAGAAGGTGCCAAA	GGGCAAAGAAAATACTACGTGC	1191 bp
PCR	4617	TTCATTGGGAAAATAAAGCTTACTG	CAAGAAGATATTGGGTGCATCA	1999 bp
PCR	4799	AGACCTGAGTTCCTGTGGTGAT	AGAAGACAGGTAGTGGTCCTGG	2474 bp
PCR	5388	TAGAGTGGGTTAAAGGGCTCTG	ACTTCCCCTGCCTCTCTTCTAC	1455 bp
PCR	5439	TGCAAACGTACACATTAAACAAA	TAAGCAACATTTTCAGACCAGC	1647 bp
PCR	5458	GAAATAAAACGCAAAAGGCTGT	TCTAGCTACGTCTAGCGTCGTG	760 bp
PCR	5537	TTTGTGTCCTTTCCTTCCCTTA	ATATCACCTGGGGTTTTCCTCT	2352 bp
PCR	5682	TCCTAAGAAGAGGACGAGGACA	GTCTGGAAGGTTGTAGGTGGAG	1581 bp
PCR	5936	AAGCACAATATCCAAGGACACC	GGTCCACAGAGATGAAGGACTC	1569 bp
PCR	6041	GCAGGAGGAATAATTTAAGGCA	CAACAAACAATAGTGCCTTCCA	1667 bp
PCR	6049	CCACCCATATATGTACACACACAC	AACAACAATAACAGAAGAAACCCA	1715 bp
qPCR	217	CAGAGAGTGGAGAATCTGTTCATGA	ATTACTTCAGCAACATGTGTCTGCTA	135 bp
qPCR	1231	GCATTGAACTCTTTCCCAAGCT	CTGCTCTCTGTTGAATGCCTTTC	100 bp
qPCR	1302	GATTCATTTCACATCTCAGGCATACT	TAGCACTATGGGAGCTCAGAAGAG	76 bp
qPCR	1701	CTCCCACGTGGCTCTGATG	AACAGCCAGTAGTTTCTTCCCTAATT	136 bp
qPCR	1932	TGTGGGAAAAAGAAGGCTGTAGT	TCACTTTCCTTTCCTGGAACCA	119 bp
qPCR	1950	CCGCTGGTCCTTTGACAATC	CGCTTCCCCACAGAGTTGA	70 bp
qPCR	2012	AGGCCTCAGAGTGGCTTACAGT	TGAGAAGTTGCATTGTGAAGTAAGG	85 bp
qPCR	2065	CTTCCAAAACTACACACAGAGAGGTAA	TGTGGTAATAGCATACACATTTTTCTTG	117 bp
qPCR	2590	GCCTGCTCTGATTAGGGCTCTT	ATGTTGCACCACCAATCTGTCT	83 bp
qPCR	2595	TTTTCTGCCAACACACAGATTAGTAA	TGAGCCACTGAGTCATTGTACATAATAA	86 bp
qPCR	2757	CCCTTCACTTAACTGACATCAGAACA	CCTCATTCTCCCAAGTCACTTCA	76 bp
qPCR	2854	GGTAGAGCCGGGAGCAGAAT	CTATCCATCACTTTAGAACACCAGAGA	74 bp
qPCR	4018	CAGTGTAGTCCTCTATTGTCATGTGGAT	GAGGCATTGACTTCATTTATGATCA	115 bp
qPCR	4799	CTCATCACAATTGCCCATCAA	AGAGGTTCCAGTTGCCTGTCTAA	86 bp
qPCR	4998	CAGAGTCCATCTCAAACGTGGTT	CCCCTTAATTGGGTAGACCTGAT	79 bp
qPCR	5235	TCTGTGCTCTTAGCAGGTGTAACTG	CACTGTTCCTCACCACCTGATG	83 bp
